# Highly selective single and multiple deuteration of unactivated C(sp^3^)-H bonds

**DOI:** 10.1038/s41467-022-31956-3

**Published:** 2022-07-22

**Authors:** Nian Li, Jinhang Li, Mingzhe Qin, Jiajun Li, Jie Han, Chengjian Zhu, Weipeng Li, Jin Xie

**Affiliations:** 1 State Key Laboratory of Coordination Chemistry, Jiangsu Key Laboratory of Advanced Organic Materials, Chemistry and Biomedicine Innovation Center (ChemBIC), School of Chemistry and Chemical Engineering, Nanjing University, Nanjing, 210023 China; 2 State Key Laboratory of Organometallic Chemistry, Shanghai Institute of Organic Chemistry, Shanghai, 200032 China; 3 College of Chemistry and Molecular Engineering, Zhengzhou University, Zhengzhou, 450001 China; 4 State Key Laboratory of Chemistry and Utilization of Carbon Based Energy Resources; College of Chemistry, Xinjiang University, Urumqi, 830017 China

**Keywords:** Synthetic chemistry methodology, Photocatalysis

## Abstract

Selective deuteration of unactivated C(sp^3^)-H bonds is a highly attractive but challenging subject of research in pharmaceutical chemistry, material science and synthetic chemistry. Reported herein is a practical, highly selective and economical efficient hydrogen/deuterium (H/D) exchange of unactivated C(sp^3^)-H bonds by synergistic photocatalysis and hydrogen atom transfer (HAT) catalysis. With the easily prepared PMP-substituted amides as nitrogen-centered radical precursors, a wide range of structurally diverse amides can undergo predictable radical H/D exchange smoothly with inexpensive D_2_O as the sole deuterium source, giving rise to the distal tertiary, secondary and primary C(sp^3^)-H bonds selectively deuterated products in yields of up to 99% and excellent D-incorporations. In addition to precise monodeuteration, this strategy can also achieve multideuteration of the substrates contain more than one remote C(sp^3^)-H bond, which opens a method to address multi-functionalization of distal unactivated C(sp^3^)–H bonds.

## Abstract (web summary)

Deuteration of unactivated C(sp3)-̵̵̵̵̵̵̵–H bonds is a simple route to deuterated compounds, of use in pharmaceutical chemistry, material science, and synthetic chemistry. Here, the authors describe a hydrogen/deuterium (H/D) exchange of remote unactivated C(sp3)-̵̵̵̵̵̵̵–H bonds via photocatalysis, proceeding through hydrogen atom transfer.

## Introduction

**Fig. 1:**
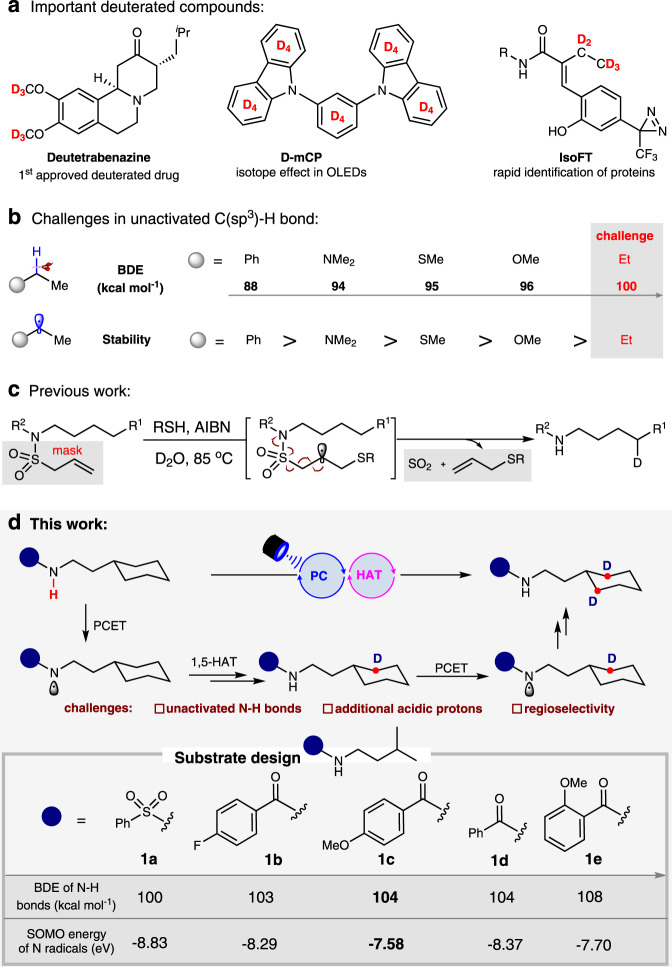
Project background and design plan for radical deuteration of unactivated C(sp^3^) bonds. **a** The significance of deuterated compounds. **b** The BDE of different C(sp^3^)-H bonds and stability of related alkyl radicals. **c** Previous work on N-X bonds cleavage induced deuteration of remote C(sp^3^)-H bonds. **d** This work: Tunable H/D exchange of single or multiple unactivated C(sp^3^)-H bonds.

The precise deuterium labeling technique is of significant value in the investigation of kinetic isotopic effects^1,2^, the discovery of pharmaceutical drugs^3–6^, and material modifications^7^ as well as biochemical techniques^8,9^ (Fig. 1a). In recent decades, the synthesis of deuterated molecules has attracted much attention from organic chemists^10–29^. For instance, our group has developed a practical deoxygenative deuteration of carboxylic acids for the synthesis of D-labeled aldehydes using synergistic catalysis^16^. Among various reliable synthetic methods for deuterium incorporation, the radical hydrogen−deuterium (H/D) exchange strategy of C(sp^3^)-H bonds is one of the most practical and atom-economical methods due to the mild reaction conditions and the excellent functional group compatibility. In 2017 for example, MacMillan et al.^30^ developed an elegant photoredox-catalyzed H/D exchange of α-amino C(sp^3^)–H bonds, and in 2020, Wu et al. reported a visible-light-driven H/D exchange of hydridic C(sp^3^)–H bonds with tetrabutylammonium decatungstate (TBADT)^31^. Notwithstanding these efforts, radical H/D exchange of unactivated C(sp^3^)-H bonds remains highly challenging due to the stronger bond dissociation energy (BDE) of unactivated C(sp^3^)-H bonds compared with C(sp^3^)-H moieties adjacent to an aryl ring or a heteroatom (Fig. 1b).

Very recently, Studer and coworkers^32^ reported an interesting radical monodeuteration of remote unactivated C(sp^3^)-H bonds via 1,5-hydrogen atom transfer (HAT) induced by a nitrogen-centered radical, which is generated in-situ using a stoichiometric of thiol (RSH) in the presence of AIBN at 85 °C (Fig. 1c). This is a powerful method, but the preinstallation of an allylsulfonyl moiety on the nitrogen atom is crucial to successful initiation of the radical rearrangement to generate the N-centered radical and produce 1 equivalent of SO_2_ and allylsulfides as the concomitant products. Thus we questioned if the more challenging free N-H contained amides are competent substrates for radical H/D exchange of remote C(sp^3^)-H bonds through proton-coupled electron transfer (PCET)^33–36^ process. The key challenges to such reactions stem from concerns that the single electron oxidation of strong N-H bonds (100~110 kcal mol^−1^)^37,38^ and the acidic protons in N-H moiety would prevent a high deuterium incorporation from D_2_O.

Herein, we develop a highly selective and efficient hydrogen/deuterium (H/D) exchange of unactivated C(sp^3^)-H bonds by using D_2_O as commercially cheap deuterium source, delivering a wide range of remote tertiary, secondary and primary C(sp^3^)-H bonds deuterated amides in high yields and D-incorporations. Moreover, with this protocol, the precise radical H/D exchange of multiple remote C(sp^3^)-H bonds also has been realized (Fig. 1d).

## Results

### Substrate design

We first performed DFT calculations to estimate the BDEs of N-H bonds with different substituents and the singly occupied molecular orbital (SOMO) energies of the corresponding nitrogen radicals. Interestingly, it was found that different amides have different N-H BDEs and different SOMO energies of nitrogen-centered radicals, which indicates a possibility with which to tune the reactivity towards the PCET and 1,5-HAT processes by synergistic photoredox catalysis. As shown in Fig. 1d, the N-H bond in the PMP-substituted amide (**1c**) has a moderate BDE (~104 kcal mol^−1^) while the corresponding nitrogen-centered radical has the highest SOMO energy (−7.58 eV). According to the polarity-matching rule, this might favor a fast 1,5-HAT process of unactivated C(sp^3^)-H bonds.

### Reaction optimization

Experimental results have shown that the amide **1c** is the most efficient substrate, and can afford the desired D-labeled product (**3c**) in 85% isolated yield with 0.97 D by using D_2_O as the sole D-source (Table 1, entry 1). It was found that the optimal reaction conditions include Ir^III^ (1 mol%) as the photocatalyst, Bu_4_NOP(O)(OBu)_2_ (5 mol%) as base, **2a** (10 mol%) as HAT catalyst and chlorobenzene (PhCl) as solvent. Other substituted amides (**1a**, **1b**, **1d** and **1e**) were also examined under these conditions (entries 2–5) but the D-incorporations of benzenesulfonamide (**1a**), 4-fluorobenzamide (**1b**), benzamide(**1d**) and 2-methoxyphenyl-substituted amide (**1e**) were found to be significantly lower (<0.30 D), this might be caused by the mismatched SOMO energy or strong DBE of the N-H bond. Importantly, base optimization experiments illustrated the unique role of Bu_4_NOP(O)(OBu)_2_ (entries 6 and 7). We envisioned that the phosphate base would accelerate the PCET process by the hydrogen bonding between the amide N-H and the phosphate anion^39^. In addition to the HAT catalyst **2a**, other thiols (**2b** and **2c)** are also good HAT catalysts, which can furnish the target product in good yield with slightly decreased D-incorporation (entries 8 and 9, 0.92 D and 0.95 D respectively). The use of other solvents, such as PhCF_3_ or toluene (entries 10 and 11), can deliver the products in satisfactory results (>95% yields with 0.92 D).

**Table 1:** Optimization of reaction conditions^a^.


Entry	Variation of standard conditions	Yield^b^	D-inc.^c^
1	None	98% (85%)	0.97 D
2	**1a** instead of **1c**	93%	0.26 D
3	**1b** instead of **1c**	92%	0.22 D
4	**1d** instead of **1c**	96%	0.26 D
5	**1e** instead of **1c**	97%	0.27 D
6	K_3_PO_4_ (1.0 equiv.) instead of Bu_4_NOP(O)(OBu)_2_	98%	n.d.
7	KO^*t*^Bu (1.0 equiv.) instead of Bu_4_NOP(O)(OBu)_2_	94%	n.d.
8	**2b** instead of **2a**	98%	0.92 D
9	**2c** instead of **2a**	98%	0.95 D
10	PhCF_3_ instead of PhCl	97%	0.92 D
11	Toluene instead of PhCl	96%	0.92 D


^a^Standard reaction conditions: Ir^III^ (1 mol%), Bu_4_NOP(O)(OBu)_2_ (5 mol%), **2a** (10 mol%), **1c** (0.1 mmol), D_2_O (110 equiv., 0.2 mL), PhCl (2.0 mL), blue LEDs, 24 h, ambient temperature (with a fan to cool), in argon atmosphere. ^b^Measured by GC using biphenyl as internal standard, the isolated yield is given in the parentheses. ^c^Deuterium incorporation was determined by HRMS-ESI and ^1^H NMR spectroscopy. *n.d.* not detected, PhCl chlorobenzene, PhCF_3_ benzotrifluoride.

### Substrate scope

**Fig. 2:**
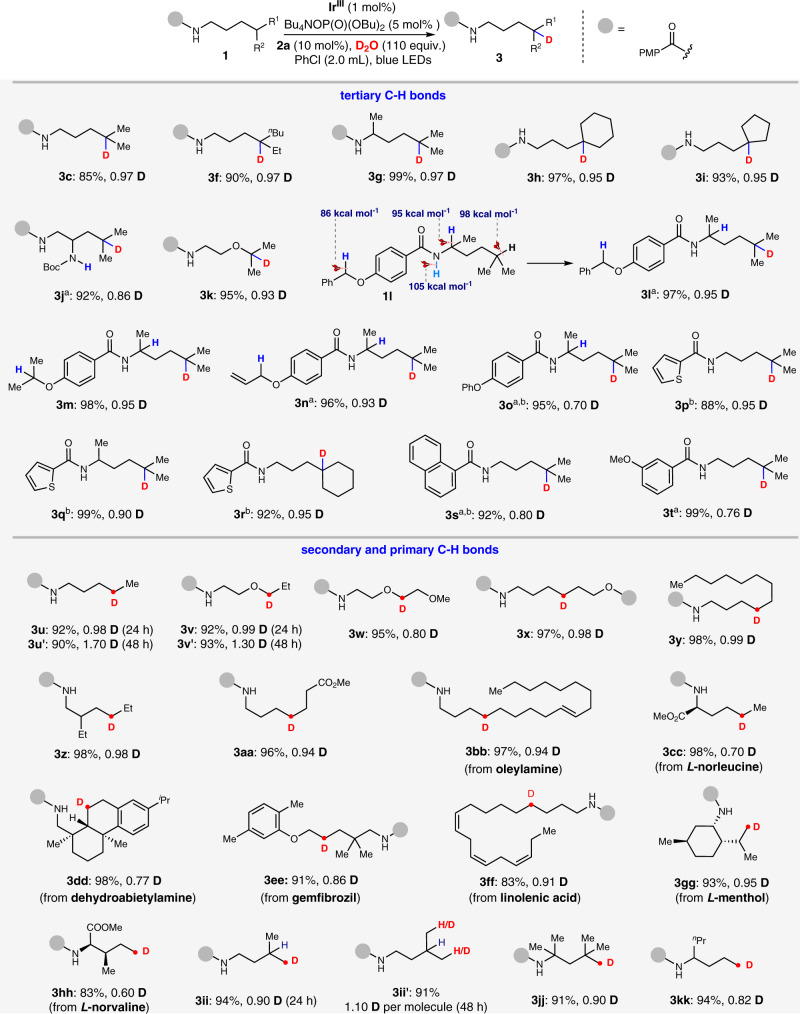
Scope of the radical H/D exchange of C(sp^3^)-H bonds. Reaction conditions: Ir^III^ (1 mol%), Bu_4_NOP(O)(OBu)_2_ (5 mol%), **2a** (10 mol%), amide **1** (0.1 mmol), D_2_O (110 equiv., 0.2 mL), PhCl (2.0 mL), blue LEDs, 24 h, ambient temperature (with a fan to cool), in argon atmosphere. Isolated yields are shown and the deuterium incorporation ratio was determined by ^1^H NMR spectroscopy. PMP = 4-methoxyphenyl. ^a^48 h. ^b^**2c** (10 mol%) instead of **2a**.

To evaluate the functional group generality of this H/D exchange strategy, a variety of structurally diverse amides were subjected to this protocol. In general, the tertiary, secondary and primary C(sp^3^)-H bonds of those amides were smoothly converted to C(sp^3^)-D bonds (**3c**, **3f**–**3kk**) in excellent yields and D-incorporations. As shown in Fig. 2, we first investigated the compatibility of PMP-substituted amides with a remote tertiary C-H bond (**3c**, **3f**–**3k**). The H/D exchange of amides containing five- or six-membered carbocycles (**3** **h**, **3i**), or an oxygen atom (**3k**) worked efficiently (>90% yield, >0.90 D). When the amide (**1j**) has an acidic hydrogen (BocN-H), the D-incorporation of corresponding product (**3j**) was decreased slightly (0.86 D). Apart from PMP-substituted amides, other diverse benzamide derivatives (**1l**–**1t**) were deuterated smoothly as well with a moderate to good D-incorporation of 0.70–0.95 D (**3l**–**3t**). More significantly, the chemoselectivity of this H/D exchange was found to be promising (**3l**–**3o**). Hydridic C(sp^3^)–H bonds such as those in benzylic, allylic, α-amino or α-oxy positions in amides remain intact during radical H/D exchange even though the relevant BDEs are much lower than those of remote unactivated C(sp^3^)–H bonds. Achieving this result by other H/D exchange strategy^31^ is very difficult and further demonstrates the excellent regioselectivity of the 1,5-HAT process.

Compared with tertiary C(sp^3^)-H bonds, the 1,5-HAT of primary and secondary C(sp^3^)-H bonds is more challenging due to the higher BDEs and lower stability of carbon-centered radicals^40–44^. Despite this, the deuteration of the secondary C(sp^3^)-H bonds (**3u**–**3ff** ) proceeded perfectly (>95% yields, >0.70 D) with good functional group (-OMe, -COOMe and alkenyl) tolerance. And primary C(sp^3^)-H bonds are also suitable for this protocol, the remote deuterated products (**3gg-3kk**) were obtained in good yields with acceptable D-incorporations (0.60–0.95 D). Moreover, the derivatives of some complex molecules, such as oleylamine, *L*-norleucine, dehydroabietylamine, gemfibrozil, linolenic acid, *L*-menthol, *L*-norvaline, can undergo this radical H/D exchange smoothly to give the products (**3bb**–**3hh**) with moderate to good D-incorporations. Furthermore, we found that the multiple deuteration products **3** **u’**, **3** **v’** and **3ii’** (a mixture of mono- and di-deuterated compound) of secondary or primary C(sp^3^)-H bonds can be obtained by prolonging the reaction time from 24 h to 48 h.

**Fig. 3:**
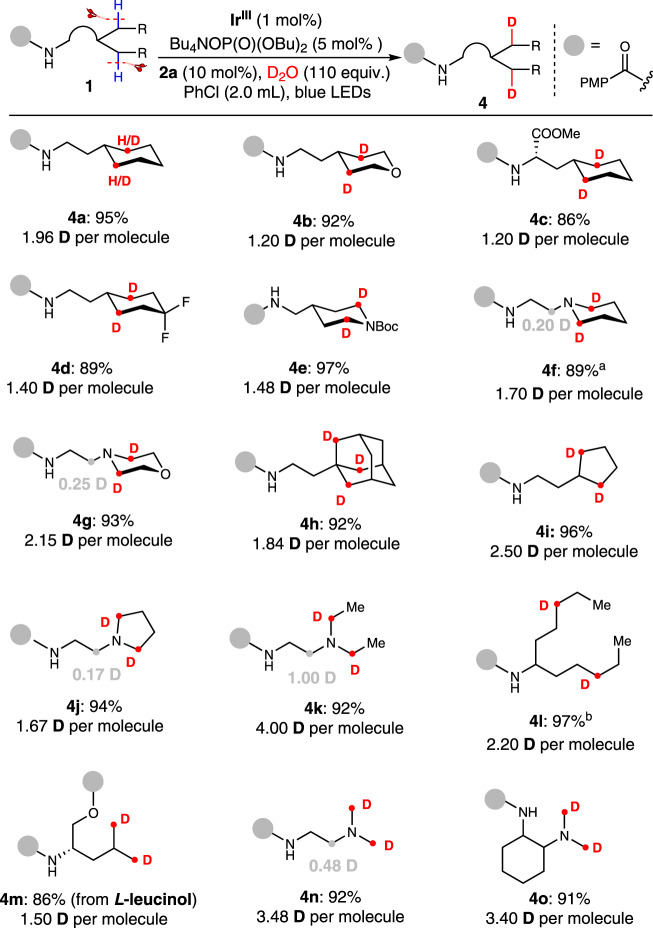
Multi-deuteration of C(sp^3^)-H bonds. Reaction conditions: Ir^III^ (1 mol%), Bu_4_NOP(O)(OBu)_2_ (5 mol%), **2a** (10 mol%), **1** (0.1 mmol), D_2_O (110 equiv., 0.2 mL), PhCl (2.0 mL), blue LEDs, 24 h, ambient temperature (with a fan to cool), in argon atmosphere. Isolated yields are shown and the deuterium incorporation ratio was determined by ^1^H NMR spectroscopy. ^a^12 h. ^b^48 h.

Inspired by the di-deuteration of **1ii**, we wondered that beyond the precise deuteration of one remote C(sp^3^)-H bond, if this 1,5-HAT strategy can achieve the di-deuteration of two remote unactivated C-H bonds. Delightfully, our method successfully realized the multideuteration when the substrate contains more than one remote C(sp^3^)-H bond (Fig. 3), a result that is difficult to achieve with previous method^32^. To investigate the practicality of this finding, a wide range of amides with six-membered rings were examined. Structures containing cyclohexane (**4a**, **4c** and **4d**), tetrahydropyran (**4b**), hexahydropiperidine (**4e**, **4** **f** ) and morpholine (**4** **g**) were successfully multideuterated with 1.20–2.15 D per molecule. The gem-difluoride group (**4d**, 89% yield, 1.40 D per molecule), the ester group (**4c**, 86% yield, 1.20 D per molecule) were entirely compatible. Additionally, the adamantyl (**4** **h**, 92% yield, 1.84 D per molecule), cyclopentyl (**4i**, 96% yield, 2.50 D per molecule) and tetrahydropyrrolyl (**4j**, 94% yield, 1.67 D per molecule) groups performed excellently. Besides cyclic compounds, the acyclic structures including both secondary and primary C(sp^3^)-H bonds were converted to the corresponding multi-deuterated products (**4k-4o**) in excellent yield (86%-97%) and satisfactory D-incorporation (1.50–4.00 D per molecule). This unprecedented process provides an opportunity for the multi-functionalization of distal unactivated C(sp^3^)–H bonds.

### Synthetic application

**Fig. 4:**
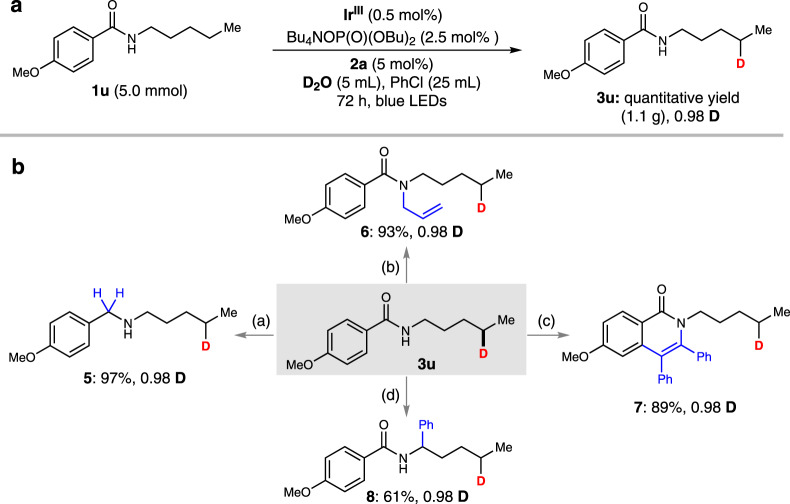
Synthetic application. **a** 5 mmol scale-up experiment. **b** Downstream synthetic transformations and please see Supplementary Information for reaction conditions.

As shown in Fig. 4, when the remote deuteration reaction of **1** **u** was scaled-up to 5.0 mmol, **3** **u** was obtained with quantitative yield and satisfying D-incorporation (0.98 D). There are some promising synthetic potentials of this deuterated amide product (**3** **u**), which is a versatile substrate for downstream transformations to deliver the secondary amine (**5**) and tertiary amide (**6**) in excellent yields. Additionally, the amide group in **3** **u** can be esteemed as a directing group for transition-metal-catalyzed C − H bond functionalization. For example, rhodium-catalyzed C-H activation of **3** **u** can react with diphenyl acetylene successfully to generate the oxidative cycloaddition product (**7**). A photoredox/nickel dual catalyzed C(sp^2^)-C(sp^3^) coupling of **3** **u** with bromobenzene to afford product (**8**) further demonstrated its synthetic robustness of the deuterated amide.

### Mechanism proposal

**Fig. 5:**
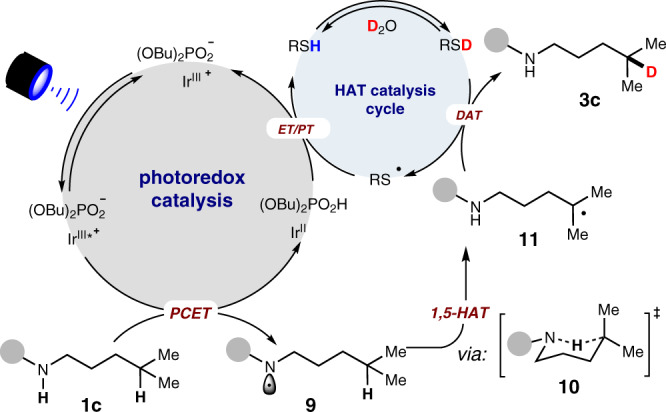
Mechanism proposal. Proposed mechanism for this synergistic catalyzed remote C-H bond deuteration.

As the use of strong base KO^*t*^Bu failed in this deuteration reaction, we exclude the deprotonation-electron transfer pathway^33^ and a plausible mechanism based on our previous work on synergistic catalyzed deuteration^16,17,45–53^ is proposed in Fig. 5. We anticipate that firstly, a non-covalent complex will be formed between the Ir^III^ photocatalyst and Bu_4_NOP(O)(OBu)_2_^54^. The resulting Ir^III^-complex formed in-situ is excited to Ir^III^* by visible light and meanwhile a hydrogen bond is formed from (BuO)_2_PO_2_^−^ and the amide N − H of substrate (**1c**), thus triggering the PCET process^39^ to generate the nitrogen radical (**9**). Then the hydrogen atom of the remote C(sp^3^)-H is transferred to the N-radical and a carbon-centered radical (**11**) is produced via a six-membered ring intermediate (**10**). In a HAT catalytic cycle analogous to our previous work^16,17^, RSD is generated in-situ from RSH and excess D_2_O as a result of the distinction in the pK_a_ values. Subsequently, the nucleophilic alkyl radical (**11**) undergoes deuterium atom transfer (DAT) from RS-D, furnishing the desired product (**3c**). The resulting electrophilic thiyl radical is readily reduced by Ir^II^ to thiol anion and also complete the photocatalytic cycle^39^, and this is followed by a proton transfer from Bu_4_NOPO_2_H (pKa ≈ 12 in MeCN)^55^ to regenerate RSH (pK_a_ ≈ 21 in MeCN for PhSH)^56,57^.

## Discussion

We have developed a highly selective, economic and practical H/D exchange protocol of remote unactivated C(sp^3^)-H bonds with D_2_O as cheap deuderium source by utilzing a synergistic photoredox catalysis and organocatalysis system. With the DFT calculation optimized free N-H contained amides as N-radical precusor to abstract the remote hydrogen, a wide range of remote tertiary, secondary and primary C(sp^3^)-H bonds can be smoothly converted to the corresponding C-D bonds in excellent yields (up to 99%) and high levels of D-incorporation. This protocol can also achieve tunable multideuteration of substrates containing more than one remote C(sp^3^)-H sites, which represents an significant step-forward in 1,5-HAT-induced multi-functionaztion. This report is an important advance in the controllable synthesis of monodeuterated and multideuterated compounds.

## Methods

### General procedure for deuteration

A screw cap dram vial was charged with Ir^III^ (1.1 mg, 1 mol%), tetrabutylammonium dibutyl phosphate (2.3 mg, 5 mol%), thiol catalyst **2a** (2.8 mg, 10 mol%) or **2c** (2.4 mg, 10 mol%), amide (0.1 mmol, 1 equiv), then the vial was delivered to glove box, PhCl (2.0 mL) and D_2_O (0.2 mL) were added. The vial was stirred under the irradiation of blue LEDs at room temperature for the indicated time. After the reaction was finished, the reaction mixture was extracted by ethyl acetate, dried by anhydrous Na_2_SO_4_, filtered and collected the organic layer. The organic solvent was removed under the reduced pressure. The residue was purified by column chromatography on silica gel to obtain the desired products.

## Supplementary information

Supplementary Information

## Data Availability

We declare that all other data supporting the findings of this study are available within the article and Supplementary Information files.

## References

[1] Wiberg KB (1955). The deuterium isotope effect. Chem. Rev..

[2] Scheppele SE (1972). Kinetic isotope effects as a valid measure of structure-reactivity relationships:isotope effects and nonclassical theory. Chem. Rev..

[3] Schmidt C (2017). First deuterated drug approved. Nat. Biotechnol..

[4] Tung RD (2016). Deuterium medicinal chemistry comes of age. Future Med. Chem..

[5] Gant TG (2014). Using Deuterium in Drug Discovery: Leaving the Label in the Drug. J. Med. Chem..

[6] Pirali T, Serafini M, Cargnin S, Genazzani AA (2019). Applications of Deuterium in Medicinal Chemistry. J. Med. Chem..

[7] Liu X (2020). Isotope Effect in the Magneto-Optoelectronic Response of Organic Light-Emitting Diodes Based on Donor–Acceptor Exciplexes. Adv. Mat..

[8] Tomohiro T, Morimoto S, Shima T, Chiba J, Hatanaka Y (2014). An Isotope-Coded Fluorogenic Cross-Linker for High-Performance Target Identification Based on Photoaffinity Labeling. Angew. Chem. Int. Ed..

[9] Atzrodt J, Derdau V, Kerr WJ, Reid M (2018). Deuterium- and Tritium-Labelled Compounds: Applications in the Life Sciences. Angew. Chem. Int. Ed..

[10] Kurimoto A, Sherbo RS, Cao Y, Loo NWX, Berlinguette CP (2020). Electrolytic deuteration of unsaturated bonds without using D_2_. Nat. Catal..

[11] Liu X, Liu R, Qiu J, Cheng X, Li G (2020). Chemical-reductant-free electrochemical deuteration reaction german edition: using deuterium oxide. Angew. Chem. Int. Ed..

[12] Ou W (2021). Room-temperature palladium-catalyzed deuterogenolysis of carbon oxygen bonds towards deuterated pharmaceuticals. Angew. Chem. Int. Ed..

[13] Smith JA (2020). Preparation of cyclohexene isotopologues and stereoisotopomers from benzene. Nature.

[14] Vang ZP (2021). Copper-catalyzed transfer hydrodeuteration of aryl alkenes with quantitative isotopomer purity analysis by molecular rotational resonance spectroscopy. J. Am. Chem. Soc..

[15] Nan X, Wang Y, Li X, Tung C, Wu L (2021). Site-selective D_2_O-mediated deuteration of diaryl alcohols via quantum dots photocatalysis. Chem. Commun..

[16] Zhang M, Yuan XA, Zhu C, Xie J (2019). Deoxygenative deuteration of carboxylic acids with D_2_O. Angew. Chem. Int. Ed..

[17] Li N (2021). A highly selective decarboxylative deuteration of carboxylic acids. Chem. Sci..

[18] Shao T (2019). Photoredox-catalyzed enantioselective α-deuteration of azaarenes with D_2_O. iScience.

[19] Li Y (2021). Organophotocatalytic selective deuterodehalogenation of aryl or alkyl chlorides. Nat. Commun..

[20] Zhang B (2021). Electrocatalytic water-splitting for the controllable and sustainable synthesis of deuterated chemicals. Sci. Bull..

[21] Soulard V, Villa G, Vollmar DP, Renaud P (2017). Radical deuteration with D_2_O: catalysis and mechanistic insights. J. Am. Chem. Soc..

[22] Atzrodt J, Derdau V, Kerr WJ, Reid M (2018). Deuterium- and tritium-labelled compounds: applications in the life sciences. Angew. Chem. Int. Ed..

[23] Zhou R (2019). Visible-light-mediated deuteration of silanes with deuterium oxide. Chem. Sci..

[24] Zhang Y, Ji P, Dong Y, Wei Y, Wang W (2020). Deuteration of formyl groups via a catalytic radical H/D exchange approach. ACS Catal..

[25] Uttry A, Mal S, Gemmeren Mvan (2021). Late-stage *β*‑C(sp^3^)−H deuteration of carboxylic acids. J. Am. Chem. Soc..

[26] Sattler A (2018). Hydrogen/deuterium (H/D) exchange catalysis in alkanes. ACS Catal..

[27] Puleo TR, Strong AJ, Bandar JS (2019). Catalytic α‑selective deuteration of styrene derivatives. J. Am. Chem. Soc..

[28] Geng H (2019). Practical synthesis of C1 deuterated aldehydes enabled by NHC catalysis. Nat. Catal..

[29] Atzrodt J, Derdau V, Fey T, Zimmermann J (2007). The renaissance of H/D exchange. Angew. Chem. Int. Ed..

[30] Loh YY (2017). Photoredox-catalyzed deuteration and tritiation of pharmaceutical compounds. Science.

[31] Kuang Y (2020). Visible light driven deuteration of formyl C–H and hydridic C(sp^3^)–H bonds in feedstock chemicals and pharmaceutical molecules. Chem. Sci..

[32] Wang L, Xia Y, Derdau V, Studer A (2021). Remote Site-Selective Radical C(sp^3^)-H monodeuteration of amides using D_2_O. Angew. Chem. Int. Ed..

[33] John CKC, Rovis T (2016). Amide-directed photoredox-catalysed C–C bond formation at unactivated *sp*^3^ C–H bonds. Nature.

[34] Choi GJ, Zhu Q, Miller DC, Gu CJ, Knowles RR (2016). Catalytic alkylation of remote C–H bonds enabled by proton-coupled electron transfer. Nature.

[35] Gentry EC, Knowles RR (2016). Synthetic applications of proton-coupled electron transfer. Acc. Chem. Res..

[36] Murray PRD (2021). Photochemical and electrochemical applications of proton-coupled electron transfer in organic synthesis. Chem. Rev..

[37] Bordwell FG, Zhang S, Zhang X, Liu W (1995). Homolytic bond dissociation enthalpies of the acidic H-A bonds caused by proximate substituents in sets of methyl ketones, carboxylic esters, and carboxamides related to changes in ground state energies. J. Am. Chem. Soc..

[38] Bordwell FG, Ji G (1991). Effects of structural changes on acidities and homolytic bond dissociation energies of the hydrogen-nitrogen bonds in amidines, carboxamides, and thiocarboxamides. J. Am. Chem. Soc..

[39] Zhu Q, Graff DE, Knowles RR (2018). Intermolecular anti-markovnikov hydroamination of unactivated alkenes with sulfonamides enabled by proton-coupled electron transfer. J. Am. Chem. Soc..

[40] Wu X, Zhu C (2020). Radical Functionalization of Remote C(sp^3^)–H bonds mediated by unprotected alcohols and amides. CCS Chem..

[41] Guo W, Wang Q, Zhu J (2021). Visible light photoredox-catalysed remote C–H functionalisation enabled by 1,5-hydrogen atom transfer (1,5-HAT). Chem. Soc. Rev..

[42] Liu L, Duan X, Guo L (2021). Recent Advance in Iminyl Radical Triggered C–H and C–C bond functionalization of oxime esters via 1,5-HAT and -carbon scission. Synthesis.

[43] Chen H, Yu S (2020). Remote C–C bond formation via visible light photoredox-catalyzed intramolecular hydrogen atom transfer. Org. Biomol. Chem..

[44] Lu Q, Glorius F (2017). Radical enantioselective C(sp^3^)−H functionalization. Angew. Chem. Int. Ed..

[45] Xu W (2018). Synergistic catalysis for the umpolung trifluoromethylthiolationof tertiary ethers. Angew. Chem. Int. Ed..

[46] Zhu C (2020). Photoredox-controlled b-regioselective radical hydroboration of activated alkenes with nhc-boranes. Angew. Chem. Int. Ed..

[47] Xu W, Wang W, Liu T, Xie J, Zhu C (2019). Late-stage trifluoromethylthiolation of benzylic C-H bonds. Nat. Commun..

[48] Zhou N, Yuan X-A, Zhao Y, Xie J, Zhu C (2018). Synergistic photoredoxcatalysis and organocatalysis for inverse hydroboration of imines. Angew. Chem. Int. Ed..

[49] Zhang M, Xie J, Zhu C (2018). A general deoxygenation approach for synthesis of ketones from aromatic carboxylic acids and alkenes. Nat. Commun..

[50] Ruzi R, Liu K, Zhu C, Xie J (2020). Upgrading ketone synthesis direct from carboxylic acids and organohalides. Nat. Commun..

[51] Zhao C, Xia S, Wang C, Wang W, Xie J (2022). Opportunities and challenges of visible light-driven triple synergistic catalysis. Chem. Catal..

[52] Ning Y (2021). Site-specific umpolung amidation of carboxylic acids via triplet synergistic catalysis. Nat. Commun..

[53] Li Y (2022). Highly selective synthesis of all-carbon tetrasubstituted alkenes by deoxygenative alkenylation of carboxylic acids. Nat. Commun..

[54] Morton CM (2019). C−H alkylation via multisite-proton-coupled electron transfer of an aliphatic C−H bond. J. Am. Chem. Soc..

[55] Rueping M, Nachtsheim BJ, Ieawsuwan W, Atodiresei I (2011). Modulating the acidity: highly acidic Brønsted acids in asymmetric catalysis. Angew. Chem. Int. Ed..

[56] Bordwell FG, Hughes DLJ (1982). Thiol acidities and thiolate ion reactivities toward butyl chloride in dimethyl sulfoxide solution. The question of curvature in Broensted plots. Org. Chem..

[57] Chantooni MK, Kolthoff IM (1976). Comparison of substituent effects on dissociation and conjugation of phenols with those of carboxylic acids in acetonitrile, N,N-dimethylformamide, and dimethyl sulfoxide. J. Phys. Chem..

