# Supplementary material for: Highly selective single and multiple deuteration of unactivated C(sp3)-H bonds
Source: Nat Commun. 2022 Jul 22;13:4224. doi: 10.1038/s41467-022-31956-3 (PMC9307835; doi:10.1038/s41467-022-31956-3)
Supplement: Supplementary file 1 — Supplementary Information [file 41467_2022_31956_MOESM1_ESM.pdf]

Supplementary Information

Highly Selective Single and Multiple Deuteration of Unactivated C(sp^3)-H Bonds

Li et al.

Supplementary Methods

1. General information.

All solvents and chemicals were obtained from commercial suppliers and used without further purification. Flash column chromatographic purification of products was accomplished using forced-flow chromatography on Silica Gel (200-300 mesh). ^{1}H NMR, ^{13}C NMR and ^{19}F NMR spectra were recorded on a 400 MHz spectrometer in $CDCl_3$ (δ_H = 7.26 ppm, δ_C = 77.00 ppm as standard). Data for ^{1}H NMR are reported as follows: chemical shift (ppm, scale), multiplicity, coupling constant (Hz), and integration. Data for ^{13}C NMR are reported in terms of chemical shift (ppm, scale), multiplicity, and coupling constant (Hz). GC yield were performed on Nexis GC-2030. High-resolution mass spectra were obtained by ESI on a TOF mass analyzer. The blue LEDs were purchased from Kessil.

2. Optimization of reaction conditions.

Supplementary Table 1. Deuteration of tertiary C(sp^3)-H bonds[a].

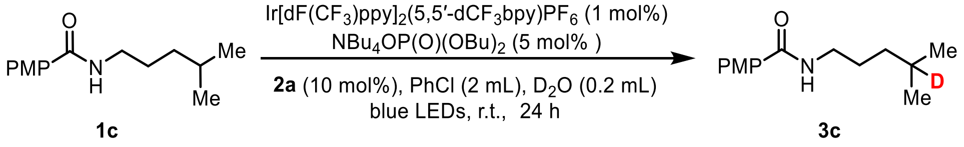

Entry	Variation of standard conditions	Yield[b]	D-inc.[c]
1	None	98% (85%)	0.97 D
2	Ir[dF(CF_3)ppy]$_2$(dtbbpy)PF_6	97%	n.d.
3	K_3PO_4 (1.0 equiv.) instead of $NBu_4OP(O)(OBu)_2$	98%	n.d.
4	KO^{t}Bu (1.0 equiv.) instead of $NBu_4OP(O)(OBu)_2$	94%	n.d.
5	TMG (1.0 equiv.) instead of $NBu_4OP(O)(OBu)_2$	88%	n.d.
6	collidine (1.0 equiv.) instead of $NBu_4OP(O)(OBu)_2$	96%	n.d.
7	**2b** instead of **2a**	98%	0.92 D
8	**2c** instead of **2a**	98%	0.95 D
9	$PhCF_3$ instead of PhCl	97%	0.92 D
10	PhMe instead of PhCl	96%	0.92 D
11	DMF instead of PhCl	95%	n.d.
12	DCE instead of PhCl	92%	0.07 D
13	Without photocatalyst or light	98%	n.d
14	Without **2a**	96%	n.d
15	Without $NBu_4OP(O)(OBu)_2$	95%	n.d

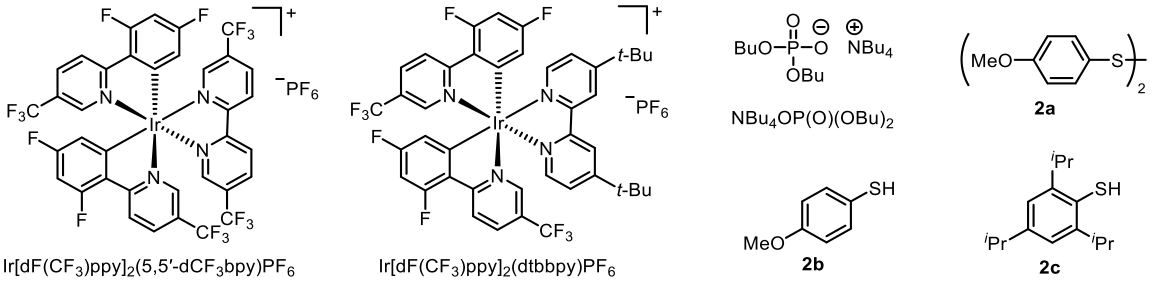

[a]Reaction conditions: **PC** (1 mol%), $NBu_4OP(O)(OBu)_2$ (5 mol%), **2a** (10 mol%), **1c** (0.1 mmol), D_2O (0.2 mL), PhCl (2 mL), blue LEDs, 24 h. [b]Measured by GC using biphenyl as internal standard, the isolated yield was given in the parentheses. [c]Deuterium incorporation was determined by HRMS-ESI and ^{1}H NMR. TMG = *N,N'*-Tetramethylguanidine, DCE = 1,2-dichloroethane, DMF = Dimethylformamide, n.d. = not detected. **1c** was synthesized according to **GP**.

Supplementary Table 2. Deuteration of secondary C(sp^3)-H bonds[a].

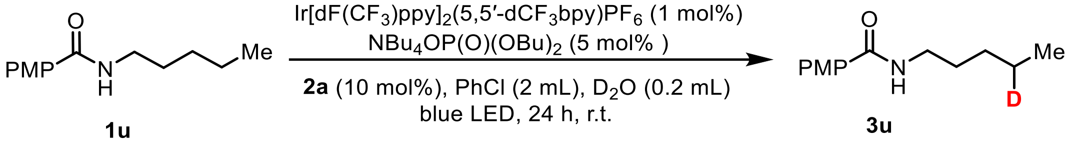

Entry	Variation of standard conditions	Yield[b]	D-inc.[c]
1	None	(99%)92%	0.98 D
2	**2b** instead of **2a**	92%	0.75 D
3	**2c** instead of **2a**	97%	0.20 D
4	**2d** instead of **2a**	96%	0.50 D
5	**2e** instead of **2a**	98%	0.48 D
6	**2f** instead of **2a**	93%	0.32 D
7	**2g** instead of **2a**	95%	0.28 D
8	**2h** instead of **2a**	96%	0.22 D
9	**2i** instead of **2a**	96%	0.21 D
10	**2j** – **2o** instead of **2a**	96%	n.d.
11	48 h instead of 24 h	90%	1.7 D
12	Double portion of catalysts[d]	93%	1.8 D

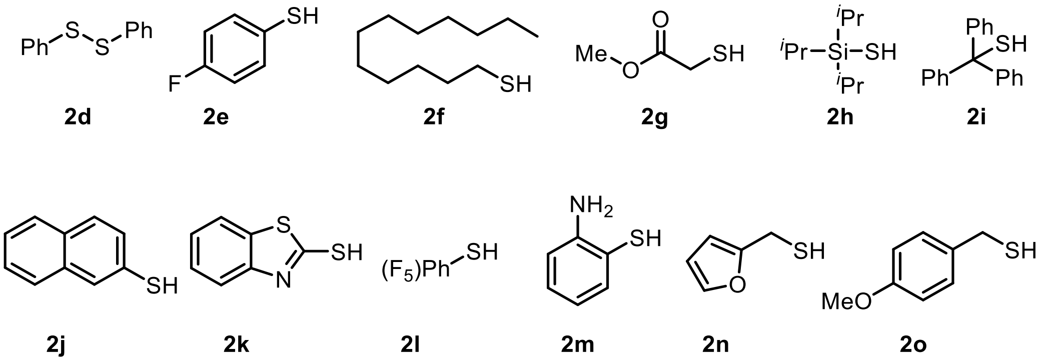

[a]Reaction conditions: **PC** (1 mol%), $NBu_4OP(O)(OBu)_2$ (5 mol%), **2a** (10 mol%), **1u** (0.1 mmol), D_2O (0.2 mL), PhCl (2 mL), blue LEDs, 24 h. [b]Measured by GC using biphenyl as internal standard, the isolated yield was given in the parentheses. [c]Deuterium incorporation was determined by HRMS-ESI and ^{1}H NMR. n.d. = not detected. **1u** was synthesized according to **GP**. [d]**PC** (2 mol%), $NBu_4OP(O)(OBu)_2$ (10 mol%), **2a** (20 mol%), 24 h.

3. General procedure for the deuteration of remote C(sp^3)-H bonds.

3.1 Reaction set-up.

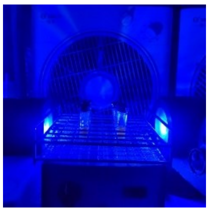

Supplementary Fig 1. Reaction set-up.

(the fan was used to keep the reaction temperatre is around ambient temperature)

3.2 General procedure.

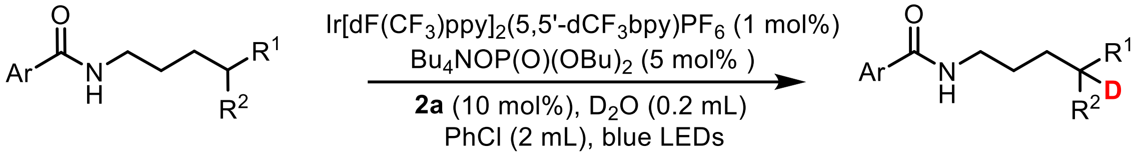

A screw cap dram vial was charged with Ir[dF(CF_3)ppy)]$_2$(5,5'-dCF_3bpy)PF_6 (1.1 mg, 1 mol%), tetrabutylammonium dibutyl phosphate (2.3 mg, 5 mol%), thiol catalyst **2a** (2.8 mg, 10 mol%) or **2c** (2.4 mg, 10 mol%), amide (0.1 mmol, 1 equiv), then the vial was delivered to glove box, PhCl (2.0 mL) and D_2O (0.2 mL) were added. The vial was stirred under the irradiation of two blue LEDs at room temperature for the indicated time. After the reaction was finished, the reaction mixture was extracted by ethyl acetate, dried by anhydrous Na_2SO_4, filtered and collected the organic layer. The organic solvent was removed under the reduced pressure. The residue was purified by column chromatography on silica gel to obtain the desired products.

3.3 Characterization data.

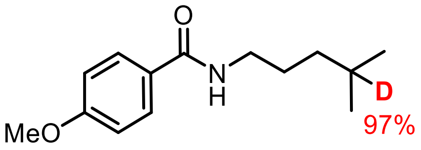

4-methoxy-N-(4-methylpentyl-4-d)benzamide (**3c**)

The title compound was prepared according to **GP**. After purified by column chromatography on silica gel (eluent: petroleum ether/EtOAc =5:1), the title compound (20.1 mg) was obtained in 85% yield as light yellow solid (mp: 89-91 °C).

^{1}H NMR (400 MHz, Chloroform-*d*) δ 7.77 – 7.68 (m, 2H), 6.95 – 6.86 (m, 2H), 6.10 (s, 1H), 3.84 (s, 3H), 3.41 (td, *J* = 7.2, 5.7 Hz, 2H), 1.64 – 1.55 (m, 2H), 1.27 – 1.22 (m, 2H), 1.64 – 1.55 (m, 2.03H, **0.97D**), 1.27 – 1.22 (m, 2H), 0.88 (s, 6H). ^{13}C NMR (101 MHz, Chloroform-*d*) δ 167.0, 162.0, 128.6, 127.1, 113.7, 55.4, 40.3, 36.0, 27.6, **27.3 (t, *J* = 19.2 Hz)**, 22.4. IR (ATR): ν = 3317, 2954, 2865, 1632, 1257, 765, 750 cm^{-1}. HRMS (ESI): m/z Calculated for $C_{14}H_{21}DNO_2^+$ [M+H$^+$]: 237.1708, found 237.1703.

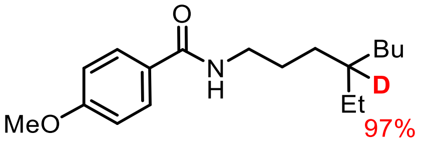

N-(4-ethyloctyl-4-d)-4-methoxybenzamide (**3f**)

The title compound was prepared according to **GP**. After purified by column chromatography on silica gel (eluent: petroleum ether/EtOAc =5:1), the title compound (27.1 mg) was obtained in 90% yield as colorless oil.

^{1}H NMR (400 MHz, Chloroform-*d*) δ 7.76 – 7.67 (m, 2H), 6.95 – 6.87 (m, 2H), 6.15 (d, *J* = 5.9 Hz, 1H), 3.83 (s, 3H), 3.40 (td, *J* = 7.2, 5.7 Hz, 2H), 1.63 – 1.51 (m, 2H), 1.32 – 1.24 (m, 6H), 1.24 – 1.19 (m, 4.03H, **0.97D**), 0.87 (t, *J* = 6.9 Hz, 3H), 0.82 (t, *J* = 7.4 Hz, 3H). ^{13}C NMR (101 MHz, Chloroform-*d*) δ 167.0, 162.0, 128.6, 127.1, 113.7, 55.3, 40.4, **38.0 (t, *J* = 18.7 Hz)**, 32.6, 30.3, 28.9, 26.9, 25.6, 23.1, 14.1, 10.8. IR (ATR): ν = 3007, 2959, 2931, 1632, 1276, 1260, 762, 750 cm^{-1}. HRMS (ESI): m/z Calculated for $C_{18}H_{29}DNO_2^+$ [M+H$^+$]: 293.2334, found 293.2328.

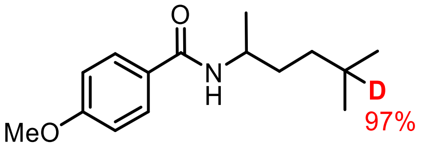

4-methoxy-N-(5-methylhexan-2-yl-5-d)benzamide (**3g**)

The title compound was prepared according to **GP**. After purified by column chromatography on silica gel (eluent: petroleum ether/EtOAc =5:1), the title compound (25.7 mg) was obtained in 99% yield as light yellow solid (mp: 85-88 °C).

^{1}H NMR (400 MHz, Chloroform-d) δ 7.80 – 7.62 (m, 2H), 6.98 – 6.81 (m, 2H), 5.91 (d, J = 8.2 Hz, 1H), 4.21 – 4.04 (m, 1H), 3.83 (s, 3H), 1.56 – 1.44 (m, 2.03H, **0.97D**), 1.28 – 1.22 (m, 2H), 1.21 (d, J = 6.5 Hz, 3H), 0.86 (s, 6H). ^{13}C NMR (101 MHz, Chloroform-*d*) δ 166.2, 161.9, 128.5, 127.3, 113.6, 55.3, 45.8, 35.0, 34.8, **27.4 (t, *J* = 19.1 Hz)**, 22.4, 22.4, 21.1. IR (ATR): ν = 3305, 3005, 2958, 1629, 1276, 764, 750 cm^{-1}. HRMS (ESI): m/z Calculated for $C_{15}H_{23}DNO_2^+$ [M+H$^+$]: 251.1864, found 251.1860.

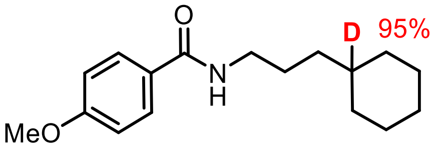

N-(3-(cyclohexyl-1-d)propyl)-4-methoxybenzamide (**3h**)

The title compound was prepared according to **GP**. After purified by column chromatography on silica gel (eluent: petroleum ether/EtOAc =5:1), the title compound (26.8 mg) was obtained in 97% yield as light yellow solid (mp: 110-116 °C).

^{1}H NMR (400 MHz, Chloroform-*d*) δ 7.79 – 7.61 (m, 2H), 6.98 – 6.82 (m, 2H), 6.17 (s, 1H), 3.83 (s, 3H), 3.39 (td, *J* = 7.3, 5.7 Hz, 2H), 1.72 – 1.54 (m, 7H), 1.25 – 1.05 (m, 5.05H, **0.95D**), 0.92 – 0.79 (m, 2H). ^{13}C NMR (101 MHz, Chloroform-*d*) δ 167.0, 162.0, 128.6, 127.1, 113.6, 55.3, 40.3, **36.8 (t, *J* = 18.8 Hz)**, 34.6, 33.2, 27.0, 26.6, 26.3. IR (ATR): ν = 3316, 2920, 2848, 1630, 1607, 1254, 766, 750 cm^{-1}. HRMS (ESI): m/z Calculated for $C_{17}H_{25}DNO_2^+$ [M+H$^+$]: 277.2021, found 277.2012.

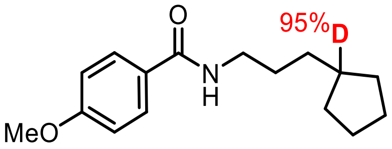

N-(3-(cyclopentyl-1-d)propyl)-4-methoxybenzamide (**3i**)

The title compound was prepared according to **GP**. After purified by column chromatography on silica gel (eluent: petroleum ether/EtOAc =5:1), the title compound (25.2 mg) was obtained in 93% yield as light yellow solid (mp: 80-82 °C).

^{1}H NMR (400 MHz, Chloroform-*d*) δ 7.77 – 7.69 (m, 2H), 6.95 – 6.83 (m, 2H), 6.22 (s, 1H), 3.82 (s, 3H), 3.40 (td, *J* = 7.2, 5.7 Hz, 2H), 1.79 – 1.67 (m, 2.05H, **0.95D**), 1.63 – 1.53 (m, 4H), 1.53 – 1.41 (m, 2H), 1.38 – 1.32 (m, 2H), 1.10 – 1.00 (m, 2H). ^{13}C NMR (101 MHz, Chloroform-*d*) δ 167.13, 162.11, 128.73, 127.26, 113.77, 55.48, 40.38, **39.45 (t, *J* = 19.3 Hz)**, 33.42, 32.66, 29.05, 25.27. IR (ATR): ν = 3314, 2943, 2862, 1631, 1505, 1276, 765, 750 cm^{-1}. HRMS (ESI): m/z Calculated for $C_{16}H_{23}DNO_2^+$ [M+H$^+$]: 263.1864, found 263.1859.

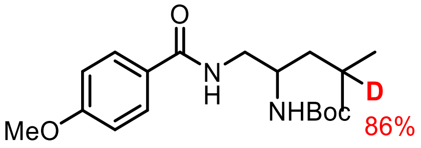

tert-butyl (1-(4-methoxybenzamido)-4-methylpentan-2-yl)carbamate (**3j**)

The title compound was prepared according to **GP**. After purified by column chromatography on silica gel (eluent: petroleum ether/EtOAc =3:1 to 1:1), the title compound (32.3 mg) was obtained in 92% yield as light yellow solid (mp: 130-135 °C).

^{1}H NMR (400 MHz, Chloroform-*d*) δ 7.77 (d, *J* = 8.5 Hz, 2H), 7.18 (s, 1H), 6.88 (d, *J* = 8.7 Hz, 2H), 4.62 (d, *J* = 8.4 Hz, 1H), 3.93 – 3.84 (m, 1H), 3.83 (s, 3H), 3.42 (t, *J* = 5.8 Hz, 2H), 1.77 – 1.67 (m, 0.14H, **0.86D**), 1.38 (s, 9H), 1.37 – 1.28 (m, 2H), 0.98 – 0.82 (m, 6H). ^{13}C NMR (101 MHz, Chloroform-*d*) δ 167.2, 162.0, 157.2, 128.8, 126.6, 113.5, 79.7, 55.3, 48.7, 46.8, 41.9, 28.3, 24.8, **24.4 (t, *J* = 20.1 Hz)**, 22.9, 22.0. IR (ATR): ν = 3325, 2955, 1687, 1276, 1259, 764, 750 cm^{-1}. HRMS (ESI): m/z Calculated for $C_{19}H_{30}DN_2O_4^+$ [M+H$^+$]: 352.2341, found 352.2338.

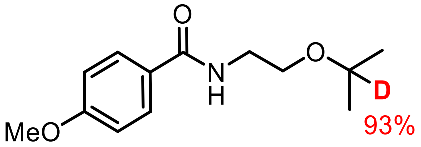

4-methoxy-N-(2-((propan-2-yl-2-d)oxy)ethyl)benzamide (**3k**)

The title compound was prepared according to **GP**. After purified by column chromatography on silica gel (eluent: petroleum ether/EtOAc =3:1), the title compound (22.6 mg) was obtained in 95% yield as light yellow solid (mp: 69-71 °C).

^{1}H NMR (400 MHz, Chloroform-*d*) δ 7.80 – 7.67 (m, 2H), 6.95 – 6.86 (m, 2H), 6.50 (s, 1H), 3.83 (s, 3H), 3.63 – 3.55 (m, 4.07H, **0.93D**), 1.16 (s, 6H). ^{13}C NMR (101 MHz, Chloroform-*d*) δ 166.9, 162.0, 128.6, 126.9, 113.7, **71.4 (t, *J* = 21.8 Hz)**, 66.6, 55.3, 40.0, 21.9. IR (ATR): ν = 3328, 2970, 2933, 1633, 1607, 1504, 1255, 766, 750 cm^{-1}. HRMS (ESI): m/z Calculated for $C_{13}H_{19}DNO_3^+$ [M+H$^+$]: 239.1500, found 239.1495.

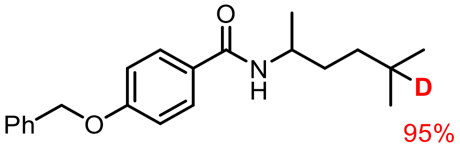

4-(benzyloxy)-N-(5-methylhexan-2-yl-5-d)benzamide (**3l**)

The title compound was prepared according to **GP**. After purified by column chromatography on silica gel (eluent: petroleum ether/EtOAc =5:1), the title compound (31.8 mg) was obtained in 97% yield as light yellow solid (mp: 117-119 °C).

^{1}H NMR (400 MHz, Chloroform-*d*) δ 7.76 – 7.69 (m, 2H), 7.45 – 7.36 (m, 4H), 7.36 – 7.30 (m, 1H), 7.01 – 6.95 (m, 2H), 5.88 (d, *J* = 8.4 Hz, 1H), 5.10 (s, 2H), 4.20 – 4.09 (m, 1H), 1.57 – 1.49 (m, 2.05H, **0.95D**), 1.29 – 1.23 (m, 2H), 1.23 – 1.20 (m, 3H), 0.87 (s, 6H). ^{13}C NMR (101 MHz, Chloroform-*d*) δ 166.2, 161.1, 136.4, 128.6, 128.5, 128.1, 127.6, 127.4, 114.6, 70.0, 45.8, 35.0, 34.9, **27.5 (t, *J* = 19.0 Hz)**, 22.4, 21.1. IR (ATR): ν = 3304, 2962, 2931, 2867, 1629, 764, 747 cm^{-1}. HRMS (ESI): m/z Calculated for $C_{21}H_{27}DNO_2^+$ [M+H$^+$]: 327.2177, found 327.2170.

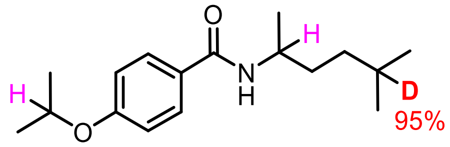

4-isopropoxy-N-(5-methylhexan-2-yl-5-d)benzamide (**3m**)

The title compound was prepared according to **GP**. After purified by column chromatography on silica gel (eluent: petroleum ether/EtOAc =5:1), the title compound (28.0 mg) was obtained in 98% yield as light yellow solid (mp: 69-73 °C).

^{1}H NMR (400 MHz, Chloroform-d) δ 7.75 – 7.64 (m, 2H), 6.95 – 6.83 (m, 2H), 5.85 (d, J = 8.5 Hz, 1H), 4.67 – 4.53 (m, 1H), 4.22 – 4.07 (m, 1H), 1.56 – 1.46 (m, 2.05H, **0.95D**), 1.34 (d, J = 6.1 Hz, 6H), 1.26 – 1.22 (m, 2H), 1.21 (d, J = 6.63 Hz, 3H), 0.86 (s, 6H). ^{13}C NMR (101 MHz, Chloroform-*d*) δ 166.3, 160.4, 128.5, 126.9, 115.2, 69.9, 45.8, 35.0, 34.9, **27.5 (t, *J* = 19.2 Hz)**, 22.4, 21.9, 21.1. IR (ATR): ν = 3307, 2956, 2929, 2866, 1628, 1249, 767, 750 cm^{-1}. HRMS (ESI): m/z Calculated for $C_{17}H_{27}DNO_2^+$ [M+H$^+$]: 279.2177, found 279.2170.

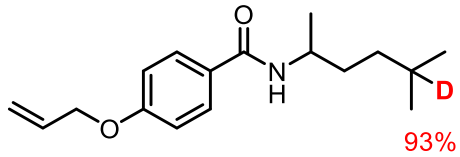

4-(allyloxy)-N-(5-methylhexan-2-yl-5-d)benzamide (**3n**)

The title compound was prepared according to **GP**. After purified by column chromatography on silica gel (eluent: petroleum ether/EtOAc =5:1), the title compound (26.5 mg) was obtained in 96% yield as light yellow solid (mp: 76-78 °C).

^{1}H NMR (400 MHz, Chloroform-*d*) δ 7.75 – 7.64 (m, 2H), 6.97 – 6.86 (m, 2H), 6.10 – 5.97 (m, 1H), 5.85 (d, *J* = 8.4 Hz, 1H), 5.48 – 5.37 (m, 1H), 5.34 – 5.23 (m, 1H), 4.57 (dt, *J* = 5.3, 1.6 Hz, 2H), 4.20 – 4.07 (m, 1H), 1.57 – 1.48 (m, 2.07H, **0.93D**), 1.28 – 1.17 (m, 5H), 0.92 – 0.80 (m, 6H). ^{13}C NMR (101 MHz, Chloroform-*d*) δ 166.3, 160.9, 132.7, 128.5, 127.4, 118.0, 114.4, 68.8, 45.8, 35.0, 34.8, **27.4 (t, *J* = 19.0 Hz)**, 22.4, 21.1. IR (ATR): ν = 3303, 2956, 1628, 1275, 1262, 764, 750 cm^{-1}. HRMS (ESI): m/z Calculated for $C_{17}H_{25}DNO_2^+$ [M+H$^+$]: 277.2021, found 277.2016.

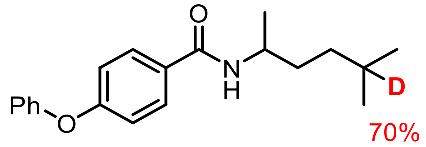

N-(5-methylhexan-2-yl-5-d)-4-phenoxybenzamide (**3o**)

The title compound was prepared according to **GP**. After purified by column chromatography on silica gel (eluent: petroleum ether/EtOAc =5:1), the title compound (29.8 mg) was obtained in 95% yield as light yellow solid (mp: 98-100 °C).

^{1}H NMR (400 MHz, Chloroform-*d*) δ 7.79 – 7.68 (m, 2H), 7.41 – 7.31 (m, 2H), 7.19 – 7.11 (m, 1H), 7.08 – 7.00 (m, 2H), 7.01 – 6.96 (m, 2H), 5.96 (d, *J* = 8.4 Hz, 1H), 4.36 – 3.93 (m, 1H), 1.57 – 1.48 (m, 2.30H, **0.70D**), 1.28 – 1.20 (m, 5H), 0.88 (s, 6H). ^{13}C NMR (101 MHz, Chloroform-*d*) δ 166.1, 160.1, 156.0, 129.9, 129.5, 128.7, 124.1, 119.6, 117.8, 46.0, 35.0, 34.8, **27.4 (t, *J* = 19.2 Hz)**, 22.4, 21.0. IR (ATR): ν = 3296, 2956, 2929, 1630, 1242, 764, 750 cm^{-1}. HRMS (ESI): m/z Calculated for $C_{20}H_{25}DNO_2^{+}$ [M+H^{+}]: 313.2021, found 313.2016.

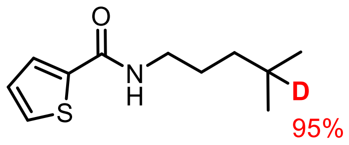

N-(4-methylpentyl-4-d)thiophene-2-carboxamide (**3p**)

The title compound was prepared according to **GP**. After purified by column chromatography on silica gel (eluent: petroleum ether/EtOAc =5:1), the title compound (18.5 mg) was obtained in 88% yield as colorless oil.

^{1}H NMR (400 MHz, Chloroform-*d*) δ 7.50 (dd, *J* = 3.7, 1.2 Hz, 1H), 7.44 (dd, *J* = 5.0, 1.2 Hz, 1H), 7.05 (dd, *J* = 5.0, 3.7 Hz, 1H), 6.17 (s, 1H), 3.39 (td, *J* = 7.3, 5.8 Hz, 2H), 1.63 – 1.54 (m, 2.05H, **0.95D**), 1.26 – 1.21 (m, 2H), 0.87 (s, 6H). ^{13}C NMR (101 MHz, Chloroform-*d*) δ 161.8, 139.2, 129.6, 127.8, 127.5, 40.3, 35.9, 27.5, **27.3 (t, *J* = 19.0 Hz)**, 22.4. IR (ATR): ν = 3303, 2955, 1625, 1551, 1276, 1261, 764, 750 cm^{-1}. HRMS (ESI): m/z Calculated for $C_{11}H_{16}DNOS^{+}$ [M+H^{+}]: 213.1165, found 213.1165.

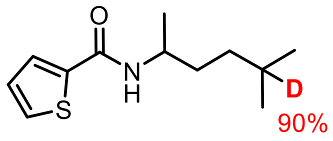

N-(5-methylhexan-2-yl-5-d)thiophene-2-carboxamide (**3q**)

The title compound was prepared according to **GP**. After purified by column chromatography on silica gel (eluent: petroleum ether/EtOAc =10:1), the title compound (22.6 mg) was obtained in 99% yield as light yellow solid (mp: 85-87 °C).

^{1}H NMR (400 MHz, Chloroform-*d*) δ 7.49 (dd, *J* = 3.7, 1.2 Hz, 1H), 7.44 (dd, *J* = 5.0, 1.2 Hz, 1H), 7.05 (dd, *J* = 5.0, 3.7 Hz, 1H), 5.86 (d, *J* = 8.5 Hz, 1H), 4.21 – 4.01 (m, 1H), 1.57 – 1.46 (m, 2.10H, **0.90D**), 1.26 – 1.19 (m, 5H), 0.86 (s, 6H). ^{13}C NMR (101 MHz, Chloroform-*d*) δ 161.1, 139.5, 129.5, 127.6, 127.5, 46.1, 35.0, 34.8, **27.4 (t, *J* = 19.1 Hz)**, 22.4, 21.0. IR (ATR): ν = 3302, 2956, 2929,

2866, 1620, 1548, 764, 750 cm^{-1}. HRMS (ESI): m/z Calculated for $C_{12}H_{19}DNOS^+$ [M+H^+]: 227.1323, found 227.1321.

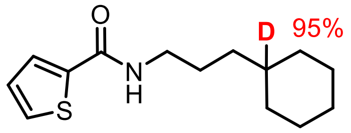

N-(3-(cyclohexyl-1-d)propyl)thiophene-2-carboxamide (**3r**)

The title compound was prepared according to **GP**. After purified by column chromatography on silica gel (eluent: petroleum ether/EtOAc =5:1), the title compound (23.2 mg) was obtained in 92% yield as white solid (mp: 85-91 °C).

^{1}H NMR (400 MHz, Chloroform-d) δ 7.50 (dd, J = 3.7, 1.2 Hz, 1H), 7.44 (dd, J = 5.0, 1.2 Hz, 1H), 7.05 (dd, J = 5.0, 3.7 Hz, 1H), 6.16 (t, J = 5.6 Hz, 1H), 3.38 (td, J = 7.3, 5.8 Hz, 2H), 1.73 – 1.54 (m, 7H), 1.25 – 1.06 (m, 5.05H, **0.95D**), 0.94 – 0.78 (m, 2H). ^{13}C NMR (101 MHz, Chloroform-*d*) δ 161.8, 139.2, 129.5, 127.8, 127.5, 40.3, **36.8 (t, *J* = 18.8 Hz)**, 34.5, 33.2, 27.0, 26.6, 26.3. IR (ATR): ν = 3305, 2920, 2848, 1622, 1549, 1261, 764, 750 cm^{-1}. HRMS (ESI): m/z Calculated for $C_{14}H_{21}DNOS^+$ [M+H^+]: 253.1479, found 253.1470.

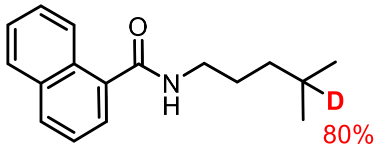

N-(4-methylpentyl-4-d)-1-naphthamide (**3s**)

The title compound was prepared according to **GP**. After purified by column chromatography on silica gel (eluent: petroleum ether/EtOAc =10:1), the title compound (23.4 mg) was obtained in 92% yield as light yellow solid (mp: 95-100 °C).

^{1}H NMR (400 MHz, Chloroform-*d*) δ8.31 – 8.21 (m, 1H), 7.93 – 7.77 (m, 2H), 7.58 – 7.46 (m, 3H), 7.40 (dd, *J* = 8.1, 7.1 Hz, 1H), 6.14 (s, 1H), 3.51 – 3.40 (m, 2H), 1.66 – 1.57 (m, 2.20H, **0.80D**), 1.30 – 1.24 (m, 2H), 0.95 – 0.85 (m, 6H). ^{13}C NMR (101 MHz, Chloroform-*d*) δ 169.5, 134.8, 133.6, 130.3, 130.1, 128.2, 126.9, 126.3, 125.4, 124.7, 124.6, 40.2, 36.0, 27.7, **27.3 (t, *J* = 19.0 Hz)**, 22.4. IR (ATR): ν = 3271, 2594, 2929, 2867, 1636, 1538, 765, 750 cm^{-1}. HRMS (ESI): m/z Calculated for $C_{17}H_{21}DNO^+$ [M+H^+]: 257.1759, found 257.1750.

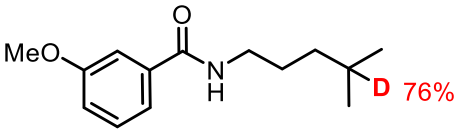

3-methoxy-N-(4-methylpentyl-4-d)benzamide (**3t**)

The title compound was prepared according to **GP**. After purified by column chromatography on silica gel (eluent: petroleum ether/EtOAc =5:1), the title compound (23.5 mg) was obtained in 99% yield as colorless oil.

^{1}H NMR (400 MHz, Chloroform-*d*) δ 7.38 – 7.32 (m, 1H), 7.29 – 7.26 (m, 2H), 7.04 – 6.94 (m, 1H), 6.42 (t, *J* = 5.9 Hz, 1H), 3.80 (s, 3H), 3.39 (td, *J* = 7.3, 5.7 Hz, 2H), 1.61 – 1.52 (m, 2.24H, **0.76D**), 1.25 – 1.19 (m, 2H), 0.89 – 0.83 (m, 6H). ^{13}C NMR (101 MHz, Chloroform-*d*) δ 167.3, 159.7, 136.3, 129.4, 118.6, 117.4, 112.2, 55.3, 40.3, 35.9, 27.7, **27.1 (t, *J* = 19.4 Hz)**, 22.3. IR (ATR): ν = 3309, 2954, 2867, 1636, 1543, 902, 751, 690 cm^{-1}. HRMS (ESI): m/z Calculated for $C_{14}H_{21}DNO_2^+$ [M+H$^+$]: 237.1708, found 237.1699.

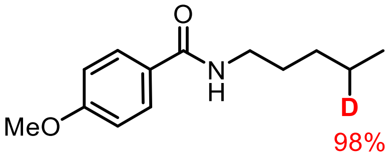

4-methoxy-N-(pentyl-4-d)benzamide (**3u**)

The title compound was prepared according to **GP**. After purified by column chromatography on silica gel (eluent: petroleum ether/EtOAc =5:1), the title compound (20.4 mg) was obtained in 92% yield as light yellow solid (mp: 68-72 °C).

3u: ^{1}H NMR (400 MHz, Chloroform-*d*) δ 7.78 – 7.68 (m, 2H), 6.94 – 6.86 (m, 2H), 6.16 (s, 1H), 3.83 (s, 3H), 3.41 (td, *J* = 7.2, 5.8 Hz, 2H), 1.64 – 1.54 (m, 2H), 1.38 – 1.24 (m, 3.02H, **0.98D**), 0.94 – 0.83 (m, 3H). ^{13}C NMR (101 MHz, Chloroform-*d*) δ 167.0, 162.0, 128.6, 127.1, 113.6, 55.3, 40.0, 29.4, 29.0 (t, *J* = 10.2 Hz), **22.0 (t, *J* = 19.1 Hz)**, 13.8 (t, *J* = 11.3 Hz). IR (ATR): ν = 3312, 2956, 2930, 1632, 1257, 765, 750 cm^{-1}. HRMS (ESI): m/z Calculated for $C_{13}H_{19}DNO_2^+$ [M+H$^+$]: 223.1551, found 223.1544.

3u': Rection conditions: **PC** (1 mol%), $NBu_4OP(O)(OBu)_2$ (5 mol%), **2a** (10 mol%), 48 h. ^{1}H NMR (400 MHz, Chloroform-*d*) δ 7.79 – 7.68 (m, 2H), 6.94 – 6.86 (m, 2H), 6.20 (s, 1H), 3.82 (s, 3H), 3.41 (td, *J* = 7.3, 5.7 Hz, 2H), 1.64 – 1.54 (m, 2H), 1.37 – 1.29 (m, 2.30H, **1.70D**), 0.94 – 0.83 (m, 3H). ^{13}C NMR (101 MHz, Chloroform-*d*) δ 167.0, 162.0, 128.6, 127.1, 113.6, 55.3, 40.0, 29.3, 28.9, **22.0 (p, *J* = 19.8 Hz)**, 13.7.

3u": Rection conditions: **PC** (2 mol%), $NBu_4OP(O)(OBu)_2$ (10 mol%), **2a** (20 mol%), 24 h. ^{1}H NMR (400 MHz, Chloroform-*d*) δ 7.76 – 7.69 (m, 2H), 6.92 – 6.86 (m, 2H), 6.19 (s, 1H), 3.82 (s, 3H), 3.41 (td, *J* = 7.3, 5.7 Hz, 2H), 1.64 – 1.54 (m, 2H), 1.37 – 1.28 (m, 2.20H, **1.80D**), 0.88 (s, 3H).

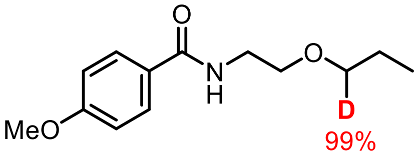

4-methoxy-N-(2-(propoxy)ethyl)benzamide (**3v**)

The title compound was prepared according to **GP**. After purified by column chromatography on silica gel (eluent: petroleum ether/EtOAc =3:1), the title compound (21.9 mg) was obtained in 92% yield as colorless oil.

^{1}H NMR (400 MHz, Chloroform-*d*) δ 7.75 – 7.71 (m, 2H), 6.92 – 6.89 (m, 2H), 6.51 (s, 1H), 3.83 (s, 3H), 3.65 – 3.60 (m, 2H), 3.59 – 3.56 (m, 2H), 3.47 – 3.35 (m, 1.01H, **0.99D**), 1.59 (p, *J* = 7.2 Hz, 2H), 0.92 (t, *J* = 7.4 Hz, 3H). ^{13}C NMR (101 MHz, Chloroform-*d*) δ 166.9, 162.0, 128.6, 126.9, 113.7, **72.4 (t, *J* = 21.2 Hz)**, 69.2 (t, *J* = 2.6 Hz), 55.3, 39.7, 22.7 (t, *J* = 11.2 Hz), 10.5. IR (ATR): ν = 3328, 2962, 2935, 1634, 1505, 1257, 765, 750 cm^{-1}. HRMS (ESI): m/z Calculated for $C_{13}H_{19}DNO_3^+$ [M+H$^+$]: 239.1500, found 239.1495.

Rection conditions: **PC** (1 mol%), $NBu_4OP(O)(OBu)_2$ (5 mol%), **2a** (10 mol%), 48 h. ^{1}H NMR (400 MHz, Chloroform-*d*) δ 7.78 – 7.70 (m, 2H), 6.94 – 6.88 (m, 2H), 6.51 (s, 1H), 3.83 (s, 3H), 3.64 – 3.60 (m, 2H), 3.60 – 3.54 (m, 2H), 3.45 – 3.36 (m, 0.70H, **1.30D**), 1.59 (p, *J* = 7.0 Hz, 2H), 0.92 (t, *J* = 7.4 Hz, 3H).

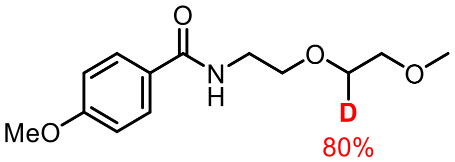

4-methoxy-N-(2-(2-methoxyethoxy-1-d)ethyl)benzamide (**3w**)

The title compound was prepared according to **GP**. After purified by column chromatography on silica gel (eluent: petroleum ether/EtOAc =1:1), the title compound (27.2 mg) was obtained in 95% yield as colorless oil.

^{1}H NMR (400 MHz, Chloroform-*d*) δ 7.80 – 7.70 (m, 2H), 6.93 – 6.86 (m, 2H), 6.69 (s, 1H), 3.83 (s, 3H), 3.68 – 3.61 (m, 5.20H, **0.80D**), 3.57 – 3.50 (m, 2H), 3.37 (s, 3H). ^{13}C NMR (101 MHz, Chloroform-*d*) δ 166.9, 162.0, 128.7, 126.8, 113.6, 72.0 – 71.5 (m), 70.1, **69.7 (t, *J* = 21.5 Hz)**, 59.0, 55.3, 39.5. IR (ATR): ν = 3315, 3006. 2878, 1637, 1276, 764, 750 cm^{-1}. HRMS (ESI): m/z Calculated for $C_{13}H_{19}DNO_4^+$ [M+H$^+$]: 255.1450, found 255.1446.

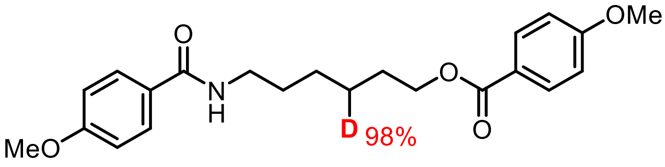

6-(4-methoxybenzamido)hexyl-3-d 4-methoxybenzoate (**3x**)

The title compound was prepared according to **GP**. After purified by column chromatography on silica gel (eluent: petroleum ether/EtOAc =3:1), the title compound (37.5 mg) was obtained in 97% yield as white solid (mp: 122-125 °C).

^{1}H NMR (400 MHz, Chloroform-*d*) δ 8.02 – 7.93 (m, 2H), 7.77 – 7.70 (m, 2H), 6.92 – 6.85 (m, 4H), 6.30 (s, 1H), 4.27 (t, *J* = 6.5 Hz, 2H), 3.82 (d, *J* = 7.7 Hz, 6H), 3.42 (q, *J* = 6.8 Hz, 2H), 1.74 (p, *J* = 6.4 Hz, 2H), 1.61 (p, *J* = 7.3 Hz, 2H), 1.51 – 1.37 (m, 3.02H, **0.98D**). ^{13}C NMR (101 MHz, Chloroform-*d*) δ 167.0, 166.4, 163.2, 161.9, 131.5, 128.6, 127.0, 122.8, 113.6, 113.5, 64.5, 55.3, 55.3, 39.8, 29.6, 28.5 (t, *J* = 9.7 Hz), 26.4 (t, *J* = 10.3 Hz), **25.3 (t, *J* = 19.3 Hz)**. IR (ATR): ν = 3327, 2934, 2857, 1709, 1632, 1256, 767, 750 cm^{-1}. HRMS (ESI): m/z Calculated for $C_{22}H_{27}DNO_5^+$ [M+H$^+$]: 387.2025, found 387.2021.

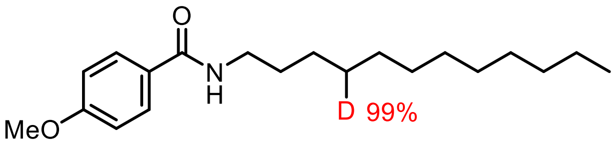

N-(dodecyl-4-d)-4-methoxybenzamide (**3y**)

The title compound was prepared according to **GP**. After purified by column chromatography on silica gel (eluent: petroleum ether/EtOAc =5:1), the title compound (31.5 mg) was obtained in 98% yield as light yellow solid (mp: 95-98 °C).

^{1}H NMR (400 MHz, Chloroform-*d*) δ 7.75 – 7.69 (m, 2H), 6.92 – 6.86 (m, 2H), 6.17 (t, *J* = 5.8 Hz, 1H), 3.83 (s, 3H), 3.41 (td, *J* = 7.2, 5.7 Hz, 2H), 1.62 – 1.54 (m, 2H), 1.39 – 1.16 (m, 17.01H, 0.99D), 0.87 (t, *J* = 6.9 Hz, 3H). ^{13}C NMR (101 MHz, Chloroform-*d*) δ 167.0, 162.0, 128.6, 127.1, 113.6, 55.3, 40.0, 31.9, 29.7, 29.6, 29.6, 29.5, 29.4, 29.3, **28.9 (t, *J* = 18.8 Hz)**, 27.1 – 26.4 (m), 22.6, 14.1. IR (ATR): ν = 3338, 2918, 2849, 1628, 1258, 765, 750 cm^{-1}. HRMS (ESI): m/z Calculated for $C_{20}H_{33}DNO_2^+$ [M+H$^+$]: 321.2647, found 321.2640.

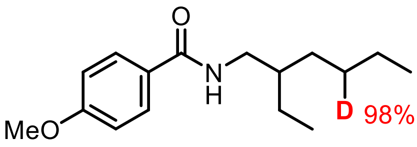

N-(2-ethylhexyl-4-d)-4-methoxybenzamide (**3z**)

The title compound was prepared according to **GP**. After purified by column chromatography on silica gel (eluent: petroleum ether/EtOAc =5:1), the title compound (25.8 mg) was obtained in 98% yield as white solid (mp: 88-91 °C).

^{1}H NMR (400 MHz, Chloroform-*d*) δ 7.76 – 7.68 (m, 2H), 6.94 – 6.87 (m, 2H), 6.11 (t, *J* = 5.9 Hz, 1H), 3.83 (s, 3H), 3.42 – 3.31 (m, 2H), 1.59 – 1.49 (m, 1H), 1.45 – 1.17 (m, 7.02H, **0.98D**), 0.99

– 0.81 (m, 6H). ^{13}C NMR (101 MHz, Chloroform-*d*) δ 167.1, 162.0, 128.5, 127.2, 113.7, 55.3, 42.9, 39.5, 31.2 – 30.7 (m), **28.5 (t, *J* = 19.0 Hz)**, 24.3, 23.2 – 22.4 (m), 14.0, 10.9. IR (ATR): ν = 3311, 2958, 2827, 2872, 1631, 1254, 766, 750 cm^{-1}. HRMS (ESI): m/z Calculated for $C_{16}H_{25}DNO_2^+$ [M+H$^+$]: 265.2021, found 265.2014.

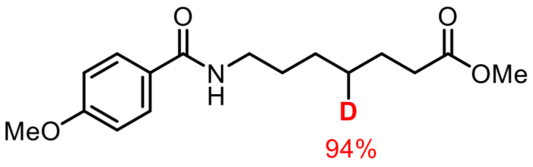

methyl 7-(4-methoxybenzamido)heptanoate-4-d (**3aa**)

The title compound was prepared according to **GP**. After purified by column chromatography on silica gel (eluent: petroleum ether/EtOAc =3:1), the title compound (28.1 mg) was obtained in 96% yield as light yellow solid (mp: 79-82 °C).

^{1}H NMR (400 MHz, Chloroform-*d*) δ 7.75 – 7.70 (m, 2H), 6.89 (dd, *J* = 8.9, 2.4 Hz, 2H), 6.25 (s, 1H), 3.82 (s, 3H), 3.64 (s, 3H), 3.43 – 3.36 (m, 2H), 2.28 (t, *J* = 7.4 Hz, 2H), 1.63 – 1.54 (m, 4H), 1.44 – 1.18 (m, 3.06H, **0.94D**). ^{13}C NMR (101 MHz, Chloroform-*d*) δ 174.1, 167.0, 162.0, 128.6, 127.0, 113.6, 55.3, 51.4, 39.8, 33.9, 29.4, **28.3 (t, *J* = 19.0 Hz)**, 26.4 (t, *J* = 10.2 Hz), 24.6 (t, *J* = 10.0 Hz). IR (ATR): ν = 3330, 2932, 2858, 1735, 1630, 1253, 766, 750 cm^{-1}. HRMS (ESI): m/z Calculated for $C_{16}H_{23}DNO_4^+$ [M+H$^+$]: 295.1763, found 295.1758.

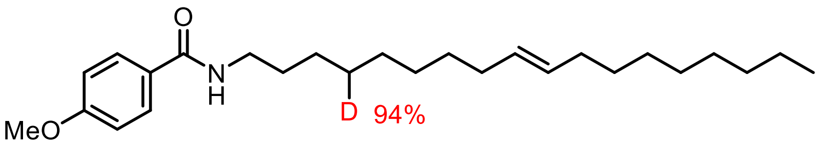

(E)-4-methoxy-N-(octadec-9-en-1-yl-4-d)benzamide (**3bb**)

The title compound was prepared according to **GP**. After purified by column chromatography on silica gel (eluent: petroleum ether/EtOAc =5:1), the title compound (38.8 mg) was obtained in 97% yield as light yellow solid (mp: 92-96 °C).

^{1}H NMR (400 MHz, Chloroform-*d*) δ 7.76 – 7.66 (m, 2H), 6.94 – 6.84 (m, 2H), 6.23 – 6.09 (m, 1H), 5.42 – 5.28 (m, 2H), 3.83 (s, 3H), 3.41 (td, *J* = 7.3, 5.7 Hz, 2H), 2.09 – 1.78 (m, 4H), 1.58 (p, *J* = 7.3 Hz, 2H), 1.35 – 1.21 (m, 21.06H, **0.94D**), 0.87 (t, *J* = 6.8 Hz, 3H). ^{13}C NMR (101 MHz, Chloroform-*d*) δ 167.0, 162.0, 130.4, 129.9, 128.6, 127.1, 113.6, 55.3, 40.0, 32.6, 31.9, 29.7, 29.7, 29.5, 29.5, 29.4, 29.3, 29.3, **29.0 (t, *J* = 18.7 Hz)**, 27.2, 26.9 (t, *J* = 10.2 Hz), 22.6, 14.1. IR (ATR): ν = 3334, 2920, 2851, 1629, 1258, 765, 750 cm^{-1}. HRMS (ESI): m/z Calculated for $C_{26}H_{43}DNO_2^+$ [M+H$^+$]: 403.3429, found 403.3422.

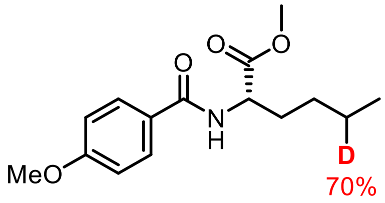

methyl (2S)-2-(4-methoxybenzamido)hexanoate-5-d (**3cc**)

The title compound was prepared according to **GP**. After purified by column chromatography on silica gel (eluent: petroleum ether/EtOAc =3:1), the title compound (27.6 mg) was obtained in 98% yield as light yellow solid (mp: 80-83 °C).

^{1}H NMR (400 MHz, Chloroform-*d*) δ 7.79 – 7.73 (m, 2H), 6.93 – 6.88 (m, 2H), 6.62 (d, *J* = 7.7 Hz, 1H), 4.79 (td, *J* = 7.5, 5.4 Hz, 1H), 3.83 (s, 3H), 3.76 (s, 3H), 1.98 – 1.89 (m, 1H), 1.80 – 1.71 (m, 1H), 1.40 – 1.26 (m, 3.30H, **0.70D**), 0.91 – 0.84 (m, 3H). ^{13}C NMR (101 MHz, Chloroform-*d*) δ 173.4, 166.4, 162.3, 128.8, 126.2, 113.7, 55.4, 52.4, 52.3, 32.4, 27.7 – 26.9 (m), **21.9 (t, *J* = 19.1 Hz)**, 14.2 – 13.2 (m). IR (ATR): ν = 3311, 3007, 2955, 2862, 1744, 1637, 1276, 1259, 764, 750 cm^{-1}. HRMS (ESI): m/z Calculated for $C_{15}H_{21}DNO_4^+$ [M+H$^+$]: 281.1606, found 281.1598.

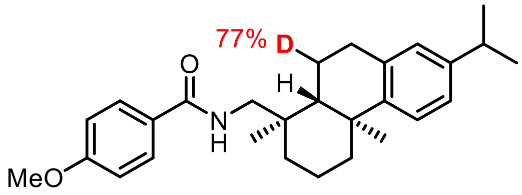

N-(((1R,4aS,10aR)-7-isopropyl-1,4a-dimethyl-1,2,3,4,4a,9,10,10a-octahydrophenanthren-1-yl-10-d)methyl)-4-methoxybenzamide (**3dd**)

The title compound was prepared according to **GP**. After purified by column chromatography on silica gel (eluent: petroleum ether/EtOAc =5:1), the title compound (41.2 mg) was obtained in 98% yield as light yellow solid (mp: 160-163 °C).

^{1}H NMR (400 MHz, Chloroform-*d*) δ 7.74 – 7.68 (m, 2H), 7.18 (d, *J* = 8.1 Hz, 1H), 7.00 (dd, *J* = 8.1, 2.0 Hz, 1H), 6.94 – 6.86 (m, 3H), 6.16 – 6.08 (m, 1H), 3.83 (s, 3H), 3.48 – 3.28 (m, 2H), 2.98 – 2.88 (m, 1H), 2.89 – 2.74 (m, 2H), 2.35 – 2.26 (m, 1H), 2.02 – 1.95 (m, 0.23H, **0.77D**), 1.88 – 1.66 (m, 3H), 1.56 – 1.46 (m, 2H), 1.44 – 1.33 (m, 2H), 1.27 – 1.23 (m, 6H), 1.22 (s, 3H), 1.01 (s, 3H). ^{13}C NMR (101 MHz, Chloroform-*d*) δ 167.1, 162.0, 147.0, 145.5, 134.7, 128.6, 127.1, 126.9, 124.2, 123.8, 113.7, 55.3, 50.2, 45.9 – 45.5 (m), 38.3, 37.6, 37.5, 36.4, 33.4, 30.5 – 30.1 (m), 25.4, 23.9, 19.0, 18.8, 18.6. IR (ATR): ν = 3319, 2958, 2927, 2866, 1635, 1276, 765, 750 cm^{-1}. HRMS (ESI): m/z Calculated for $C_{28}H_{37}DNO_2^+$ [M+H$^+$]: 421.2960, found 421.2950.

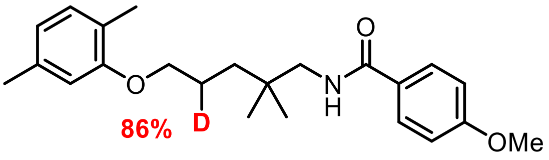

N-(5-(2,5-dimethylphenoxy)-2,2-dimethylpentyl-4-d)-4-methoxybenzamide (**3ee**)

The title compound was prepared according to **GP**. After purified by column chromatography on silica gel (eluent: petroleum ether/EtOAc =5:1), the title compound (33.6 mg) was obtained in 91% yield as white solid (mp: 132-136 °C).

^{1}H NMR (400 MHz, Chloroform-*d*) δ 7.77 – 7.69 (m, 2H), 7.00 (d, *J* = 7.4 Hz, 1H), 6.96 – 6.87 (m, 2H), 6.69 – 6.65 (m, 1H), 6.63 (d, *J* = 1.6 Hz, 1H), 6.19 – 6.11 (m, 1H), 3.94 (t, *J* = 6.1 Hz, 2H), 3.84 (s, 3H), 3.33 (d, *J* = 6.3 Hz, 2H), 2.31 (s, 3H), 2.16 – 2.12 (m, 2.14H, **0.86D**), 1.86 – 1.78 (m, 2H), 1.52 – 1.43 (m, 2H), 1.00 (s, 6H). ^{13}C NMR (101 MHz, Chloroform-*d*) δ 167.1, 162.0, 156.9, 136.4, 130.2, 128.5, 127.2, 123.5, 120.7, 113.7, 112.1, 68.4, 55.4, 49.2, 36.1, 34.4, 25.1, 24.2, 21.4, 15.7, **15.5 (t, *J* = 19.5 Hz)**. HRMS (ESI): m/z Calculated for $C_{23}H_{31}DNO_3^+$ [M+H$^+$]: 371.2440, found 371.2428.

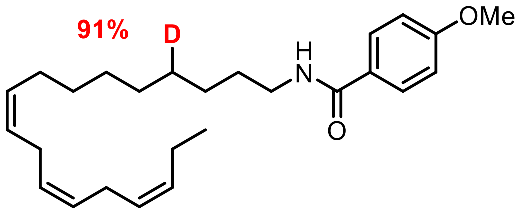

4-methoxy-N-((9Z,12Z,15Z)-octadeca-9,12,15-trien-1-yl-4-d)benzamide (**3ff**)

The title compound was prepared according to **GP**. After purified by column chromatography on silica gel (eluent: petroleum ether/EtOAc =5:1), the title compound (33 mg) was obtained in 83% yield as colorless oil.

^{1}H NMR (400 MHz, Chloroform-*d*) δ 7.74 – 7.70 (m, 2H), 6.92 – 6.88 (m, 2H), 6.14 (t, *J* = 5.8 Hz, 1H), 5.57 – 5.14 (m, 6H), 3.83 (s, 3H), 3.44 – 3.38 (m, 2H), 2.88 – 2.63 (m, 4H), 2.12 – 1.92 (m, 4H), 1.65 – 1.52 (m, 2H), 1.36 – 1.26 (m, 9.09H, **0.91D**), 0.97 (t, *J* = 7.5 Hz, 3H). ^{13}C NMR (101 MHz, Chloroform-*d*) δ 167.0, 162.0, 131.9, 130.3, 128.6, 128.2, 127.6, 127.1, 127.1, 113.6, 55.3, 40.0, 29.7, 29.6, 29.4, 29.3, 29.2, **28.9 (t, *J* = 19.0 Hz)**, 27.2, 27.0, 25.6, 25.5, 20.5, 14.2. HRMS (ESI): m/z Calculated for $C_{26}H_{39}DNO_2^+$ [M+H$^+$]: 399.3087, found 399.3093.

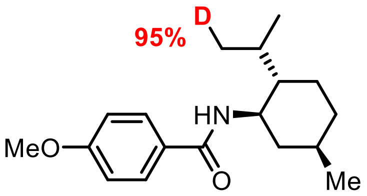

4-methoxy-N-((1R,2S,5R)-5-methyl-2-((R)-propan-2-yl-1-d)cyclohexyl)benzamide (**3gg**)

The title compound was prepared according to **GP**. After purified by column chromatography on silica gel (eluent: petroleum ether/EtOAc =5:1), the title compound (27 mg) was obtained in 93% yield as white solid (mp: 110-113 °C).

^{1}H NMR (400 MHz, Chloroform-*d*) δ 7.71 (d, J = 8.8 Hz, 2H), 6.95 – 6.89 (m, 2H), 6.17 – 6.03 (m, 1H), 4.58 – 4.48 (m, 1H), 3.84 (s, 3H), 2.03 – 1.95 (m, 1H), 1.95 – 1.87 (m, 1H), 1.82 – 1.74 (m, 1H), 1.56 – 1.44 (m, 1H), 1.43 – 1.33 (m, 1H), 1.18 – 0.95 (m, 4H), 0.95 – 0.85 (m, 8.05H, **0.95D**). ^{13}C NMR (101 MHz, Chloroform-*d*) δ 166.1, 162.0, 128.5, 127.6, 113.7, 55.4, 46.4, 46.3, 40.1, 34.7, 30.0 – 29.4 (m), 27.1, 25.7, 22.2, 21.0, 20.8, **20.5 (t, J = 18.5 Hz)**. HRMS (ESI): m/z Calculated for $C_{18}H_{27}DNO_2^+$ [M+H$^+$]: 291.2177, found 291.2168.

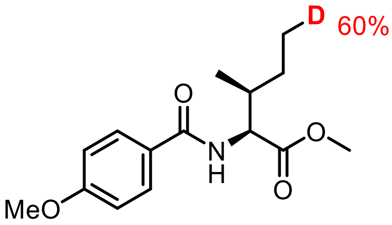

methyl (4-methoxybenzoyl)-L-isoleucinate-5-d (**3hh**)

The title compound was prepared according to **GP**. After purified by column chromatography on silica gel (eluent: petroleum ether/EtOAc =3:1), the title compound (23.2 mg) was obtained in 83% yield as light yellow solid (mp: 76-79 °C).

^{1}H NMR (400 MHz, Chloroform-*d*) δ 7.80 – 7.74 (m, 2H), 6.95 – 6.89 (m, 2H), 6.58 (d, J = 8.5 Hz, 1H), 4.80 (dd, J = 8.5, 5.0 Hz, 1H), 3.84 (s, 3H), 3.76 (s, 3H), 2.06 – 1.93 (m, 1H), 1.58 – 1.46 (m, 1H), 1.31 – 1.19 (m, 1H), 0.98 – 0.91 (m, 5.40H, **0.60D**). ^{13}C NMR (101 MHz, Chloroform-*d*) δ 172.8, 166.6, 162.4, 128.9, 126.4, 113.8, 56.7, 55.4, 52.1, 38.3, 25.4, 15.5, 11.6, **11.2 (t, J = 19.3 Hz)**. IR (ATR): ν = 3319, 2963, 2877, 1740, 1637, 1255, 766, 750 cm^{-1}. HRMS (ESI): m/z Calculated for $C_{15}H_{21}DNO_4^+$ [M+H$^+$]: 281.1606, found 281.1599.

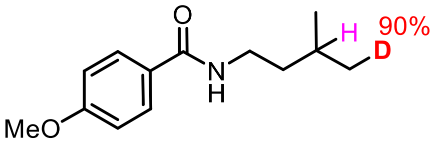

4-methoxy-N-(3-methylbutyl-4-d)benzamide (**3ii**)

The title compound was prepared according to **GP**. After purified by column chromatography on silica gel (eluent: petroleum ether/EtOAc =5:1), the title compound (20.8 mg) was obtained in 94% yield as light yellow solid (mp: 65-68 °C).

^{1}H NMR (400 MHz, Chloroform-*d*) δ 7.77 – 7.66 (m, 2H), 6.94 – 6.86 (m, 2H), 6.11 (s, 1H), 3.83 (s, 3H), 3.50 – 3.39 (m, 2H), 1.72 – 1.59 (m, 1H), 1.53 – 1.45 (m, 2H), 0.94 (d, J = 6.6 Hz, 5.10H, **0.90D**). ^{13}C NMR (101 MHz, Chloroform-*d*) δ 167.0, 162.0, 128.6, 127.1, 113.6, 55.3, 38.6, 38.3, 25.9,

22.5, **22.2 (t, *J* = 19.0 Hz)**. IR (ATR): ν = 3312, 2954, 2929, 1631, 1505, 1254, 1180, 766, 750 cm^{-1}. HRMS (ESI): m/z Calculated for $C_{13}H_{19}DNO_2^+$ [M+H$^+$]: 223.1551, found 223.1545.

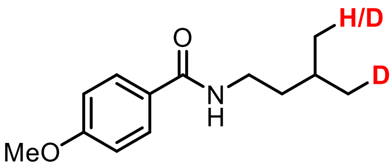

4-methoxy-N-(3-methylbutyl-4-d)benzamide (**3ii'**)

Rection conditions: **PC** (1 mol%), $NBu_4OP(O)(OBu)_2$ (5 mol%), **2a** (10 mol%), 48 h. ^{1}H NMR (400 MHz, Chloroform-*d*) δ 7.76 – 7.68 (m, 2H), 6.93 – 6.86 (m, 2H), 6.12 (s, 1H), 3.83 (s, 3H), 3.48 – 3.40 (m, 2H), 1.72 – 1.60 (m, 1H), 1.53 – 1.45 (m, 2H), 0.97 – 0.88 (m, 4.90H, **1.10D**). ^{13}C NMR (126 MHz, Chloroform-*d*) δ 167.0, 162.0, 128.6, 127.1, 113.6, 55.3, 38.5, 38.3, 26.1 – 25.4 (m), 22.5, 22.4, **22.2 (t, *J* = 19.2 Hz, 3ii'-*d*$_1$), 22.1 (t, *J* = 19.2 Hz, 3ii'-*d*$_2$)**. HRMS (ESI): m/z Calculated for $C_{13}H_{19}DNO_2^+$ [M+H$^+$]: 223.1551, found 223.1545. HRMS (ESI): m/z Calculated for $C_{13}H_{18}D_2NO_2^+$ [M+H$^+$]: 224.1614, found 224.1608.

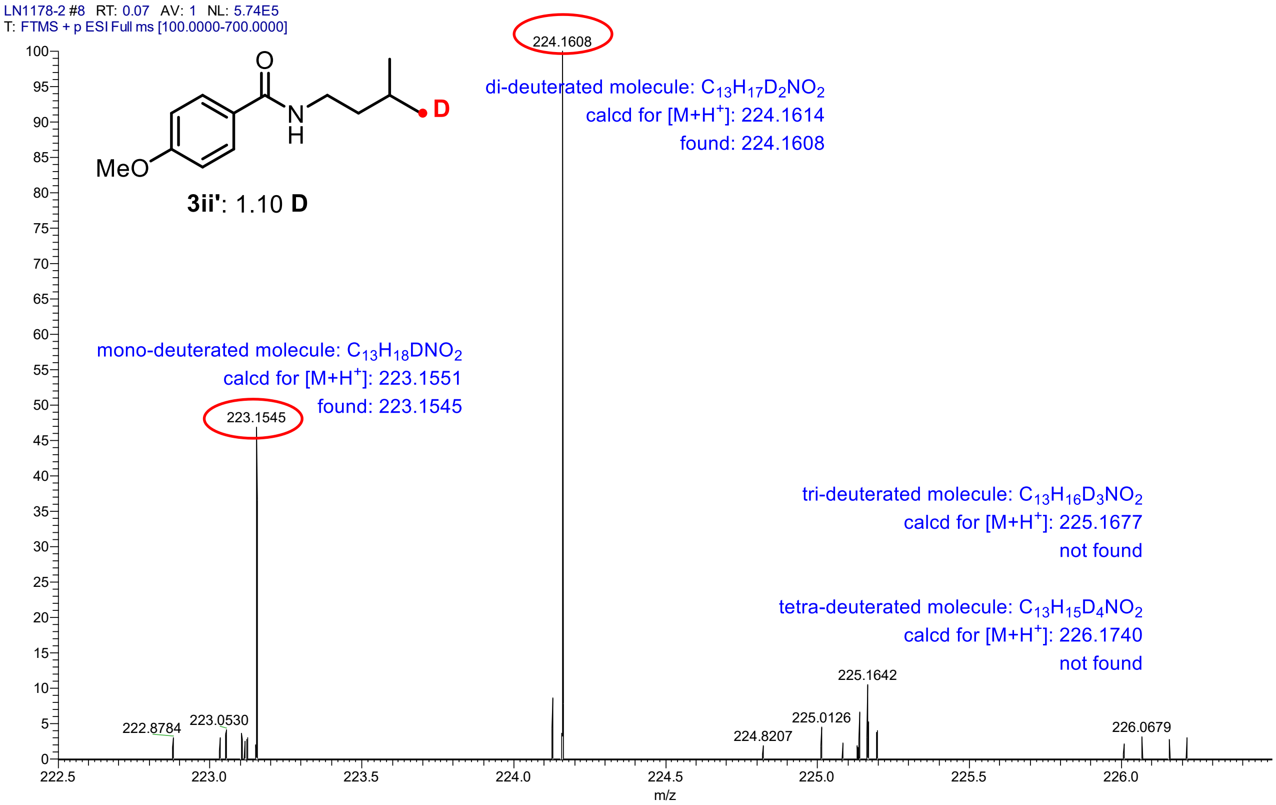

Supplementary Figure 2. HRMS spectrum of **3ii'**.

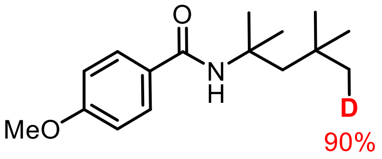

4-methoxy-N-(2,4,4-trimethylpentan-2-yl-5-d)benzamide (**3jj**)

The title compound was prepared according to **GP**. After purified by column chromatography on silica gel (eluent: petroleum ether/EtOAc =5:1), the title compound (24.0 mg) was obtained in 91% yield as light yellow solid (mp: 90-92 °C).

^{1}H NMR (400 MHz, Chloroform-*d*) δ 7.70 – 7.60 (m, 2H), 6.93 – 6.85 (m, 2H), 5.88 (s, 1H), 3.82 (s, 3H), 1.85 (s, 2H), 1.51 (s, 6H), 1.03 (s, 8..10H, **0.90D**). ^{13}C NMR (101 MHz, Chloroform-*d*) δ 166.1, 161.8, 128.5, 128.3, 113.6, 55.3, 51.8, 31.5, **31.1 (t, *J* = 19.1 Hz)**, 29.3. IR (ATR): ν = 3315, 2953, 2838, 1635, 1255, 766, 750 cm^{-1}. HRMS (ESI): m/z Calculated for $C_{16}H_{25}DNO_2^+$ [M+H$^+$]: 265.2021, found 265.2014.

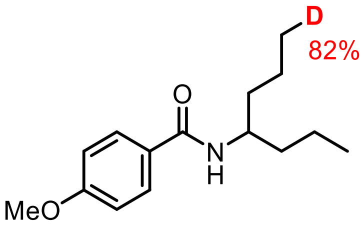

N-(heptan-4-yl-1-d)-4-methoxybenzamide (**3kk**)

The title compound was prepared according to **GP**. After purified by column chromatography on silica gel (eluent: petroleum ether/EtOAc =5:1), the title compound (23.7 mg) was obtained in 94% yield as light yellow solid (mp: 135-139 °C).

^{1}H NMR (400 MHz, Chloroform-*d*) δ 7.76 – 7.69 (m, 2H), 6.95 – 6.89 (m, 2H), 5.70 (d, *J* = 9.1 Hz, 1H), 4.21 – 4.10 (m, 1H), 3.84 (s, 3H), 1.61 – 1.51 (m, 2H), 1.49 – 1.31 (m, 6H), 0.95 – 0.88 (m, 5.18H, **0.82D**). ^{13}C NMR (101 MHz, Chloroform-*d*) δ 166.5, 162.0, 128.5, 127.4, 113.7, 55.4, 49.2, 37.7, 19.2, 14.1, **13.7 (t, *J* = 19.3 Hz)**. IR (ATR): ν = 3301, 2955, 1627, 1276, 1258, 764, 750 cm^{-1}. HRMS (ESI): m/z Calculated for $C_{15}H_{23}DNO_2^+$ [M+H$^+$]: 251.1864, found 251.1856.

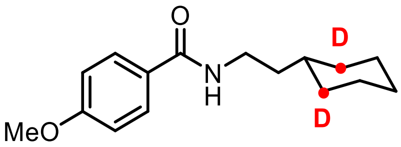

N-(2-(cyclohexyl-2,6-d$_2$)ethyl)-4-methoxybenzamide (**4a**)

The title compound was prepared according to **GP**. After purified by column chromatography on silica gel (eluent: petroleum ether/EtOAc =5:1), the title compound (24.8 mg) was obtained in 95% yield as light yellow solid (mp: 86-89 °C).

^{1}H NMR (400 MHz, Chloroform-*d*) δ 7.75 – 7.69 (m, 2H), 6.92 – 6.86 (m, 2H), 6.13 (s, 1H), 3.83 (s, 3H), 3.43 (dt, *J* = 7.5, 5.9 Hz, 2H), 1.75 – 1.61 (m, 4.02H, **0.98D**), 1.48 (q, *J* = 7.1 Hz, 2H), 1.35 – 1.28 (m, 1H), 1.24 – 1.08 (m, 3H), 0.99 – 0.84 (m, 1.02H, **0.98D**).^{13}C NMR (101 MHz, Chloroform-

d) δ 166.9, 161.9, 128.6, 127.1, 113.6, 55.3, 37.8, 37.1, 35.3 (p, *J* = 9.2 Hz), **32.7 (t, *J* = 19.4 Hz)**, 26.4, 26.1 (t, *J* = 10.7 Hz). IR (ATR): ν = 3312, 2920, 2851, 1631, 1504, 1256, 765, 750 cm^{-1}. HRMS (ESI): m/z Calculated for $C_{16}H_{22}D_2NO_2^+$ [M+H$^+$]: 264.1927, found 264.1920.

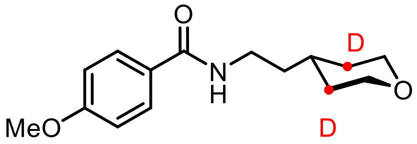

4-methoxy-N-(2-((3R,5R)-tetrahydro-2H-pyran-4-yl-3,5-d$_2$*)ethyl)benzamide* (**4b**)

The title compound was prepared according to **GP**. After purified by column chromatography on silica gel (eluent: petroleum ether/EtOAc =3:1), the title compound (24.4 mg) was obtained in 92% yield as light yellow solid (mp: 88-90 °C).

^{1}H NMR (400 MHz, Chloroform-*d*) δ 7.75 – 7.68 (m, 2H), 6.89 – 6.83 (m, 2H), 6.46 (d, *J* = 6.0 Hz, 1H), 3.89 (d, *J* = 10.9 Hz, 2H), 3.80 (s, 3H), 3.41 (q, *J* = 7.0 Hz, 2H), 3.37 – 3.25 (m, 2H), 1.61 – 1.49 (m, 4.40H, **0.60D**), 1.32 – 1.20 (m, 1.40H, **0.60D**). ^{13}C NMR (101 MHz, Chloroform-*d*) δ 167.0, 161.9, 128.6, 126.8, 113.5, 67.8, 55.3, 37.2, 36.7, 32.8, **32.7 – 32.0 (m)**. IR (ATR): ν = 3318, 2927, 2840, 1632, 1505, 1255, 765, 750 cm^{-1}. HRMS (ESI): m/z Calculated for $C_{15}H_{20}D_2NO_3^+$ [M+H$^+$]: 266.1720, found 266.1714.

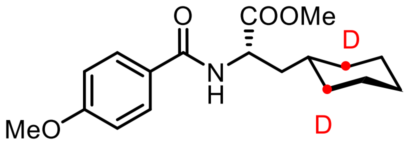

methyl (2S)-3-(cyclohexyl-2,6-d$_2$*)-2-(4-methoxybenzamido)propanoate* (**4c**)

The title compound was prepared according to **GP**. After purified by column chromatography on silica gel (eluent: petroleum ether/EtOAc =3:1), the title compound (27.5 mg) was obtained in 86% yield as light yellow solid (mp: 102-105 °C).

^{1}H NMR (400 MHz, Chloroform-*d*) δ 7.84 – 7.68 (m, 2H), 6.96 – 6.88 (m, 2H), 6.48 (d, *J* = 8.2 Hz, 1H), 4.85 (td, *J* = 8.6, 5.3 Hz, 1H), 3.84 (s, 3H), 3.75 (s, 3H), 1.88 – 1.60 (m, 6.40H, **0.60D**), 1.42 – 1.33 (m, 1H), 1.25 – 1.10 (m, 3H), 1.03 – 0.85 (m, 1.40H, **0.60D**). ^{13}C NMR (101 MHz, Chloroform-*d*) δ 174.0, 166.5, 162.3, 128.9, 126.2, 113.7, 55.4, 52.3, 50.5, 40.3, 34.4 – 33.8 (m), 33.0 (t, *J* = 19.0 Hz), **32.2 (t, *J* = 18.1 Hz)**, 26.7 – 25.3 (m). IR (ATR): ν = 3326, 2922, 2851, 1744, 1634, 1256, 765, 750 cm^{-1}. HRMS (ESI): m/z Calculated for $C_{18}H_{25}DNO_4^+$ [M+H$^+$]: 321.1919, found 321.1919. HRMS (ESI): m/z Calculated for $C_{18}H_{24}D_2NO_4^+$ [M+H$^+$]: 322.1982, found 322.1977.

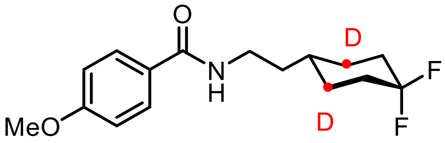

N-(2-(4,4-difluorocyclohexyl-2,6-d$_2$)ethyl)-4-methoxybenzamide (**4d**)

The title compound was prepared according to **GP**. After purified by column chromatography on silica gel (eluent: petroleum ether/EtOAc =5:1), the title compound (26.5 mg) was obtained in 89% yield as light yellow solid (mp: 94-95 °C).

^{1}H NMR (400 MHz, Chloroform-*d*) δ 7.77 – 7.68 (m, 2H), 6.94 – 6.86 (m, 2H), 6.20 (s, 1H), 3.83 (s, 3H), 3.45 (dt, *J* = 7.6, 5.8 Hz, 2H), 2.10 – 2.00 (m, 2H), 1.84 – 1.77 (m, 1.30H, **0.70D**), 1.75 – 1.59 (m, 2H), 1.58 – 1.51 (m, 2H), 1.41 (s, 1H), 1.35 – 1.25 (m, 1.30H, **0.70D**). ^{13}C NMR (101 MHz, Chloroform-*d*) δ 167.0, 162.1, 128.6, 126.8, 113.7, 55.4, 37.9, 35.7, 33.8 – 32.6 (m), 28.8 (d, *J* = 9.4 Hz), 28.6 – 28.0 (m). ^{19}F NMR (376 MHz, Chloroform-*d*) δ -91.6 (d, *J* = 235.1 Hz), -101.9 (d, *J* = 235.0 Hz). IR (ATR): ν = 3306, 2936, 2862, 1631, 1505, 1256, 765, 750 cm^{-1}. HRMS (ESI): m/z Calculated for $C_{16}H_{21}DF_2NO_2^+$ [M+H$^+$]: 299.1676, found 299.1672. HRMS (ESI): m/z Calculated for $C_{16}H_{20}D_2F_2NO_2^+$ [M+H$^+$]: 300.1739, found 300.173.

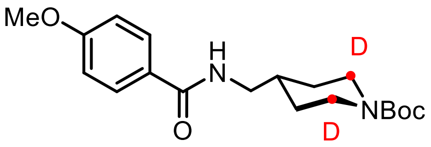

tert-butyl 4-((4-methoxybenzamido)methyl)piperidine-1-carboxylate-2,6-d2 (**4e**)

The title compound was prepared according to **GP**. After purified by column chromatography on silica gel (eluent: DCM/MeOH =20:1), the title compound (33.7 mg) was obtained in 97% yield as light yellow oil.

^{1}H NMR (400 MHz, Chloroform-*d*) δ 7.76 – 7.69 (m, 2H), 6.93 – 6.86 (m, 2H), 6.36 (s, 1H), 4.14 – 4.03 (m, 1.44H, **0.56D**), 3.83 (s, 3H), 3.31 (s, 2H), 2.66 (t, *J* = 12.8 Hz, 1.08H, **0.92D**), 1.83 – 1.72 (m, 1H), 1.69 (d, *J* = 14.1 Hz, 2H), 1.43 (s, 9H), 1.21 – 1.09 (m, 2H). ^{13}C NMR (101 MHz, Chloroform-*d*) δ 167.1, 162.1, 154.8, 128.6, 126.8, 113.7, 79.3, 55.4, 45.3, 43.4, 36.4, 30.3 – 29.3 (m), 28.4. IR (ATR): ν = 3317, 3005, 2976, 2929, 1689, 1257, 765, 750 cm^{-1}. HRMS (ESI): m/z Calculated for $C_{19}H_{28}DN_2O_4^+$ [M+H$^+$]: 350.2185, found 350.2184. HRMS (ESI): m/z Calculated for $C_{19}H_{27}D_2N_2O_4^+$ [M+H$^+$]: 351.2247, found 351.2245.

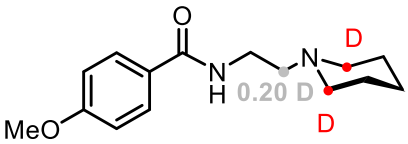

4-methoxy-N-(2-(piperidin-1-yl-2,6-d$_2$)ethyl-2-d)benzamide (**4f**)

The title compound was prepared according to **GP**. After purified by column chromatography on silica gel (eluent: DCM/MeOH =20:1), the title compound (23.5 mg) was obtained in 89% yield as light yellow oil.

^{1}H NMR (400 MHz, Chloroform-*d*) δ 7.80 – 7.71 (m, 2H), 6.99 (s, 1H), 6.94 – 6.88 (m, 2H), 3.83 (s, 3H), 3.56 – 3.46 (m, 2H), 2.60 – 2.49 (m, 1.80H, **0.20D**), 2.44 (s, 2.50H, **1.50D**), 1.66 – 1.54 (m, 4H), 1.52 – 1.40 (m, 2H). ^{13}C NMR (101 MHz, Chloroform-*d*) δ 166.8, 162.0, 128.7, 127.0, 113.6, 57.1, 55.3, **53.8 (t, *J* = 20.3 Hz)**, 36.2, 25.9, 24.2. IR (ATR): ν = 3303, 2934, 2854, 1635, 1258, 764, 750 cm^{-1}. HRMS (ESI): m/z Calculated for $C_{15}H_{22}DN_2O_2^+$ [M+H$^+$]: 264.1817, found 264.1812. HRMS (ESI): m/z Calculated for $C_{15}H_{21}D_2N_2O_2^+$ [M+H$^+$]: 265.1880, found 265.1872. HRMS (ESI): m/z Calculated for $C_{15}H_{20}D_3N_2O_2^+$ [M+H$^+$]: 266.1932, found 266.1942.

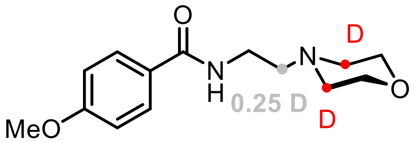

4-methoxy-N-(2-(morpholino-3,5-d₂)ethyl-2-d)benzamide (**4g**)

The title compound was prepared according to **GP**. After purified by column chromatography on silica gel (eluent: DCM/MeOH =10:1), the title compound (22.5 mg) was obtained in 98% yield as light yellow oil.

^{1}H NMR (400 MHz, Chloroform-*d*) δ 7.76 – 7.71 (m, 2H), 6.95 – 6.89 (m, 2H), 6.72 (s, 1H), 3.84 (s, 3H), 3.76 – 3.65 (m, 4H), 3.55 – 3.49 (m, 2H), 2.63 – 2.52 (m, 1.75H, **0.25D**), 2.51 – 2.43 (m, 2.10H, **1.90D**). ^{13}C NMR (101 MHz, Chloroform-*d*) δ 166.9, 162.0, 128.6, 126.9, 113.7, 66.9 (t, *J* = 6.5 Hz), 56.9, 55.3, **52.9 (t, *J* = 20.1 Hz)**, 36.0. IR (ATR): ν = 3325, 2965, 2853, 1633, 1504, 1254, 766, 750 cm^{-1}. HRMS (ESI): m/z Calculated for $C_{14}H_{19}D_2N_2O_3^+$ [M+H$^+$]: 267.1672, found 267.1664.

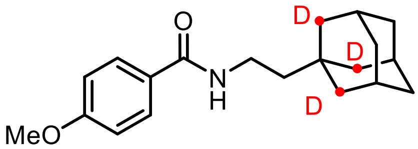

N-(2-((1S,3R,5S,7S,8S)-adamantan-1-yl-2,8-d₂)ethyl)-4-methoxybenzamide (**4h**)

The title compound was prepared according to **GP**. After purified by column chromatography on silica gel (eluent: petroleum ether/EtOAc =5:1), the title compound (29.0 mg) was obtained in 92% yield as light yellow oil.

^{1}H NMR (400 MHz, Chloroform-*d*) δ 7.76 – 7.65 (m, 2H), 6.94 – 6.85 (m, 2H), 6.05 (t, *J* = 5.5 Hz, 1H), 3.82 (s, 3H), 3.51 – 3.36 (m, 2H), 2.00 – 1.89 (m, 3H), 1.74 – 1.67 (m, 3H), 1.66 – 1.58 (m, 3H), 1.55 – 1.49 (m, 4.16H, **1.84D**), 1.40 – 1.33 (m, 2H). ^{13}C NMR (101 MHz, Chloroform-*d*) δ 166.9, 161.9, 128.5, 127.2, 113.6, 55.3, 43.9, **41.9 (t, *J* = 19.0 Hz)**, 37.0, 35.1, 32.1 – 31.3 (m), 28.7 – 28.2

(m). IR (ATR): ν = 3309, 2963, 1634, 1504, 1254, 766, 750 cm^{-1}. HRMS (ESI): m/z Calculated for $C_{20}H_{27}DNO_2^+$ [M+H$^+$]: 315.2177, found 315.2172. HRMS (ESI): m/z Calculated for $C_{20}H_{26}D_2NO_2^+$ [M+H$^+$]: 316.2240, found 316.2238.

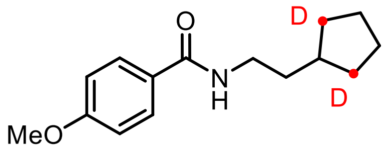

N-(2-(cyclopentyl-2,2,5-d_4)ethyl)-4-methoxybenzamide (**4i**)

The title compound was prepared according to **GP**. After purified by column chromatography on silica gel (eluent: petroleum ether/EtOAc =5:1), the title compound (23.9 mg) was obtained in 96% yield as light yellow solid (mp: 89-92 °C).

^{1}H NMR (400 MHz, Chloroform-*d*) δ 7.78 – 7.67 (m, 2H), 6.94 – 6.87 (m, 2H), 6.15 (s, 1H), 3.83 (s, 3H), 3.47 – 3.39 (m, 2H), 1.85 – 1.74 (m, 2.00H, **1.00D**), 1.65 – 1.54 (m, 4H), 1.54 – 1.46 (m, 2H), 1.17 – 1.05 (m, 0.50H, **1.50D**). ^{13}C NMR (101 MHz, Chloroform-*d*) δ 166.9, 162.0, 128.6, 127.1, 113.6, 55.3, 39.5, 37.6 (t, *J* = 9.9 Hz), 35.9, **32.9 – 31.0 (m)**, 25.1-24.9 (m). IR (ATR): ν = 3312, 2937, 2870, 1630, 1504, 1254, 766, 750 cm^{-1}. HRMS (ESI): m/z Calculated for $C_{15}H_{20}D_2NO_2^+$ [M+H$^+$]: 250.1771, found 250.1767. HRMS (ESI): m/z Calculated for $C_{15}H_{19}D_3NO_2^+$ [M+H$^+$]: 251.1833, found 251.1824.

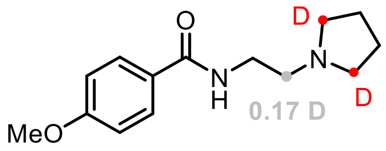

4-methoxy-N-(2-(pyrrolidin-1-yl-2,5-d_2)ethyl-2-d)benzamide (**4j**)

The title compound was prepared according to **GP**. After purified by column chromatography on silica gel (eluent: DCM/MeOH =20:1), the title compound (23.5 mg) was obtained in 94% yield as light yellow solid (mp: 86-89 °C).

^{1}H NMR (400 MHz, Chloroform-*d*) δ 7.78 – 7.72 (m, 2H), 6.92 – 6.87 (m, 2H), 6.83 (s, 1H), 3.83 (s, 3H), 3.57 – 3.48 (m, 2H), 2.75 – 2.61 (m, 1.83H, **0.17D**), 2.59 – 2.49 (m, 2.50H, **1.50D**), 1.82 – 1.72 (m, 4H). ^{13}C NMR (101 MHz, Chloroform-*d*) δ 167.0, 162.0, 128.7, 127.0, 113.6, 55.3, 54.6, 53.9, **53.5 (t, *J* = 20.5 Hz)**, 38.4, 23.4 (t, *J* = 11.4 Hz). IR (ATR): ν = 3330, 2962, 2800, 1634, 1276, 1257, 765, 750 cm^{-1}. HRMS (ESI): m/z Calculated for $C_{14}H_{20}DN_2O_2^+$ [M+H$^+$]: 250.1660, found 250.1657. HRMS (ESI): m/z Calculated for $C_{14}H_{19}D_2N_2O_2^+$ [M+H$^+$]: 251.1723, found 251.1719.

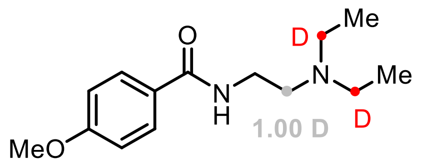

N-(2-(bis(ethyl-1-d)amino)ethyl-2-d)-4-methoxybenzamide (**4k**)

The title compound was prepared according to **GP**. After purified by column chromatography on silica gel (eluent: DCM/MeOH =20:1), the title compound (23.0 mg) was obtained in 92% yield as light yellow oil.

^{1}H NMR (400 MHz, Chloroform-*d*) δ 7.81 (d, *J* = 8.4 Hz, 2H), 7.27 (s, 1H), 6.97 – 6.87 (m, 2H), 3.84 (s, 3H), 3.54 (d, *J* = 2.3 Hz, 2H), 2.79 – 2.71 (m, 1.00H, **1.00D**), 2.70 – 2.61 (m, 1.00H, **3.00D**), 1.15 – 1.05 (m, 6H). ^{13}C NMR (101 MHz, Chloroform-*d*) δ 166.9, 162.0, 128.8, 126.7, 113.7, 55.3, 52.0 – 50.3 (m), **46.7 (t, *J* = 20.0 Hz)**, 36.8 (t, *J* = 9.4 Hz), 11.0. IR (ATR): ν = 3312, 2966, 2934, 1635, 1258, 764, 750 cm^{-1}. HRMS (ESI): m/z Calculated for $C_{14}H_{20}D_3N_2O_2^+$ [M+H$^+$]: 254.1942, found 254.1938.

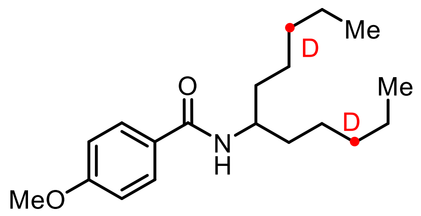

4-methoxy-N-(undecan-6-yl-3,9-d$_2$)benzamide (**4l**)

The title compound was prepared according to **GP**. After purified by column chromatography on silica gel (eluent: petroleum ether/EtOAc =5:1), the title compound (29.7 mg) was obtained in 97% yield as light yellow solid (mp: 142-145 °C).

^{1}H NMR (400 MHz, Chloroform-*d*) δ 7.77 – 7.69 (m, 2H), 6.95 – 6.87 (m, 2H), 5.78 (d, *J* = 9.0 Hz, 1H), 4.18 – 4.02 (m, 1H), 3.83 (s, 3H), 1.64 – 1.51 (m, 2H), 1.48 – 1.22 (m, 11.80H, **2.2D**), 0.86 (t, *J* = 7.1 Hz, 6H). ^{13}C NMR (101 MHz, Chloroform-*d*) δ 166.5, 161.9, 128.5, 127.4, 113.6, 55.3, 49.7, 35.3 (t, *J* = 2.7 Hz), **31.4 (t, *J* = 18.9 Hz)**, 25.5 (t, *J* = 10.5 Hz), 22.4 (t, *J* = 10.3 Hz), 14.0 (t, *J* = 2.7 Hz). IR (ATR): ν = 3273, 2956, 2927, 2855, 1626, 1254, 765, 750 cm^{-1}. HRMS (ESI): m/z Calculated for $C_{19}H_{30}D_2NO_2^+$ [M+H$^+$]: 308.2553, found 308.2544.

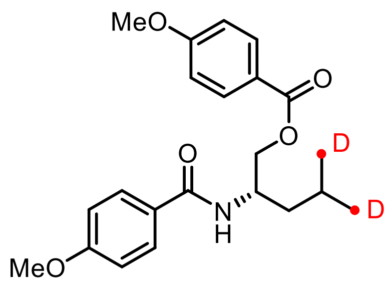

(S)-2-(4-methoxybenzamido)-4-(methyl-d)pentyl-5-d 4-methoxybenzoate (**4m**)

The title compound was prepared according to **GP**. After purified by column chromatography on silica gel (eluent: petroleum ether/EtOAc =3:1), the title compound (33.3 mg) was obtained in 86% yield as white solid (mp: 120-122 °C).

^{1}H NMR (400 MHz, Chloroform-*d*) δ 8.03 – 7.91 (m, 2H), 7.77 – 7.67 (m, 2H), 6.94 – 6.82 (m, 4H), 6.29 (d, *J* = 8.7 Hz, 1H), 4.66 – 4.54 (m, 1H), 4.47 – 4.31 (m, 2H), 3.82 (d, *J* = 8.8 Hz, 6H), 1.81 – 1.67 (m, 1H), 1.63 – 1.43 (m, 2H), 1.01 – 0.92 (m, 4.50H, **1.50D**). ^{13}C NMR (101 MHz, Chloroform-*d*) δ 166.62, 166.53, 163.46, 162.06, 131.65, 128.64, 126.83, 122.17, 113.66, 113.64, 66.64, 55.37, 55.32, 47.35, 40.93, 25.10 – 24.15 (m), **22.79 (t, *J* = 19.2 Hz), 22.09 (t, *J* = 19.1 Hz)**. IR (ATR): ν = 3311, 2955, 2840, 1712, 1631, 1256, 766, 750 cm^{-1}. HRMS (ESI): m/z Calculated for $C_{22}H_{27}DNO_5^+$ [M+H$^+$]: 387.2025, found 387.2019. (ESI): m/z Calculated for $C_{22}H_{26}D_2NO_5^+$ [M+H$^+$]: 388.2088, found 388.2078.

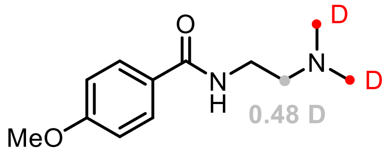

N-(2-(bis(methyl-d)amino)ethyl-2-d)-4-methoxybenzamide (**4n**)

The title compound was prepared according to **GP**. After purified by column chromatography on silica gel (eluent: DCM/MeOH =20:1), the title compound (20.5 mg) was obtained in 92% yield as light yellow oil.

^{1}H NMR (400 MHz, Chloroform-*d*) δ 7.81 – 7.72 (m, 2H), 6.95 – 6.87 (m, 2H), 6.75 (s, 1H), 3.83 (s, 3H), 3.55 – 3.44 (m, 2H), 2.53 – 2.44 (m, 1.52H, **0.48D**), 2.28 – 2.19 (m, 3.00H, **3.00D**). ^{13}C NMR (101 MHz, Chloroform-*d*) δ 166.9, 162.0, 128.7, 127.0, 113.6, 57.7, 55.3, **45.4 – 43.5 (m)**, 37.2 – 36.8 (m). IR (ATR): ν = 3316, 2943, 2838, 1634, 1256, 765, 750 cm^{-1}. HRMS (ESI): m/z Calculated for $C_{12}H_{18}DN_2O_2^+$ [M+H$^+$]: 224.150, found 224.1499. HRMS (ESI): m/z Calculated for $C_{12}H_{17}D_2N_2O_2^+$ [M+H$^+$]: 225.1567, found 225.1560. HRMS (ESI): m/z Calculated for $C_{12}H_{16}D_3N_2O_2^+$ [M+H$^+$]: 226.1629, found 226.1621. HRMS (ESI): m/z Calculated for $C_{12}H_{15}D_4N_2O_2^+$ [M+H$^+$]: 227.1692, found 227.1682.

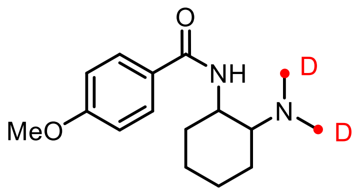

N-(2-(bis(methyl-d)amino)cyclohexyl)-4-methoxybenzamide (**4o**)

The title compound was prepared according to **GP**. After purified by column chromatography on silica gel (eluent: DCM/MeOH =20:1), the title compound (25.1 mg) was obtained in 91% yield as light yellow solid (mp: 107-110 °C).

^{1}H NMR (400 MHz, Chloroform-*d*) δ 7.78 – 7.71 (m, 2H), 6.97 – 6.92 (m, 1H), 6.92 – 6.86 (m, 2H), 3.83 (s, 3H), 3.67 – 3.52 (m, 1H), 2.71 – 2.64 (m, 1H), 2.48 – 2.35 (m, 1H), 2.25 – 2.18 (m, 2.60H, **3.40D**), 1.97 – 1.76 (m, 2H), 1.75 – 1.65 (m, 1H), 1.43 – 1.31 (m, 1H), 1.30 – 1.20 (m, 2H), 1.17 – 1.07 (m, 1H). ^{13}C NMR (101 MHz, Chloroform-*d*) δ 167.4, 161.9, 128.7, 127.6, 113.5, 66.4, 55.3, 51.7, **40.1 – 37.5 (m)**, 32.6, 25.4, 24.6, 21.2. IR (ATR): ν = 3332, 3006, 2931, 2857, 1629, 1276, 765, 750 cm^{-1}. HRMS (ESI): m/z Calculated for $C_{16}H_{24}DO_2^+$ [M+H$^+$]: 278.1973, found 278.1972. HRMS (ESI): m/z Calculated for $C_{16}H_{23}D_2N_2O_2^+$ [M+H$^+$]: 279.2036, found 279.2032. HRMS (ESI): m/z Calculated for $C_{16}H_{22}D_3O_2^+$ [M+H$^+$]: 280.2099, found 280.2090.

4. Synthetic Application

5.0 mmol scale synthesis of Compound 3u

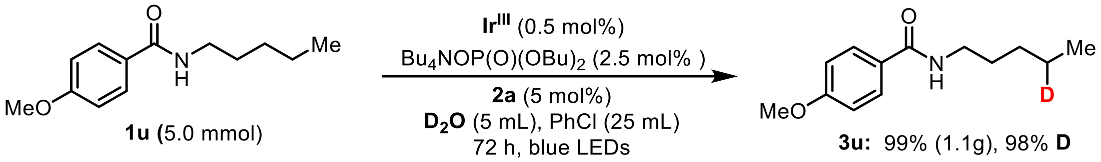

An 100 mL tube was charged with Ir[dF(CF$_3$)ppy)]$_2$(5,5'-dCF$_3$bpy)PF$_6$ (27.5 mg, 0.5 mol%), tetrabutylammonium dibutyl phosphate (57.5 mg, 2.5 mol%), thiol catalyst **2a** (70 mg, 5 mol%), **1u** (1.105 g, 5.0 mmol, 1.0 equiv), then the tube was delivered to glove box, PhCl (25 mL) and D_2O (5 mL) were added. The tube was stirred under the irradiation of two blue LEDs at room temperature for 72 h. After the reaction was finished, the reaction mixture was extracted by ethyl acetate, dried by anhydrous Na_2SO_4, filtered and collected the organic layer. The organic solvent was removed under the reduced pressure. The residue was purified by column chromatography on silica gel (Eluent: petroleum ether/ethyl acetate = 5/1) to afford the product **3u** (1.1 g, 98% yield) as a white to grey solid.

Synthesis of Compound 5

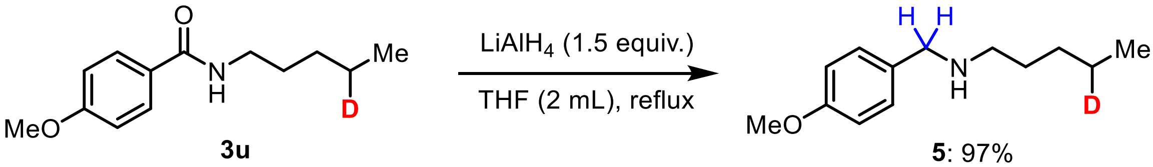

***N-(4-methoxybenzyl)pentan-4-d-1-amine* (5)**. Under air atmosphere, in an oven-dried tube, to a mixture of lithium aluminum hydride (1M in THF, 0.3 mL, 0.3 mmol, 1.5 equiv.) in anhydrous THF (1 mL), **3u** (44.4 mg, 0.2 mmol, 1.0 equiv.) was added slowly at 0 ˚C. After stirring at 0 ˚C for 1 h, the reaction mixture was heated up slowly and was allowed to reflux overnight. At 0 ˚C, the reaction

mixture was quenched slowly with the addition of 15% aqueous NaOH solution. The mixture was extracted with ethyl acetate three times. The combined organic layers were dried over Na_2SO_4 and filtered. The filtered solution was concentrated and the crude product was purified by flash chromatography (Eluent: petroleum ether/ethyl acetate = 5/1) to afford the product **7** (40.3 mg, 97% yield) as a colorless oil. ^{1}H NMR (400 MHz, Chloroform-*d*) δ 7.26 – 7.22 (m, 2H), 6.92 – 6.80 (m, 2H), 3.79 (s, 3H), 3.73 (s, 2H), 2.61 (t, *J* = 7.3 Hz, 2H), 1.56 – 1.47 (m, 2H), 1.31 – 1.24 (m, 3.02H, **0.98D**), 0.91 – 0.85 (m, 3H).. ^{13}C NMR (101 MHz, Chloroform-*d*) δ 158.6, 132.0, 129.4, 113.7, 55.2, 53.2, 49.2, 29.4, 29.3, **22.1 (t, *J* = 19.0 Hz)**, 13.9 (t, *J* = 11.4 Hz). HRMS (ESI): m/z Calculated for $C_{13}H_{21}DNO^+$ [M+H$^+$]: 209.1759, found 209.1759.

Synthesis of Compound 6

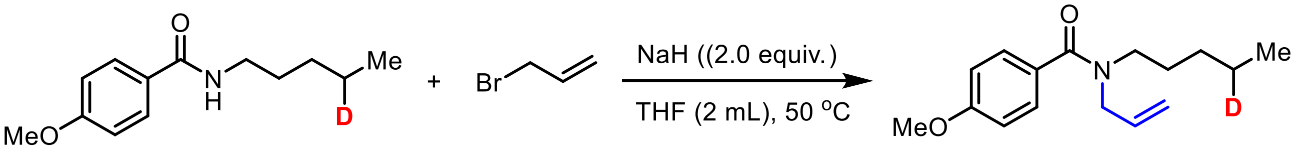

3u 2.0 equiv. **6**: 93%

***N*-allyl-4-methoxy-*N*-(pentyl-4-*d*)benzamide** (**6**). The title compound was synthesized according to the reported literature.[1] Under Ar atmosphere, **3u** (44.4 mg, 0.2 mmol, 1.0 equiv.) dissolved in 1.0 mL THF was added to a suspension of sodium hydride (60% dispersion in mineral oil, 16 mg, 2.0 equiv.) in 1.0 mL THF at 0°C and stirred for 30 min at 50°C. Then allyl bromide (35 uL, 2.0 equiv.) was added at 0°C, and the mixture was stirred overnight at 50 °C. The reaction mixture was quenched with water in an ice bath and then was extracted with ethyl acetate three times. The combined organic layers were dried over Na_2SO_4 and filtered. The filtered solution was concentrated and the crude product was purified by flash chromatography (Eluent: petroleum ether/ethyl acetate = 5/1) to afford the product **8** (48.9 mg, 93% yield) as a colorless oil. ^{1}H NMR (400 MHz, Chloroform-*d*) δ 7.38 – 7.31 (m, 2H), 6.91 – 6.83 (m, 2H), 5.78 (s, 1H), 5.25 – 5.14 (m, 2H), 4.22 – 3.83 (m, 2H), 3.79 (s, 3H), 3.59 – 3.10 (m, 2H), 1.57 (s, 2H), 1.38 – 1.03 (m, 3.02H, **0.98D**), 0.93 – 0.75 (m, 3H). ^{13}C NMR (101 MHz, Chloroform-*d*) δ 171.6, 160.4, 133.7, 129.0, 128.4, 117.1, 113.5, 55.3, 51.7, 45.2, 28.9, 27.0, **21.9 (t, *J* = 18.7 Hz)**, 13.8 (t, *J* = 11.3 Hz). HRMS (ESI): m/z Calculated for $C_{16}H_{23}DNO_2^+$ [M+H$^+$]: 263.1864, found 263.1857.

Synthesis of Compound 7

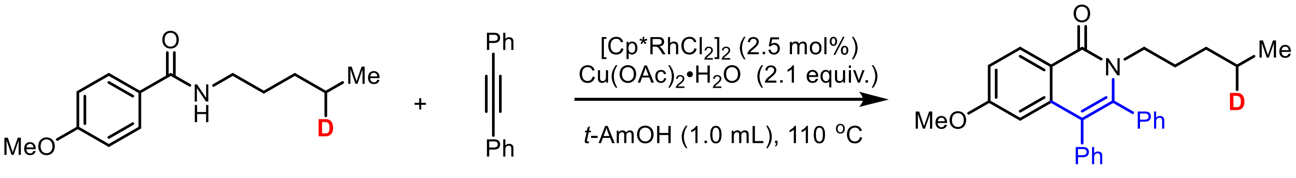

3u 1.25 equiv. **7**: 89%

6-methoxy-2-(pentyl-4-d)-3,4-diphenylisoquinolin-1(2H)-one (**7**). The title compound was synthesized according to the reported literature.[2] To a 10 mL vial equipped with a stirring bar was added **3u** (22.2 mg, 0.1 mmol, 1.0 equiv.), diphenylacetylene (22.3 mg, 0.125 mmol, 1.25 equiv.), $[Cp^*RhCl_2]_2$ (1.6 mg, 2.5 mol %) and $Cu(OAc)_2$•H_2O (42 mg, 2.1 equiv.), the tube was evacuated and backfilled with Ar (three times), *t*-amyl alcohol (1.0 ml) was added by syringe under Ar. The tube was then sealed and was placed into a 110 °C heating block for 16 h. The reaction was quenched by 10% NH_4OH and extracted with ethyl acetate three times. The combined organic layers were dried over Na_2SO_4 and filtered. The filtered solution was concentrated and the crude product was purified by flash chromatography (Eluent: petroleum ether/ethyl acetate = 10/1) to afford the product **6** (35.5 mg, 89% yield) as a white solid (mp: 187-193 °C). ^{1}H NMR (400 MHz, Chloroform-*d*) δ 8.48 (d, *J* = 8.9 Hz, 1H), 7.22 – 7.09 (m, 8H), 7.09 – 7.03 (m, 3H), 6.48 (d, *J* = 2.5 Hz, 1H), 3.87 – 3.76 (m, 2H), 3.68 (s, 3H), 1.64 – 1.55 (m, 2H), 1.16 – 1.02 (m, 3.02H, **0.98D**), 0.79 – 0.73 (m, 3H). ^{13}C NMR (101 MHz, Chloroform-*d*) δ 162.5, 161.9, 141.8, 139.2, 136.7, 134.9, 131.5, 130.2, 130.0, 128.1, 127.9, 127.8, 126.7, 119.2, 118.7, 115.4, 106.9, 55.2, 46.1, 28.8 (t, *J* = 10.3 Hz), 28.4, **21.6 (t, *J* = 19.1 Hz)**, 13.7 (t, *J* = 11.2 Hz). HRMS (ESI): m/z Calculated for $C_{27}H_{27}DNO_2^+$ [M+H$^+$]: 399.2177, found 399.2172.

Synthesis of Compound 8

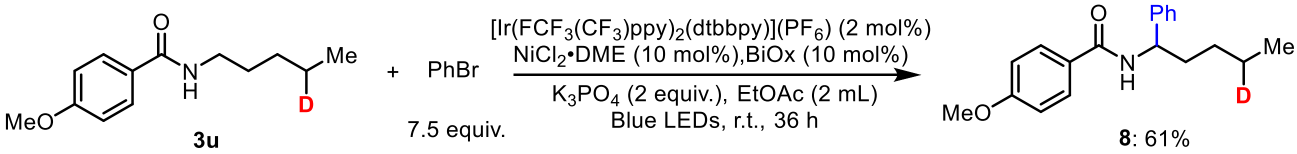

4-methoxy-N-(1-phenylpentyl-4-d)benzamide (**8**). The title compound was synthesized according to the reported literature[3] by slightly modifying the reaction conditions. To a 10 mL vial equipped with a stirring bar was added **3u** (22.2 mg, 0.1 mmol, 1.0 equiv.), the photocatalyst (2.2 mg, 2 mol%), the ligand (1.4 mg, 10 mol%) and the base (42.4 mg, 2.0 equiv). The vial was then introduced in the nitrogen-filled glovebox where the nickel catalyst (2.2 mg, 10 mol%), bromobenzene (84 µL, 7.5 equiv.) and EtOAc (2 mL) were added. The reaction was stirred and irradiated under two blue LED lamps for 36 h. The reaction was quenched by saturated NaCl and extracted with ethyl acetate three times. The combined organic layers were dried over Na_2SO_4 and filtered. The filtered solution was concentrated and the crude product was purified by flash chromatography (Eluent: petroleum ether/ethyl acetate = 5/1) to afford the product **5** (18.2 mg, 61% yield) as a white solid (mp: 147-155 °C). ^{1}H NMR (400 MHz, Chloroform-*d*) δ 7.77 – 7.71 (m, 2H), 7.38 – 7.31 (m, 4H), 7.30 – 7.26 (m, 1H), 6.95 – 6.87 (m, 2H), 6.28 (d, *J* = 8.1 Hz, 1H), 5.20 – 5.10 (m, 1H), 3.83 (s, 3H), 1.96 – 1.82 (m, 2H), 1.43 – 1.26 (m, 3.02H, **0.98D**), 0.90 – 0.82 (m, 3H). ^{13}C NMR (101 MHz, Chloroform-*d*) δ 166.1, 162.1, 142.6, 128.7, 128.6, 127.3, 126.9, 126.6, 113.7, 55.4, 53.8, 36.0, 28.3 (t, *J* = 10.2 Hz),

22.1 (t, *J* = 19.1 Hz), 13.8 (t, *J* = 11.3 Hz). HRMS (ESI): m/z Calculated for $C_{19}H_{23}DNO_2^+$ [M+H$^+$]: 299.1864, found 299.1857.

5. DFT calculation.

All the calculations were carried out with the Gaussian 16 program package[4]. Geometry optimizations and frequency calculations were performed at the M06-2X[5] functional and 6-31G(d,p) basis set. Computed SOMO energy were illustrated by CYLView software[6].

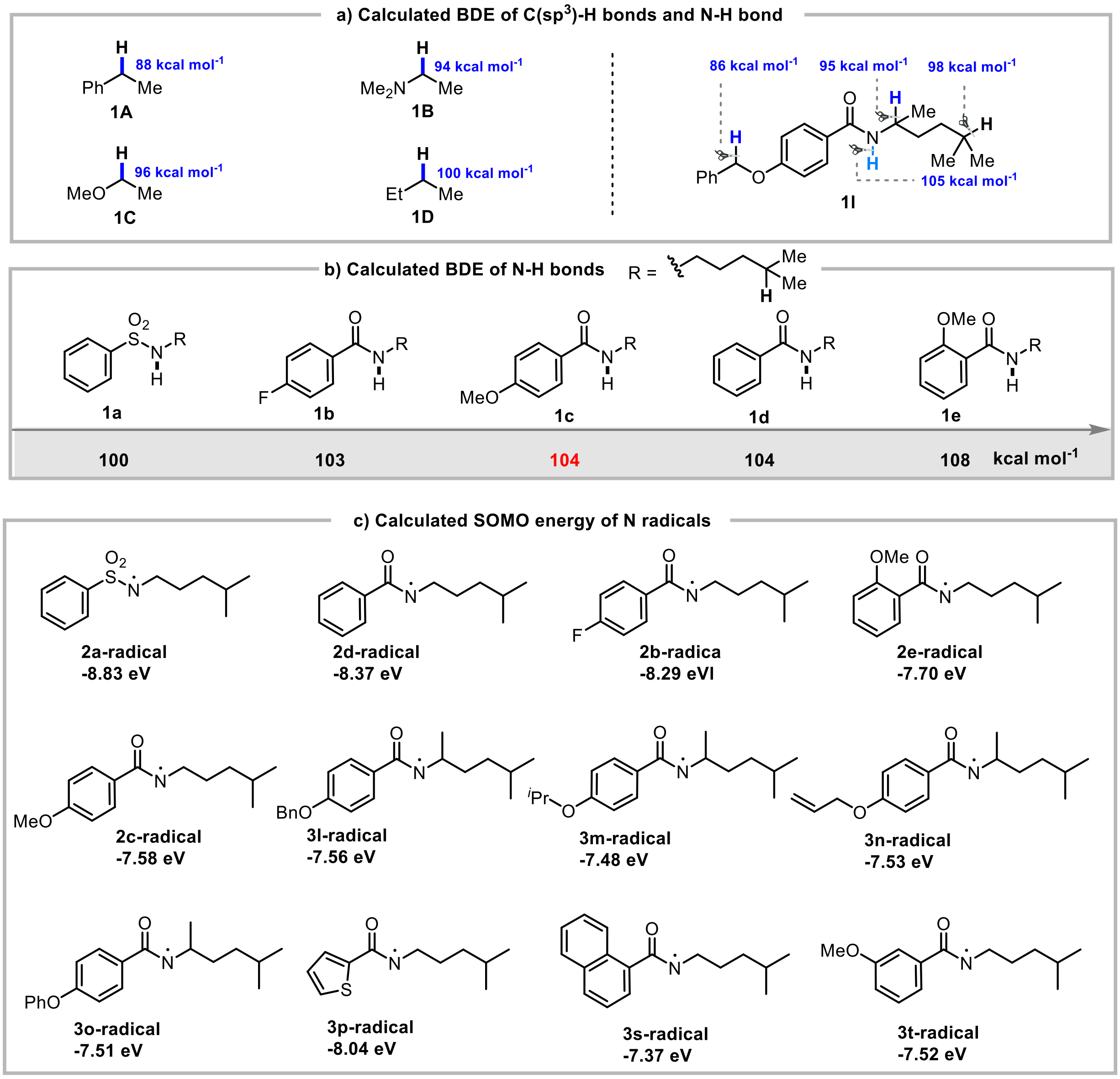

Supplementary Figure 3. DFT calculation results.

6. Prepration of amide substrates[7].

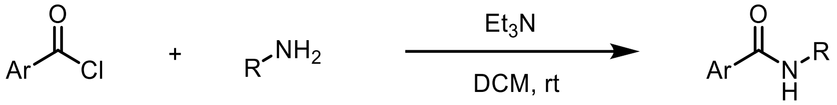

A 100 mL round-bottom flask equipped with a stirring bar was charged with aroyl chloride (5.0 mmol, 1.0 equiv), DCM (20 mL), Et_3N (1.45 mL, 10.5 mmol, 2.1 equiv) and amine/amine hydrochloride (5 mmol, 1.0 equiv). The mixture was stirred for 3 hour at room temperature. The reaction mixture was diluted with DCM and then washed with brine (20 mL), dried with anhydrous Na_2SO_4, concentrated to afford the crude product. The crude product was purified by silica gel column chromatography to get the desire product.

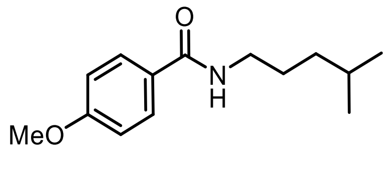

4-methoxy-N-(4-methylpentyl)benzamide (**1c**)

The title compound was synthesized according to the reported literature[7] and the characterization data is consistent with the reported data. ^{1}H NMR (400 MHz, Chloroform-*d*) δ 7.76 – 7.70 (m, 2H), 6.92 – 6.86 (m, 2H), 6.24 (s, 1H), 3.82 (s, 3H), 3.39 (td, J = 7.3, 5.8 Hz, 2H), 1.67 – 1.46 (m, 3H), 1.28 – 1.17 (m, 2H), 0.87 (d, J = 6.6 Hz, 6H). ^{13}C NMR (101 MHz, Chloroform-*d*) δ 167.0, 161.9, 128.6, 127.1, 113.6, 55.3, 40.3, 36.1, 27.8, 27.6, 22.5.

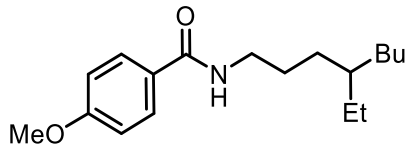

N-(4-ethyloctyl)-4-methoxybenzamide (**1f**)

The title compound was synthesized according to the reported literature[7] and the characterization data is consistent with the reported data. ^{1}H NMR (400 MHz, Chloroform-*d*) δ 7.76 – 7.69 (m, 2H), 6.94 – 6.89 (m, 2H), 6.06 (s, 1H), 3.85 (s, 3H), 3.42 (td, J = 7.3, 5.7 Hz, 2H), 1.62 – 1.53 (m, 2H), 1.32 – 1.24 (m, 6H), 1.24 – 1.17 (m, 5H), 0.87 (t, J = 6.8 Hz, 3H), 0.82 (t, J = 7.1 Hz, 3H). ^{13}C NMR (101 MHz, Chloroform-*d*) δ 167.0, 162.0, 128.6, 127.2, 113.7, 55.4, 40.5, 38.6, 32.8, 30.4, 28.9, 27.0, 25.8, 23.1, 14.2, 10.8.

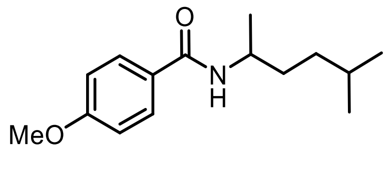

4-methoxy-N-(5-methylhexan-2-yl)benzamide (**1g**)

The title compound was synthesized according to the reported literature[8] and the characterization data is consistent with the reported data. ^{1}H NMR (400 MHz, Chloroform-*d*) δ 7.80 – 7.66 (m, 2H), 6.97 – 6.87 (m, 2H), 5.77 (d, *J* = 8.3 Hz, 1H), 4.20 – 4.10 (m, 1H), 3.85 (s, 3H), 1.58 – 1.47 (m, 3H), 1.28 – 1.21 (m, 5H), 0.88 (d, *J* = 6.6 Hz, 6H). ^{13}C NMR (101 MHz, Chloroform-*d*) δ 166.3, 162.0, 128.5, 127.3, 113.7, 55.4, 45.9, 35.1, 34.9, 28.0, 22.6, 21.1.

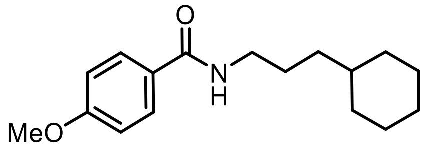

N-(3-(cyclohexyl)propyl)-4-methoxybenzamide (**1h**)

The title compound was synthesized according to the reported literature[7] and the characterization data is consistent with the reported data. ^{1}H NMR (400 MHz, Chloroform-*d*) δ 7.75 – 7.69 (m, 2H), 6.95 – 6.89 (m, 2H), 6.01 (s, 1H), 3.85 (s, 3H), 3.41 (td, *J* = 7.3, 5.7 Hz, 2H), 1.74 – 1.61 (m, 7H), 1.27 – 1.07 (m, 6H), 0.93 – 0.83 (m, 2H). ^{13}C NMR (101 MHz, Chloroform-*d*) δ 128.6, 113.7, 55.4, 40.3, 37.4, 34.7, 33.3, 27.1, 26.6, 26.3.

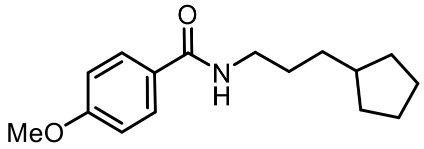

N-(3-(cyclopentyl)propyl)-4-methoxybenzamide (**1i**)

The title compound was synthesized according to the reported literature[7] and the characterization data is consistent with the reported data. ^{1}H NMR (400 MHz, Chloroform-*d*) δ 7.77 – 7.70 (m, 2H), 6.90 – 6.83 (m, 2H), 6.48 (t, *J* = 5.7 Hz, 1H), 3.80 (s, 3H), 3.37 (td, *J* = 7.2, 5.7 Hz, 2H), 1.79 – 1.66 (m, 3H), 1.63 – 1.51 (m, 4H), 1.51 – 1.42 (m, 2H), 1.37 – 1.28 (m, 2H), 1.09 – 0.97 (m, 2H). ^{13}C NMR (101 MHz, Chloroform-*d*) δ 166.95, 161.99, 128.56, 127.13, 113.68, 55.38, 40.24, 39.85, 33.40, 32.67, 28.98, 25.14.

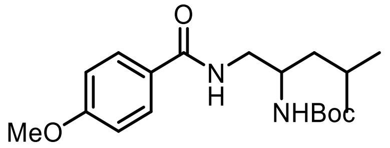

tert-butyl (1-(4-methoxybenzamido)-4-methylpentan-2-yl)carbamate (**1j**)

The title compound was synthesized according to the reported literature[9] and the characterization data is consistent with the reported data. ^{1}H NMR (400 MHz, Chloroform-*d*) δ 7.78 (d, *J* = 8.8 Hz, 2H), 7.15 (s, 1H), 6.96 – 6.87 (m, 2H), 4.55 (d, *J* = 8.5 Hz, 1H), 3.94 – 3.86 (m, 1H), 3.84 (s, 3H), 3.50

– 3.38 (m, 2H), 1.78 – 1.69 (m, 1H), 1.39 (s, 9H), 1.38 – 1.32 (m, 2H), 0.94 (dd, J = 6.6, 5.2 Hz, 6H). ^{13}C NMR (101 MHz, Chloroform-d) δ 167.2, 128.8, 113.6, 55.4, 48.7, 42.0, 28.3, 24.9, 22.9, 22.1.

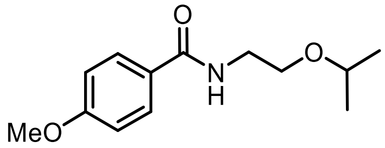

4-methoxy-N-(2-((propan-2-yl)oxy)ethyl)benzamide (**1k**)

The title compound was synthesized according to the reported literature[7] and the characterization data is consistent with the reported data. ^{1}H NMR (400 MHz, Chloroform-d) δ 7.78 – 7.71 (m, 2H), 6.96 – 6.88 (m, 2H), 6.49 (s, 1H), 3.84 (s, 3H), 3.67 – 3.55 (m, 5H), 1.17 (d, J = 6.1 Hz, 6H). ^{13}C NMR (101 MHz, Chloroform-d) δ 166.9, 162.1, 128.7, 126.9, 113.7, 71.9, 66.7, 55.4, 40.0, 22.1.

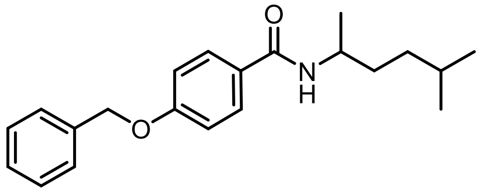

4-(benzyloxy)-N-(5-methylhexan-2-yl)benzamide (**1l**)

The title compound was synthesized according to the reported literature[10] and the characterization data is consistent with the reported data. ^{1}H NMR (400 MHz, Chloroform-d) δ 7.75 – 7.68 (m, 2H), 7.45 – 7.36 (m, 4H), 7.36 – 7.30 (m, 1H), 7.03 – 6.95 (m, 2H), 5.77 (d, J = 8.4 Hz, 1H), 5.11 (s, 2H), 4.22 – 4.06 (m, 1H), 1.57 – 1.48 (m, 3H), 1.26 – 1.20 (m, 5H), 0.88 (d, J = 6.6 Hz, 6H). ^{13}C NMR (101 MHz, Chloroform-d) δ 128.6, 128.5, 128.1, 127.4, 114.6, 70.1, 45.9, 35.1, 34.9, 28.0, 22.6, 21.1.

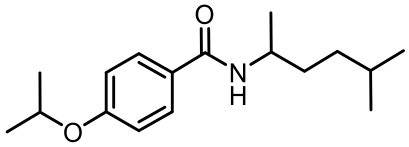

4-isopropoxy-N-(5-methylhexan-2-yl)benzamide (**1m**)

The title compound was synthesized according to the reported literature[8] and the characterization data is consistent with the reported data. ^{1}H NMR (400 MHz, Chloroform-d) δ 7.75 – 7.61 (m, 2H), 6.95 – 6.83 (m, 2H), 5.82 (d, J = 8.4 Hz, 1H), 4.66 – 4.53 (m, 1H), 4.20 – 4.06 (m, 1H), 1.61 – 1.44 (m, 3H), 1.34 (d, J = 6.1 Hz, 6H), 1.27 – 1.22 (m, 2H), 1.21 (d, J = 6.5 Hz, 3H), 0.88 (d, J = 6.6 Hz, 6H). ^{13}C NMR (101 MHz, Chloroform-d) δ 166.3, 160.4, 128.5, 126.9, 115.2, 70.0, 45.8, 35.1, 34.9, 28.0, 22.6, 21.9, 21.1.

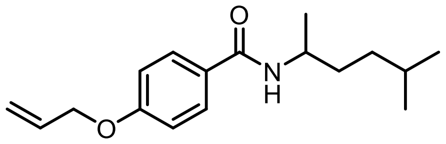

4-(allyloxy)-N-(5-methylhexan-2-yl)benzamide (**1n**)

The title compound was synthesized according to the reported literature[8] and the characterization data is consistent with the reported data. ^{1}H NMR (400 MHz, Chloroform-*d*) δ 7.71 (d, *J* = 8.8 Hz, 2H), 6.91 (d, *J* = 8.8 Hz, 2H), 6.10 – 5.93 (m, 1H), 5.89 (d, *J* = 6.9 Hz, 1H), 5.46 – 5.36 (m, 1H), 5.33 – 5.23 (m, 1H), 4.56 (d, *J* = 5.3 Hz, 2H), 4.21 – 4.06 (m, 1H), 1.55 – 1.46 (m, 2H), 1.27 – 1.18 (m, 5H), 0.86 (s, 6H). ^{13}C NMR (101 MHz, Chloroform-*d*) δ 166.2, 161.0, 132.7, 128.5, 127.5, 118.0, 114.4, 68.8, 45.9, 35.1, 34.9, 28.0, 22.6, 21.1.

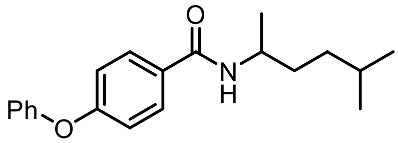

N-(5-methylhexan-2-yl)-4-phenoxybenzamide (**1o**)

The title compound was synthesized according to the reported literature[8] and the characterization data is consistent with the reported data. ^{1}H NMR (400 MHz, Chloroform-*d*) δ 7.76 – 7.69 (m, 2H), 7.41 – 7.33 (m, 2H), 7.20 – 7.12 (m, 1H), 7.08 – 7.02 (m, 2H), 7.02 – 6.96 (m, 2H), 5.80 (d, *J* = 8.2 Hz, 1H), 4.25 – 4.02 (m, 1H), 1.59 – 1.47 (m, 3H), 1.30 – 1.18 (m, 5H), 0.89 (d, *J* = 6.6 Hz, 6H). ^{13}C NMR (101 MHz, Chloroform-*d*) δ 166.1, 160.2, 156.1, 129.9, 129.5, 128.7, 124.1, 119.6, 117.9, 46.0, 35.2, 34.9, 28.0, 22.6, 21.1.

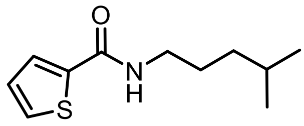

N-(4-methylpentyl)thiophene-2-carboxamide (**1p**)

The title compound was synthesized according to the reported literature[7] and the characterization data is consistent with the reported data. ^{1}H NMR (400 MHz, Chloroform-*d*) δ 7.48 (dd, *J* = 3.7, 1.2 Hz, 1H), 7.45 (dd, *J* = 4.9, 1.2 Hz, 1H), 7.07 (dd, *J* = 5.0, 3.7 Hz, 1H), 5.96 (s, 1H), 3.41 (td, *J* = 7.3, 5.8 Hz, 2H), 1.67 – 1.58 (m, 3H), 1.29 – 1.24 (m, 2H), 0.90 (d, *J* = 6.6 Hz, 6H). ^{13}C NMR (101 MHz, Chloroform-*d*) δ 129.6, 127.8, 127.5, 40.3, 36.1, 27.8, 27.6, 22.5.

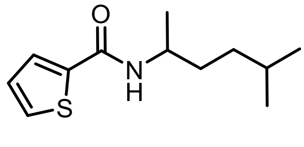

N-(5-methylhexan-2-yl)thiophene-2-carboxamide (**1q**)

The title compound was synthesized according to the reported literature[8] and the characterization data is consistent with the reported data. ^{1}H NMR (400 MHz, Chloroform-*d*) δ 7.47 (dd, *J* = 3.7, 1.2 Hz, 1H), 7.45 (dd, *J* = 5.0, 1.2 Hz, 1H), 7.06 (dd, *J* = 5.0, 3.7 Hz, 1H), 5.73 (d, *J* = 8.4 Hz, 1H), 4.22 – 4.02 (m, 1H), 1.60 – 1.48 (m, 3H), 1.29 – 1.20 (m, 5H), 0.88 (d, *J* = 6.6 Hz, 6H). ^{13}C NMR (101 MHz, Chloroform-*d*) δ 161.1, 129.5, 127.6, 127.5, 46.1, 35.1, 34.8, 27.9, 22.5, 21.0.

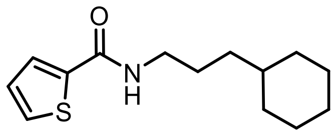

N-(3-(cyclohexyl)propyl)thiophene-2-carboxamide (**1r**)

The title compound was synthesized according to the reported literature[7] and the characterization data is consistent with the reported data. ^{1}H NMR (400 MHz, Chloroform-*d*) δ 7.48 (dd, *J* = 3.7, 1.2 Hz, 1H), 7.45 (dd, *J* = 5.0, 1.2 Hz, 1H), 7.07 (dd, *J* = 5.0, 3.7 Hz, 1H), 5.95 (s, 1H), 3.40 (td, *J* = 7.3, 5.8 Hz, 2H), 1.75 – 1.59 (m, 7H), 1.27 – 1.07 (m, 6H), 0.92 – 0.82 (m, 2H). ^{13}C NMR (101 MHz, Chloroform-*d*) δ 129.6, 127.8, 127.5, 40.3, 37.4, 34.6, 33.3, 27.1, 26.6, 26.3.

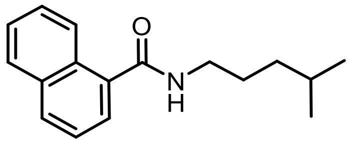

N-(4-methylpentyl)-1-naphthamide (**1s**)

The title compound was synthesized according to the reported literature[7] and the characterization data is consistent with the reported data. ^{1}H NMR (400 MHz, Chloroform-*d*) δ 8.34 – 8.26 (m, 1H), 7.94 – 7.82 (m, 2H), 7.61 – 7.49 (m, 3H), 7.45 (dd, *J* = 8.3, 7.0 Hz, 1H), 5.99 (s, 1H), 3.52 (td, *J* = 7.2, 5.8 Hz, 2H), 1.69 – 1.60 (m, 3H), 1.34 – 1.26 (m, 2H), 0.92 (d, *J* = 6.6 Hz, 6H). ^{13}C NMR (101 MHz, Chloroform-*d*) δ 169.5, 134.9, 133.7, 130.4, 130.1, 128.3, 127.0, 126.4, 125.4, 124.7, 40.3, 36.1, 27.8, 27.6, 22.6.

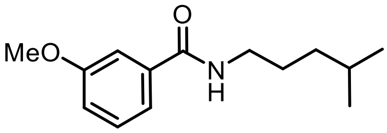

3-methoxy-N-(4-methylpentyl)benzamide (**1t**)

The title compound was synthesized according to the reported literature[7] and the characterization data is consistent with the reported data. ^{1}H NMR (400 MHz, Chloroform-*d*) δ 7.37 – 7.33 (m, 1H), 7.31 (d, *J* = 7.9 Hz, 1H), 7.27 – 7.26 (m, 1H), 7.08 – 6.97 (m, 1H), 6.12 (s, 1H), 3.85 (s, 3H), 3.43 (td, *J* = 7.2, 5.7 Hz, 2H), 1.65 – 1.54 (m, 3H), 1.29 – 1.23 (m, 2H), 0.90 (d, *J* = 6.6 Hz, 6H). ^{13}C NMR (101

MHz, Chloroform-*d*) δ 167.3, 159.8, 136.4, 129.5, 118.5, 117.5, 112.3, 55.4, 40.4, 36.1, 27.8, 27.6, 22.5.

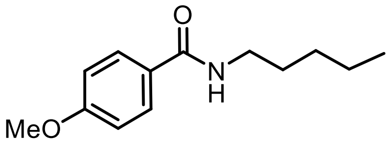

4-methoxy-N-(pentyl)benzamide (**1u**)

The title compound was synthesized according to the reported literature[7] and the characterization data is consistent with the reported data. ^{1}H NMR (400 MHz, Chloroform-*d*) δ 7.79 – 7.64 (m, 2H), 6.99 – 6.84 (m, 2H), 6.08 (s, 1H), 3.84 (s, 3H), 3.42 (td, J = 7.2, 5.7 Hz, 2H), 1.66 – 1.54 (m, 2H), 1.43 – 1.27 (m, 4H), 0.91 (t, J = 7.0 Hz, 3H). ^{13}C NMR (101 MHz, Chloroform-*d*) δ 167.0, 162.0, 128.6, 127.1, 113.7, 55.4, 40.0, 29.4, 29.1, 22.4, 14.0.

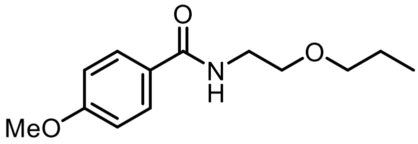

4-methoxy-N-(2-(propoxy)ethyl)benzamide (**1v**)

The title compound was synthesized according to the reported literature[7] and the characterization data is consistent with the reported data. ^{1}H NMR (400 MHz, Chloroform-*d*) δ 7.77 – 7.71 (m, 2H), 6.93 – 6.87 (m, 2H), 6.52 (s, 1H), 3.82 (s, 3H), 3.65 – 3.59 (m, 2H), 3.59 – 3.55 (m, 2H), 3.41 (t, J = 6.7 Hz, 2H), 1.59 (h, J = 7.2 Hz, 2H), 0.92 (t, J = 7.4 Hz, 3H). ^{13}C NMR (101 MHz, Chloroform-*d*) δ 166.9, 162.1, 128.7, 126.9, 113.7, 72.8, 69.3, 55.4, 39.7, 22.8, 10.5.

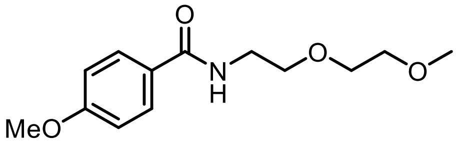

4-methoxy-N-(2-(2-methoxyethoxy)ethyl)benzamide (**1w**)

The title compound was synthesized according to the reported literature[7] and the characterization data is consistent with the reported data. ^{1}H NMR (400 MHz, Chloroform-*d*) δ 7.78 – 7.71 (m, 2H), 6.93 – 6.85 (m, 2H), 6.69 (s, 1H), 3.82 (s, 3H), 3.68 – 3.58 (m, 6H), 3.57 – 3.50 (m, 2H), 3.36 (s, 3H). ^{13}C NMR (101 MHz, Chloroform-*d*) δ 166.9, 162.0, 128.7, 126.8, 113.6, 71.8, 70.1, 69.8, 58.9, 55.3, 39.5.

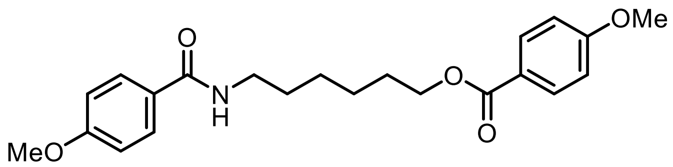

6-(4-methoxybenzamido)hexyl 4-methoxybenzoate (**1x**)

The title compound was synthesized according to the reported literature[7] and the characterization data is consistent with the reported data. ^{1}H NMR (400 MHz, Chloroform-*d*) δ 8.01 – 7.92 (m, 2H), 7.78 – 7.69 (m, 2H), 6.93 – 6.83 (m, 4H), 6.36 (t, J = 5.7 Hz, 1H), 4.26 (t, J = 6.6 Hz, 2H), 3.82 (d, J = 8.2 Hz, 6H), 3.46 – 3.35 (m, 2H), 1.79 – 1.68 (m, 2H), 1.65 – 1.57 (m, 2H), 1.52 – 1.35 (m, 4H). ^{13}C NMR (101 MHz, Chloroform-*d*) δ 167.0, 166.4, 163.2, 162.0, 131.5, 128.6, 127.0, 122.8, 113.7, 113.5, 64.5, 55.4, 55.3, 39.8, 29.6, 28.7, 26.6, 25.7.

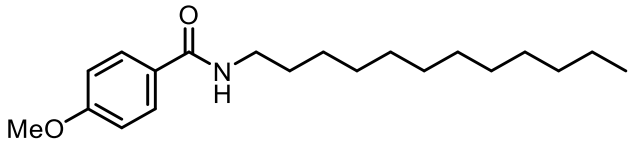

N-(dodecyl)-4-methoxybenzamide (**1y**)

The title compound was synthesized according to the reported literature[7] and the characterization data is consistent with the reported data. ^{1}H NMR (400 MHz, Chloroform-*d*) δ 7.76 – 7.69 (m, 2H), 6.92 – 6.85 (m, 2H), 6.25 (t, J = 5.8 Hz, 1H), 3.82 (s, 3H), 3.40 (td, J = 7.3, 5.8 Hz, 2H), 1.58 (p, J = 7.3 Hz, 2H), 1.38 – 1.21 (m, 18H), 0.87 (t, J = 6.8 Hz, 3H). ^{13}C NMR (101 MHz, Chloroform-*d*) δ 167.0, 161.9, 128.6, 127.1, 113.6, 55.3, 40.0, 31.9, 29.7, 29.6, 29.6, 29.5, 29.5, 29.3, 27.0, 22.6, 14.1.

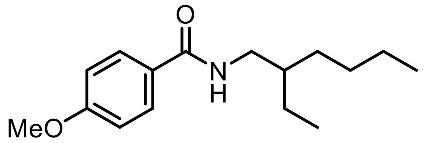

N-(2-ethylhexyl)-4-methoxybenzamide (**1z**)

The title compound was synthesized according to the reported literature[7] and the characterization data is consistent with the reported data. ^{1}H NMR (400 MHz, Chloroform-*d*) δ 7.80 – 7.67 (m, 2H), 6.92 (d, J = 8.5 Hz, 2H), 6.02 (s, 1H), 3.84 (s, 3H), 3.49 – 3.29 (m, 2H), 1.61 – 1.49 (m, 1H), 1.44 – 1.21 (m, 8H), 1.00 – 0.77 (m, 6H). ^{13}C NMR (101 MHz, Chloroform-*d*) δ 167.1, 162.0, 128.5, 127.2, 113.7, 55.4, 42.8, 39.5, 31.1, 28.9, 24.4, 23.0, 14.1, 10.9.

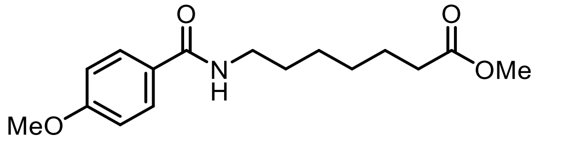

methyl 7-(4-methoxybenzamido)heptanoate (**1aa**)

The title compound was synthesized according to the reported literature[7] and the characterization data is consistent with the reported data. ^{1}H NMR (400 MHz, Chloroform-*d*) δ 7.76 – 7.66 (m, 2H), 6.94 – 6.86 (m, 2H), 6.07 (s, 1H), 3.84 (s, 3H), 3.66 (s, 3H), 3.43 – 3.36 (m, 2H), 2.31 (t, *J* = 7.4 Hz, 2H), 1.67 – 1.60 (m, 4H), 1.43 – 1.33 (m, 4H). ^{13}C NMR (101 MHz, Chloroform-*d*) δ 174.2, 167.0, 162.0, 128.6, 127.1, 113.7, 55.4, 51.5, 39.9, 33.9, 29.5, 28.7, 26.6, 24.8.

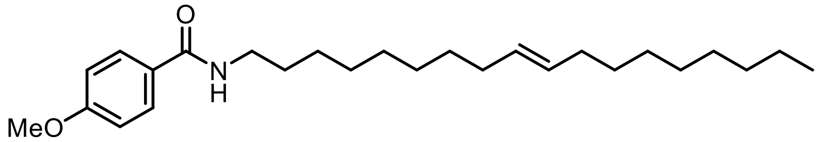

(E)-4-methoxy-N-(octadec-9-en-1-yl)benzamide (**1bb**)

The title compound was synthesized according to the reported literature[7] and the characterization data is consistent with the reported data. ^{1}H NMR (400 MHz, Chloroform-*d*) δ 7.72 (d, *J* = 8.5 Hz, 2H), 6.90 (d, *J* = 8.5 Hz, 2H), 6.24 – 5.99 (m, 1H), 5.47 – 5.22 (m, 2H), 3.83 (s, 3H), 3.48 – 3.35 (m, 2H), 2.04 – 1.78 (m, 4H), 1.64 – 1.53 (m, 2H), 1.36 – 1.22 (m, 22H), 0.87 (t, *J* = 6.6 Hz, 3H). ^{13}C NMR (101 MHz, Chloroform-*d*) δ 167.0, 162.0, 130.4, 129.9, 128.6, 127.1, 113.6, 55.3, 40.0, 32.6, 31.9, 29.7, 29.7, 29.6, 29.6, 29.5, 29.5, 29.4, 29.3, 29.2, 27.2, 27.0, 22.6, 14.1.

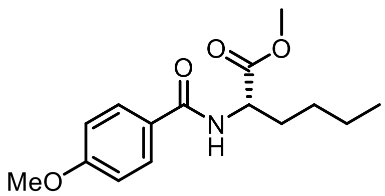

methyl (2S)-2-(4-methoxybenzamido)hexanoate (**1cc**)

The title compound was synthesized according to the reported literature[11] and the characterization data is consistent with the reported data. ^{1}H NMR (400 MHz, Chloroform-*d*) δ 7.87 – 7.72 (m, 2H), 7.00 – 6.86 (m, 2H), 6.56 (d, *J* = 7.9 Hz, 1H), 4.81 (td, *J* = 7.4, 5.4 Hz, 1H), 3.85 (s, 3H), 3.78 (s, 3H), 2.01 – 1.89 (m, 1H), 1.83 – 1.72 (m, 1H), 1.42 – 1.28 (m, 4H), 0.89 (t, *J* = 7.0 Hz, 3H). ^{13}C NMR (101 MHz, Chloroform-*d*) δ 173.4, 166.5, 162.4, 128.9, 126.3, 113.8, 55.4, 52.5, 52.4, 32.5, 27.4, 22.3, 13.9.

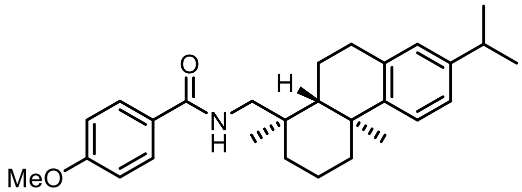

N-(((1R,4aS,10aR)-7-isopropyl-1,4a-dimethyl-1,2,3,4,4a,9,10,10a-octahydrophenanthren-1-yl)methyl)-4-methoxybenzamide (**1dd**)

The title compound was synthesized according to the reported literature[7] and the characterization data is consistent with the reported data. ^{1}H NMR (400 MHz, Chloroform-*d*) δ 7.74 – 7.67 (m, 2H), 7.17 (d, *J* = 8.2 Hz, 1H), 6.99 (dd, *J* = 8.1, 2.0 Hz, 1H), 6.94 – 6.85 (m, 3H), 6.03 (s, 1H), 3.83 (s, 3H), 3.45 – 3.29 (m, 2H), 2.98 – 2.87 (m, 1H), 2.88 – 2.76 (m, 2H), 2.36 – 2.24 (m, 1H), 2.02 – 1.94 (m, 1H), 1.84 – 1.64 (m, 3H), 1.55 – 1.47 (m, 2H), 1.43 – 1.31 (m, 2H), 1.23 (d, *J* = 3.6 Hz, 6H), 1.21 (s, 3H), 1.00 (s, 3H). ^{13}C NMR (101 MHz, Chloroform-*d*) δ 167.1, 162.1, 147.0, 145.6, 134.8, 128.6, 127.1, 126.9, 124.2, 123.9, 113.8, 55.4, 50.2, 45.8, 38.4, 37.7, 37.6, 36.4, 33.4, 30.5, 25.4, 23.9, 19.1, 18.8, 18.6.

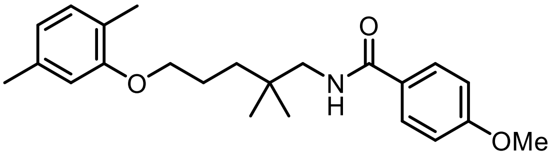

N-(5-(2,5-dimethylphenoxy)-2,2-dimethylpentyl)-4-methoxybenzamide (**1ee**)

The title compound was synthesized according to the reported literature[7]. ^{1}H NMR (400 MHz, Chloroform-*d*) δ 7.75 – 7.69 (m, 2H), 6.99 (d, *J* = 7.4 Hz, 1H), 6.95 – 6.89 (m, 2H), 6.68 – 6.61 (m, 2H), 6.09 (s, 1H), 3.94 (t, *J* = 6.2 Hz, 2H), 3.85 (s, 3H), 3.34 (d, *J* = 6.3 Hz, 2H), 2.30 (s, 3H), 2.15 (s, 3H), 1.87 – 1.78 (m, 2H), 1.51 – 1.44 (m, 2H), 1.00 (s, 6H). ^{13}C NMR (101 MHz, Chloroform-*d*) δ 167.1, 162.1, 156.9, 136.5, 130.3, 128.6, 127.2, 123.5, 120.7, 113.7, 112.1, 68.4, 55.4, 49.2, 36.1, 34.4, 25.1, 24.2, 21.4, 15.8.

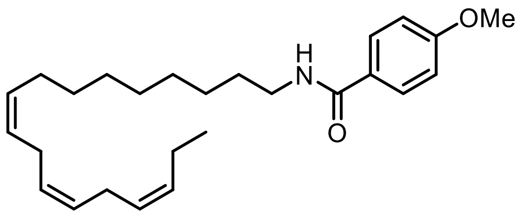

4-methoxy-N-((9Z,12Z,15Z)-octadeca-9,12,15-trien-1-yl)benzamide (**1ff**)

The title compound was synthesized according to the reported literature[7]. ^{1}H NMR (400 MHz, Chloroform-*d*) δ 7.76 – 7.69 (m, 2H), 6.95 – 6.87 (m, 2H), 6.19 – 6.06 (m, 1H), 5.50 – 5.23 (m, 6H), 3.83 (s, 3H), 3.47 – 3.37 (m, 2H), 2.88 – 2.63 (m, 4H), 2.14 – 1.99 (m, 4H), 1.65 – 1.53 (m, 2H), 1.39 – 1.28 (m, 10H), 0.97 (t, *J* = 7.5 Hz, 3H). ^{13}C NMR (101 MHz, Chloroform-*d*) δ 166.9, 162.0, 131.9, 130.3, 128.6, 128.2, 127.7, 127.1, 127.1, 113.6, 55.3, 40.0, 29.7, 29.6, 29.4, 29.3, 29.2, 27.2, 27.0, 25.6, 25.5, 20.5, 14.2.

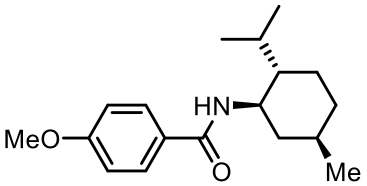

N-((1R,2S,5R)-2-isopropyl-5-methylcyclohexyl)-4-methoxybenzamide (**1gg**)

The title compound was synthesized according to the reported literature[7]. ^{1}H NMR (400 MHz, Chloroform-*d*) δ 7.77 – 7.67 (m, 2H), 6.97 – 6.89 (m, 2H), 6.08 (d, *J* = 9.0 Hz, 1H), 4.59 – 4.50 (m, 1H), 3.85 (s, 3H), 2.04 – 1.96 (m, 1H), 1.96 – 1.89 (m, 1H), 1.83 – 1.75 (m, 1H), 1.56 – 1.45 (m, 1H), 1.44 – 1.35 (m, 1H), 1.18 – 0.96 (m, 4H), 0.96 – 0.87 (m, 9H). ^{13}C NMR (101 MHz, Chloroform-*d*) δ 166.1, 162.0, 128.5, 127.6, 113.8, 55.4, 46.5, 46.4, 40.1, 34.8, 29.9, 27.1, 25.7, 22.2, 21.0, 20.9.

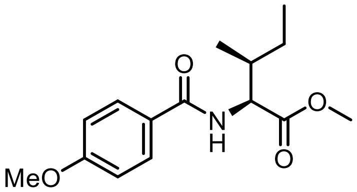

methyl (4-methoxybenzoyl)-L-isoleucinate (**1hh**)

The title compound was synthesized according to the reported literature[11] and the characterization data is consistent with the reported data. ^{1}H NMR (400 MHz, Chloroform-*d*) δ 7.82 – 7.73 (m, 2H), 6.93 (d, *J* = 8.9 Hz, 2H), 6.56 (d, *J* = 8.5 Hz, 1H), 4.80 (dd, *J* = 8.5, 5.0 Hz, 1H), 3.85 (s, 3H), 3.76 (s, 3H), 2.05 – 1.94 (m, 1H), 1.60 – 1.47 (m, 1H), 1.31 – 1.18 (m, 1H), 1.01 – 0.87 (m, 6H). ^{13}C NMR (101 MHz, Chloroform-*d*) δ 172.8, 166.5, 162.3, 128.8, 126.4, 113.8, 56.7, 55.4, 52.1, 38.3, 25.4, 15.5, 11.6.

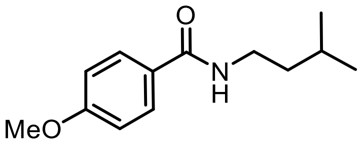

4-methoxy-N-(3-methylbutyl)benzamide (**1ii**)

The title compound was synthesized according to the reported literature[7] and the characterization data is consistent with the reported data. ^{1}H NMR (400 MHz, Chloroform-*d*) δ 7.80 – 7.60 (m, 2H), 6.97 – 6.82 (m, 2H), 6.03 (s, 1H), 3.84 (s, 3H), 3.58 – 3.31 (m, 2H), 1.71 – 1.61 (m, 1H), 1.57 – 1.38 (m, 2H), 0.95 (d, *J* = 6.6 Hz, 6H). ^{13}C NMR (101 MHz, Chloroform-*d*) δ 167.0, 162.0, 128.5, 127.1, 113.7, 55.4, 38.6, 38.3, 26.0, 22.5.

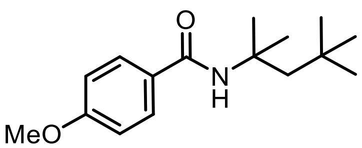

4-methoxy-N-(2,4,4-trimethylpentan-2-y)benzamide (**1jj**)

The title compound was synthesized according to the reported literature[7] and the characterization data is consistent with the reported data. ^{1}H NMR (400 MHz, Chloroform-*d*) δ 7.75 – 7.52 (m, 2H), 7.00 – 6.80 (m, 2H), 5.87 (s, 1H), 3.83 (s, 3H), 1.85 (s, 2H), 1.52 (s, 6H), 1.04 (s, 9H). ^{13}C NMR (101 MHz, Chloroform-*d*) δ 166.2, 161.8, 128.5, 128.3, 113.7, 55.4, 51.8, 31.7, 31.6, 29.3.

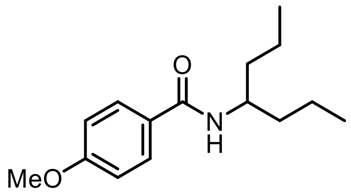

N-(heptan-4-y)-4-methoxybenzamide (**1kk**)

The title compound was synthesized according to the reported literature[7] and the characterization data is consistent with the reported data. ^{1}H NMR (400 MHz, Chloroform-*d*) δ 7.79 – 7.65 (m, 2H), 6.98 – 6.86 (m, 2H), 5.69 (d, *J* = 9.1 Hz, 1H), 4.20 – 4.09 (m, 1H), 3.84 (s, 3H), 1.60 – 1.51 (m, 2H), 1.48 – 1.30 (m, 6H), 0.93 (t, *J* = 7.1 Hz, 6H). ^{13}C NMR (101 MHz, Chloroform-*d*) δ 166.6, 162.0, 128.5, 127.4, 113.7, 55.4, 49.2, 37.7, 19.2, 14.1.

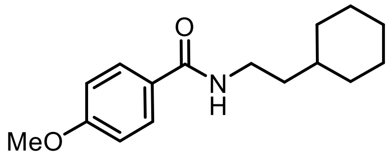

N-(2-(cyclohexyl)ethyl)-4-methoxybenzamide (**1-a**)

The title compound was synthesized according to the reported literature[8] and the characterization data is consistent with the reported data. ^{1}H NMR (400 MHz, Chloroform-*d*) δ 7.75 – 7.69 (m, 2H), 6.94 – 6.89 (m, 2H), 5.96 (s, 1H), 3.84 (s, 3H), 3.50 – 3.42 (m, 2H), 1.78 – 1.63 (m, 5H), 1.55 – 1.46 (m, 2H), 1.40 – 1.30 (m, 1H), 1.28 – 1.12 (m, 3H), 1.01 – 0.88 (m, 2H). ^{13}C NMR (101 MHz, Chloroform-*d*) δ 166.9, 162.0, 128.6, 127.2, 113.7, 55.4, 37.9, 37.2, 35.5, 33.2, 26.5, 26.2.

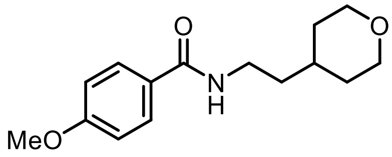

4-methoxy-N-(2-((3R,5R)-tetrahydro-2H-pyran-4-yl)ethyl)benzamide (**1-b**)

The title compound was synthesized according to the reported literature[8] and the characterization data is consistent with the reported data. ^{1}H NMR (400 MHz, Chloroform-*d*) δ 7.79 – 7.66 (m, 2H), 6.98 – 6.82 (m, 2H), 6.13 (t, *J* = 5.4 Hz, 1H), 4.02 – 3.89 (m, 2H), 3.83 (s, 3H), 3.53 – 3.42 (m, 2H),

3.36 (td, J = 11.8, 1.9 Hz, 2H), 1.69 – 1.49 (m, 5H), 1.37 – 1.26 (m, 2H). ^{13}C NMR (101 MHz, Chloroform-*d*) δ 167.0, 162.1, 128.6, 126.9, 113.7, 67.9, 55.4, 37.3, 36.8, 32.9, 32.8.

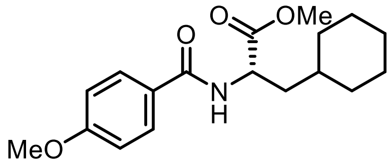

methyl (2S)-3-(cyclohexyl)-2-(4-methoxybenzamido)propanoate (**1-c**)

The title compound was synthesized according to the reported literature[8] and the characterization data is consistent with the reported data. ^{1}H NMR (400 MHz, Chloroform-*d*) δ 7.78 – 7.73 (m, 2H), 6.92 – 6.87 (m, 2H), 6.53 (d, J = 8.2 Hz, 1H), 4.85 (td, J = 8.7, 5.3 Hz, 1H), 3.83 (s, 3H), 3.74 (s, 3H), 1.87 – 1.59 (m, 7H), 1.42 – 1.33 (m, 1H), 1.26 – 1.10 (m, 3H), 1.02 – 0.87 (m, 2H). ^{13}C NMR (101 MHz, Chloroform-*d*) δ 174.0, 166.5, 162.3, 128.9, 126.2, 113.7, 55.4, 52.3, 50.4, 40.3, 34.2, 33.5, 32.6, 26.3, 26.1, 26.0.

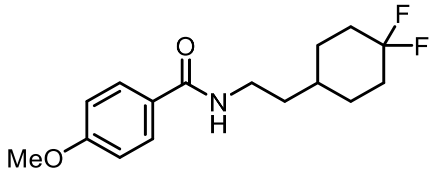

N-(2-(4,4-difluorocyclohexyl)ethyl)-4-methoxybenzamide (**1-d**)

The title compound was synthesized according to the reported literature[8] and the characterization data is consistent with the reported data. ^{1}H NMR (400 MHz, Chloroform-*d*) δ 7.80 – 7.66 (m, 2H), 7.00 – 6.86 (m, 2H), 6.02 (s, 1H), 3.85 (s, 3H), 3.53 – 3.43 (m, 2H), 2.13 – 2.03 (m, 2H), 1.90 – 1.80 (m, 2H), 1.79 – 1.63 (m, 2H), 1.60 – 1.53 (m, 2H), 1.50 – 1.39 (m, 1H), 1.37 – 1.28 (m, 2H). ^{13}C NMR (101 MHz, Chloroform-*d*) δ 167.0, 162.1, 128.6, 126.9, 113.8, 55.4, 37.9, 35.8, 33.6 (d, J = 8.3 Hz), 33.3 (d, J = 23.2 Hz), 28.9 (d, J = 9.5 Hz). ^{19}F NMR (376 MHz, Chloroform-*d*) δ -91.7 (d, J = 235.0 Hz), -102.0 (d, J = 235.1 Hz).

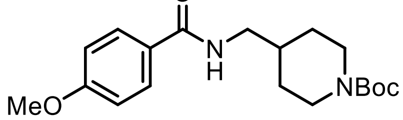

tert-butyl 4-((4-methoxybenzamido)methyl)piperidine-1-carboxylate (**1-e**)

The title compound was synthesized according to the reported literature[8] and the characterization data is consistent with the reported data. ^{1}H NMR (400 MHz, Chloroform-*d*) δ 7.80 – 7.61 (m, 2H), 6.98 – 6.84 (m, 2H), 6.25 (s, 1H), 4.23 – 3.99 (m, 2H), 3.84 (s, 3H), 3.32 (s, 2H), 2.68 (t, J = 12.7 Hz,

2H), 1.82 – 1.75 (m, 1H), 1.73 – 1.68 (m, 2H), 1.44 (s, 9H), 1.24 – 1.10 (m, 2H). ^{13}C NMR (101 MHz, Chloroform-*d*) δ 167.1, 162.1, 154.8, 128.6, 126.8, 113.7, 79.4, 55.4, 45.3, 43.5, 36.5, 29.8, 28.4.

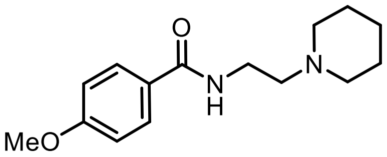

4-methoxy-N-(2-(piperidin-1-yl)ethyl)benzamide (**1-f**)

The title compound was synthesized according to the reported literature[12] and the characterization data is consistent with the reported data. ^{1}H NMR (400 MHz, Chloroform-*d*) δ 7.82 – 7.70 (m, 2H), 7.07 – 6.84 (m, 3H), 3.85 (s, 3H), 3.52 (q, *J* = 5.8 Hz, 2H), 2.55 (t, *J* = 6.0 Hz, 2H), 2.44 (s, 4H), 1.64 – 1.56 (m, 4H), 1.50 – 1.41 (m, 2H). ^{13}C NMR (101 MHz, Chloroform-*d*) δ 166.9, 162.0, 128.7, 127.0, 113.7, 57.1, 55.4, 54.3, 36.3, 26.0, 24.3.

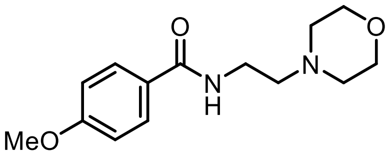

4-methoxy-N-(2-(morpholino)ethyl)benzamide (**1-g**)

The title compound was synthesized according to the reported literature[13] and the characterization data is consistent with the reported data. ^{1}H NMR (400 MHz, Chloroform-*d*) δ 7.79 – 7.68 (m, 2H), 6.98 – 6.89 (m, 2H), 6.68 (s, 1H), 3.85 (s, 3H), 3.77 – 3.69 (m, 4H), 3.54 (q, *J* = 5.6 Hz, 2H), 2.60 (t, *J* = 6.0 Hz, 2H), 2.51 (t, *J* = 4.7 Hz, 4H). ^{13}C NMR (101 MHz, Chloroform-*d*) δ 162.1, 128.7, 126.9, 113.7, 67.0, 57.0, 55.4, 53.3, 35.9.

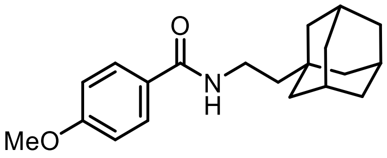

N-(2-((1S,3R,5S,7S,8S)-adamantan-1-yl)ethyl)-4-methoxybenzamide (**1-h**)

The title compound was synthesized according to the reported literature[13] and the characterization data is consistent with the reported data. ^{1}H NMR (400 MHz, Chloroform-*d*) δ 7.76 – 7.68 (m, 2H), 6.94 – 6.87 (m, 2H), 5.93 (s, 1H), 3.84 (s, 3H), 3.49 – 3.39 (m, 2H), 2.01 – 1.91 (m, 3H), 1.74 – 1.68 (m, 3H), 1.66 – 1.60 (m, 3H), 1.55 (d, *J* = 2.9 Hz, 6H), 1.41 – 1.35 (m, 2H). ^{13}C NMR (101 MHz, Chloroform-*d*) δ 166.9, 162.0, 128.5, 127.2, 113.7, 55.4, 44.0, 42.4, 37.0, 35.1, 32.0, 28.6.

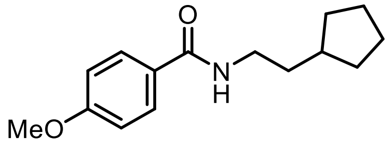

N-(2-(cyclopentyl)ethyl)-4-methoxybenzamide (**1-i**)

The title compound was synthesized according to the reported literature[7] and the characterization data is consistent with the reported data. ^{1}H NMR (400 MHz, Chloroform-*d*) δ 7.77 – 7.66 (m, 2H), 6.95 – 6.86 (m, 2H), 6.02 (s, 1H), 3.84 (s, 3H), , 3.50 – 3.40 (m, 2H), 1.93 – 1.73 (m, 3H), 1.66 – 1.60 (m, 4H), 1.57 – 1.49 (m, 2H), 1.19 – 1.09 (m, 2H). ^{13}C NMR (101 MHz, Chloroform-*d*) δ 166.9, 162.0, 128.6, 127.2, 113.7, 55.4, 39.5, 37.9, 36.0, 32.7, 25.1.

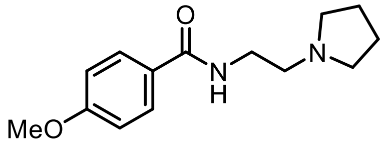

4-methoxy-N-(2-(pyrrolidin-1-yl)ethyl)benzamide (**1-j**)

The title compound was synthesized according to the reported literature[13] and the characterization data is consistent with the reported data. ^{1}H NMR (400 MHz, Chloroform-*d*) δ 7.80 – 7.69 (m, 2H), 6.95 – 6.85 (m, 2H), 6.76 (s, 1H), 3.92 – 3.76 (m, 3H), 3.59 – 3.44 (m, 2H), 2.73 – 2.63 (m, 2H), 2.60 – 2.47 (m, 4H), 1.85 – 1.64 (m, 4H). ^{13}C NMR (101 MHz, Chloroform-*d*) δ 167.0, 162.0, 128.7, 127.1, 113.6, 55.4, 54.6, 53.9, 38.5, 23.5.

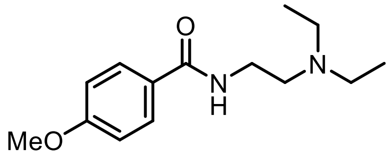

N-(2-(bis(ethyl)amino)ethyl)-4-methoxybenzamide (**1-k**)

The title compound was synthesized according to the reported literature[13] and the characterization data is consistent with the reported data. ^{1}H NMR (400 MHz, Chloroform-*d*) δ 7.82 – 7.63 (m, 2H), 6.97 (s, 1H), 6.94 – 6.87 (m, 2H), 3.83 (s, 3H), 3.50 – 3.45 (m, 2H), 2.67 – 2.63 (m, 2H), 2.57 (q, J = 7.1 Hz, 4H), 1.04 (t, J = 7.1 Hz, 6H). ^{13}C NMR (101 MHz, Chloroform-*d*) δ 166.8, 162.0, 128.6, 127.0, 113.6, 55.3, 51.4, 46.8, 37.1, 11.8.

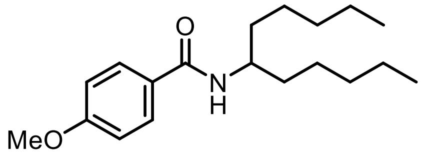

4-methoxy-N-(undecan-6-yl)benzamide (**1-l**)

The title compound was synthesized according to the reported literature[7] and the characterization data is consistent with the reported data. ^{1}H NMR (400 MHz, Chloroform-*d*) δ 7.78 – 7.66 (m, 2H), 6.96 – 6.83 (m, 2H), 5.84 (d, *J* = 9.0 Hz, 1H), 4.18 – 4.04 (m, 1H), 3.82 (s, 3H), 1.62 – 1.50 (m, 2H), 1.48 – 1.23 (m, 14H), 0.86 (t, *J* = 6.7 Hz, 6H). ^{13}C NMR (101 MHz, Chloroform-*d*) δ 166.5, 161.9, 128.5, 127.4, 113.6, 55.3, 49.7, 35.3, 31.8, 25.6, 22.5, 14.0.

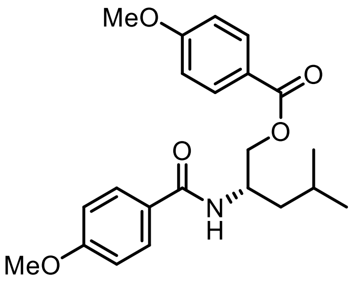

(S)-2-(4-methoxybenzamido)-4-(methyl-d)pentyl 4-methoxybenzoate (**1-m**)

The title compound was synthesized according to the reported literature[11] and the characterization data is consistent with the reported data. ^{1}H NMR (400 MHz, Chloroform-*d*) δ 8.02 – 7.94 (m, 2H), 7.75 – 7.68 (m, 2H), 6.95 – 6.87 (m, 4H), 6.19 (t, *J* = 7.5 Hz, 1H), 4.67 – 4.56 (m, 1H), 4.49 – 4.41 (m, 1H), 4.41 – 4.34 (m, 1H), 3.88 – 3.81 (m, 6H), 1.82 – 1.69 (m, 1H), 1.62 – 1.44 (m, 2H), 1.05 – 0.93 (m, 6H). ^{13}C NMR (101 MHz, Chloroform-*d*) δ 166.60, 163.51, 162.12, 132.81, 131.69, 128.65, 126.85, 122.21, 113.72, 113.69, 66.67, 55.43, 55.38, 47.41, 41.05, 24.98, 22.98, 22.42.

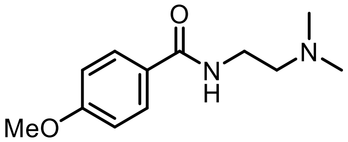

N-(2-(bis(methyl)amino)ethyl)-4-methoxybenzamide (**1-n**)

The title compound was synthesized according to the reported literature[13] and the characterization data is consistent with the reported data. ^{1}H NMR (400 MHz, Chloroform-*d*) δ 7.82 – 7.72 (m, 2H), 6.93 – 6.88 (m, 2H), 6.79 (s, 1H), 3.83 (s, 3H), 3.51 (q, *J* = 5.2 Hz, 2H), 2.52 (t, *J* = 5.9 Hz, 2H), 2.27 (s, 6H). ^{13}C NMR (101 MHz, Chloroform-*d*) δ 167.0, 162.0, 128.7, 126.9, 113.6, 57.8, 55.3, 45.1, 37.1.

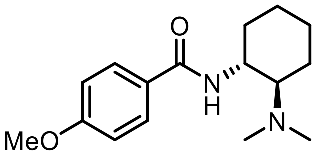

N-(2-(bis(methyl)amino)cyclohexyl)-4-methoxybenzamide (**1-o**)

The title compound was synthesized according to the reported literature[14] and the characterization data is consistent with the reported data. ^{1}H NMR (400 MHz, Chloroform-*d*) δ 7.83 – 7.73 (m, 2H),

7.07 (s, 1H), 6.94 – 6.87 (m, 2H), 3.83 (s, 3H), 3.69 – 3.61 (m, 1H), 2.68 – 2.60 (m, 1H), 2.58 – 2.48 (m, 1H), 2.28 (s, 6H), 1.98 – 1.78 (m, 2H), 1.75 – 1.67 (m, 1H), 1.44 – 1.31 (m, 1H), 1.29 – 1.21 (m, 2H), 1.21 – 1.10 (m, 1H). ^{13}C NMR (101 MHz, Chloroform-*d*) δ 167.4, 161.9, 128.9, 127.5, 113.5, 66.3, 55.3, 51.6, 39.7, 32.7, 25.3, 24.6, 21.4.

7. NMR spectra.

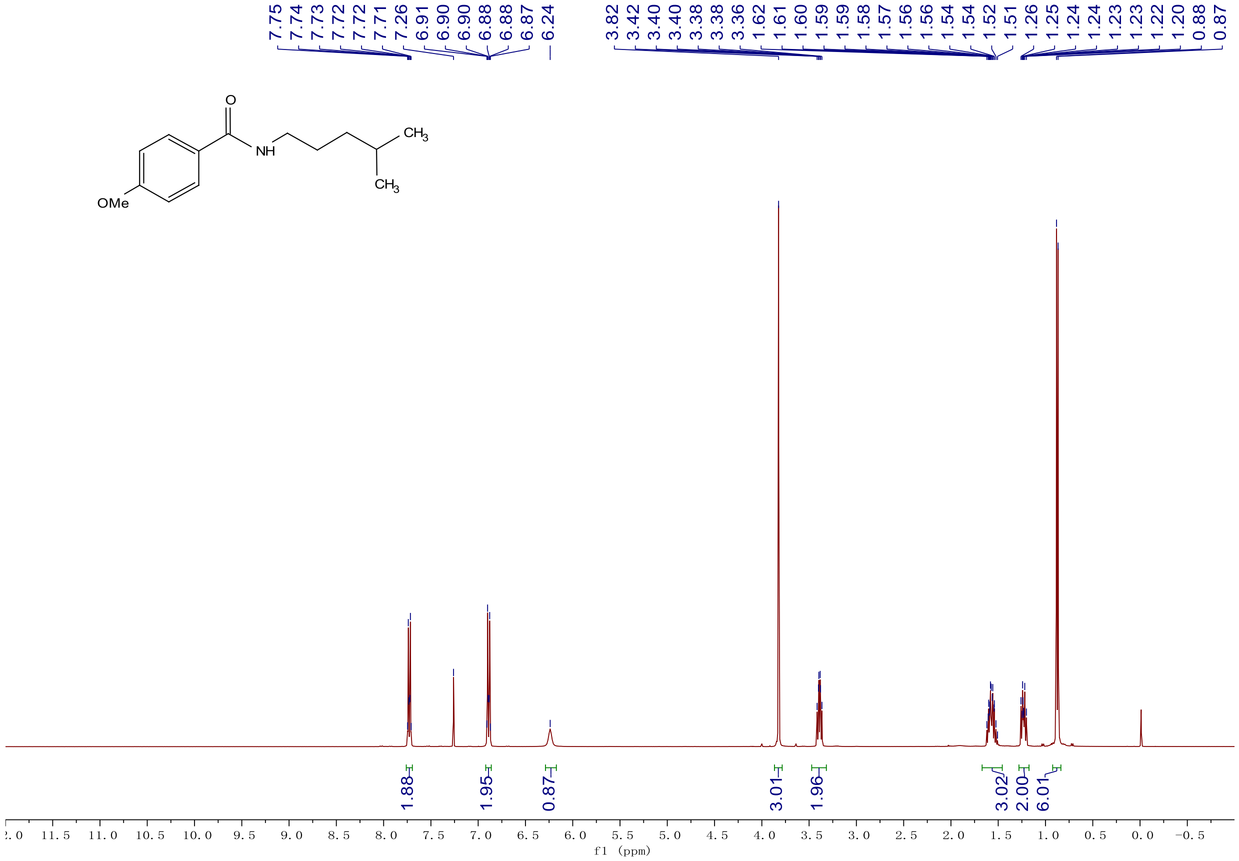

Supplementary Fig. 4. ^{1}H NMR (400 MHz, 298 K, Chloroform-*d*) spectrum of compound **1c**.

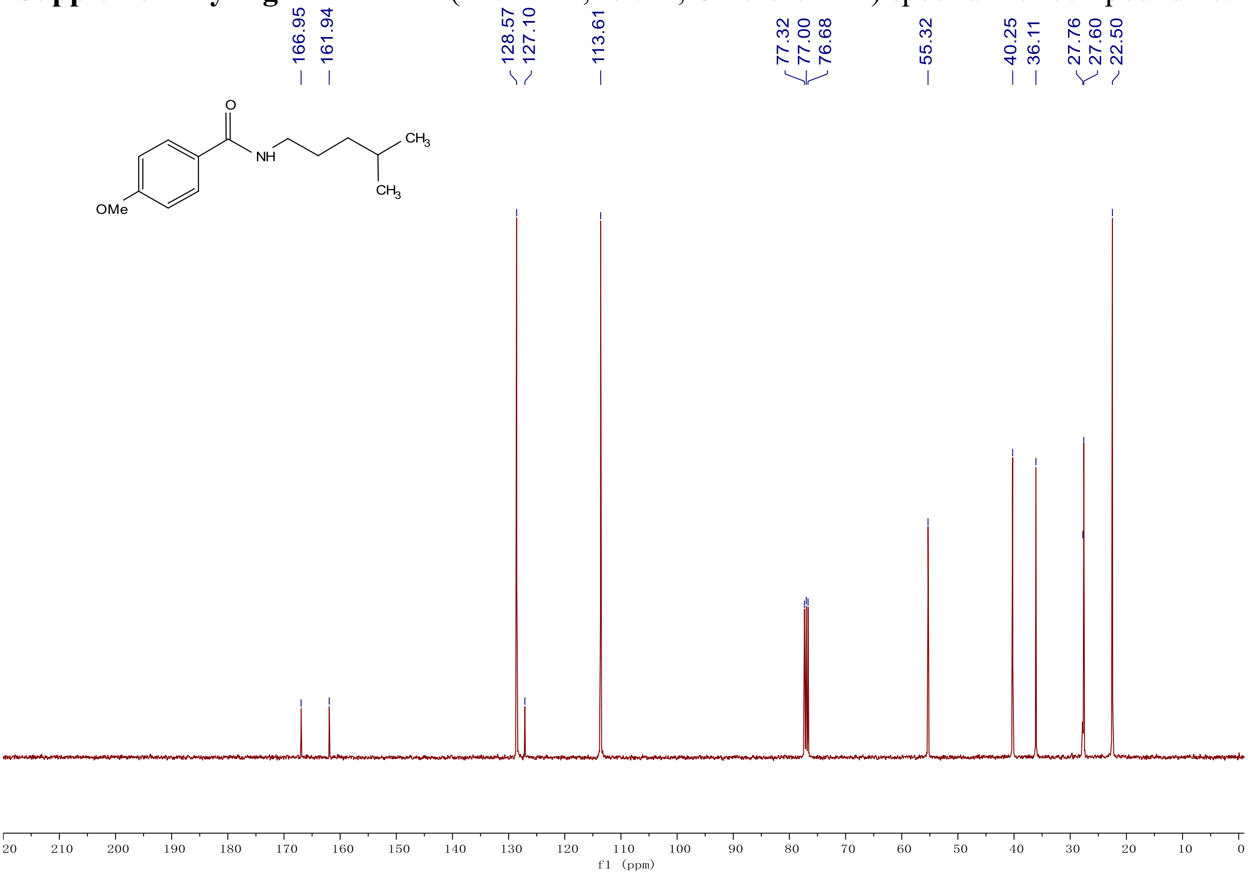

Supplementary Fig. 5. ^{13}C NMR (101 MHz, 298 K, Chloroform-*d*) spectrum of compound **1c**.

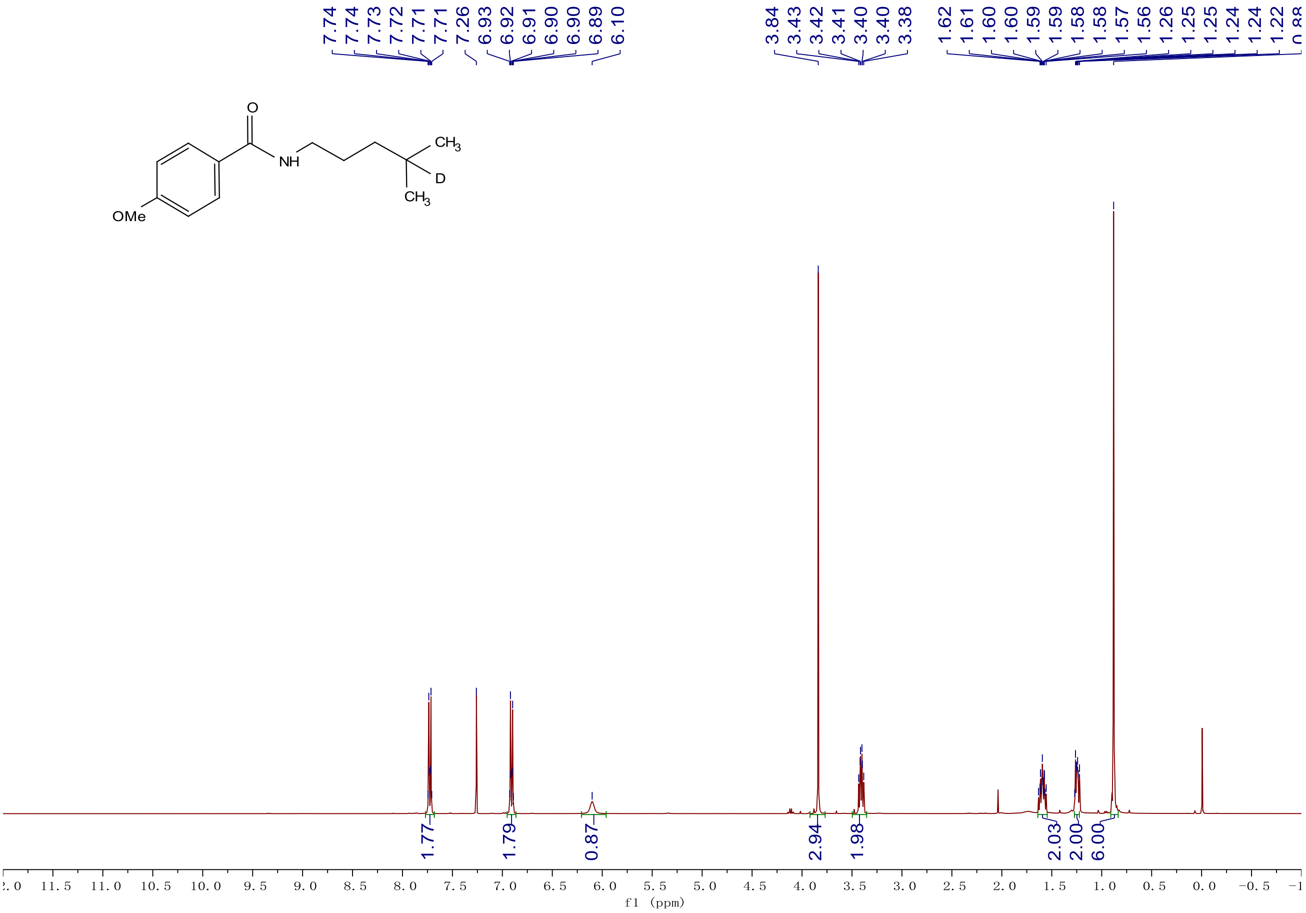

Supplementary Fig. 6. ^{1}H NMR (400 MHz, 298 K, Chloroform-*d*) spectrum of compound **3c**.

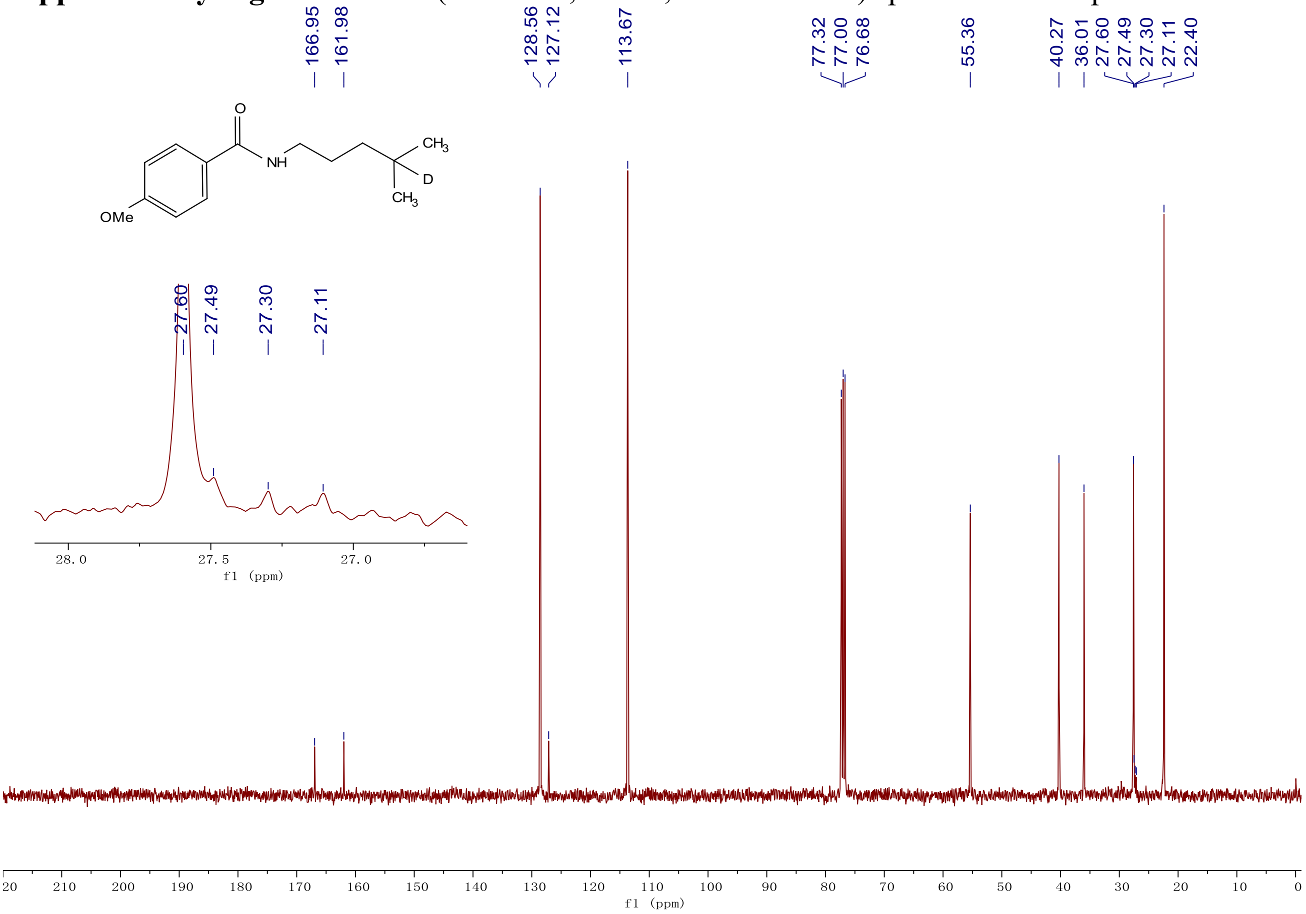

Supplementary Fig. 7. ^{13}C NMR (101 MHz, 298 K, Chloroform-*d*) spectrum of compound **3c**.

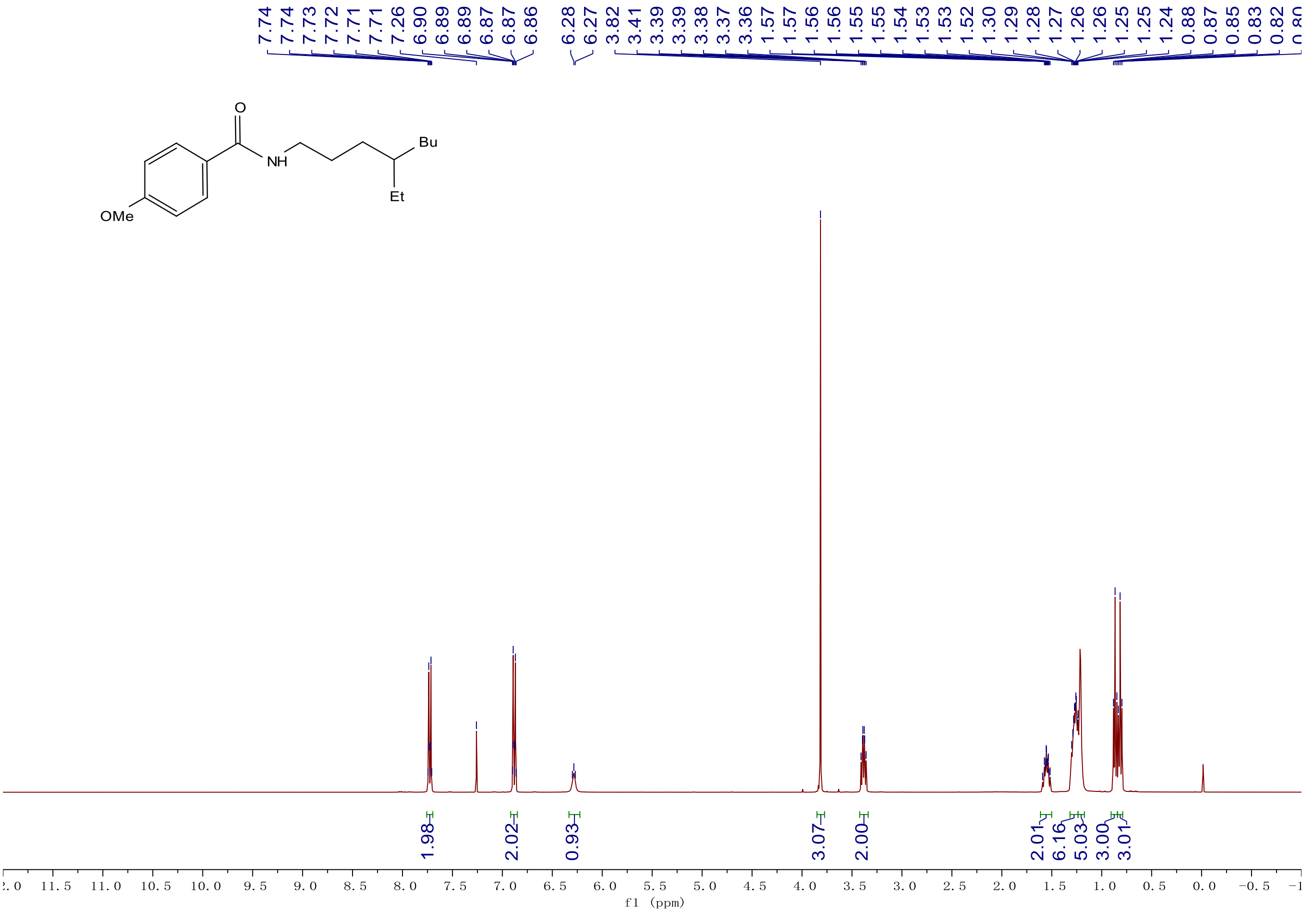

Supplementary Fig. 8. ^{1}H NMR (400 MHz, 298 K, Chloroform-*d*) spectrum of compound **1f**.

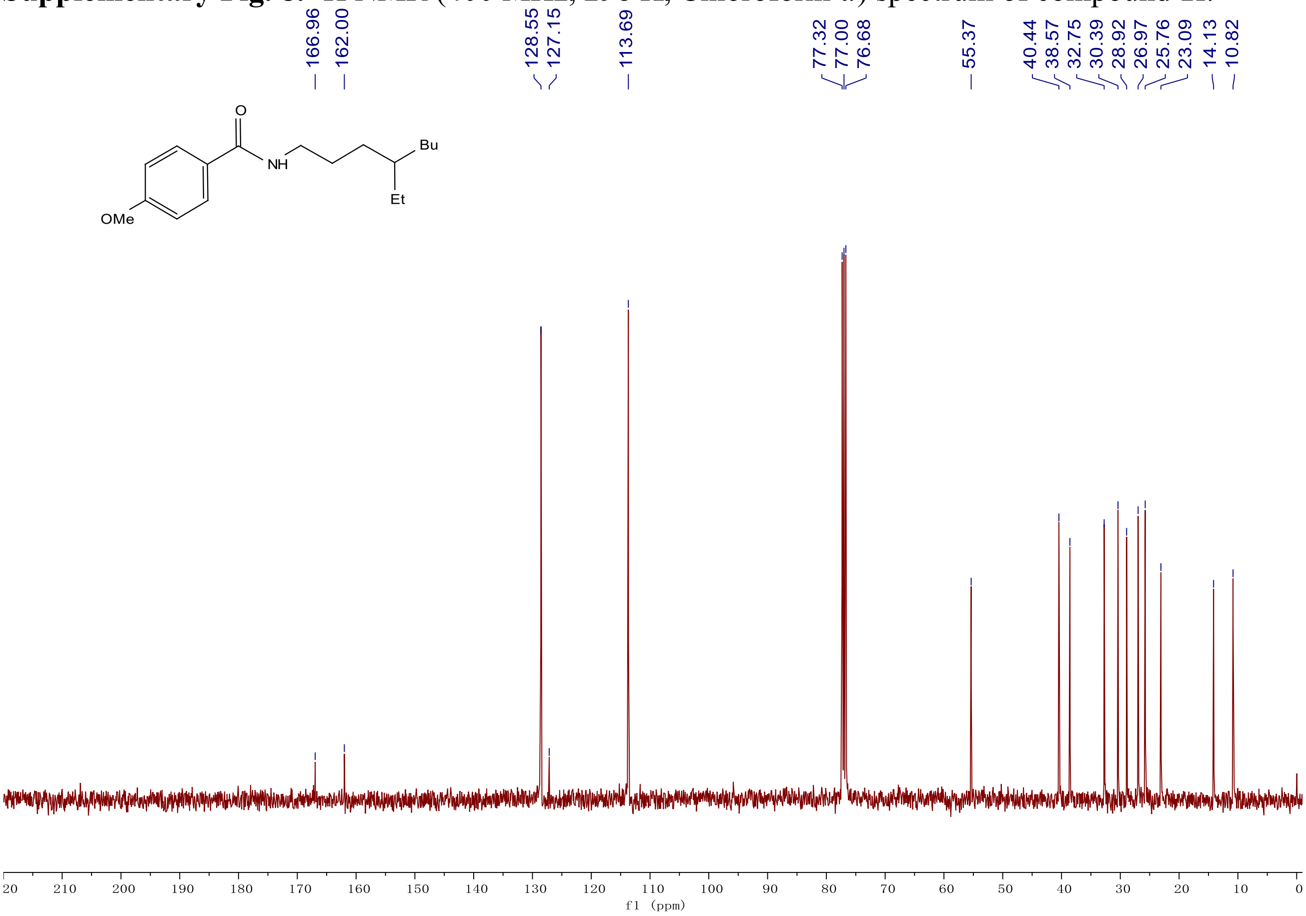

Supplementary Fig. 9. ^{13}C NMR (101 MHz, 298 K, Chloroform-*d*)spectrum of compound **1f**.

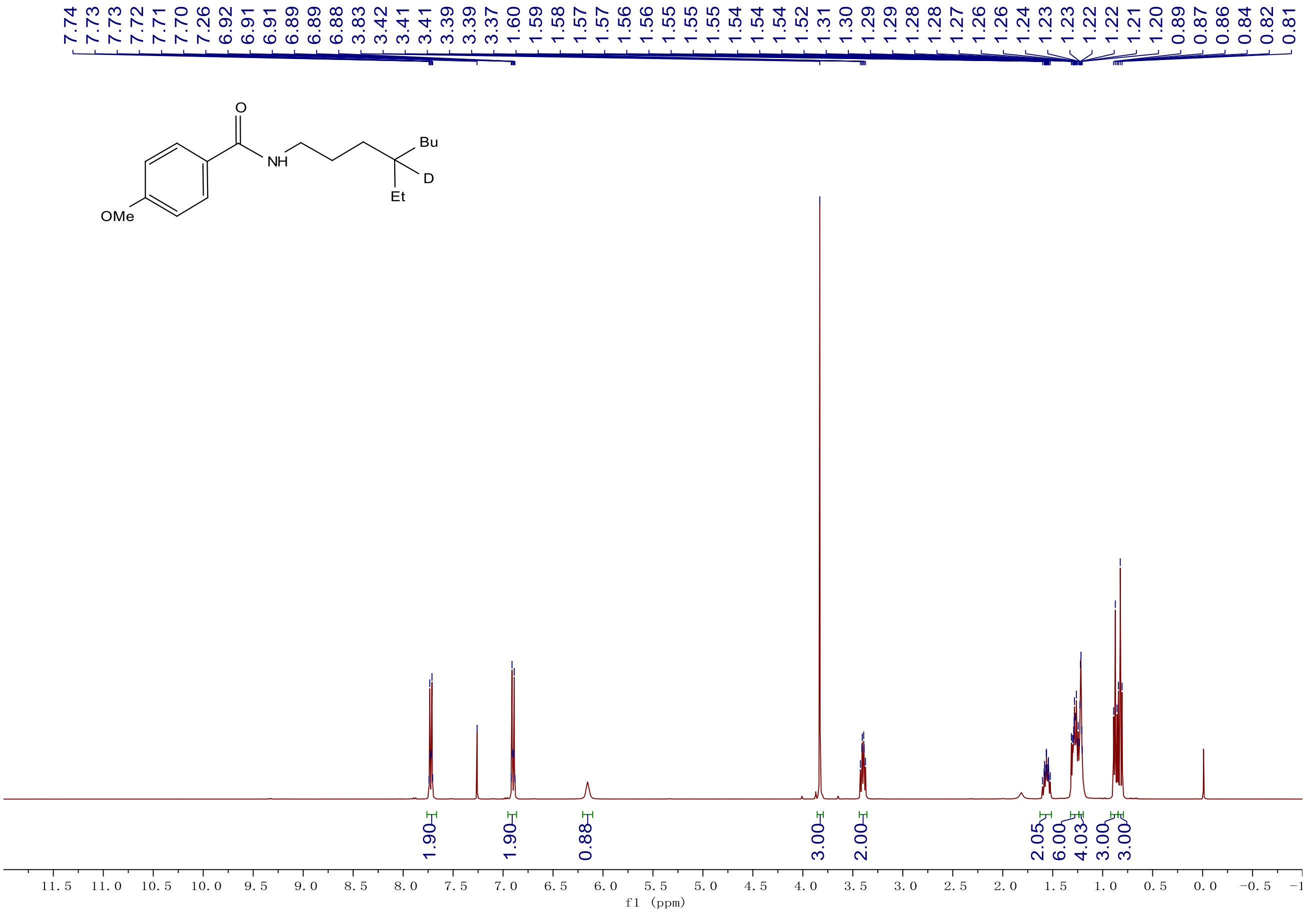

Supplementary Fig. 10. ^{1}H NMR (400 MHz, 298 K, Chloroform-d) spectrum of compound **3f**.

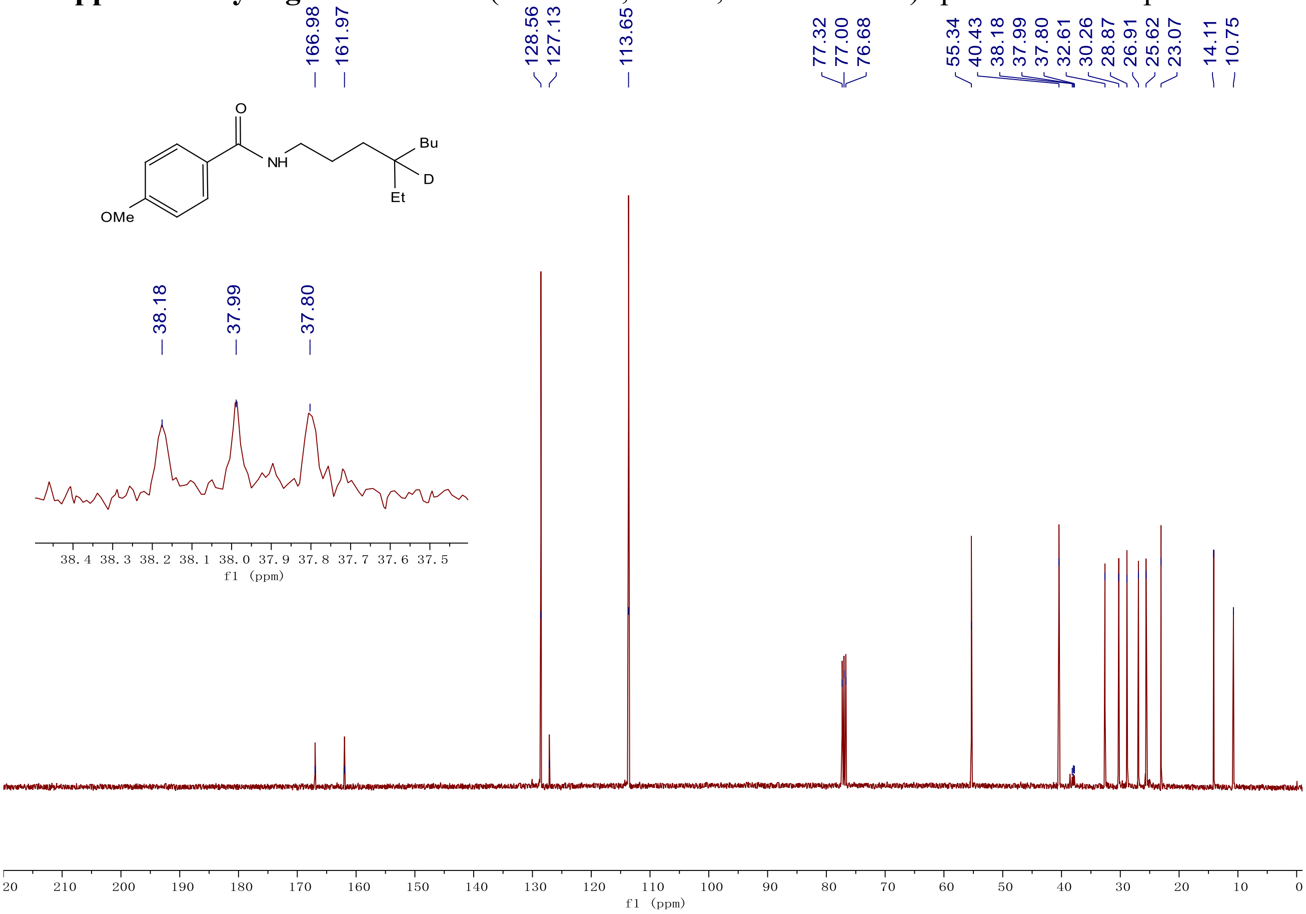

Supplementary Fig. 11. ^{13}C NMR (101 MHz, 298 K, Chloroform-d) spectrum of compound **3f**.

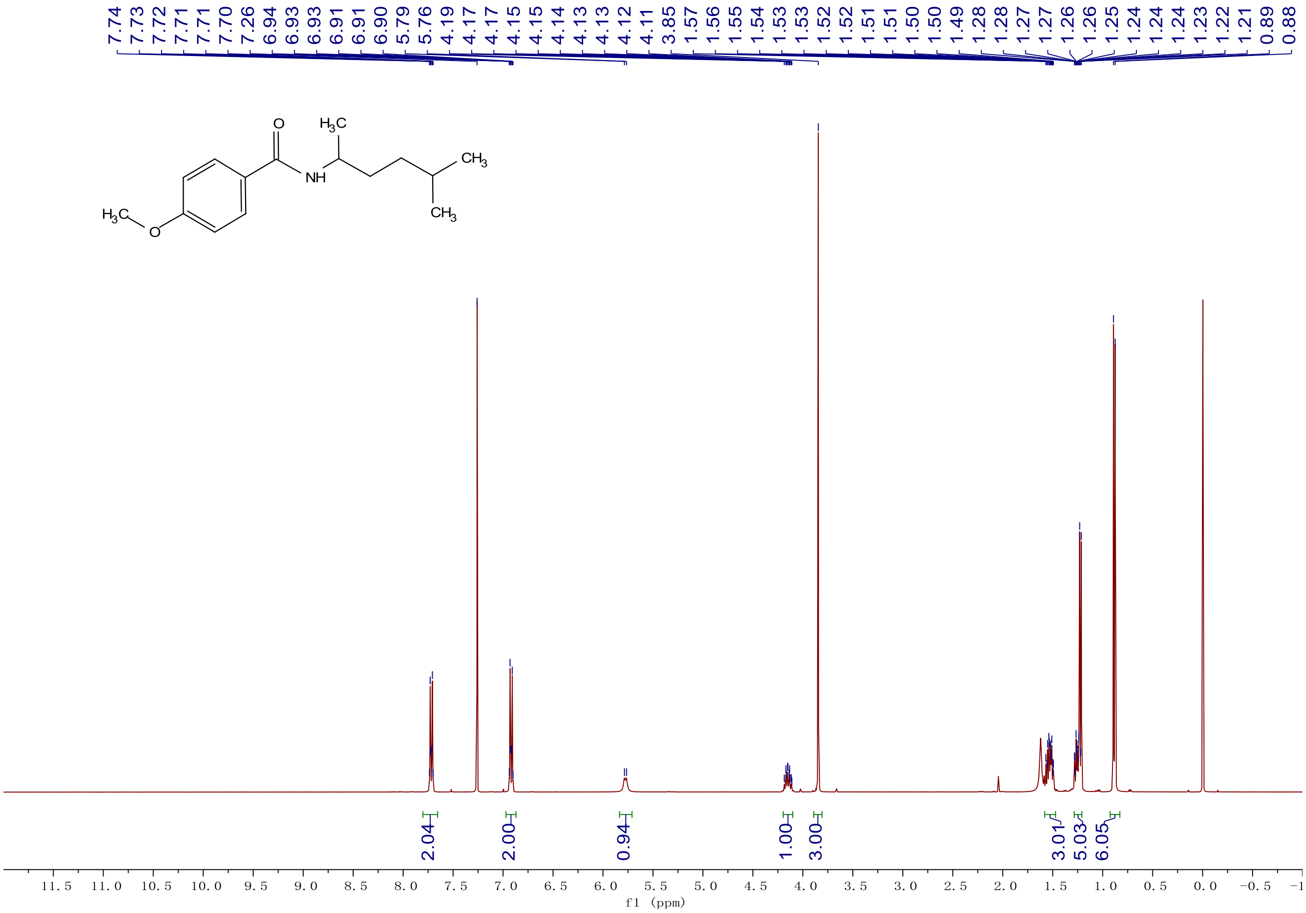

Supplementary Fig. 12. ^{1}H NMR (400 MHz, 298 K, Chloroform-*d*) spectrum of compound **1g**.

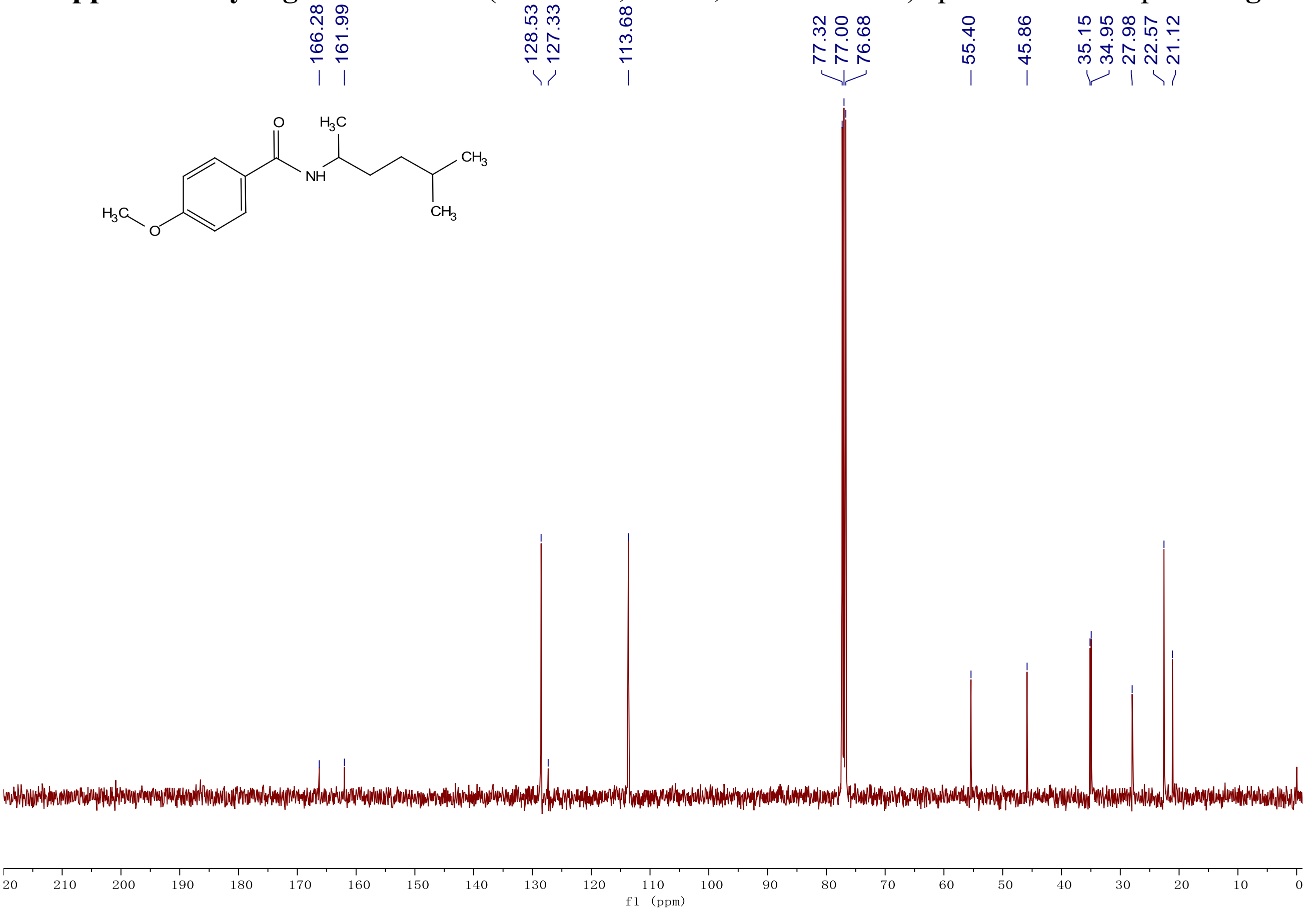

Supplementary Fig. 13. ^{13}C NMR (101 MHz, 298 K, Chloroform-*d*) spectrum of compound **1g**.

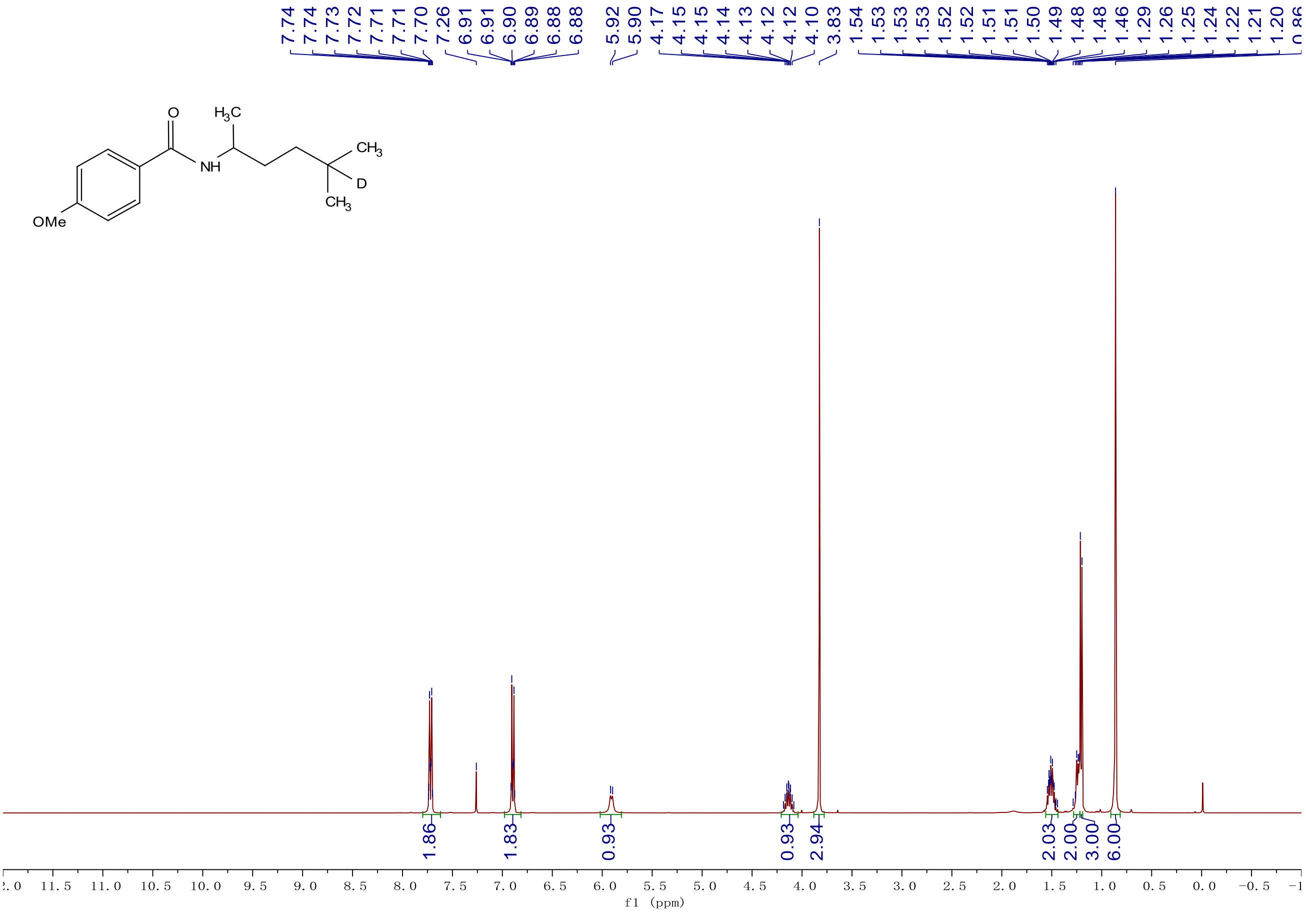

Supplementary Fig. 14. ^{1}H NMR (400 MHz, 298 K, Chloroform-*d*) spectrum of compound **3g**.

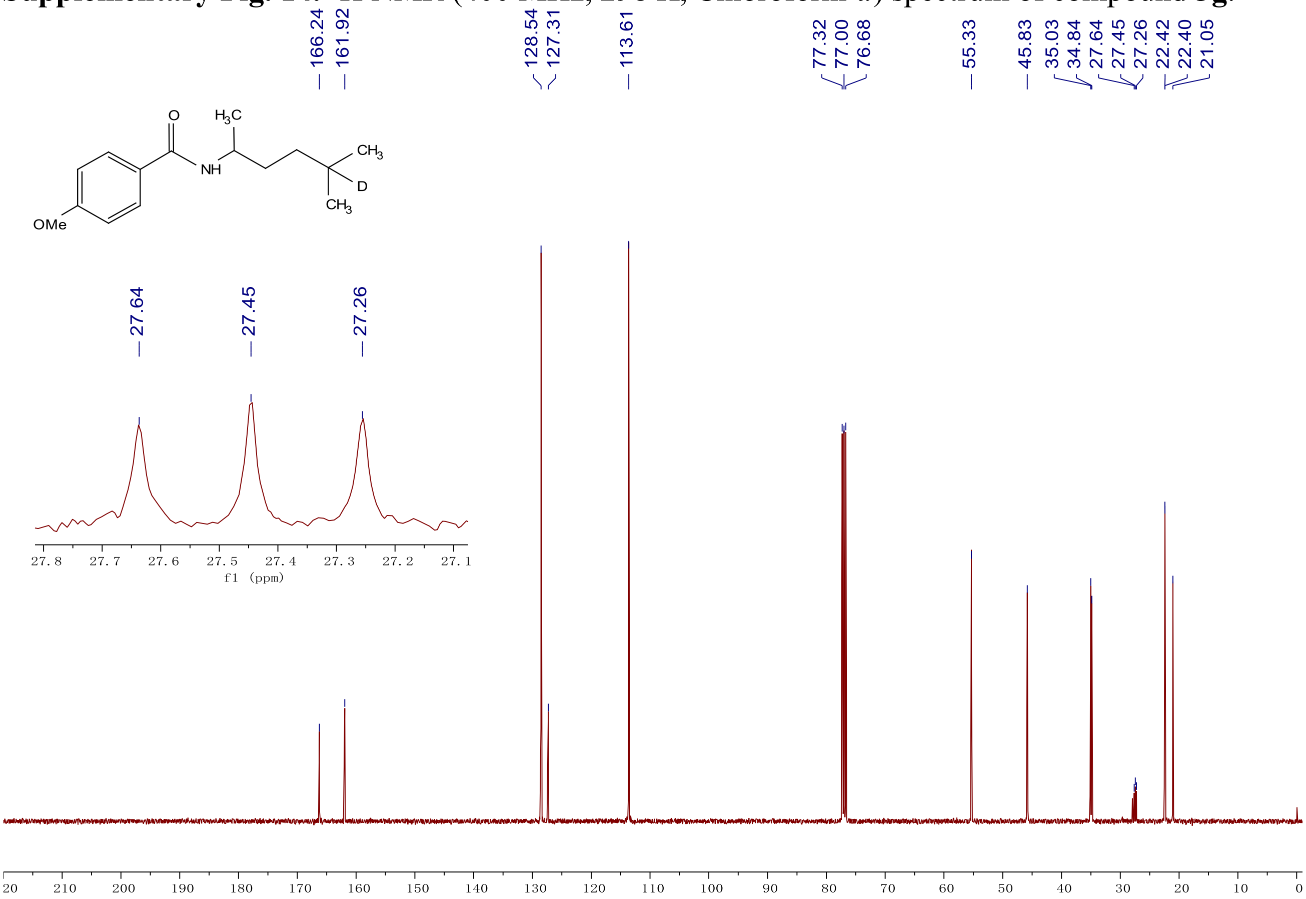

Supplementary Fig. 15. ^{13}C NMR (101 MHz, 298 K, Chloroform-*d*) spectrum of compound **3g**.

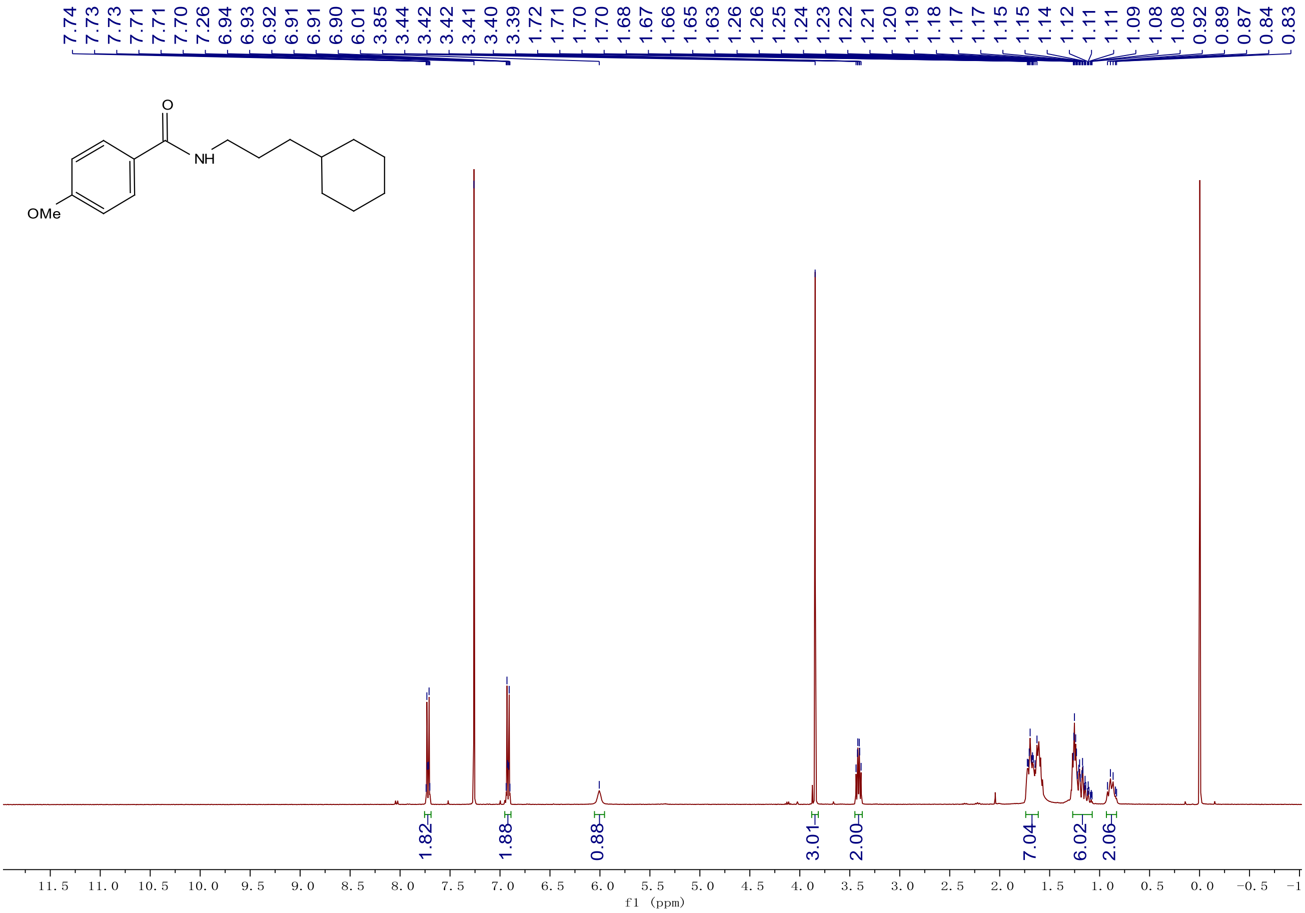

Supplementary Fig. 16. ^{1}H NMR (400 MHz, 298 K, Chloroform-*d*) spectrum of compound **1h**.

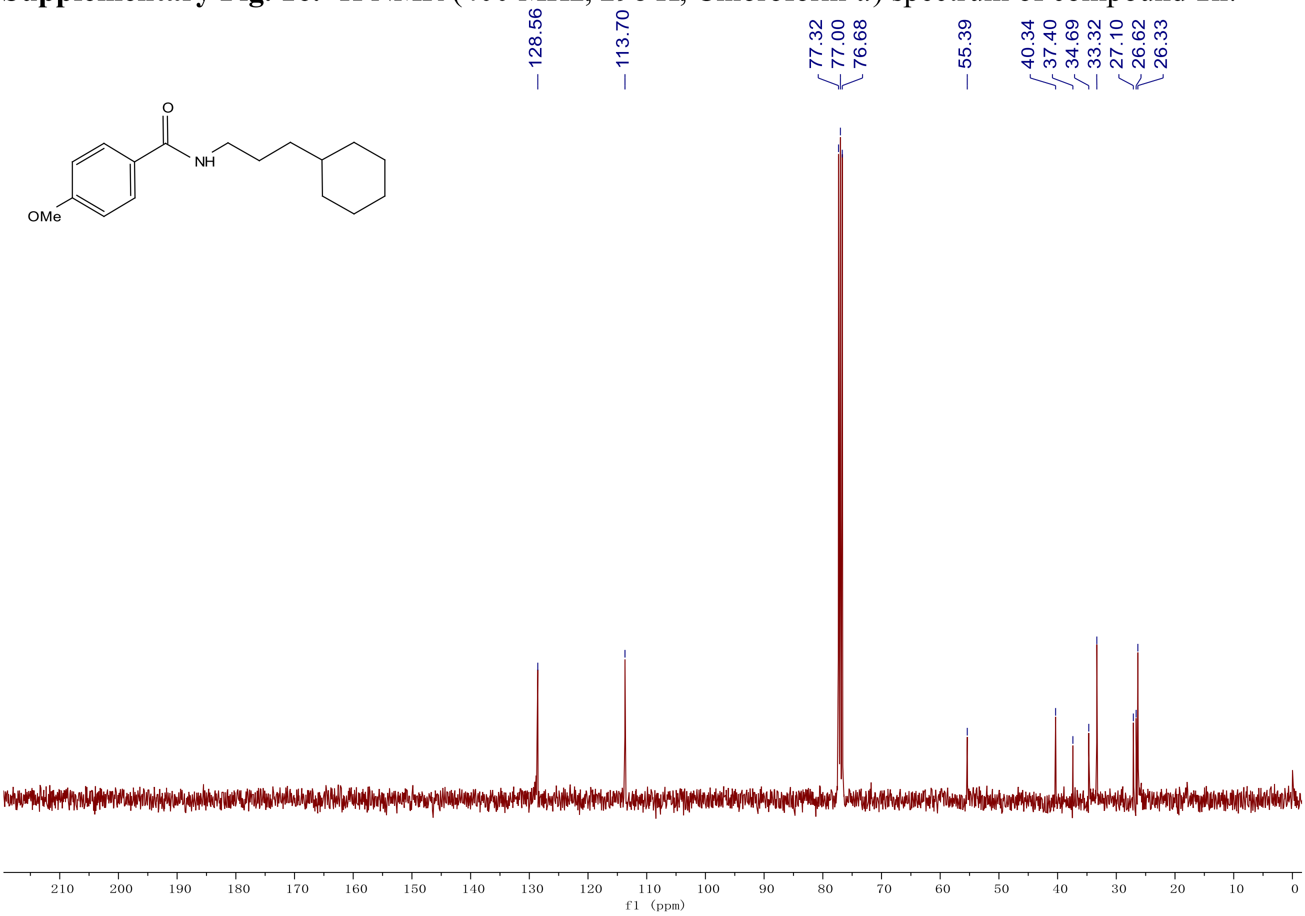

Supplementary Fig. 17. ^{13}C NMR (101 MHz, 298 K, Chloroform-*d*) spectrum of compound **1h**.

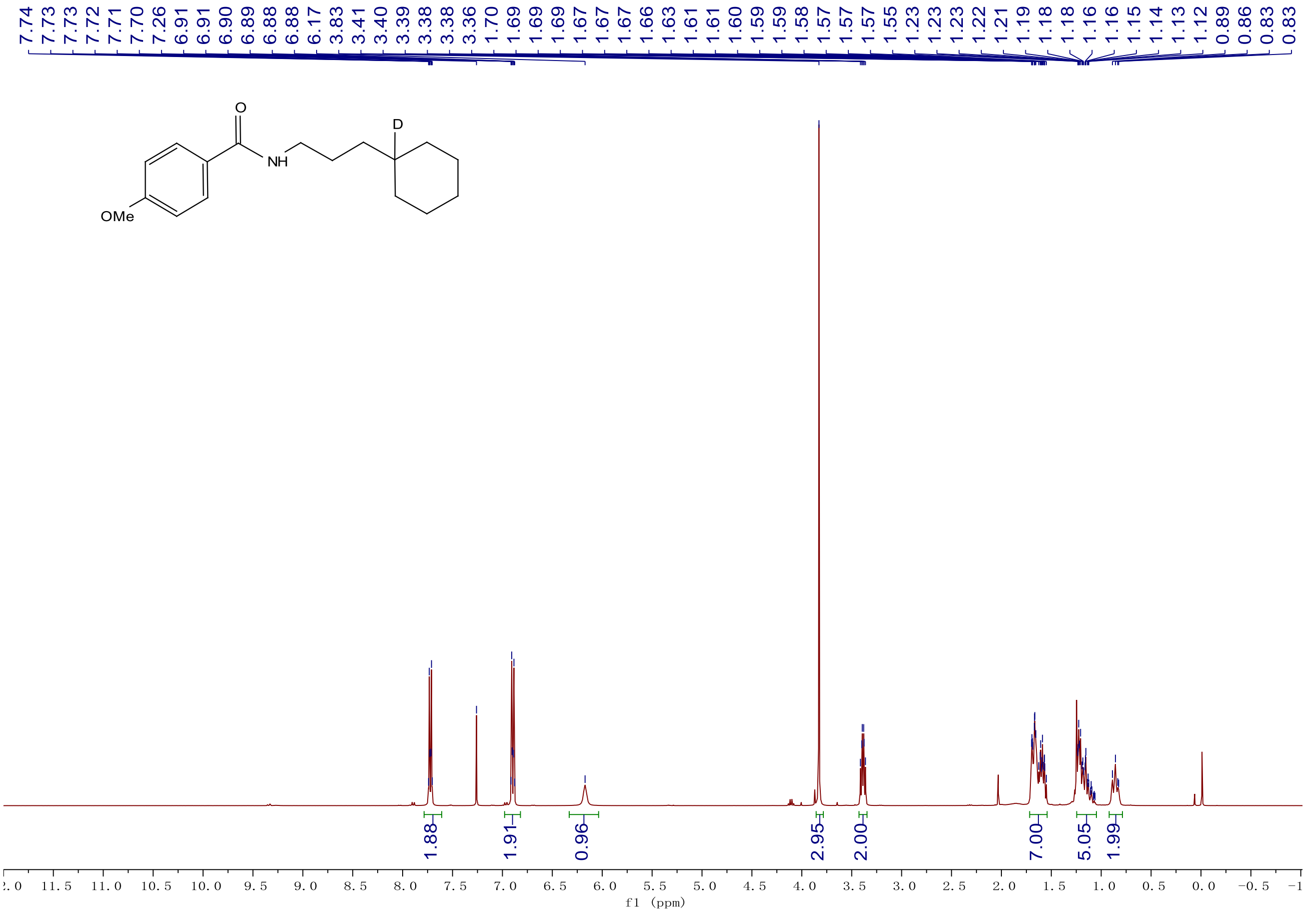

Supplementary Fig. 18. ^{1}H NMR (400 MHz, 298 K, Chloroform-*d*) spectrum of compound **3h**.

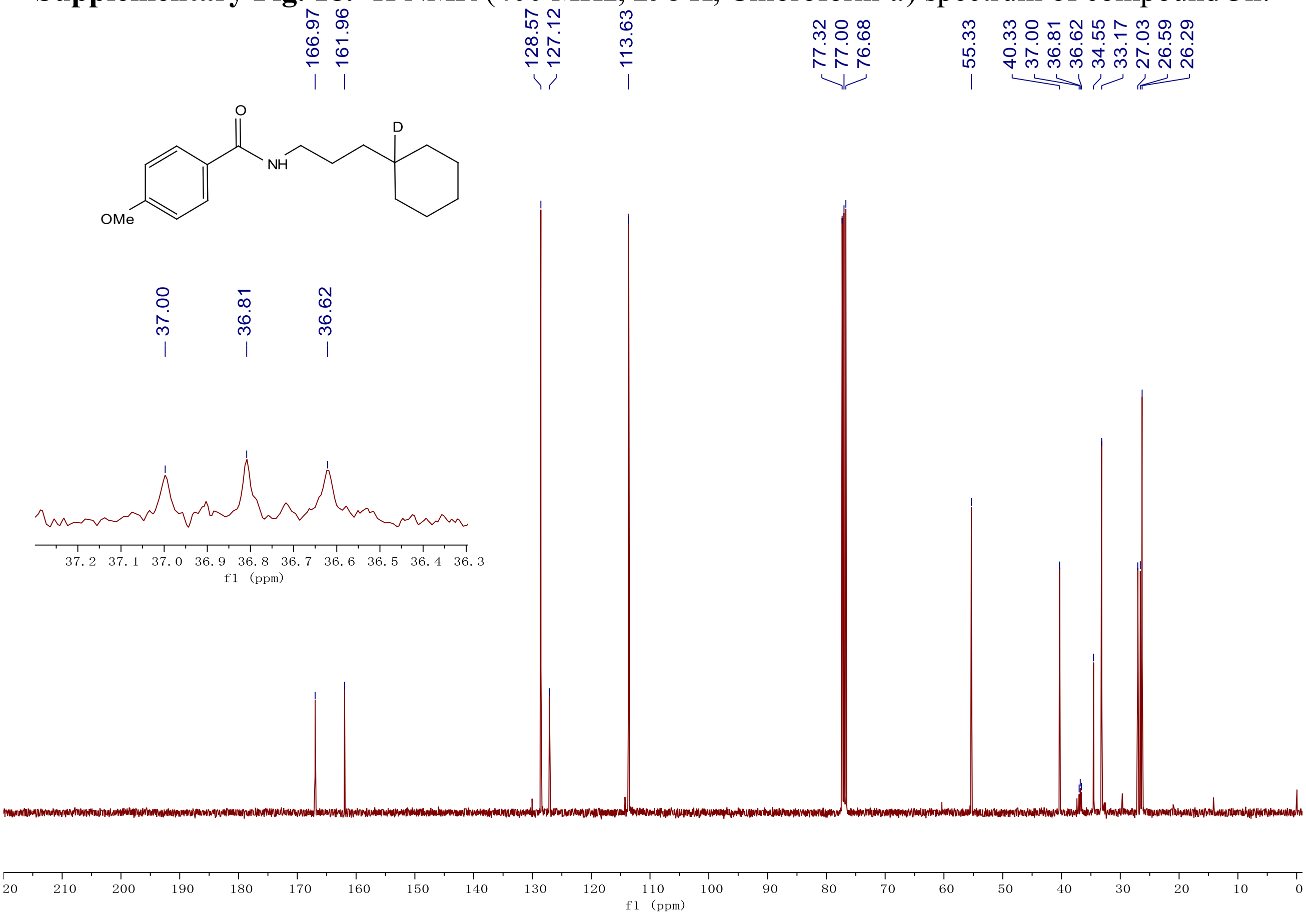

Supplementary Fig. 19. ^{13}C (101 MHz, 298 K, Chloroform-*d*) NMR spectrum of compound **3h**.

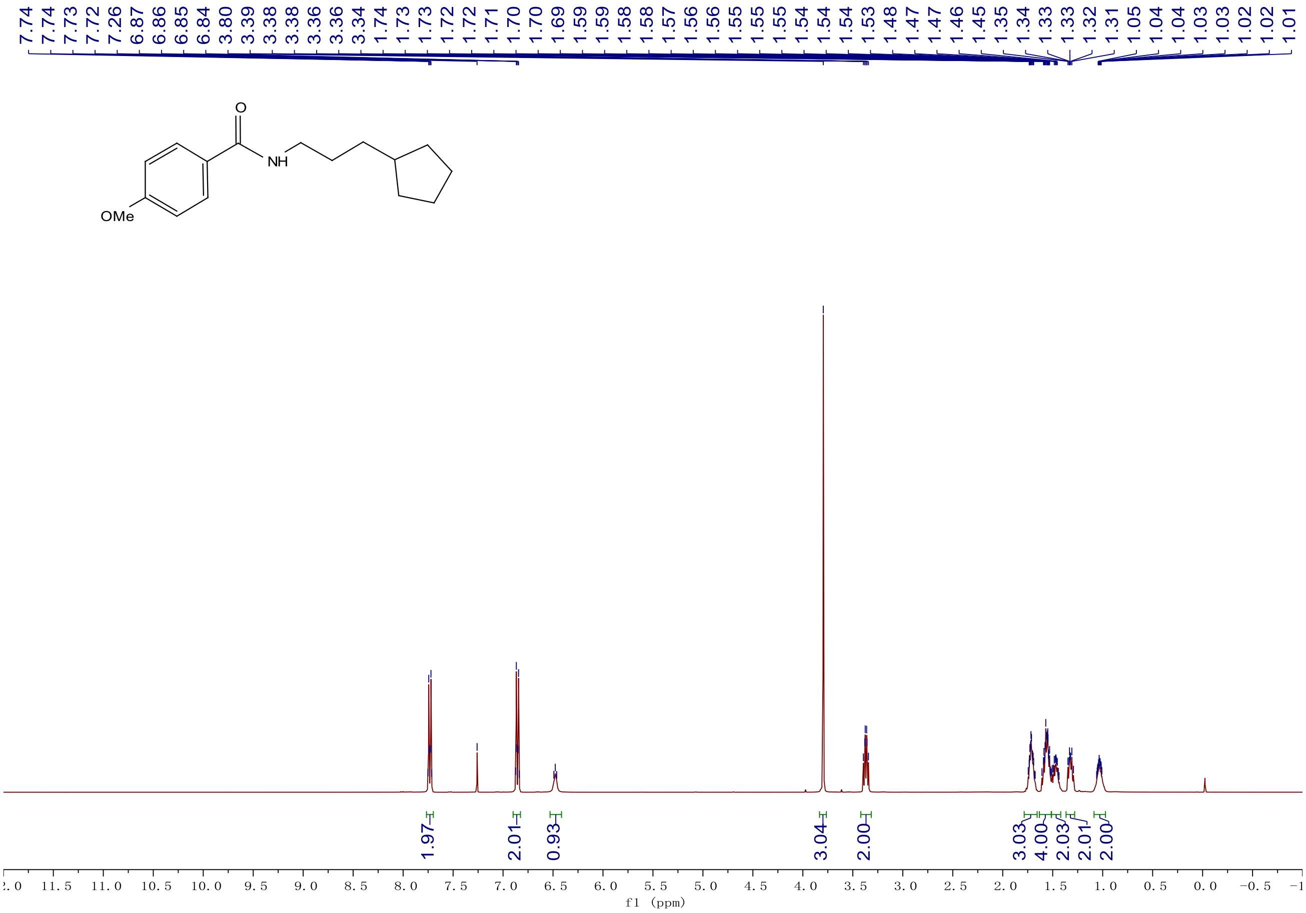

Supplementary Fig. 20. ^{1}H NMR (400 MHz, 298 K, Chloroform-*d*) spectrum of compound **1i**.

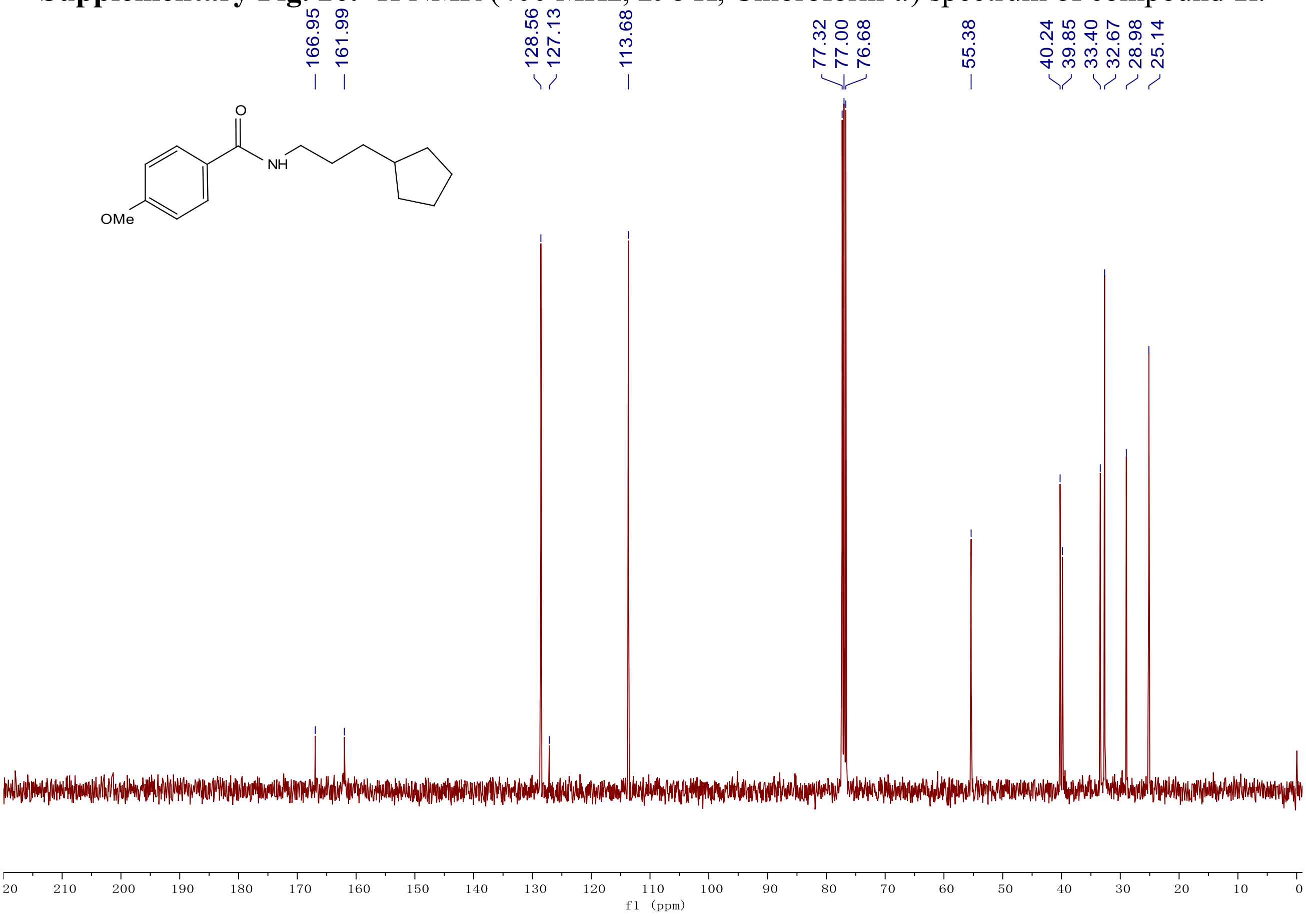

Supplementary Fig. 21. ^{13}C NMR (101 MHz, 298 K, Chloroform-*d*) spectrum of compound **1i**.

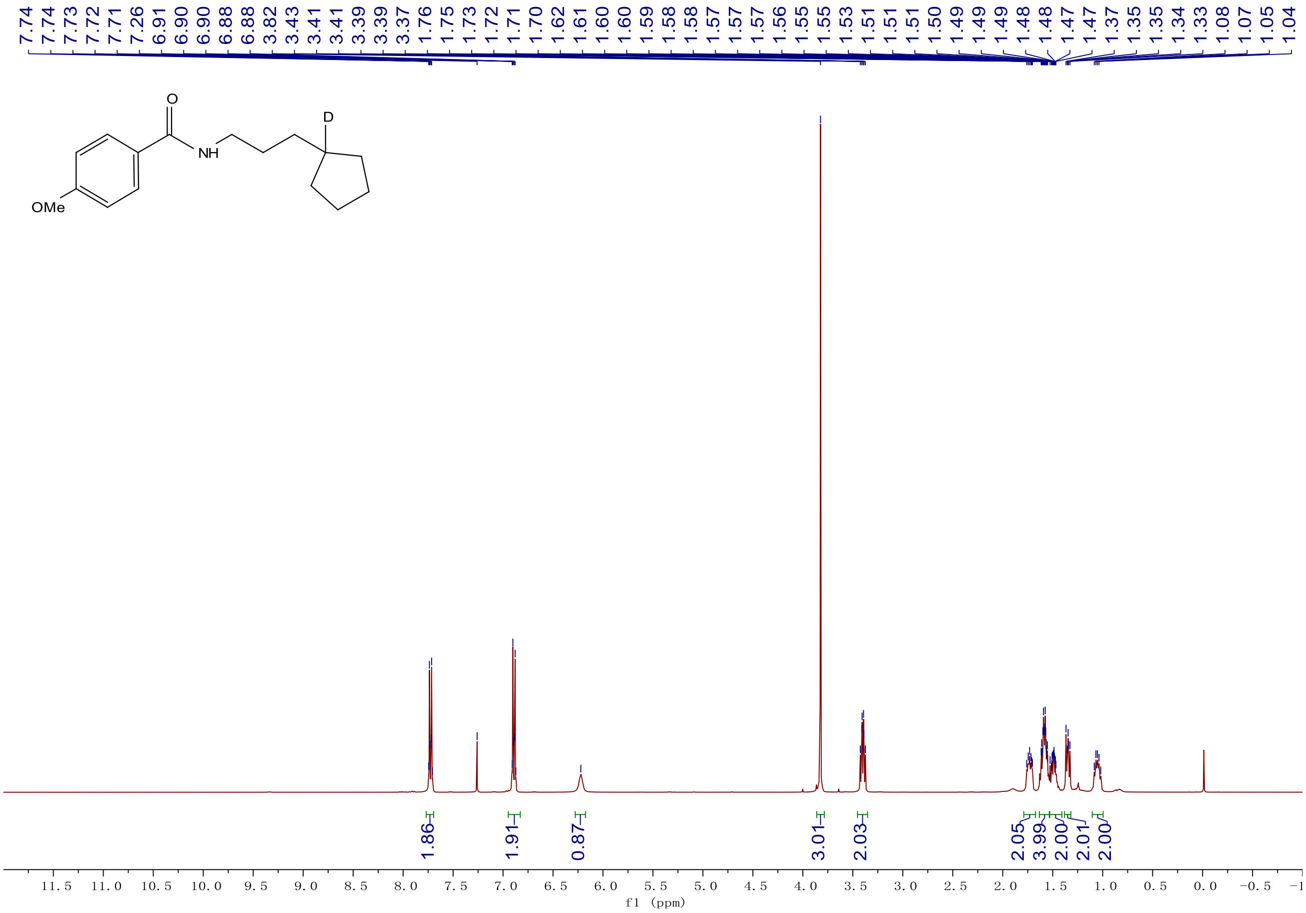

Supplementary Fig. 22. ^{1}H NMR (400 MHz, 298 K, Chloroform-*d*) spectrum of compound **3i**.

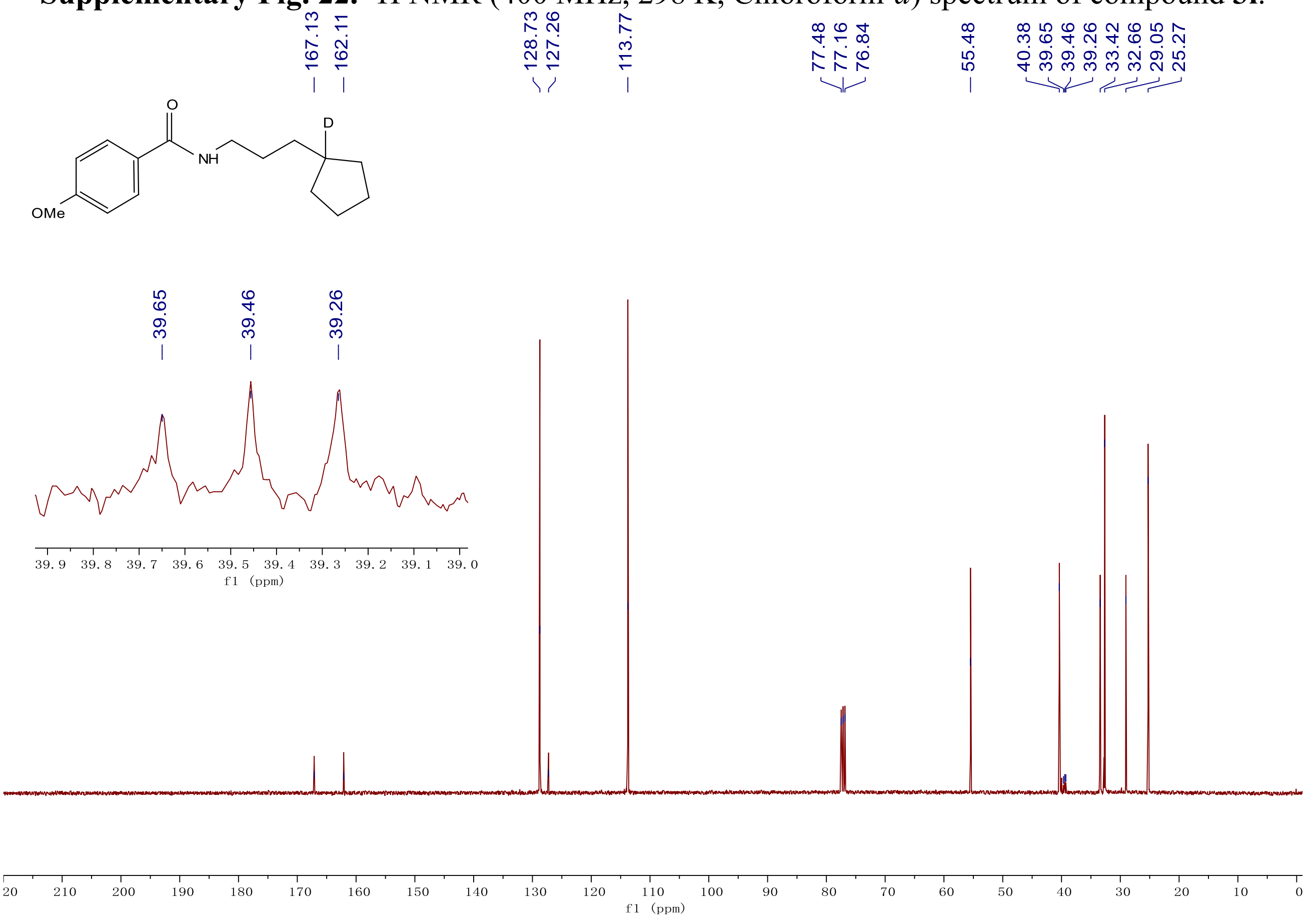

Supplementary Fig. 23. ^{13}C NMR (101 MHz, 298 K, Chloroform-*d*) spectrum of compound **3i**.

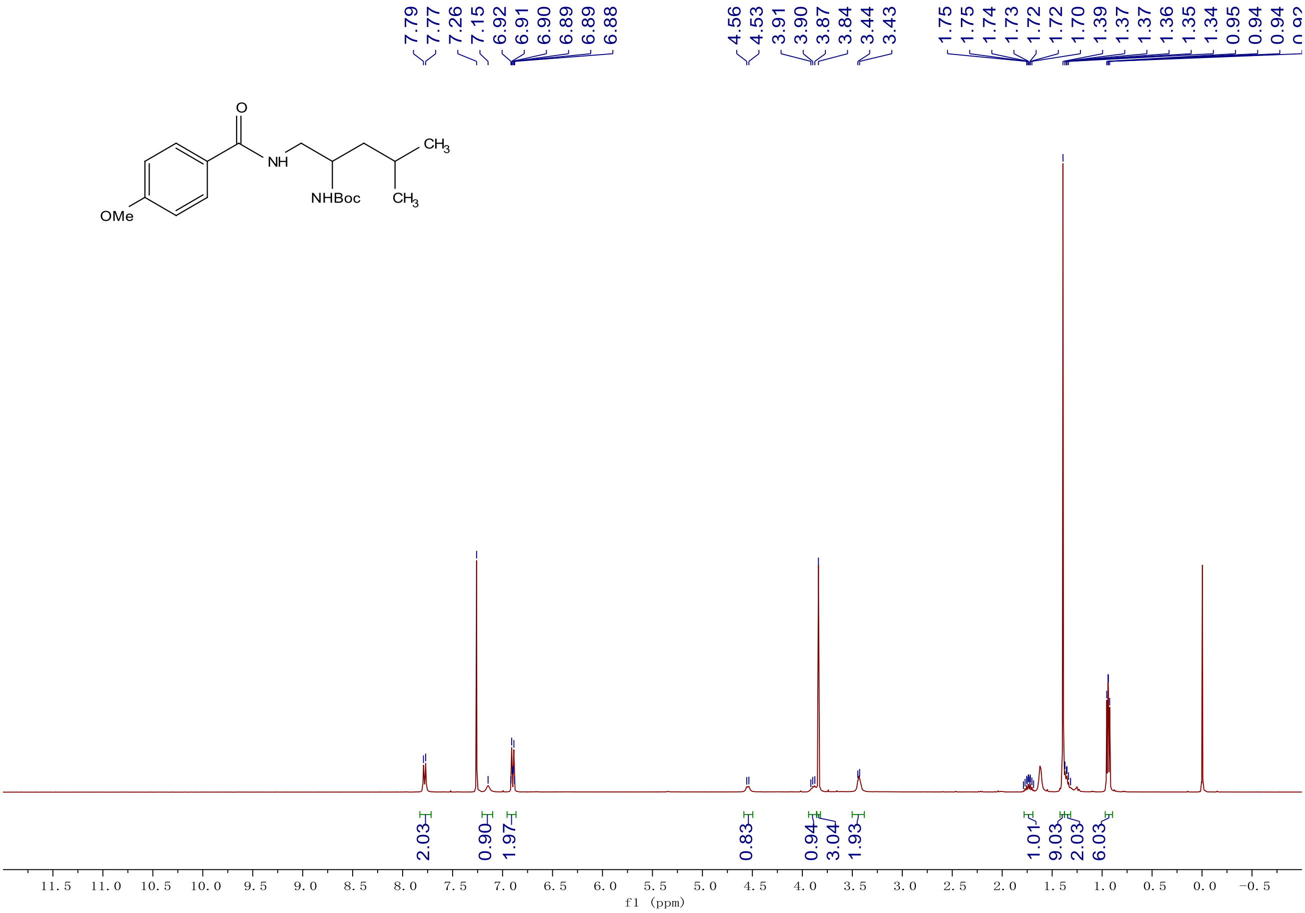

Supplementary Fig. 24. ^{1}H NMR (400 MHz, 298 K, Chloroform-*d*) spectrum of compound **1j**.

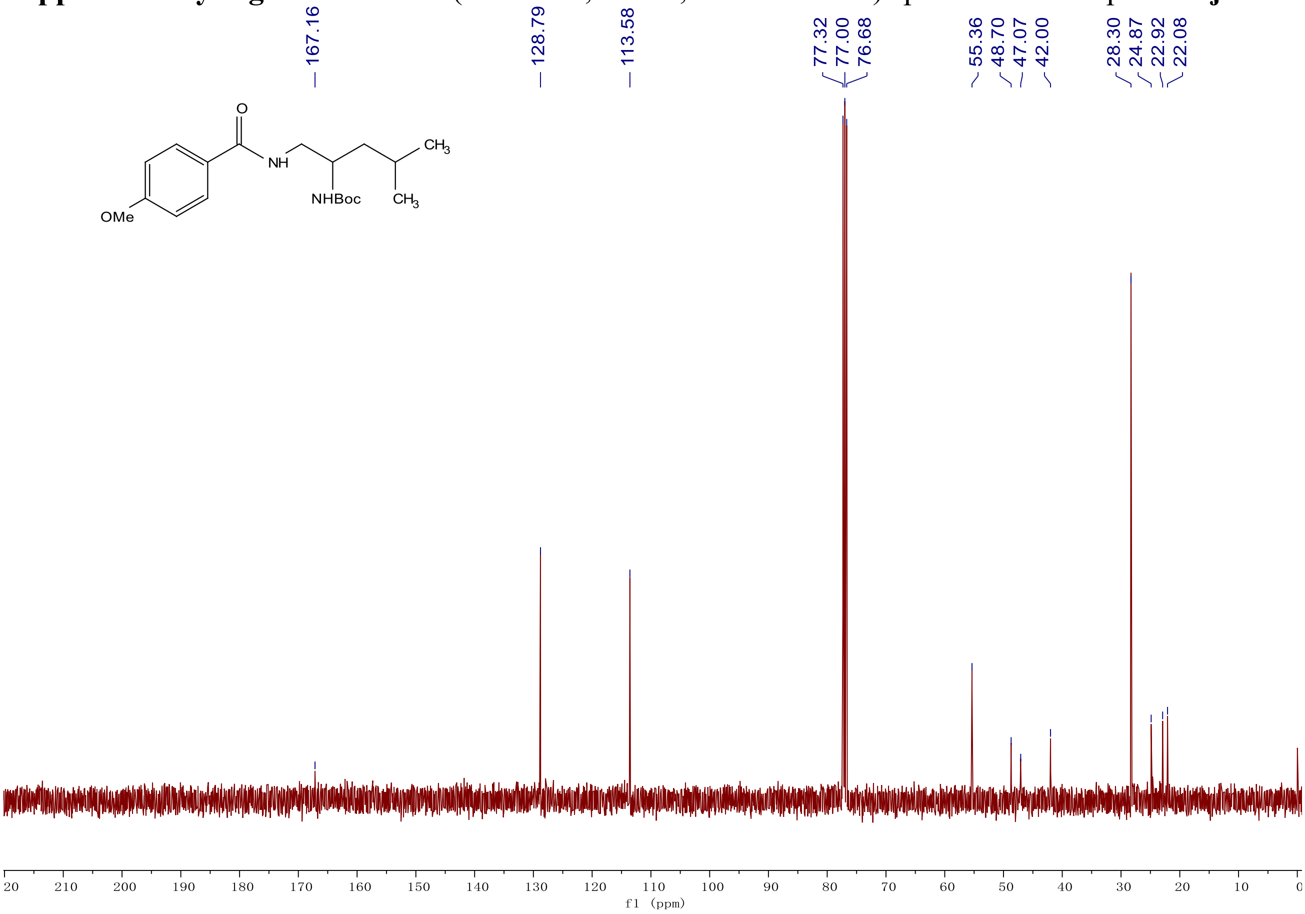

Supplementary Fig. 25. ^{13}C NMR (101 MHz, 298 K, Chloroform-*d*) spectrum of compound **1j**.

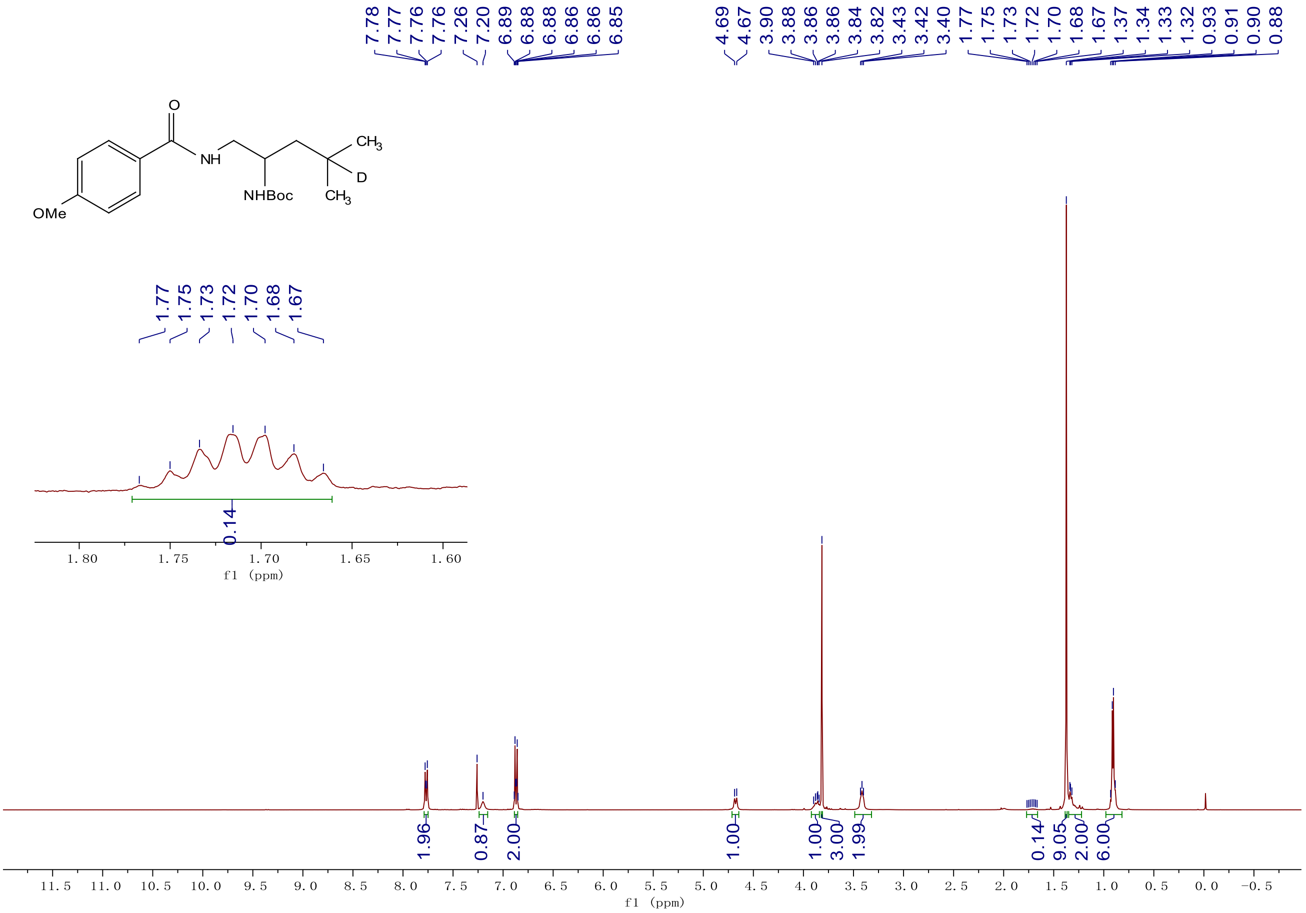

Supplementary Fig. 26. ^{1}H NMR (400 MHz, 298 K, Chloroform-*d*) spectrum of compound **3j**.

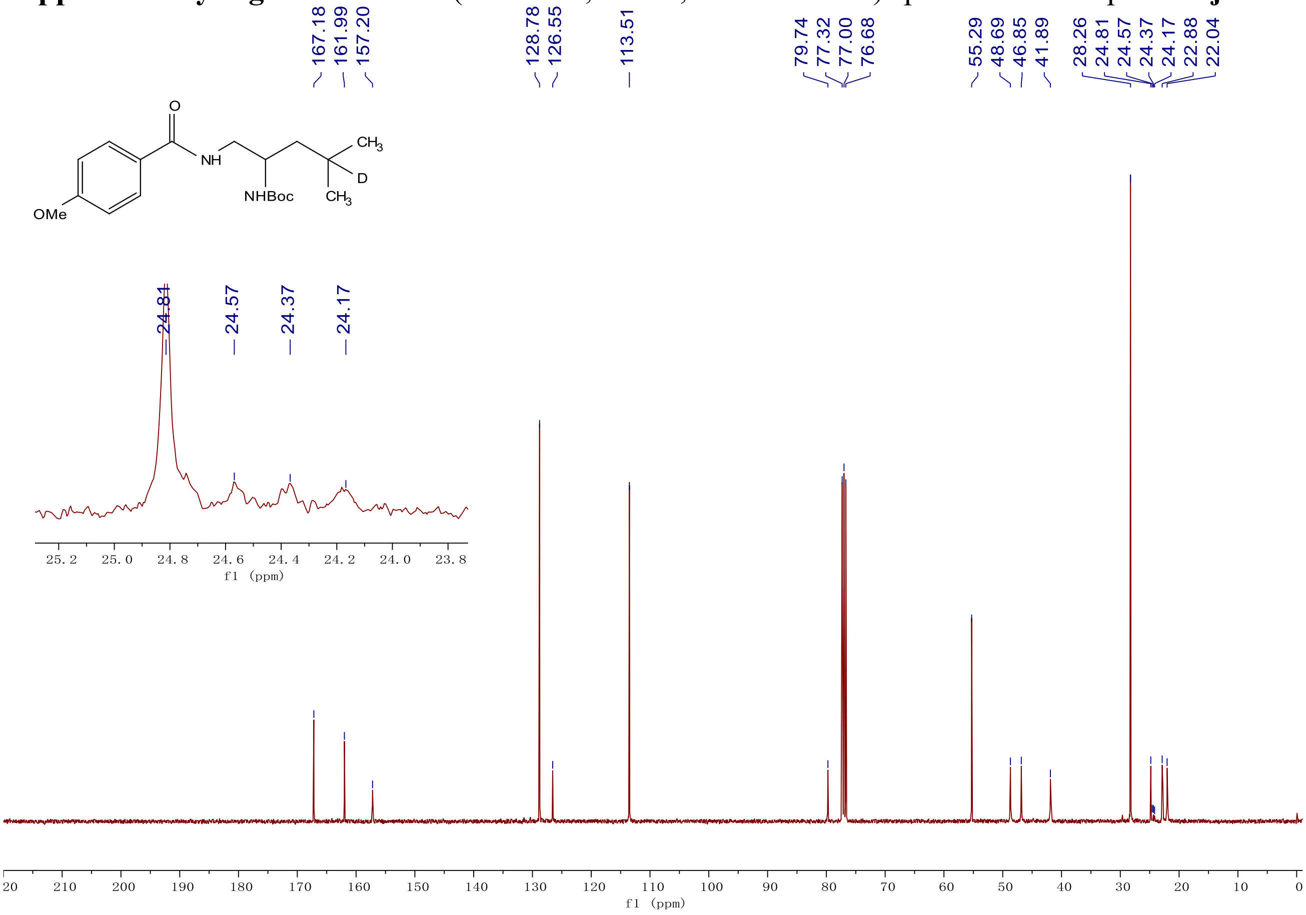

Supplementary Fig. 27. ^{13}C NMR (101 MHz, 298 K, Chloroform-*d*) spectrum of compound **3j**.

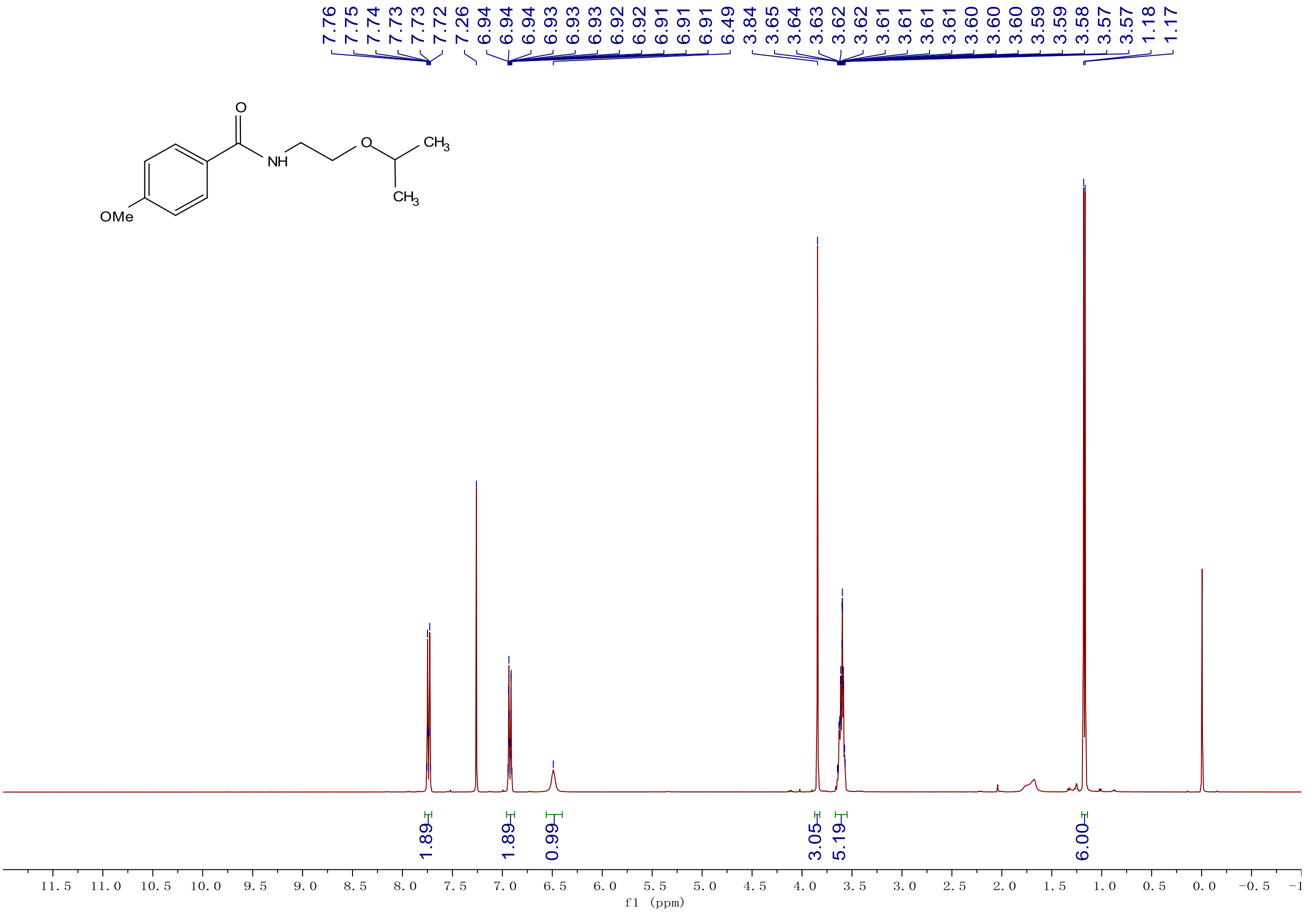

Supplementary Fig. 28. ^{1}H NMR (400 MHz, 298 K, Chloroform-*d*) spectrum of compound **1k**.

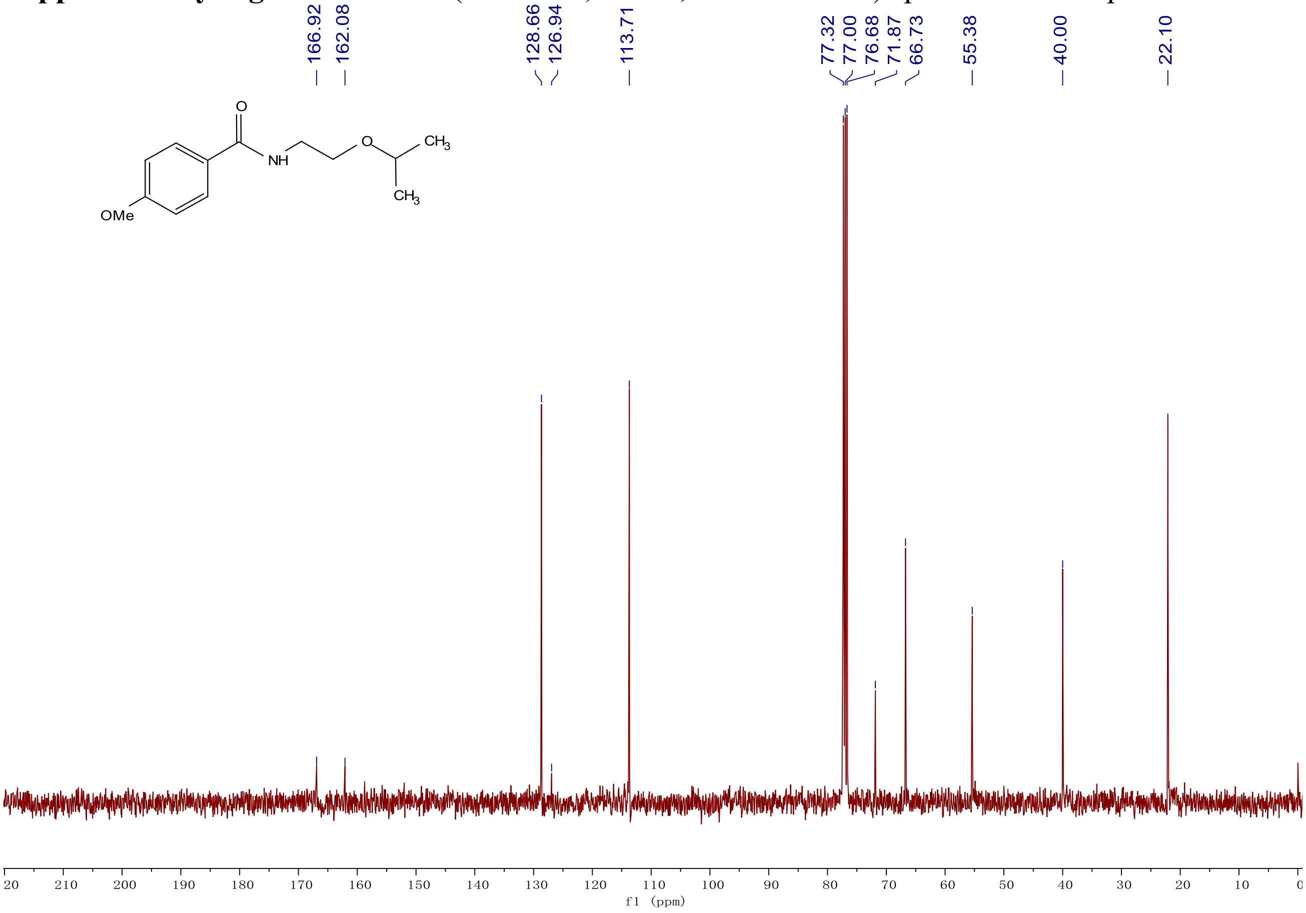

Supplementary Fig. 29. ^{13}C NMR (101 MHz, 298 K, Chloroform-*d*) spectrum of compound **1k**.

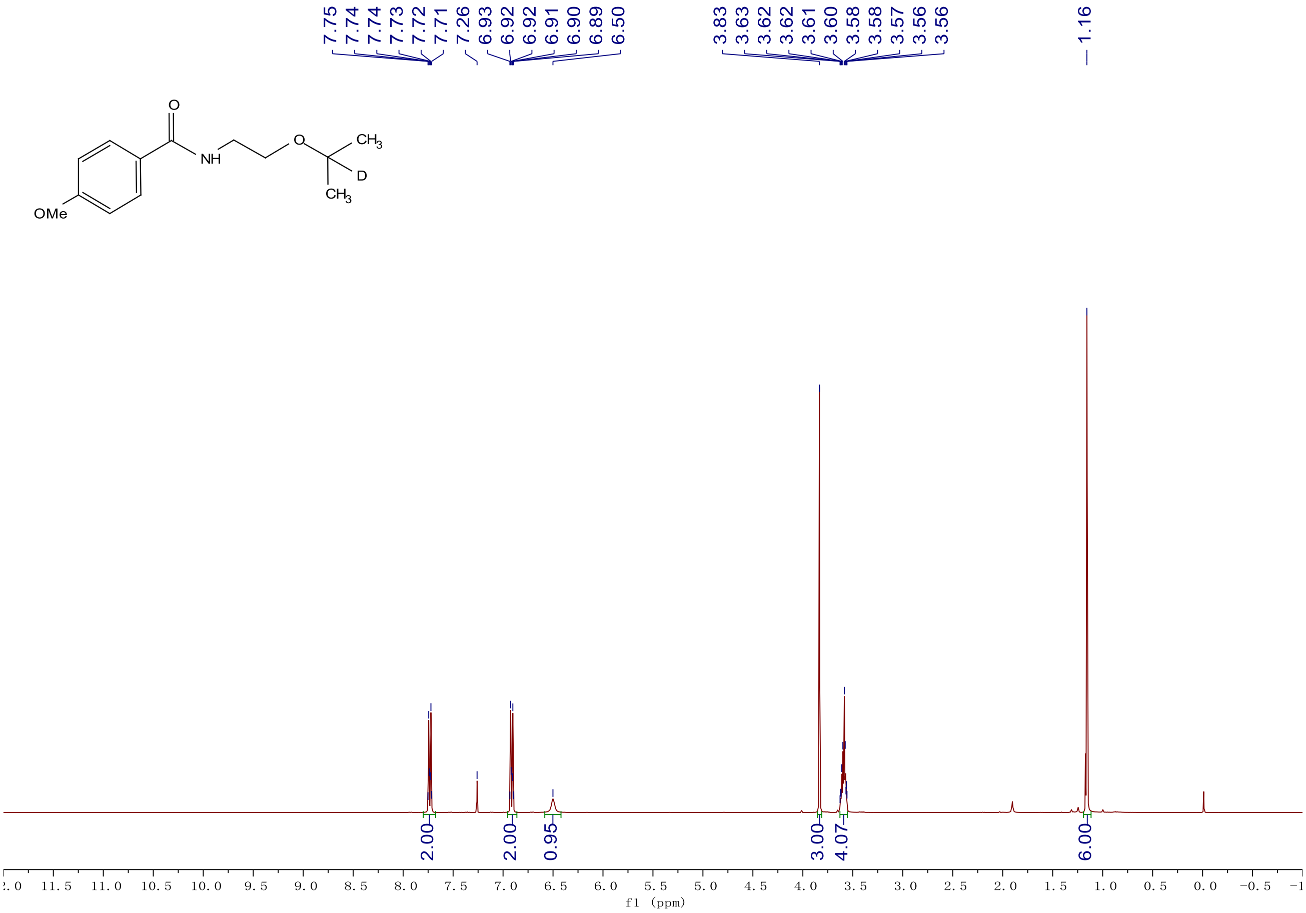

Supplementary Fig. 30. ^{1}H NMR (400 MHz, 298 K, Chloroform-*d*) spectrum of compound **3k**.

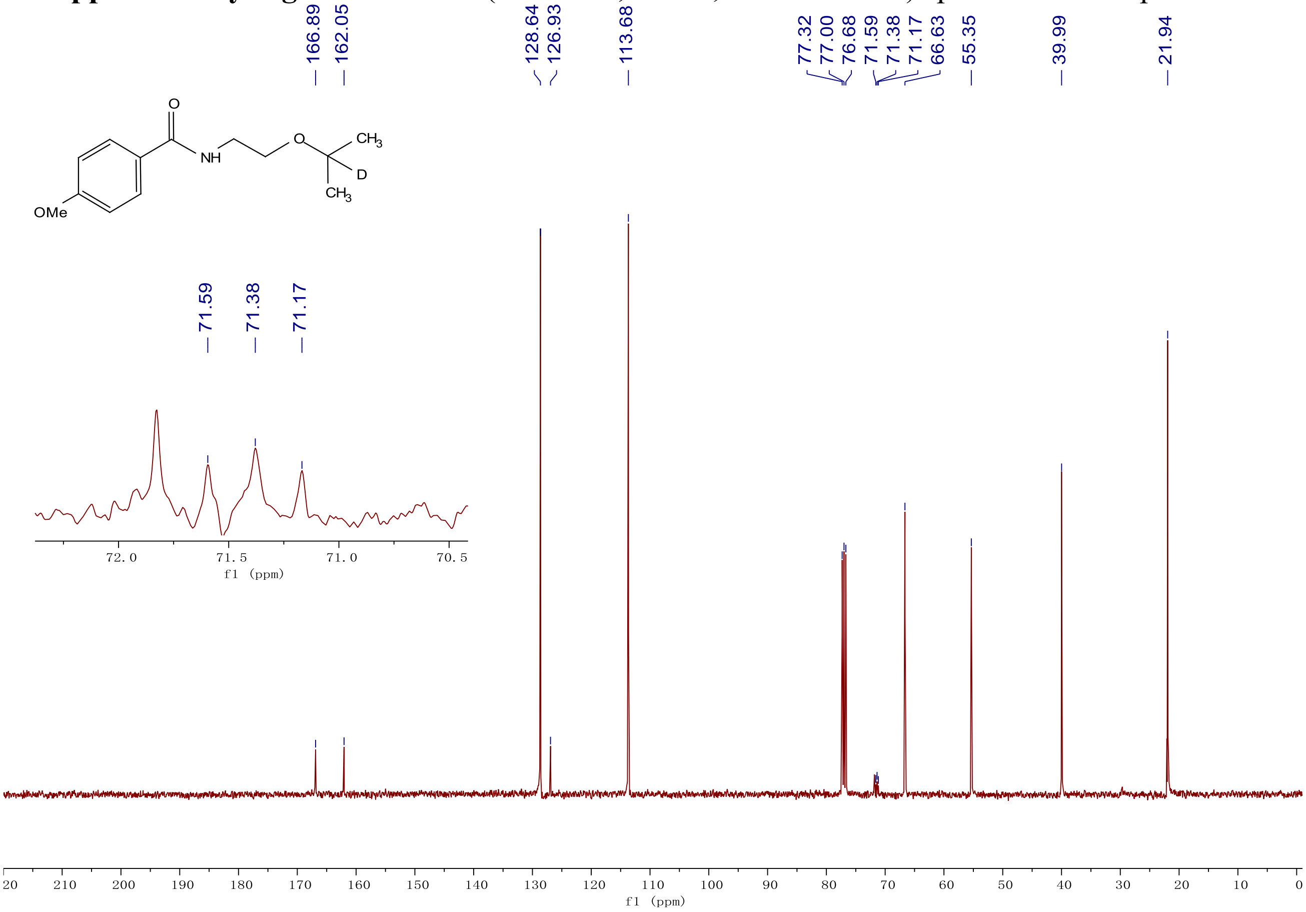

Supplementary Fig. 31. ^{13}C NMR (101 MHz, 298 K, Chloroform-*d*) spectrum of compound **3k**.

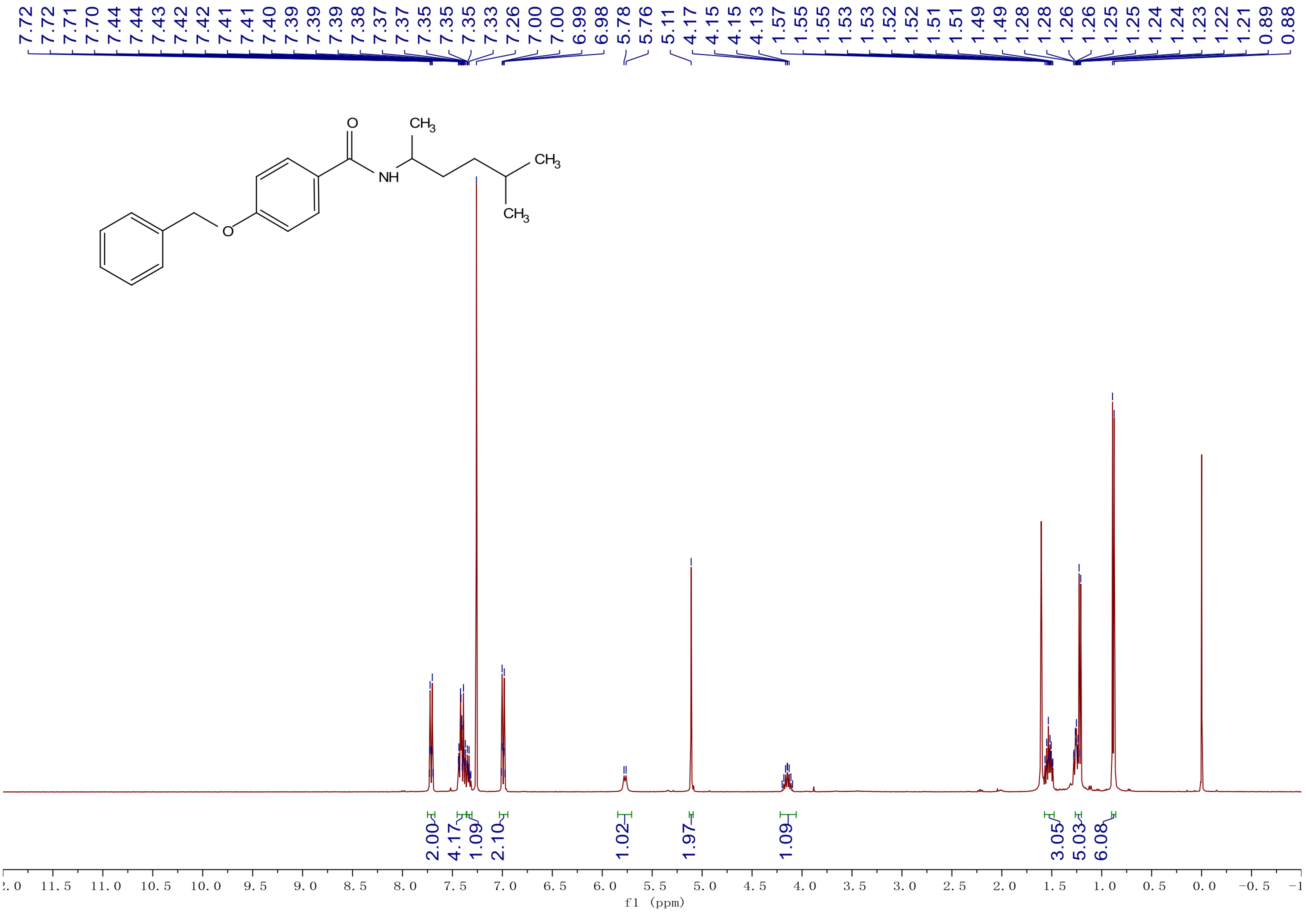

Supplementary Fig. 32. ^{1}H NMR (400 MHz, 298 K, Chloroform-*d*) spectrum of compound **1l**.

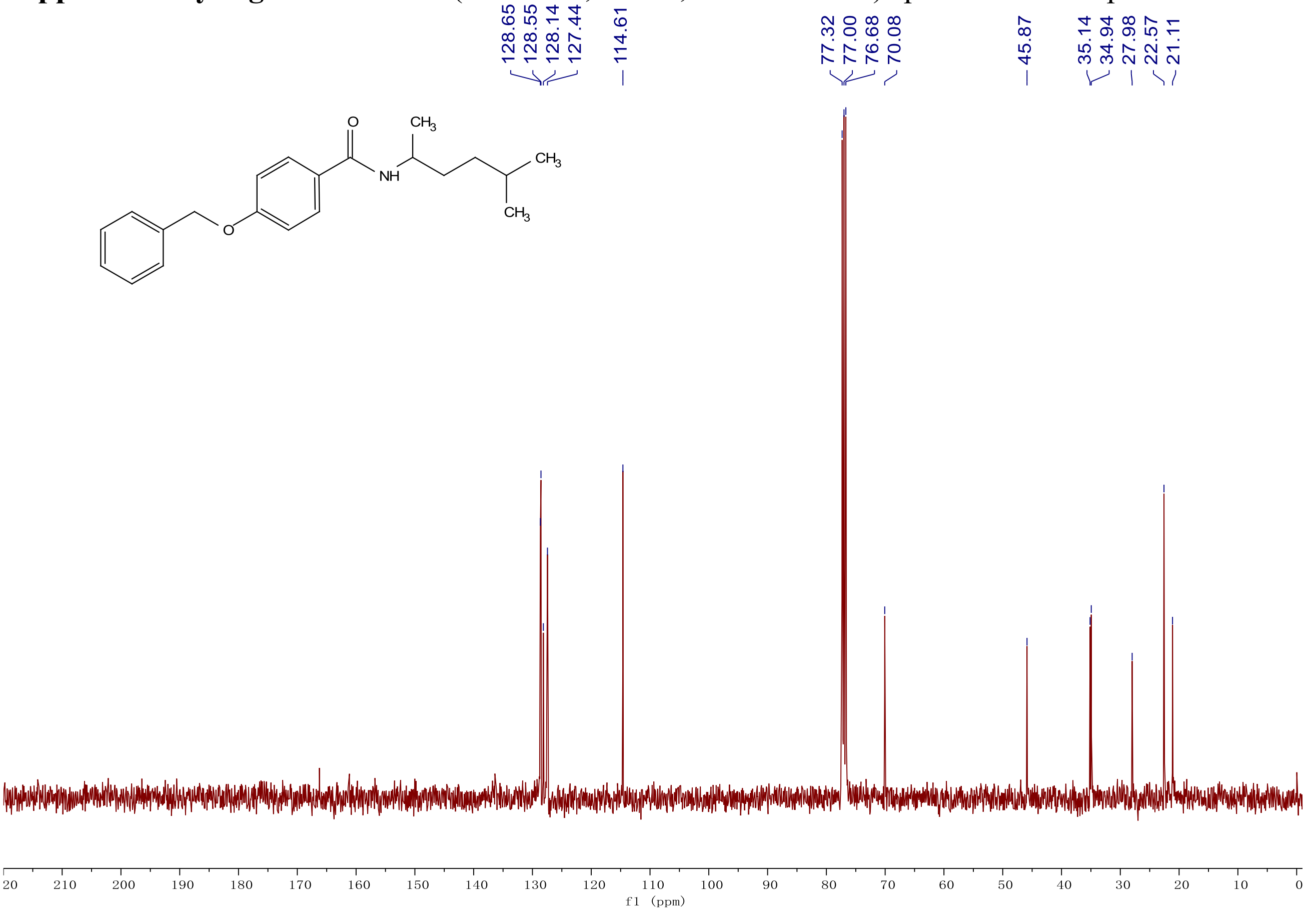

Supplementary Fig. 33. ^{13}C NMR (101 MHz, 298 K, Chloroform-*d*) spectrum of compound **1l**.

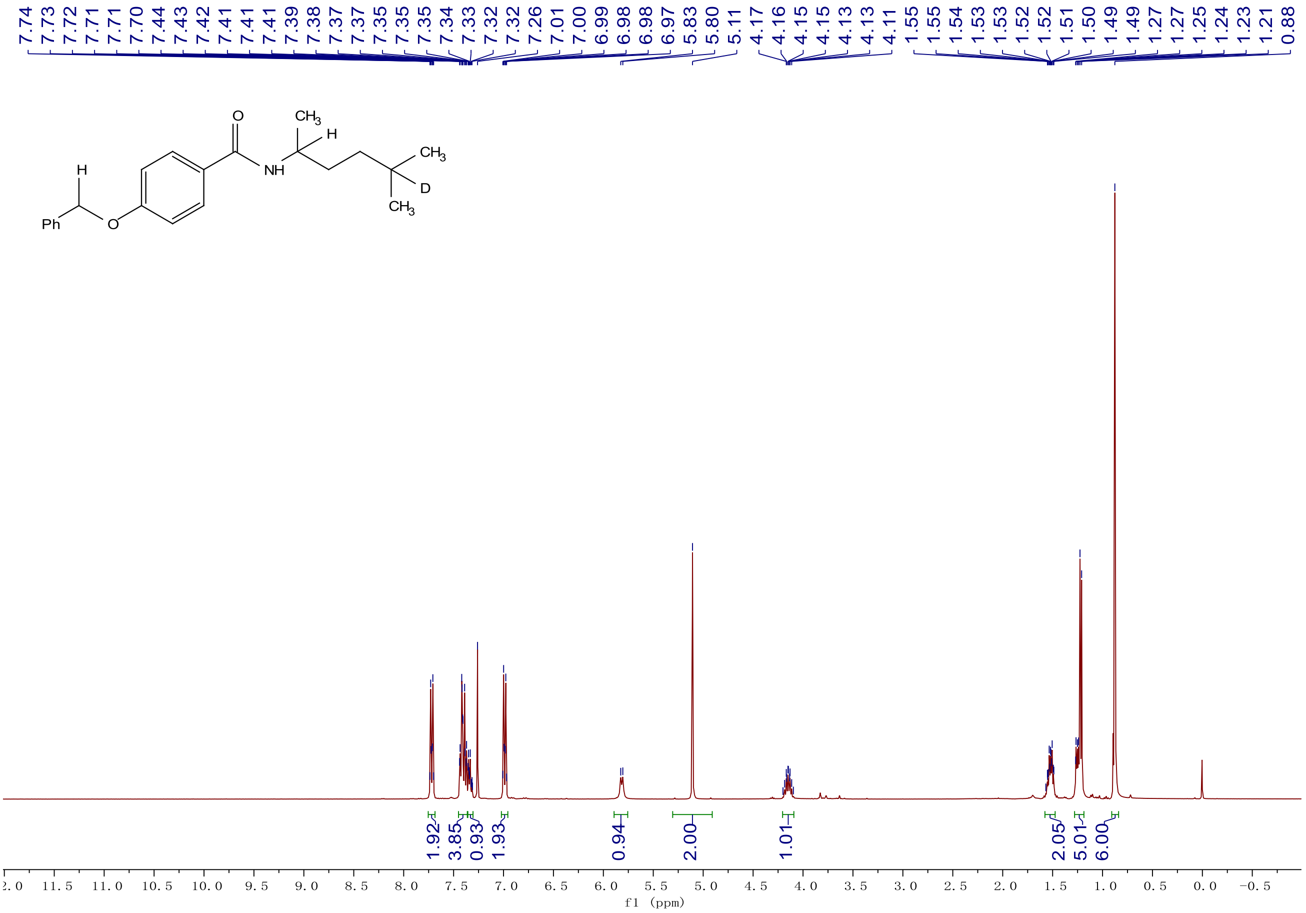

Supplementary Fig. 34. ^{1}H NMR (400 MHz, 298 K, Chloroform-*d*) spectrum of compound **3l**.

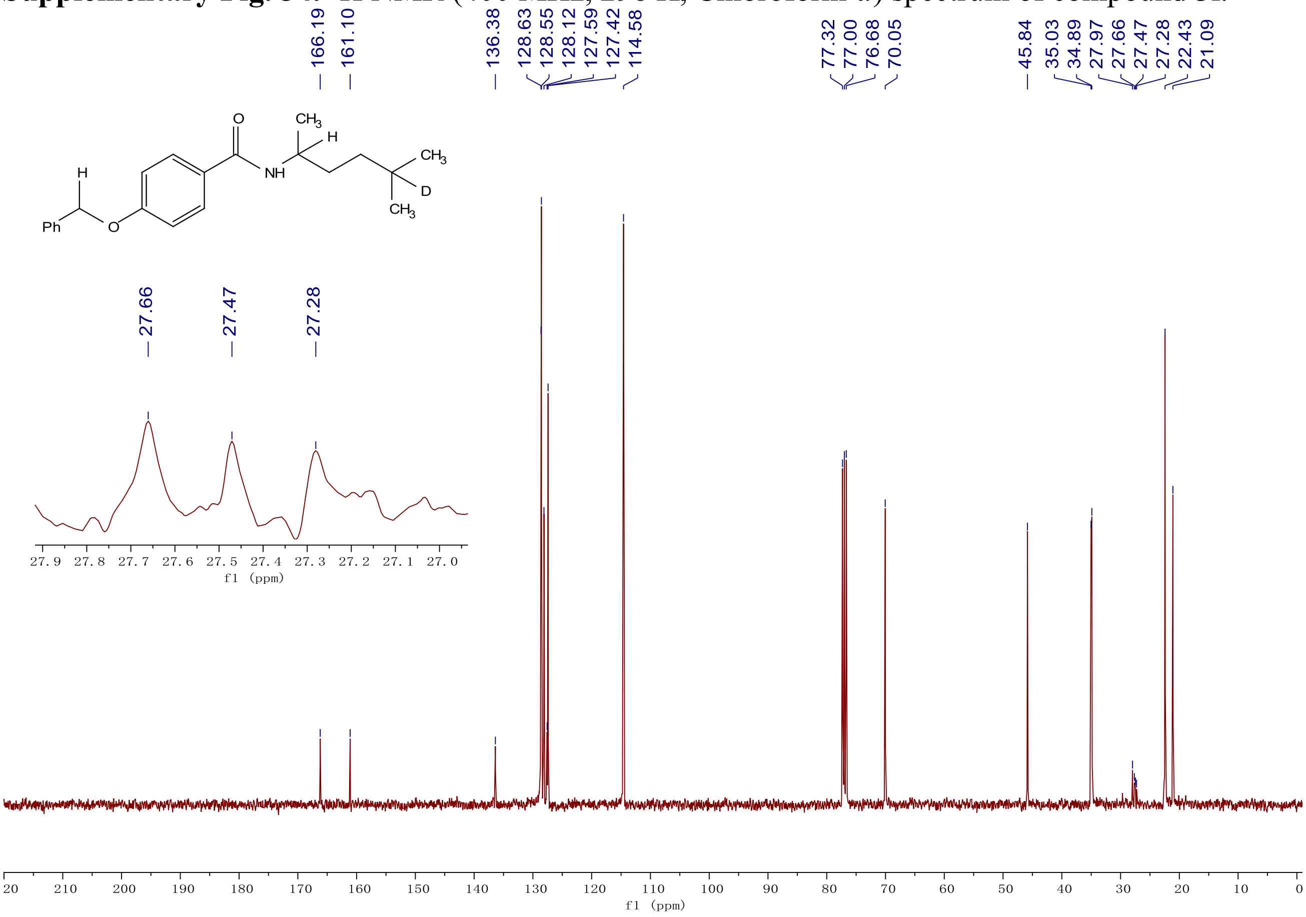

Supplementary Fig. 35. ^{13}C NMR (101 MHz, 298 K, Chloroform-*d*) spectrum of compound **3l**.

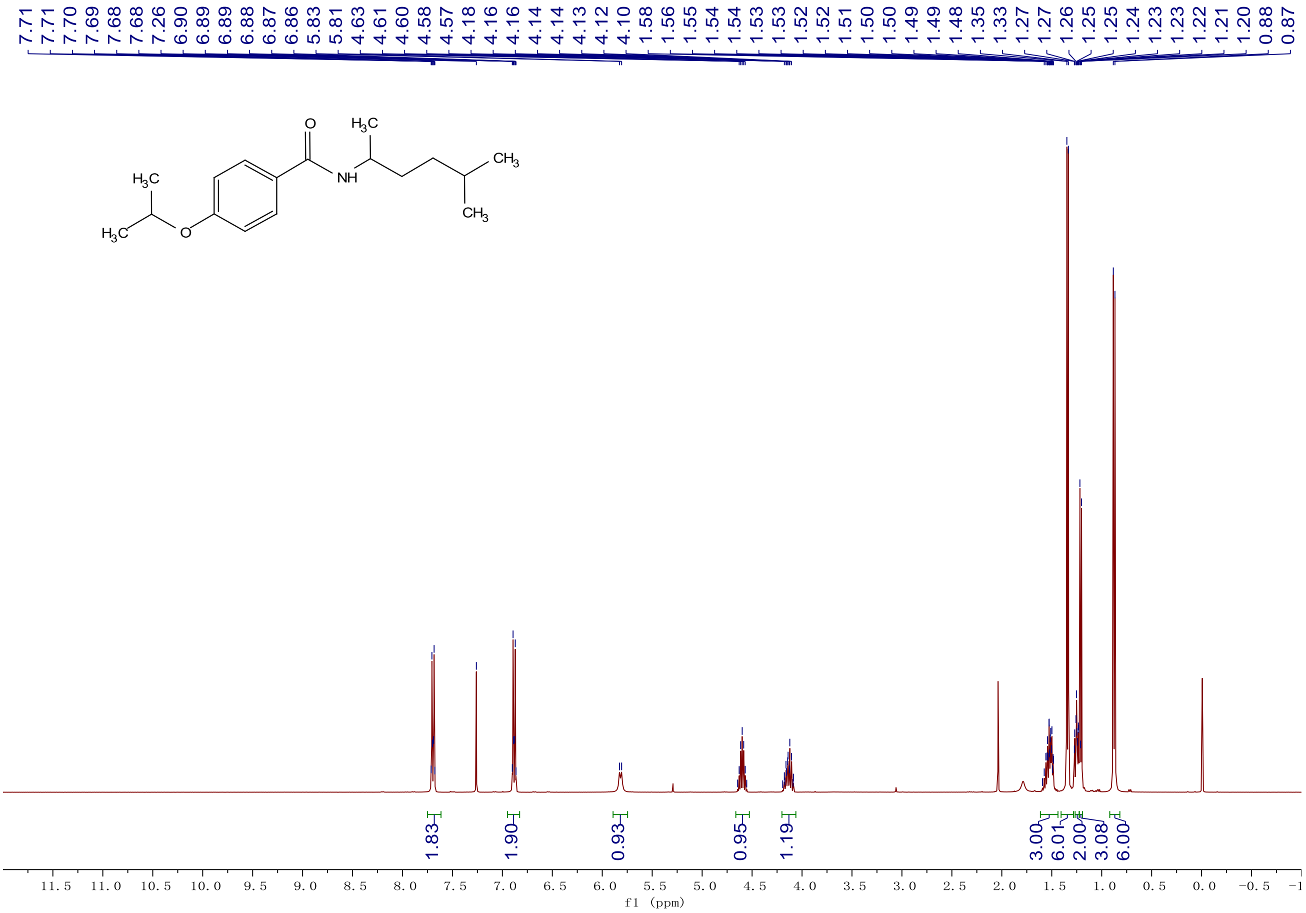

Supplementary Fig. 36. ^{1}H NMR (400 MHz, 298 K, Chloroform-*d*) spectrum of compound **1m**.

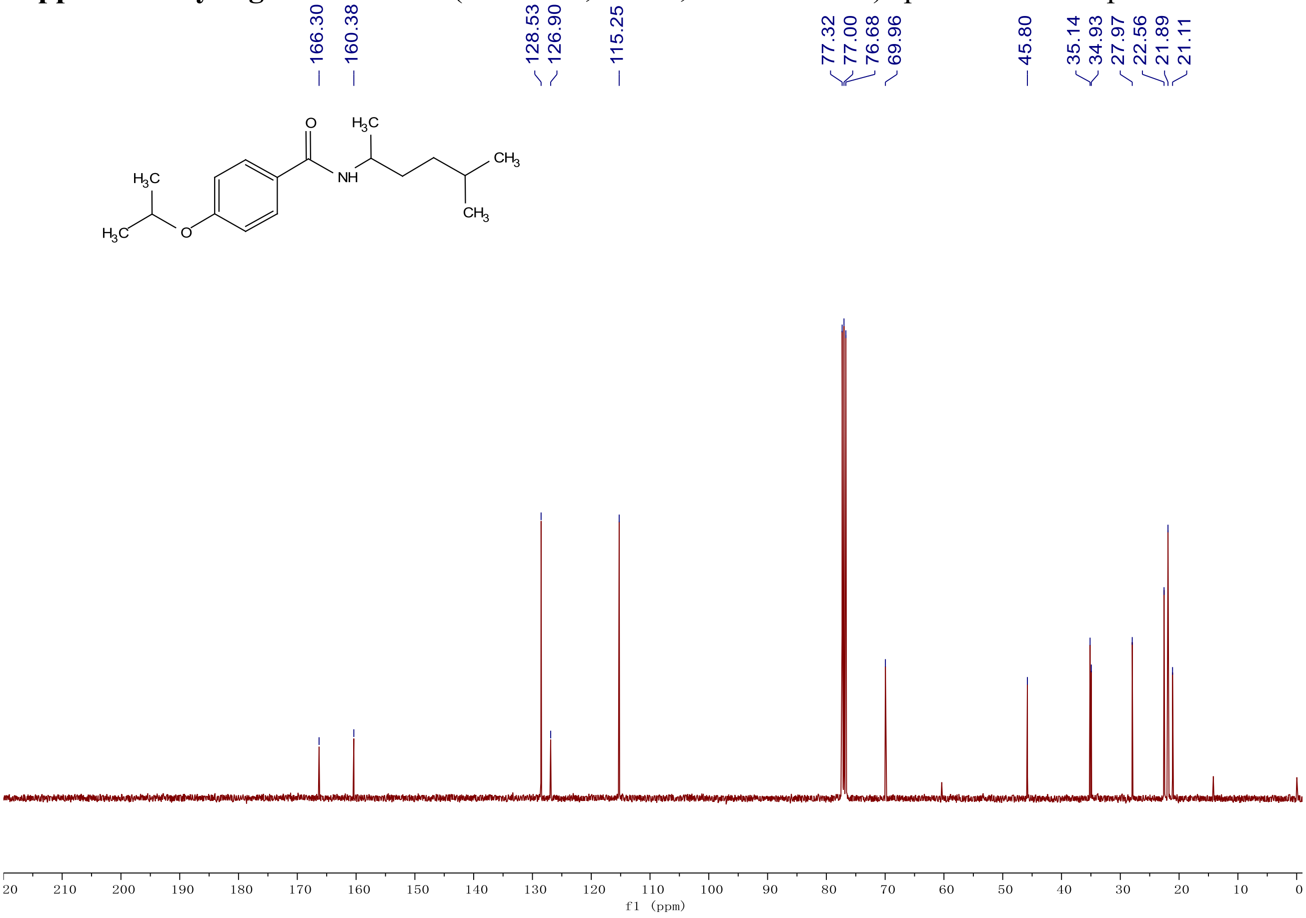

Supplementary Fig. 37. ^{13}C NMR (101 MHz, 298 K, Chloroform-*d*) spectrum of compound **1m**.

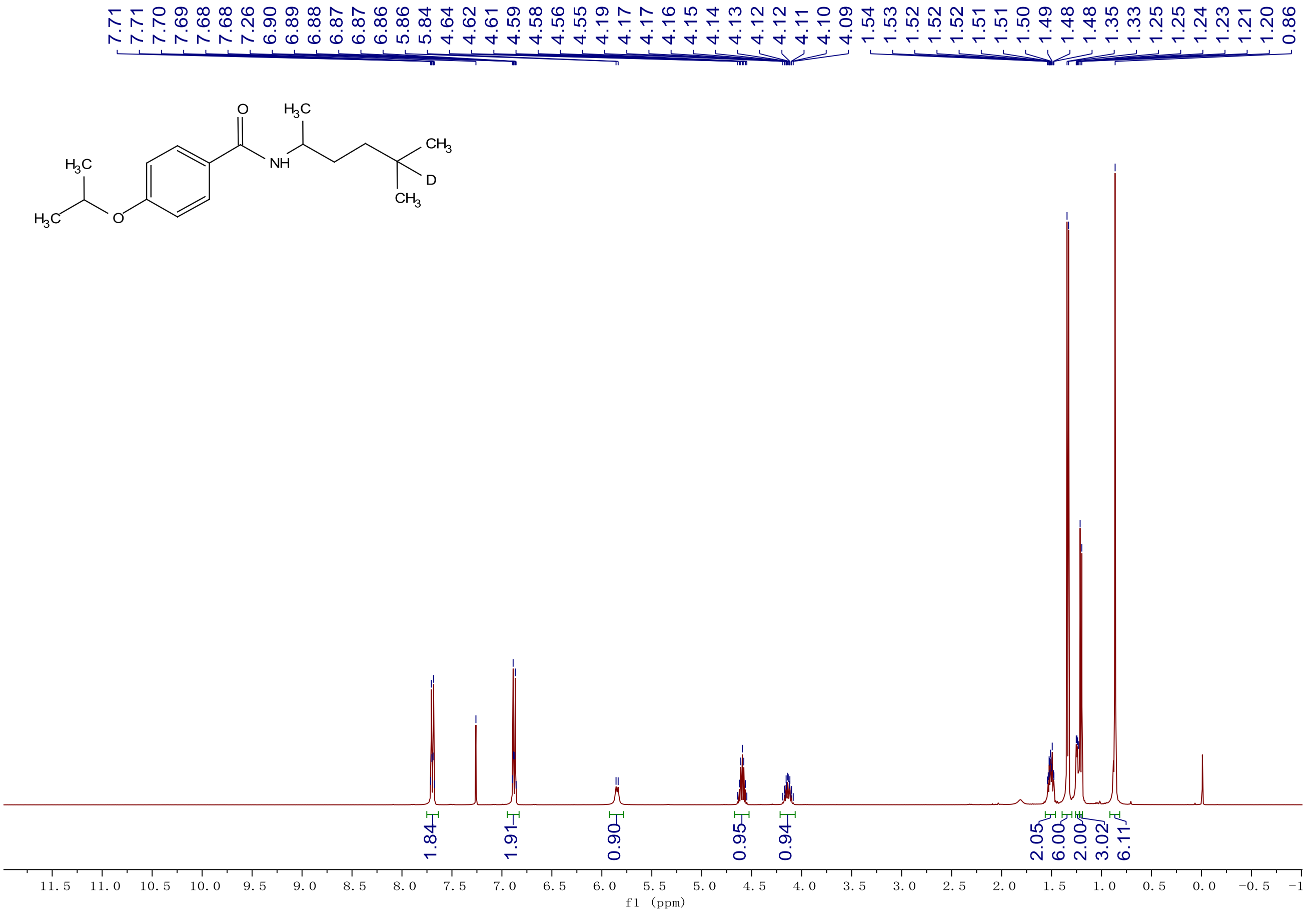

Supplementary Fig. 38. ^{1}H NMR (400 MHz, 298 K, Chloroform-*d*) spectrum of compound **3m**.

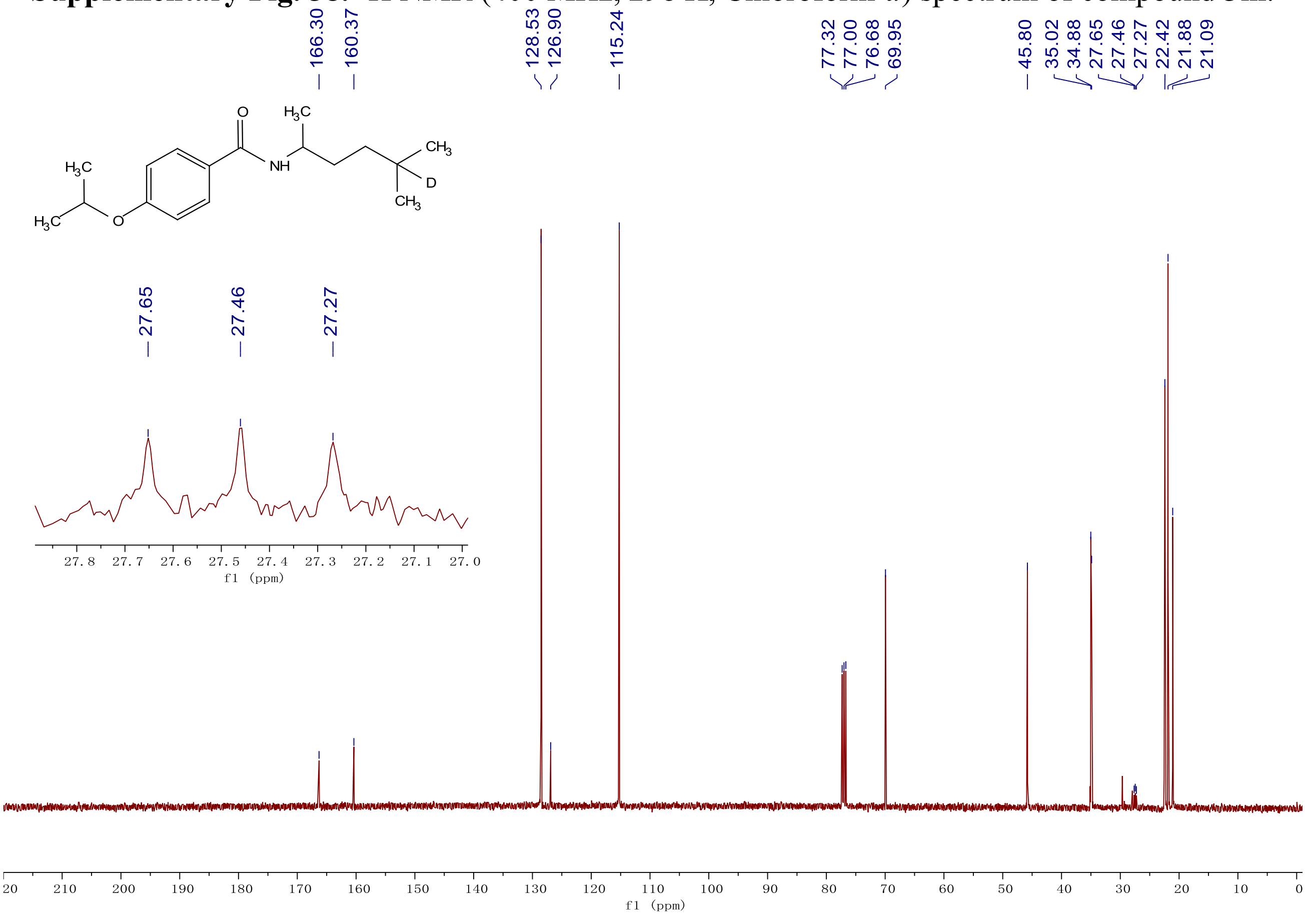

Supplementary Fig. 39. ^{13}C NMR (101 MHz, 298 K, Chloroform-*d*) spectrum of compound **3m**.

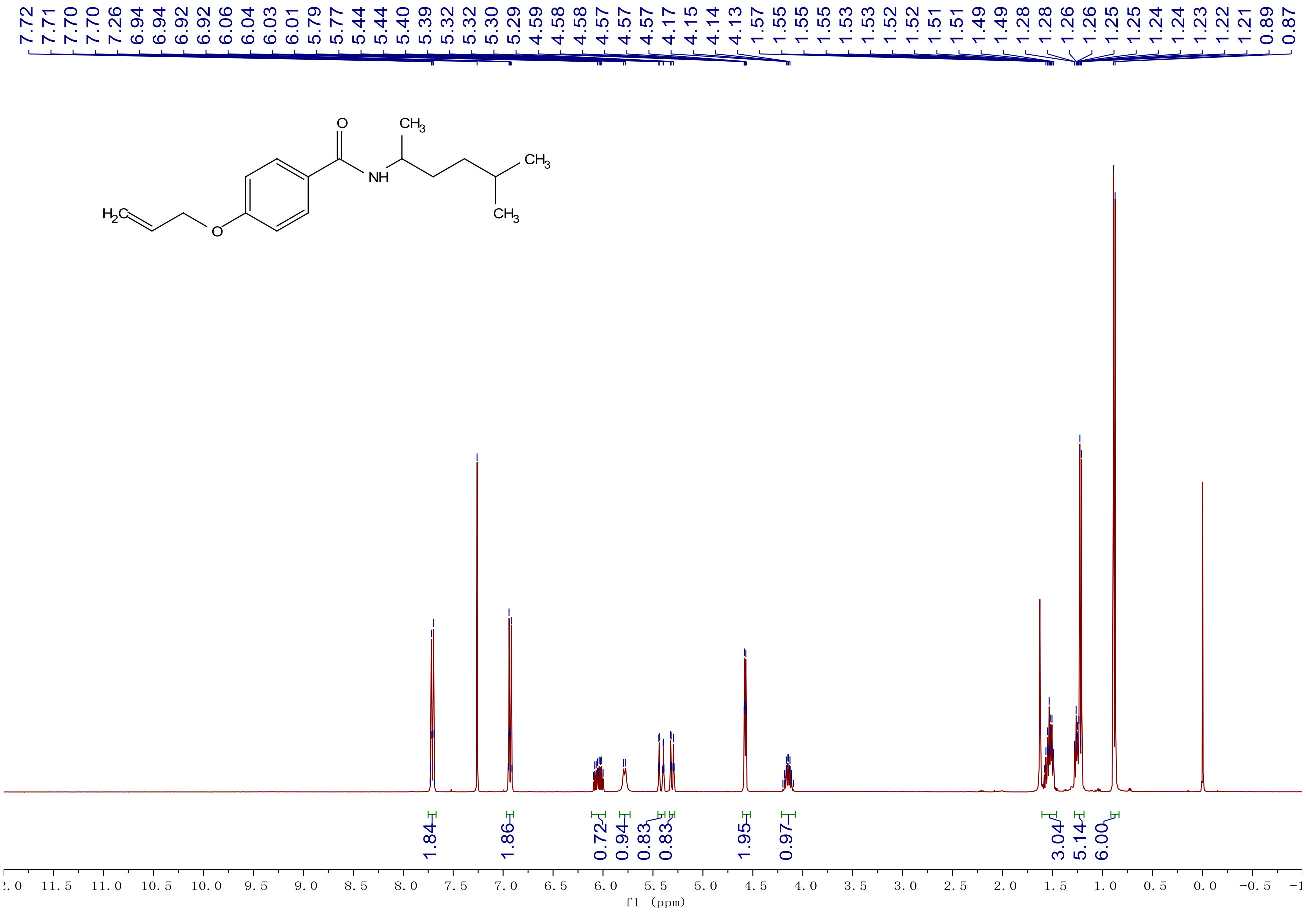

Supplementary Fig. 40. ^{1}H NMR (400 MHz, 298 K, Chloroform-*d*) spectrum of compound **1n**.

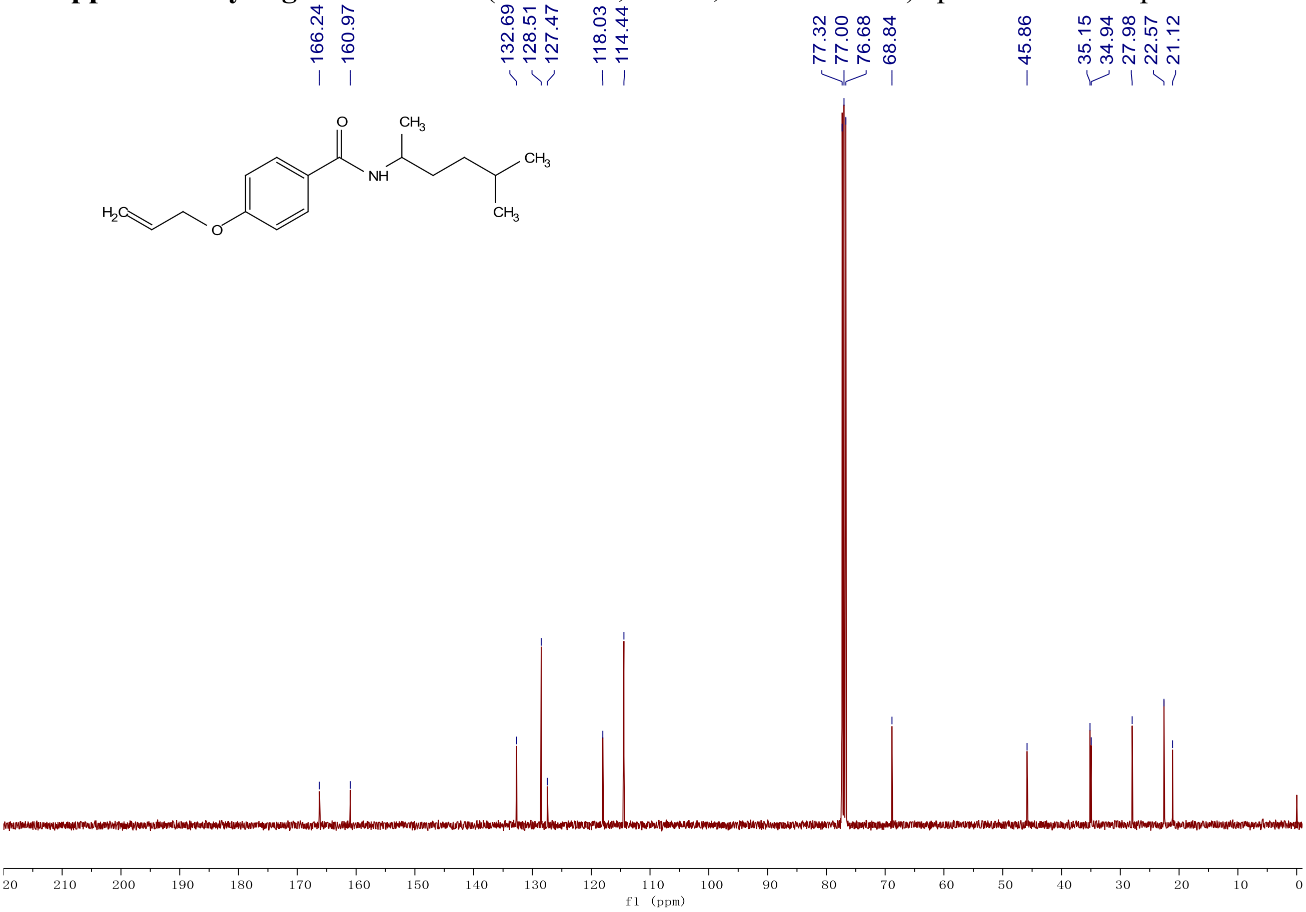

Supplementary Fig. 41. ^{13}C NMR (101 MHz, 298 K, Chloroform-*d*) spectrum of compound **1n**.

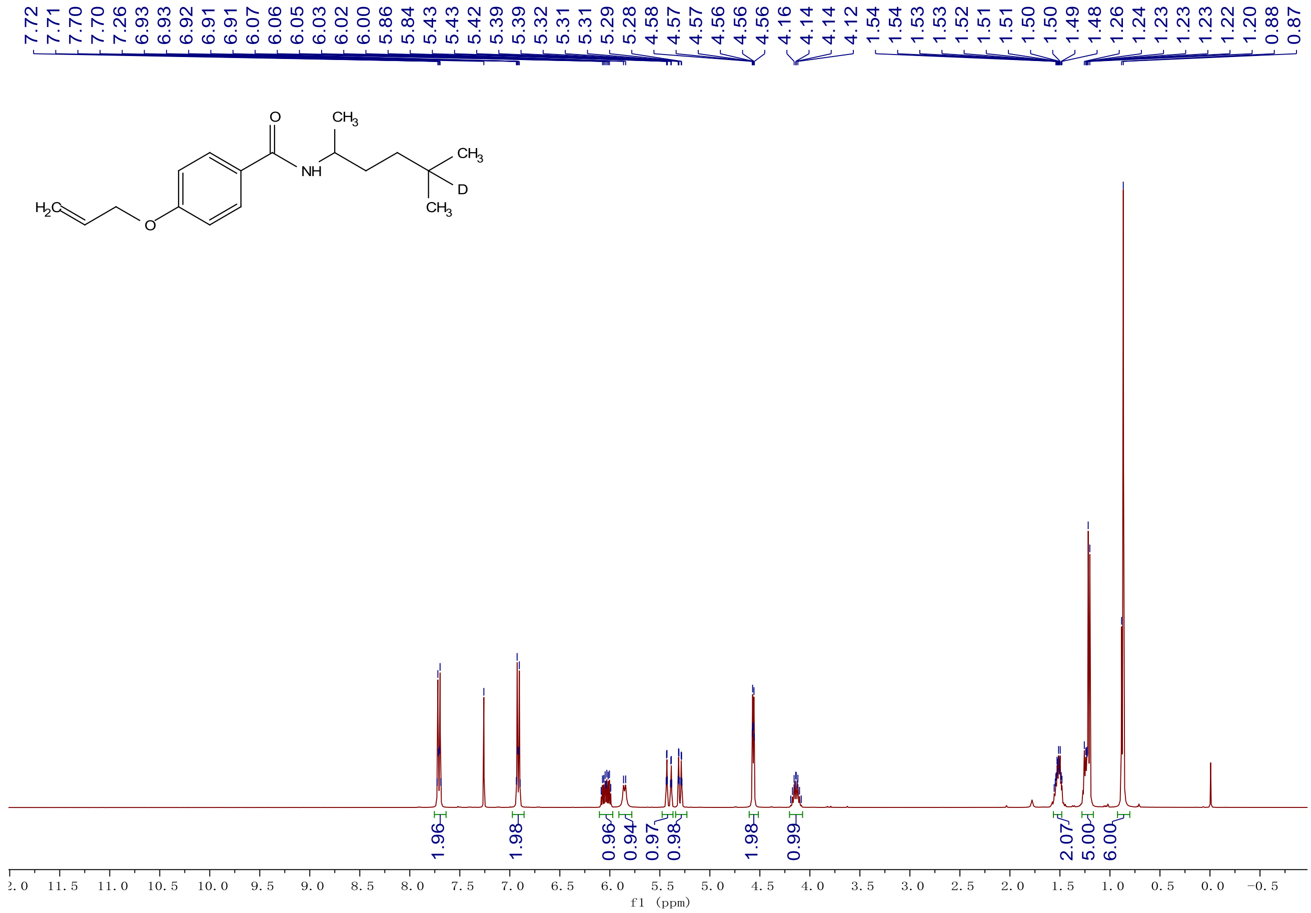

Supplementary Fig. 42. ^{1}H NMR (400 MHz, 298 K, Chloroform-d) spectrum of compound **3n**.

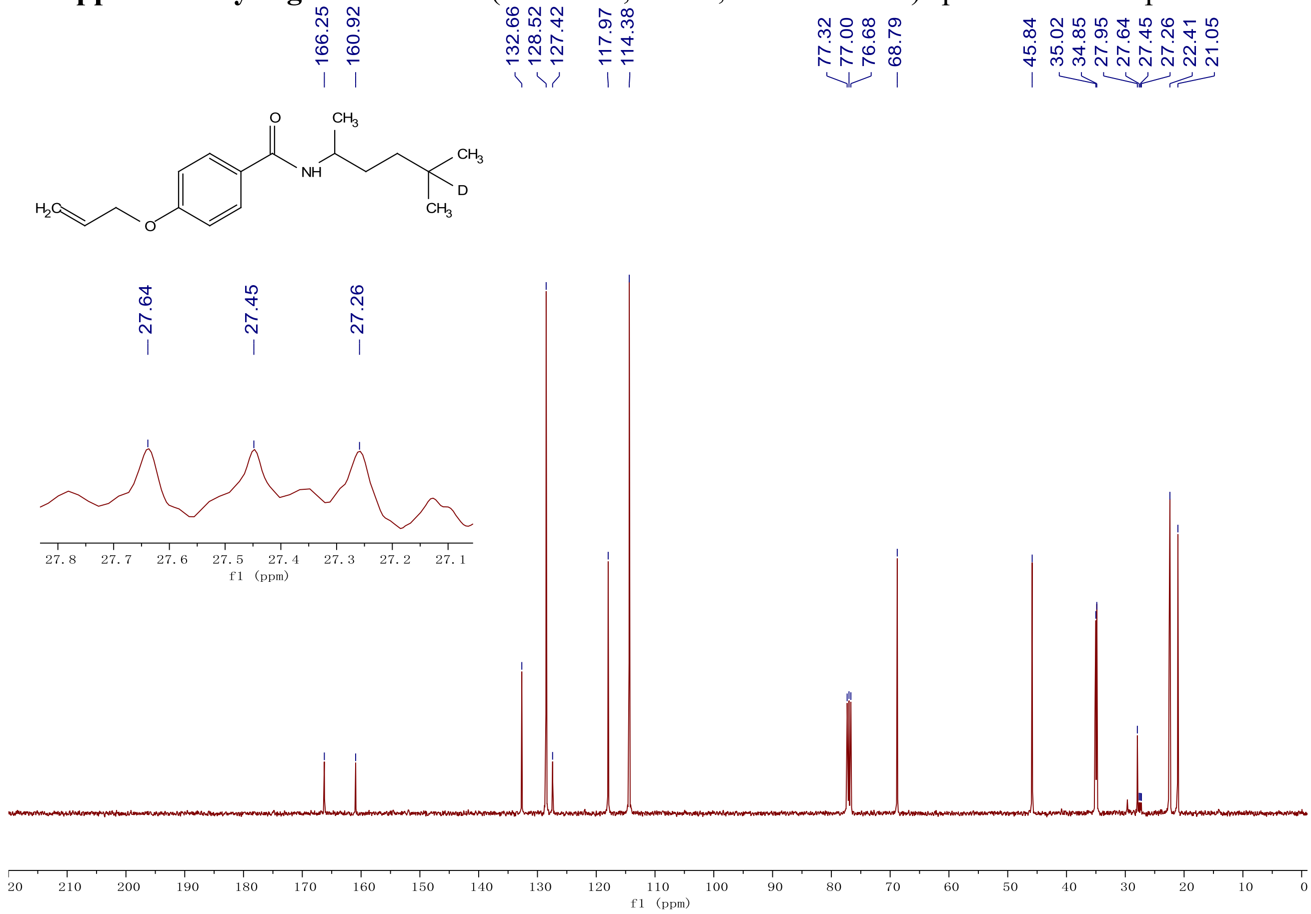

Supplementary Fig. 43. ^{13}C NMR (101 MHz, 298 K, Chloroform-d) spectrum of compound **3n**.

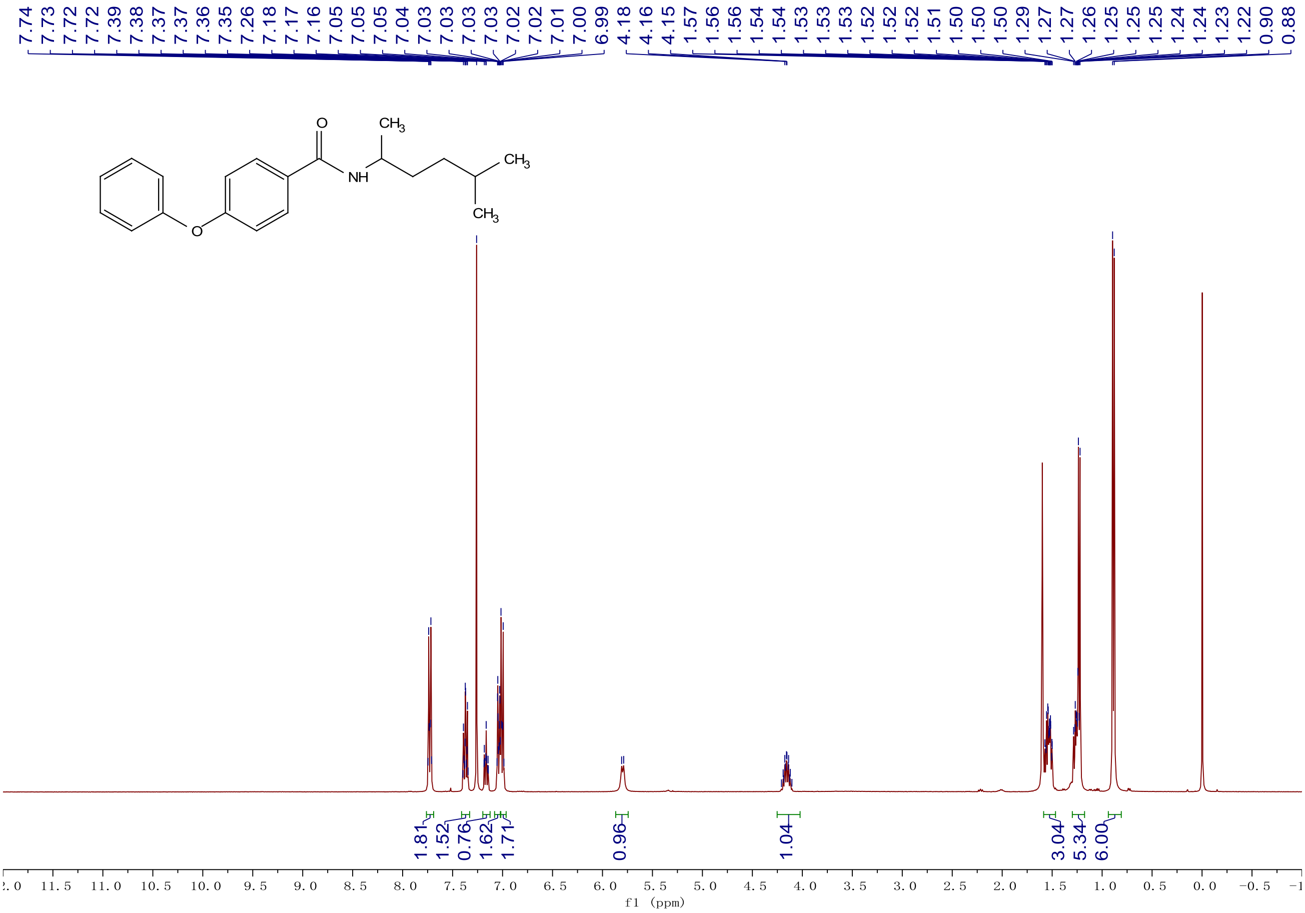

Supplementary Fig. 44. ^{1}H NMR (400 MHz, 298 K, Chloroform-*d*) spectrum of compound **1o**.

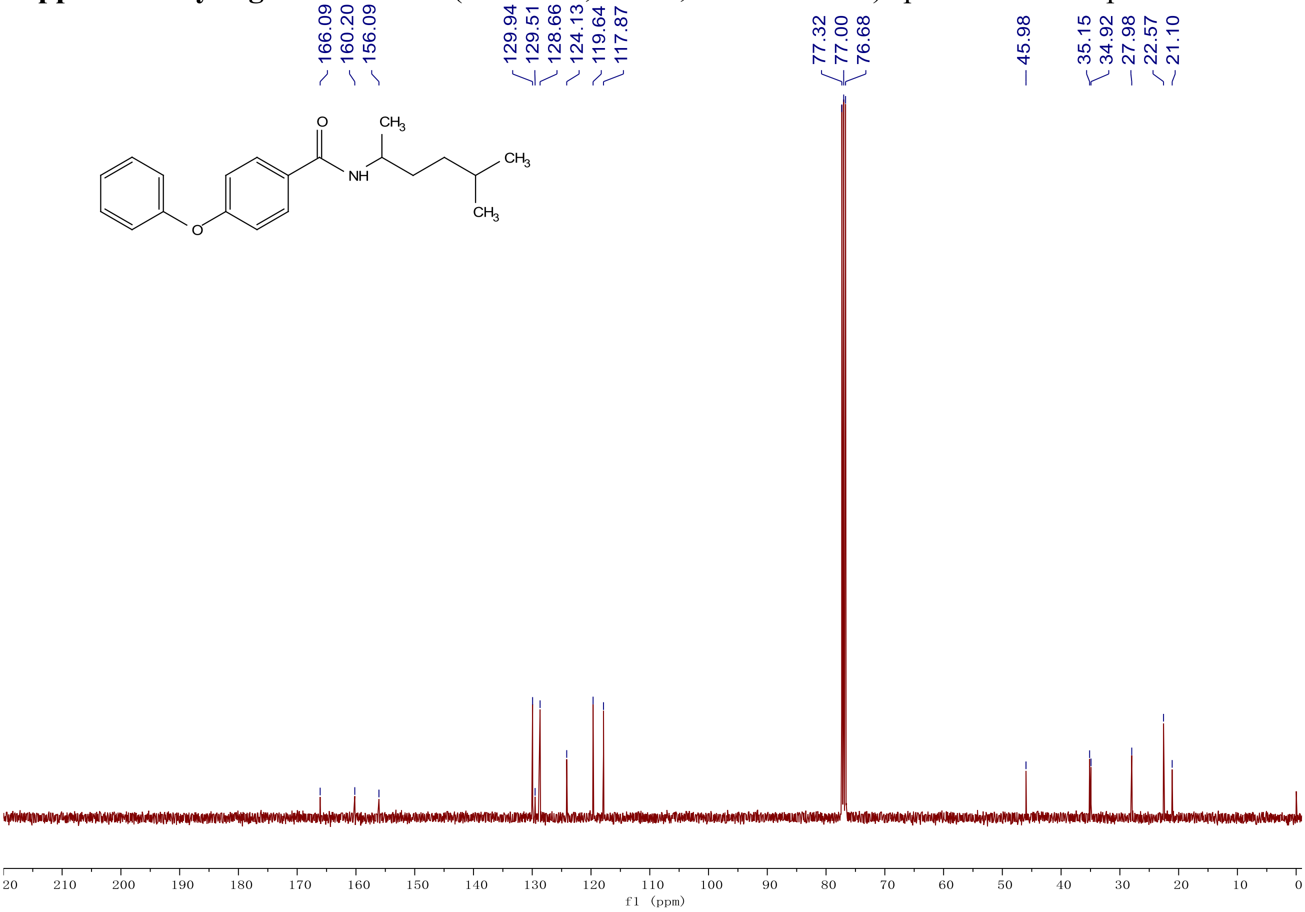

Supplementary Fig. 45. ^{13}C NMR (101 MHz, 298 K, Chloroform-*d*) spectrum of compound **1o**.

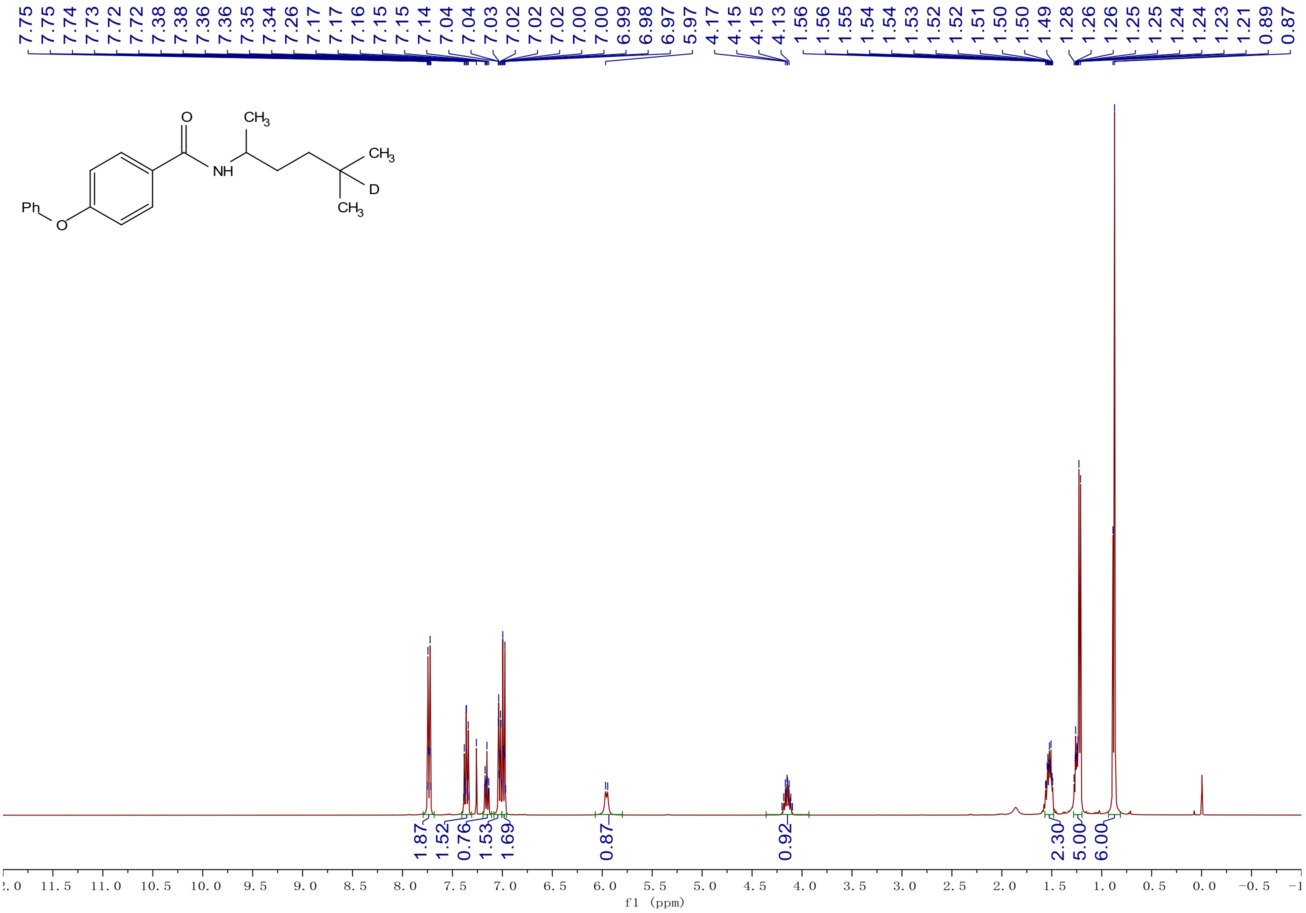

Supplementary Fig. 46. ^{1}H NMR (400 MHz, 298 K, Chloroform-*d*) spectrum of compound **3o**.

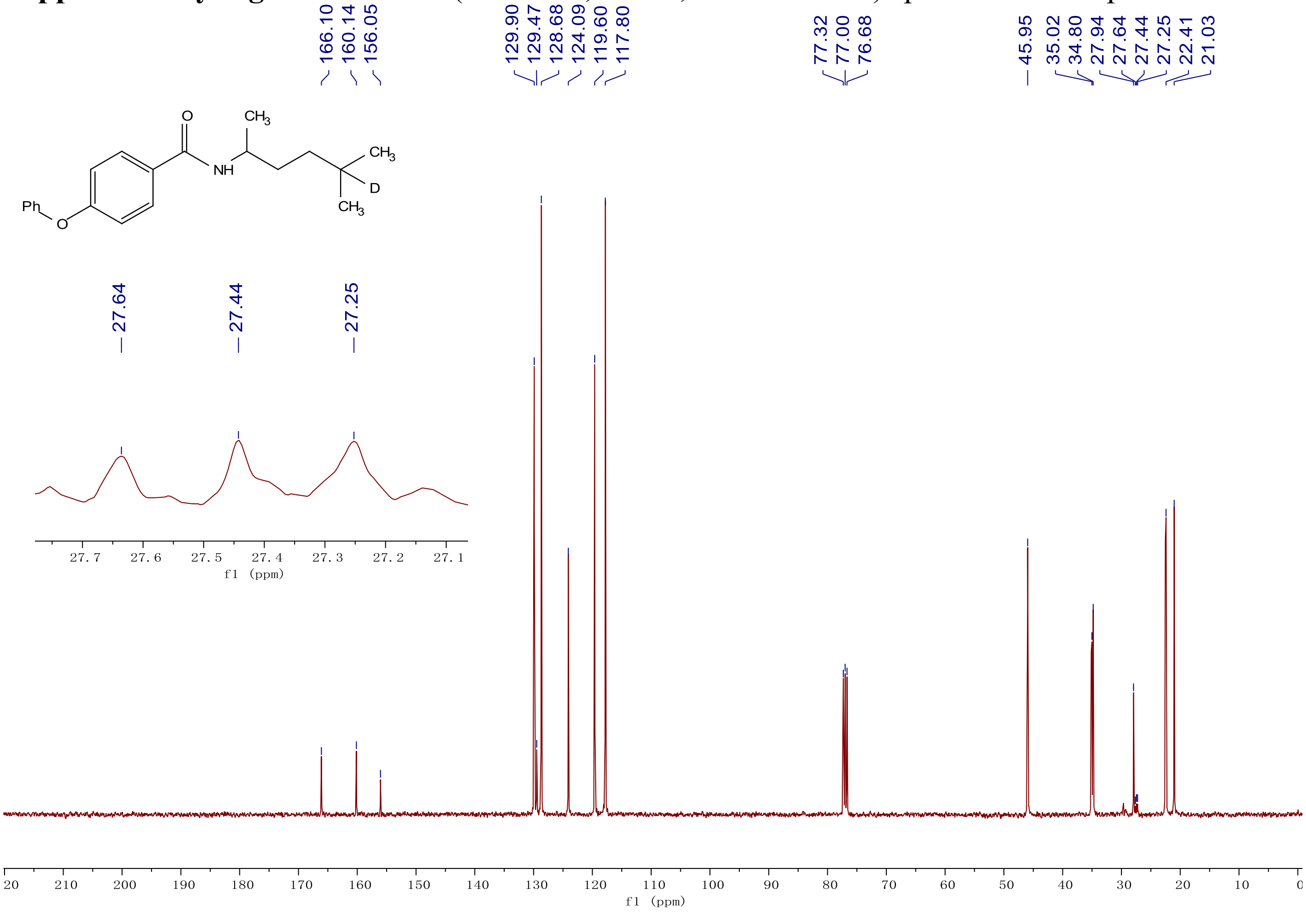

Supplementary Fig. 47. ^{13}C NMR (101 MHz, 298 K, Chloroform-*d*) spectrum of compound **3o**.

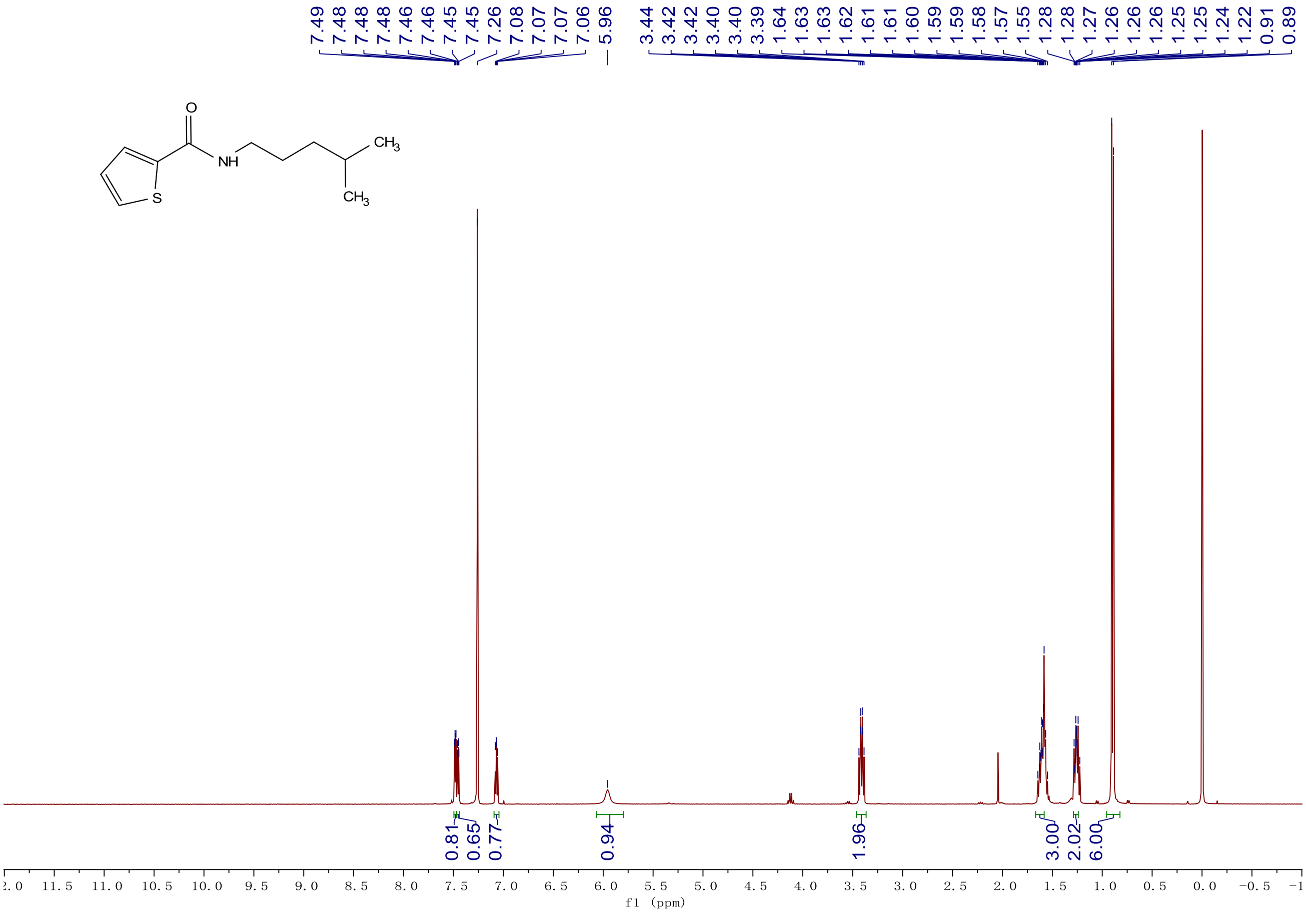

Supplementary Fig. 48. ^{1}H NMR (400 MHz, 298 K, Chloroform-*d*) spectrum of compound **1p**.

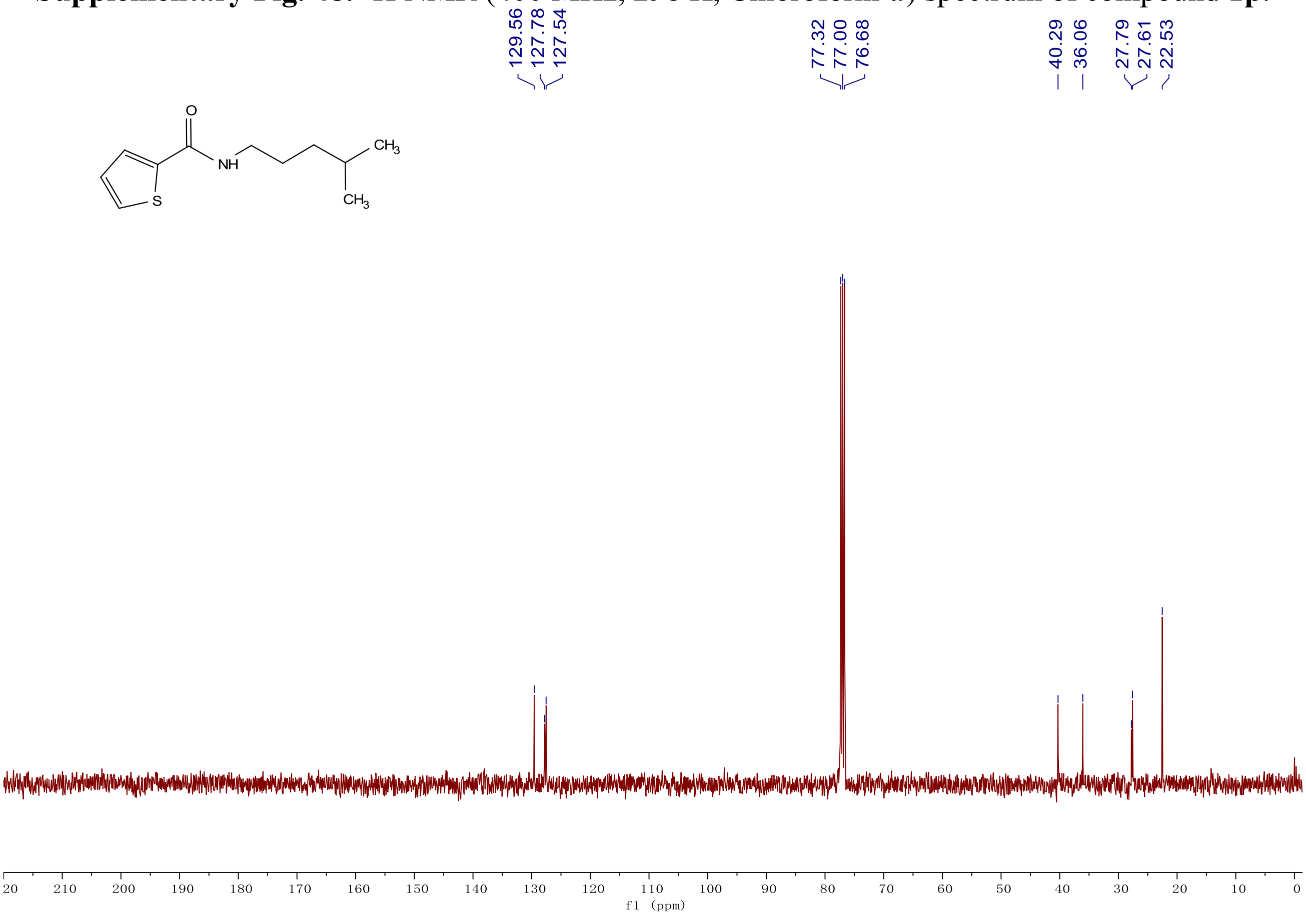

Supplementary Fig. 49. ^{13}C NMR (101 MHz, 298 K, Chloroform-*d*) spectrum of compound **1p**.

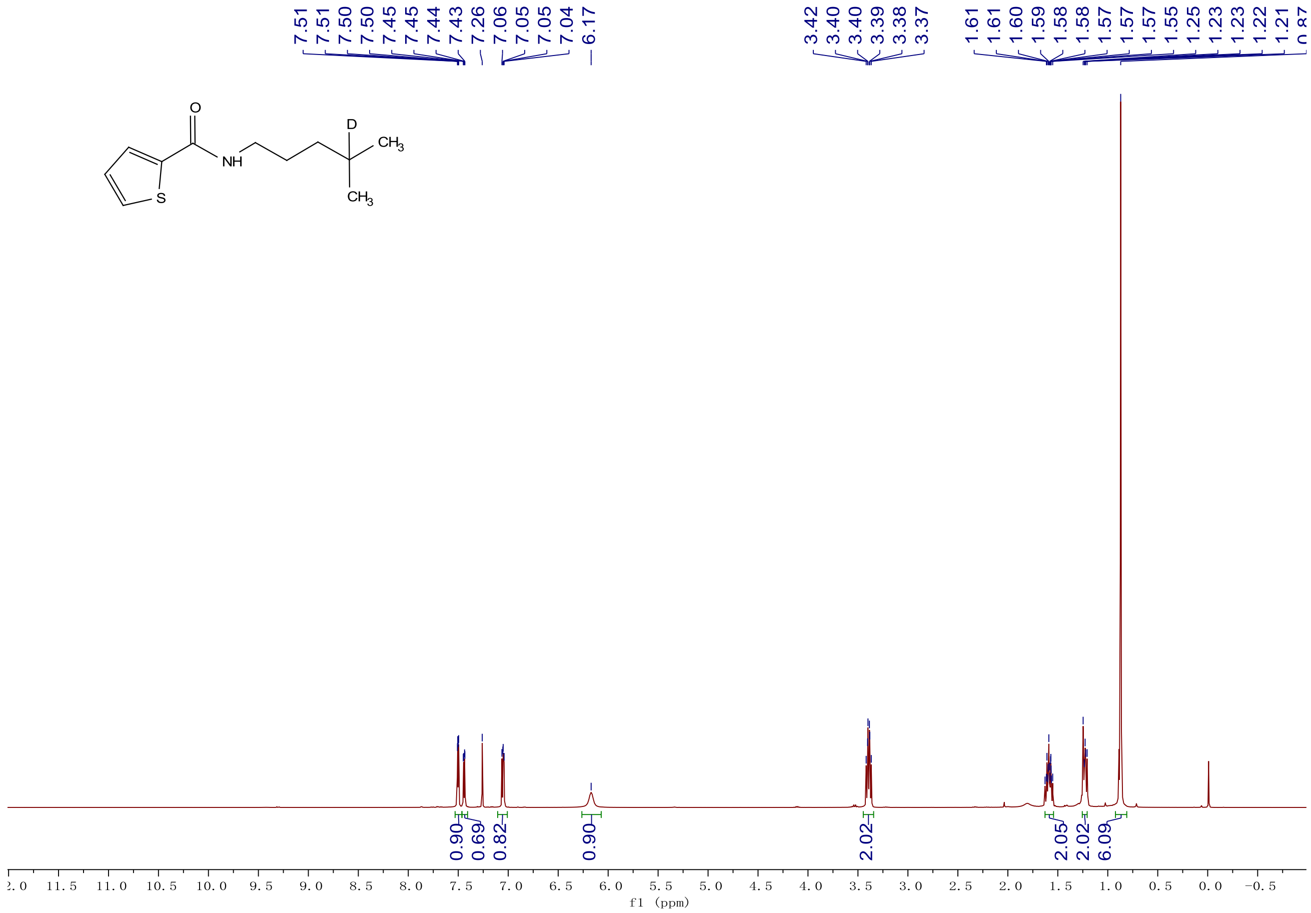

Supplementary Fig. 50. ^{1}H NMR (400 MHz, 298 K, Chloroform-*d*) spectrum of compound **3p**.

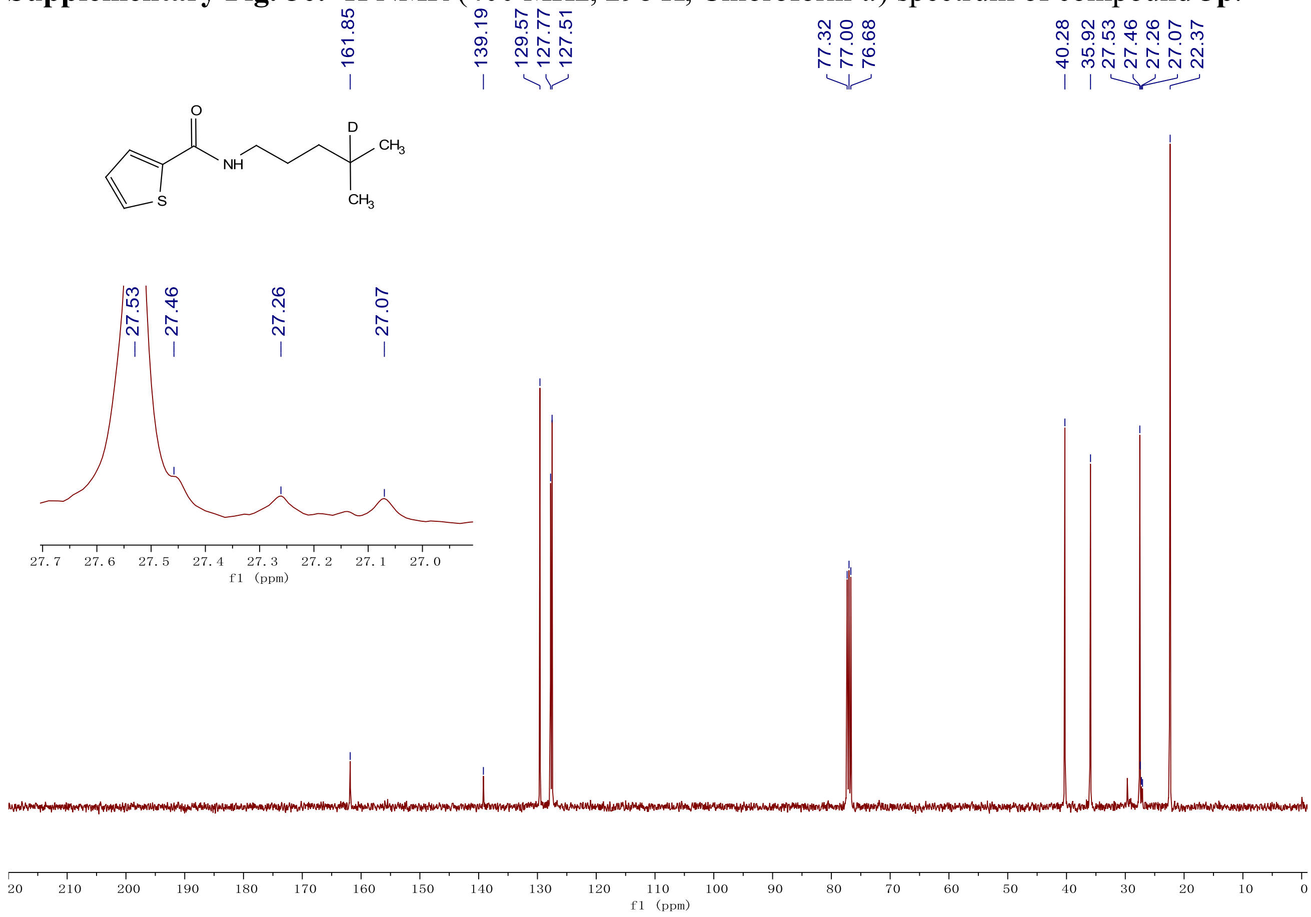

Supplementary Fig. 51. ^{13}C NMR (101 MHz, 298 K, Chloroform-*d*) spectrum of compound **3p**.

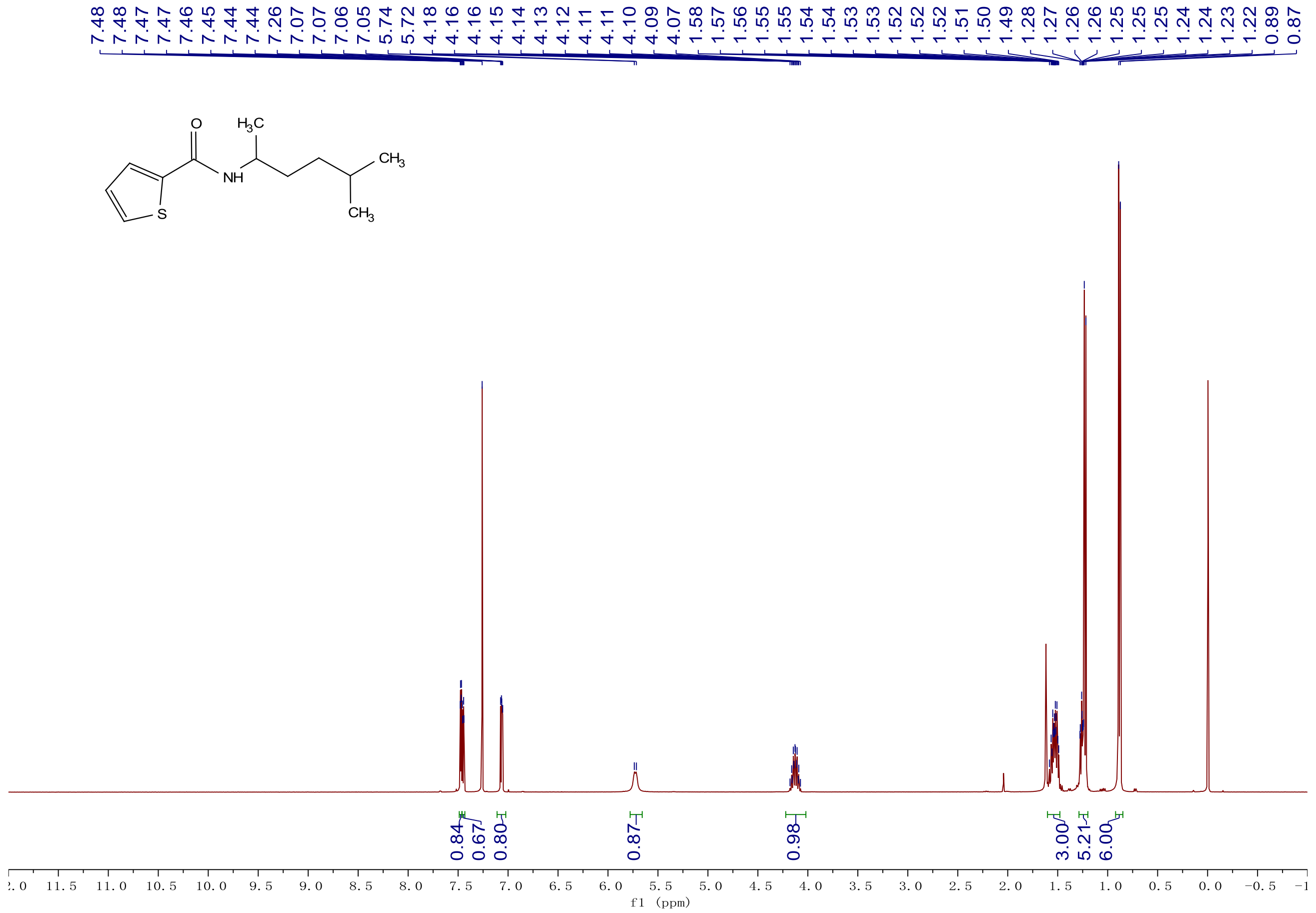

Supplementary Fig. 52. ^{1}H NMR (400 MHz, 298 K, Chloroform-*d*) spectrum of compound **1q**.

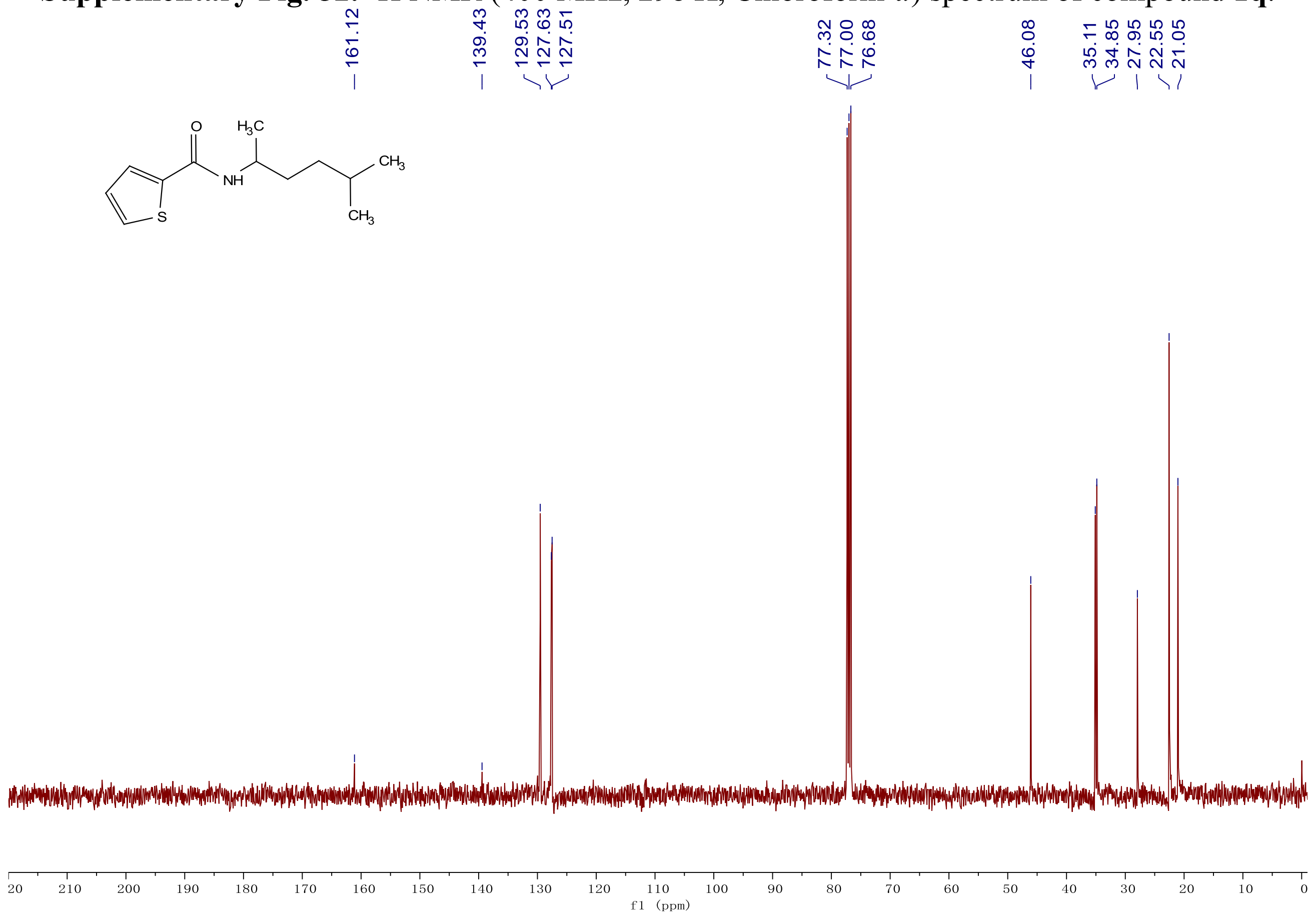

Supplementary Fig. 53. ^{13}C NMR (101 MHz, 298 K, Chloroform-*d*) spectrum of compound **1q**.

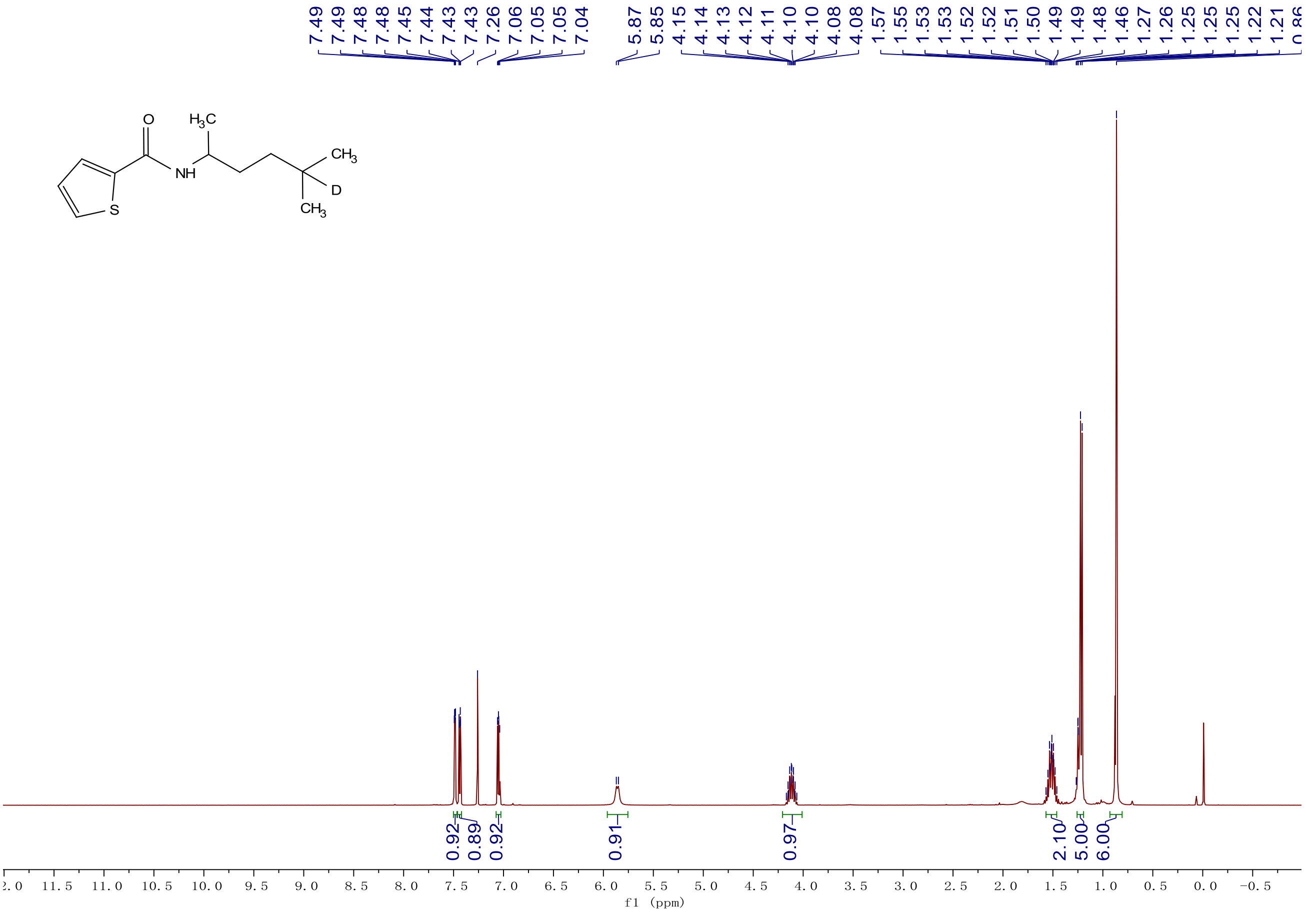

Supplementary Fig. 54. ^{1}H NMR (400 MHz, 298 K, Chloroform-*d*) spectrum of compound **3q**.

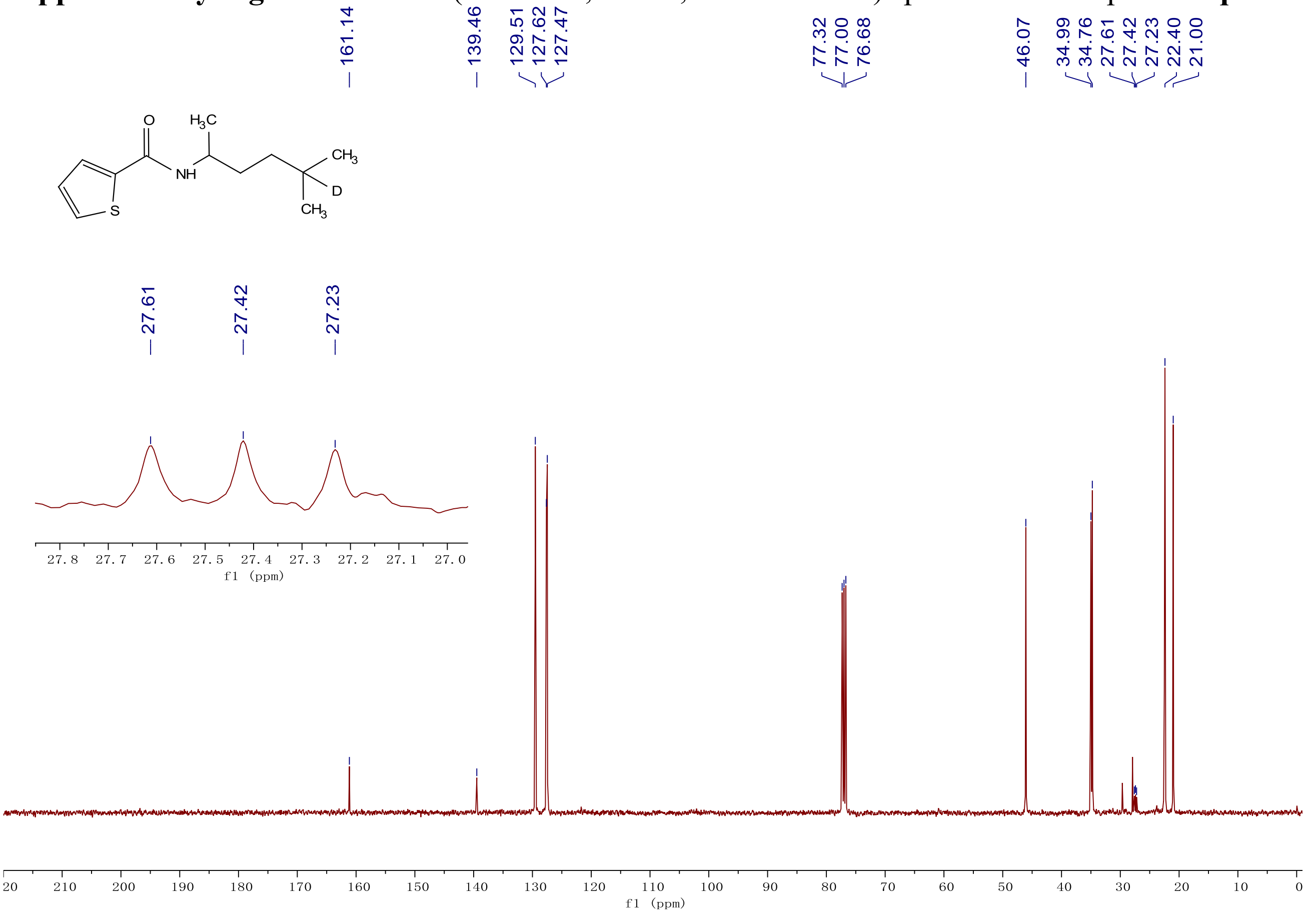

Supplementary Fig. 55. ^{13}C NMR (101 MHz, 298 K, Chloroform-*d*) spectrum of compound **3q**.

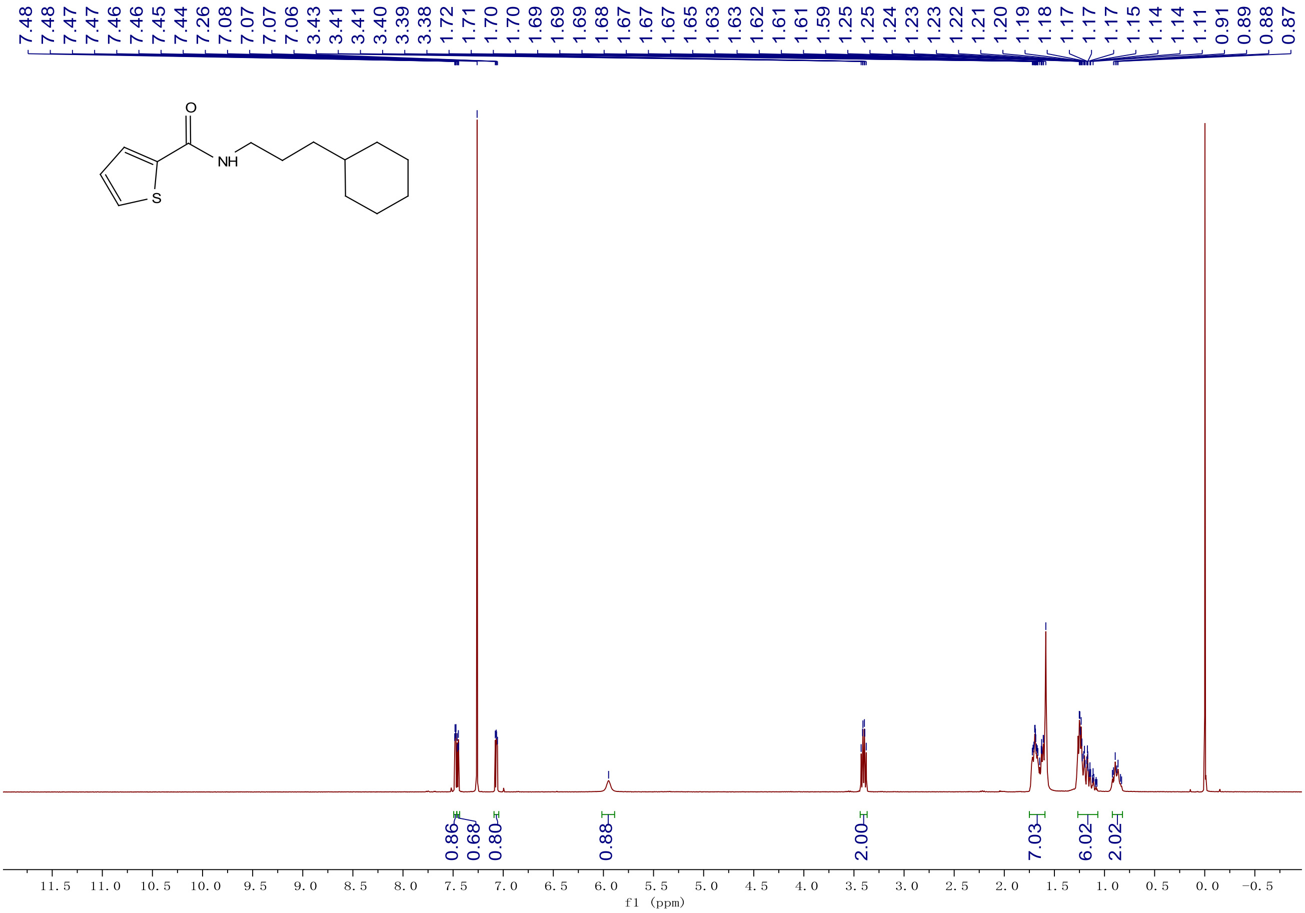

Supplementary Fig. 56. ^{1}H NMR (400 MHz, 298 K, Chloroform-*d*) spectrum of compound **1r**.

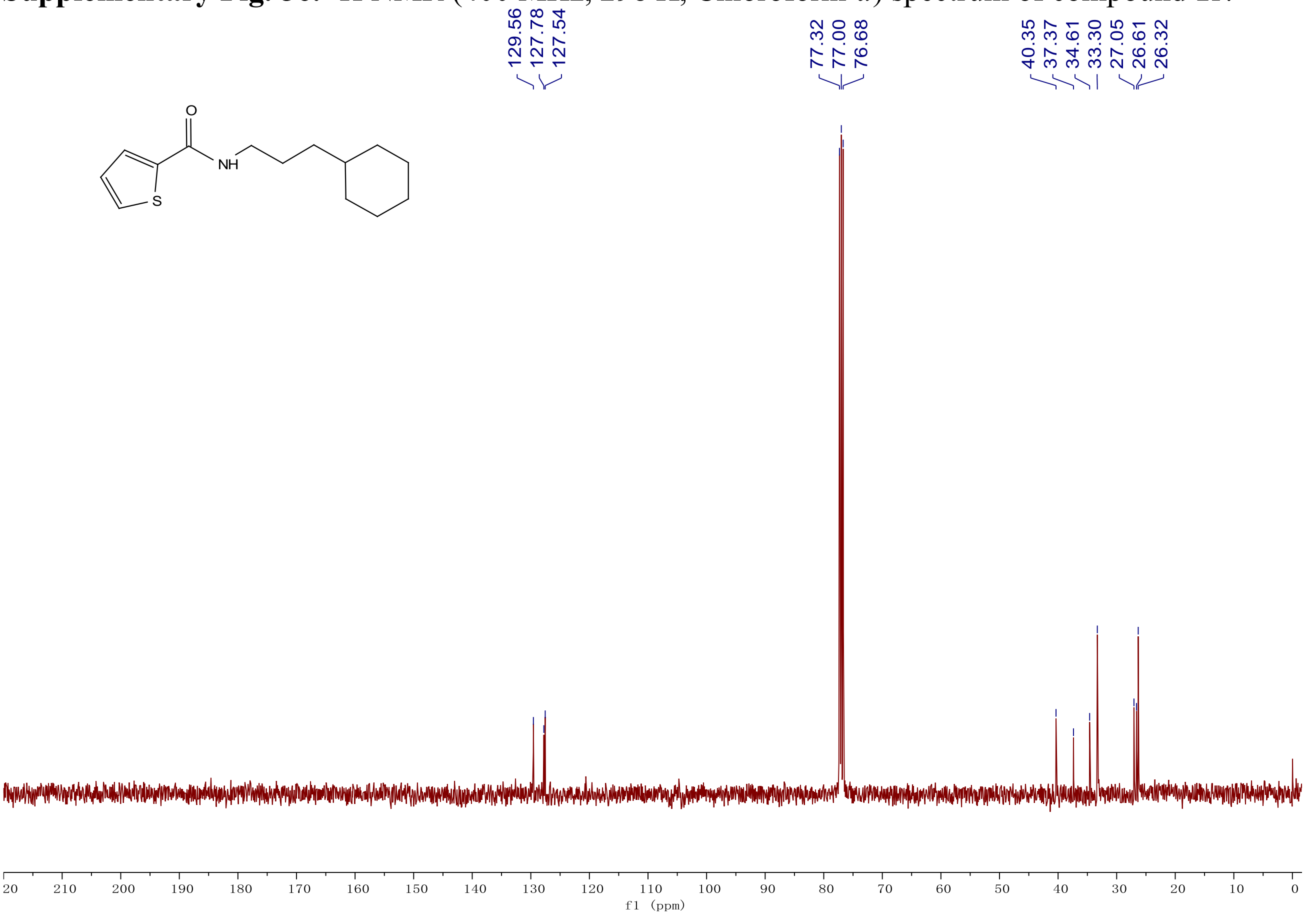

Supplementary Fig. 57. ^{13}C NMR (101 MHz, 298 K, Chloroform-*d*) spectrum of compound **1r**.

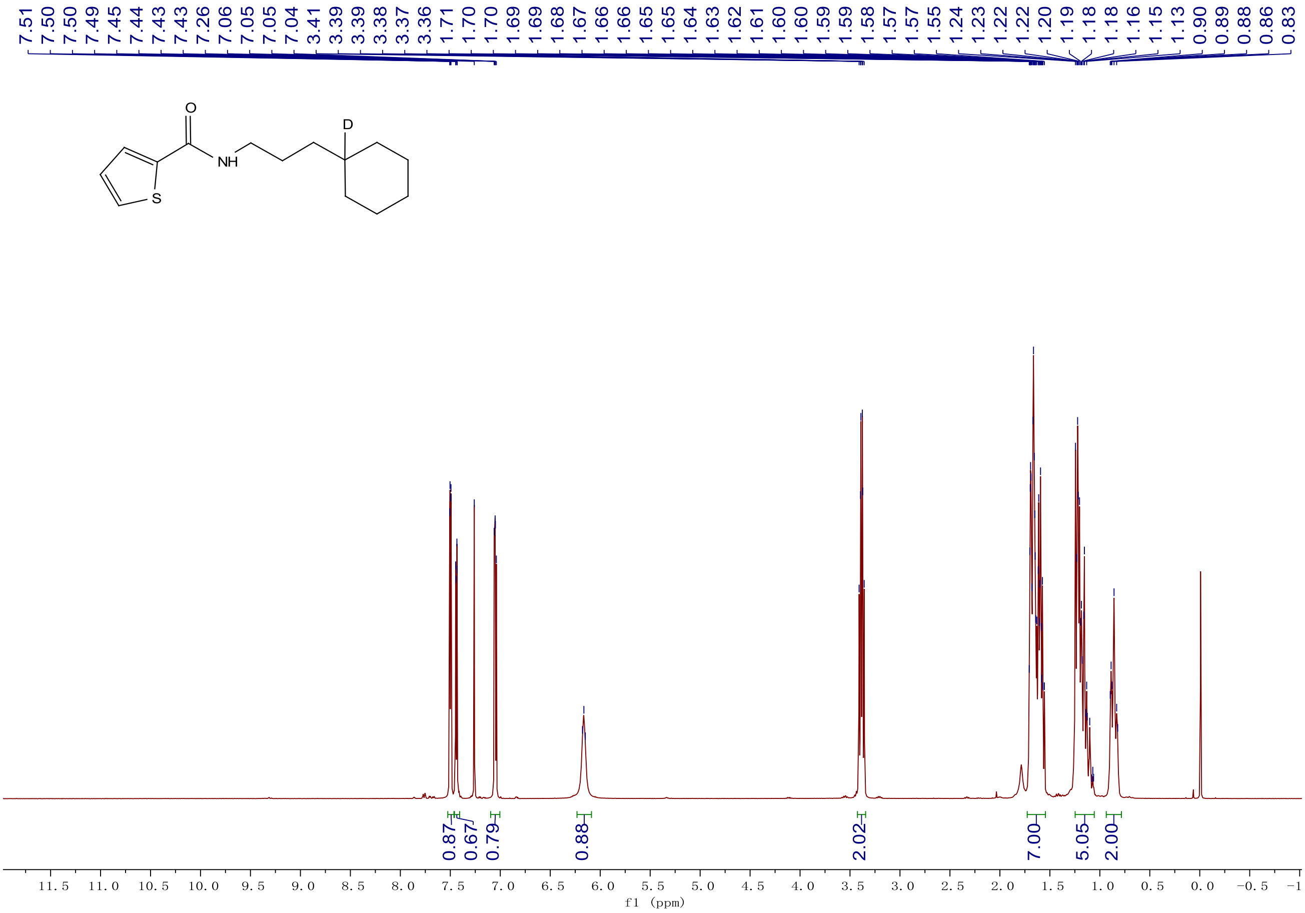

Supplementary Fig. 58. ^{1}H NMR (400 MHz, 298 K, Chloroform-*d*) spectrum of compound **3r**.

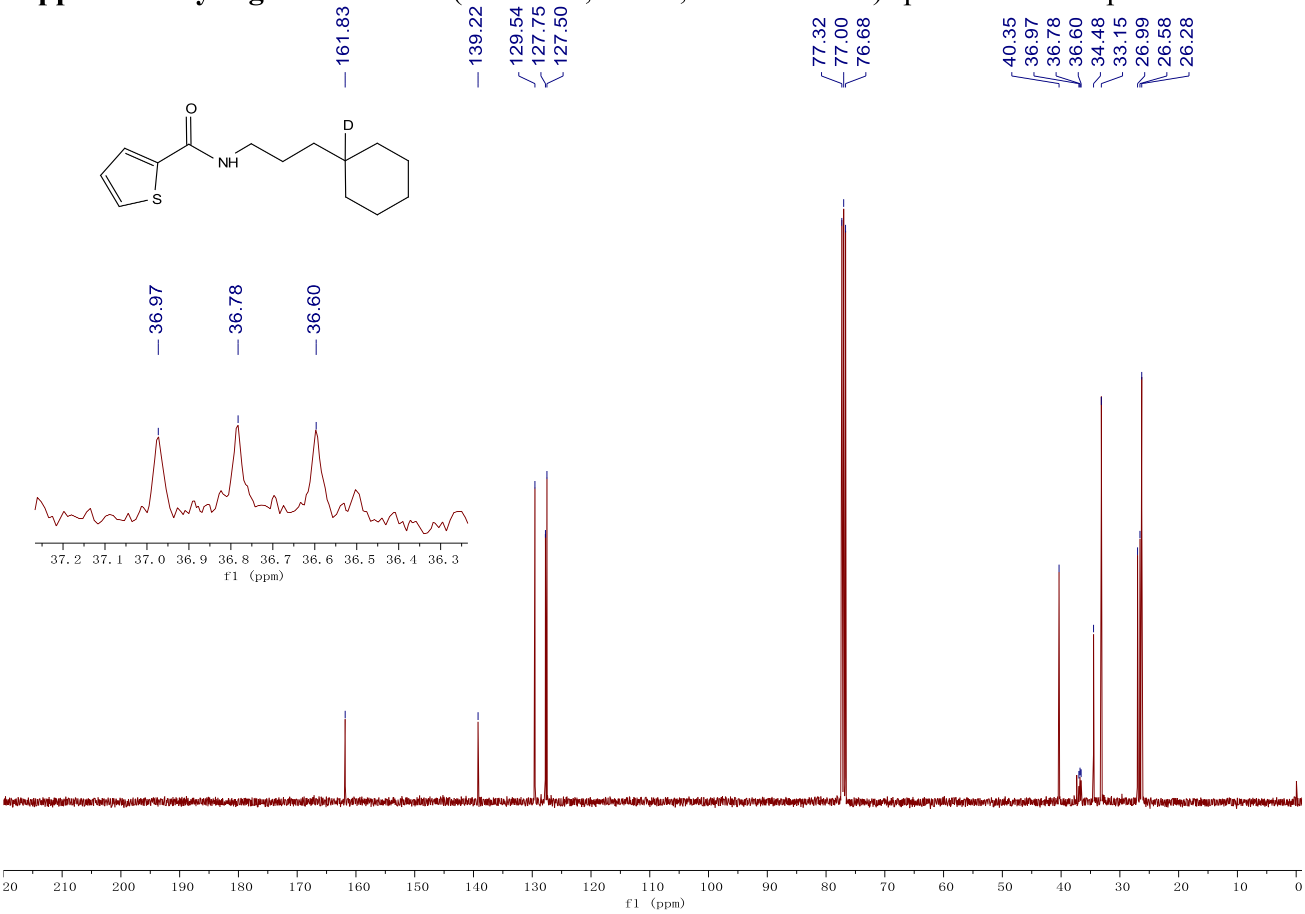

Supplementary Fig. 59. ^{13}C NMR (101 MHz, 298 K, Chloroform-*d*) spectrum of compound **3r**.

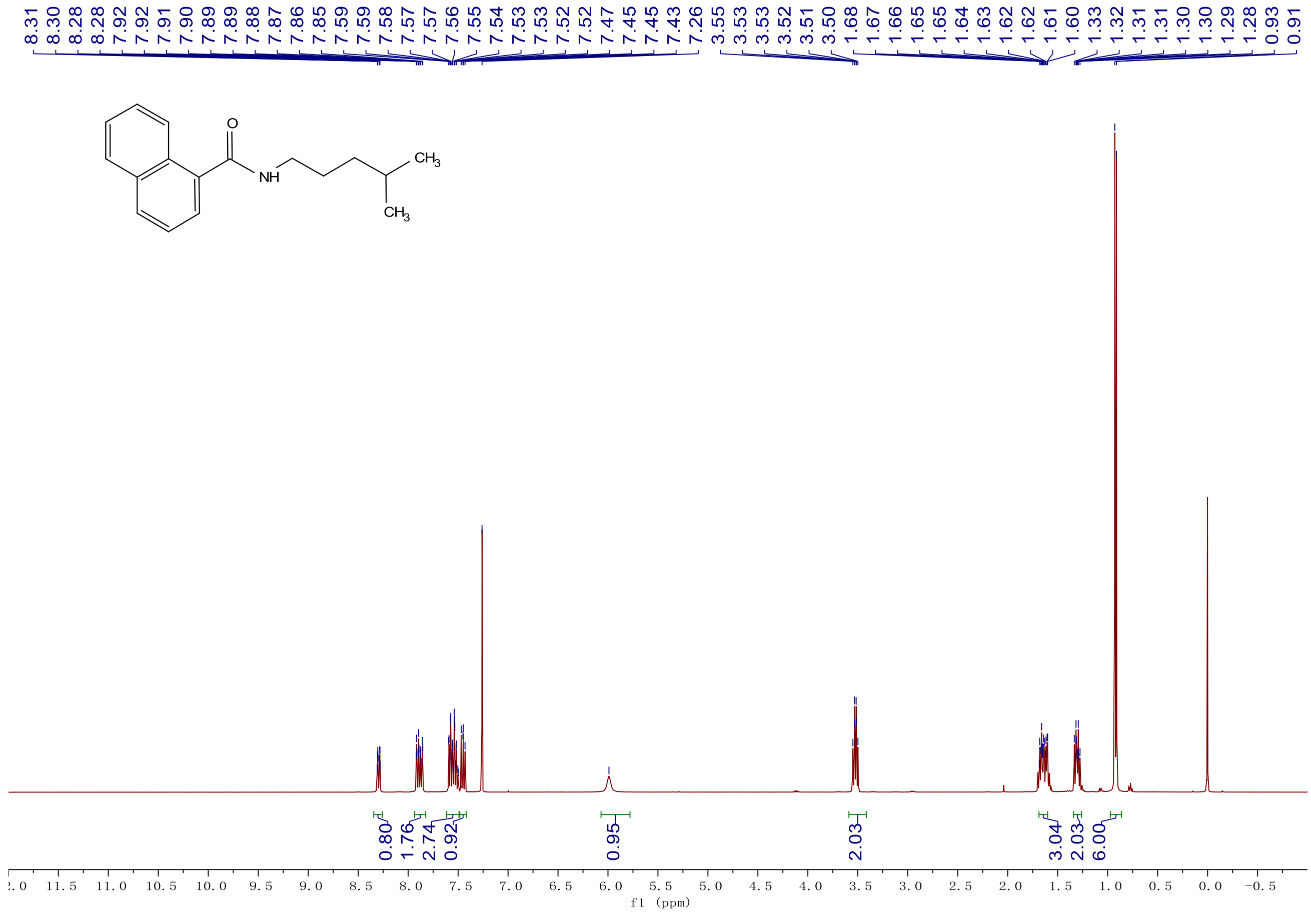

Supplementary Fig. 60. ^{1}H NMR (400 MHz, 298 K, Chloroform-*d*) spectrum of compound **1s**.

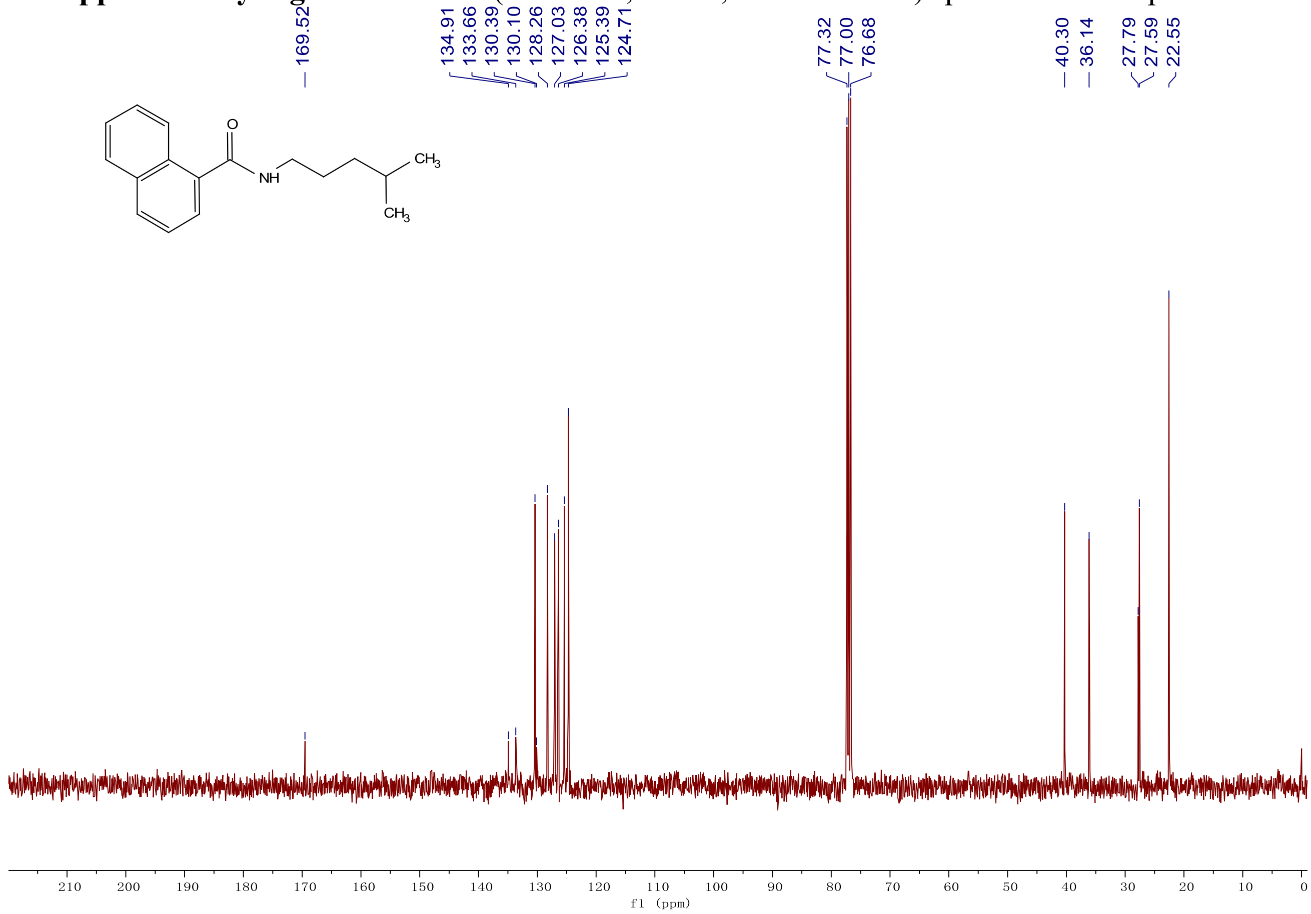

Supplementary Fig. 61. ^{13}C NMR (101 MHz, 298 K, Chloroform-*d*) spectrum of compound **1s**.

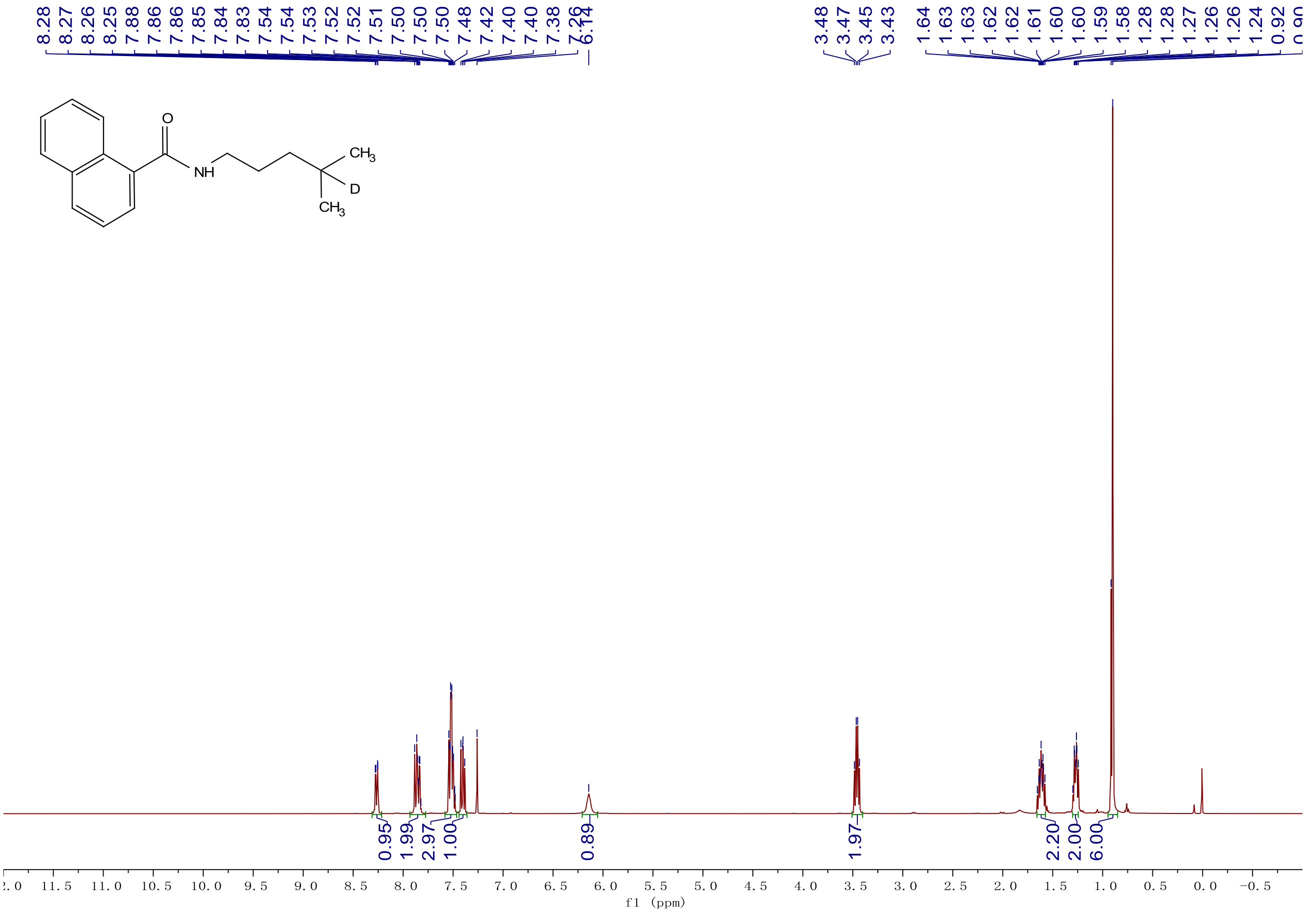

Supplementary Fig. 62. ^{1}H NMR (400 MHz, 298 K, Chloroform-*d*) spectrum of compound **3s**.

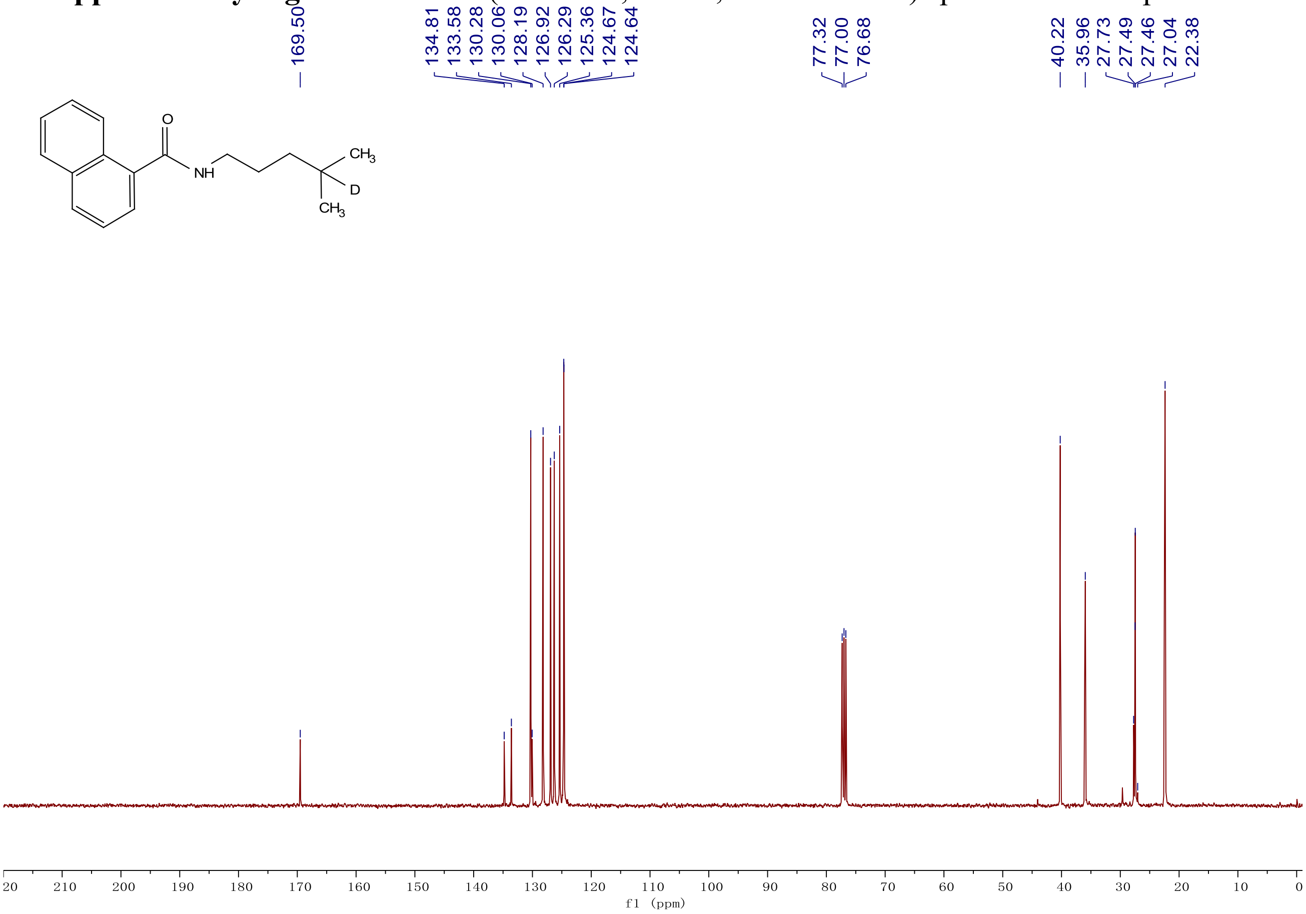

Supplementary Fig. 63. ^{13}C NMR (101 MHz, 298 K, Chloroform-*d*) spectrum of compound **3s**.

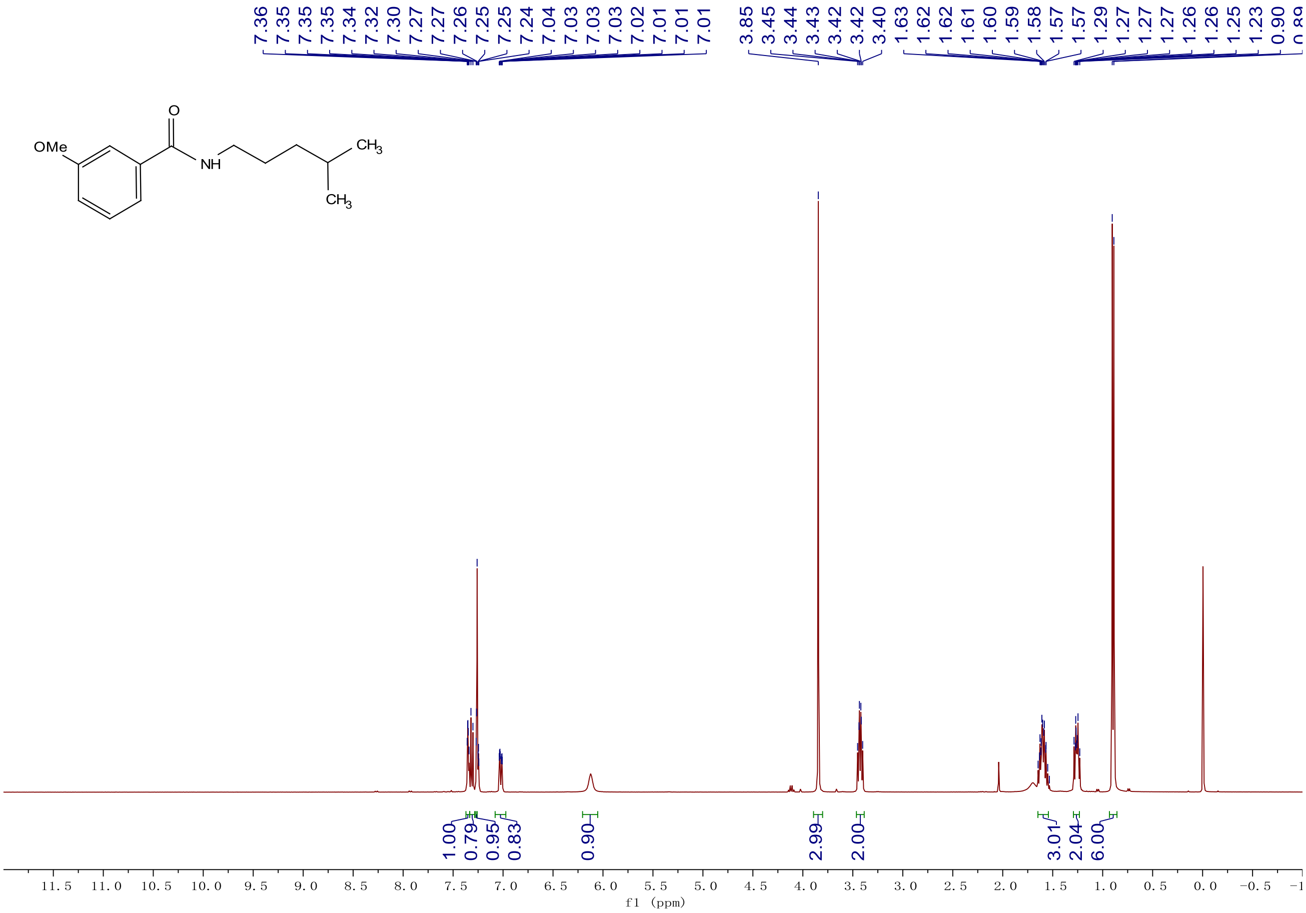

Supplementary Fig. 64. ^{1}H NMR (400 MHz, 298 K, Chloroform-*d*) spectrum of compound **1t**.

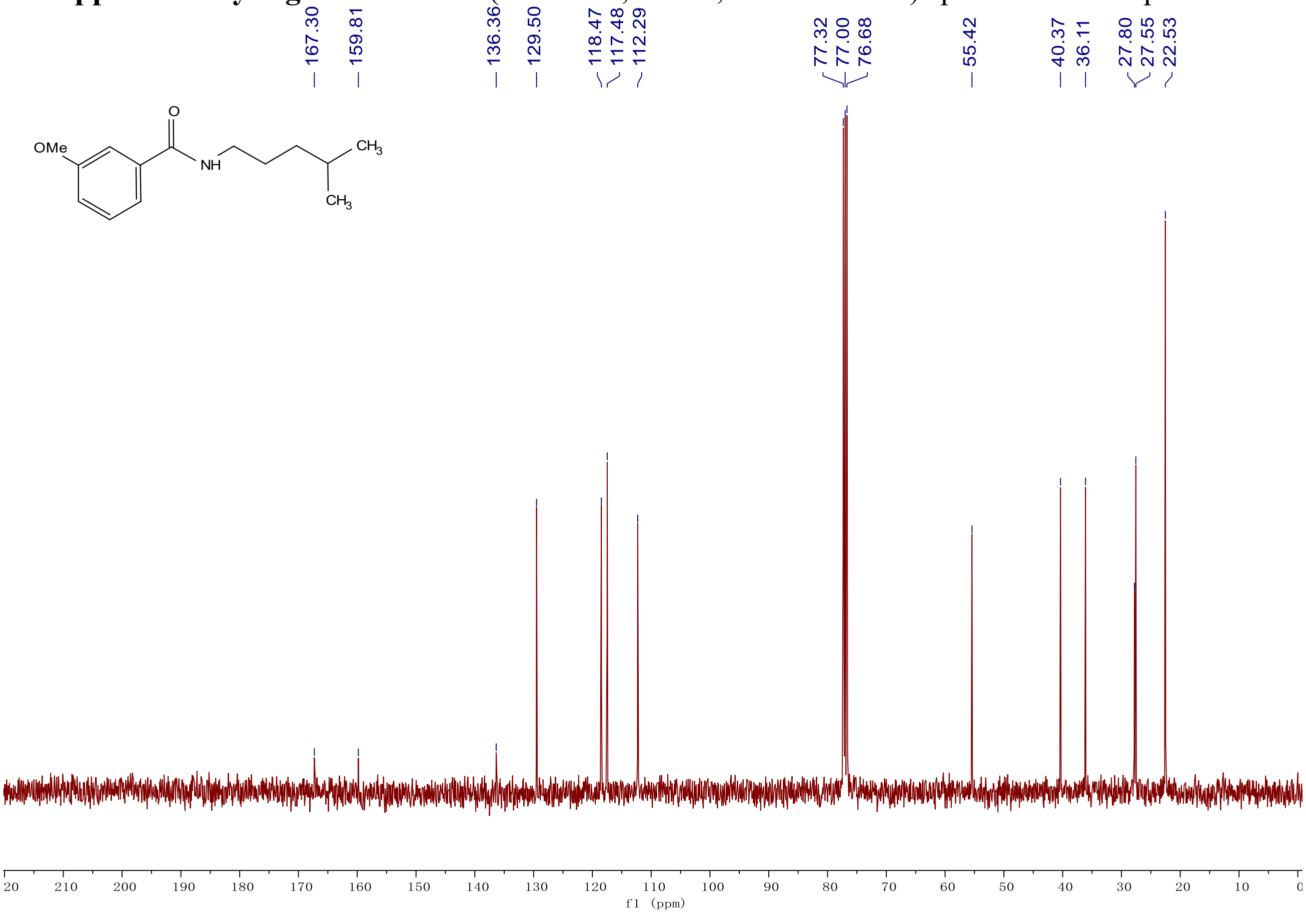

Supplementary Fig. 65. ^{13}C NMR (101 MHz, 298 K, Chloroform-*d*) spectrum of compound **1t**.

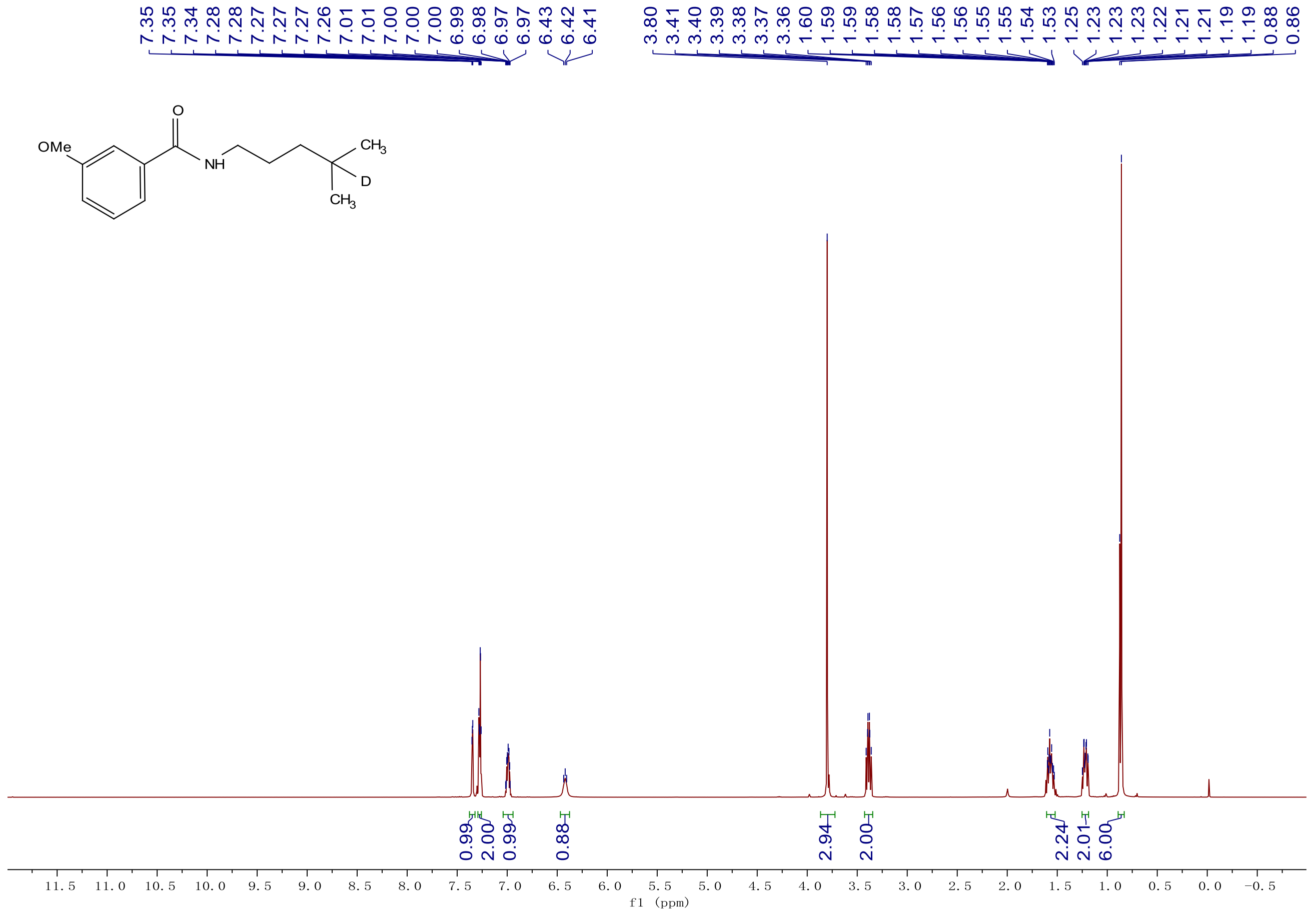

Supplementary Fig. 66. ^{1}H NMR (400 MHz, 298 K, Chloroform-*d*) spectrum of compound **3t**.

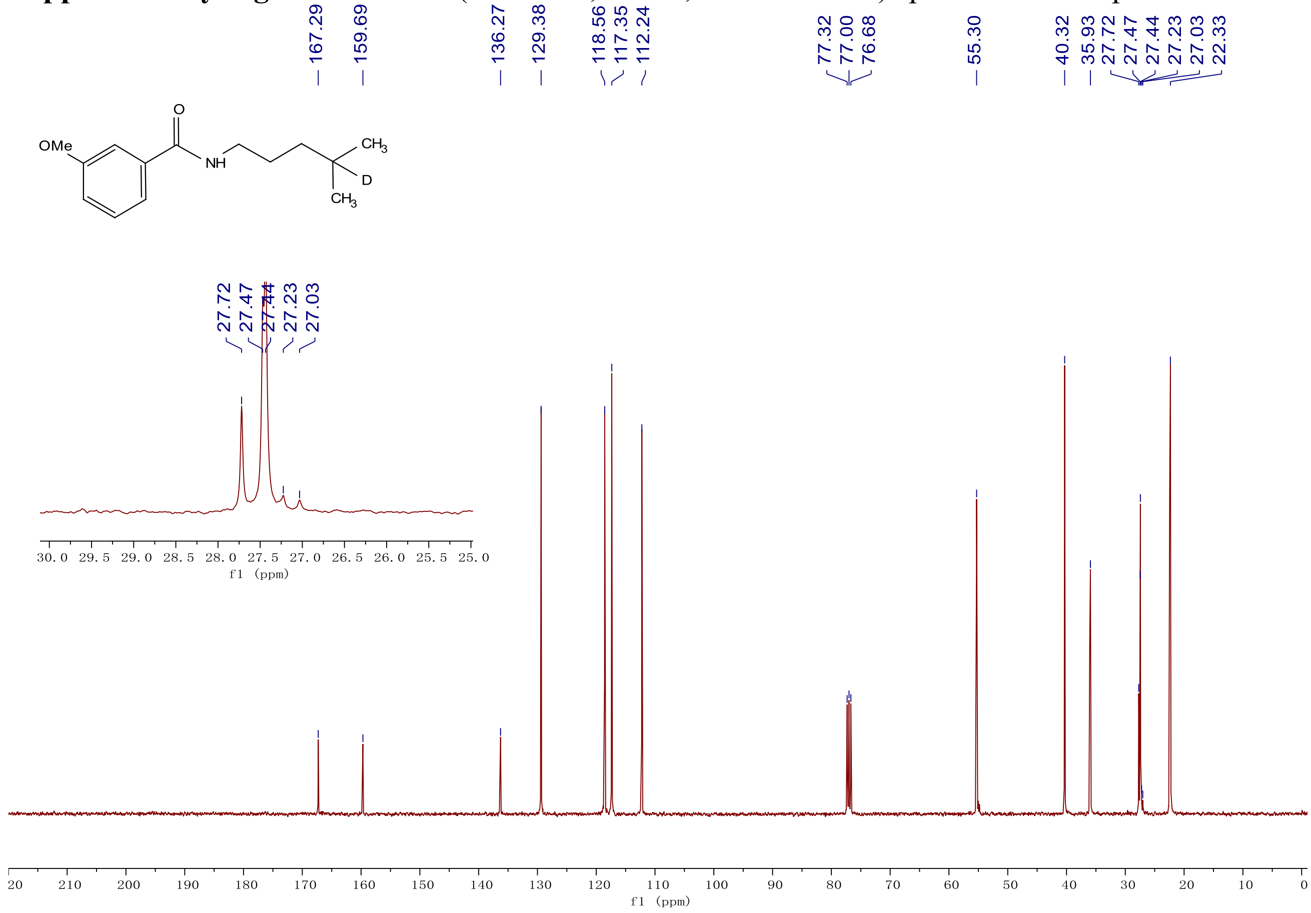

Supplementary Fig. 67. ^{13}C NMR (101 MHz, 298 K, Chloroform-*d*) spectrum of compound **3t**.

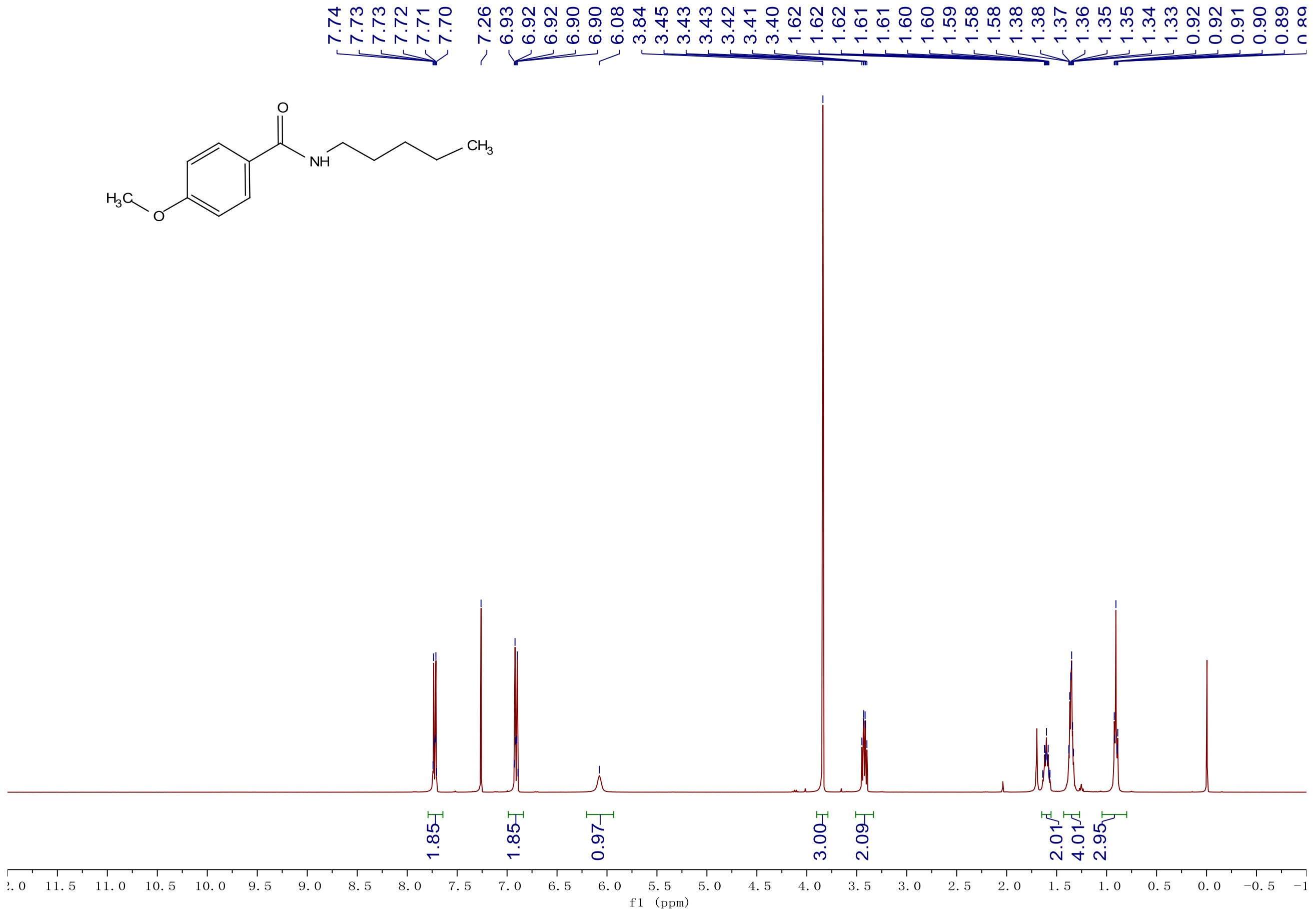

Supplementary Fig. 68. ^{1}H NMR (400 MHz, 298 K, Chloroform-*d*) spectrum of compound **1u**.

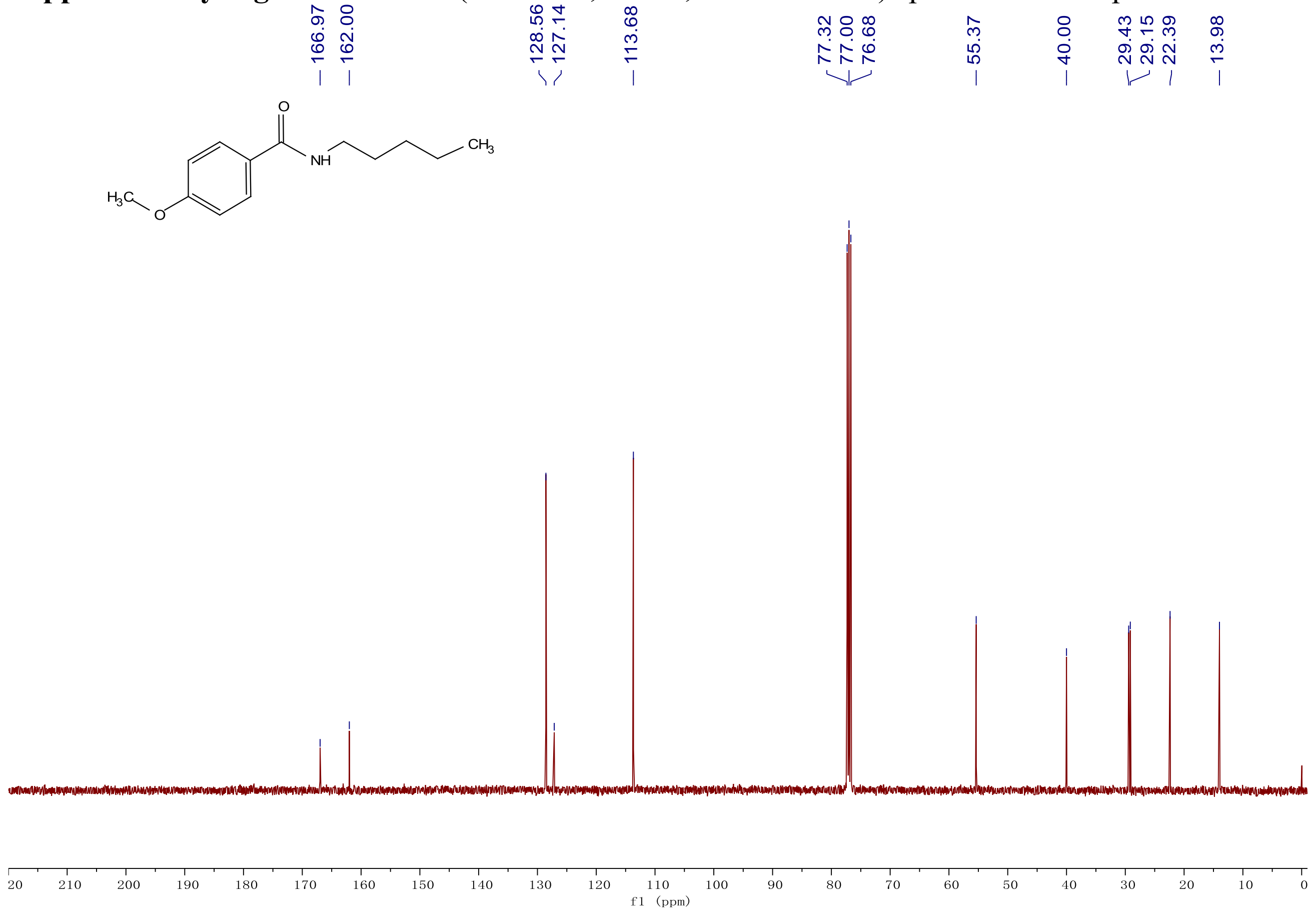

Supplementary Fig. 69. ^{13}C NMR (101 MHz, 298 K, Chloroform-*d*) spectrum of compound **1u**.

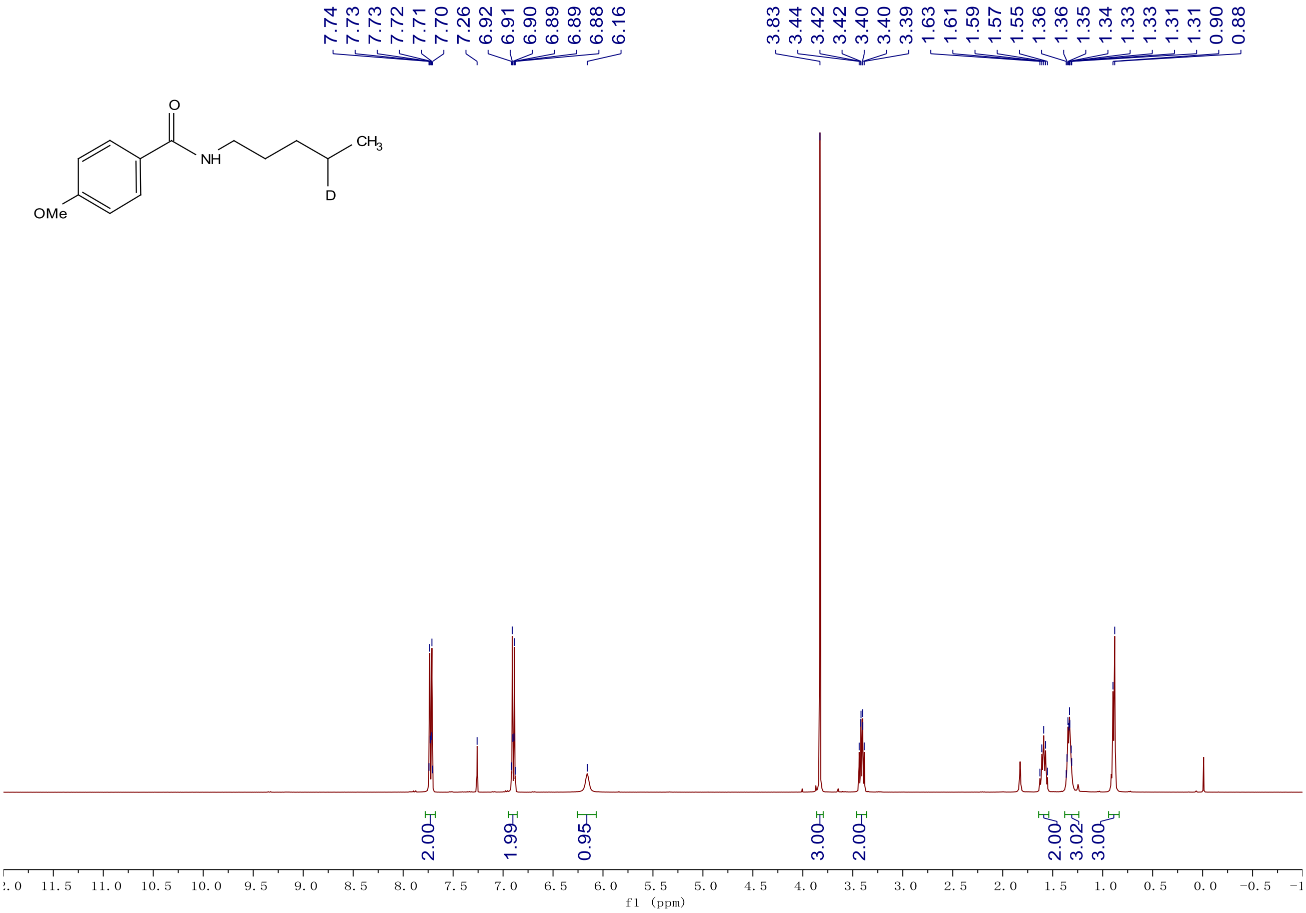

Supplementary Fig. 70. ^{1}H NMR (400 MHz, 298 K, Chloroform-*d*) spectrum of compound **3u**.

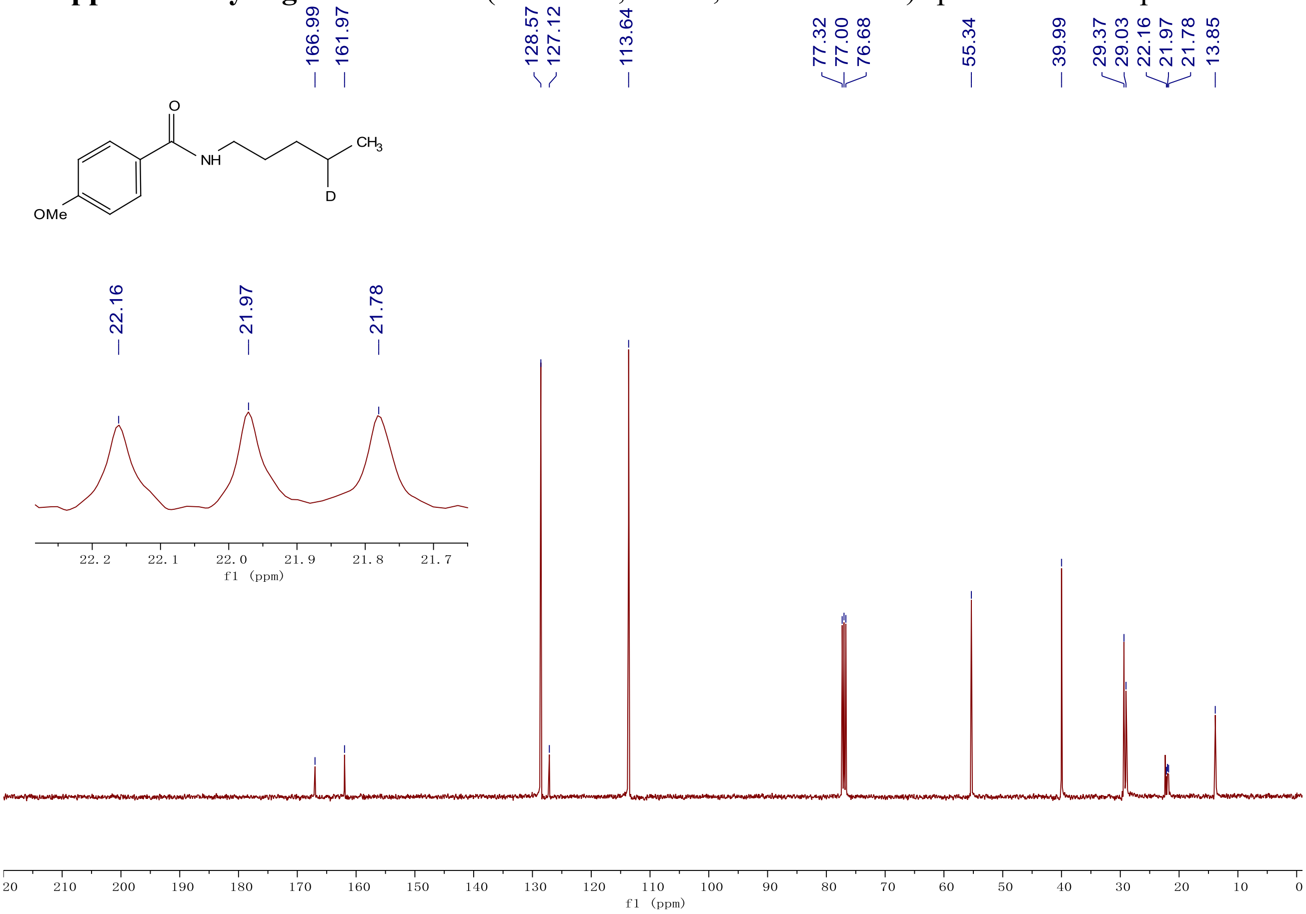

Supplementary Fig. 71. ^{13}C NMR (101 MHz, 298 K, Chloroform-*d*) spectrum of compound **3u**.

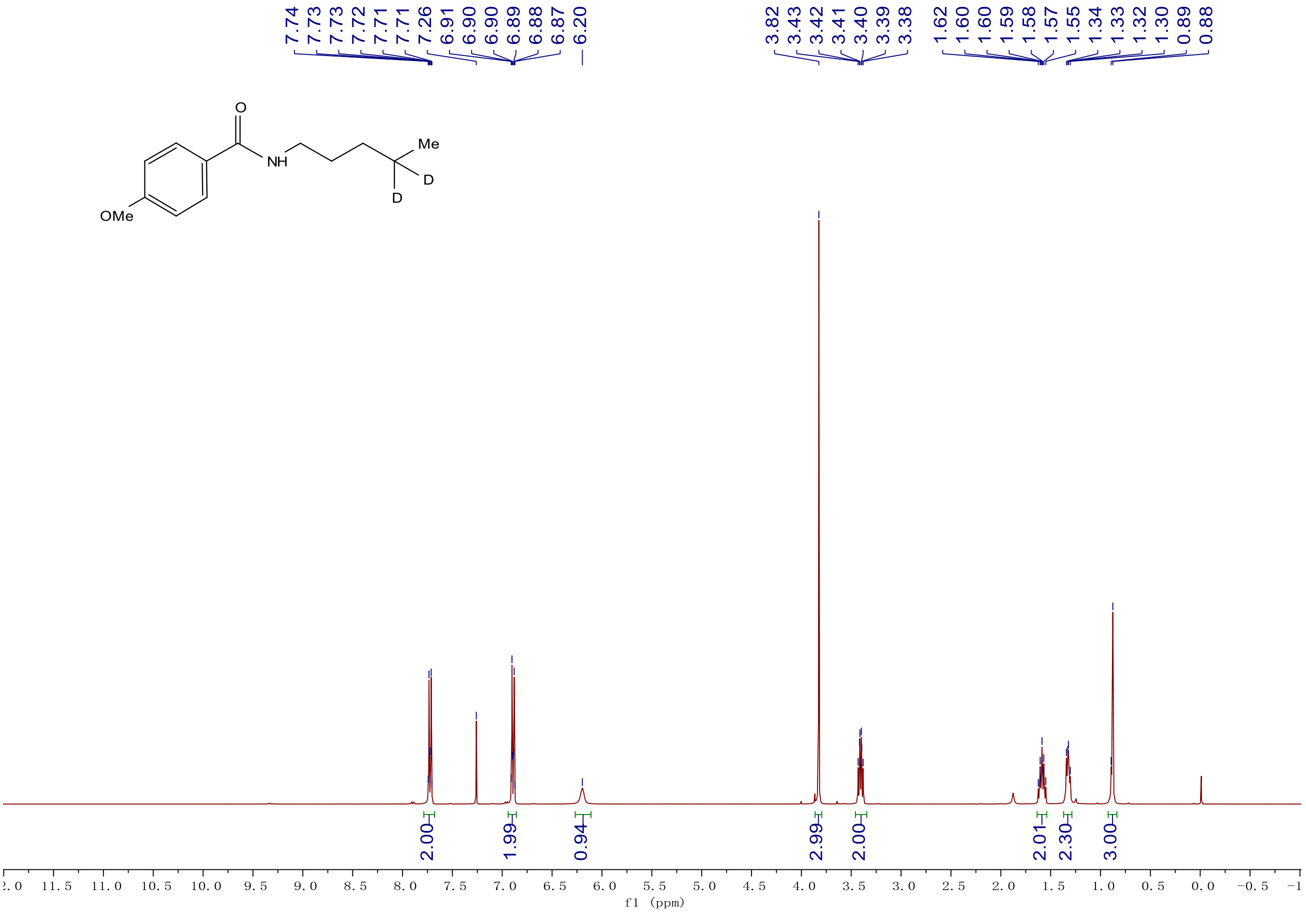

Supplementary Fig. 72. ^{1}H NMR (400 MHz, 298 K, Chloroform-*d*) spectrum of compound **3u'**.

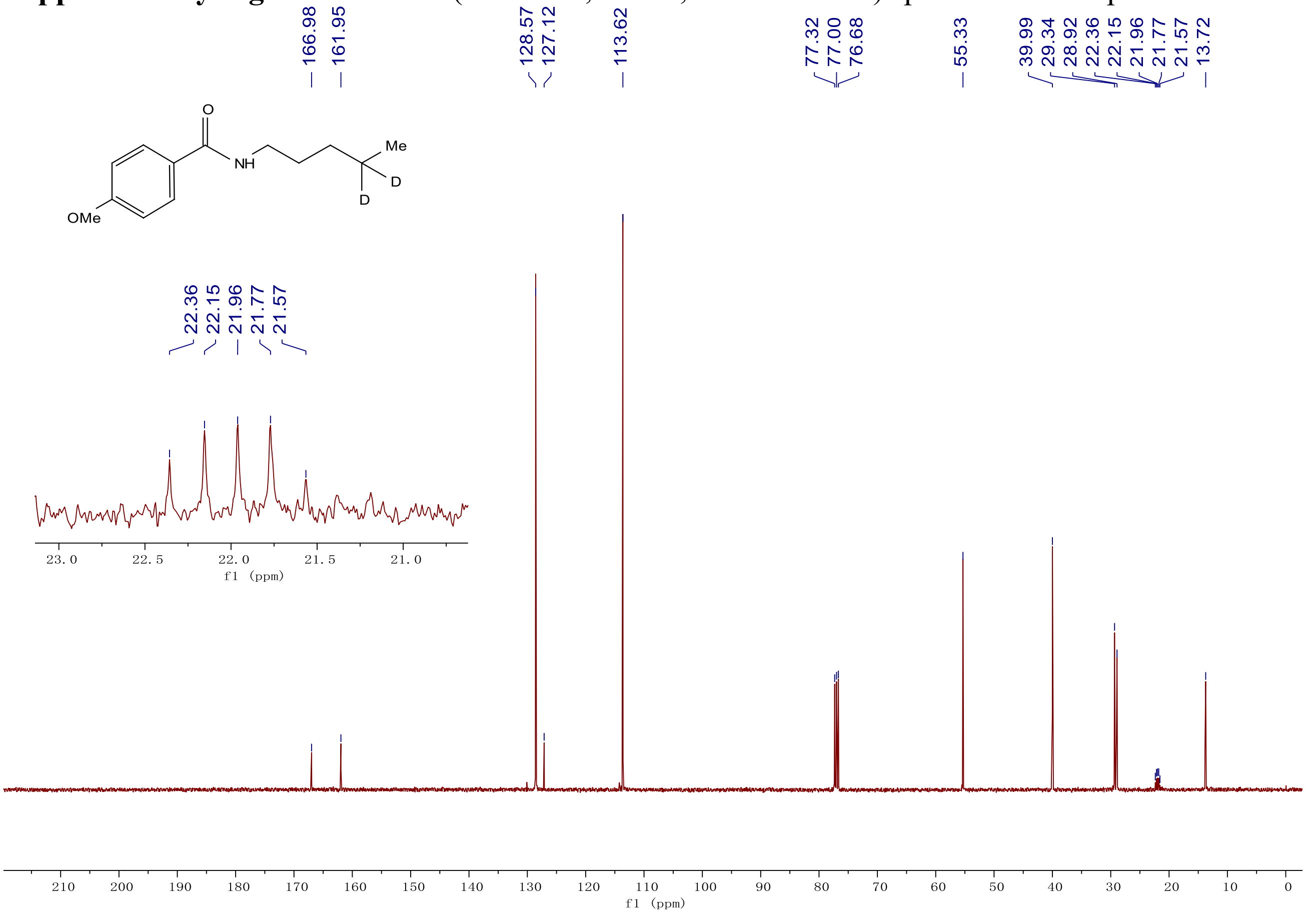

Supplementary Fig. 73. ^{13}C NMR (101 MHz, 298 K, Chloroform-*d*) spectrum of compound **3u'**.

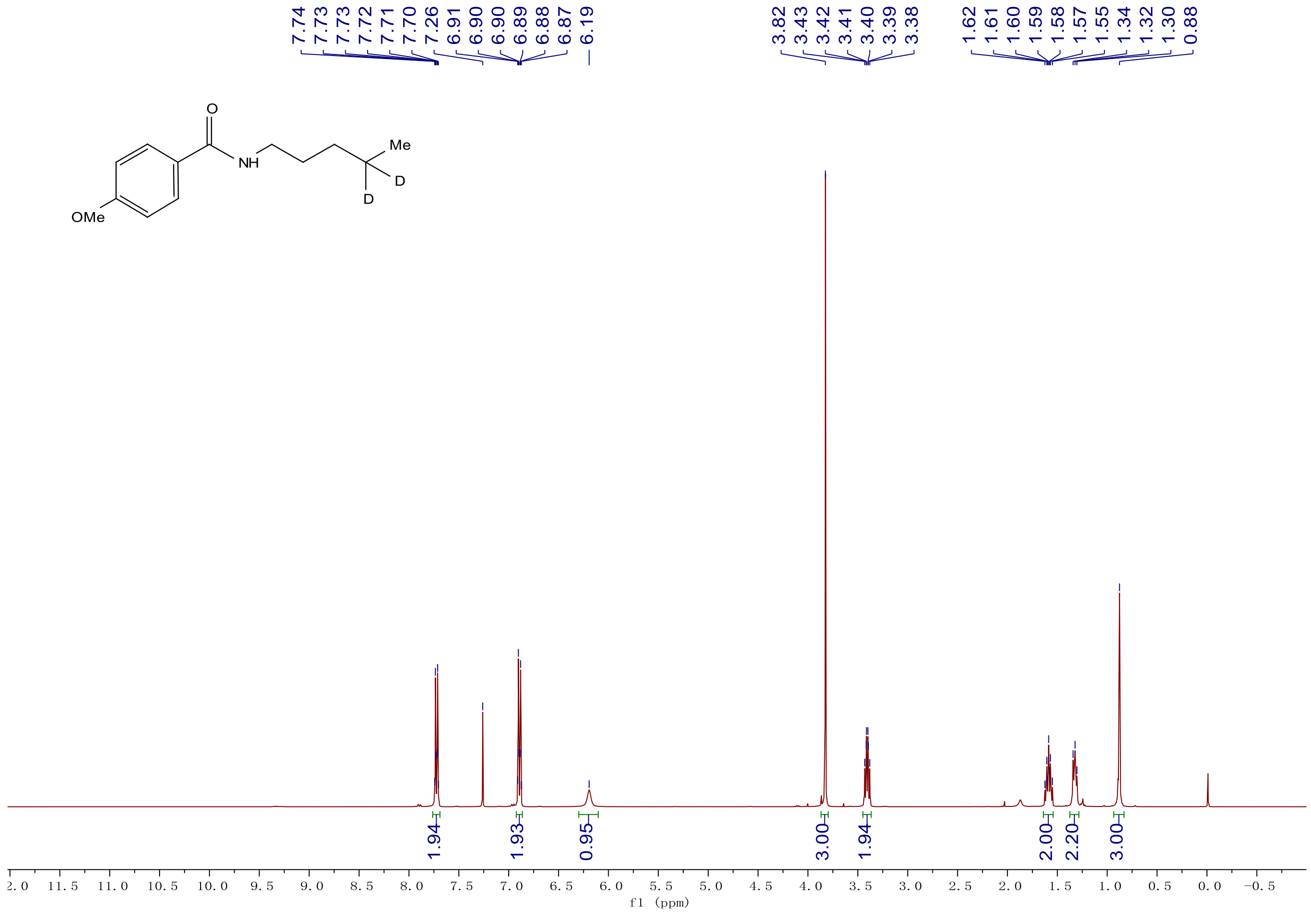

Supplementary Fig. 74. ^{1}H NMR (400 MHz, 298 K, Chloroform-*d*) spectrum of compound **3u''**.

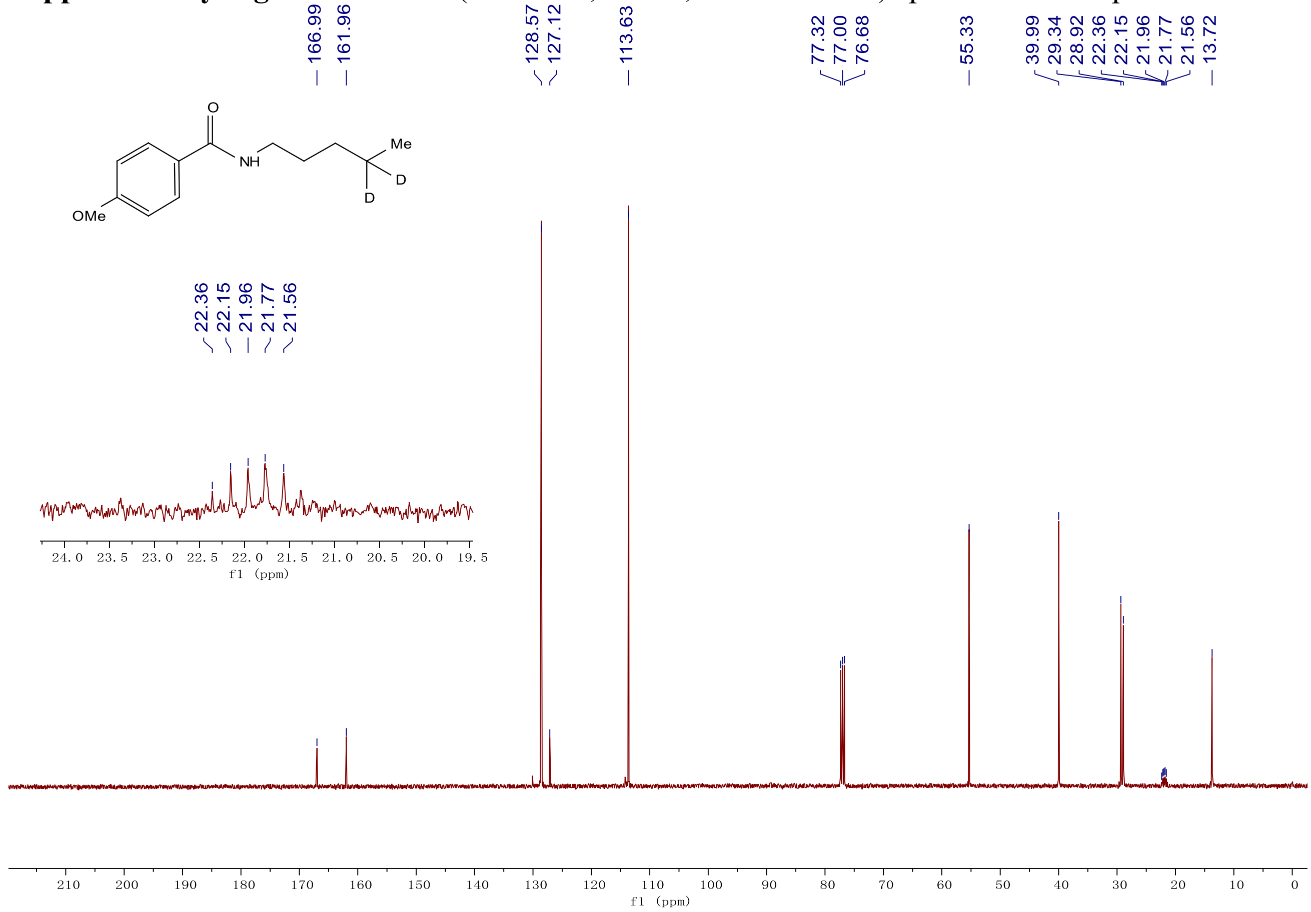

Supplementary Fig. 75. ^{13}C NMR (101 MHz, 298 K, Chloroform-*d*) spectrum of compound **3u''**.

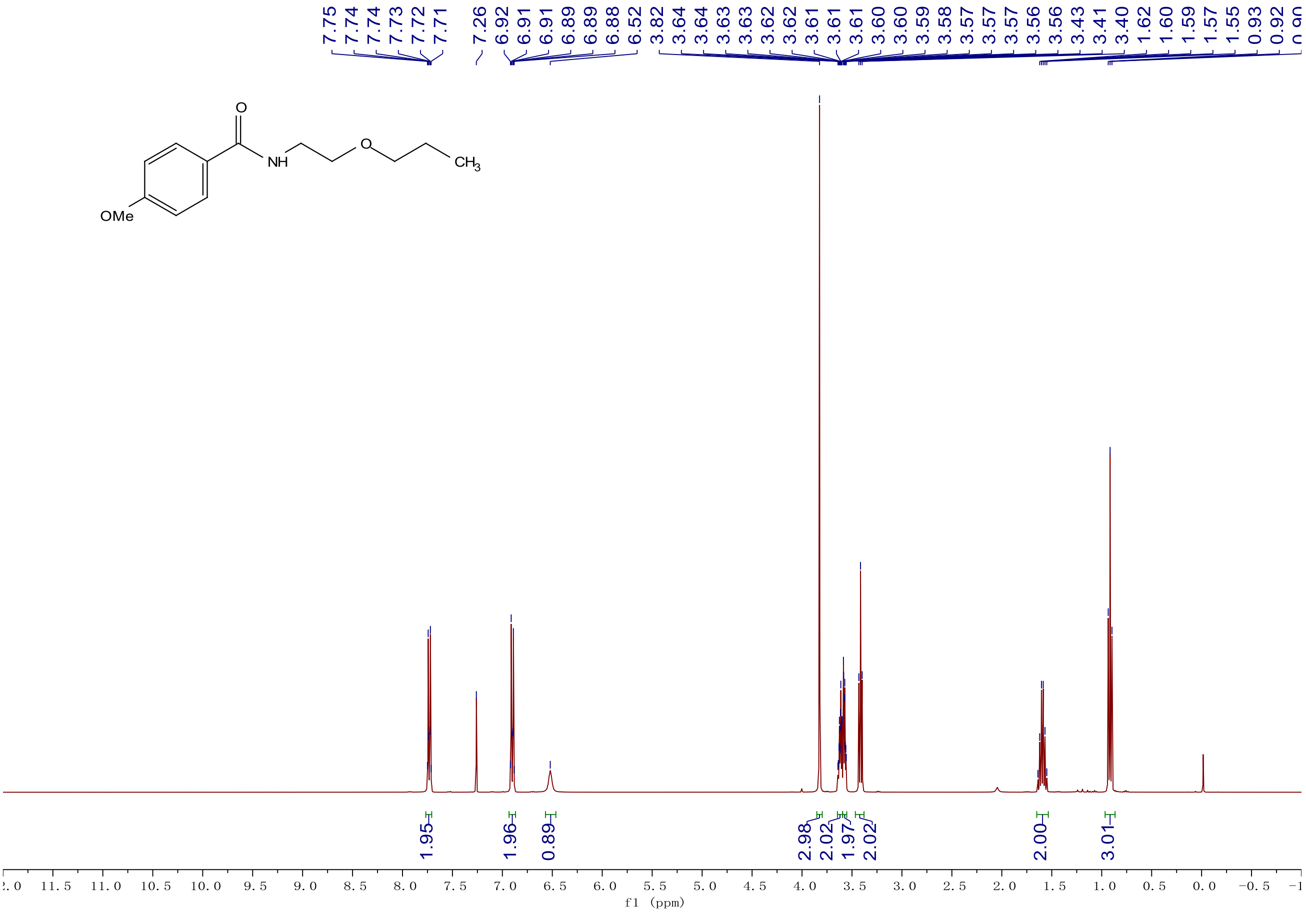

Supplementary Fig. 76. ^{1}H NMR (400 MHz, 298 K, Chloroform-*d*) spectrum of compound **1v**.

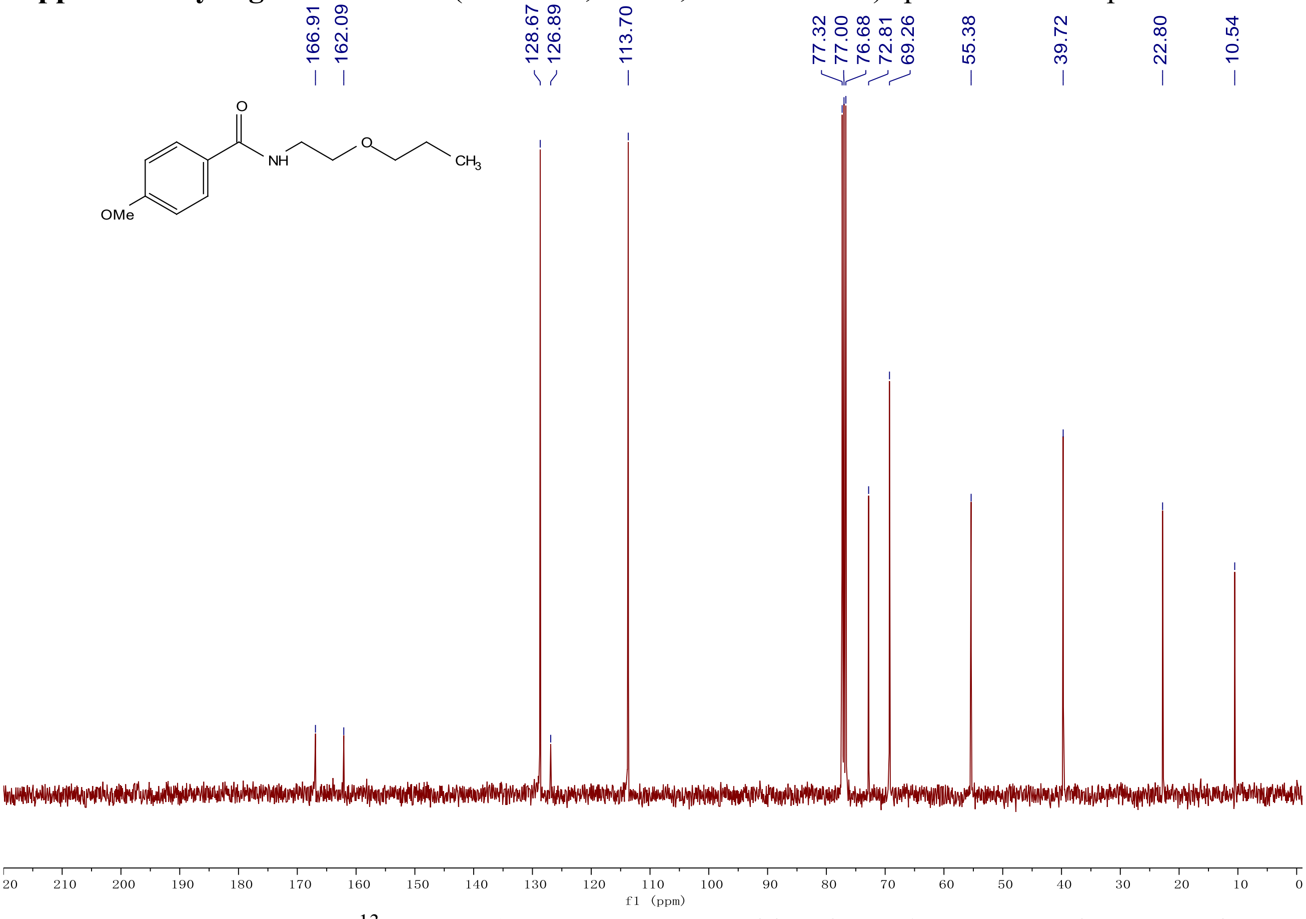

Supplementary Fig. 77. ^{13}C NMR (101 MHz, 298 K, Chloroform-*d*) spectrum of compound **1v**.

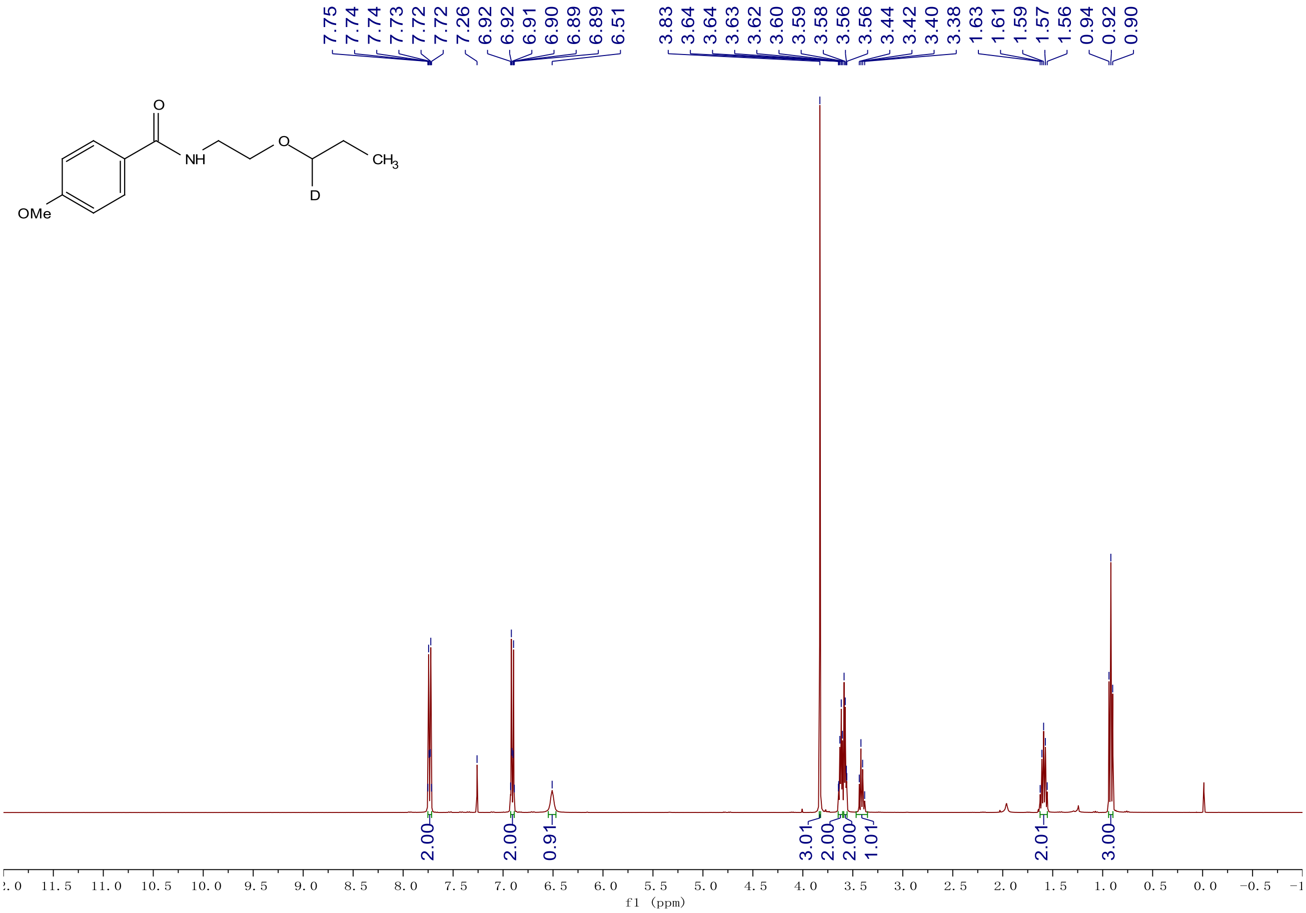

Supplementary Fig. 78. ^{1}H NMR (400 MHz, 298 K, Chloroform-*d*) spectrum of compound **3v**.

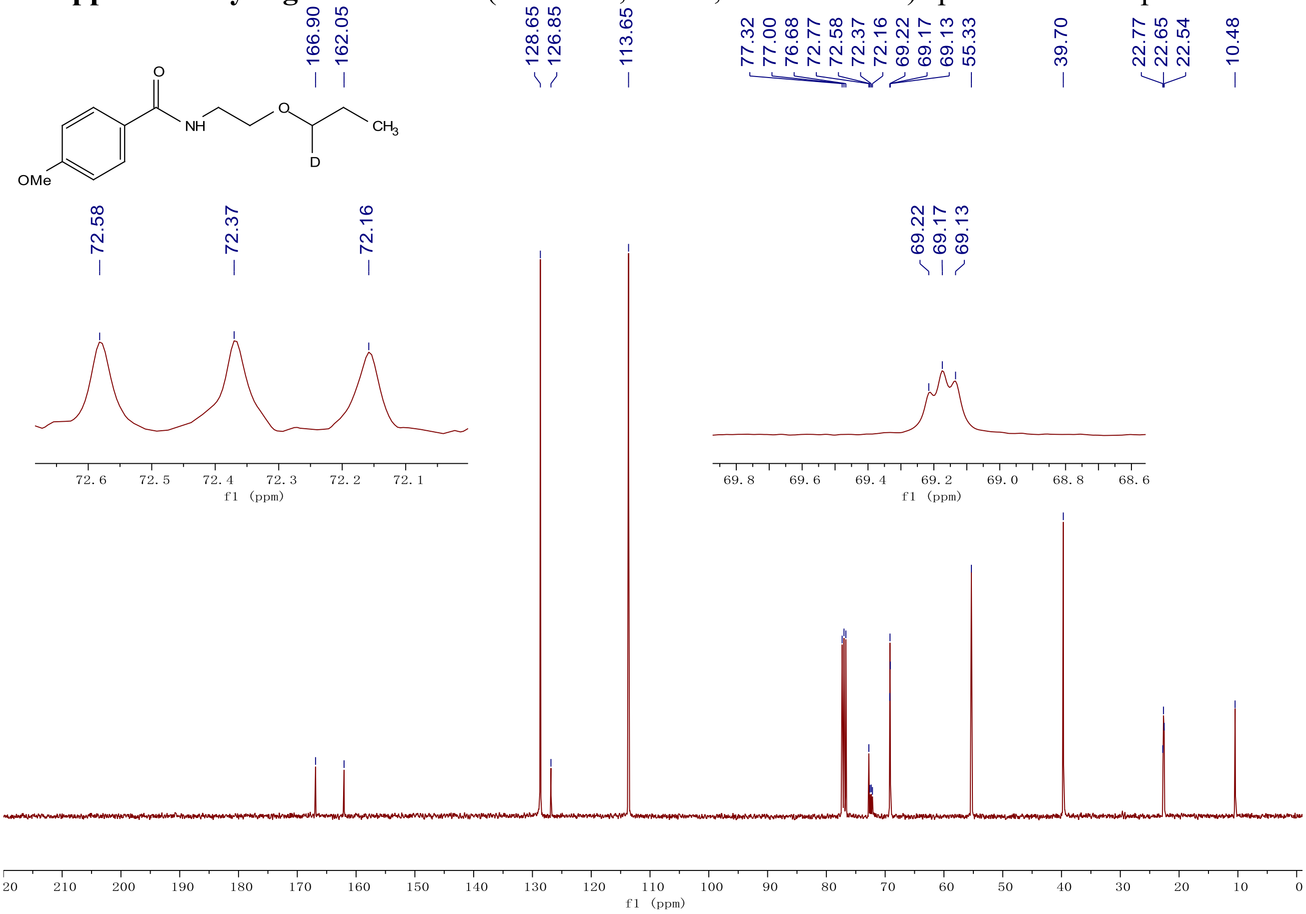

Supplementary Fig. 79. ^{13}C NMR (101 MHz, 298 K, Chloroform-*d*) spectrum of compound **3v**.

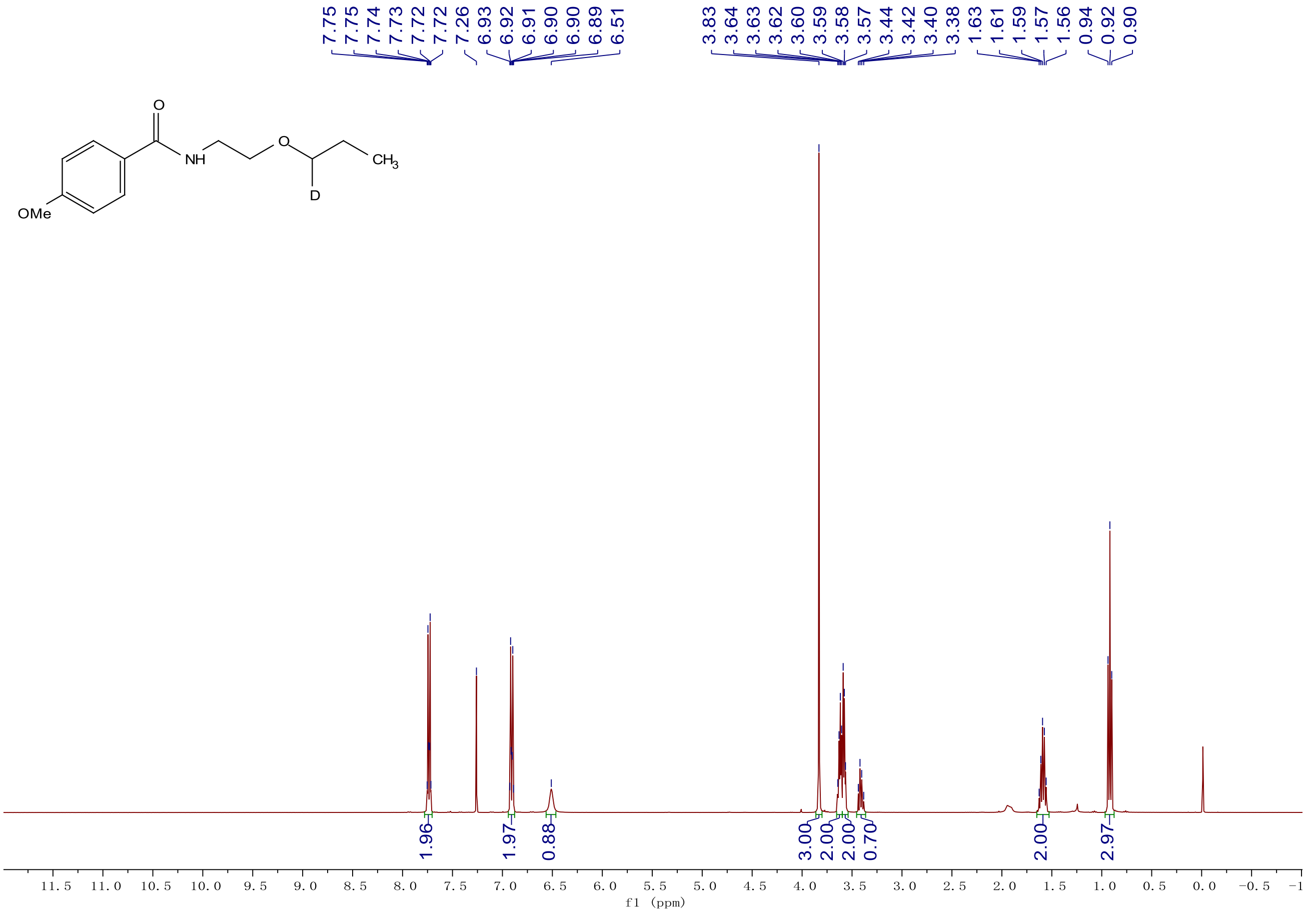

Supplementary Fig. 80. ^{1}H NMR (400 MHz, 298 K, Chloroform-*d*) spectrum of compound **3v'**.

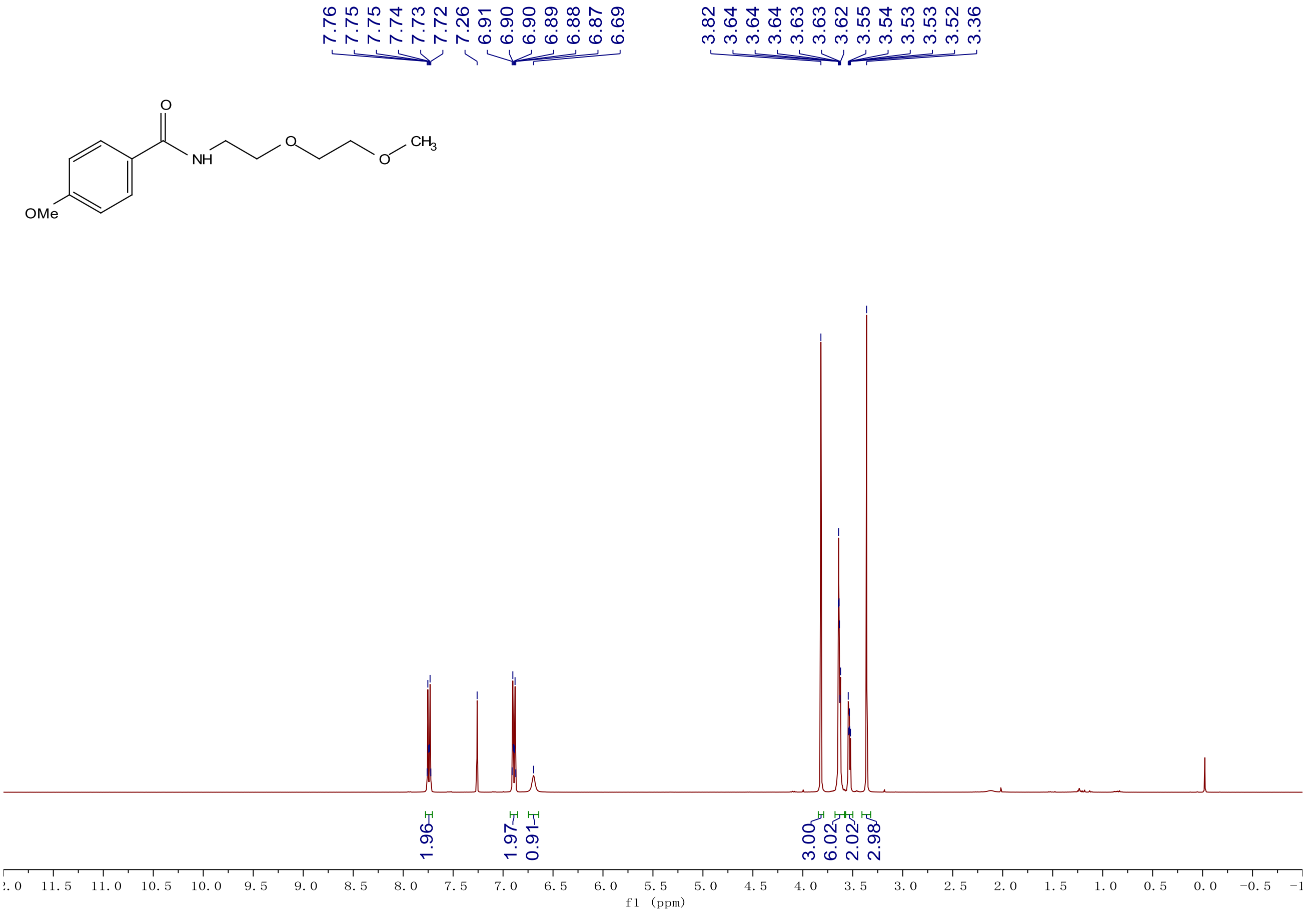

Supplementary Fig. 81. ^{1}H NMR (400 MHz, 298 K, Chloroform-*d*) spectrum of compound **1w**.

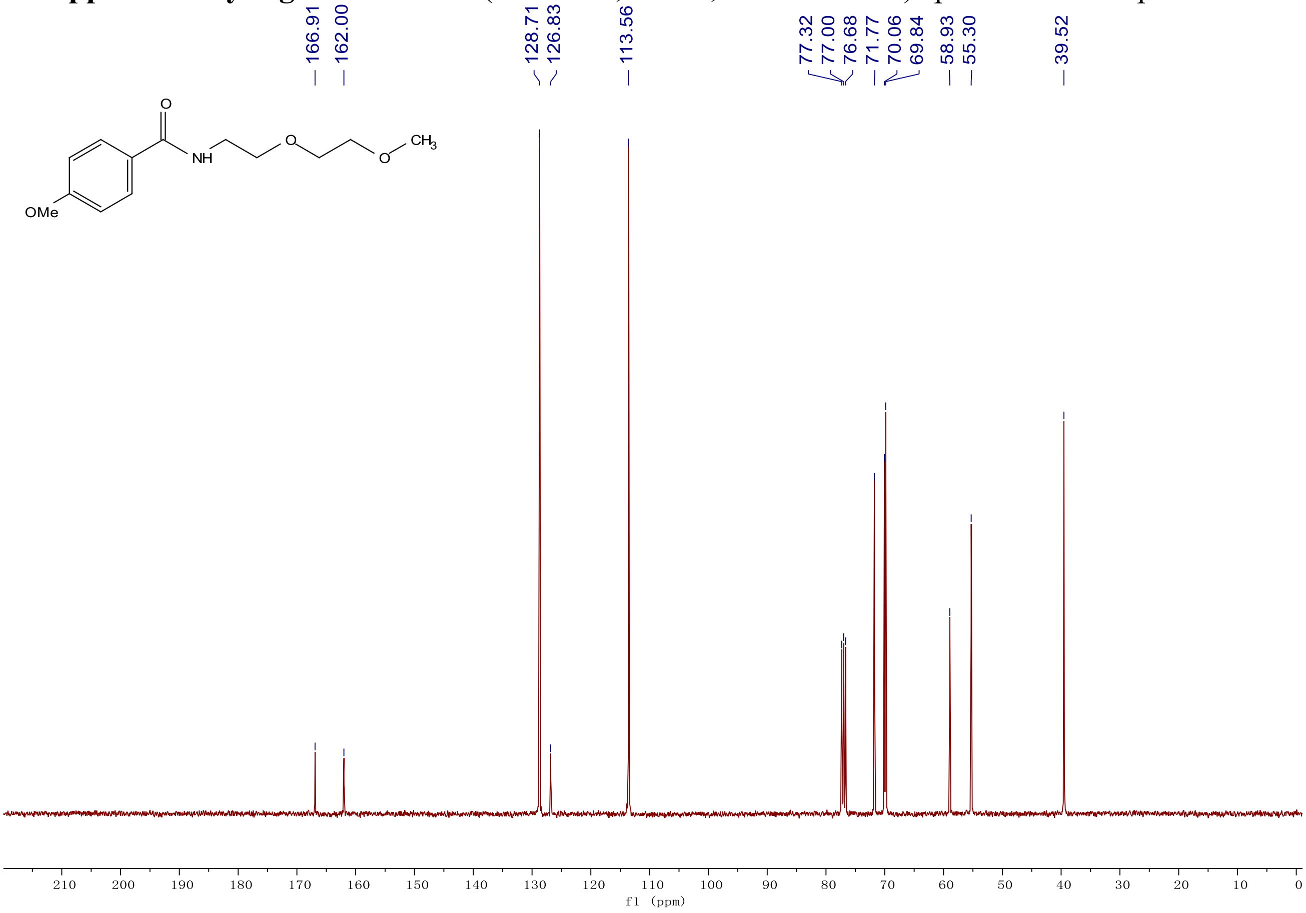

Supplementary Fig. 82. ^{13}C NMR (101 MHz, 298 K, Chloroform-*d*) spectrum of compound **1w**.

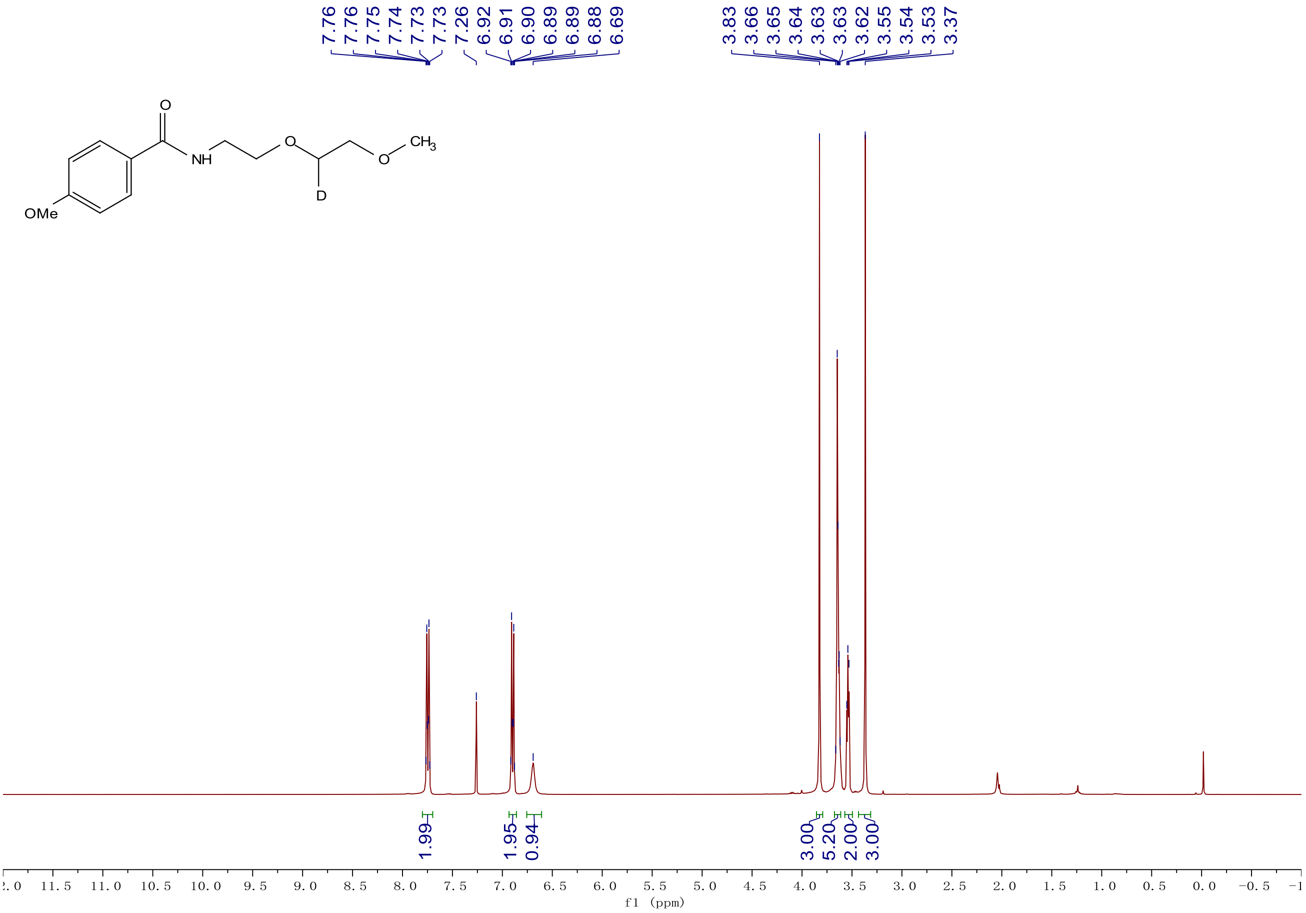

Supplementary Fig. 83. ^{1}H NMR (400 MHz, 298 K, Chloroform-*d*) spectrum of compound **3w**.

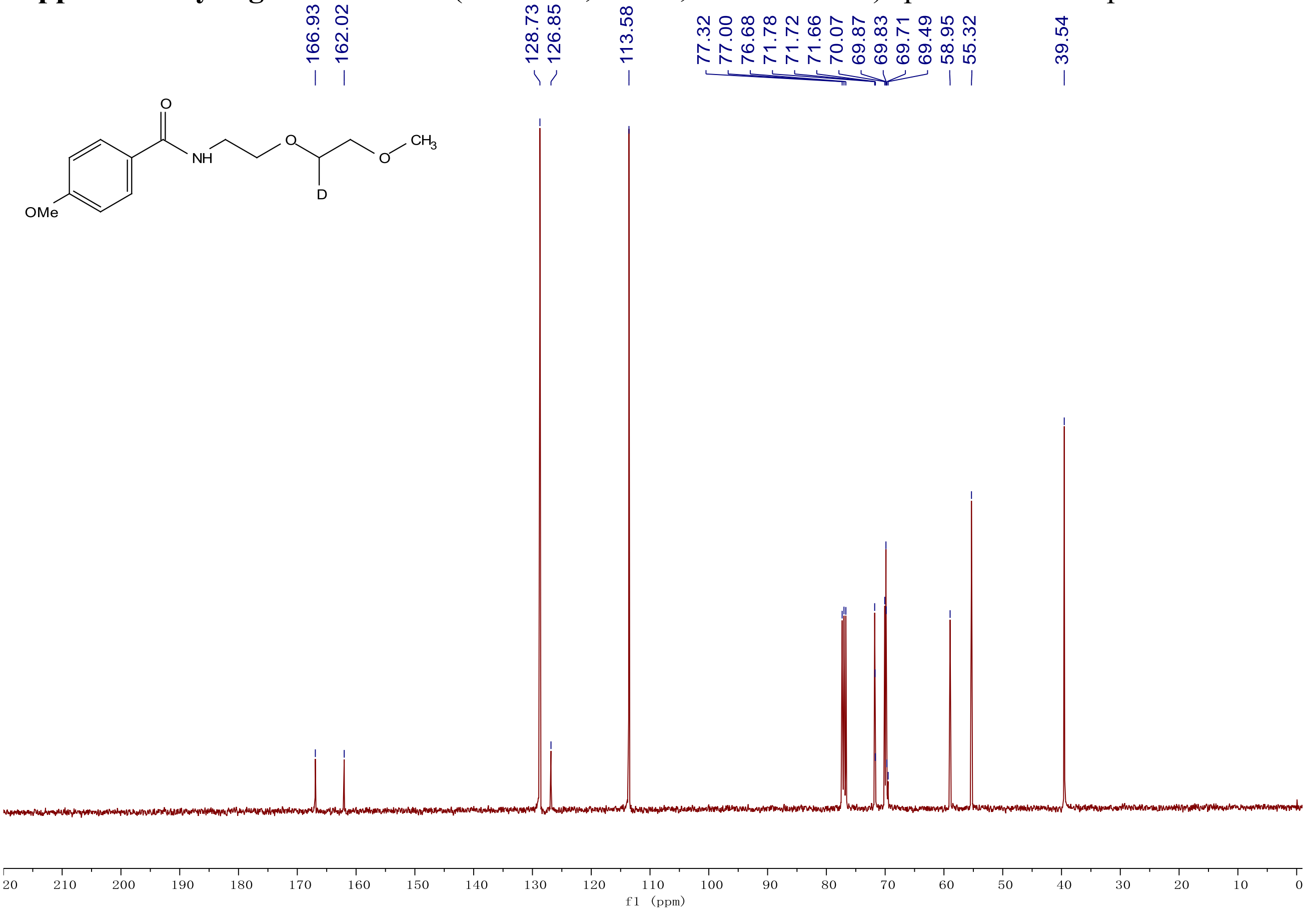

Supplementary Fig. 84. ^{13}C NMR (101 MHz, 298 K, Chloroform-*d*) spectrum of compound **3w**.

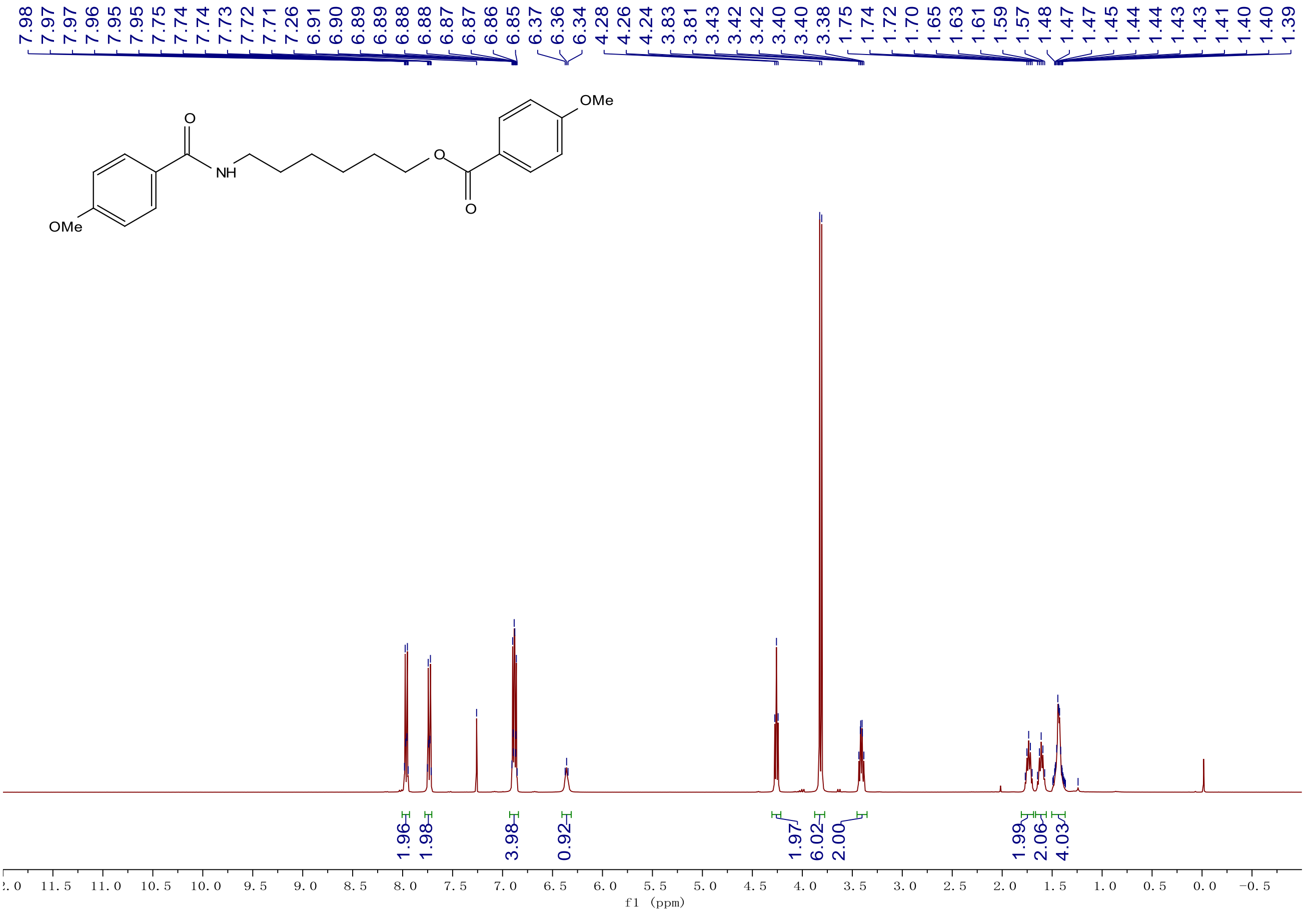

Supplementary Fig. 85. ^{1}H NMR (400 MHz, 298 K, Chloroform-*d*) spectrum of compound **1x**.

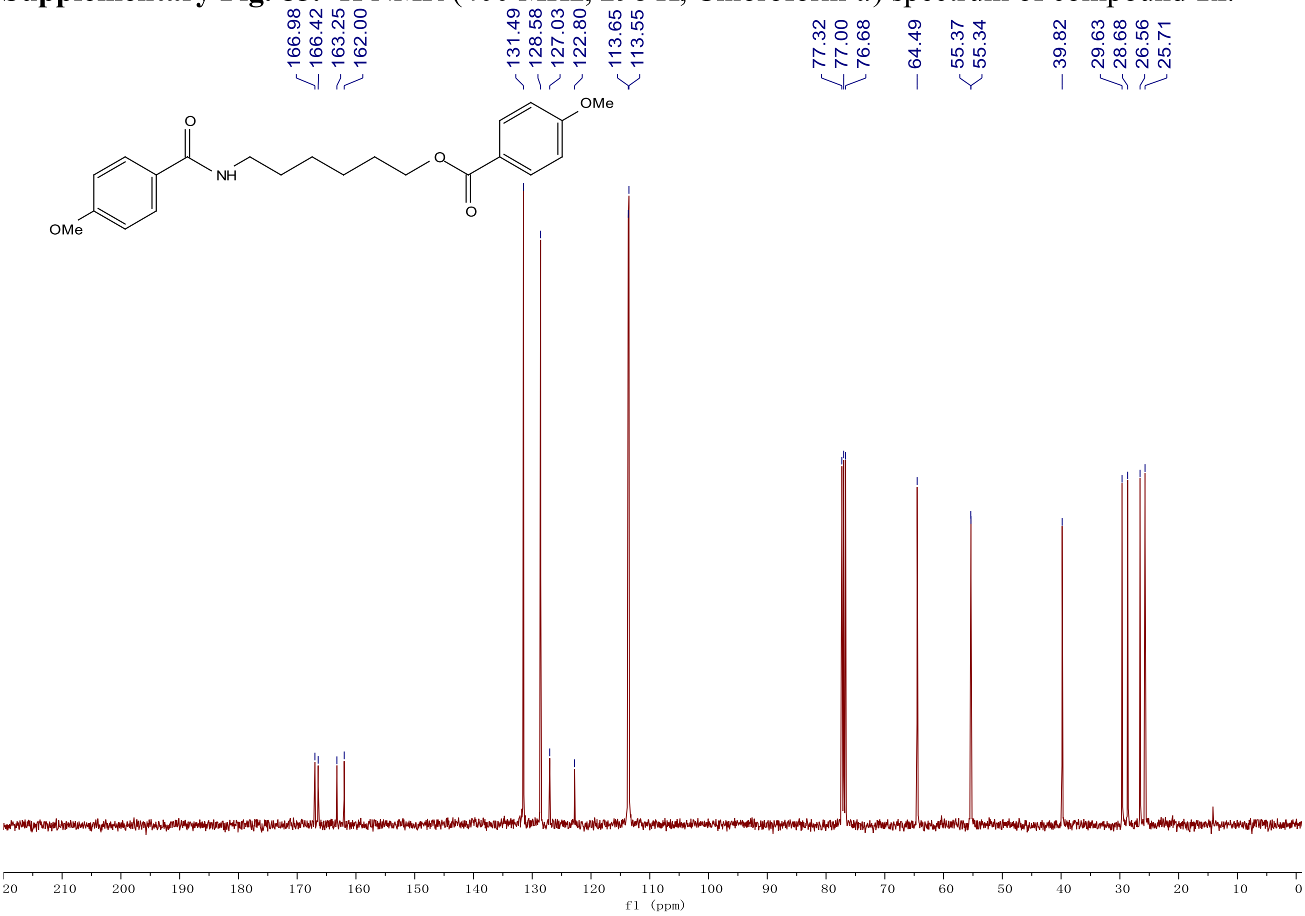

Supplementary Fig. 86. ^{13}C NMR (101 MHz, 298 K, Chloroform-*d*) spectrum of compound **1x**.

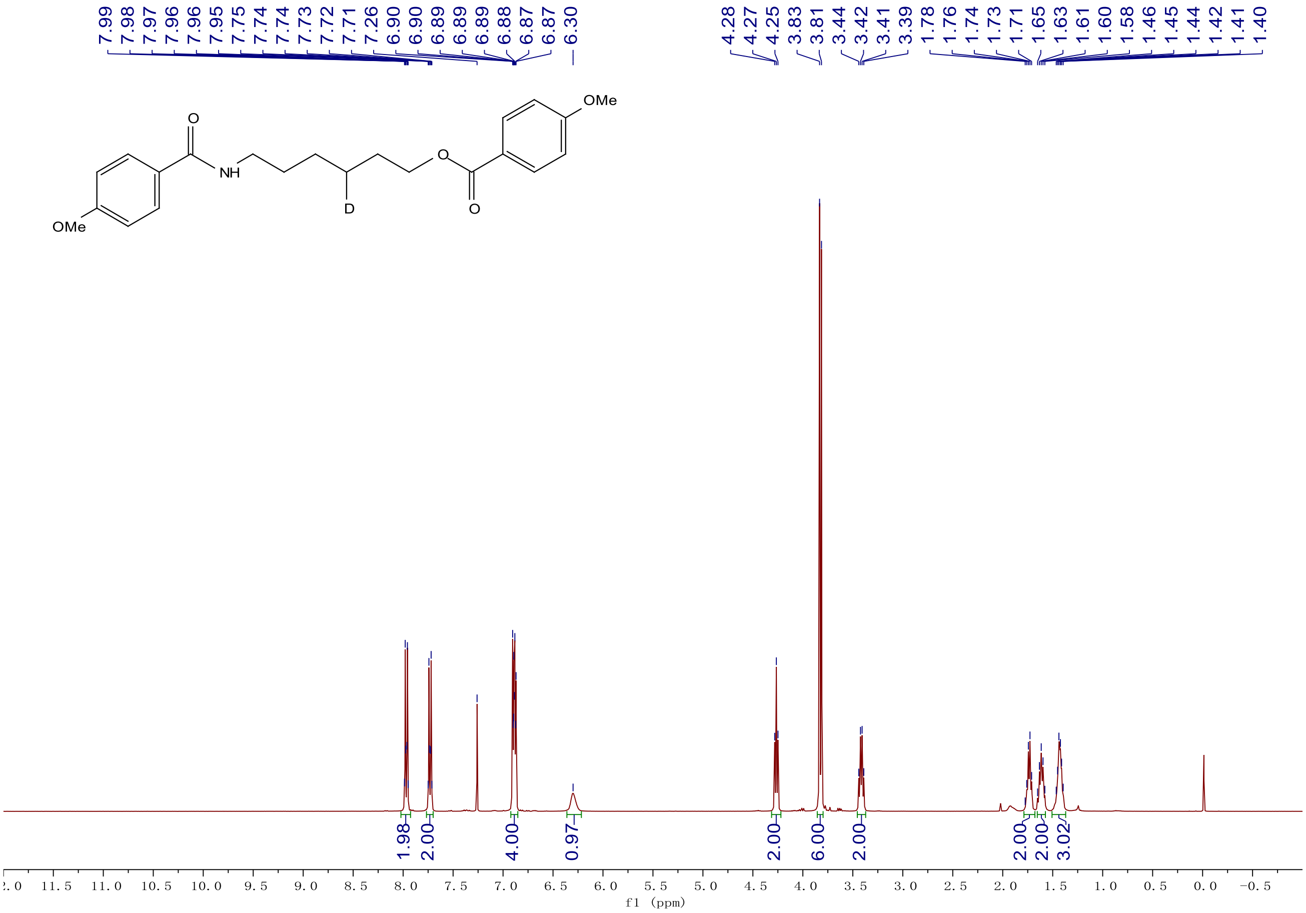

Supplementary Fig. 87. ^{1}H NMR (400 MHz, 298 K, Chloroform-*d*) spectrum of compound **3x**.

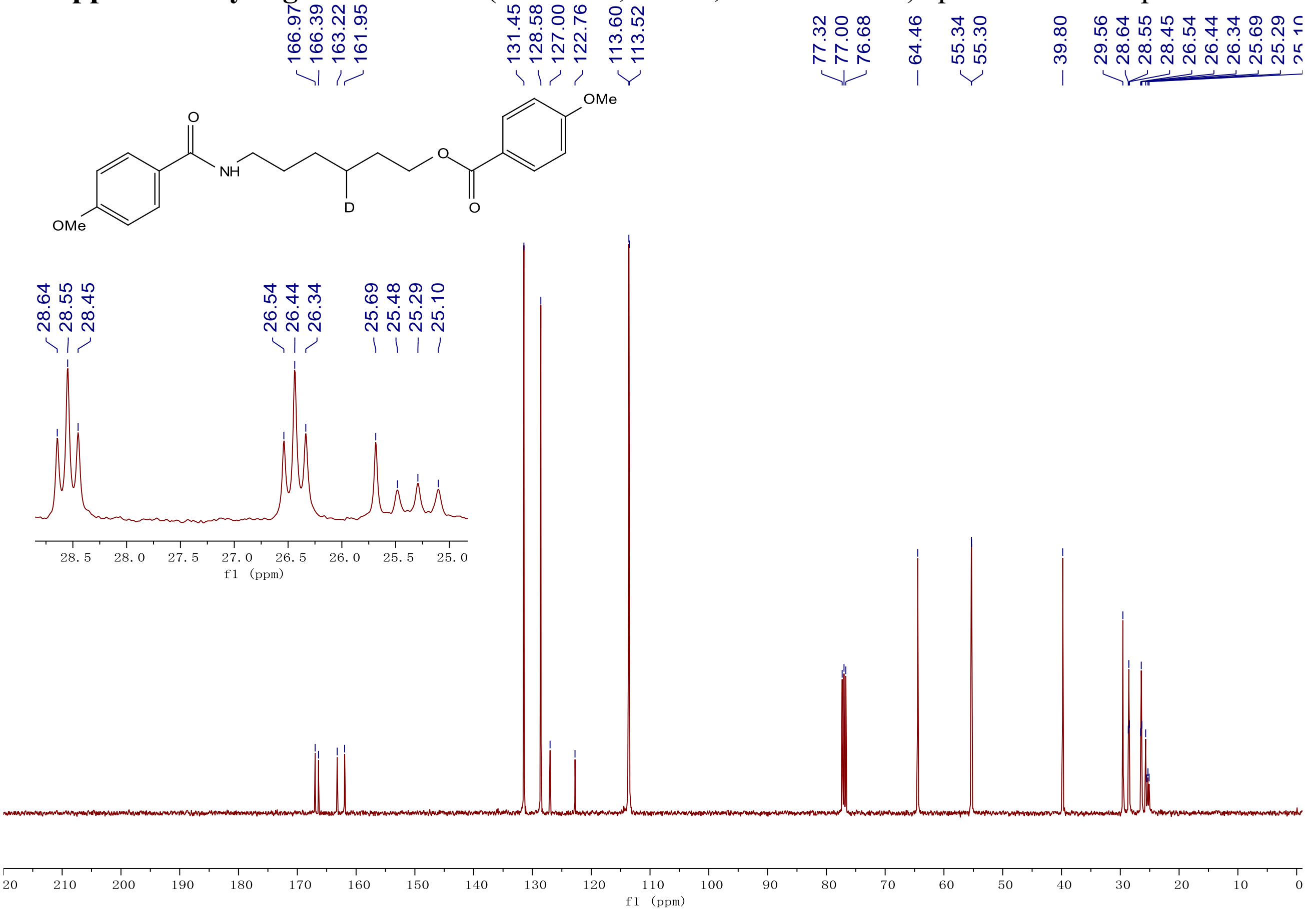

Supplementary Fig. 88. ^{13}C NMR (101 MHz, 298 K, Chloroform-*d*) spectrum of compound **3x**.

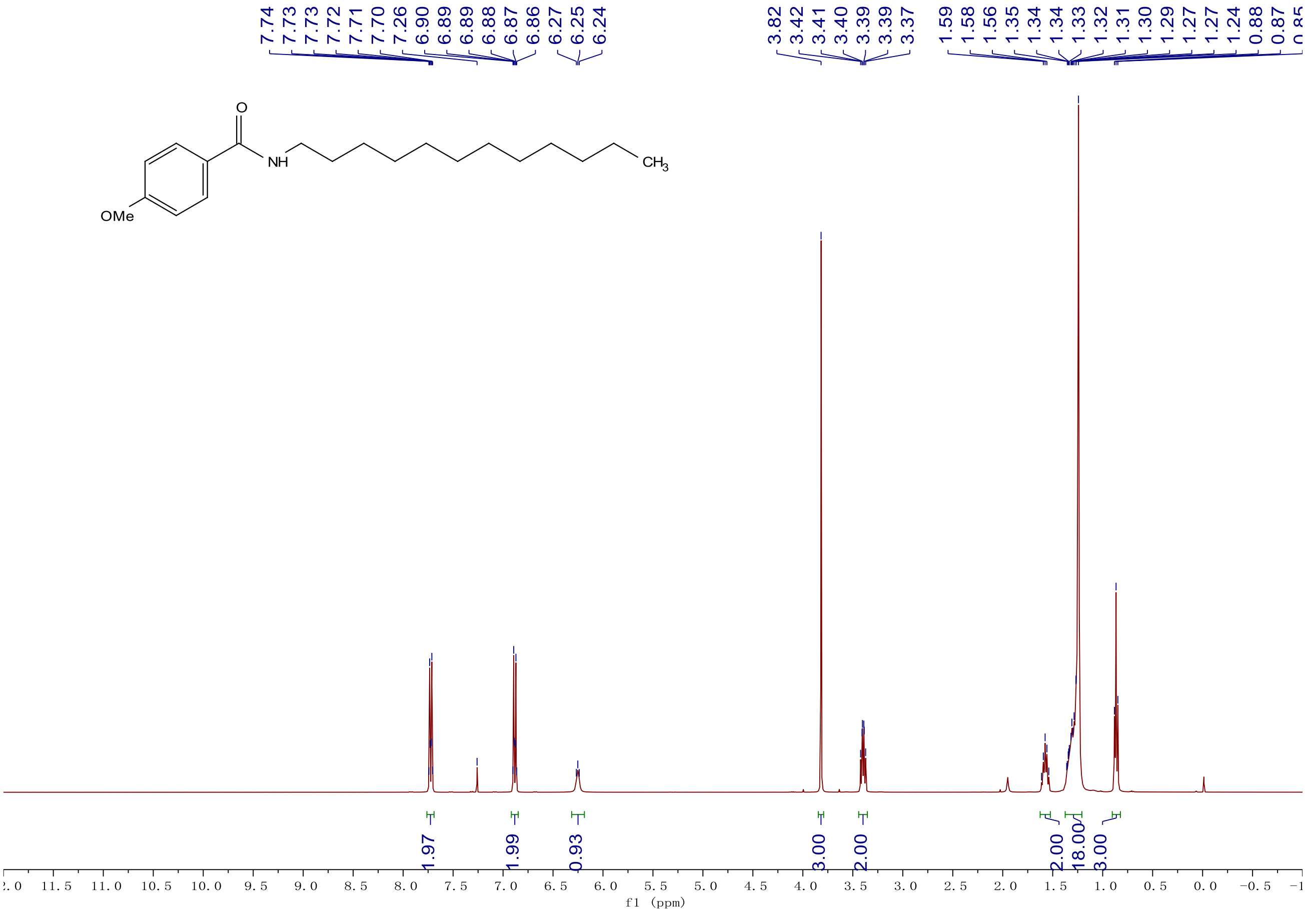

Supplementary Fig. 89. ^{1}H NMR (400 MHz, 298 K, Chloroform-*d*) spectrum of compound **1y**.

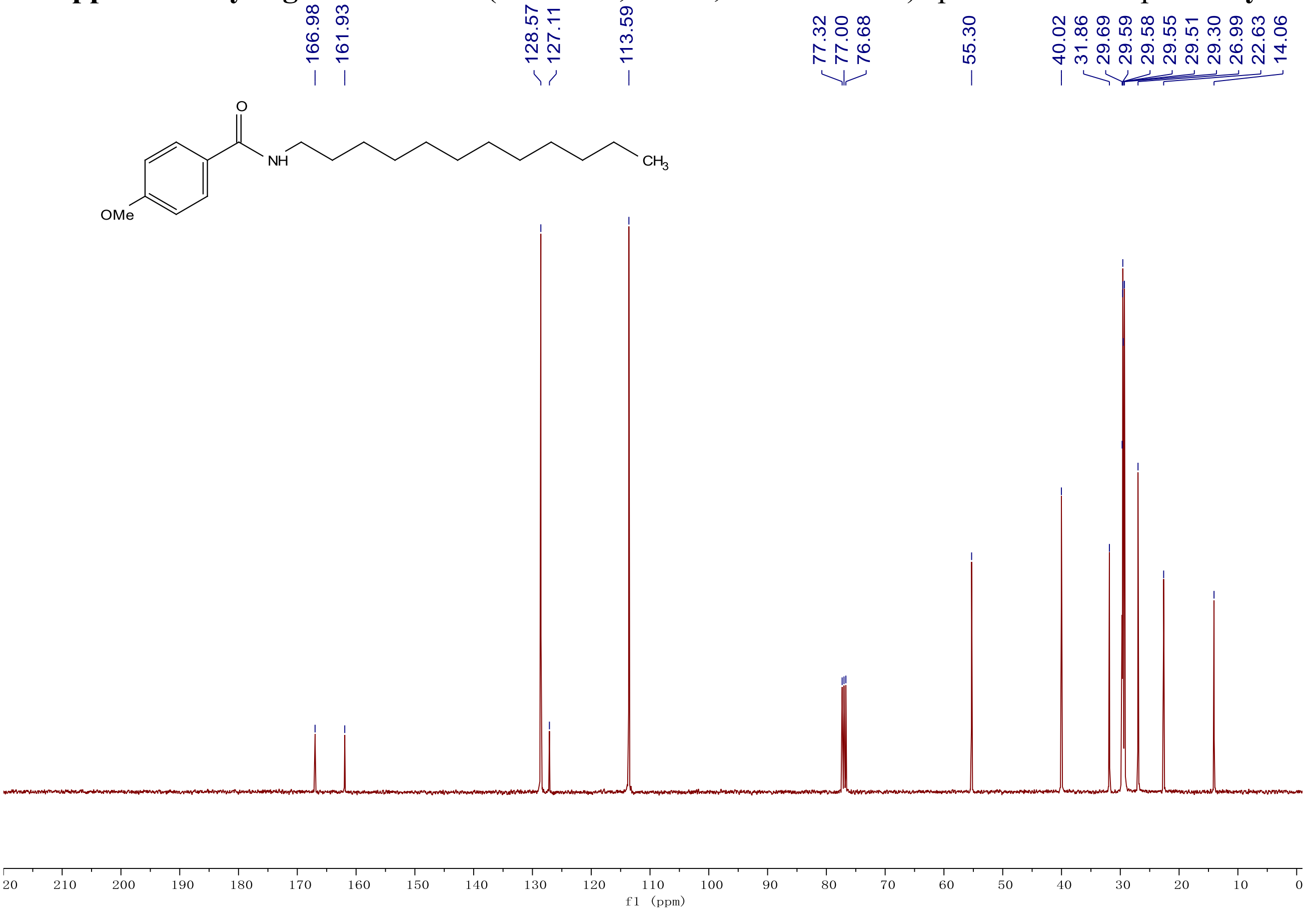

Supplementary Fig. 90. ^{13}C NMR (101 MHz, 298 K, Chloroform-*d*) spectrum of compound **1y**.

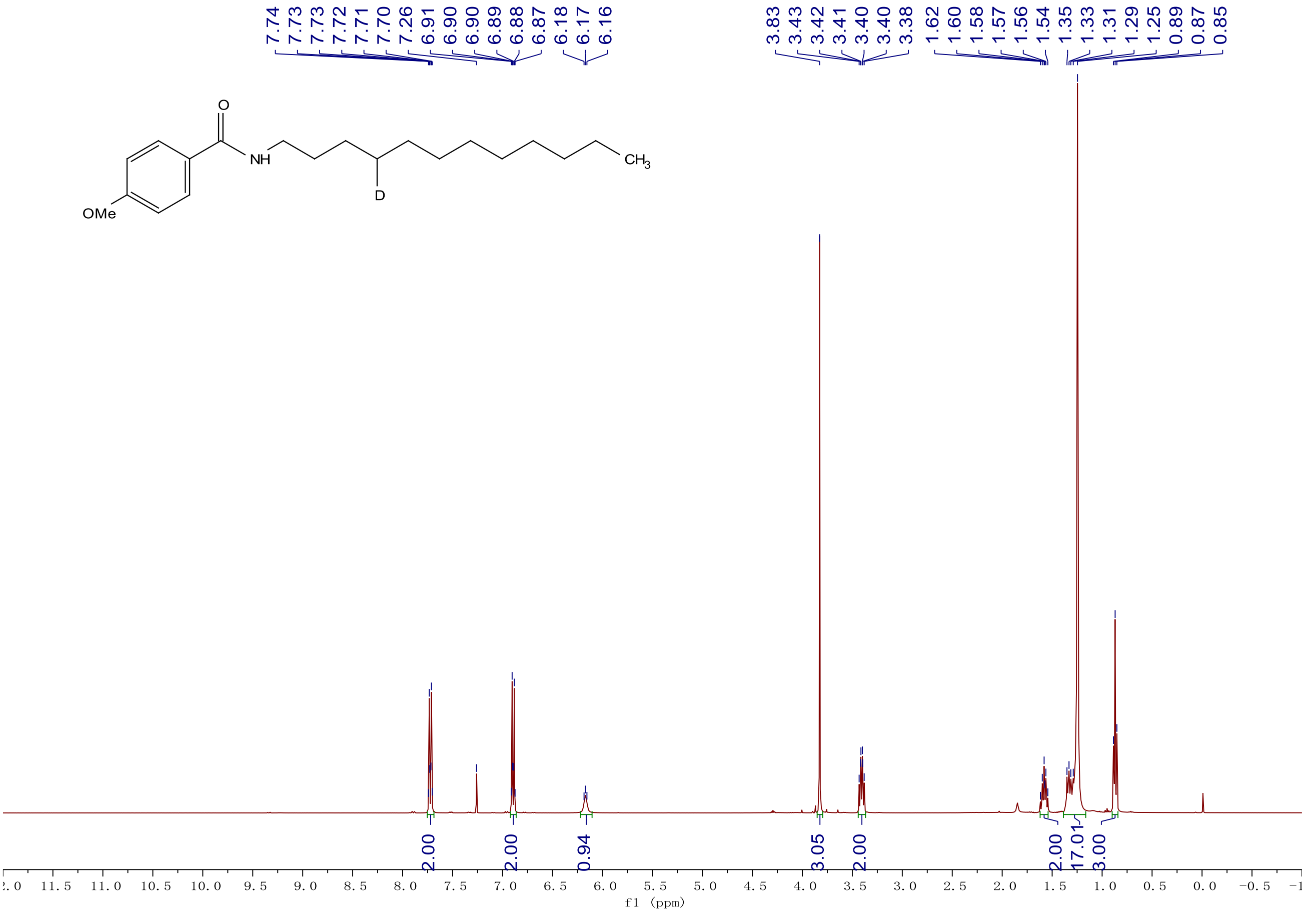

Supplementary Fig. 91. ^{1}H NMR (400 MHz, 298 K, Chloroform-*d*) spectrum of compound **3y**.

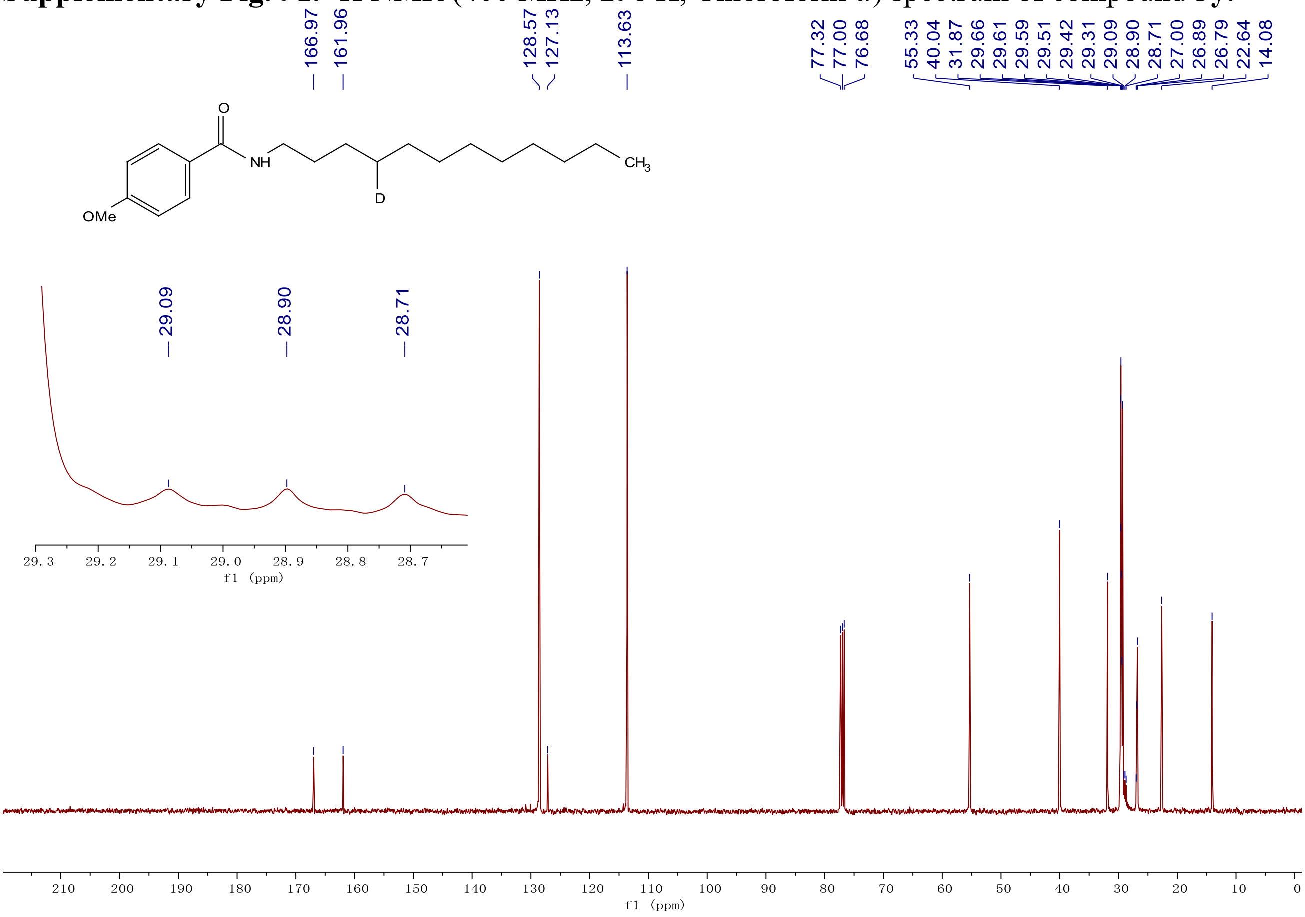

Supplementary Fig. 92. ^{13}C NMR (101 MHz, 298 K, Chloroform-*d*) spectrum of compound **3y**.

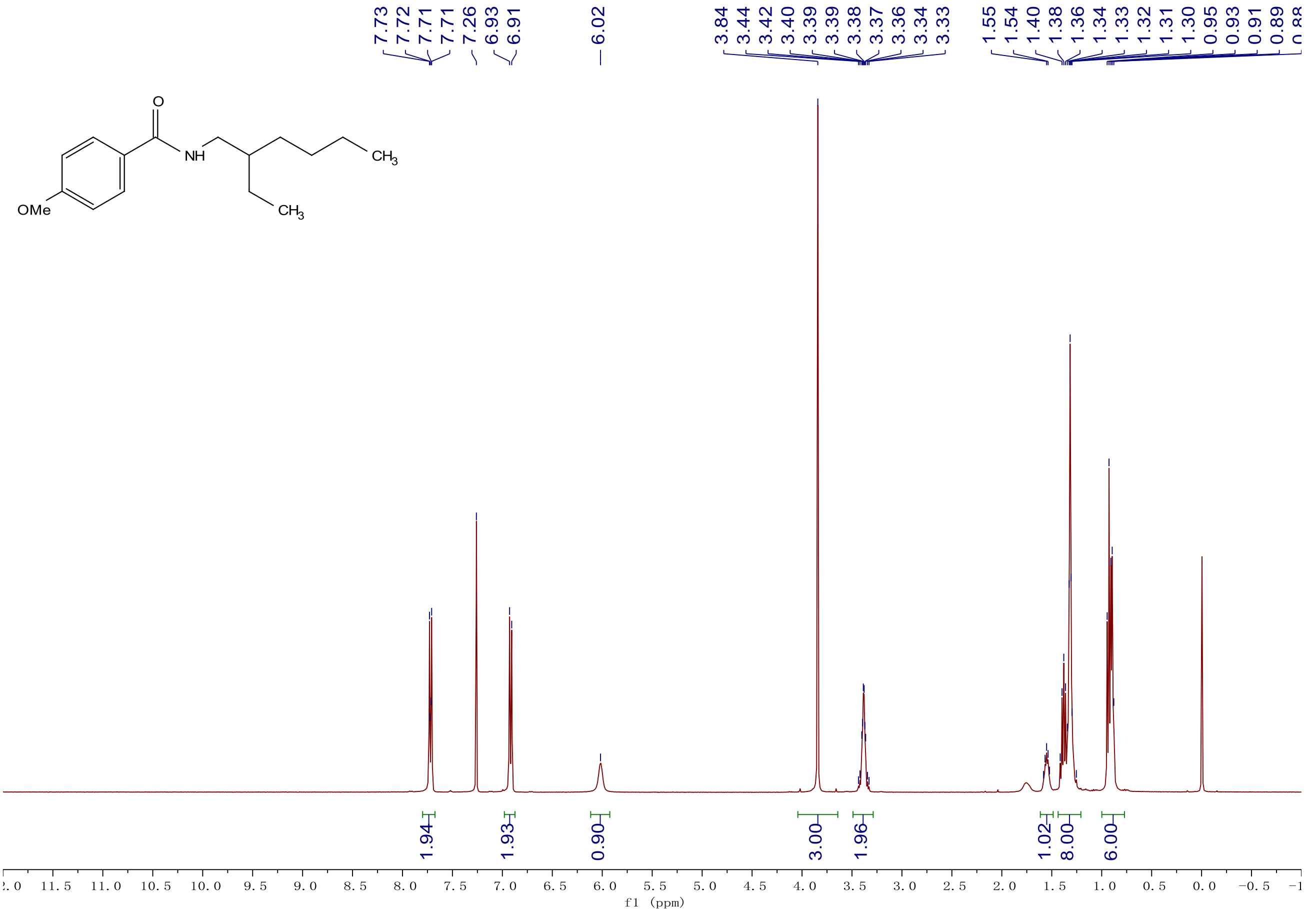

Supplementary Fig. 93. ^{1}H NMR (400 MHz, 298 K, Chloroform-*d*) spectrum of compound **1z**.

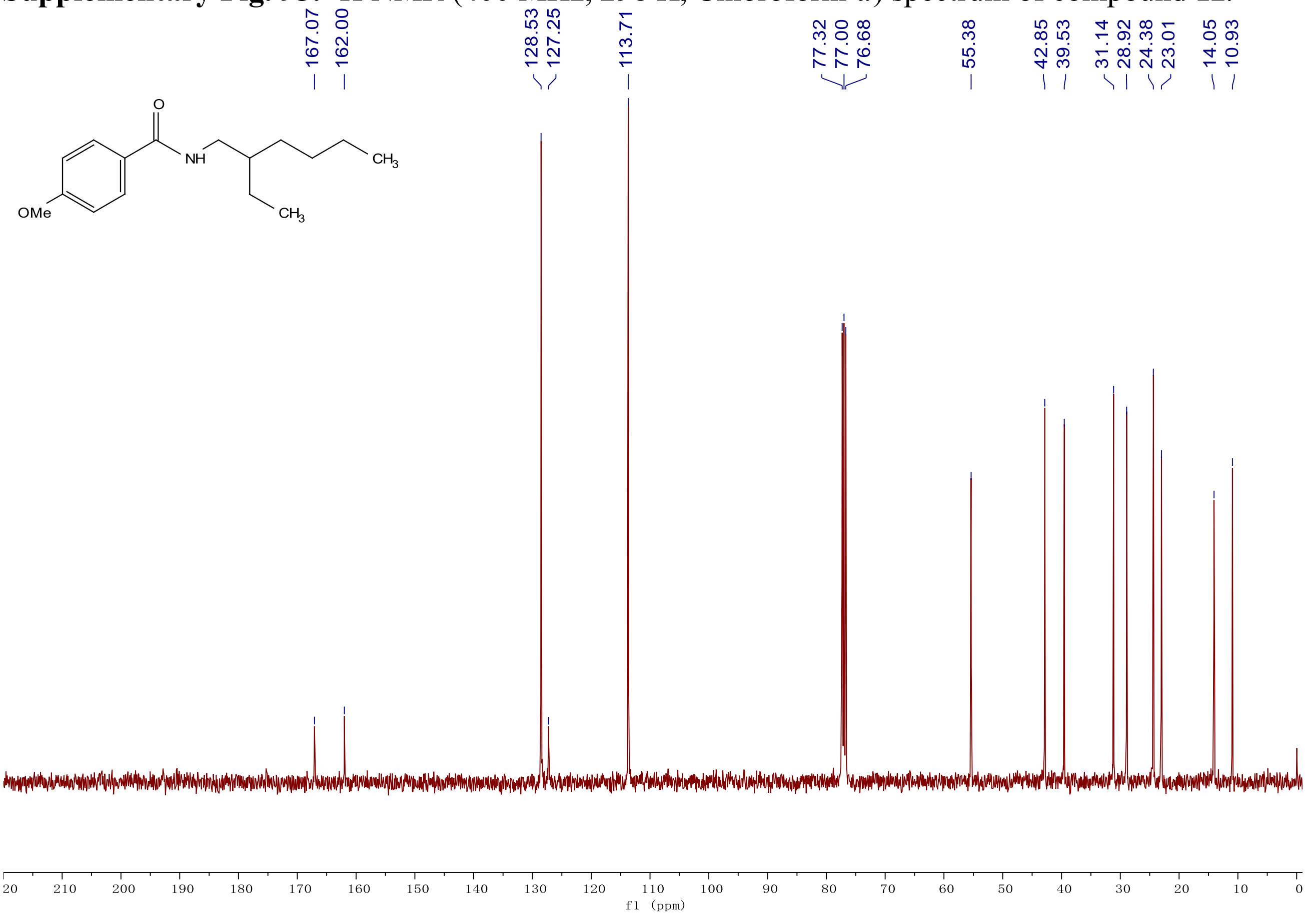

Supplementary Fig. 94. ^{13}C NMR (101 MHz, 298 K, Chloroform-*d*) spectrum of compound **1z**.

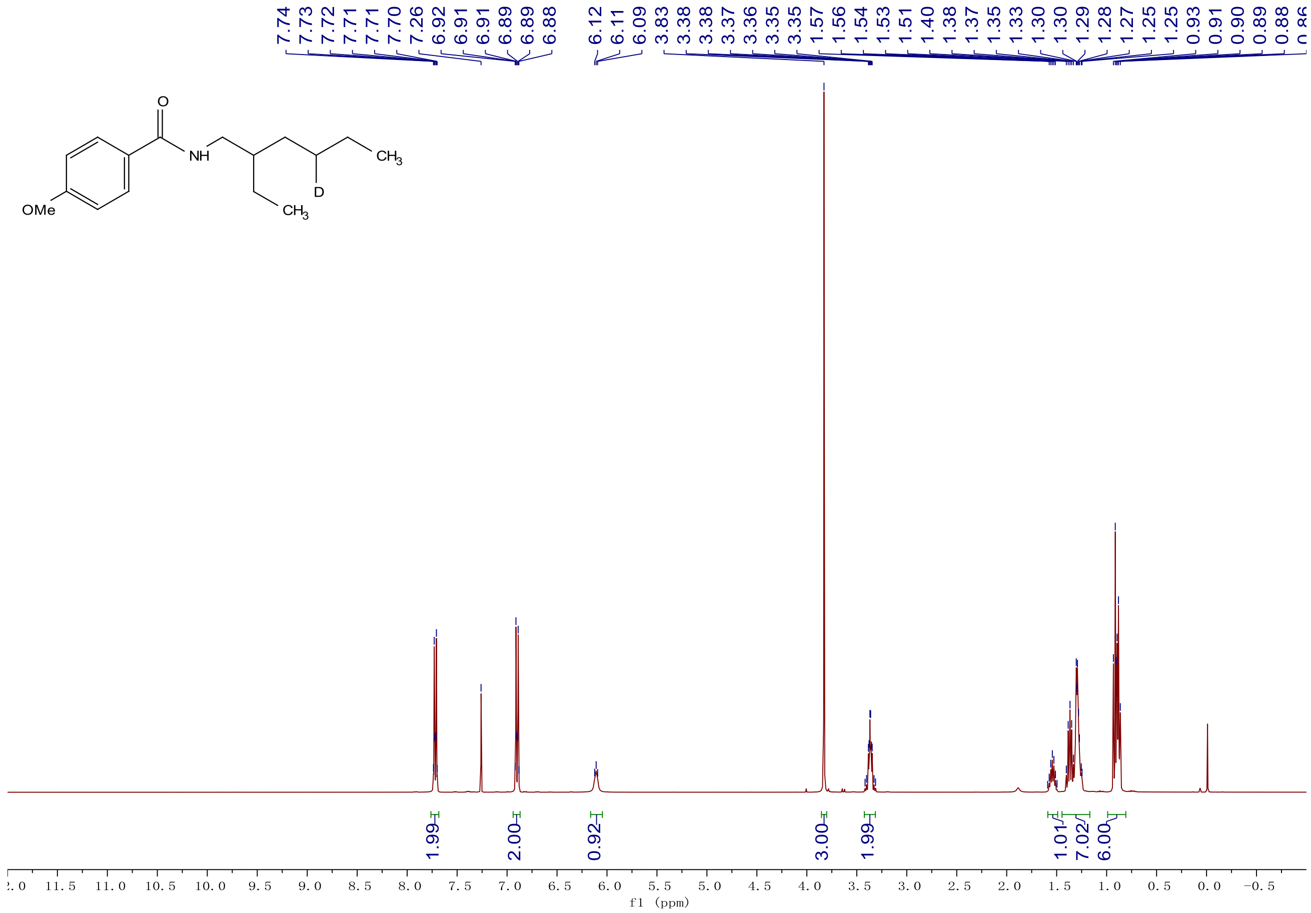

Supplementary Fig. 95. ^{1}H NMR (400 MHz, 298 K, Chloroform-*d*) spectrum of compound **3z**.

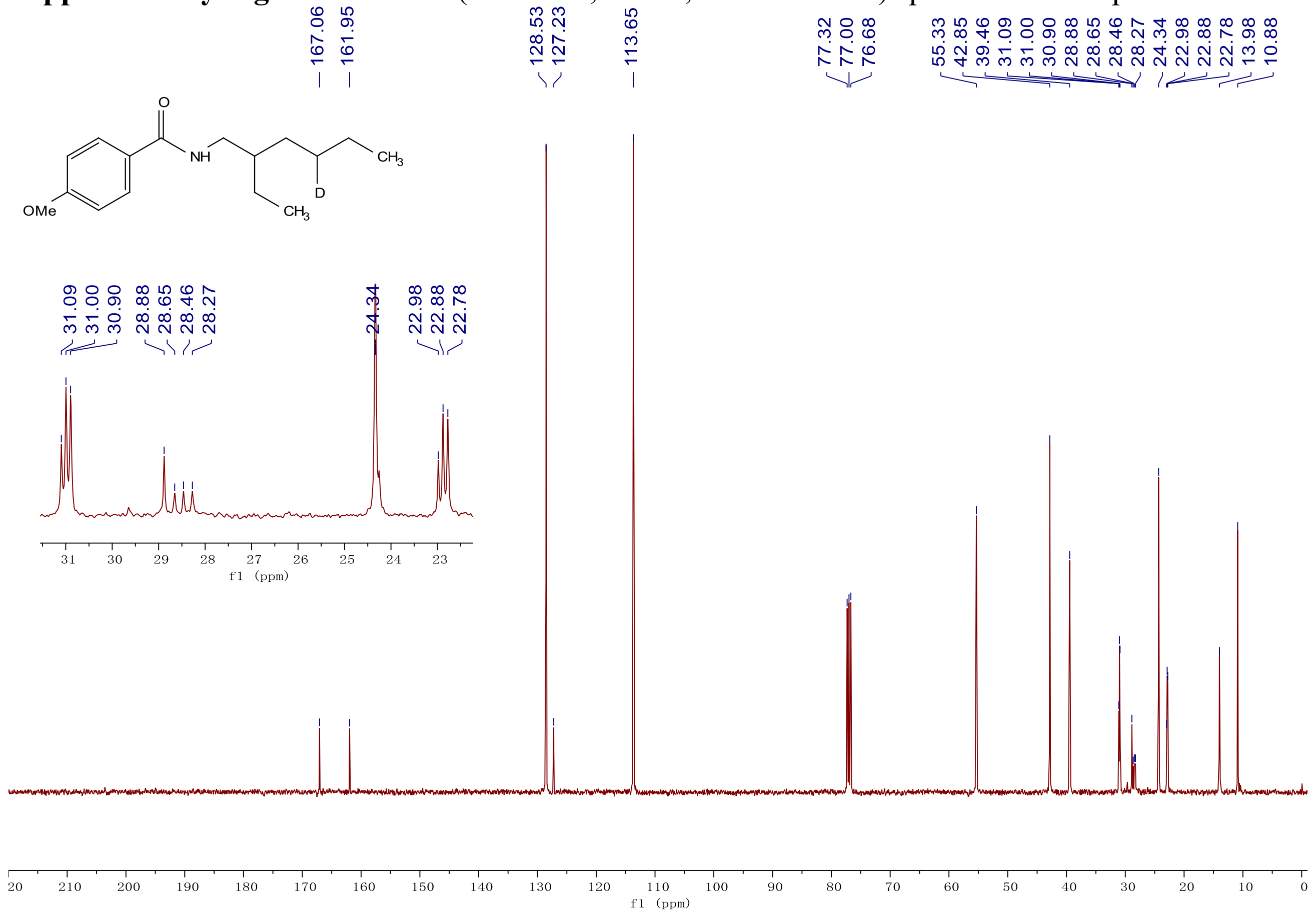

Supplementary Fig. 96. ^{13}C NMR (101 MHz, 298 K, Chloroform-*d*) spectrum of compound **3z**.

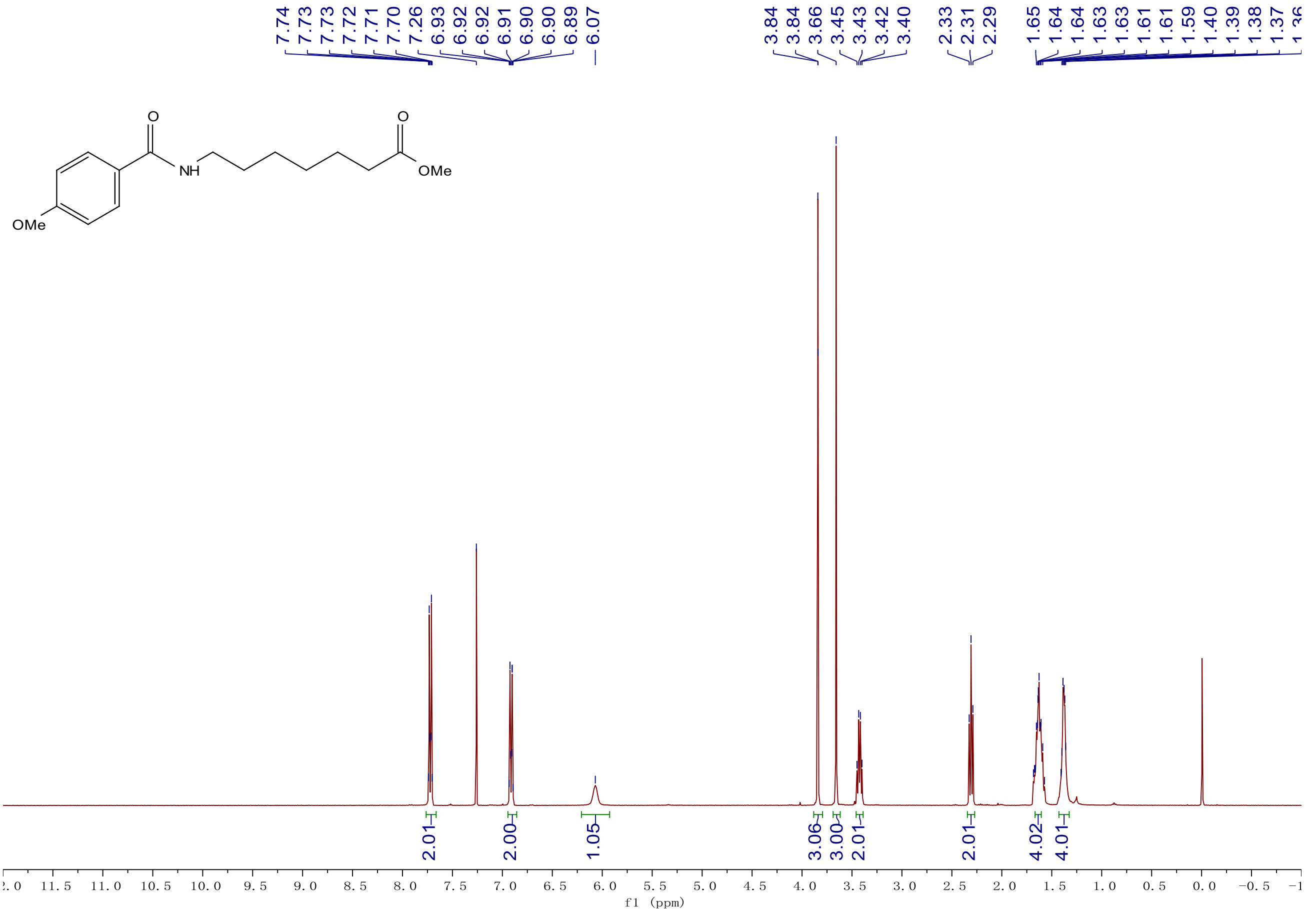

Supplementary Fig. 97. ^{1}H NMR (400 MHz, 298 K, Chloroform-*d*) spectrum of compound **1aa**.

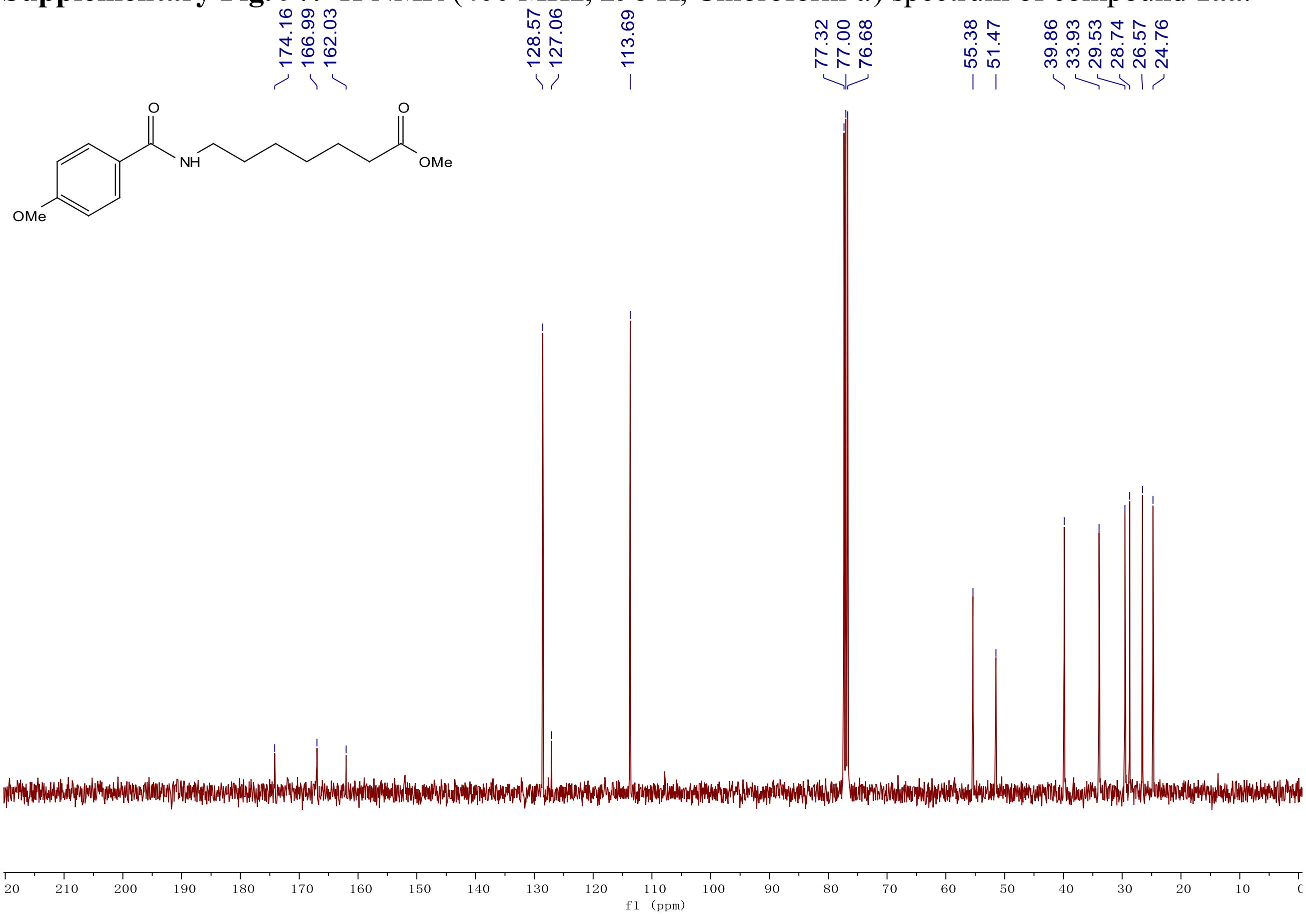

Supplementary Fig. 98. ^{13}C NMR (101 MHz, 298 K, Chloroform-*d*) spectrum of compound **1aa**.

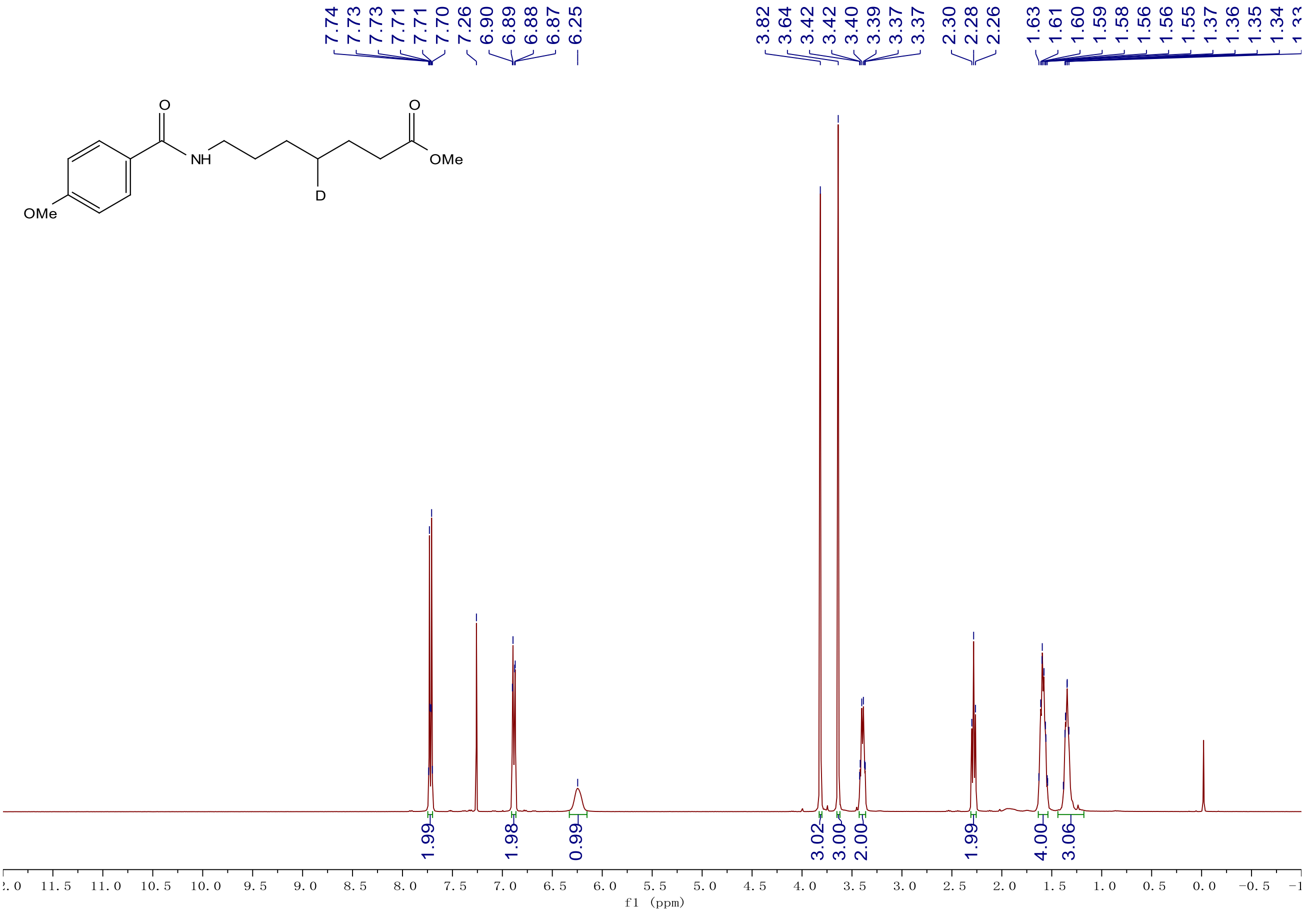

Supplementary Fig. 99. ^{1}H NMR (400 MHz, 298 K, Chloroform-*d*) spectrum of compound **3aa**.

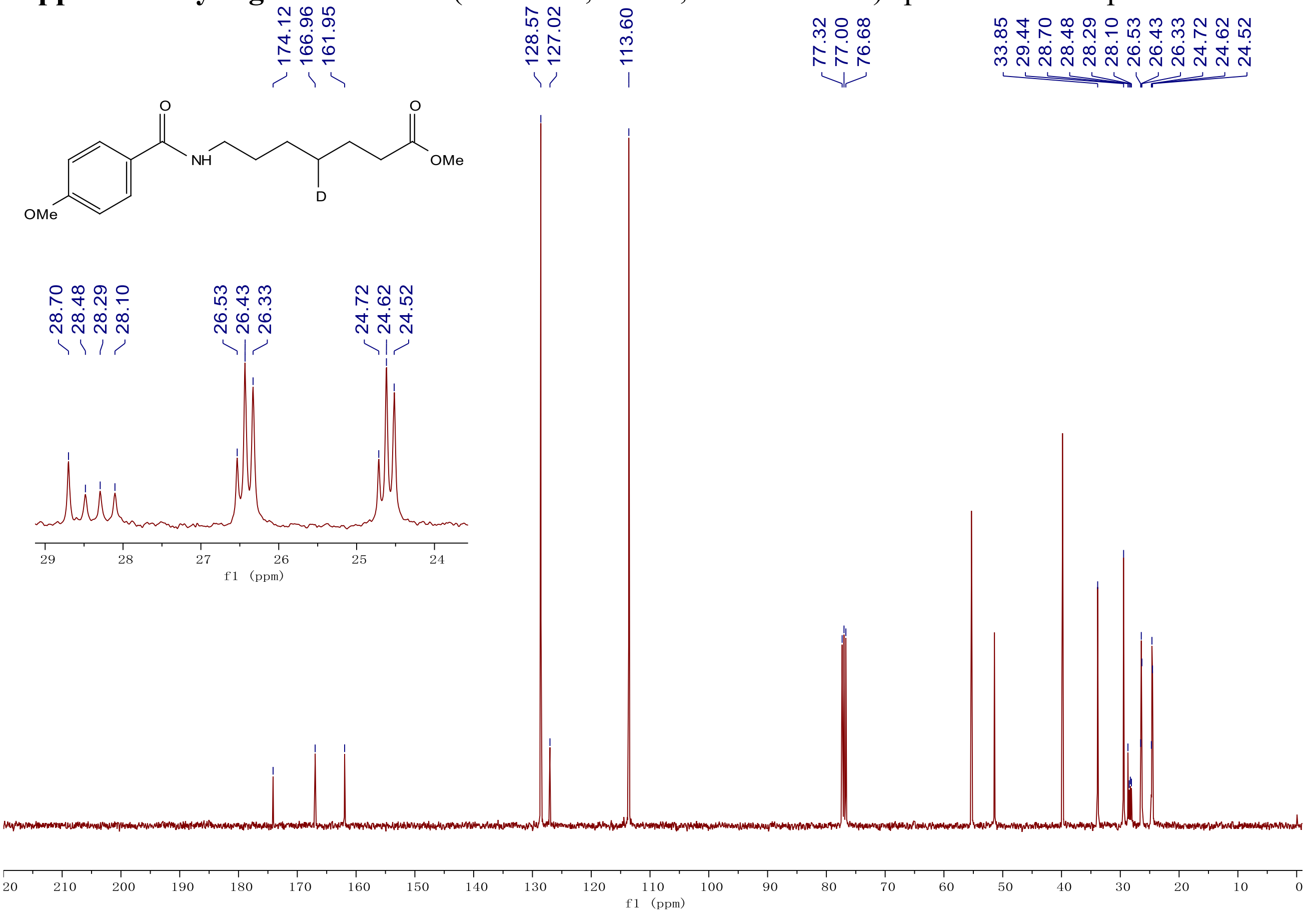

Supplementary Fig. 100. ^{13}C NMR (101 MHz, 298 K, Chloroform-*d*) spectrum of compound **3aa**.

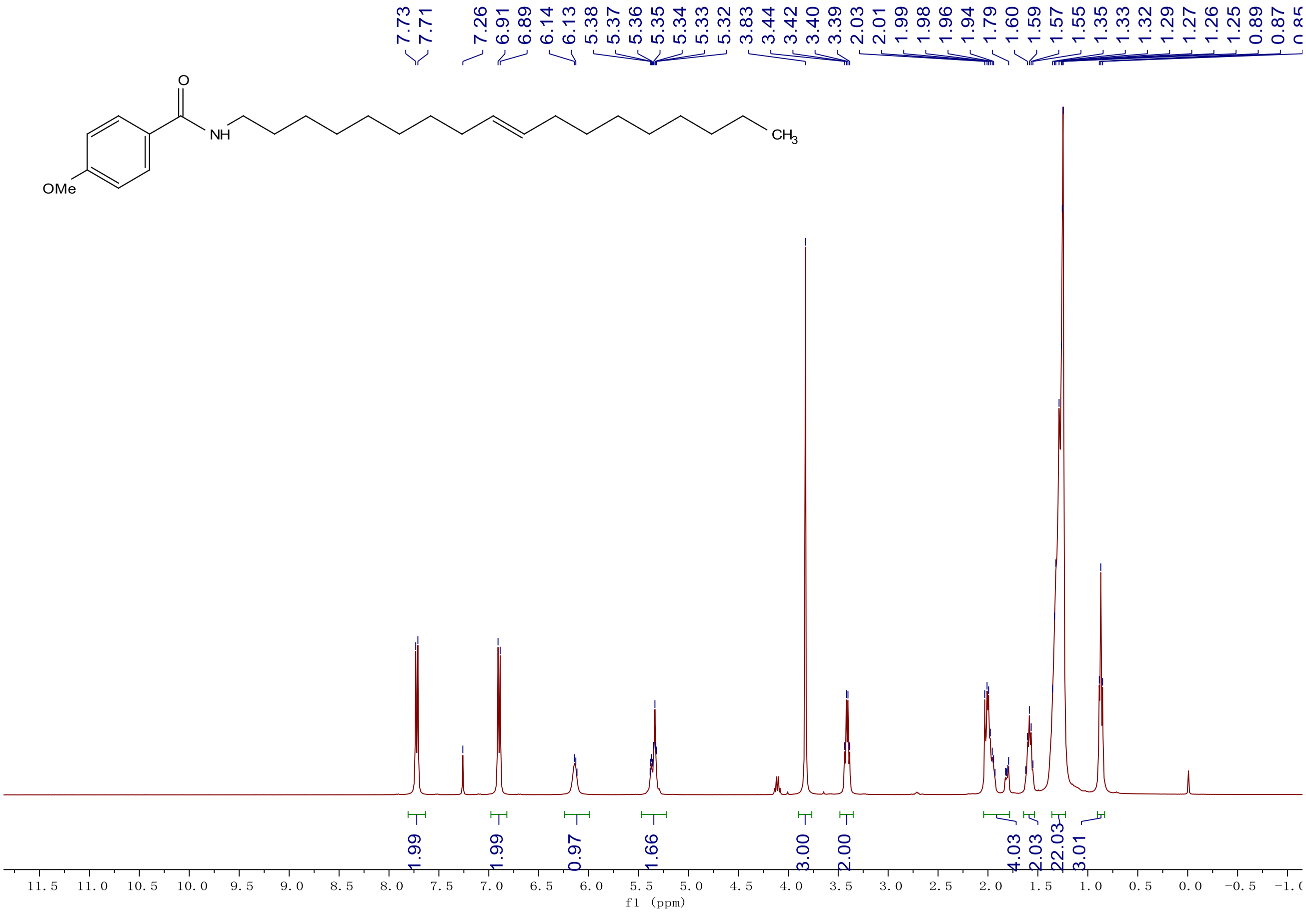

Supplementary Fig. 101. ^{1}H NMR (400 MHz, 298 K, Chloroform-*d*) spectrum of compound **1bb**.

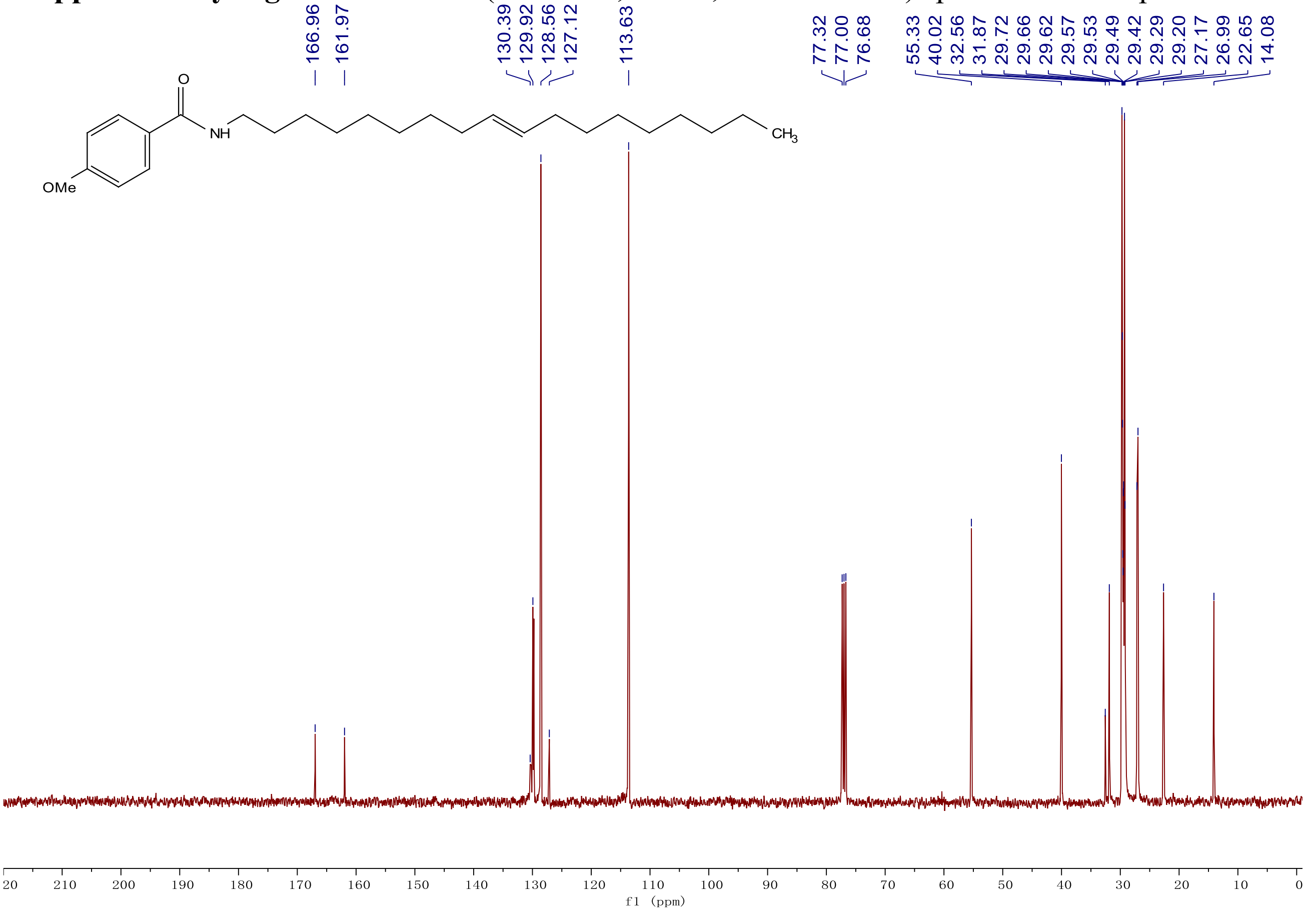

Supplementary Fig. 102. ^{13}C NMR (101 MHz, 298 K, Chloroform-*d*) spectrum of compound **1bb**.

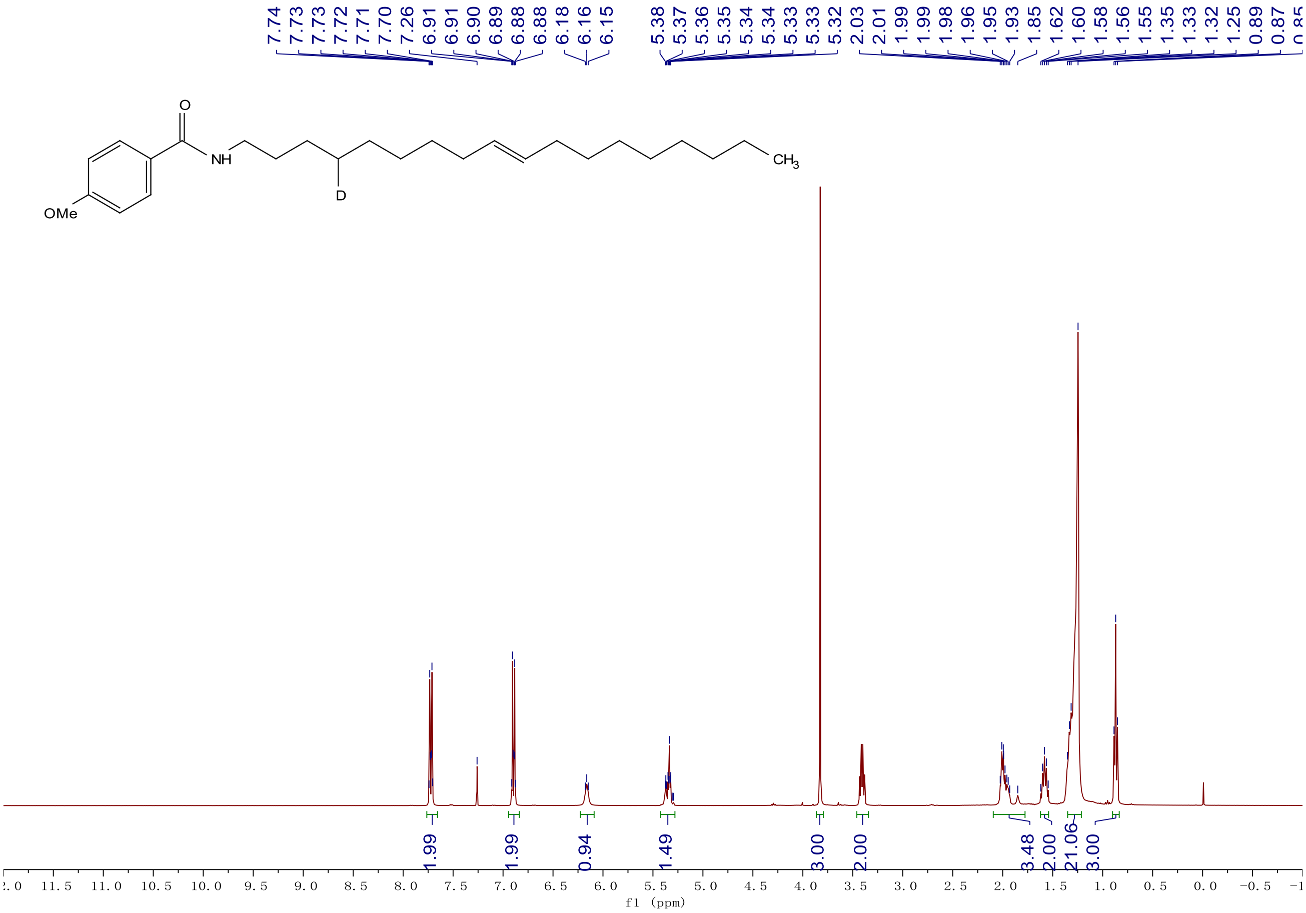

Supplementary Fig. 103. ^{1}H NMR (400 MHz, 298 K, Chloroform-*d*) spectrum of compound **3bb**.

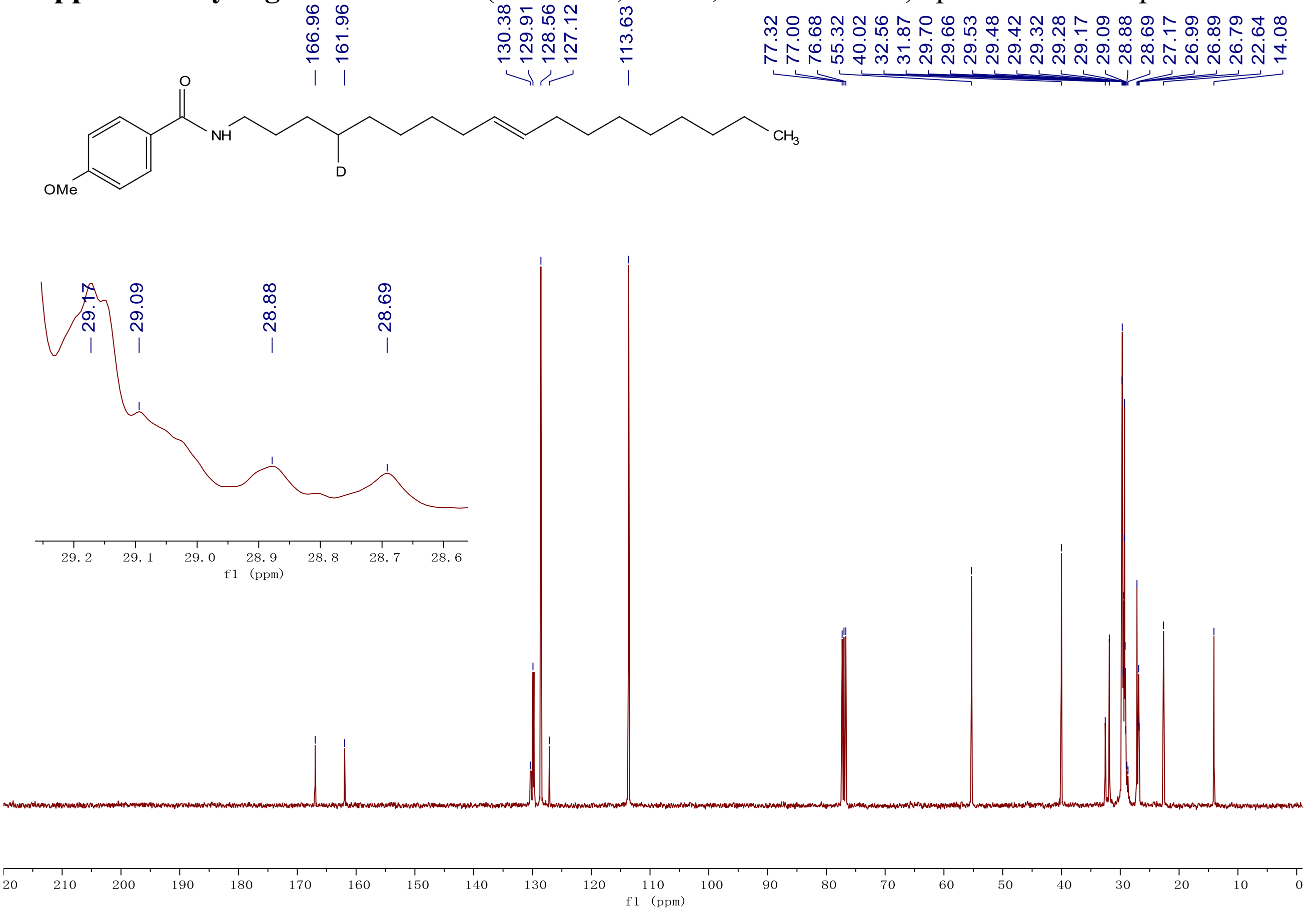

Supplementary Fig. 104. ^{13}C NMR (101MHz, 298 K, Chloroform-*d*) spectrum of compound **3bb**.

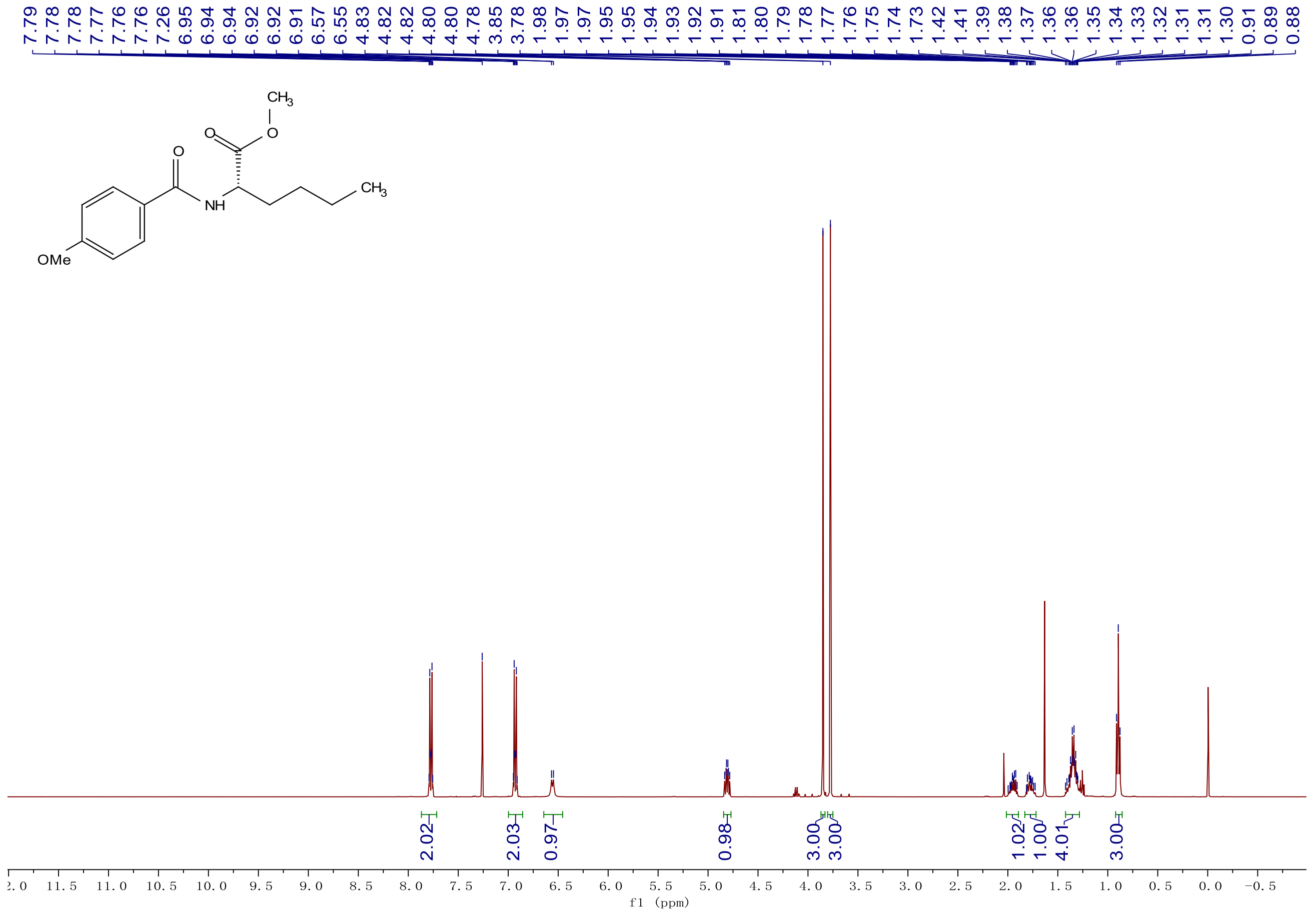

Supplementary Fig. 105. ^{1}H NMR (400 MHz, 298 K, Chloroform-*d*) spectrum of compound **1cc**.

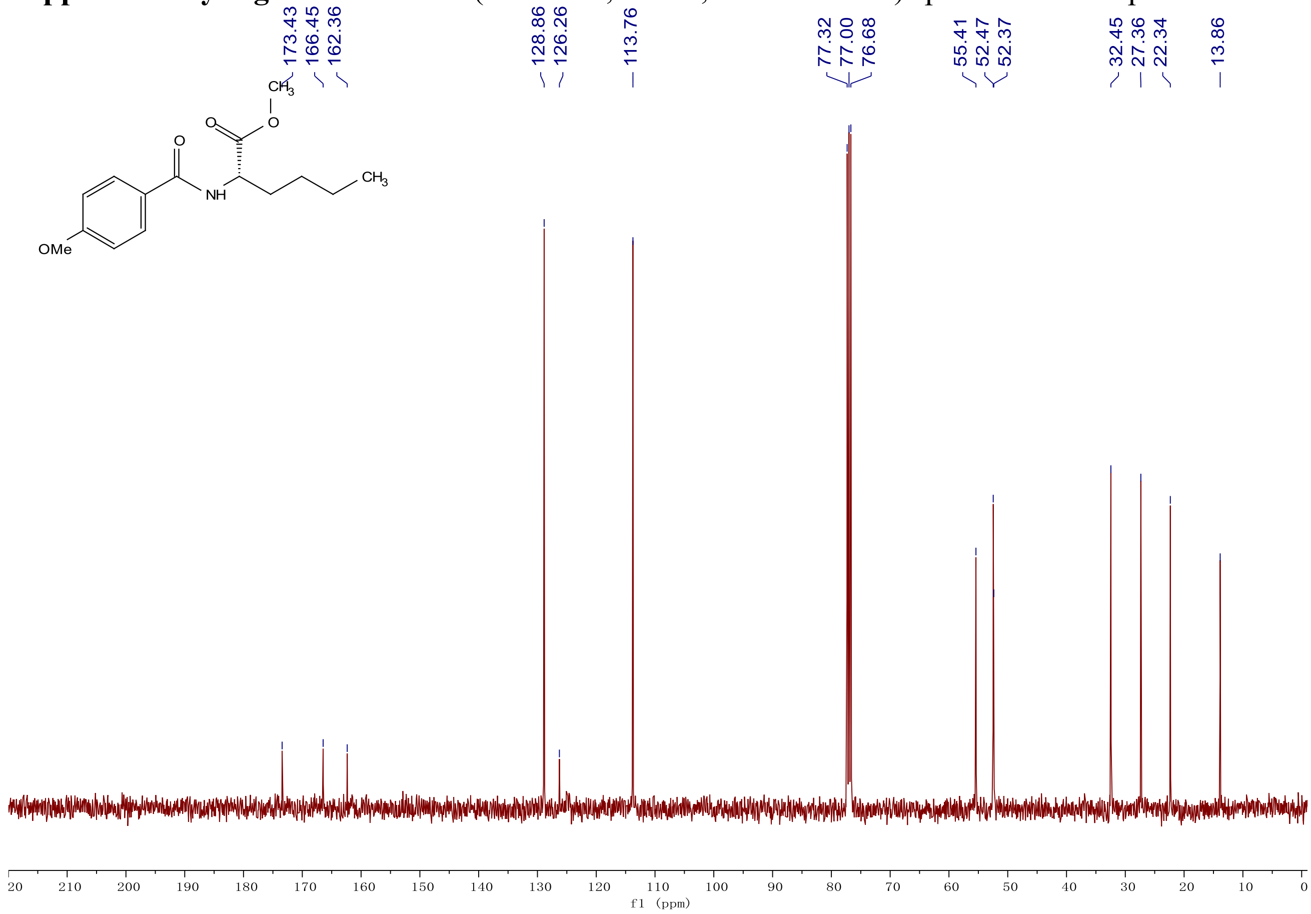

Supplementary Fig. 106. ^{13}C NMR (101 MHz, 298 K, Chloroform-*d*) spectrum of compound **1cc**.

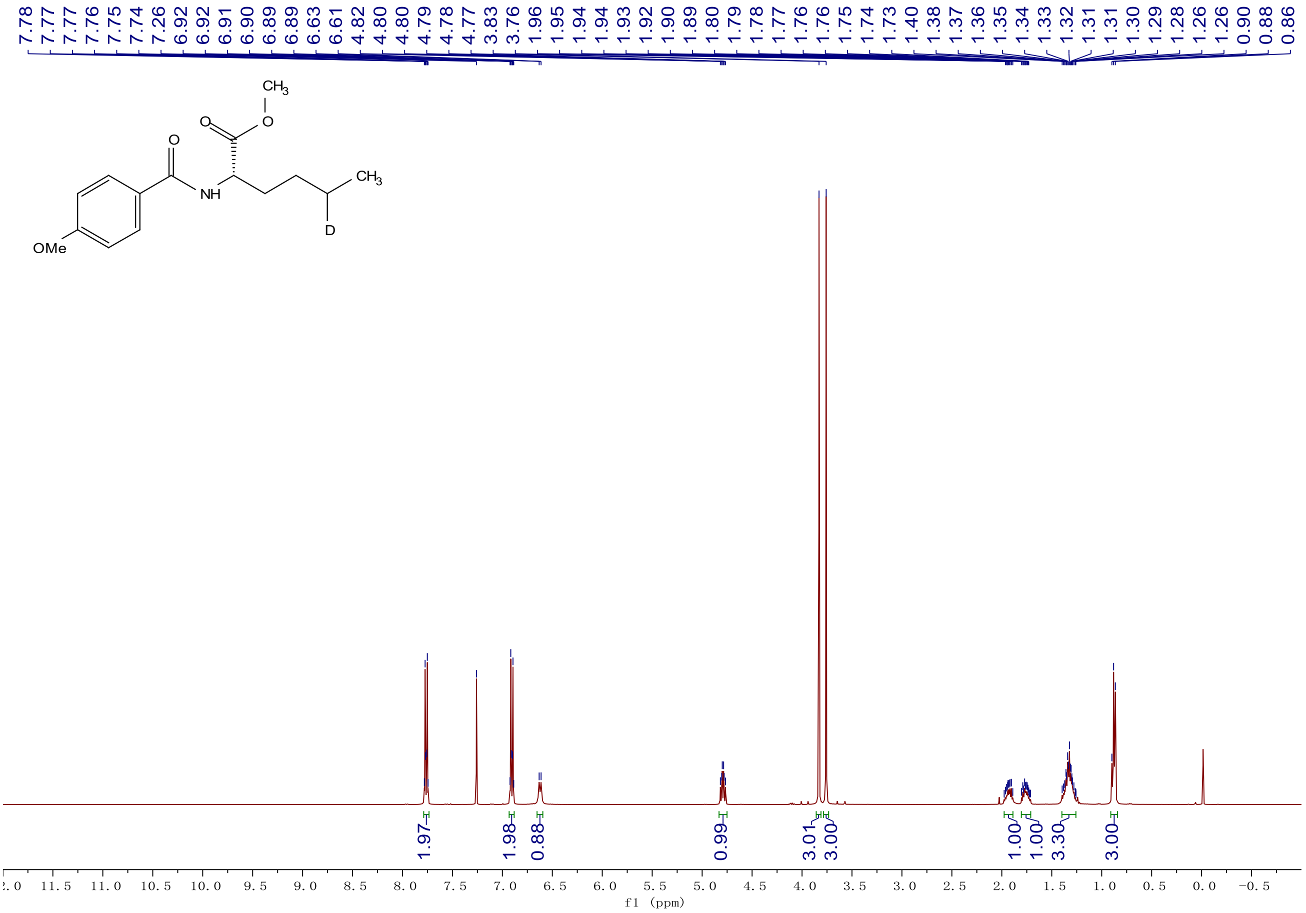

Supplementary Fig. 107. ^{1}H NMR (400 MHz, 298 K, Chloroform-*d*) spectrum of compound **3cc**.

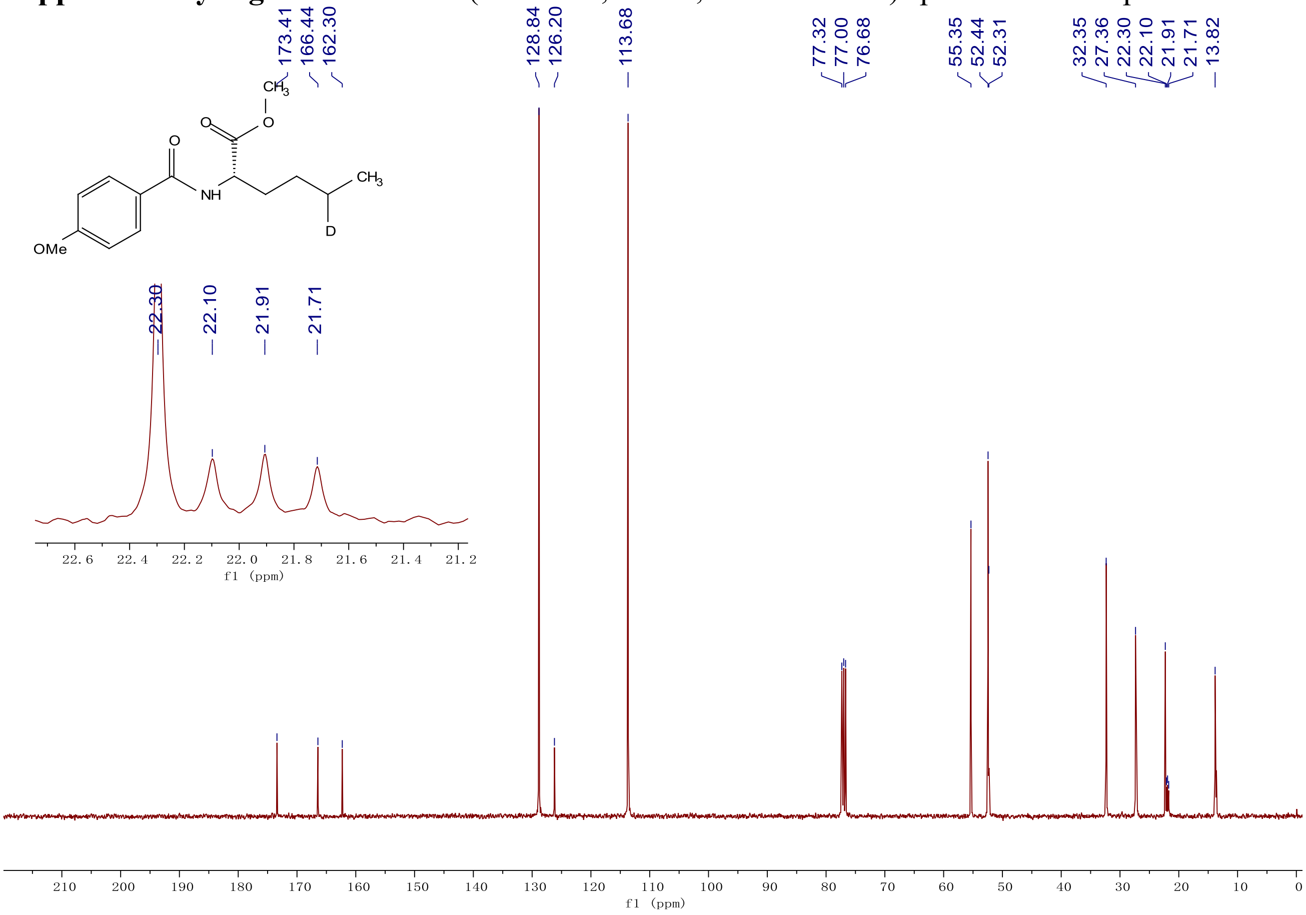

Supplementary Fig. 108. ^{13}C NMR (101 MHz, 298 K, Chloroform-*d*) spectrum of compound **3cc**.

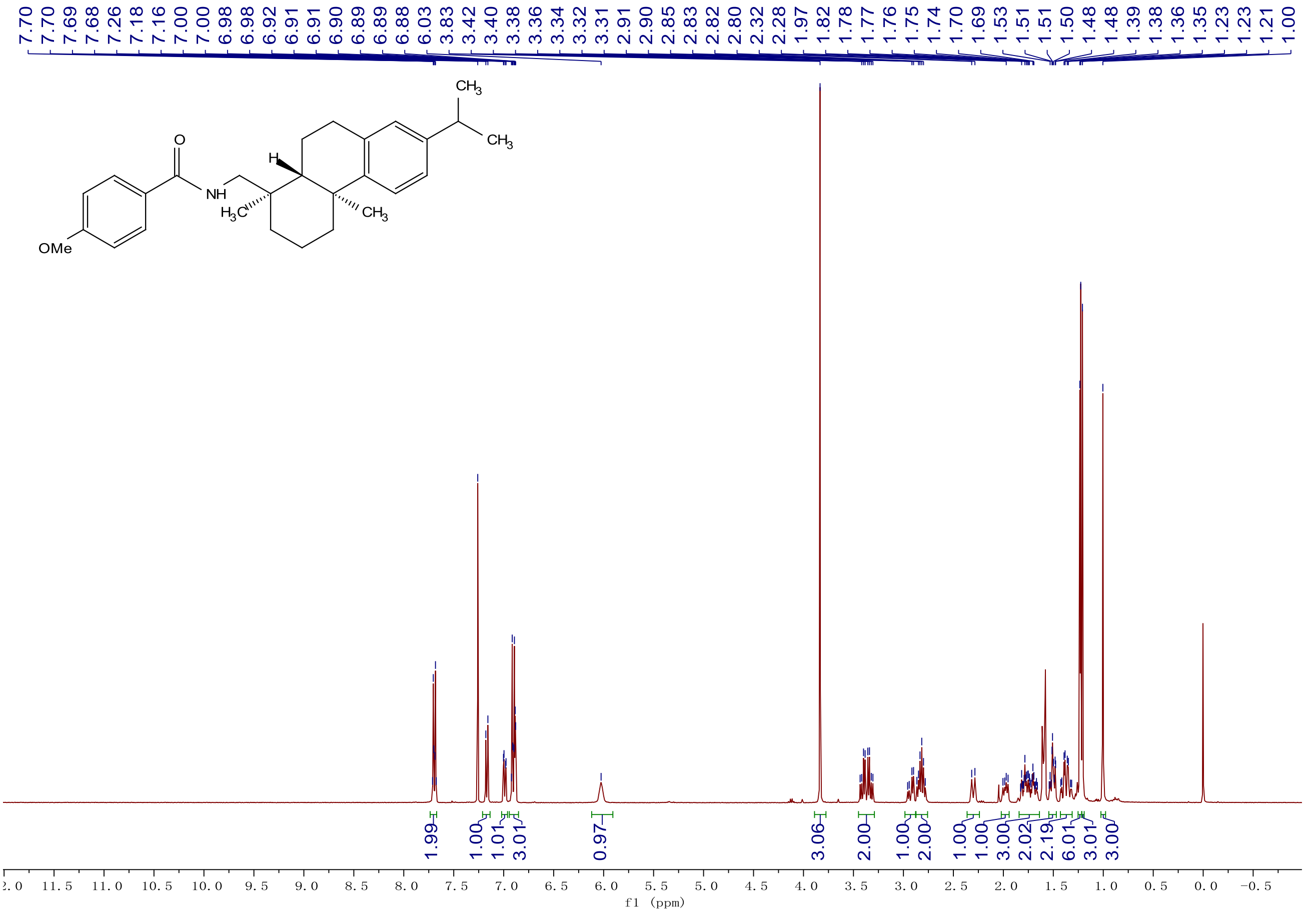

Supplementary Fig. 109. ^{1}H NMR (400 MHz, 298 K, Chloroform-*d*) spectrum of compound **1dd**.

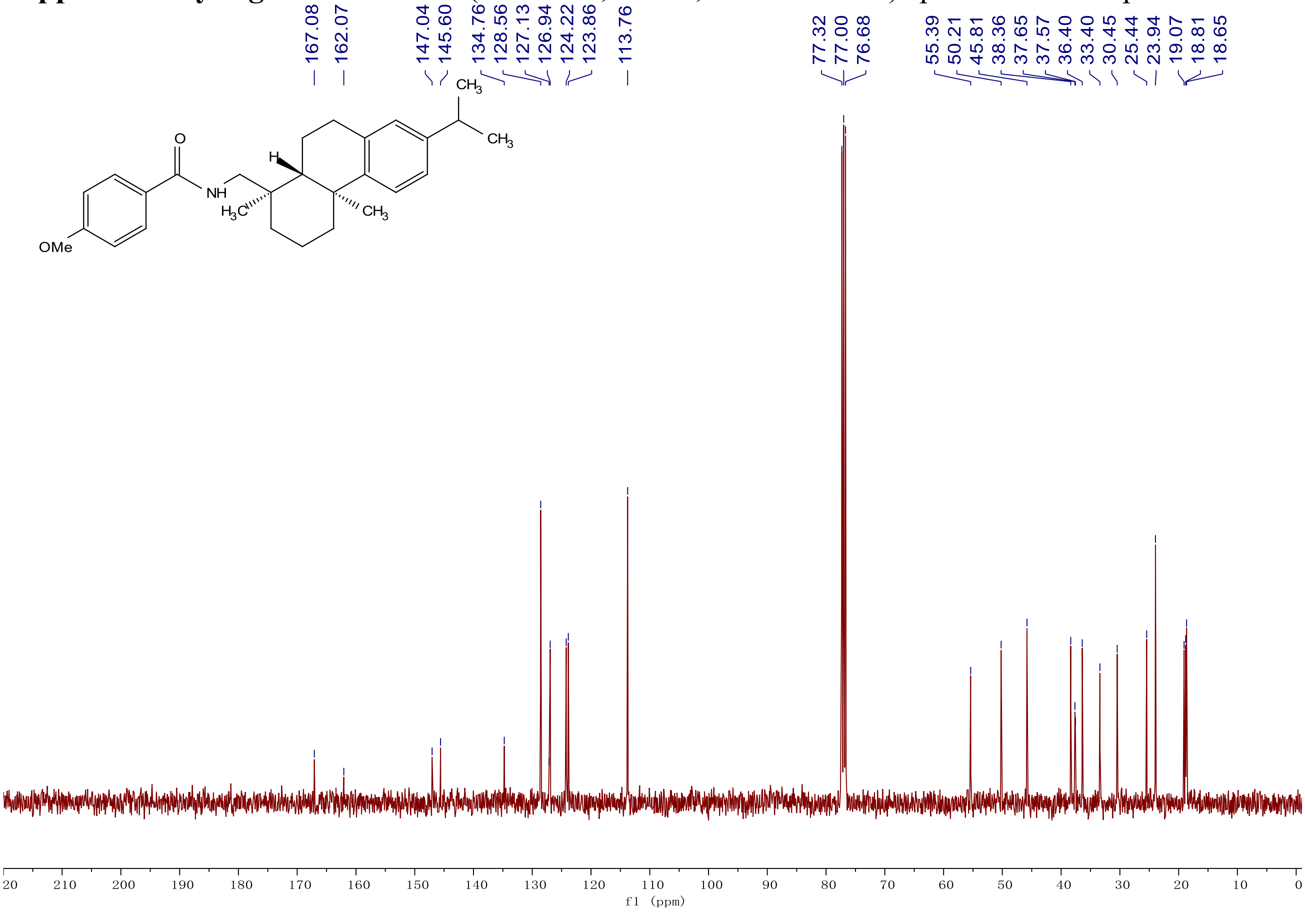

Supplementary Fig. 110. ^{13}C NMR (101 MHz, 298 K, Chloroform-*d*) spectrum of compound **1dd**.

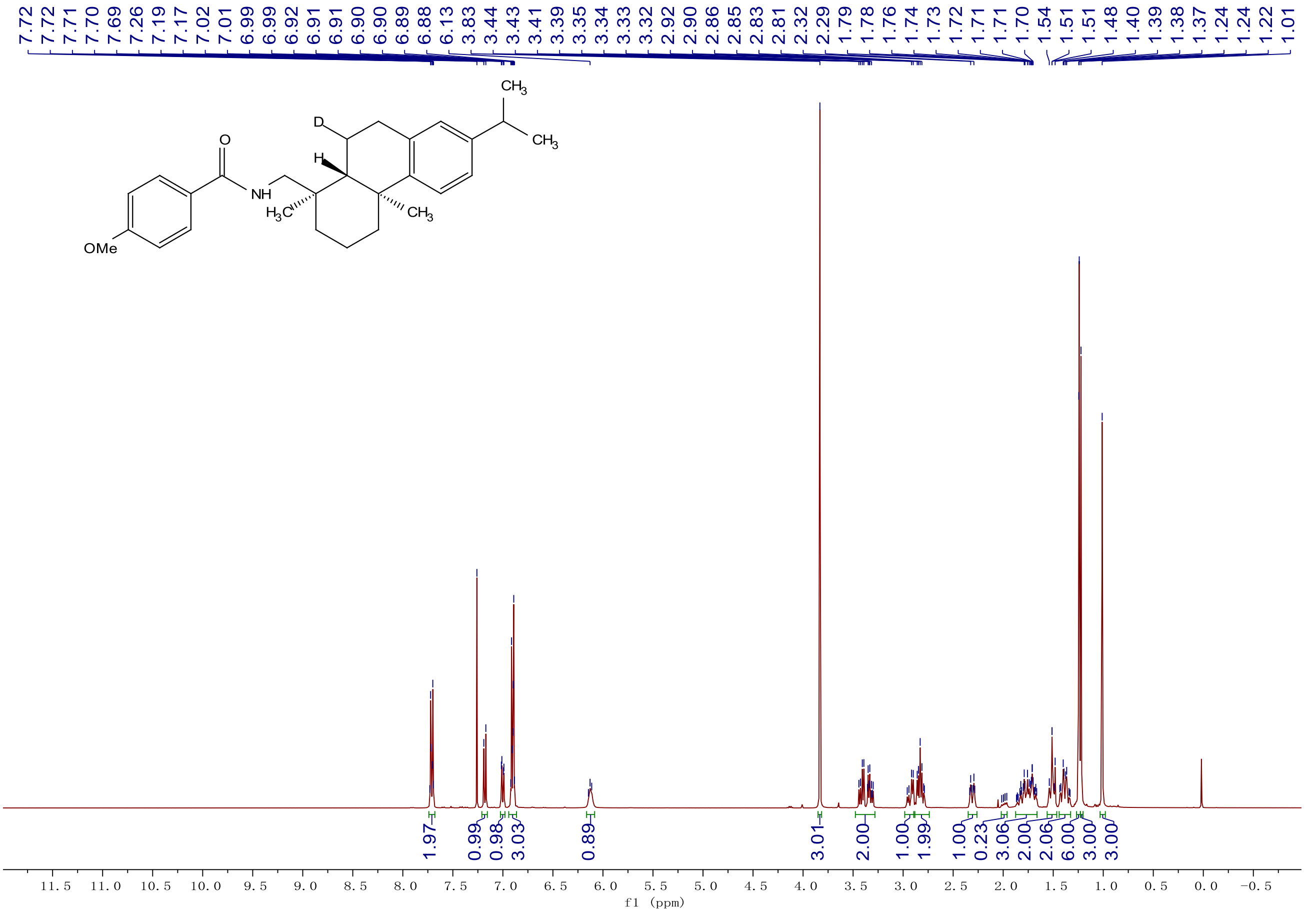

Supplementary Fig. 111. ^{1}H NMR (400 MHz, 298 K, Chloroform-*d*) spectrum of compound **3dd**.

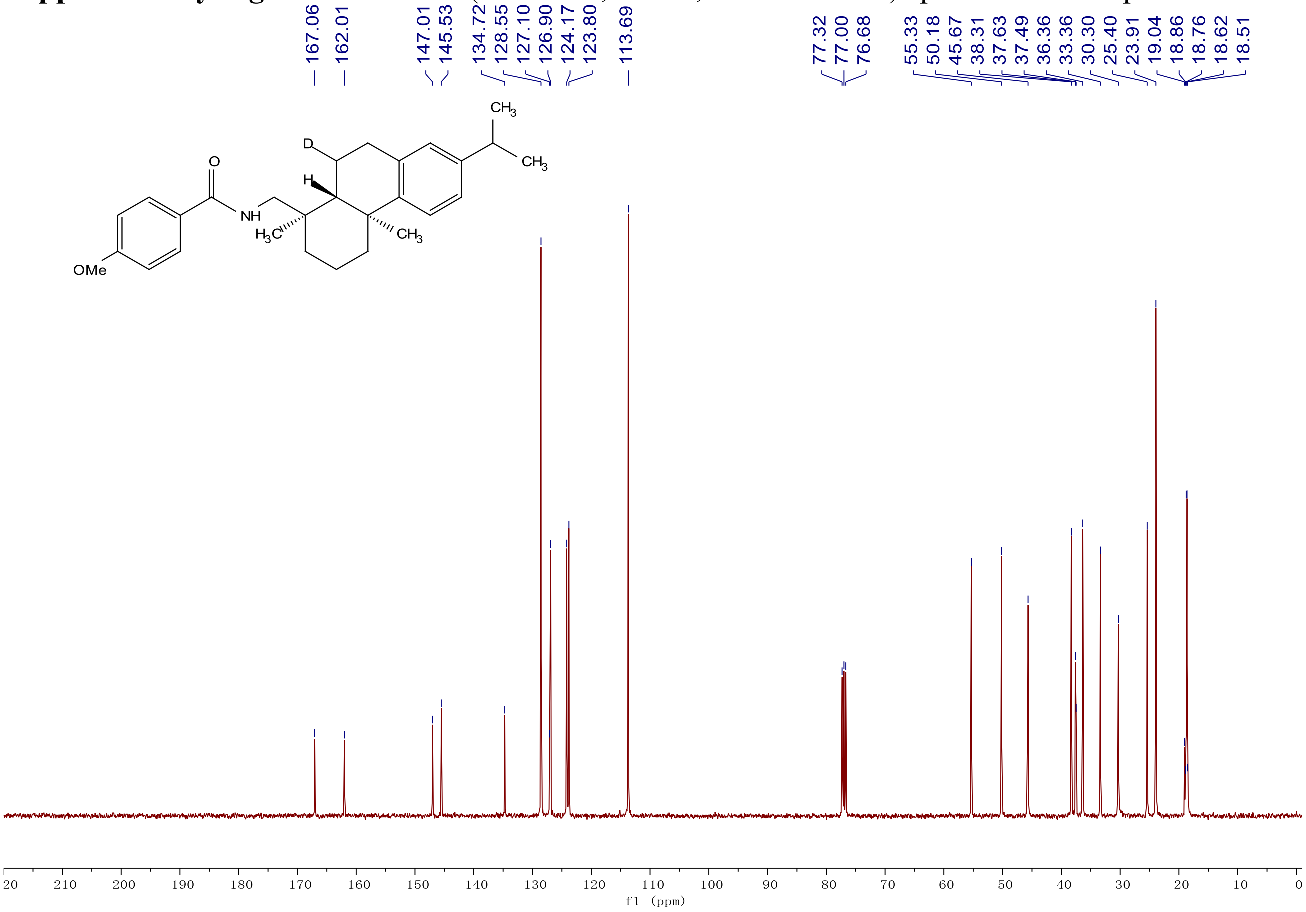

Supplementary Fig. 112. ^{13}C NMR (101 MHz, 298 K, Chloroform-*d*) spectrum of compound **3dd**.

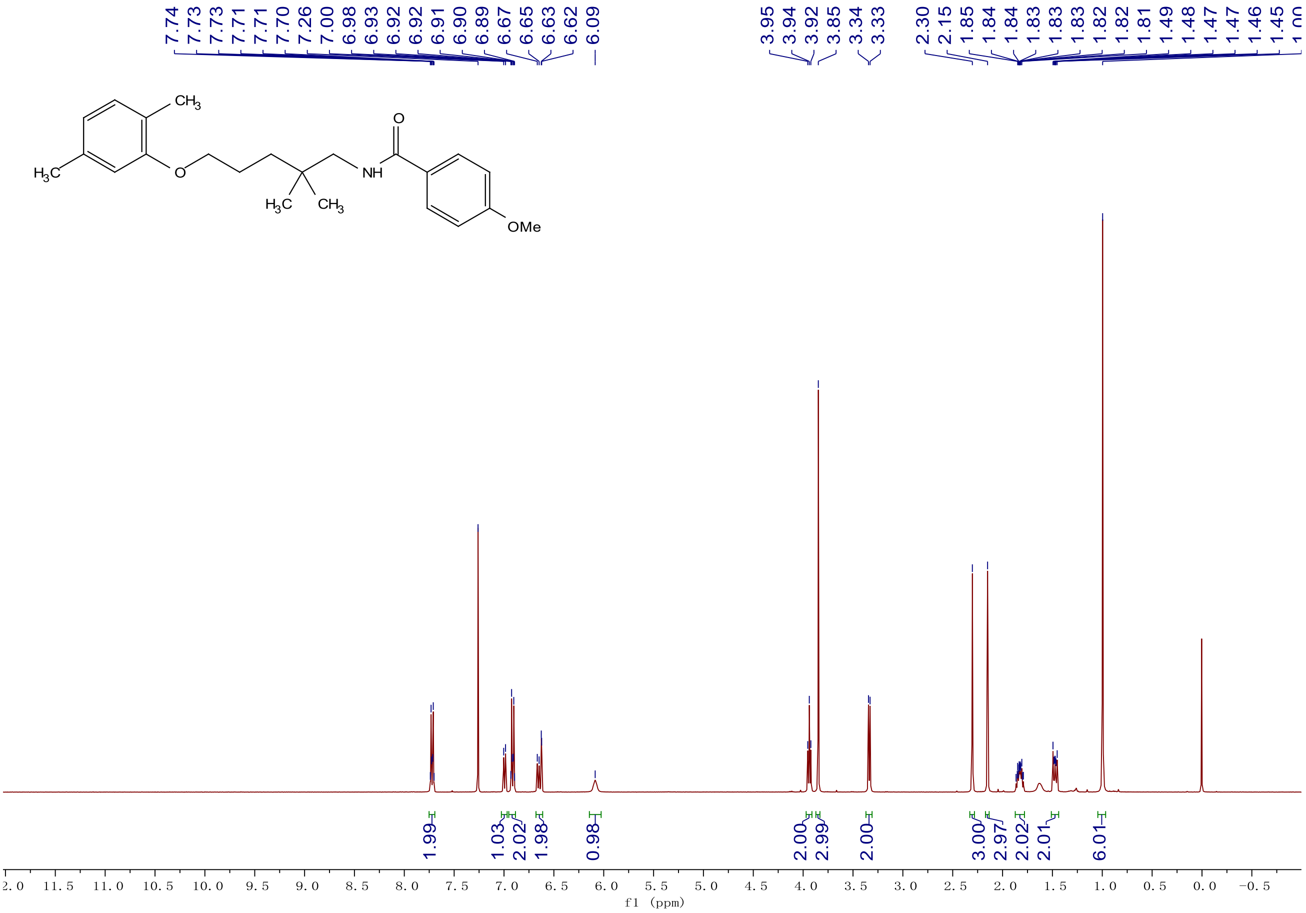

Supplementary Fig. 113. ^{1}H NMR (400 MHz, 298 K, Chloroform-d) spectrum of compound **1ee**.

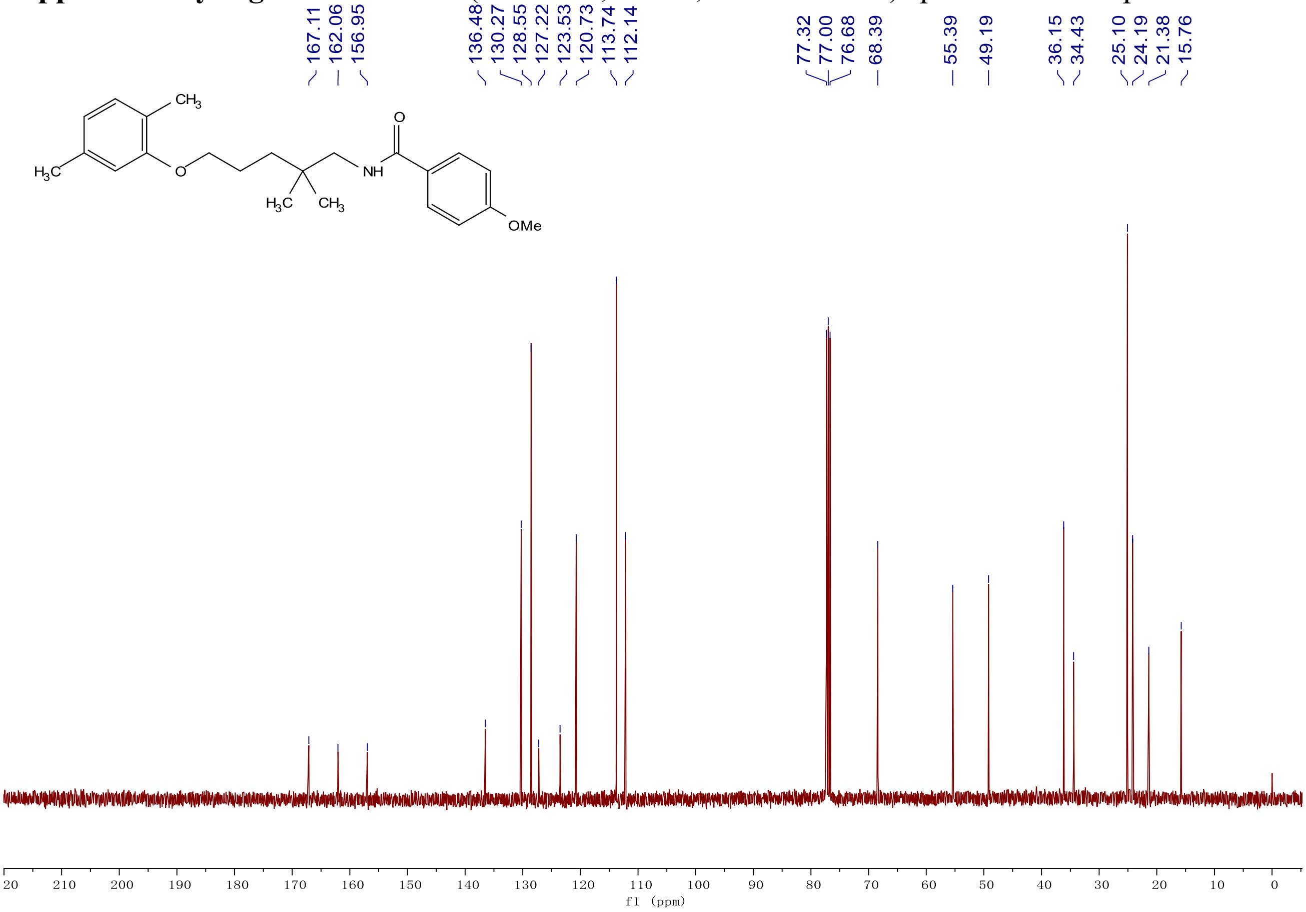

Supplementary Fig. 114. ^{13}C NMR (101 MHz, 298 K, Chloroform-d) spectrum of compound **1ee**.

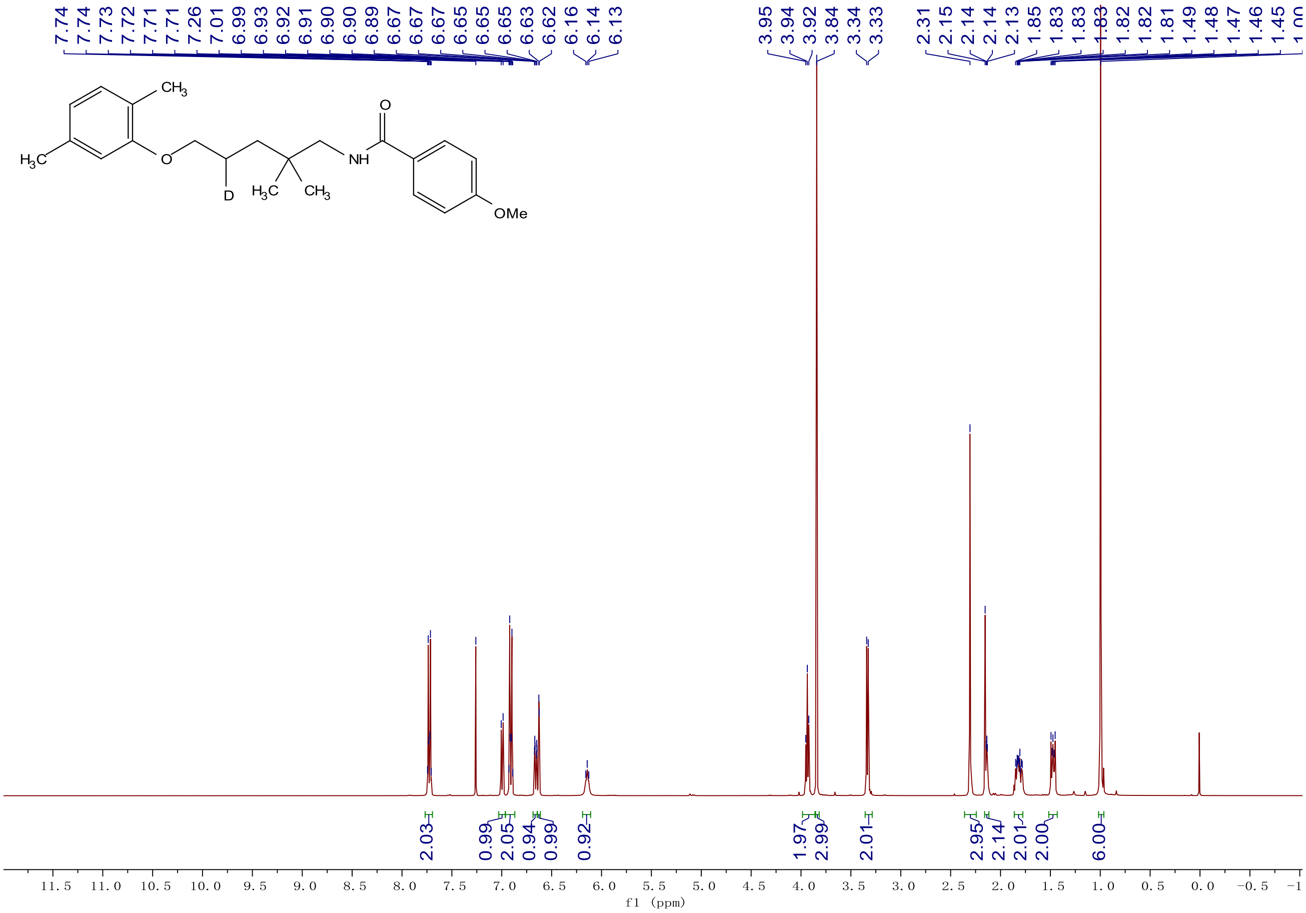

Supplementary Fig. 115. ^{1}H NMR (400 MHz, 298 K, Chloroform-*d*) spectrum of compound **3ee**.

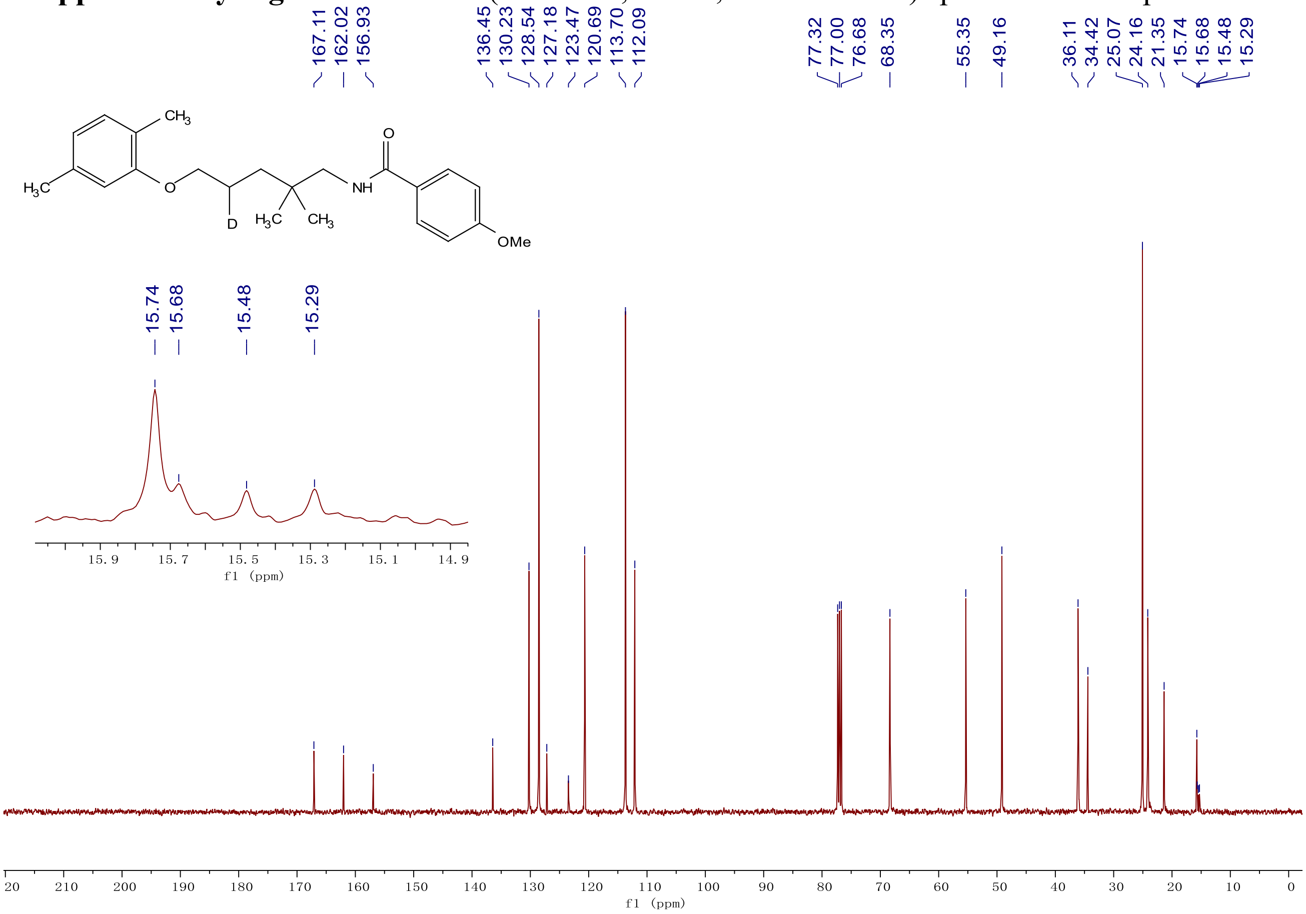

Supplementary Fig. 116. ^{13}C NMR (101 MHz, 298 K, Chloroform-*d*) spectrum of compound **3ee**.

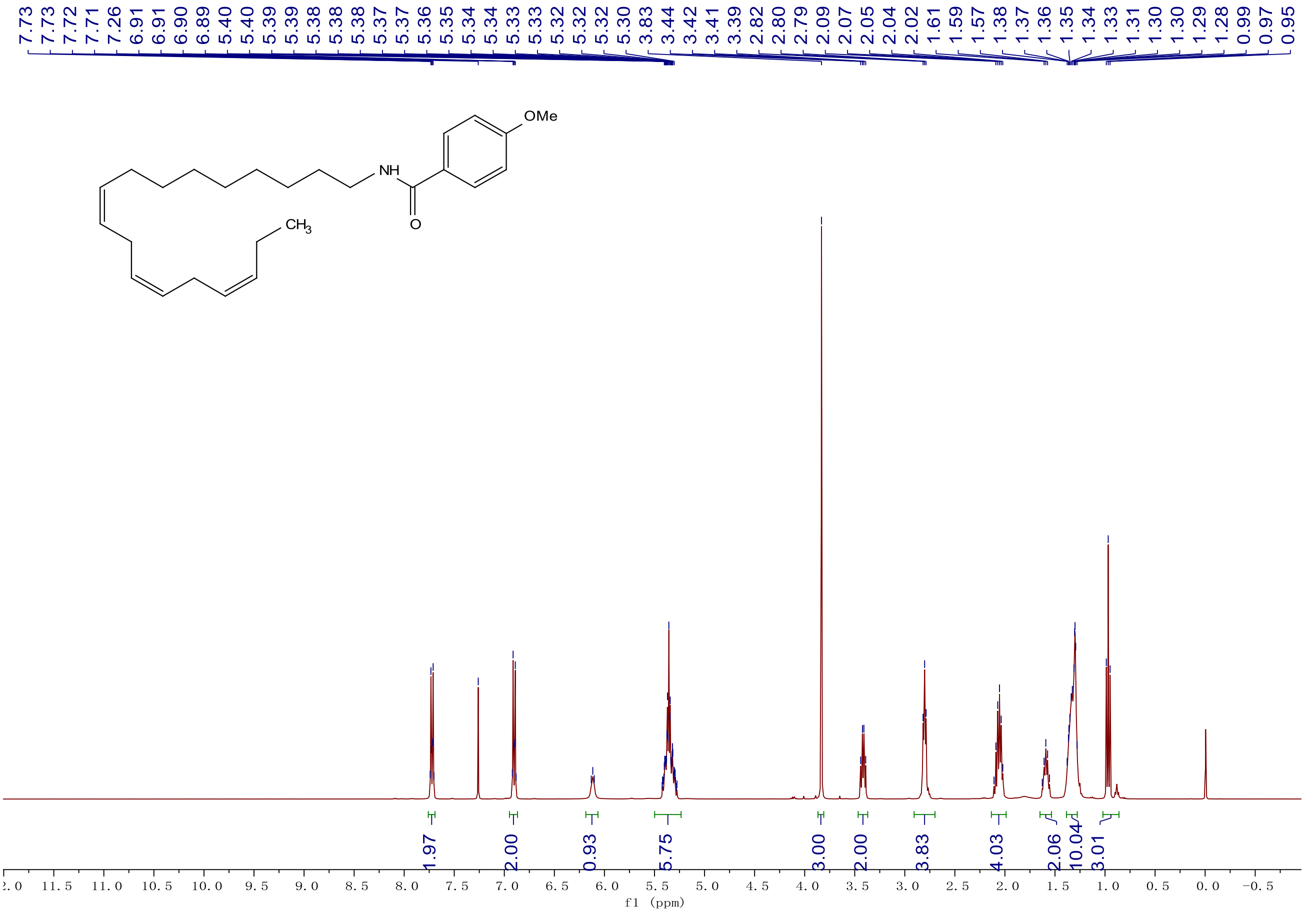

Supplementary Fig. 117. ^{1}H NMR (400 MHz, 298 K, Chloroform-*d*) spectrum of compound **1ff**.

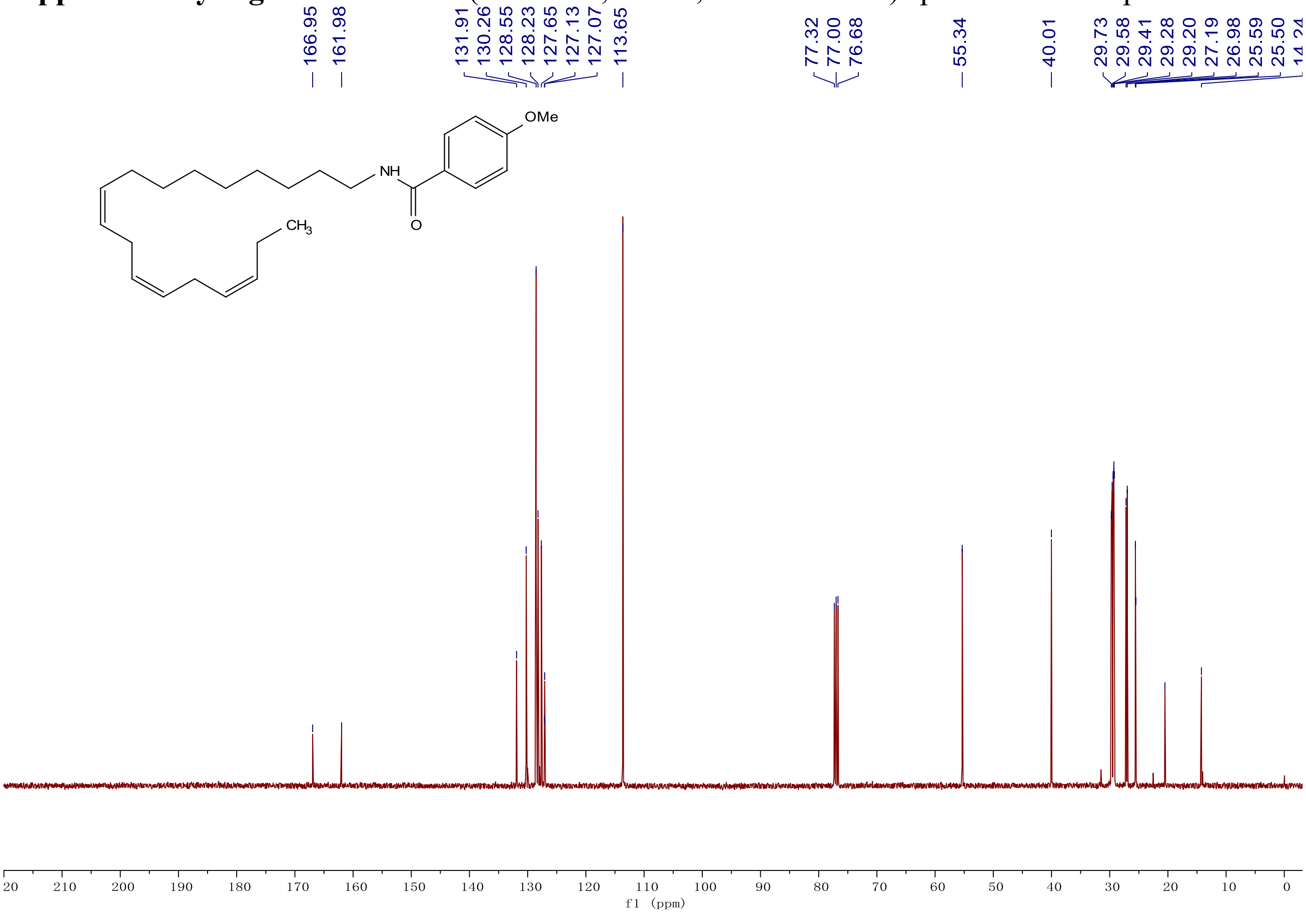

Supplementary Fig. 118. ^{13}C NMR (101 MHz, 298 K, Chloroform-*d*) spectrum of compound **1ff**.

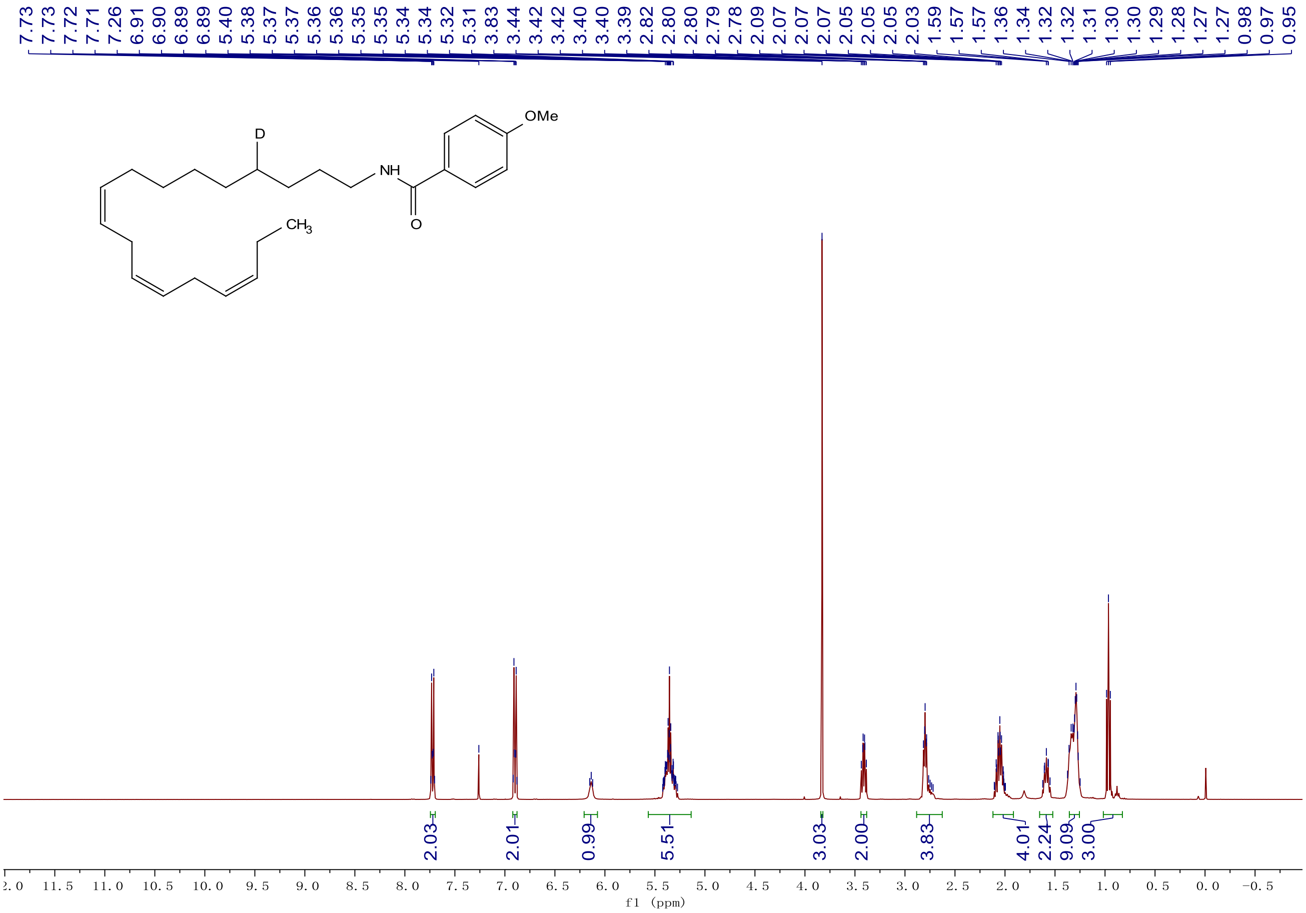

Supplementary Fig. 119. ^{1}H NMR (400 MHz, 298 K, Chloroform-*d*) spectrum of compound **3ff**.

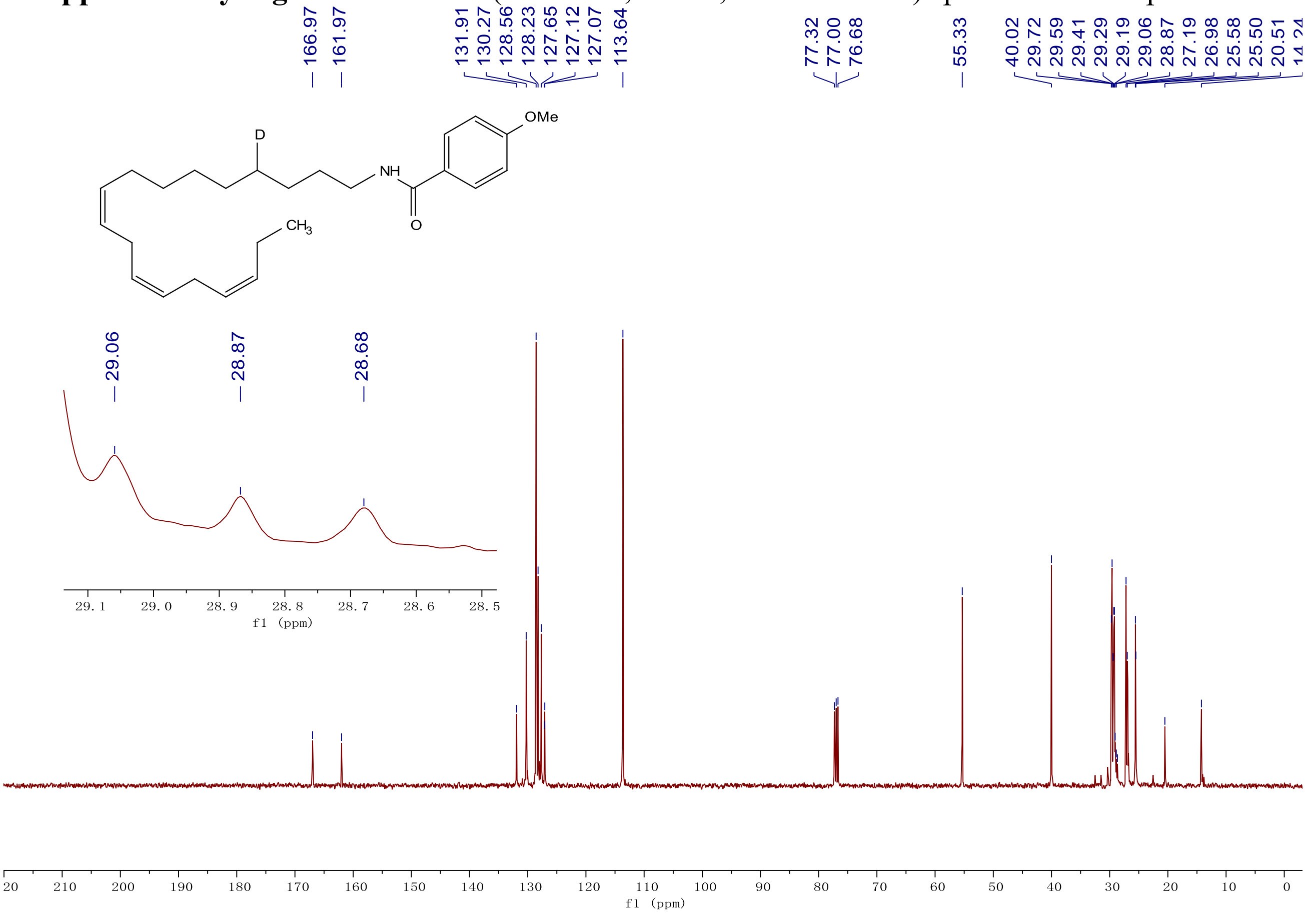

Supplementary Fig. 120. ^{13}C NMR (101 MHz, 298 K, Chloroform-*d*) spectrum of compound **3ff**.

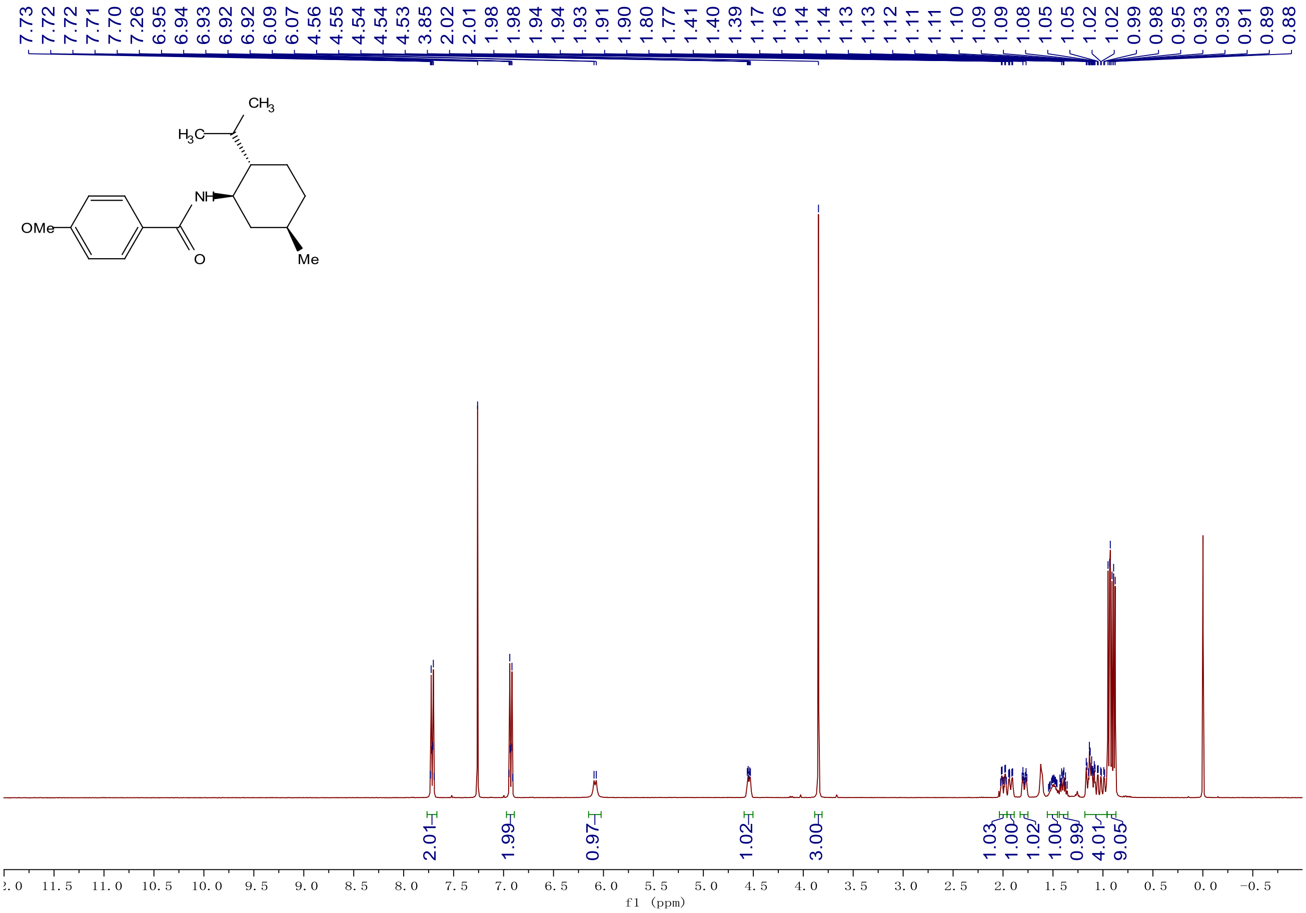

Supplementary Fig. 121. ^{1}H NMR (400 MHz, 298 K, Chloroform-*d*) spectrum of compound **1gg**.

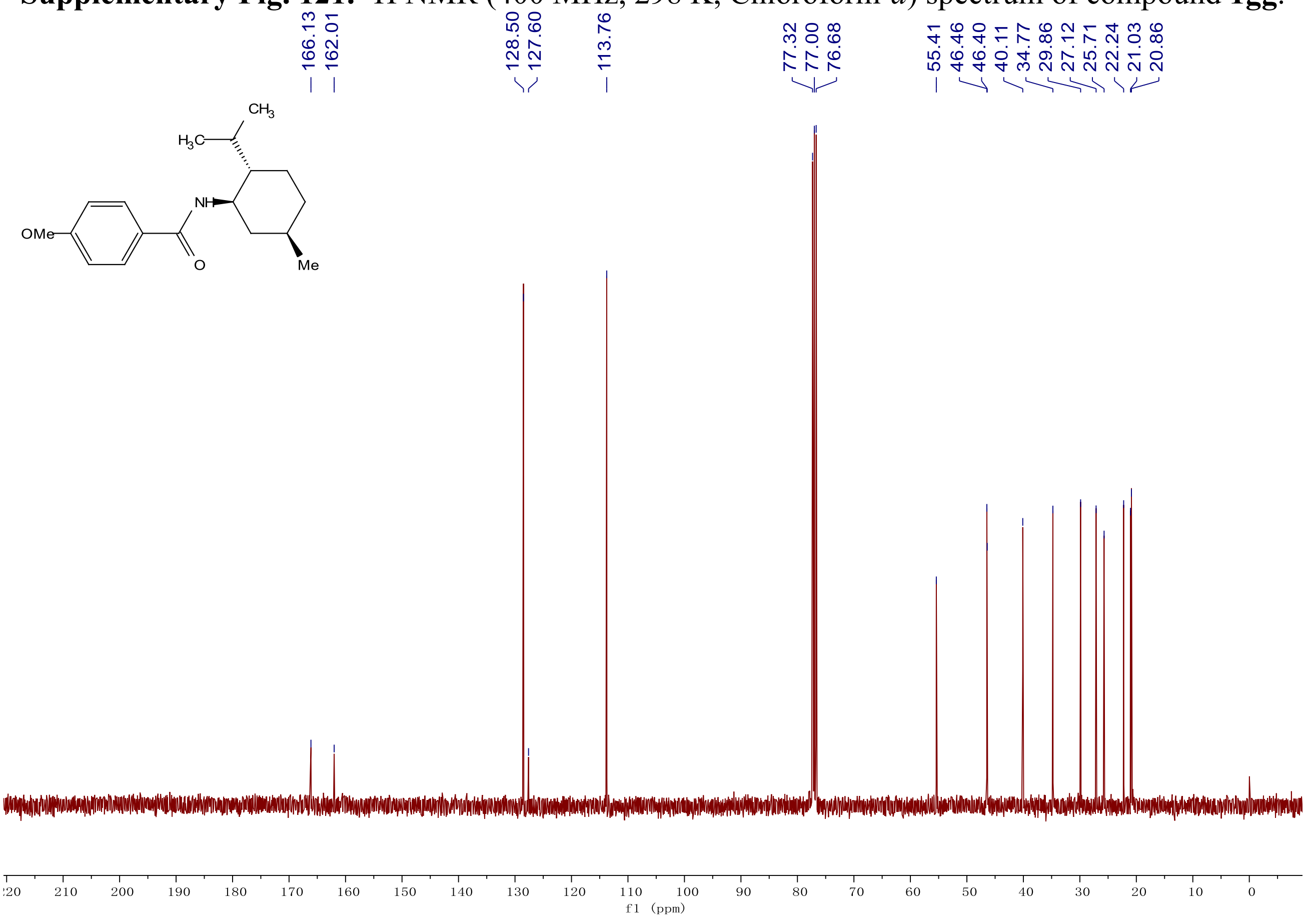

Supplementary Fig. 122. ^{13}C NMR (101 MHz, 298 K, Chloroform-*d*) spectrum of compound **1gg**.

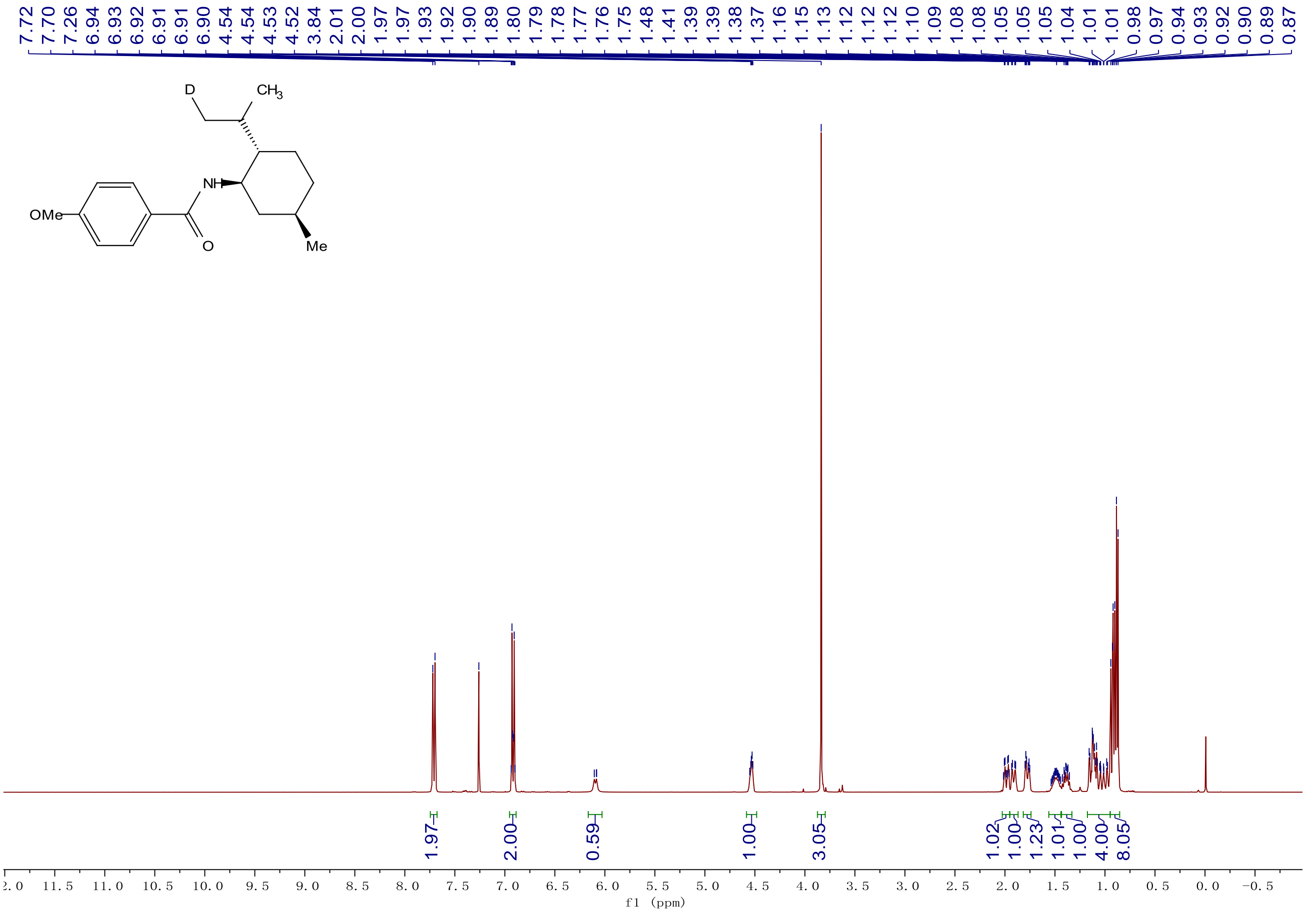

Supplementary Fig. 123. ^{1}H NMR (400 MHz, 298 K, Chloroform-*d*) spectrum of compound **3gg**.

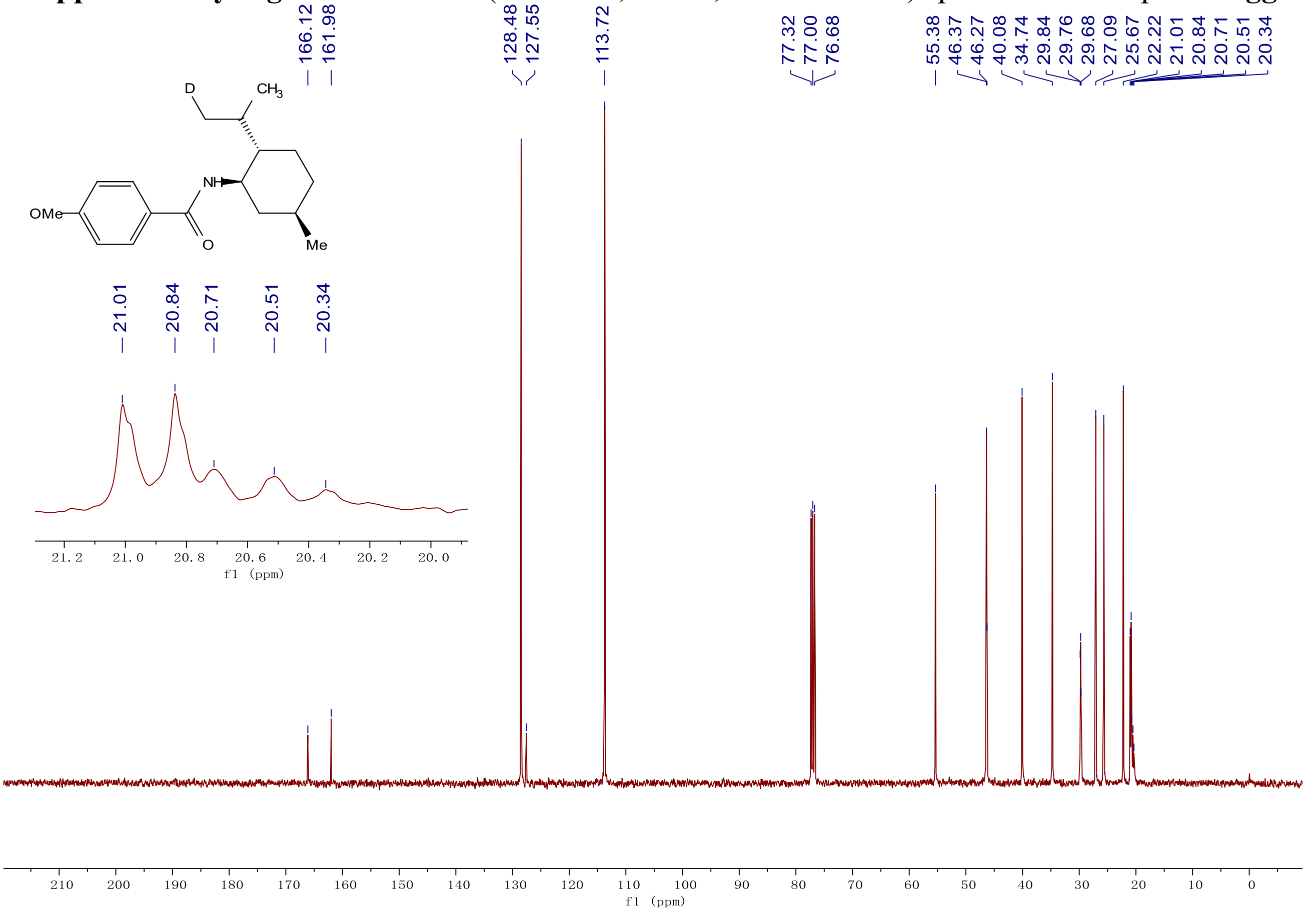

Supplementary Fig. 124. ^{13}C NMR (101 MHz, 298 K, Chloroform-*d*) spectrum of compound **3gg**.

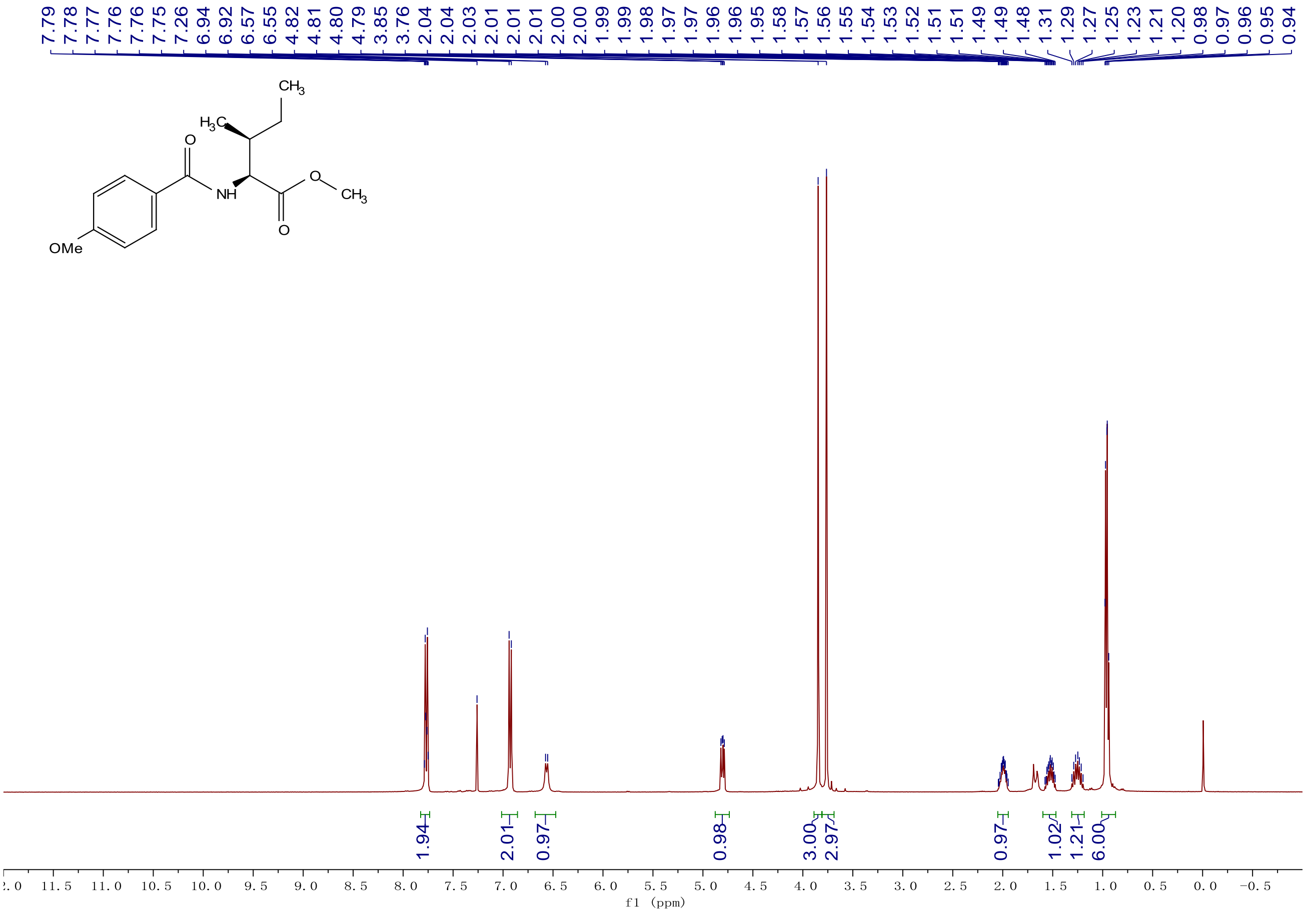

Supplementary Fig. 125. ^{1}H NMR (400 MHz, 298 K, Chloroform-*d*) spectrum of compound **1hh**.

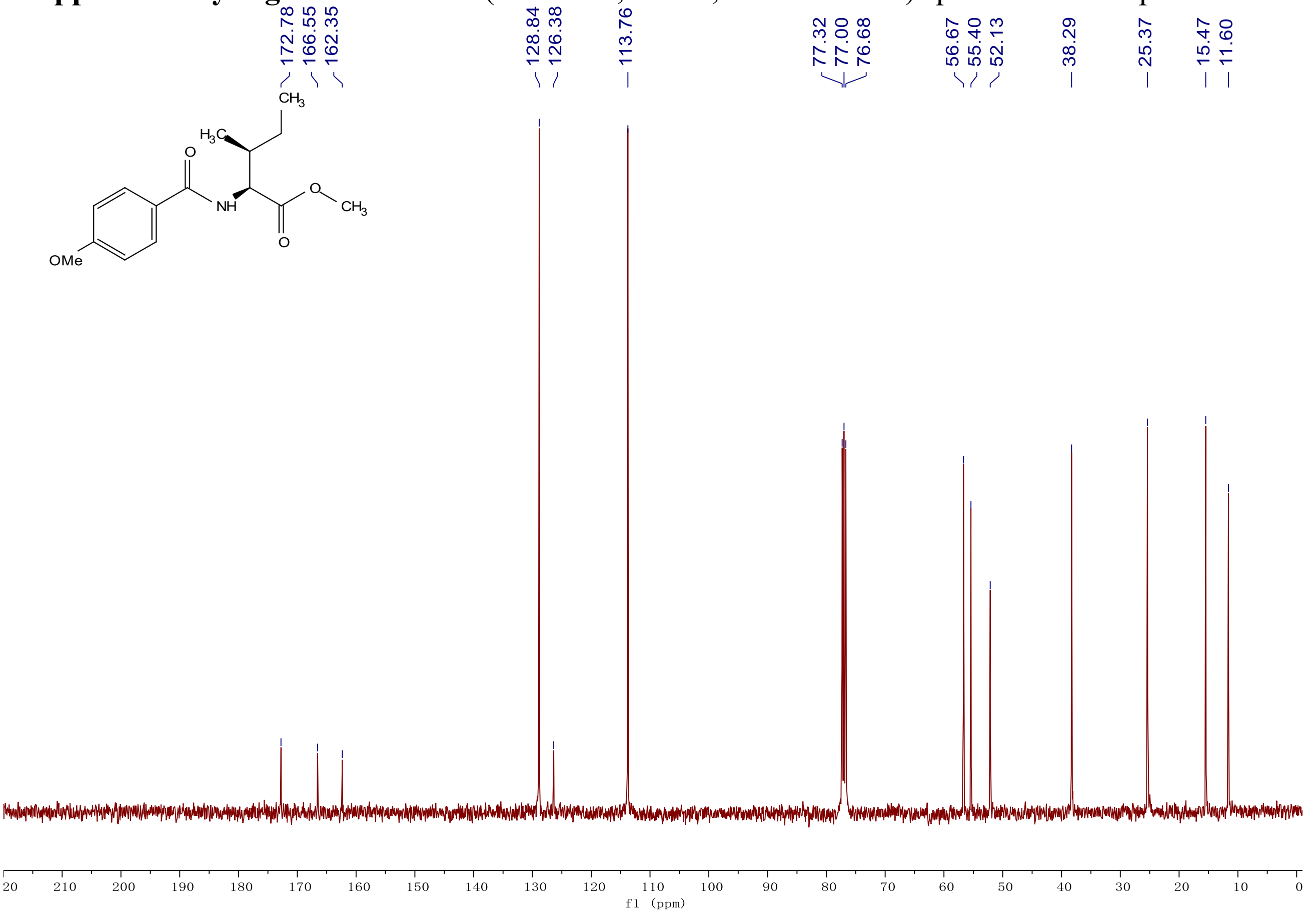

Supplementary Fig. 126. ^{13}C NMR (101 MHz, 298 K, Chloroform-*d*) spectrum of compound **1hh**.

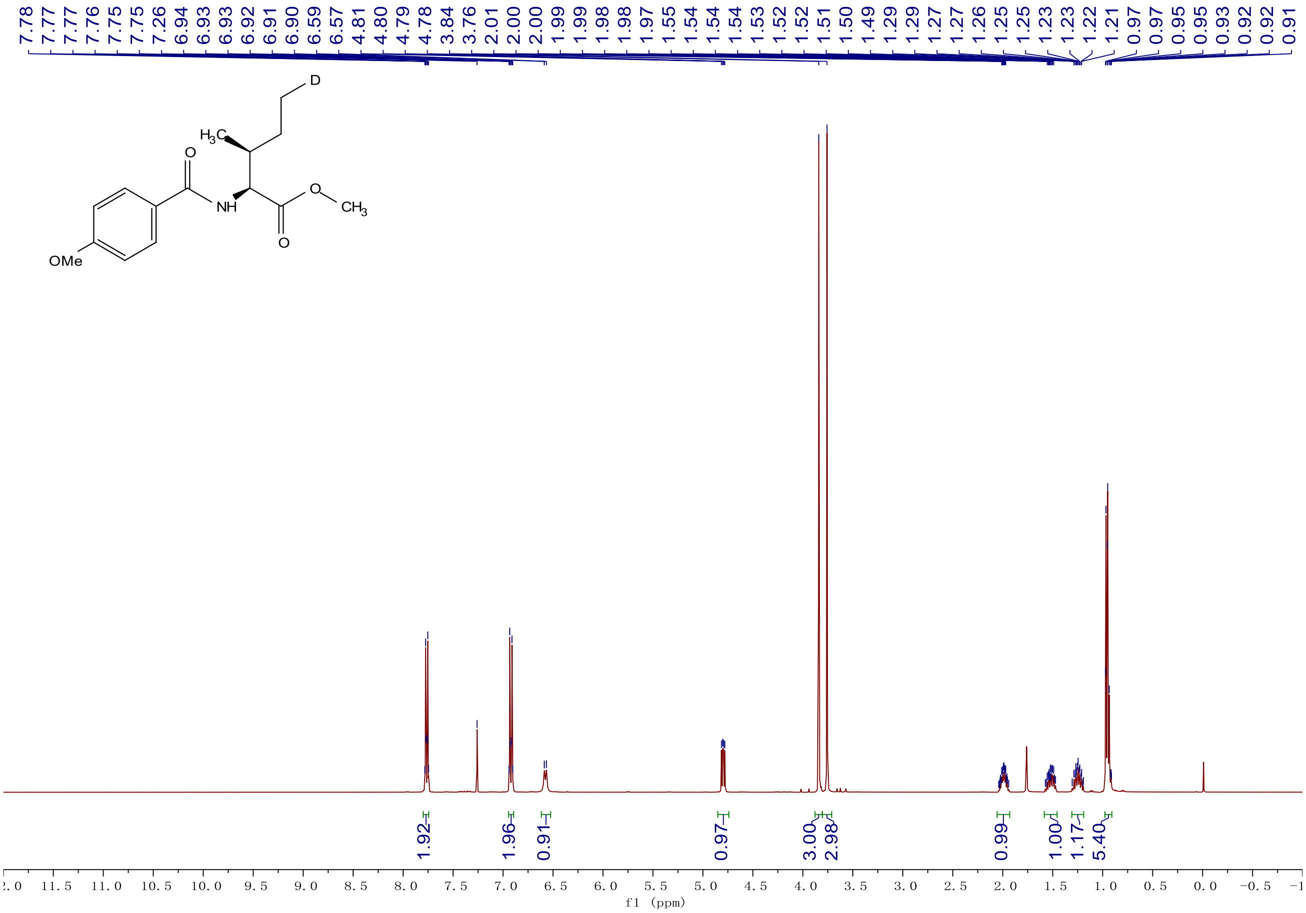

Supplementary Fig. 127. ^{1}H NMR (400 MHz, 298 K, Chloroform-*d*) spectrum of compound **3hh**.

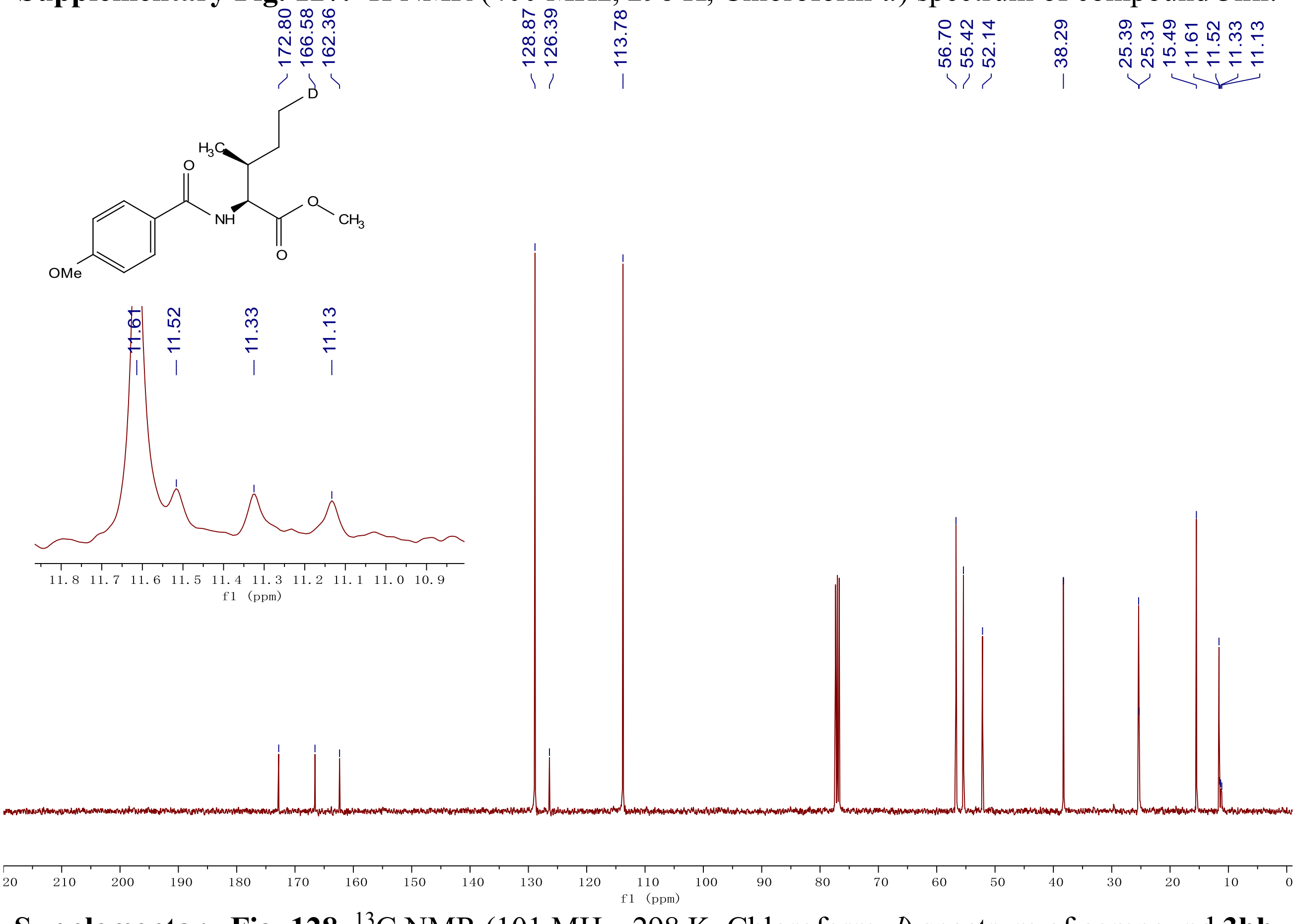

Supplementary Fig. 128. ^{13}C NMR (101 MHz, 298 K, Chloroform-*d*) spectrum of compound **3hh**.

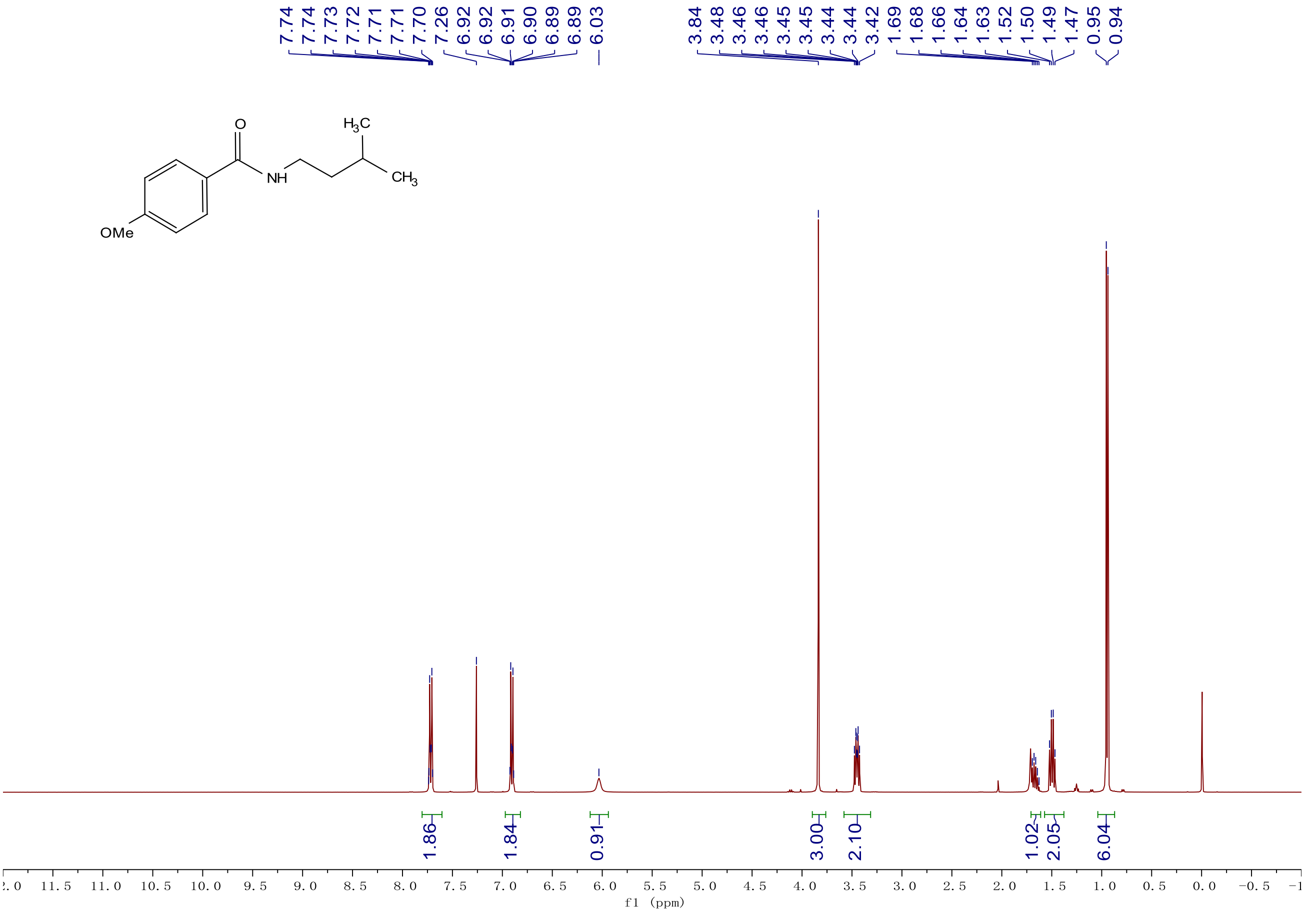

Supplementary Fig. 129. ^{1}H NMR (400 MHz, 298 K, Chloroform-*d*) spectrum of compound **1ii**.

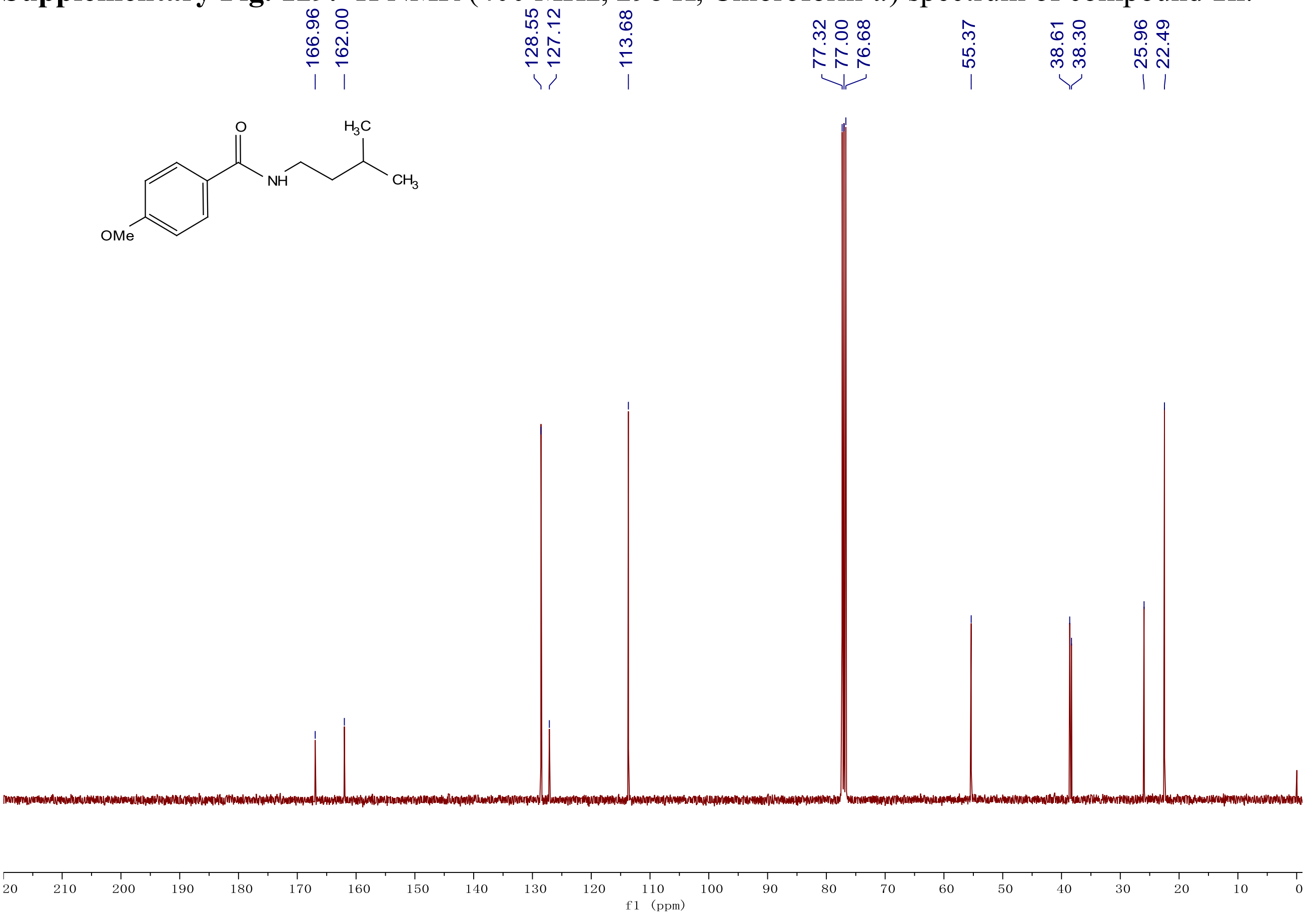

Supplementary Fig. 130. ^{13}C NMR (101 MHz, 298 K, Chloroform-*d*) spectrum of compound **1ii**.

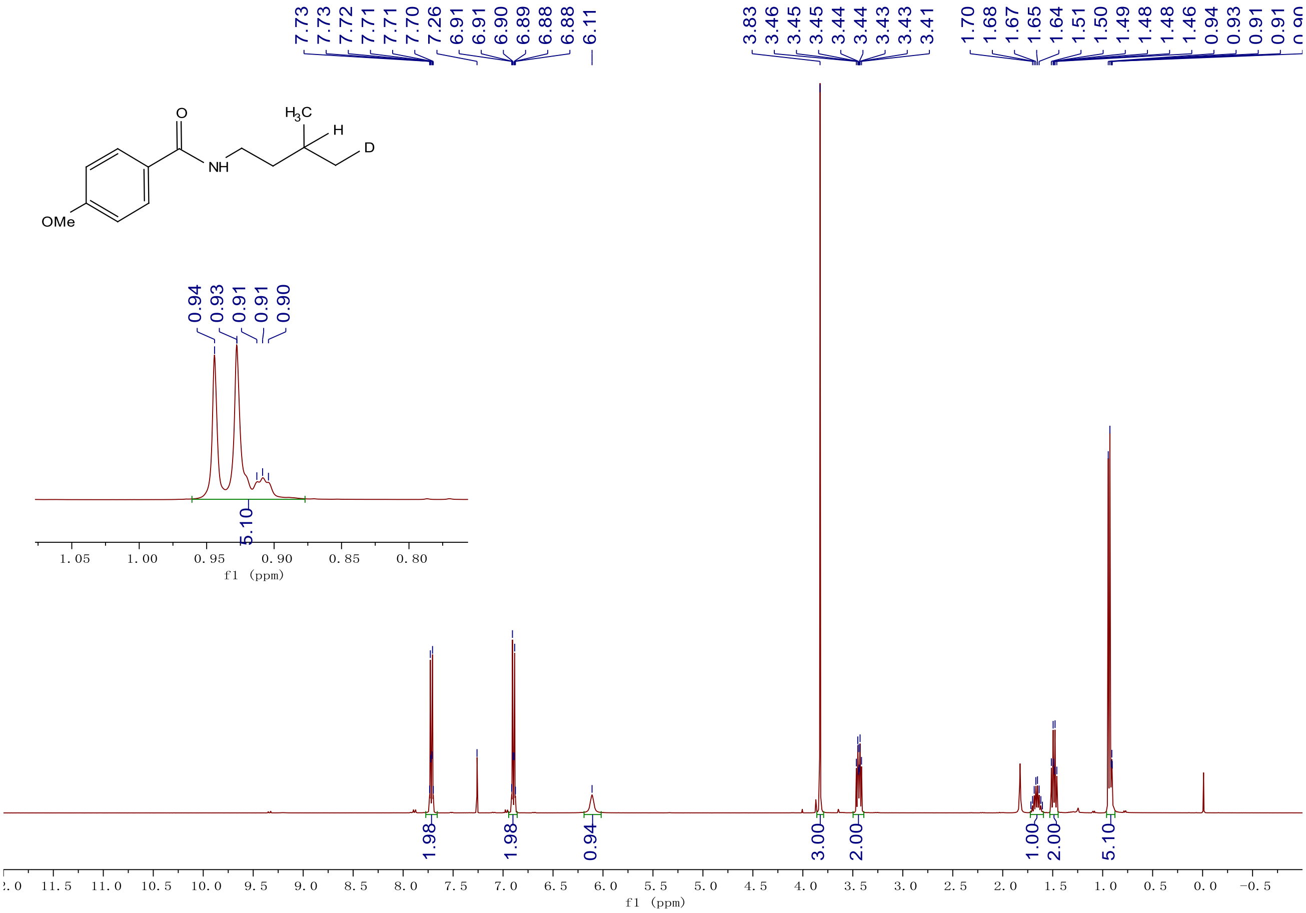

Supplementary Fig. 131. ^{1}H NMR (400 MHz, 298 K, Chloroform-*d*) spectrum of compound **3ii**.

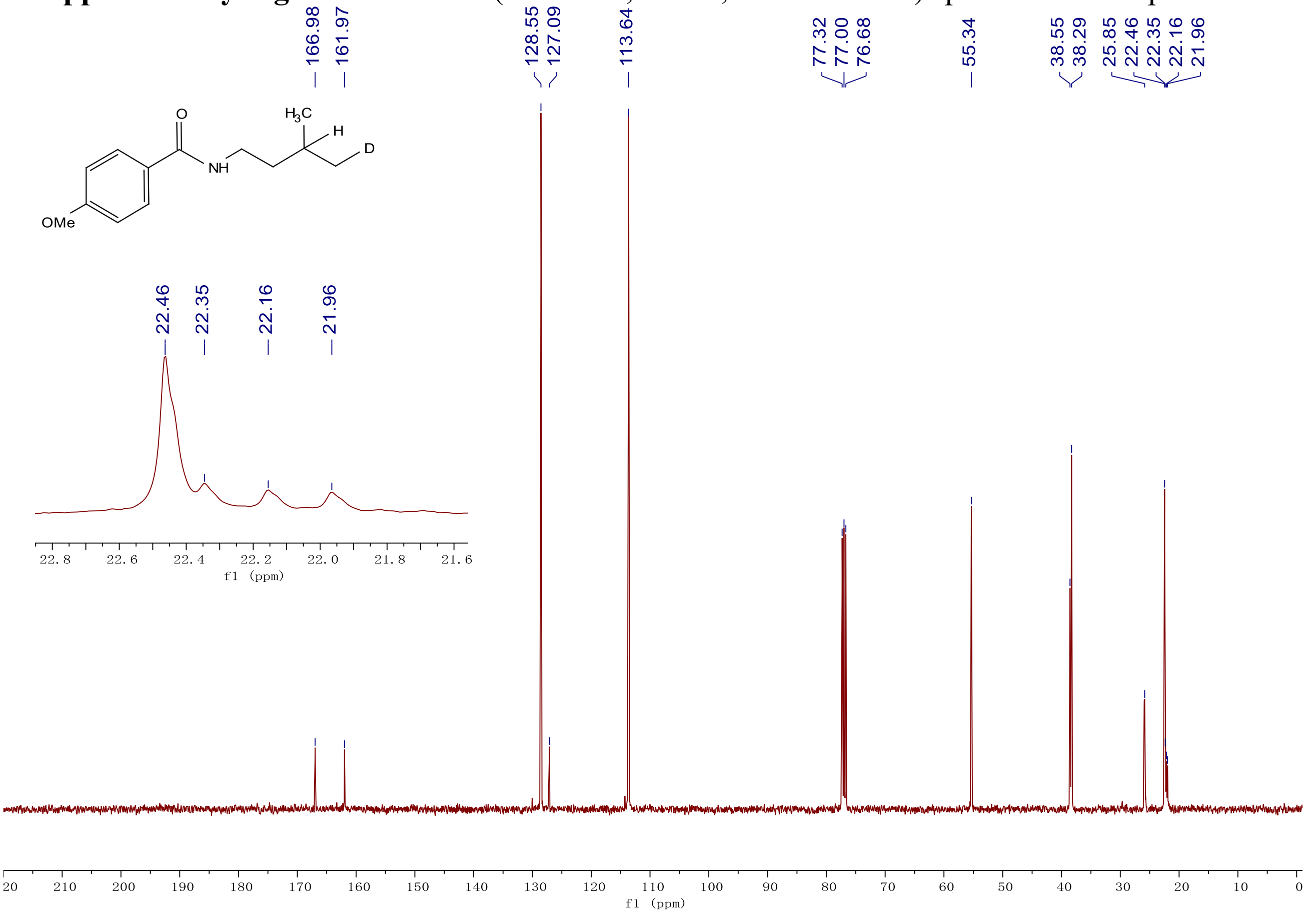

Supplementary Fig. 132. ^{13}C NMR (101 MHz, 298 K, Chloroform-*d*) spectrum of compound **3ii**.

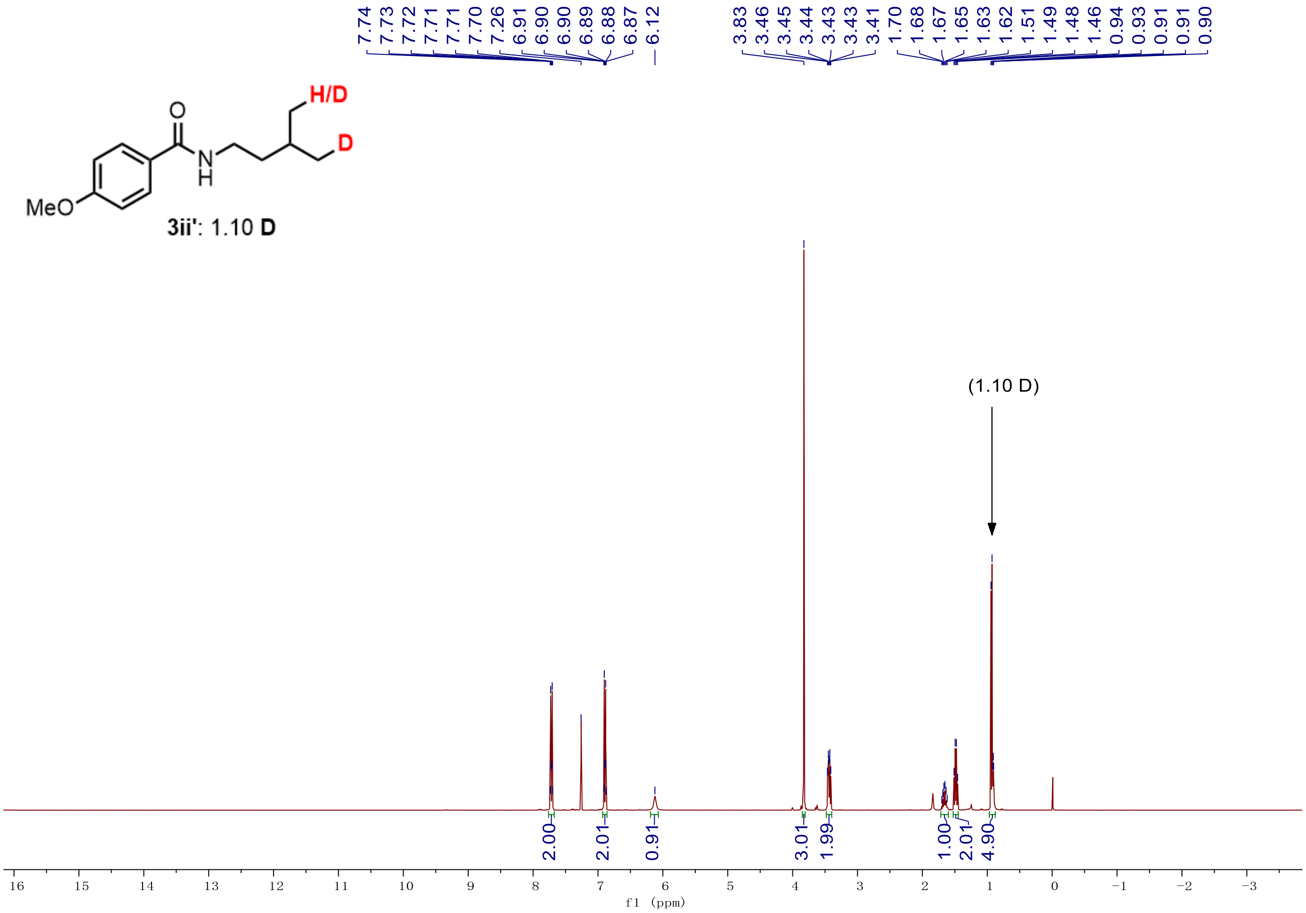

Supplementary Fig. 133. ^{1}H NMR (400 MHz, 298 K, Chloroform-*d*) spectrum of compound **3ii'**.

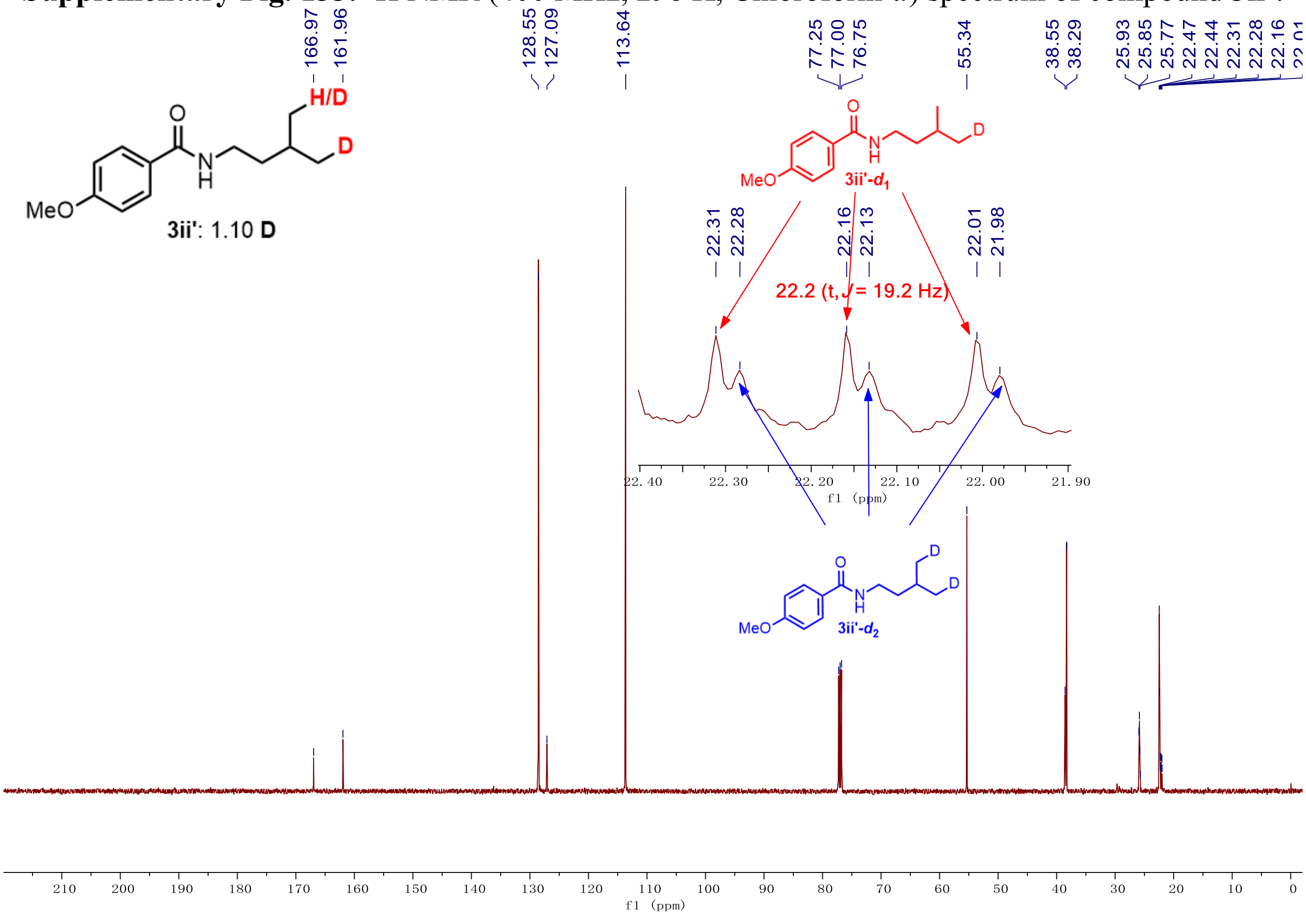

Supplementary Fig. 134. ^{13}C NMR (126 MHz, 298 K, Chloroform-*d*) spectrum of compound **3ii'**.

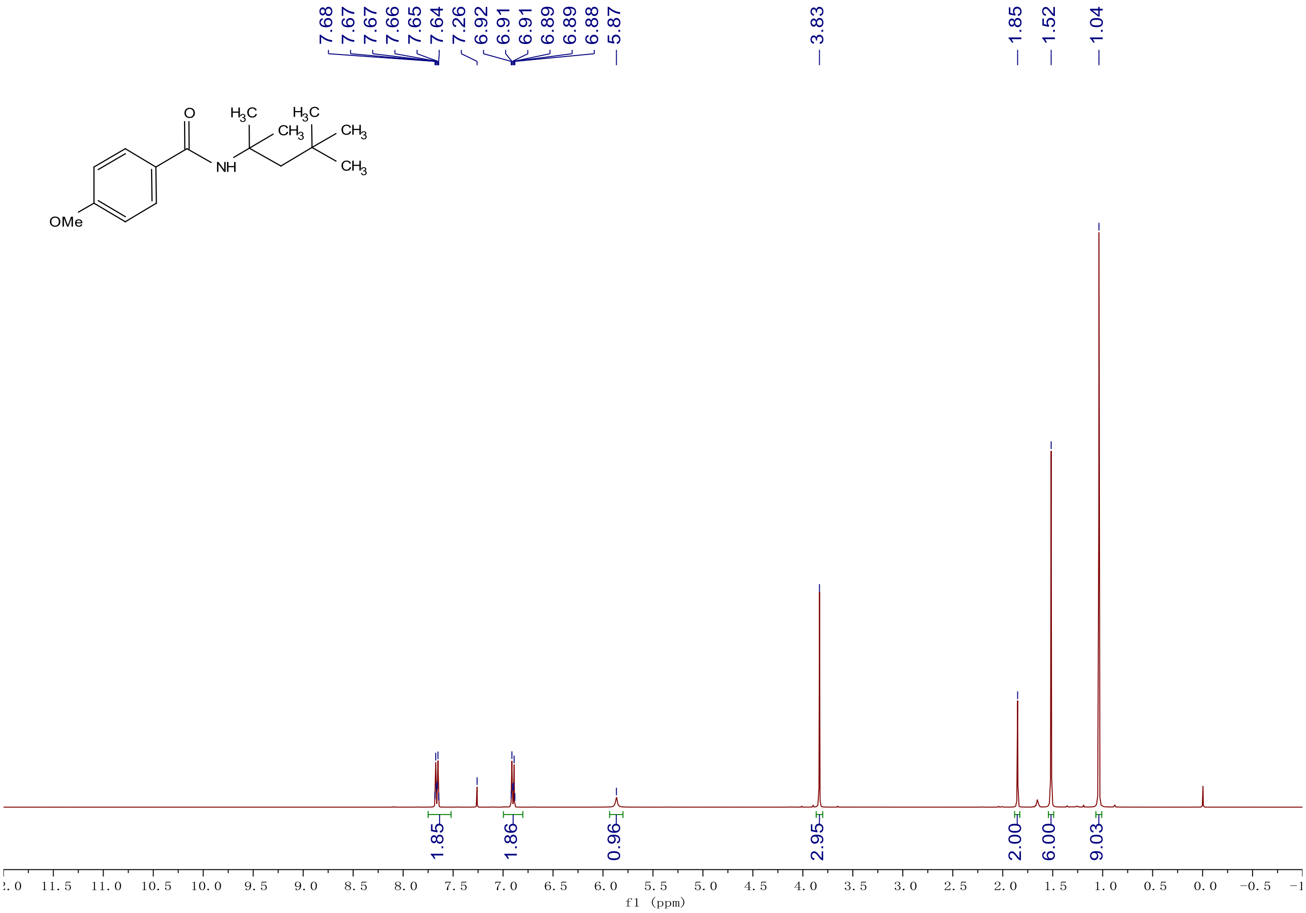

Supplementary Fig. 135. ^{1}H NMR (400 MHz, 298 K, Chloroform-*d*) spectrum of compound **1jj**.

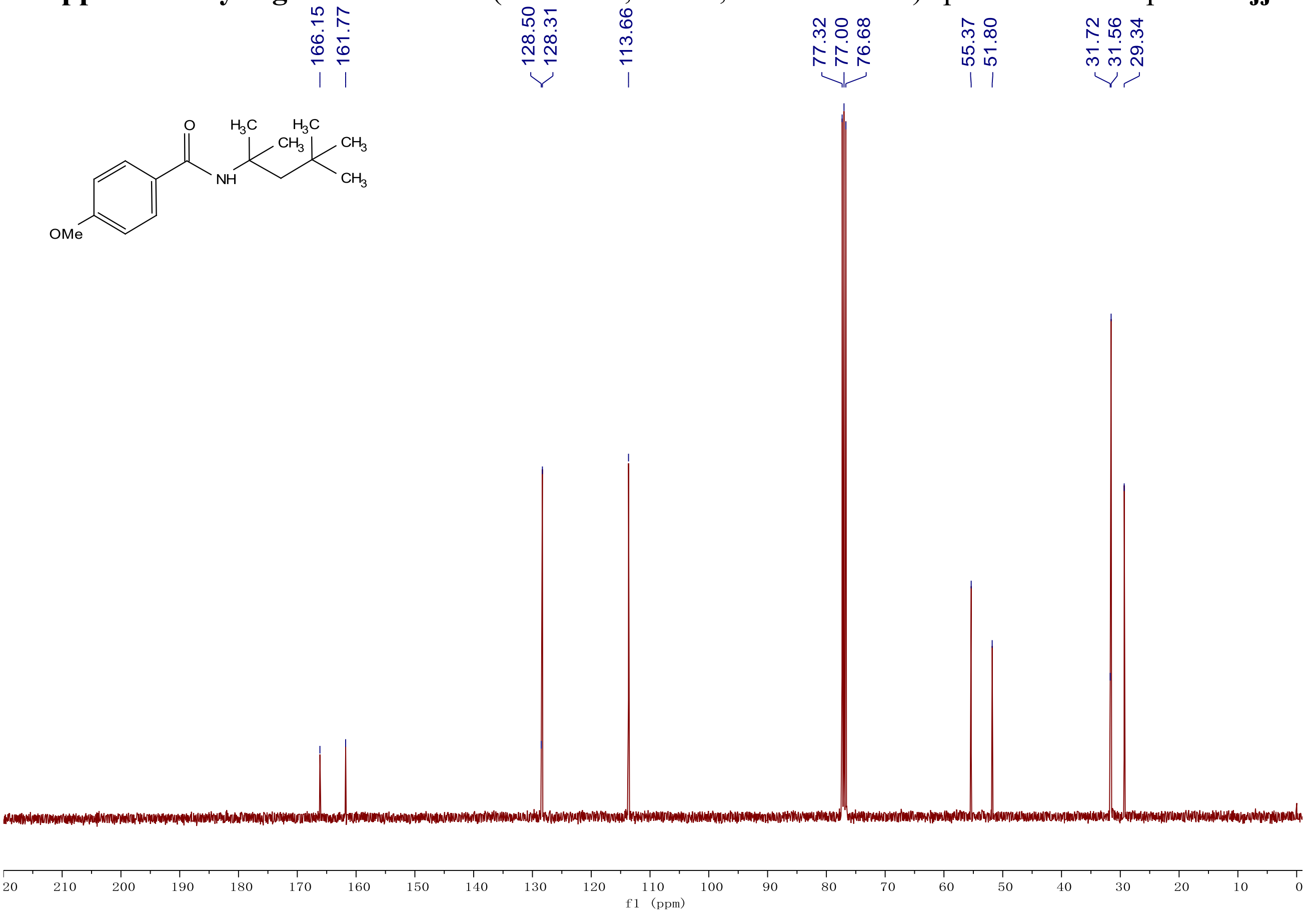

Supplementary Fig. 136. ^{13}C NMR (101 MHz, 298 K, Chloroform-*d*) spectrum of compound **1jj**.

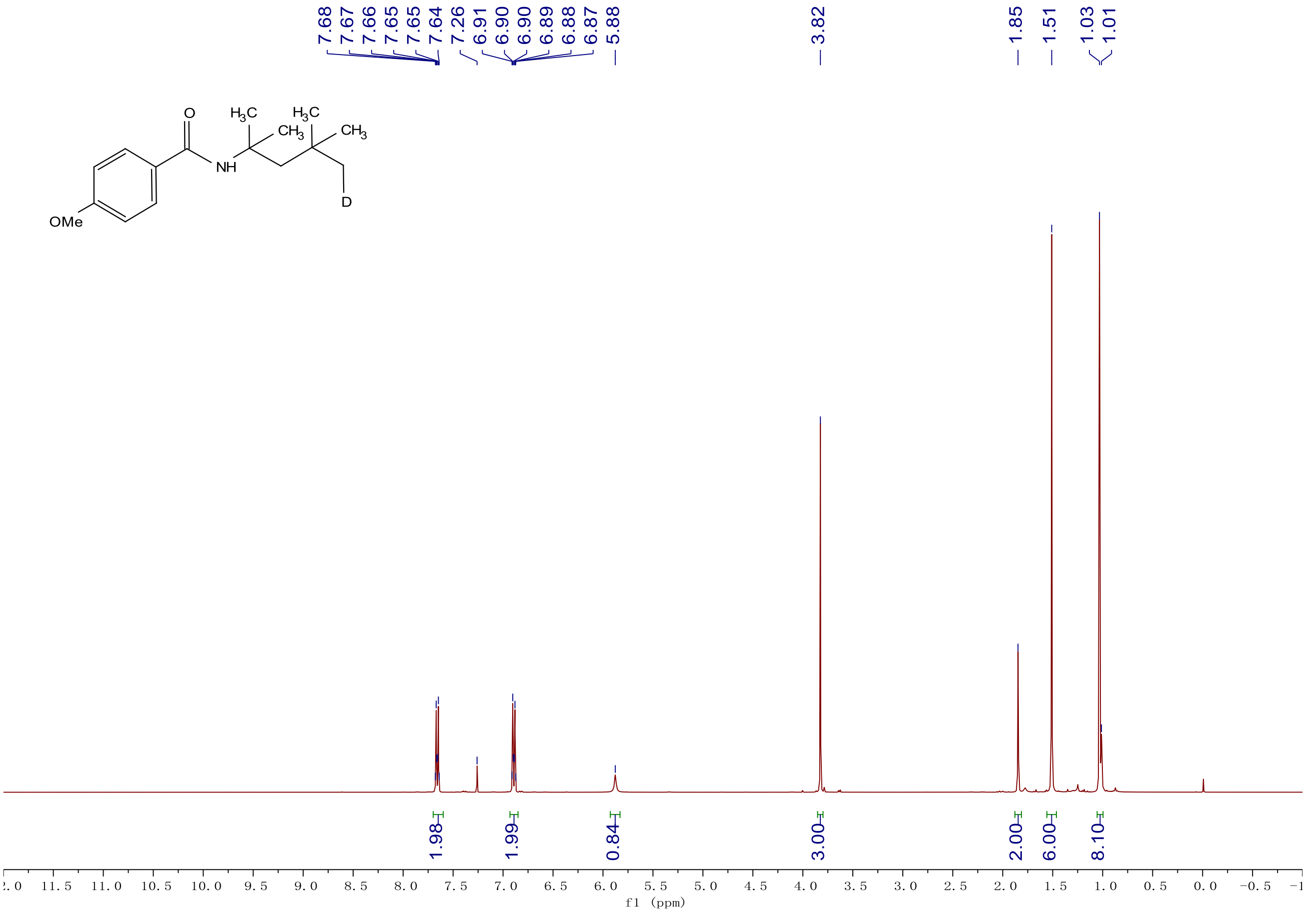

Supplementary Fig. 137. ^{1}H NMR (400 MHz, 298 K, Chloroform-*d*) spectrum of compound **3jj**.

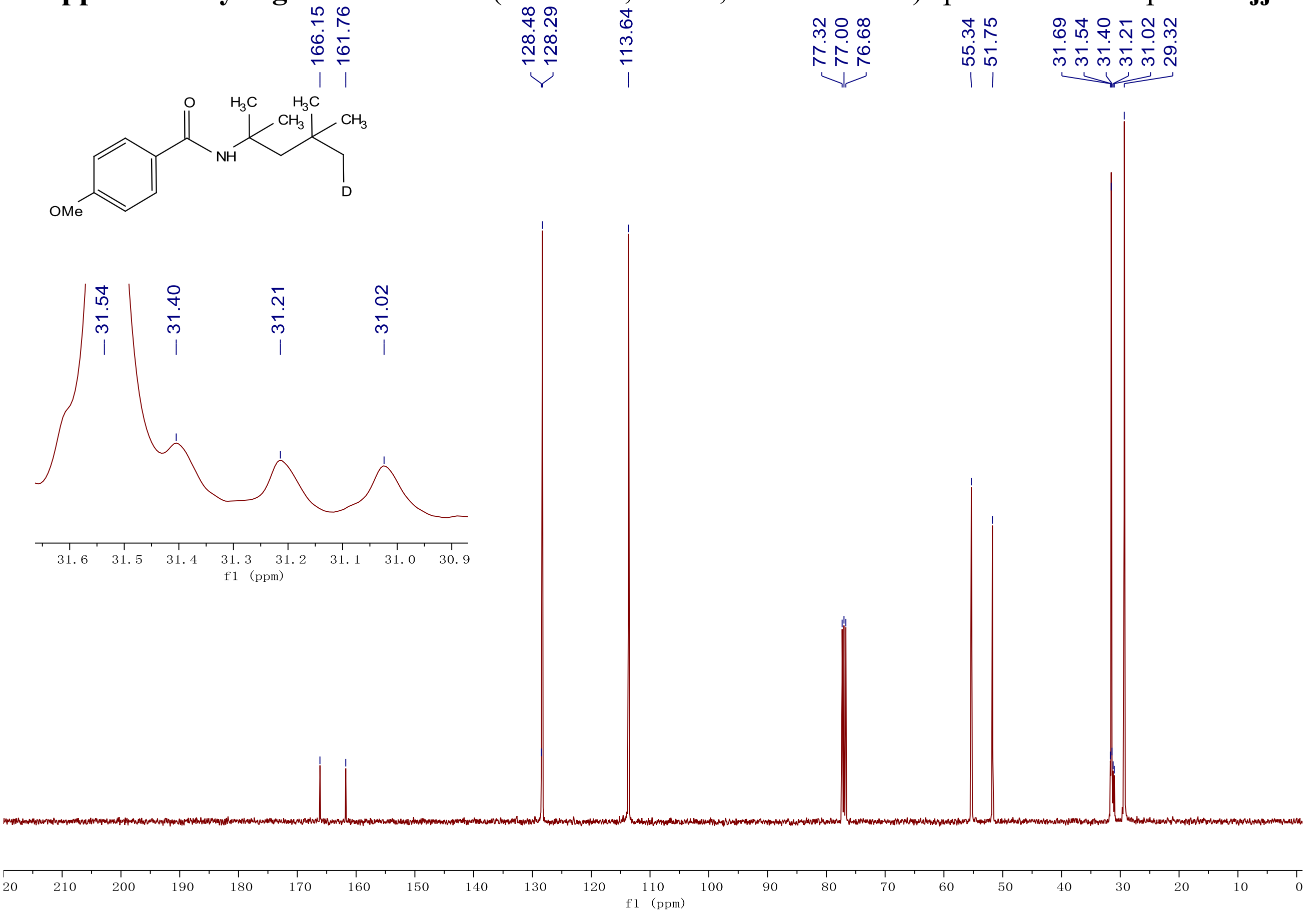

Supplementary Fig. 138. ^{13}C NMR (101 MHz, 298 K, Chloroform-*d*) spectrum of compound **3jj**.

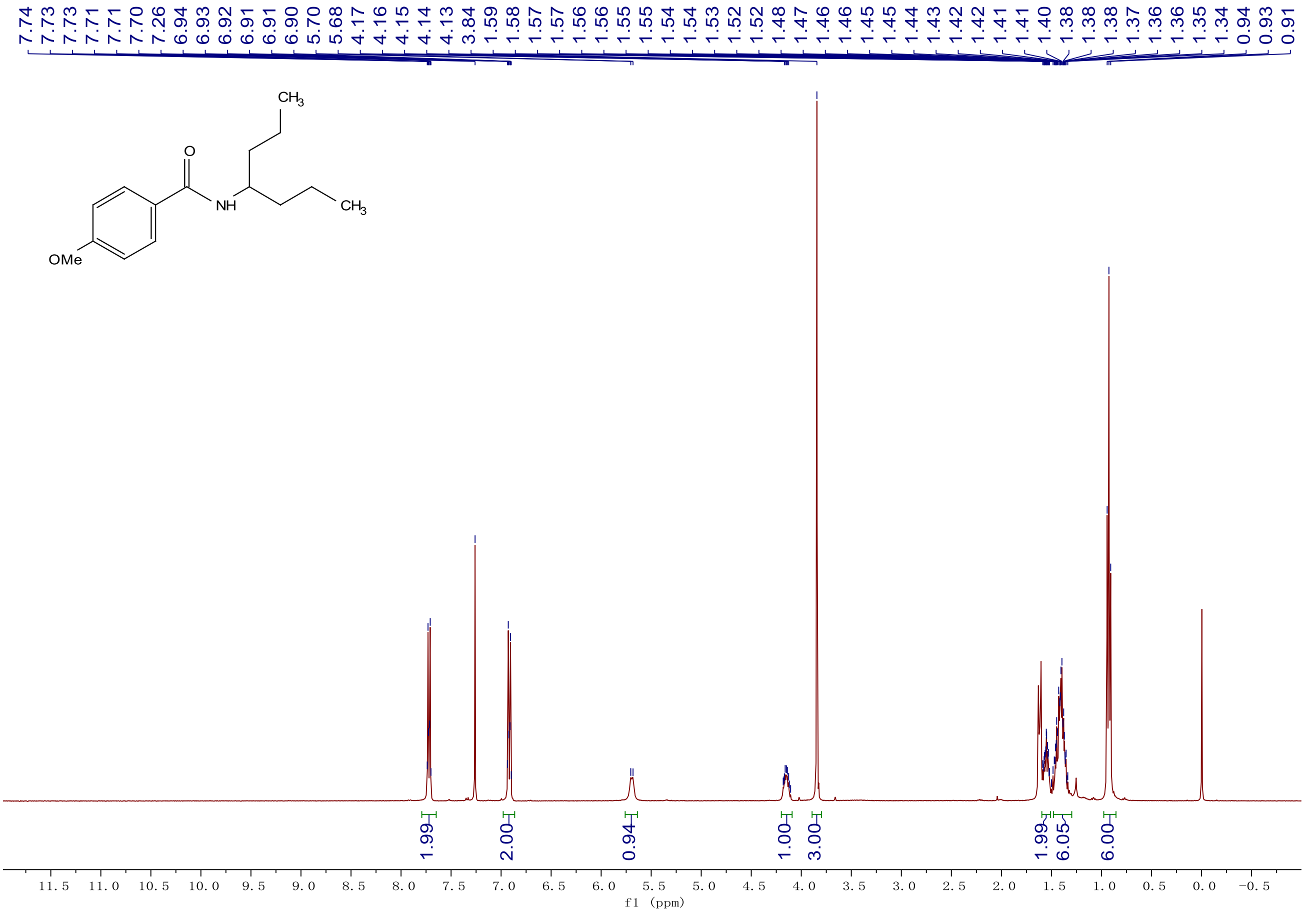

Supplementary Fig. 139. ^{1}H NMR (400 MHz, 298 K, Chloroform-*d*) spectrum of compound **1kk**.

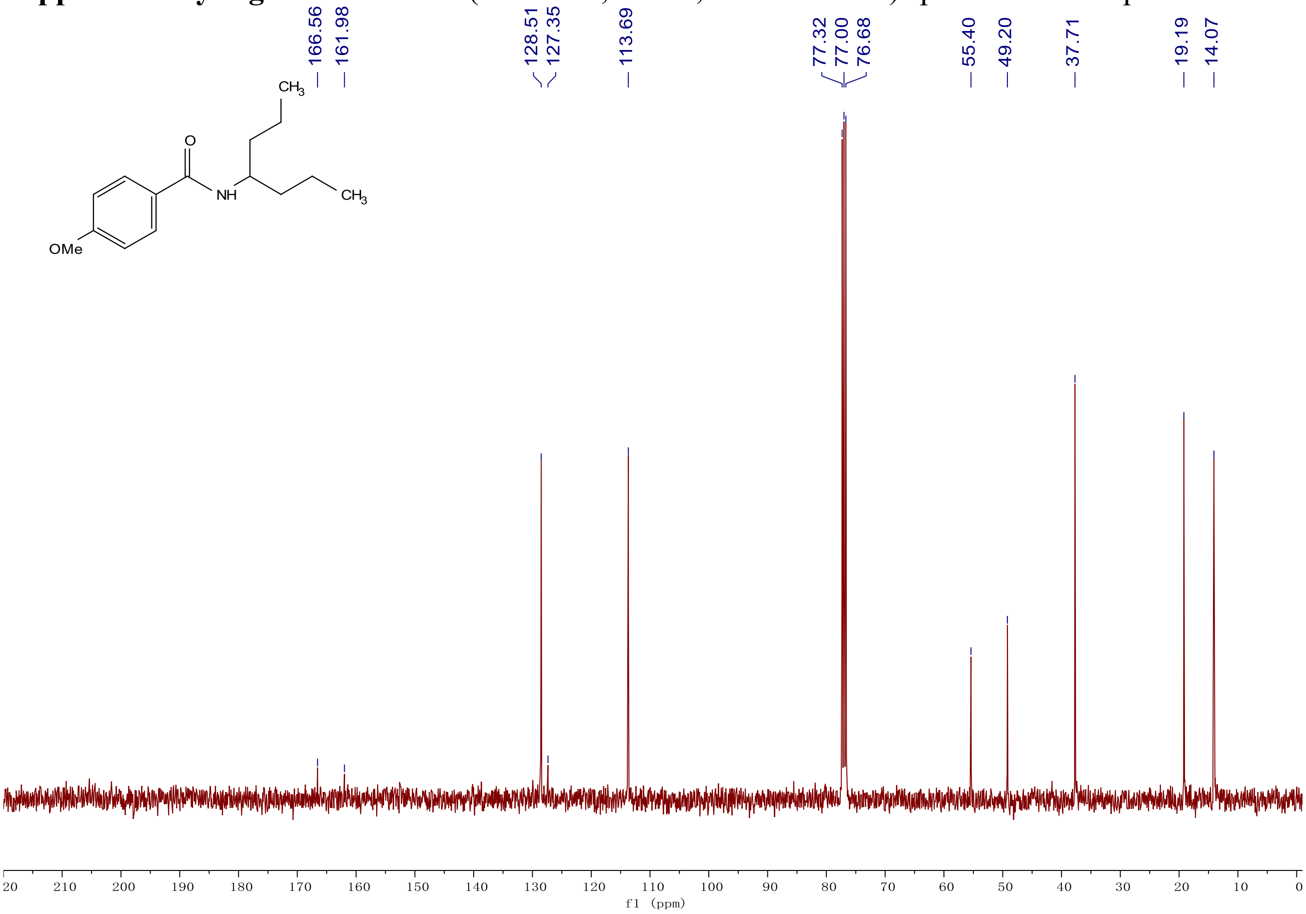

Supplementary Fig. 140. ^{13}C NMR (101 MHz, 298 K, Chloroform-*d*) spectrum of compound **1kk**.

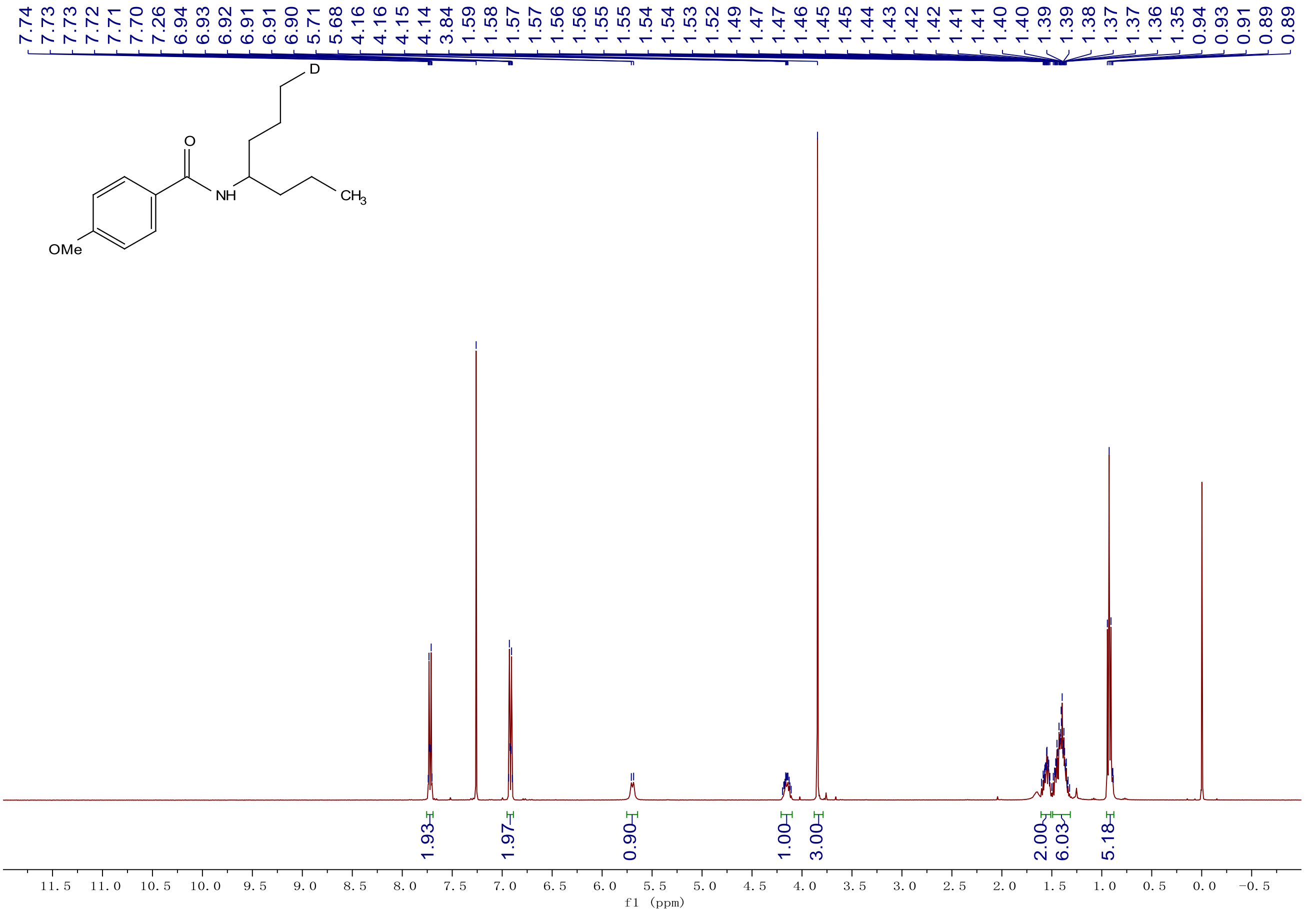

Supplementary Fig. 141. ^{1}H NMR (400 MHz, 298 K, Chloroform-*d*) spectrum of compound **3kk**.

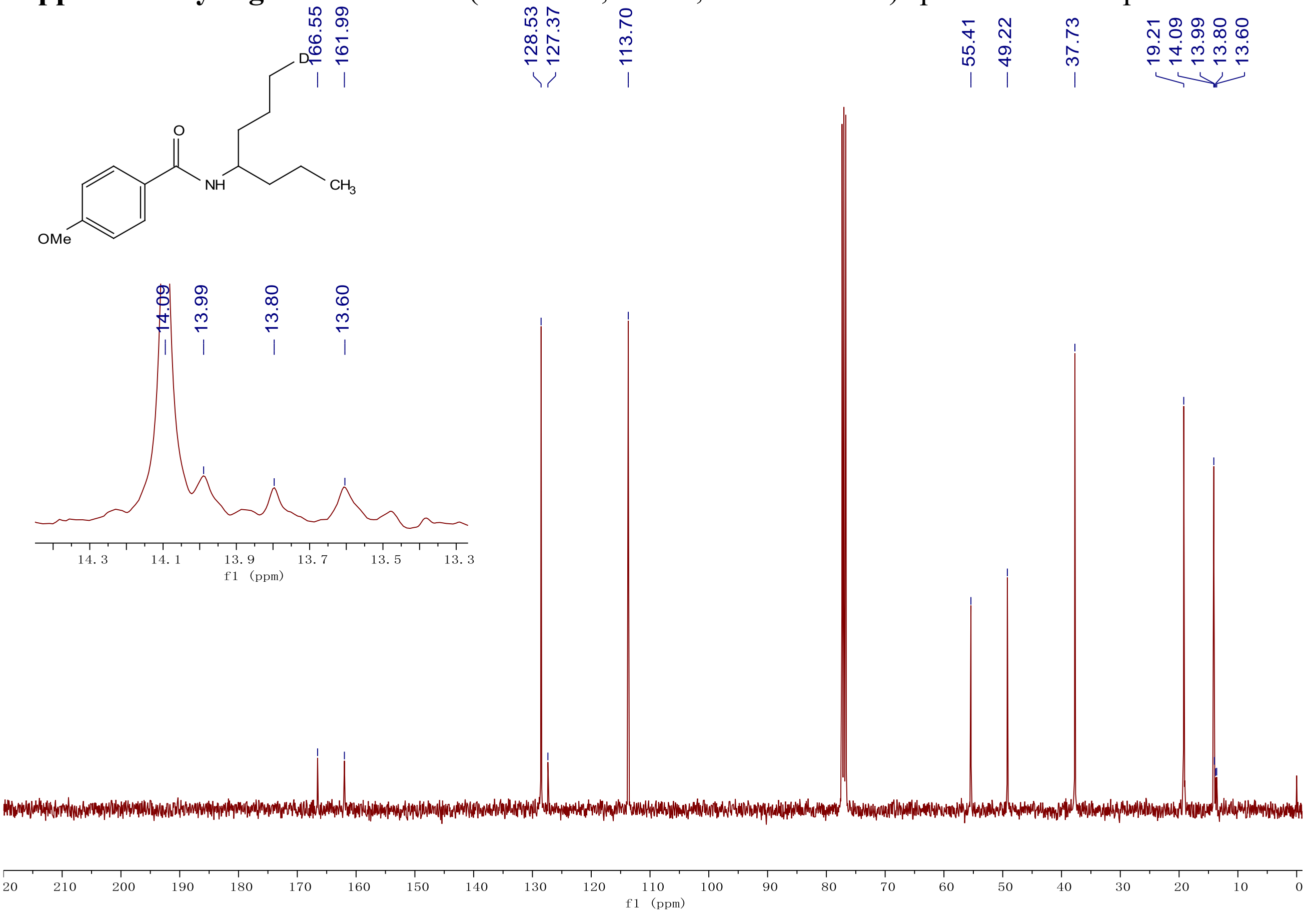

Supplementary Fig. 142. ^{13}C NMR (101 MHz, 298 K, Chloroform-*d*) spectrum of compound **3kk**.

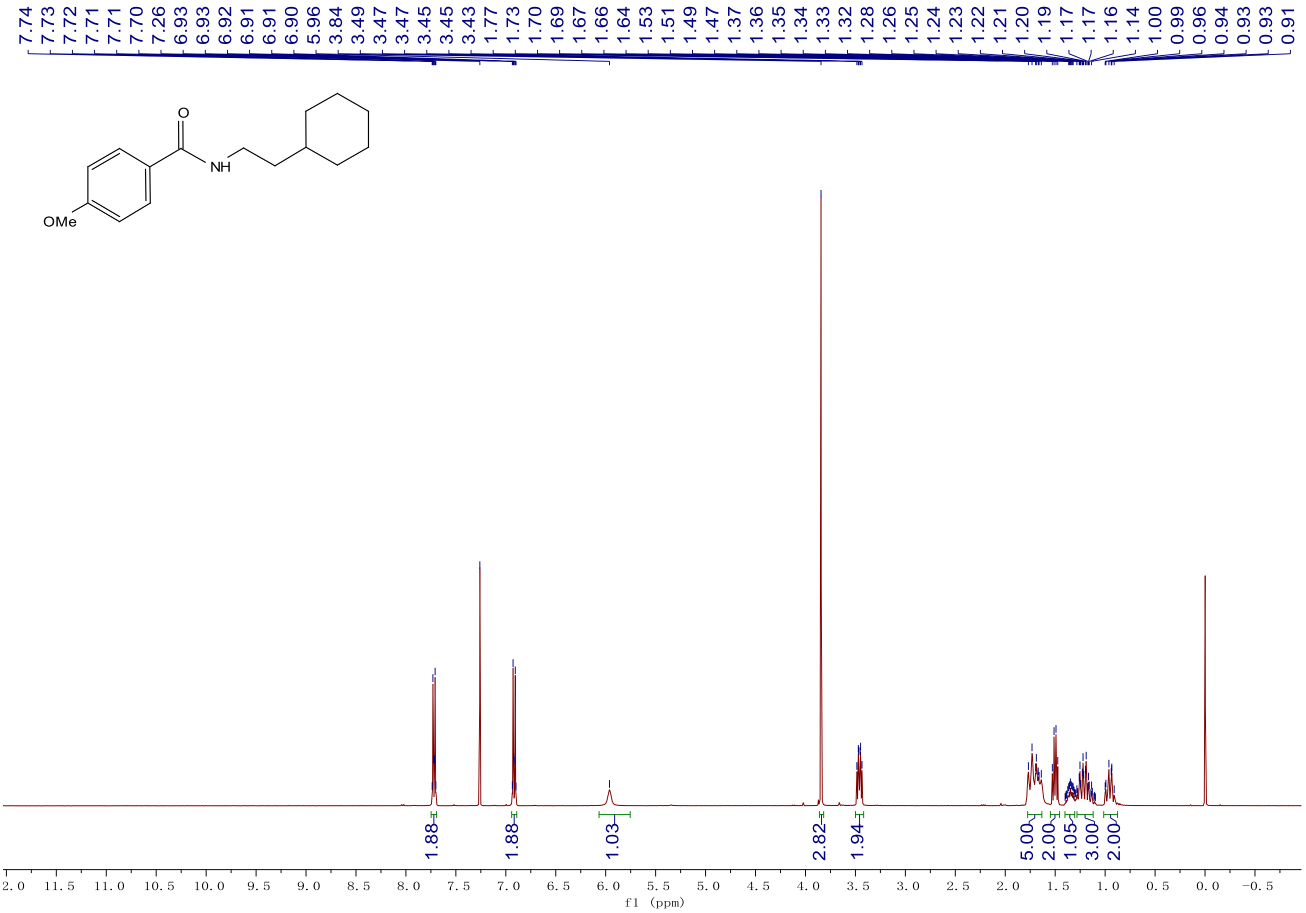

Supplementary Fig. 143. ^{1}H NMR (400 MHz, 298 K, Chloroform-*d*) spectrum of compound **1-a**.

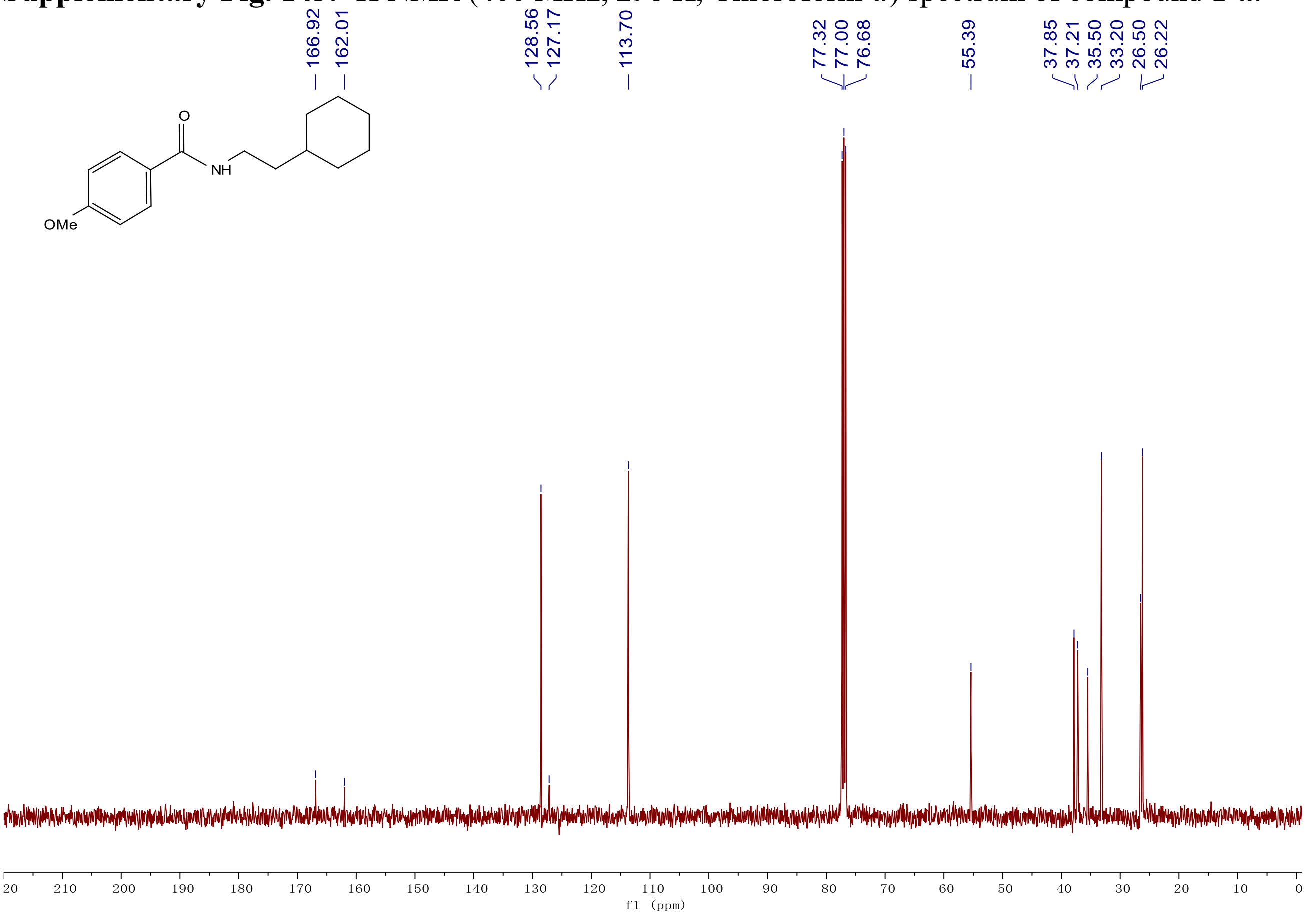

Supplementary Fig. 144. ^{13}C NMR (101 MHz, 298 K, Chloroform-*d*) spectrum of compound **1-a**.

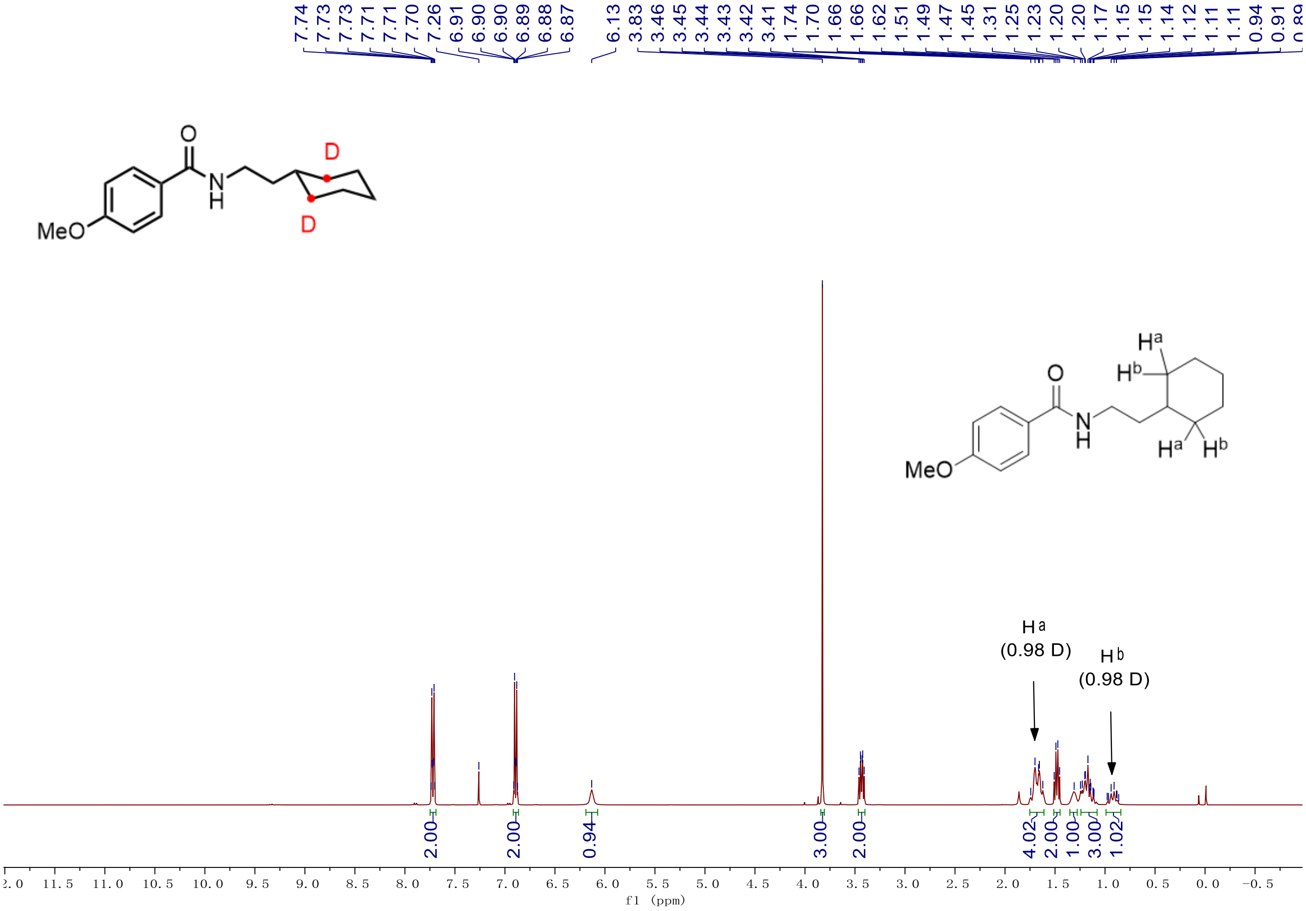

Supplementary Fig. 145. ^{1}H NMR (400 MHz, 298 K, Chloroform-*d*) spectrum of compound **4a**.

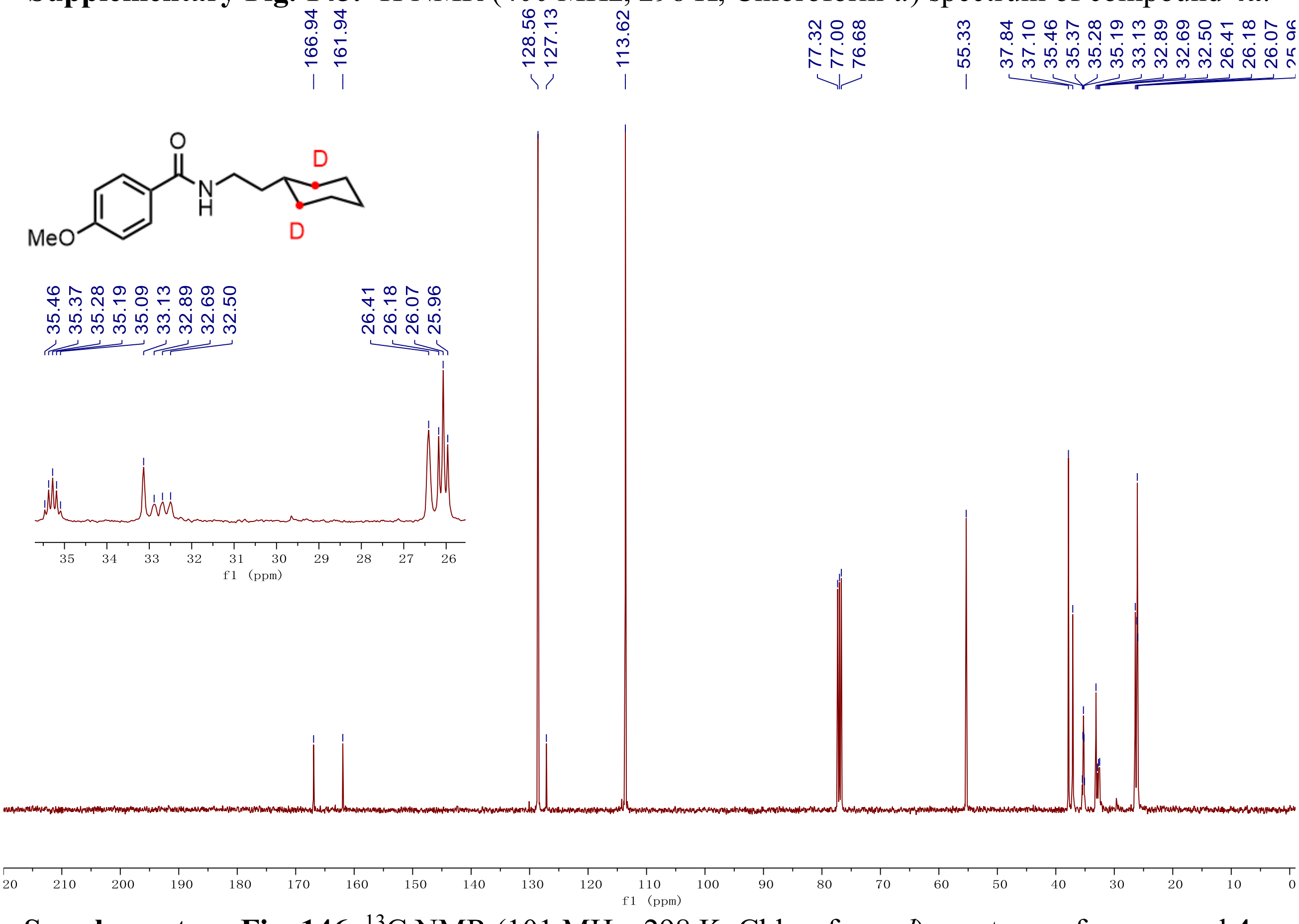

Supplementary Fig. 146. ^{13}C NMR (101 MHz, 298 K, Chloroform-*d*) spectrum of compound **4a**.

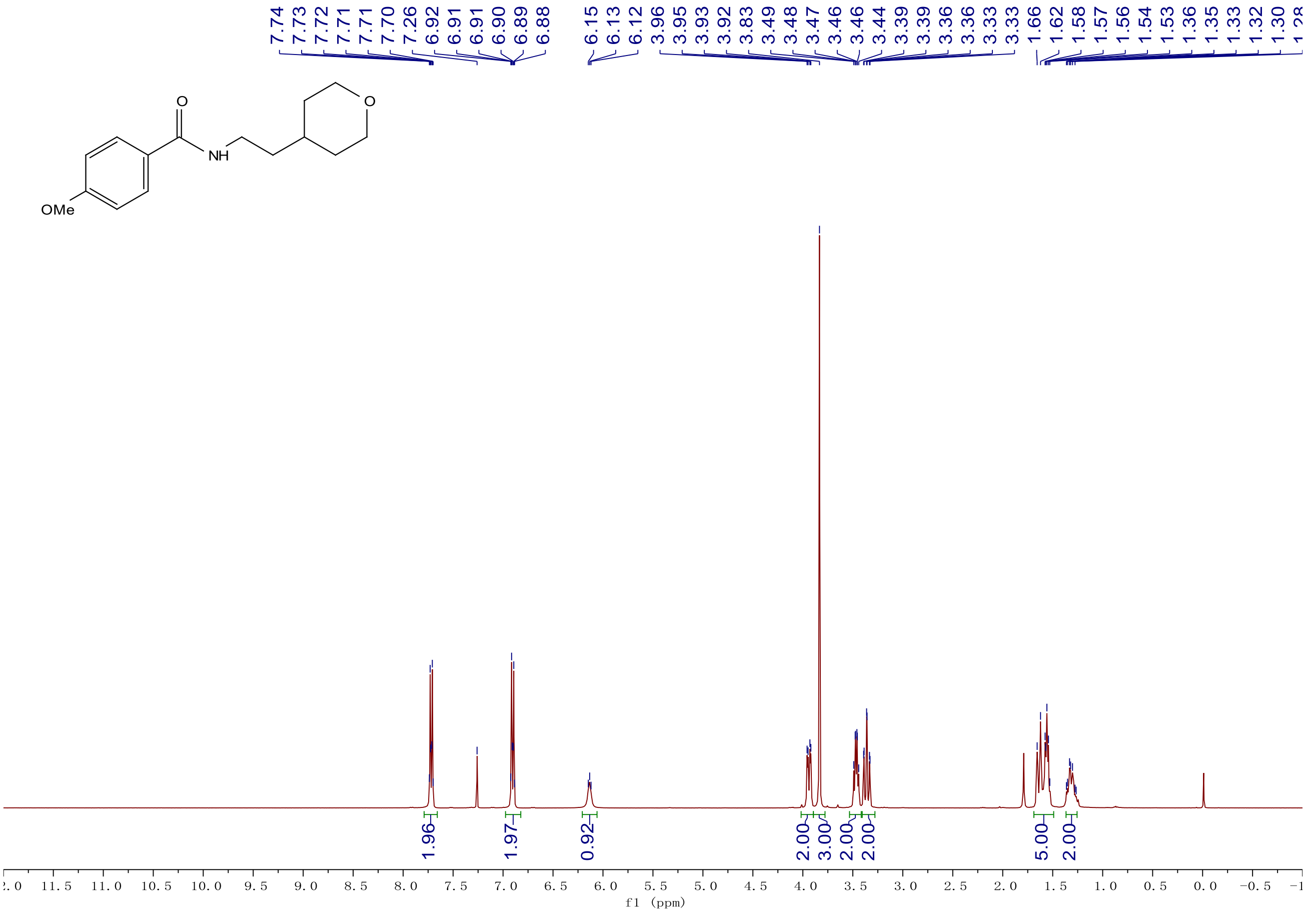

Supplementary Fig. 147. ^{1}H NMR (400 MHz, 298 K, Chloroform-*d*) spectrum of compound **1-b**.

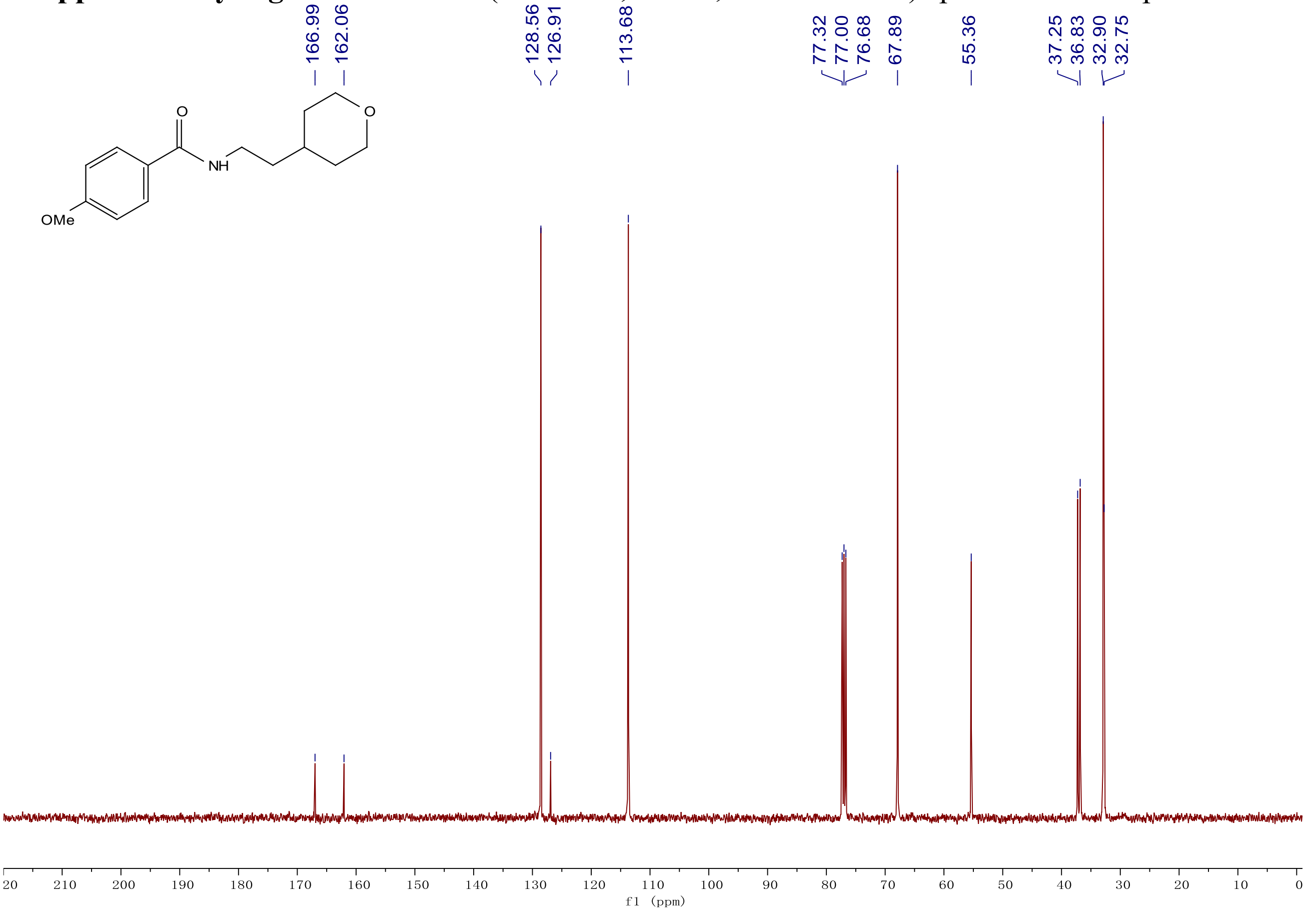

Supplementary Fig. 148. ^{13}C NMR (101 MHz, 298 K, Chloroform-*d*) spectrum of compound **1-b**.

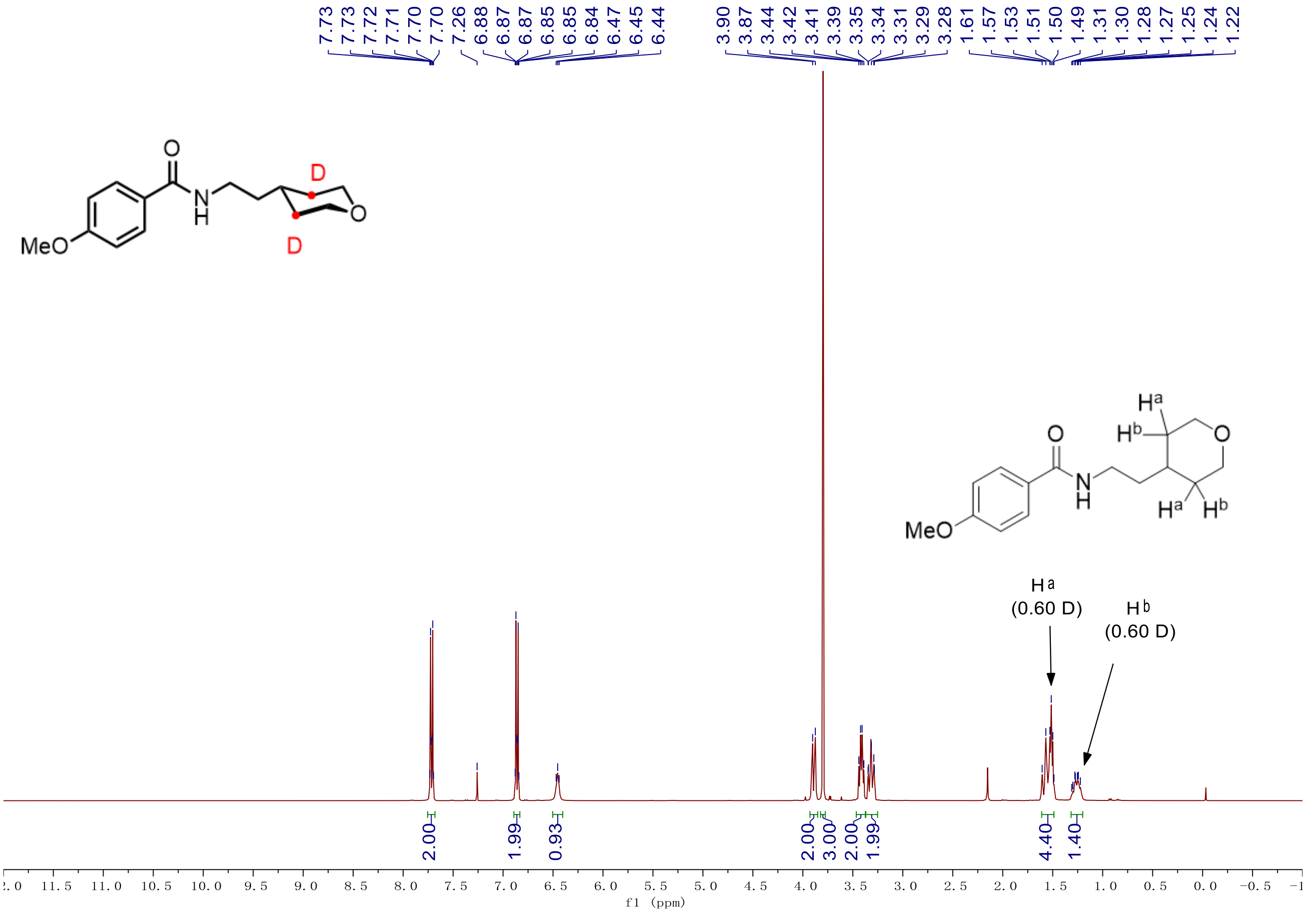

Supplementary Fig. 149. ^{1}H NMR (400 MHz, 298 K, Chloroform-*d*) spectrum of compound **4b**.

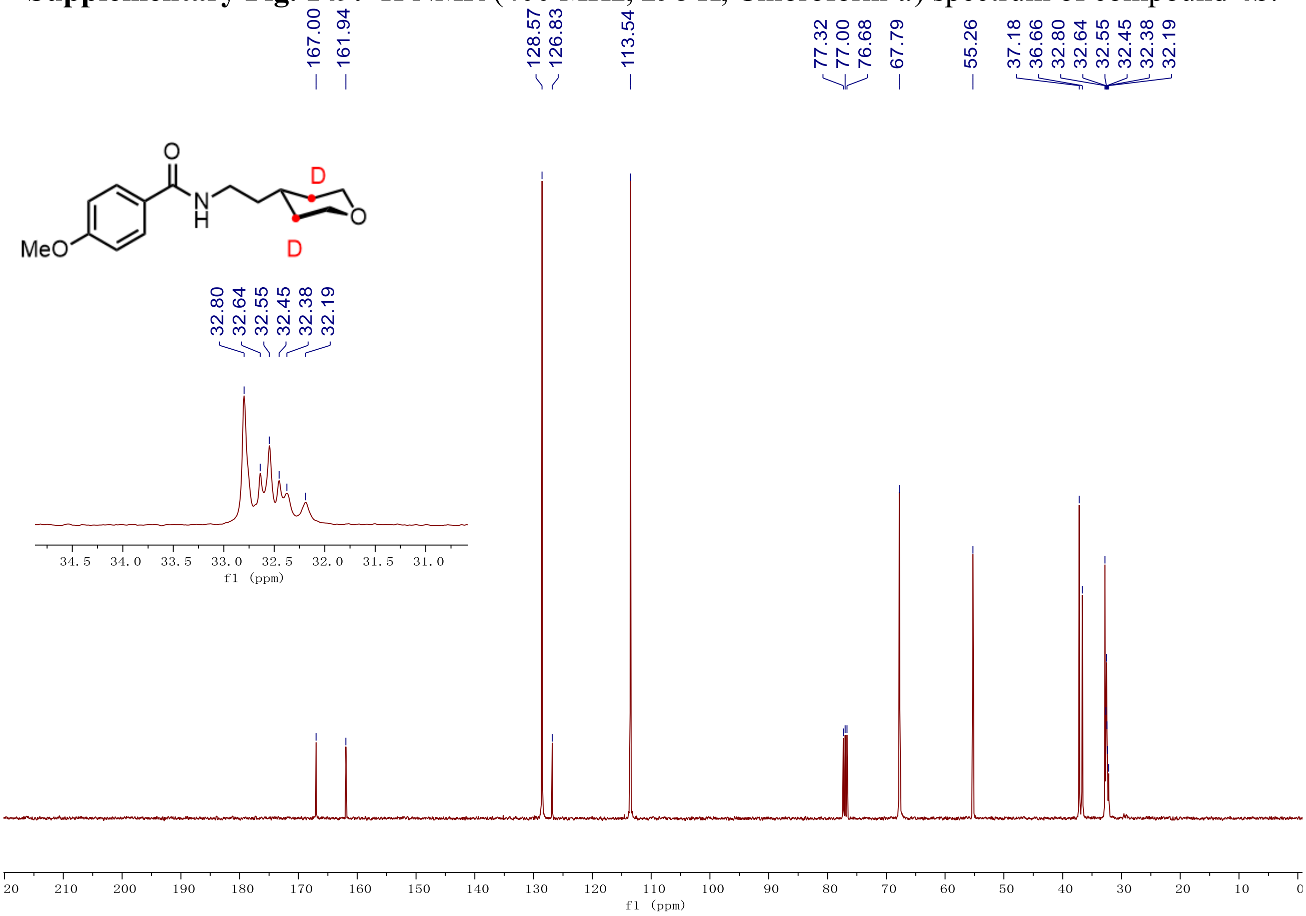

Supplementary Fig. 150. ^{13}C NMR (101 MHz, 298 K, Chloroform-*d*) spectrum of compound **4b**.

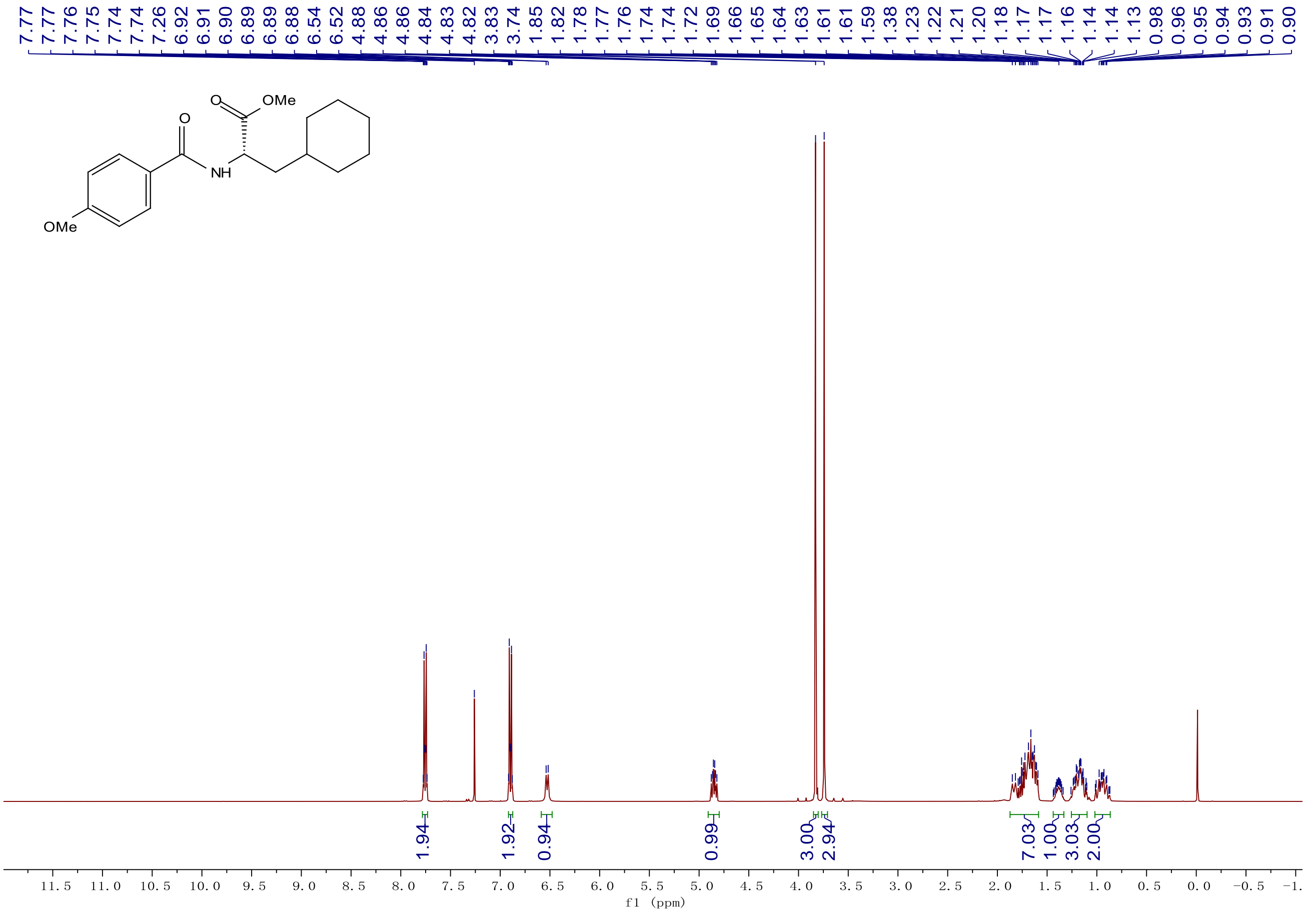

Supplementary Fig. 151. ^{1}H NMR (400 MHz, 298 K, Chloroform-*d*) spectrum of compound **1-c**.

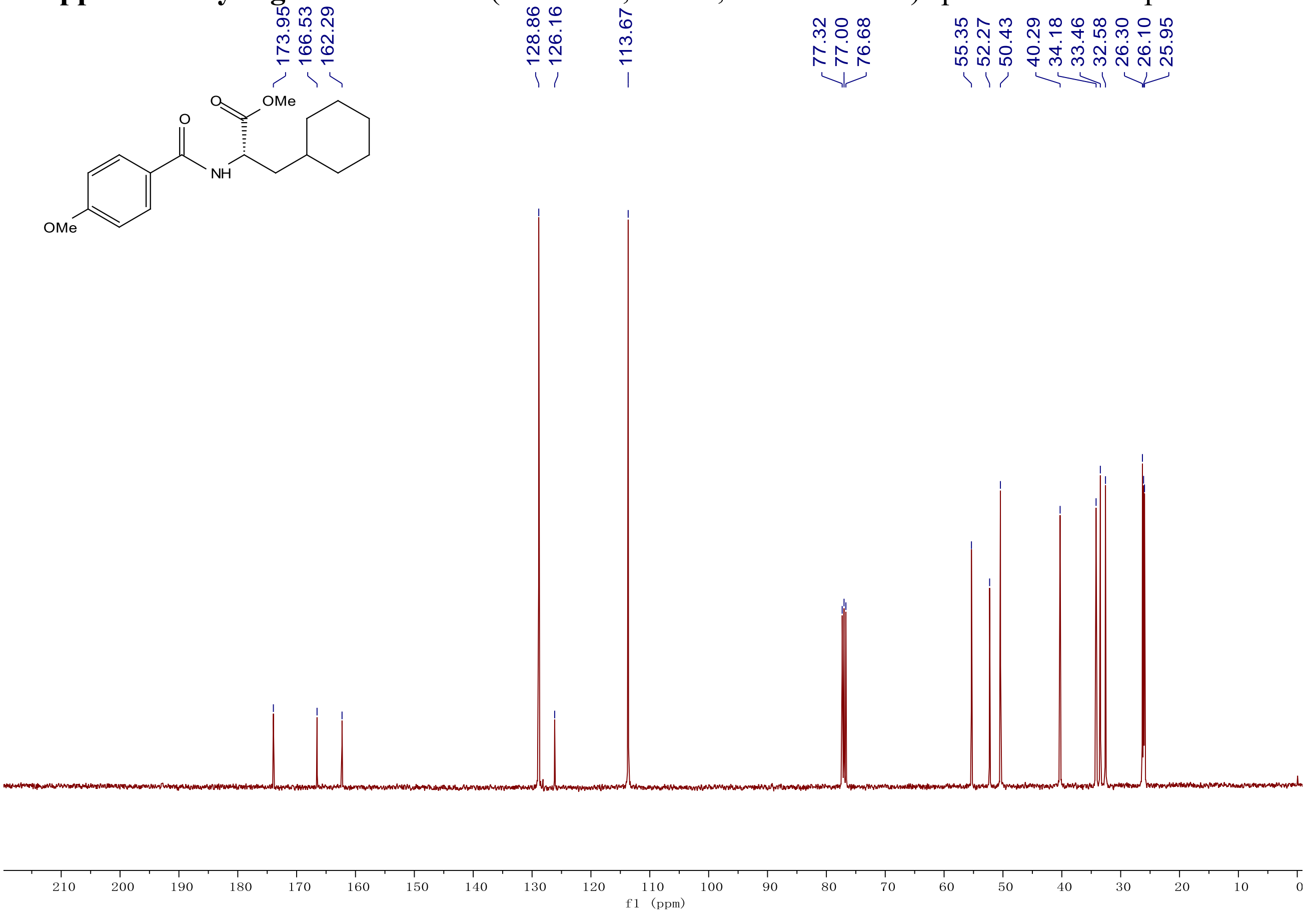

Supplementary Fig. 152. ^{13}C NMR (101 MHz, 298 K, Chloroform-*d*) spectrum of compound **1-c**.

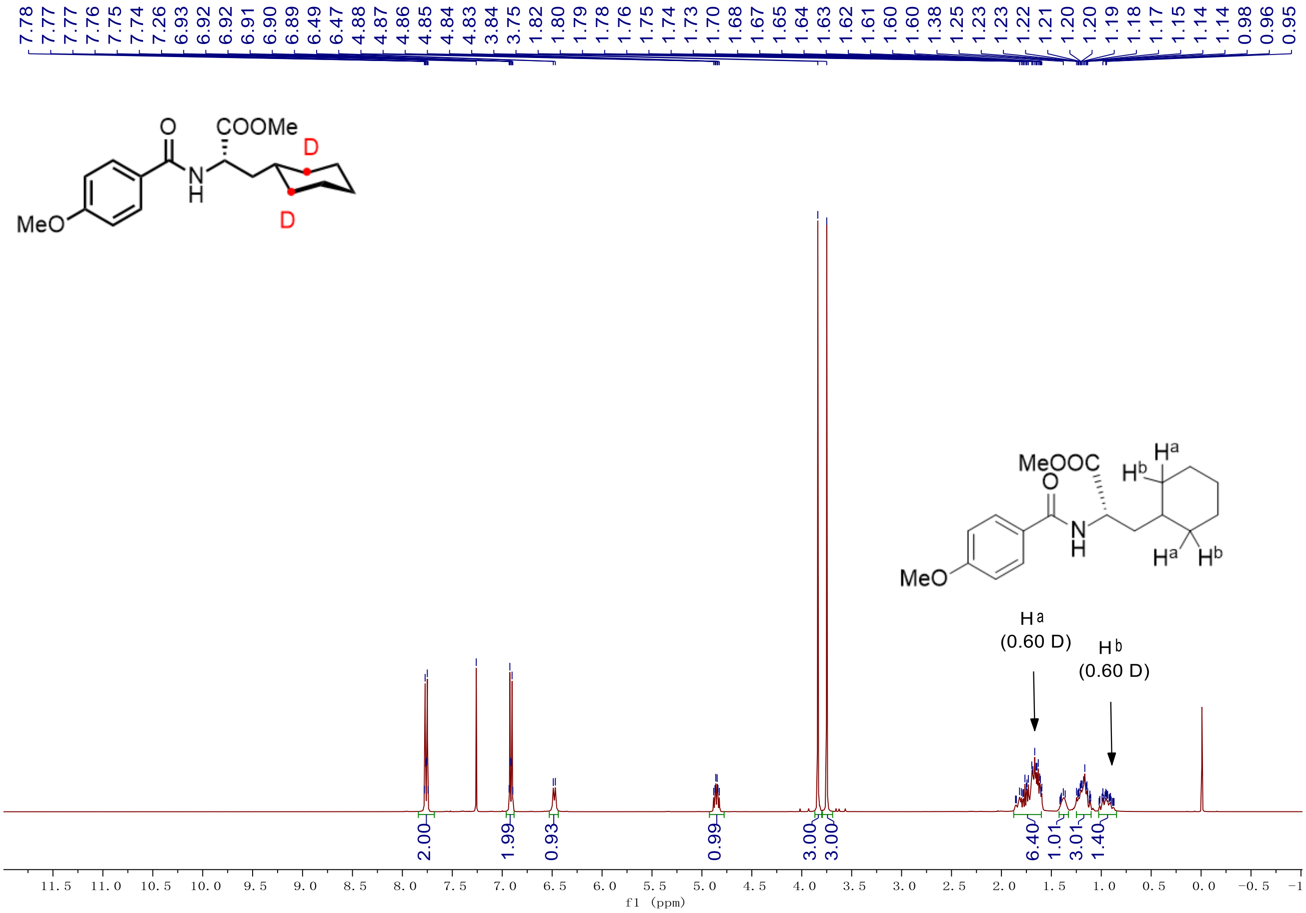

Supplementary Fig. 153. ^{1}H NMR (400 MHz, 298 K, Chloroform-*d*) spectrum of compound **4c**.

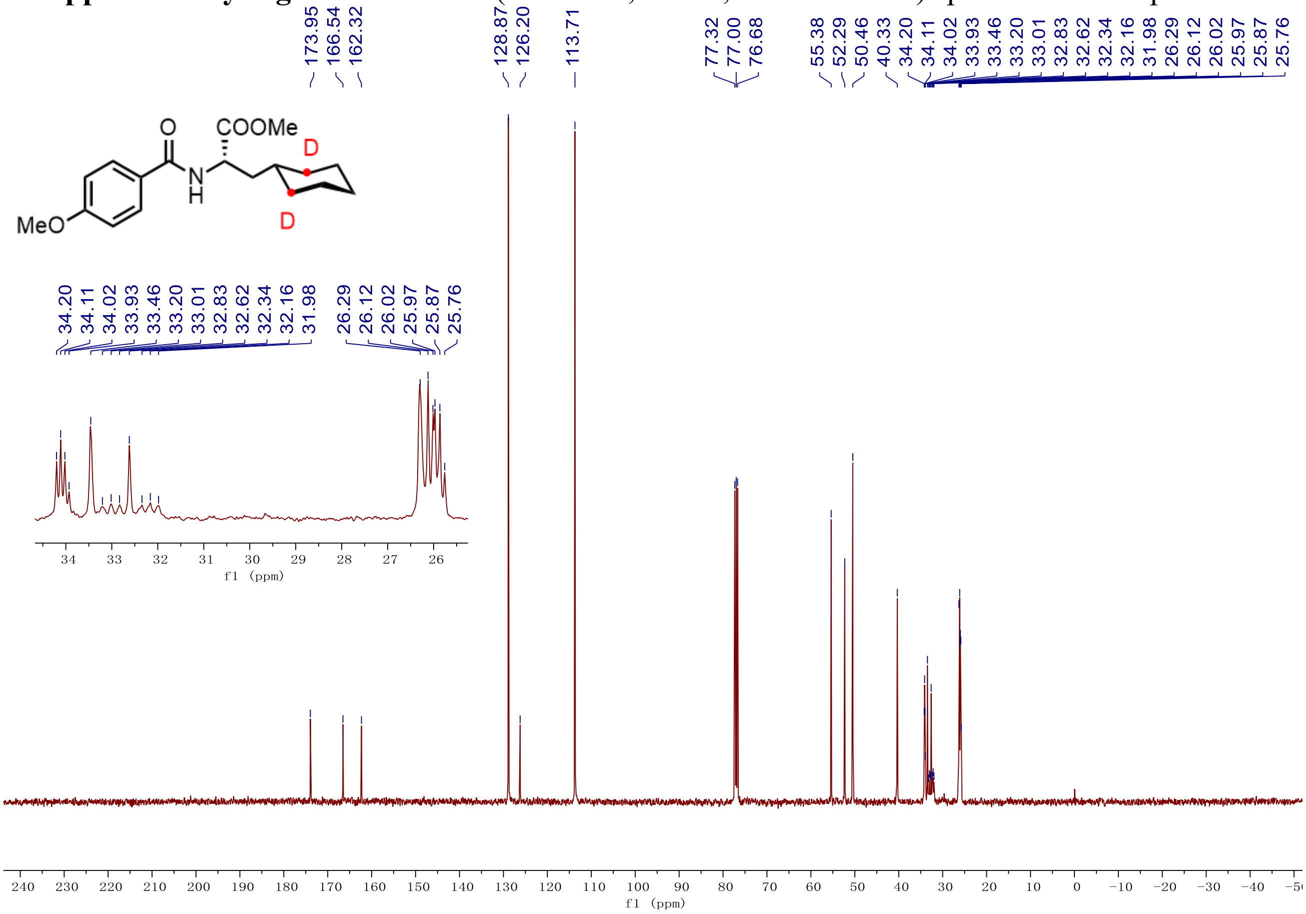

Supplementary Fig. 154. ^{13}C NMR (101 MHz, 298 K, Chloroform-*d*) spectrum of compound **4c**.

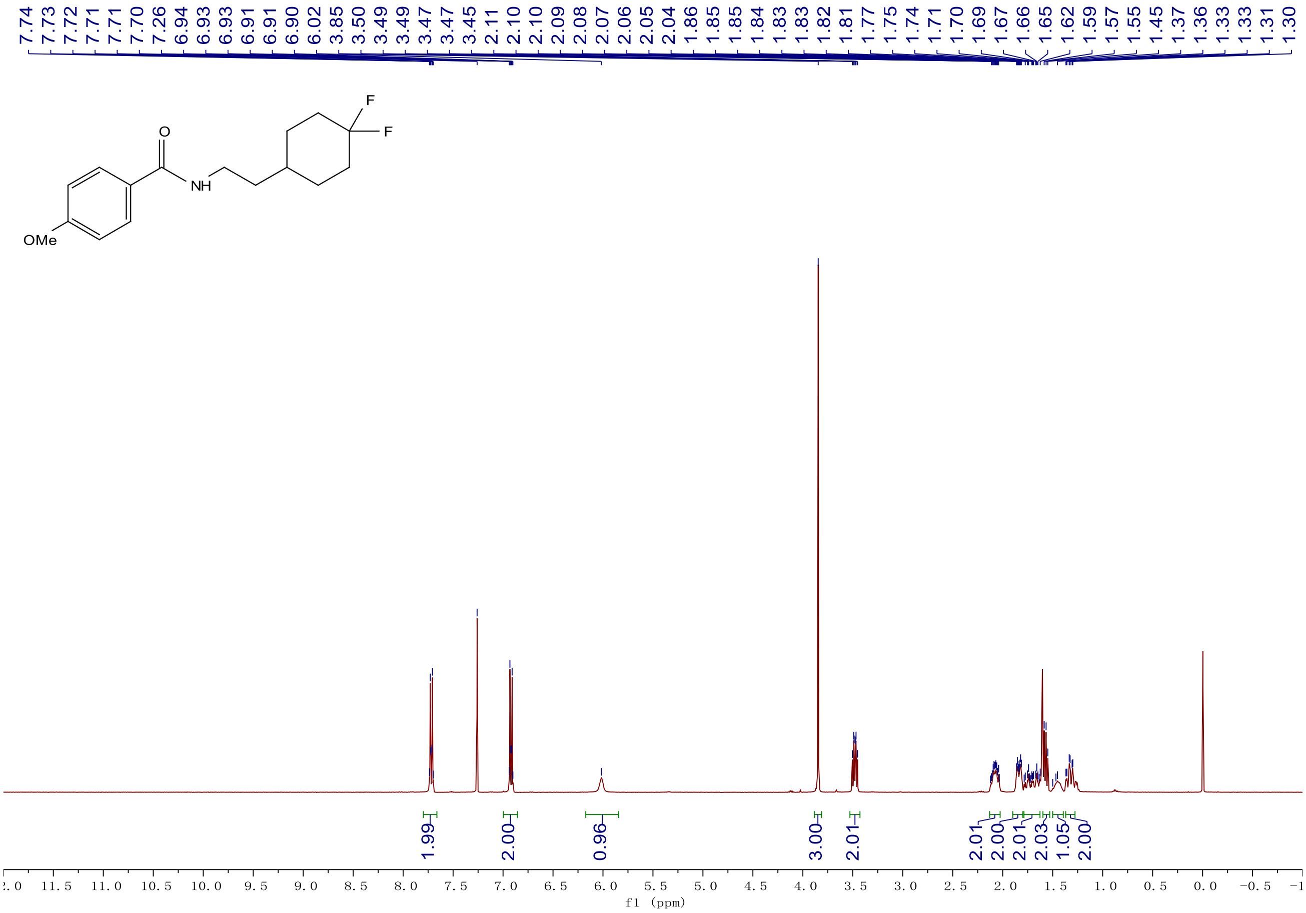

Supplementary Fig. 155. ^{1}H NMR (400 MHz, 298 K, Chloroform-*d*) spectrum of compound **1-d**.

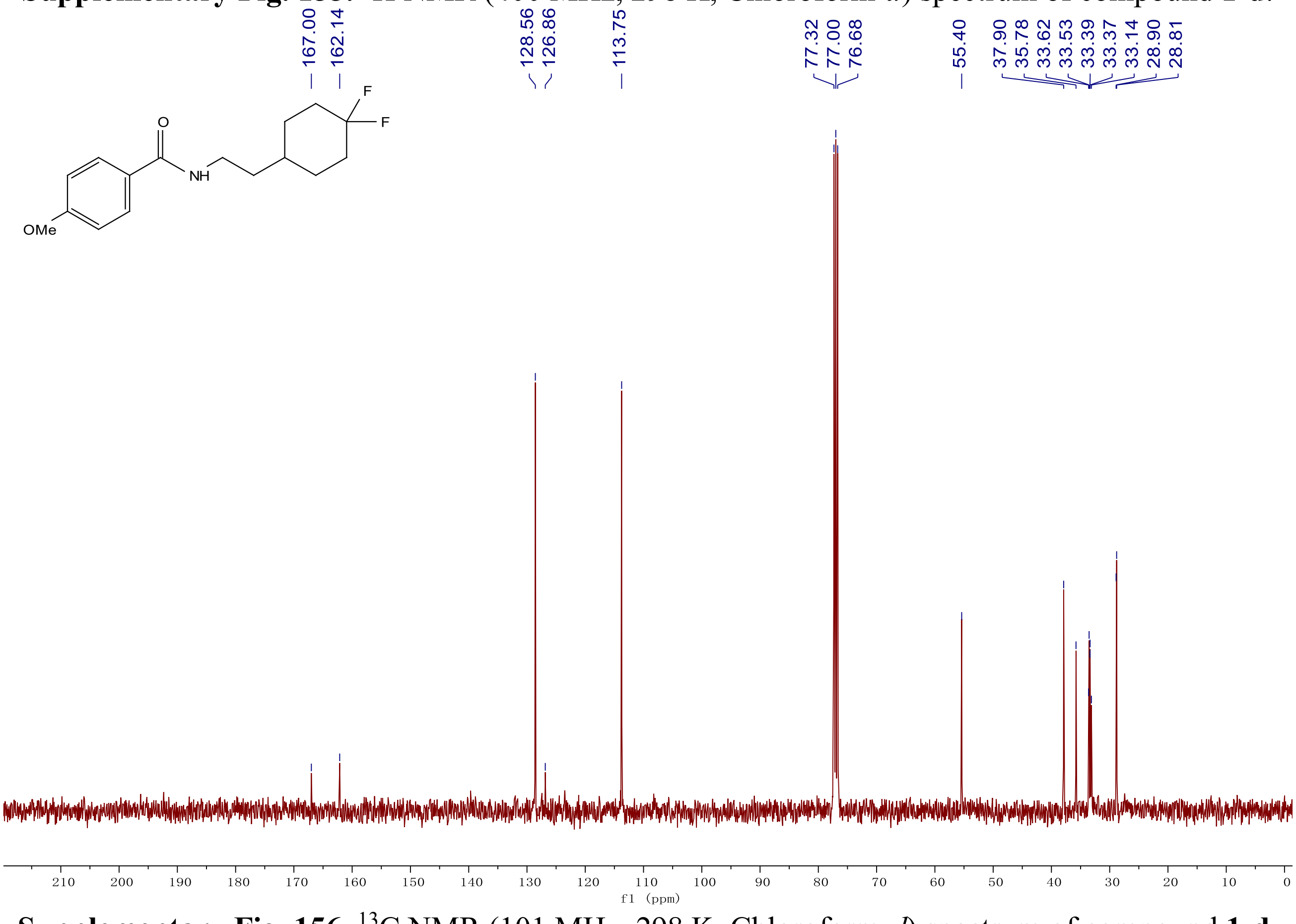

Supplementary Fig. 156. ^{13}C NMR (101 MHz, 298 K, Chloroform-*d*) spectrum of compound **1-d**.

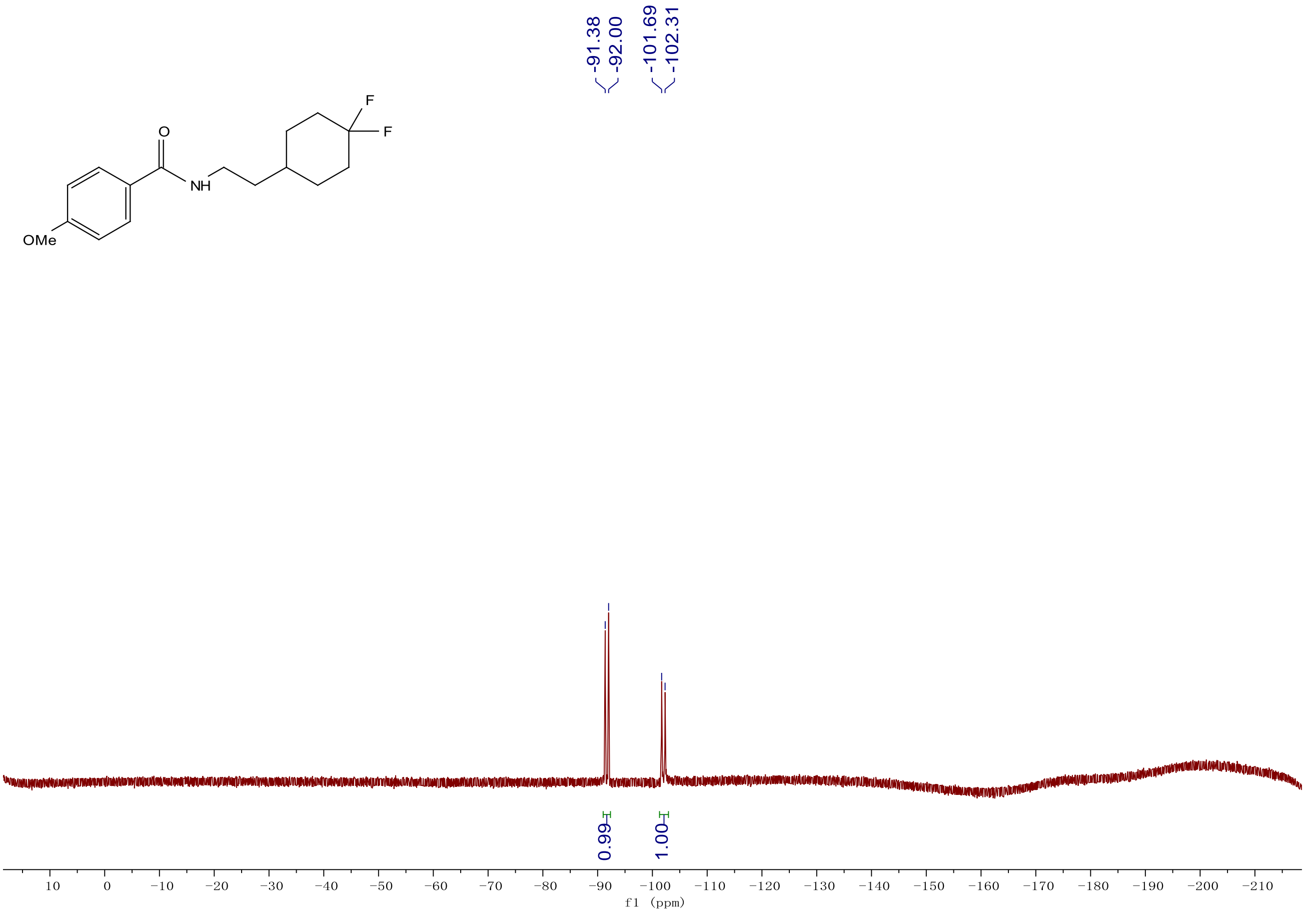

Supplementary Fig. 157. ^{19}F NMR (376 MHz, 298 K, Chloroform-*d*) spectrum of compound **1-d**.

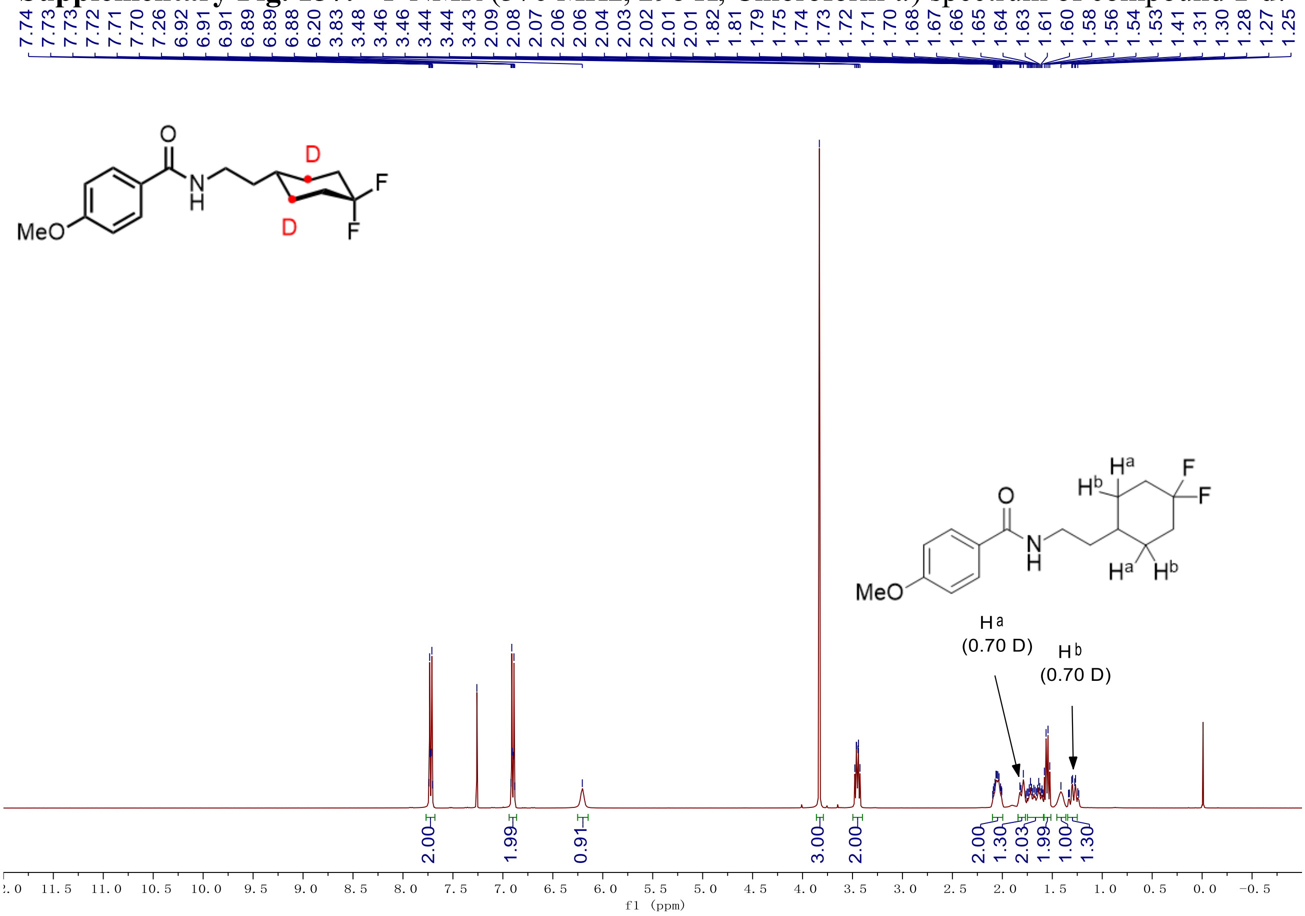

Supplementary Fig. 158. ^{1}H NMR (400 MHz, 298 K, Chloroform-*d*) spectrum of compound **4d**.

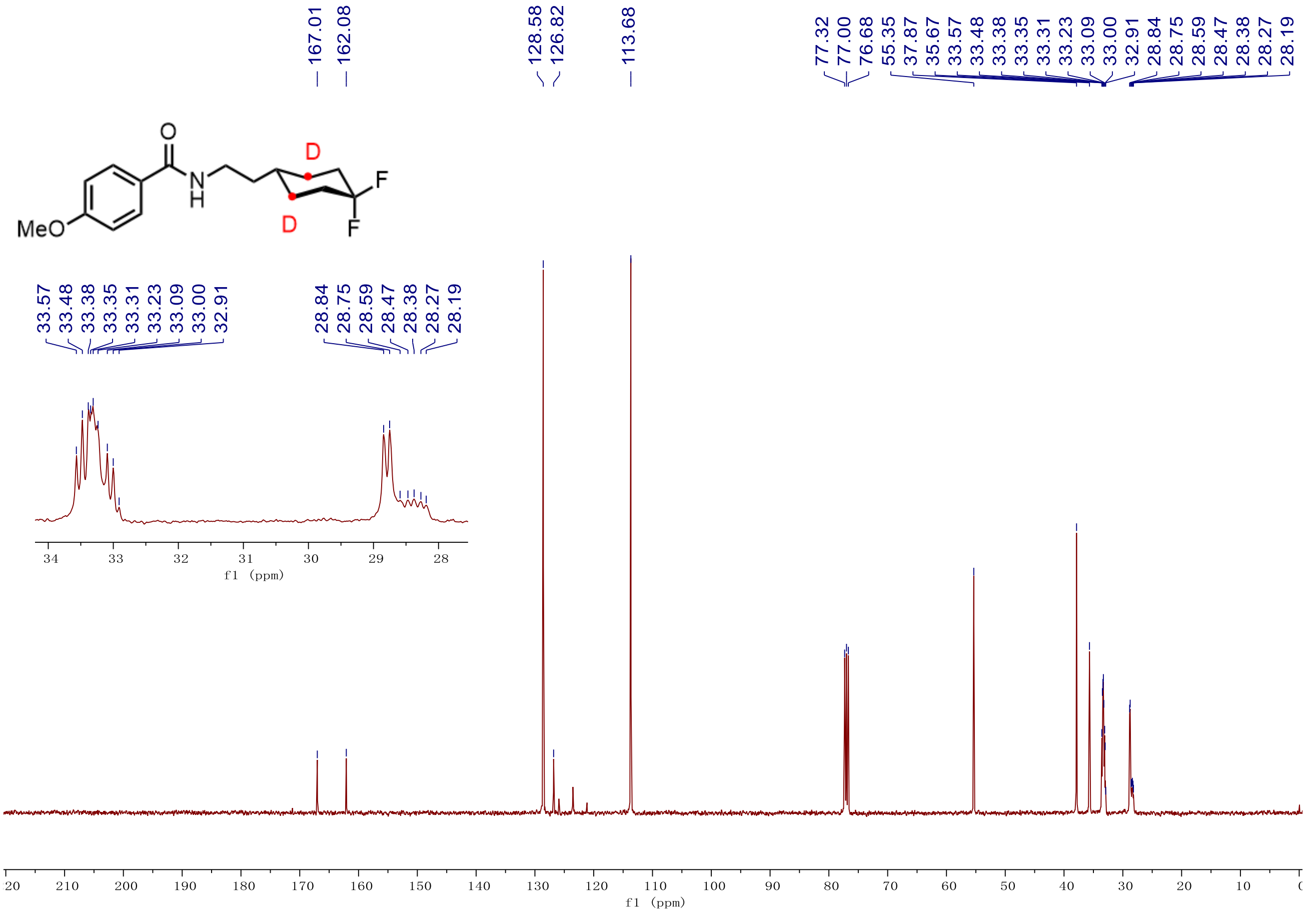

Supplementary Fig. 159. ^{13}C NMR (101 MHz, 298 K, Chloroform-*d*) spectrum of compound **4d**.

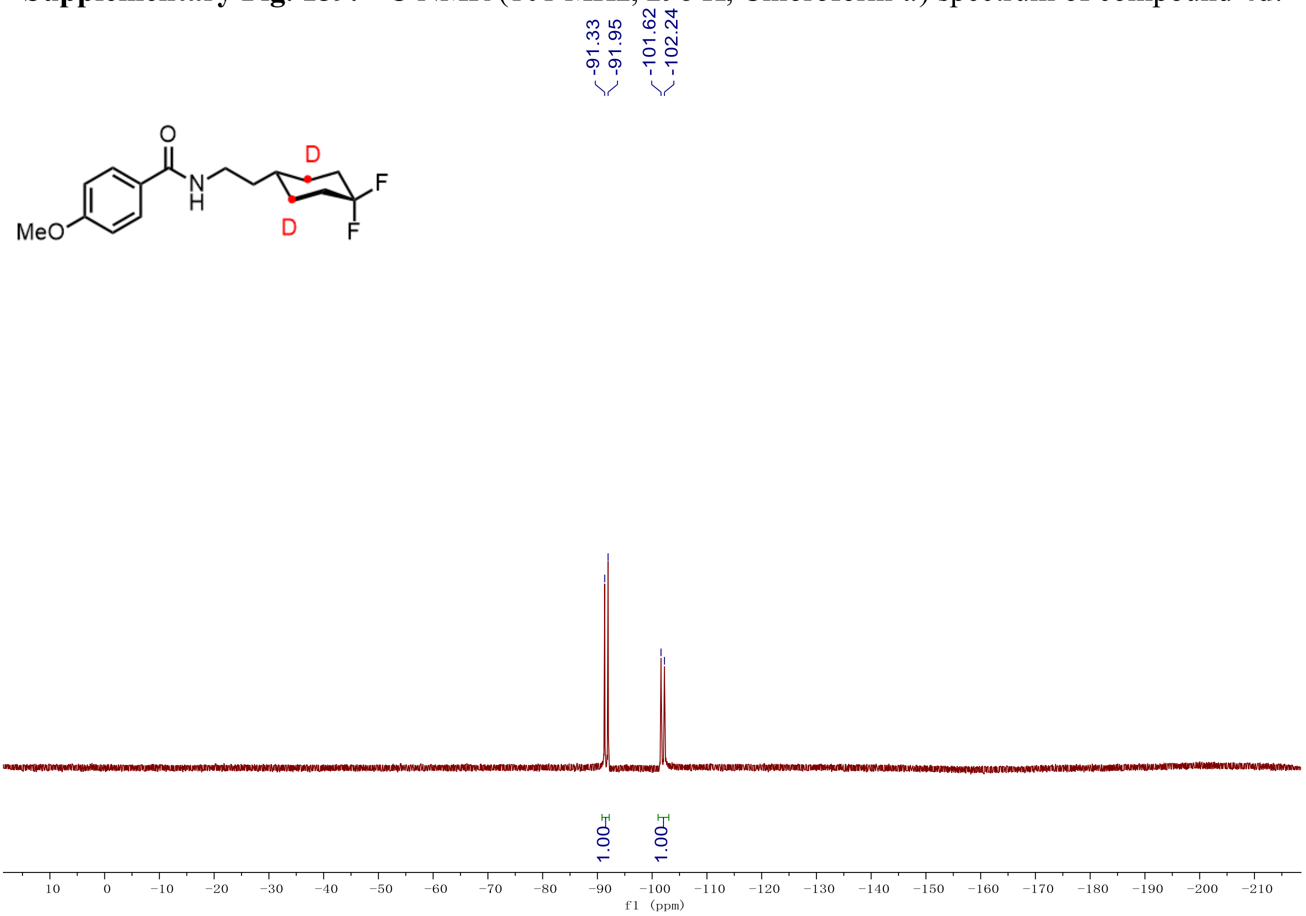

Supplementary Fig. 160. ^{19}F NMR (376 MHz, 298 K, Chloroform-*d*) spectrum of compound **4d**.

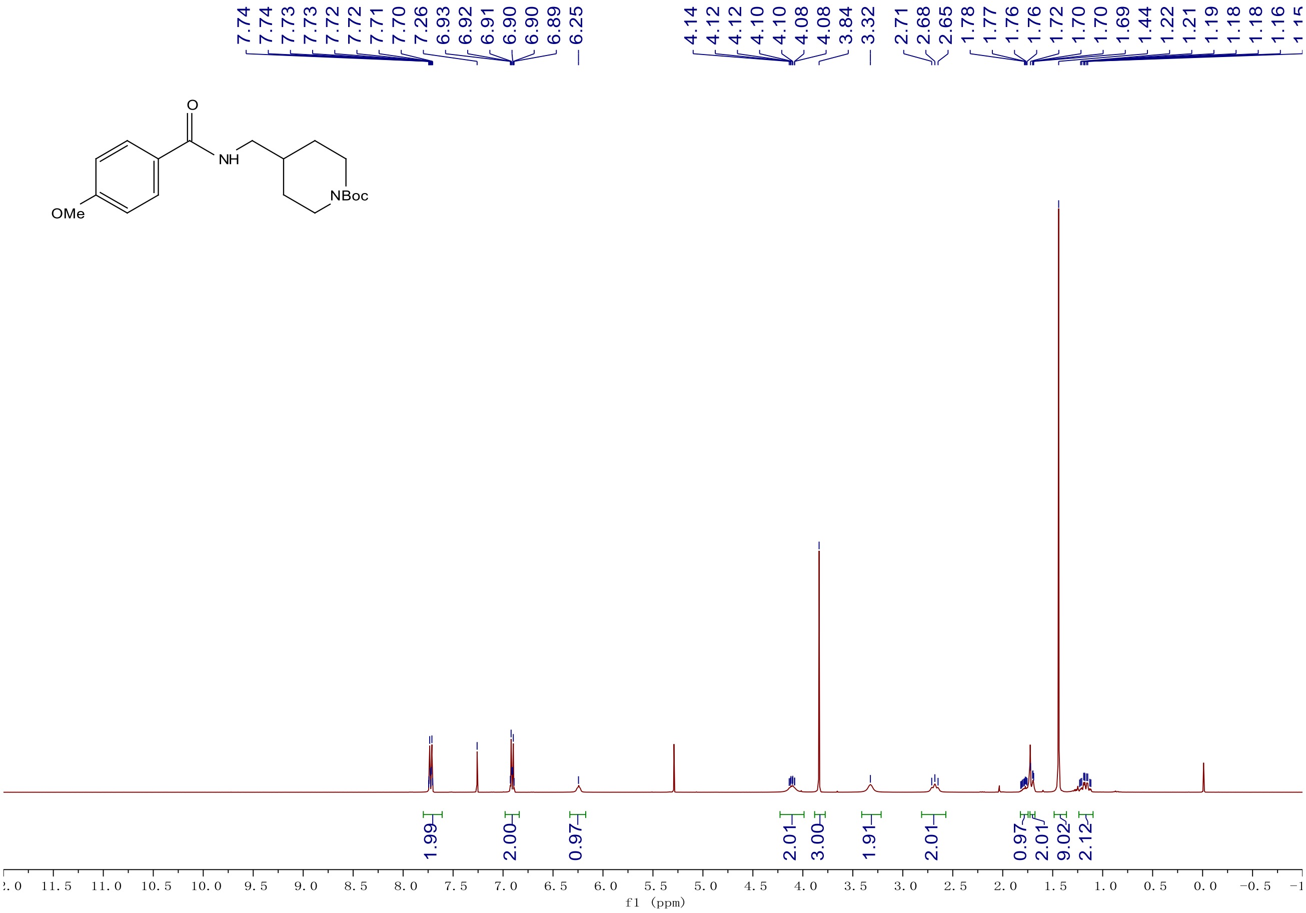

Supplementary Fig. 161. ^{1}H NMR (400 MHz, 298 K, Chloroform-*d*) spectrum of compound **1-e**.

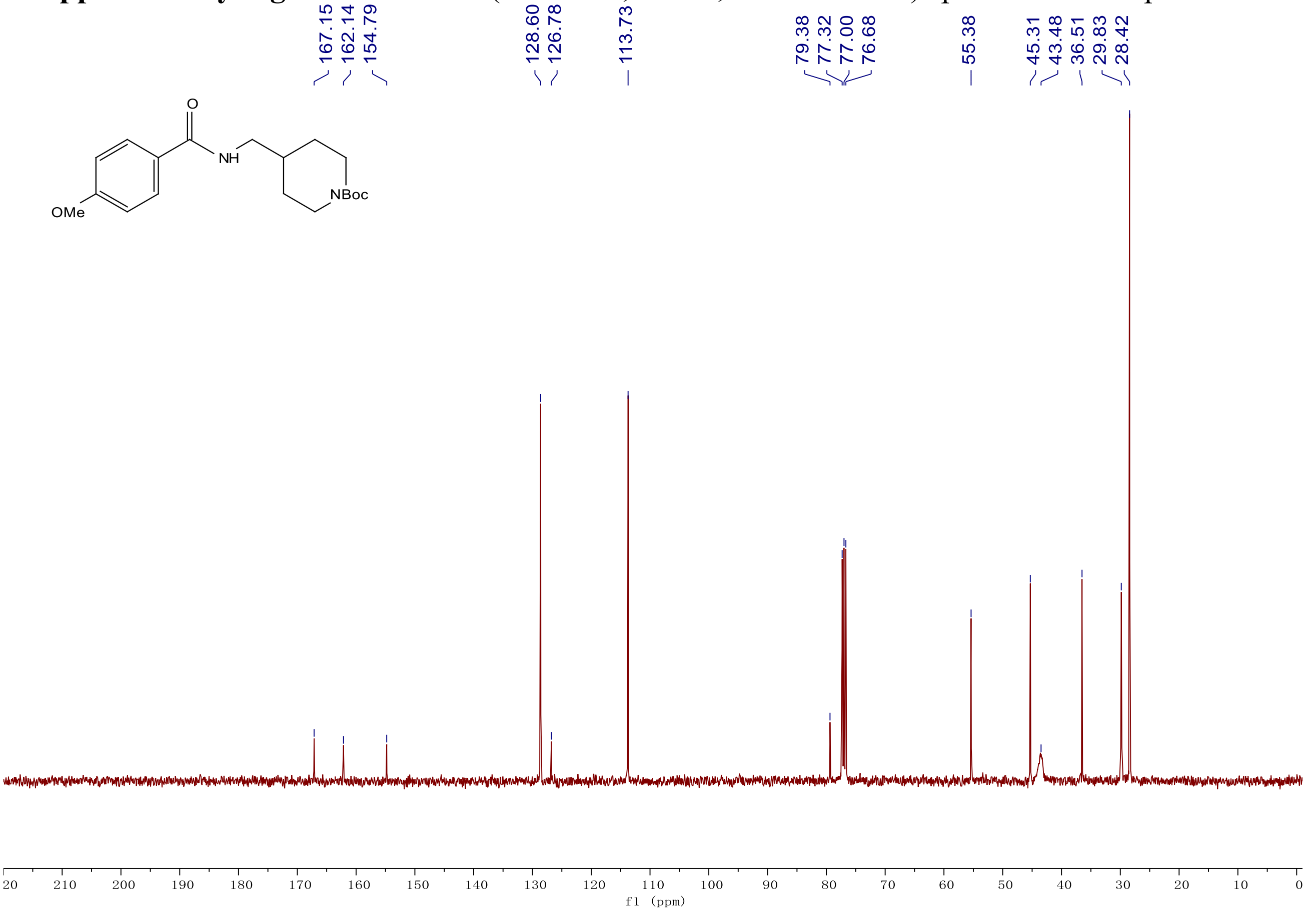

Supplementary Fig. 162. ^{13}C NMR (101 MHz, 298 K, Chloroform-*d*) spectrum of compound **1-e**.

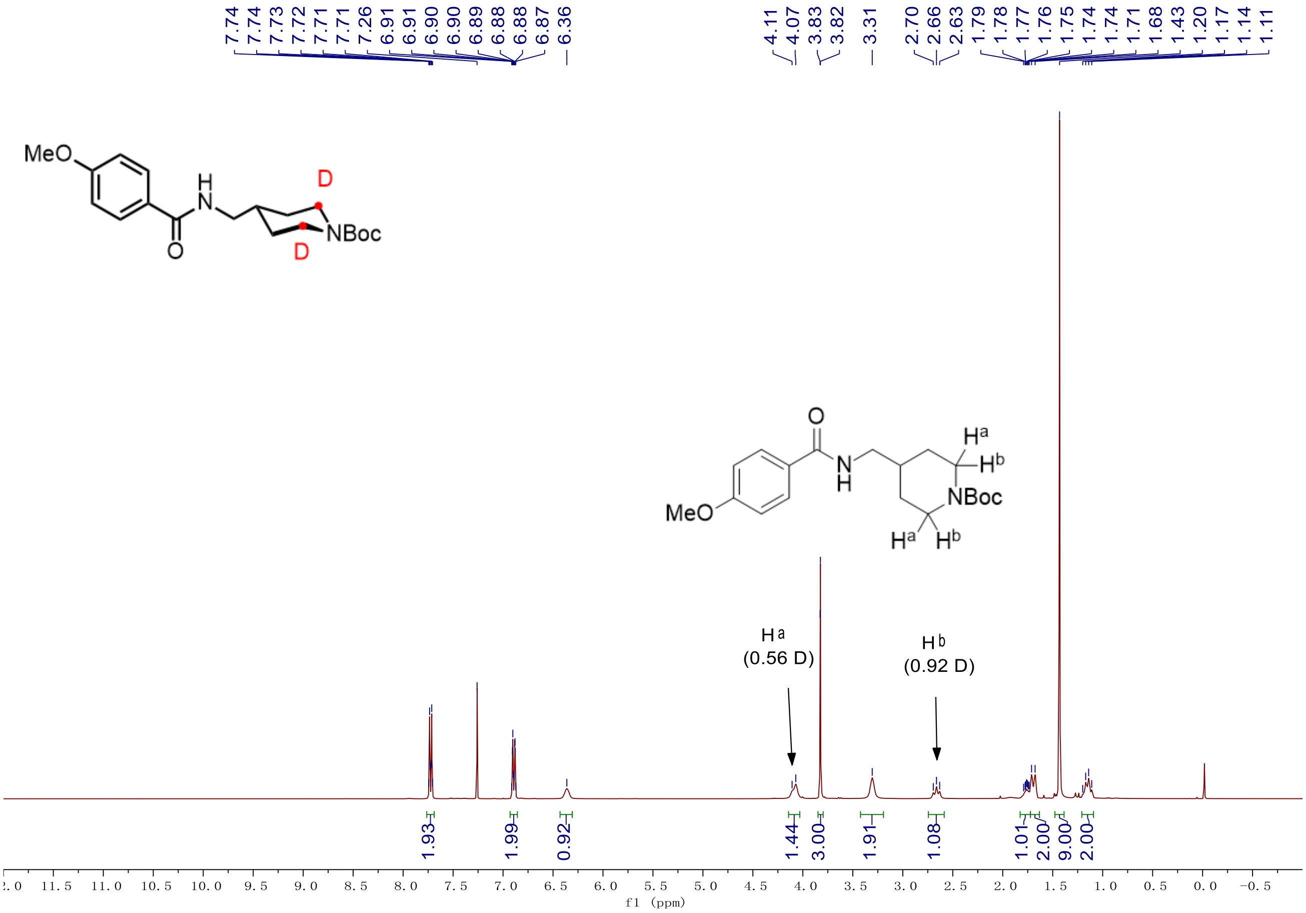

Supplementary Fig. 163. ^{1}H NMR (400 MHz, 298 K, Chloroform-*d*) spectrum of compound **4e**.

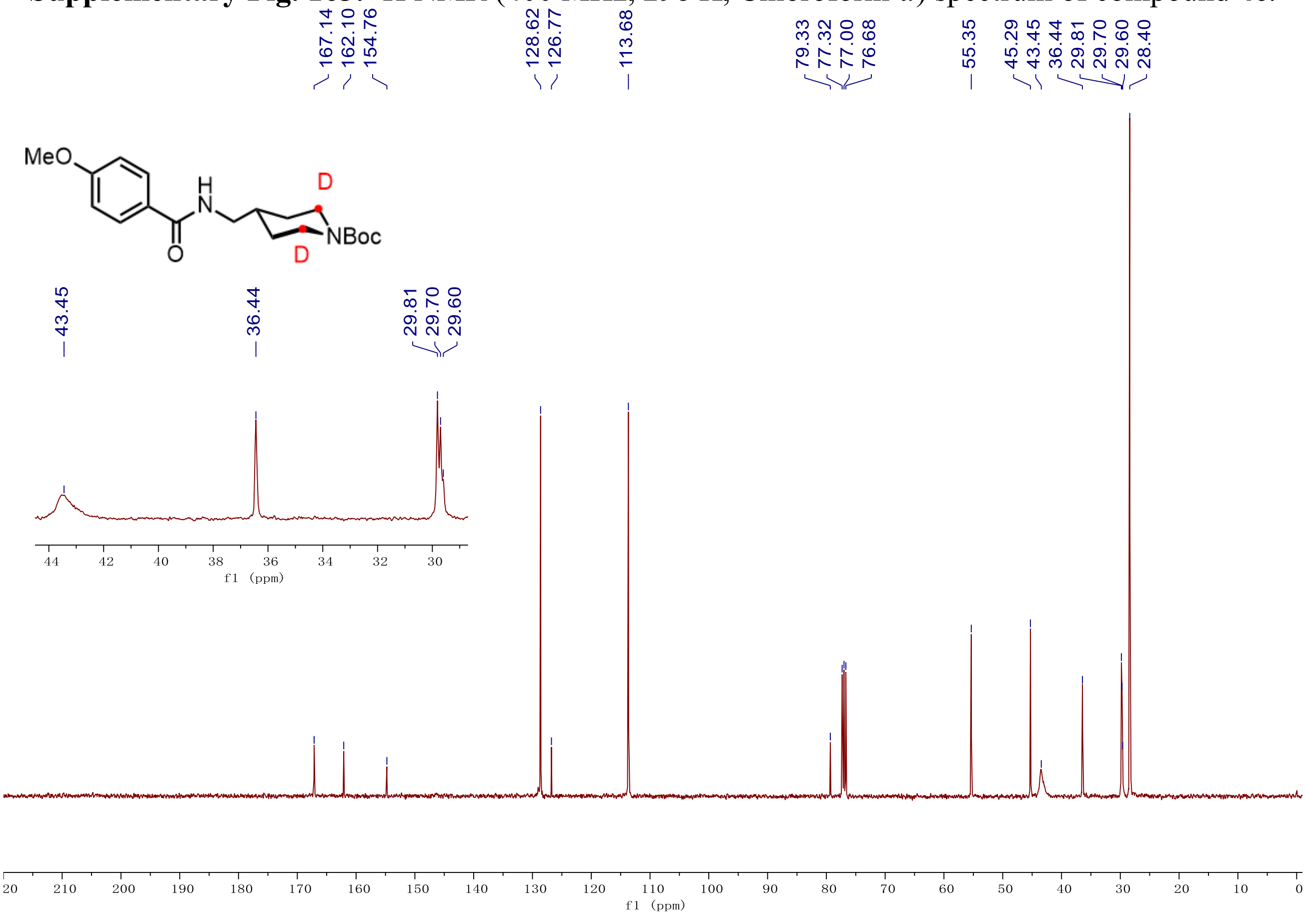

Supplementary Fig. 164. ^{13}C NMR (101 MHz, 298 K, Chloroform-*d*) spectrum of compound **4e**.

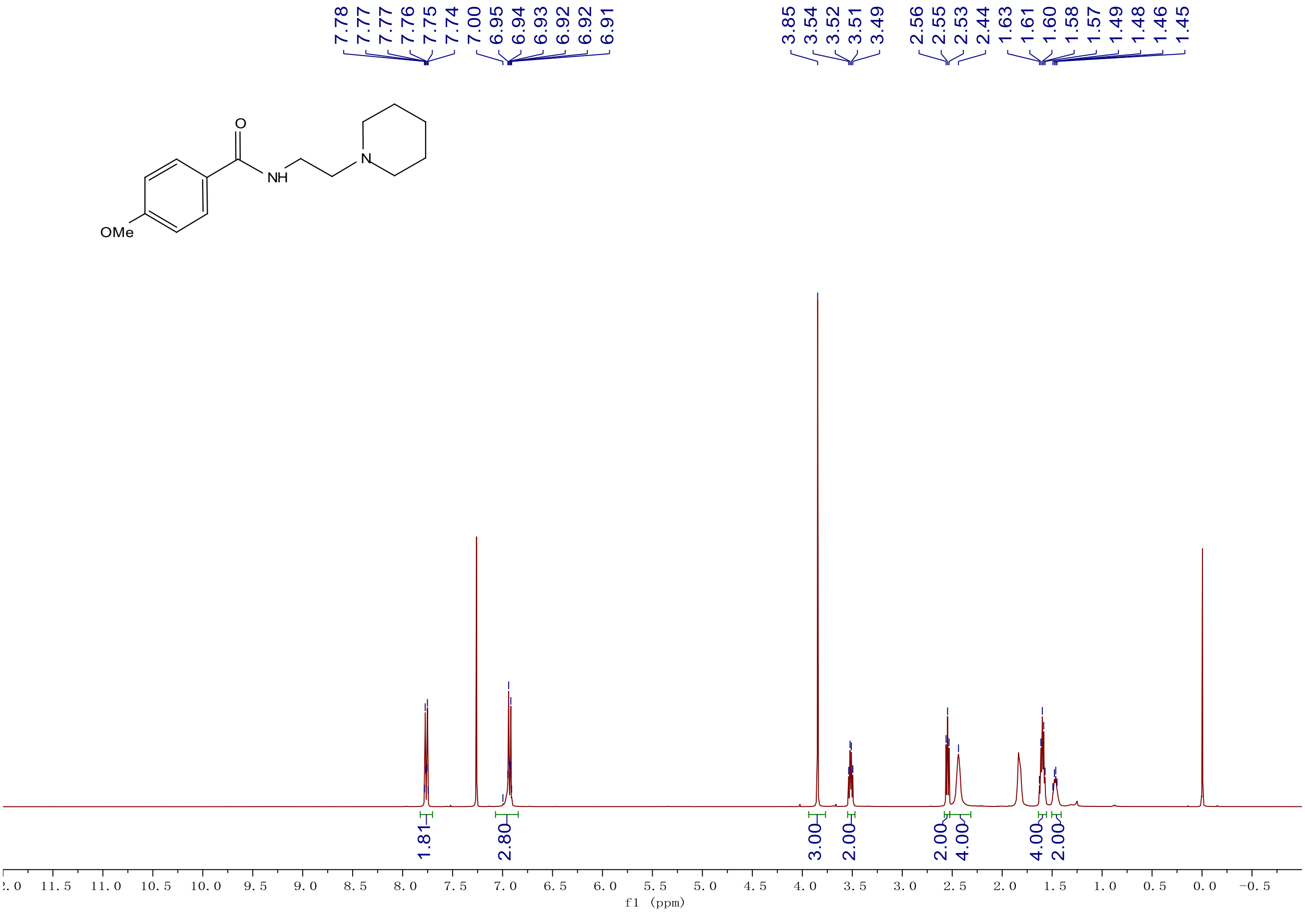

Supplementary Fig. 165. ^{1}H NMR (400 MHz, 298 K, Chloroform-*d*) spectrum of compound **1-f**.

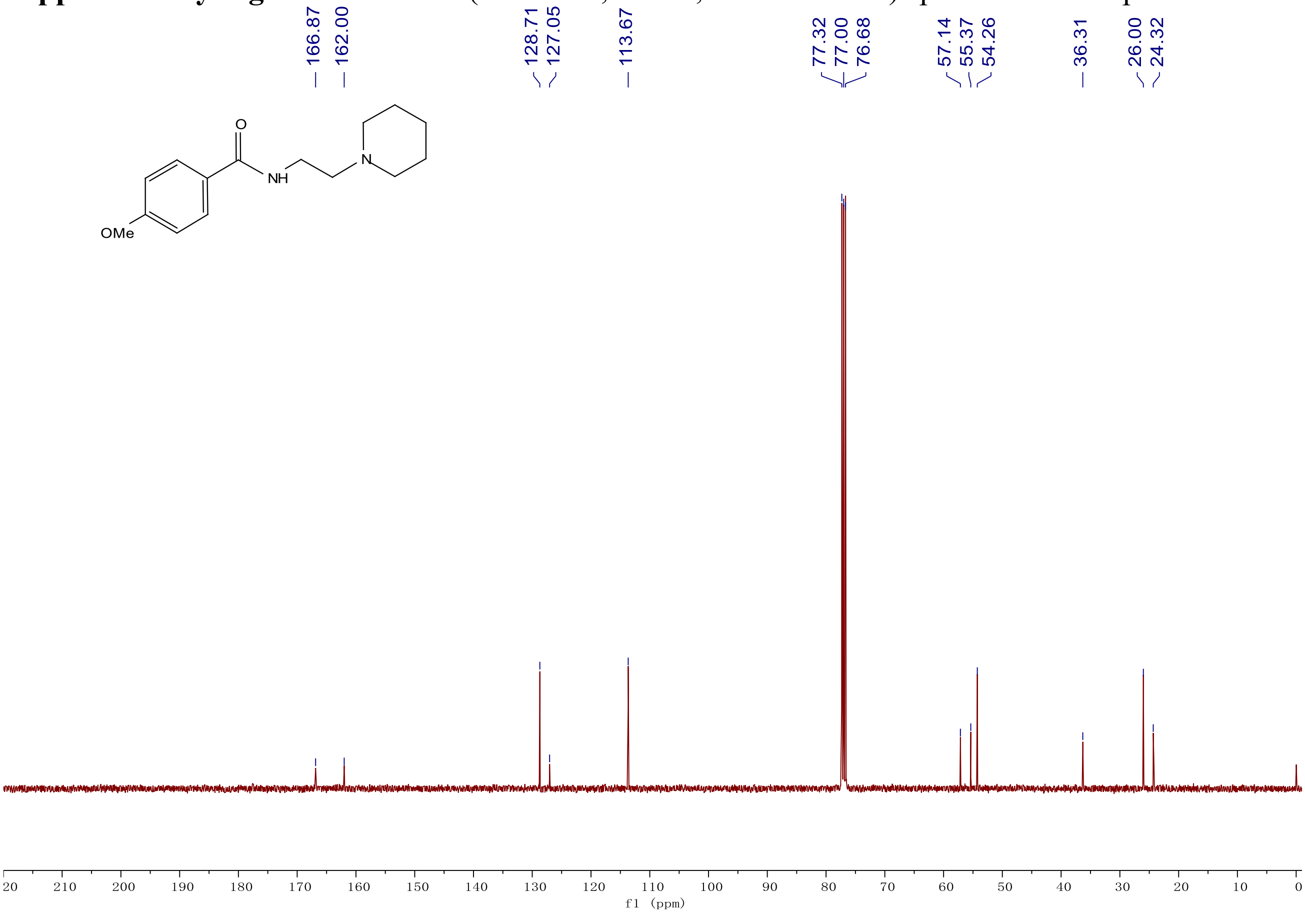

Supplementary Fig. 166. ^{13}C NMR (101 MHz, 298 K, Chloroform-*d*) spectrum of compound **1-f**.

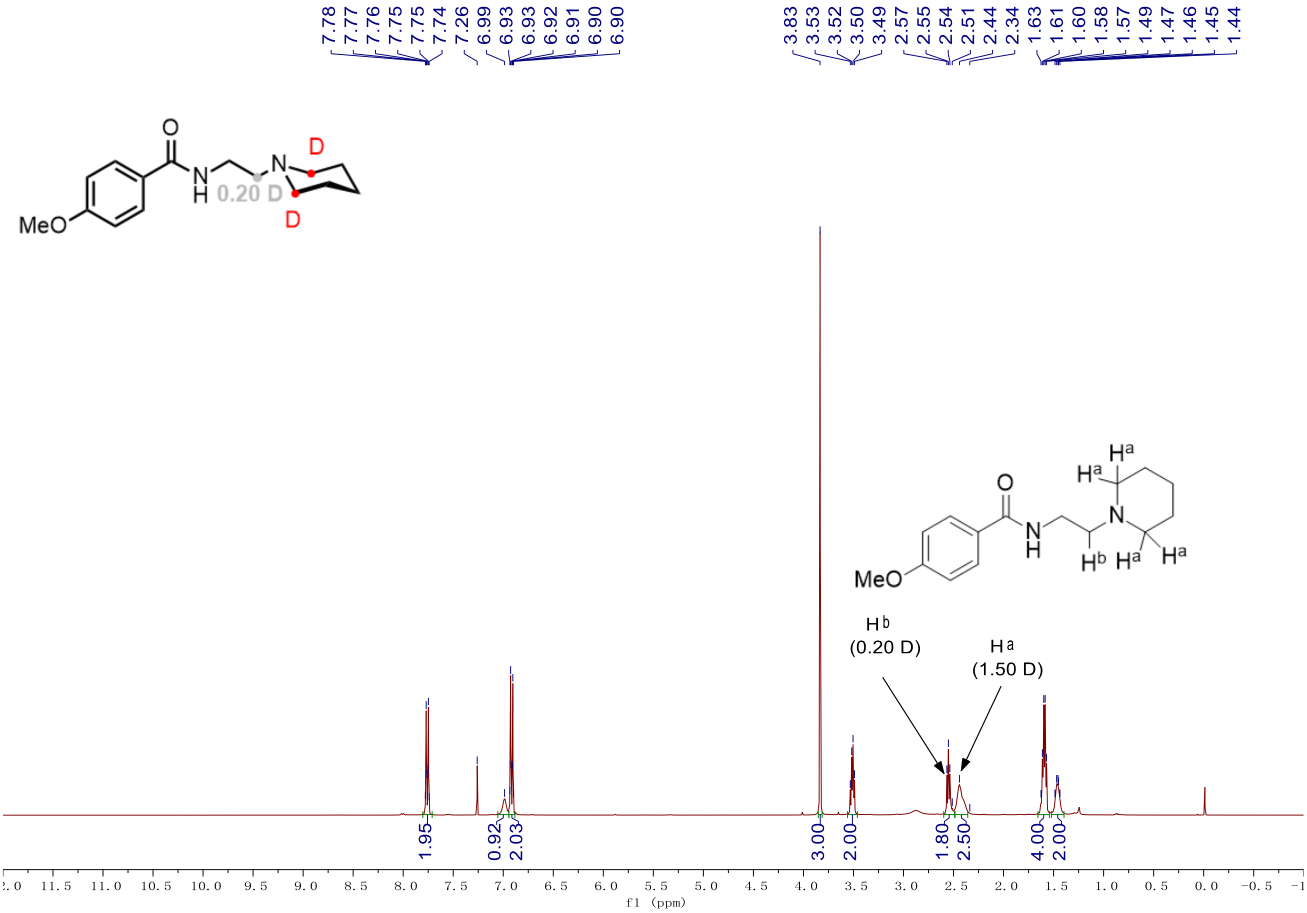

Supplementary Fig. 167. ^{1}H NMR (400 MHz, 298 K, Chloroform-*d*) spectrum of compound **4f**.

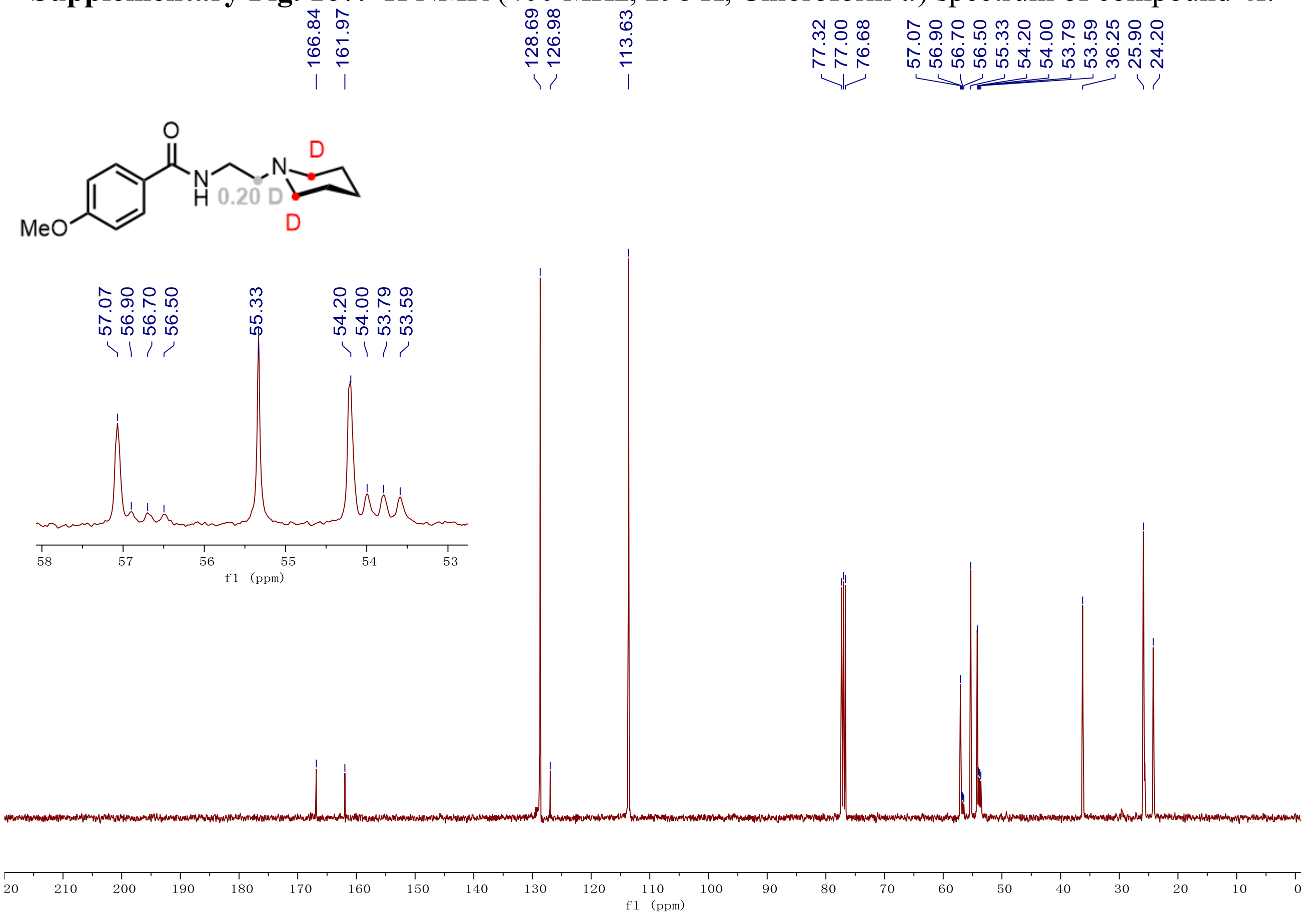

Supplementary Fig. 168. ^{13}C NMR (101 MHz, 298 K, Chloroform-*d*) spectrum of compound **4f**.

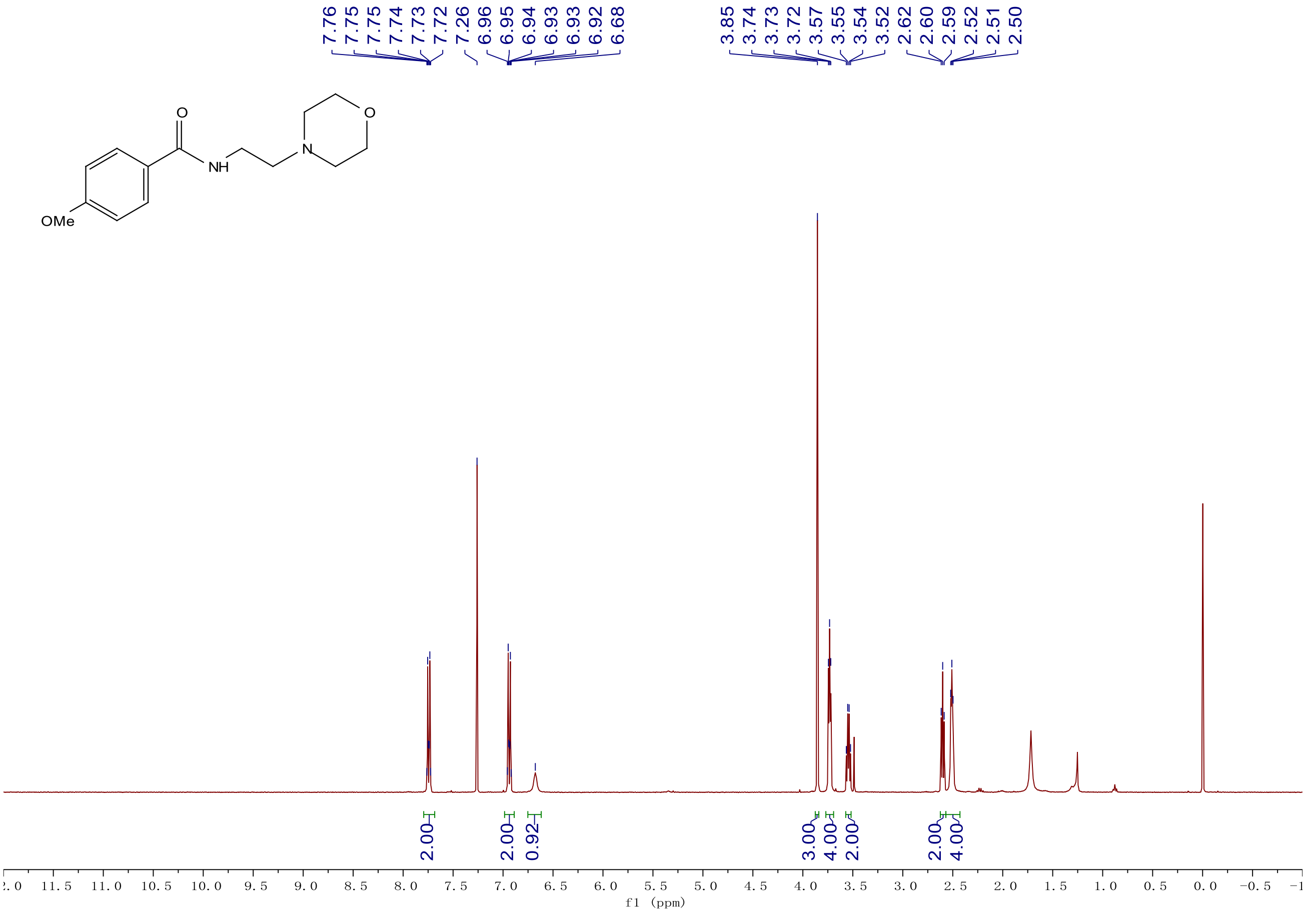

Supplementary Fig. 169. ^{1}H NMR (400 MHz, 298 K, Chloroform-*d*) spectrum of compound **1-g**.

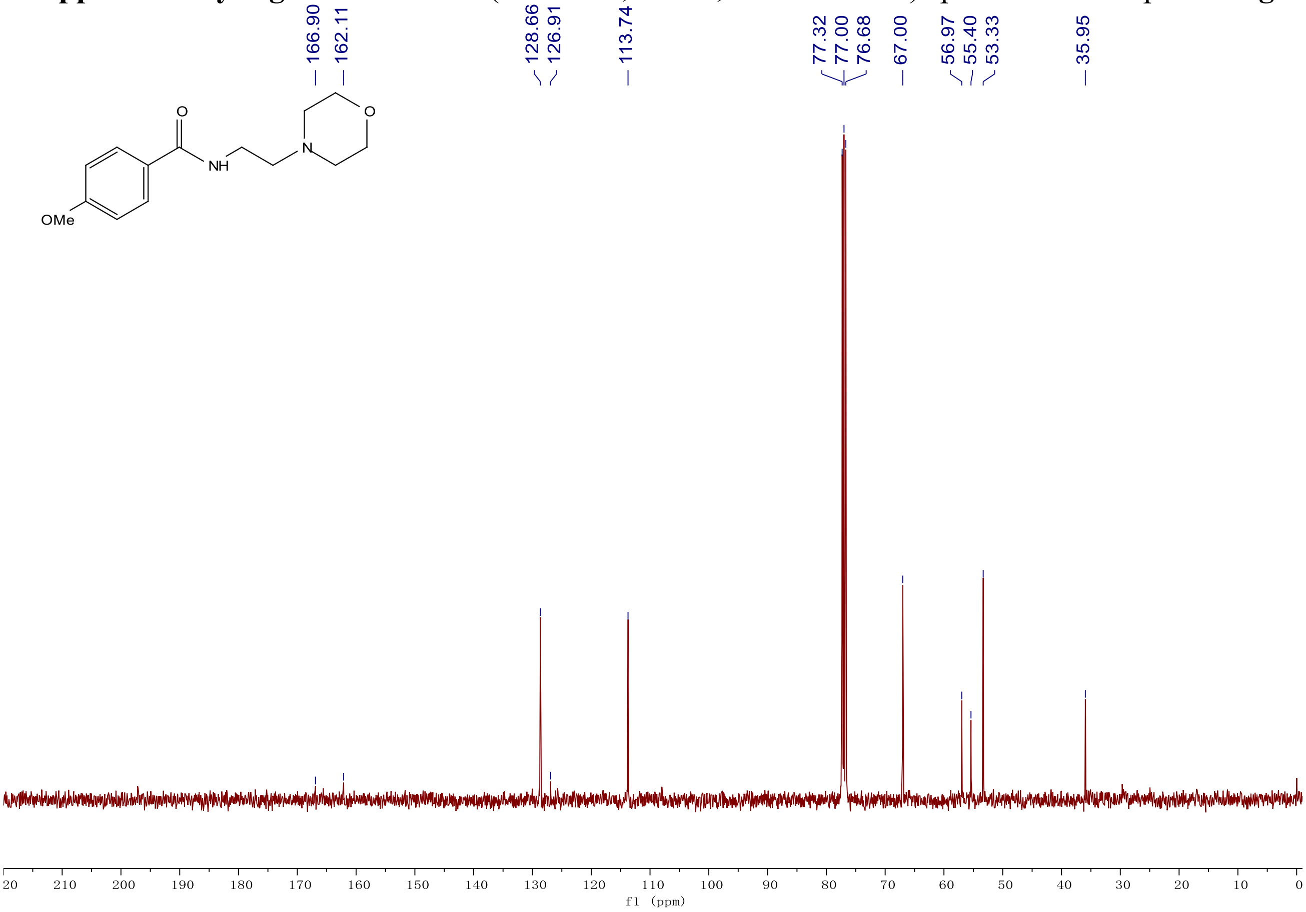

Supplementary Fig. 170. ^{13}C NMR (101 MHz, 298 K, Chloroform-*d*) spectrum of compound **1-g**.

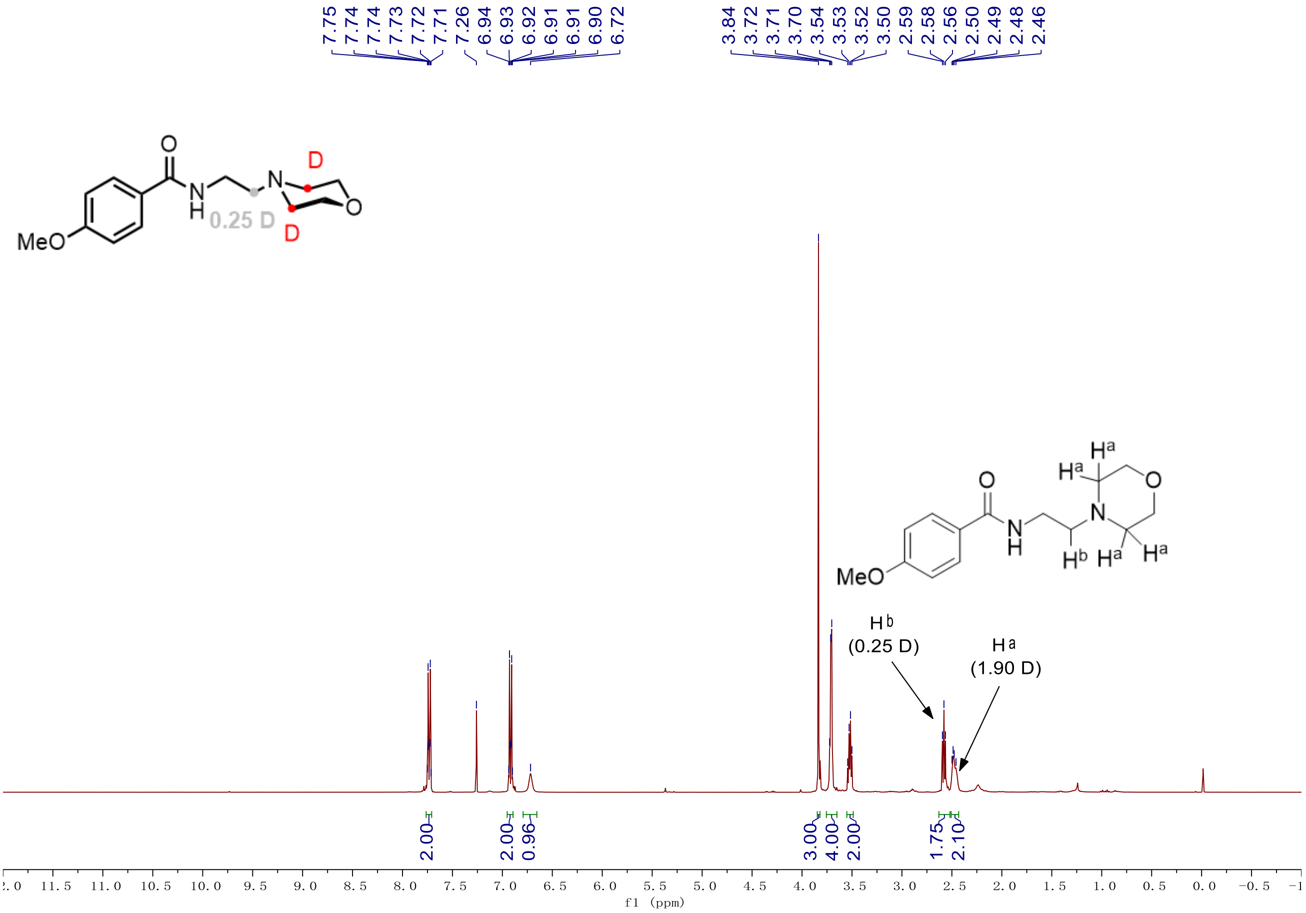

Supplementary Fig. 171. ^{1}H NMR (400 MHz, 298 K, Chloroform-*d*) spectrum of compound **4g**.

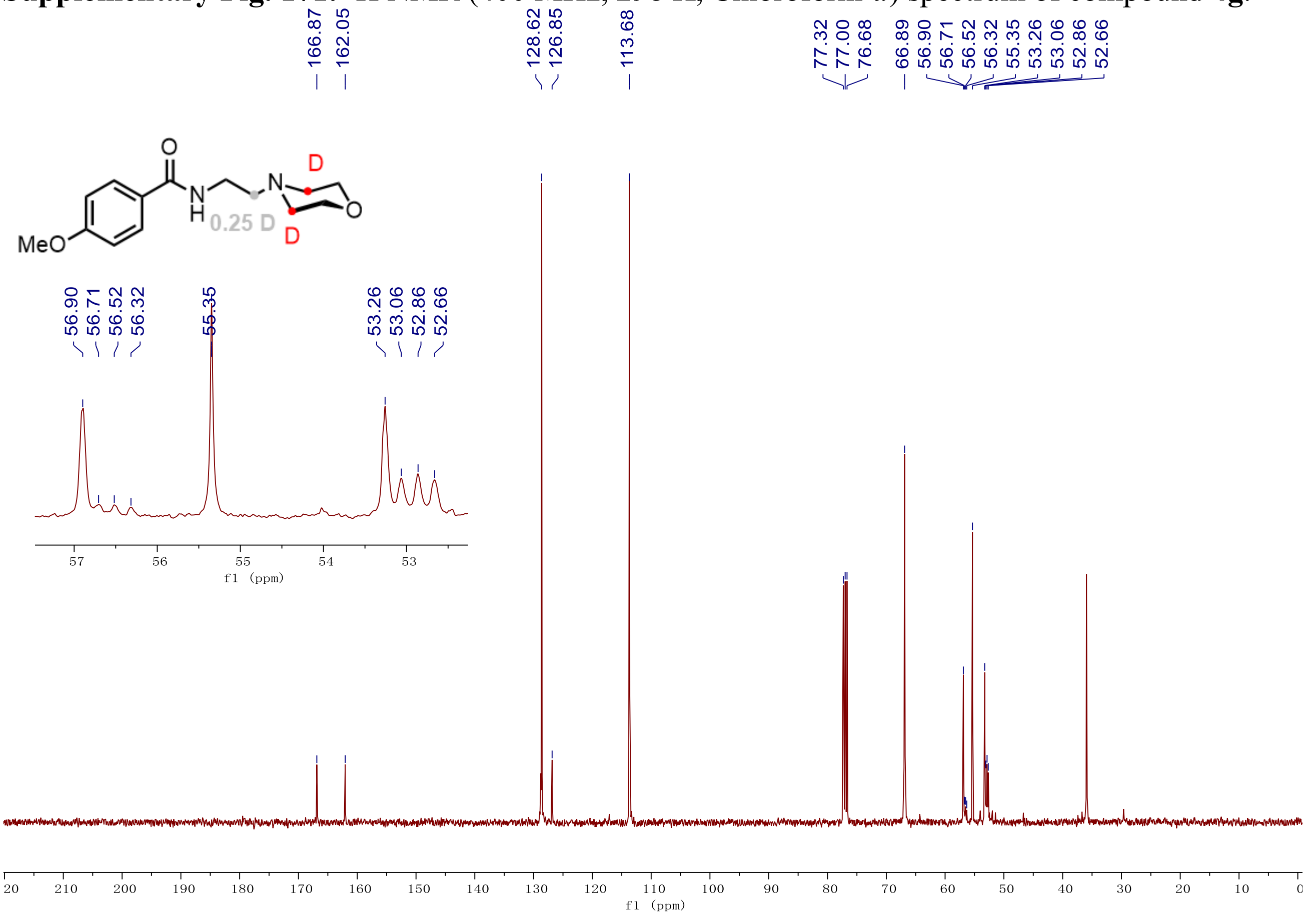

Supplementary Fig. 172. ^{13}C NMR (101 MHz, 298 K, Chloroform-*d*) spectrum of compound **4g**.

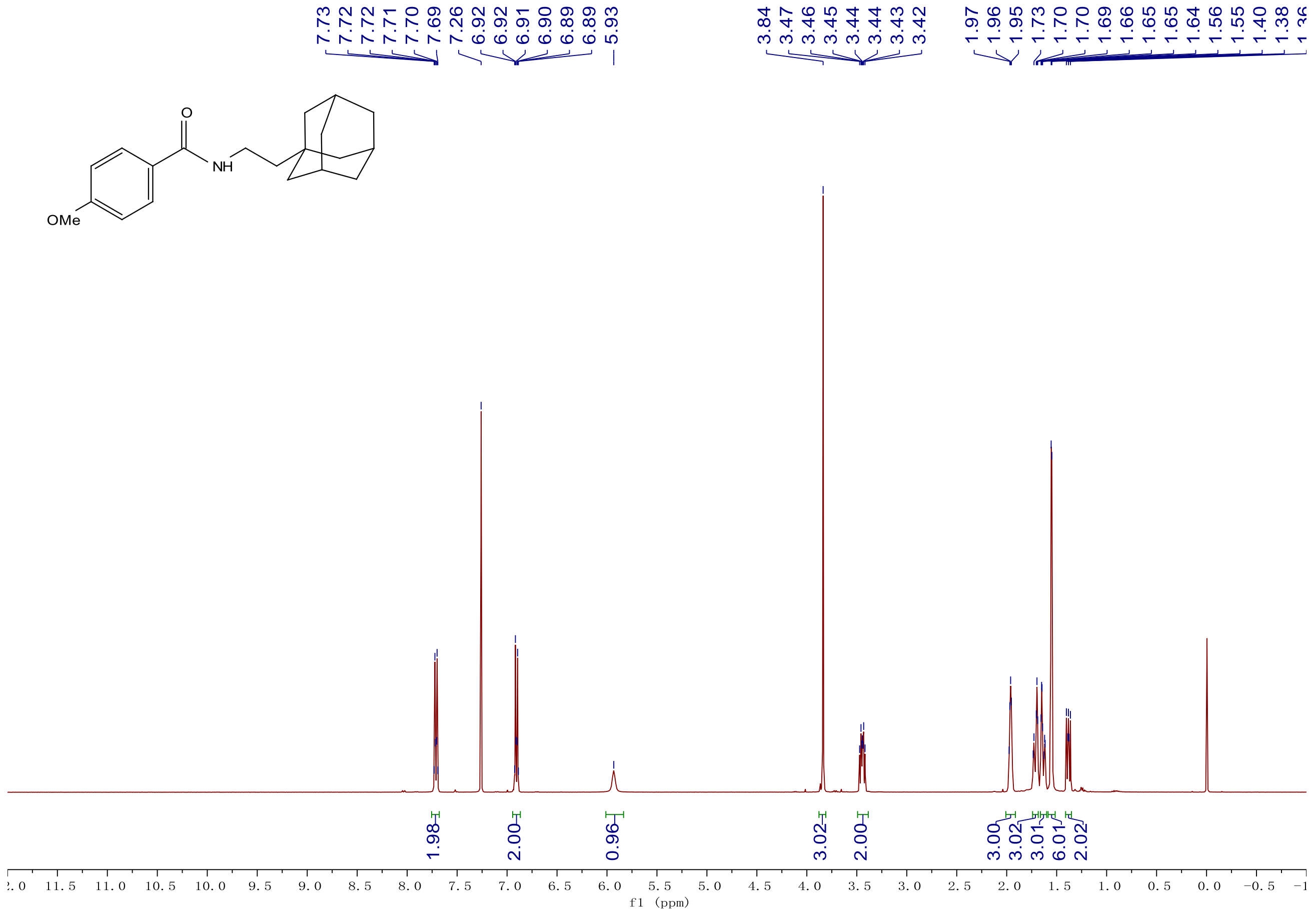

Supplementary Fig. 173. ^{1}H NMR (400 MHz, 298 K, Chloroform-*d*) spectrum of compound **1-h**.

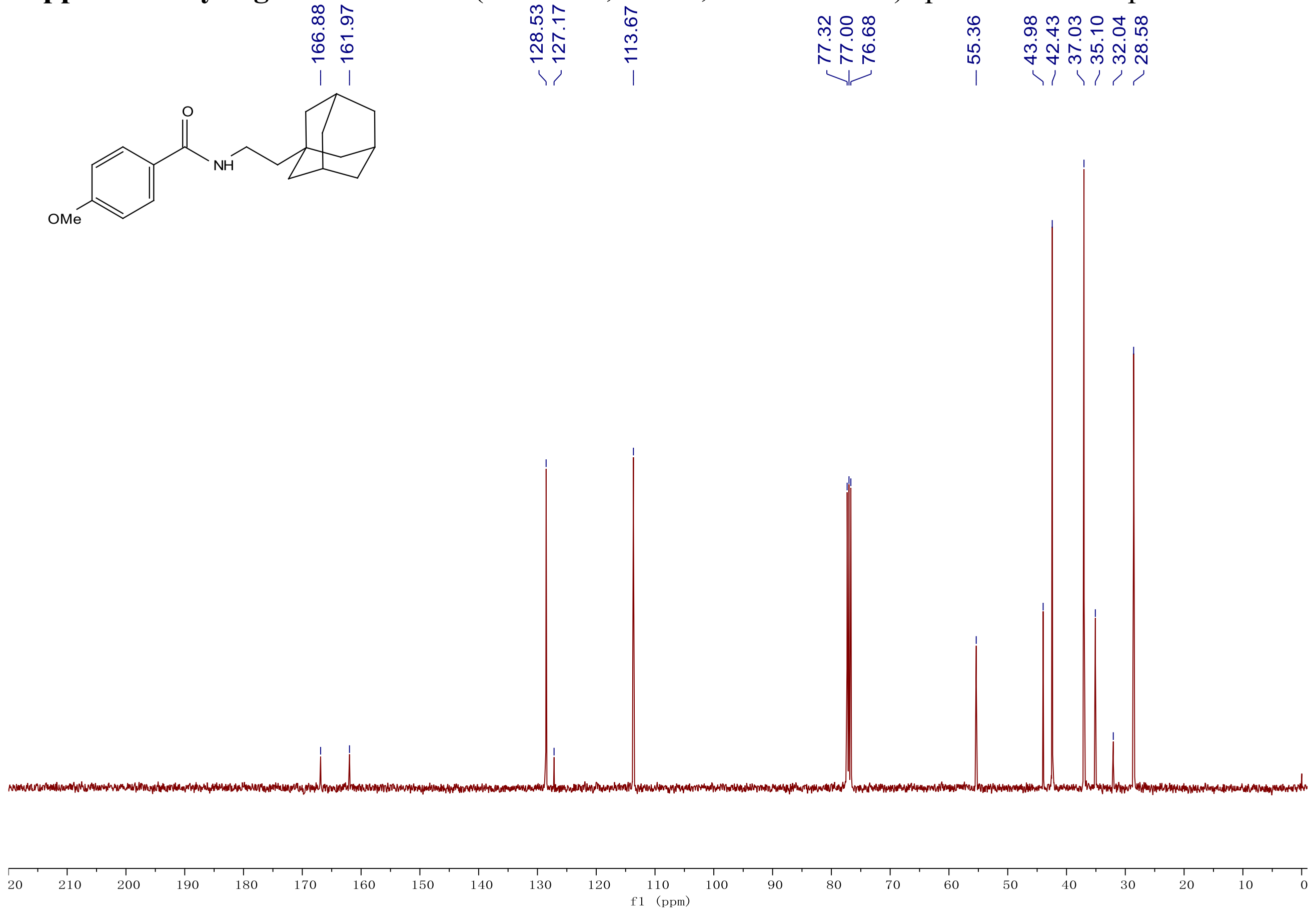

Supplementary Fig. 174. ^{13}C NMR (101 MHz, 298 K, Chloroform-*d*) spectrum of compound **1-h**.

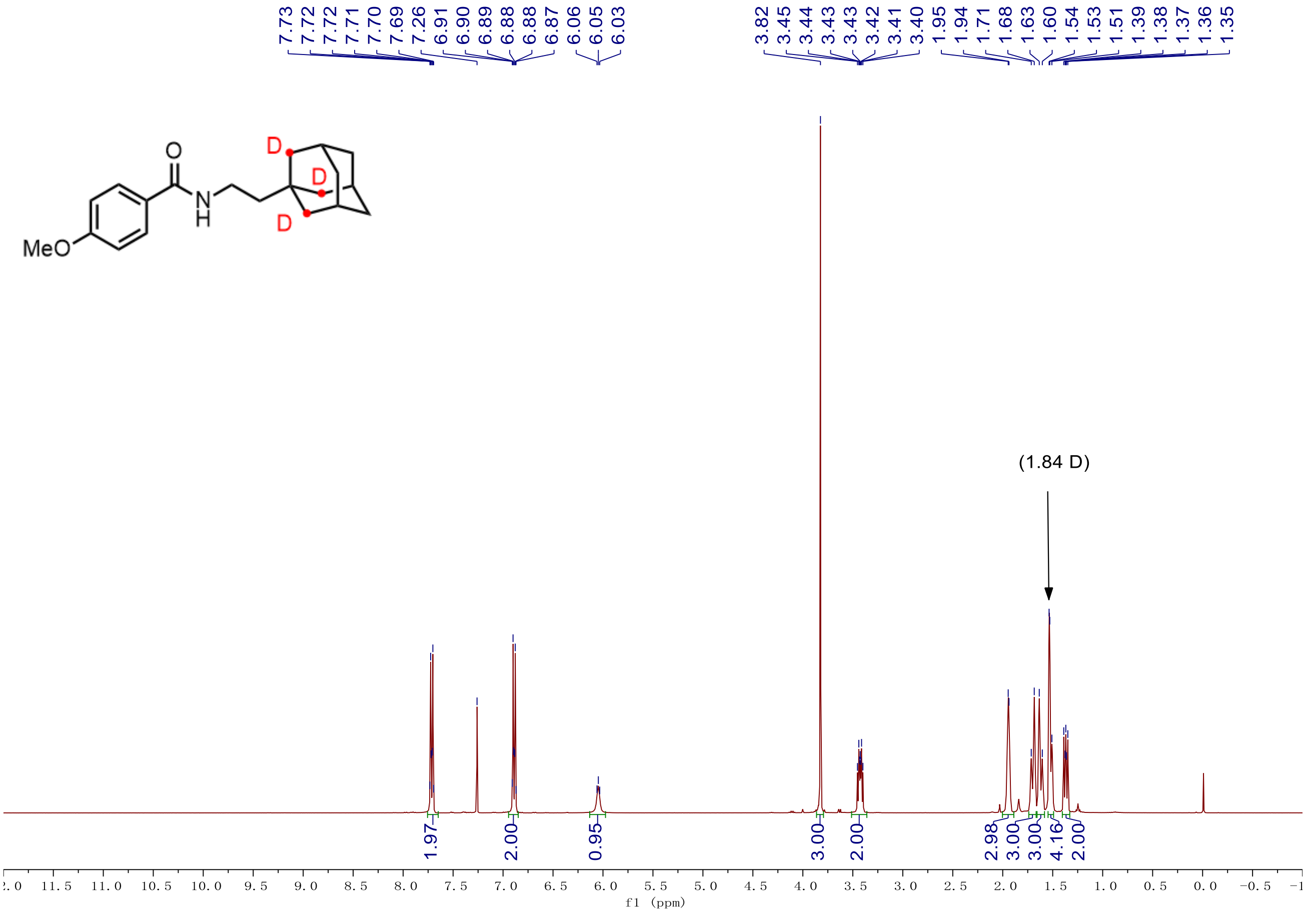

Supplementary Fig. 175. ^{1}H NMR (400 MHz, 298 K, Chloroform-*d*) spectrum of compound **4h**.

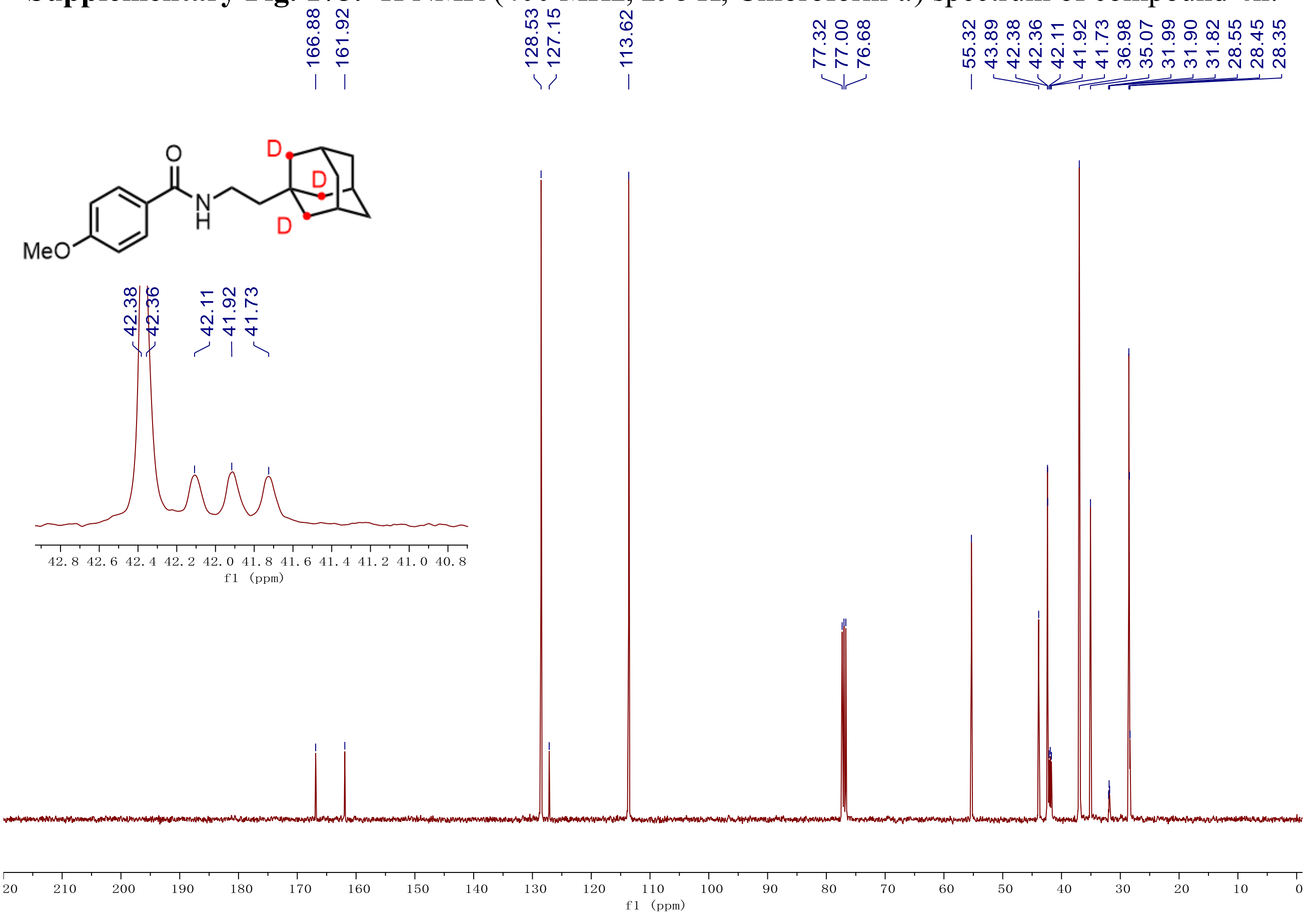

Supplementary Fig. 176. ^{13}C NMR (101 MHz, 298 K, Chloroform-*d*) spectrum of compound **4h**.

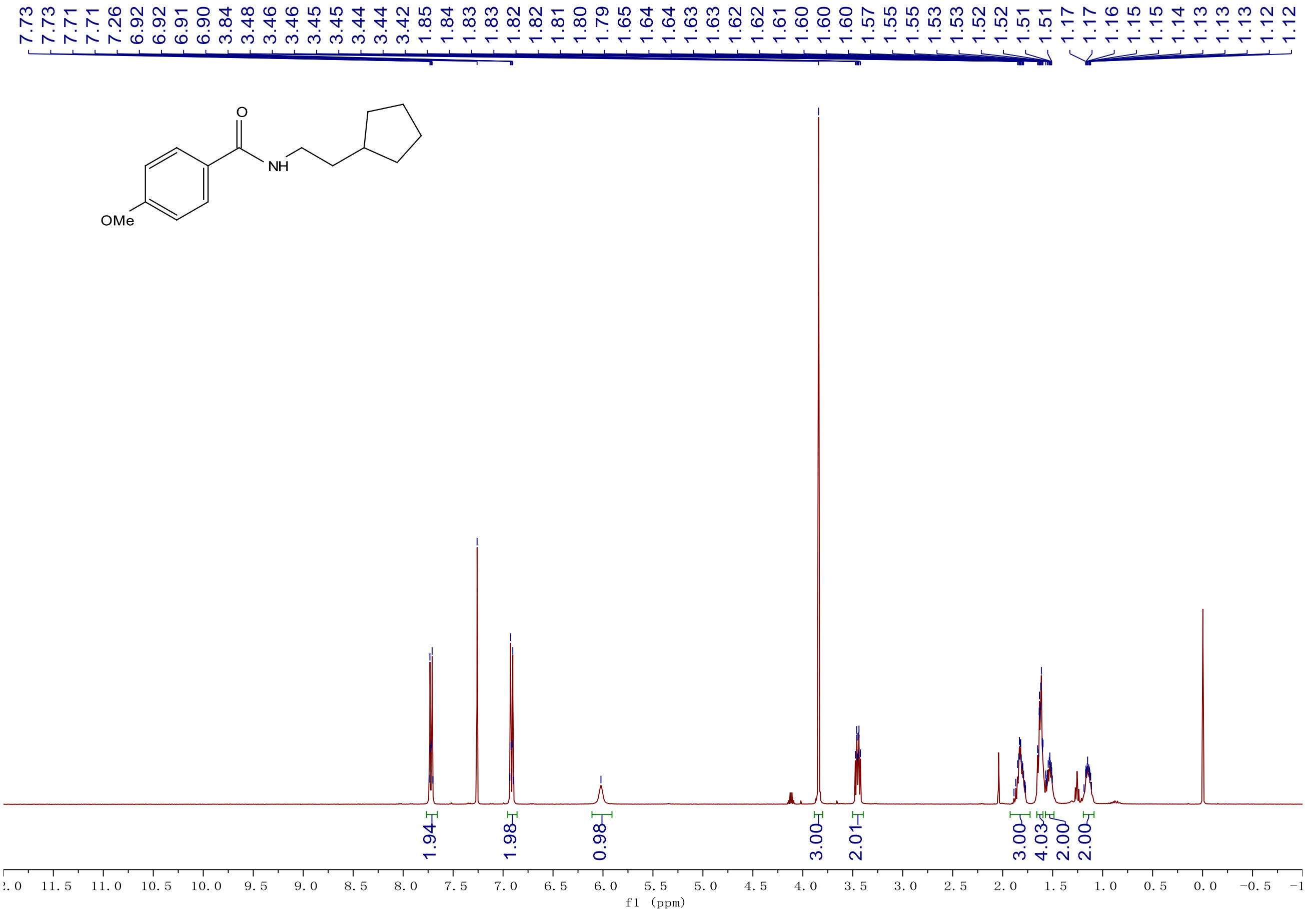

Supplementary Fig. 177. ^{1}H NMR (400 MHz, 298 K, Chloroform-*d*) spectrum of compound **1-i**.

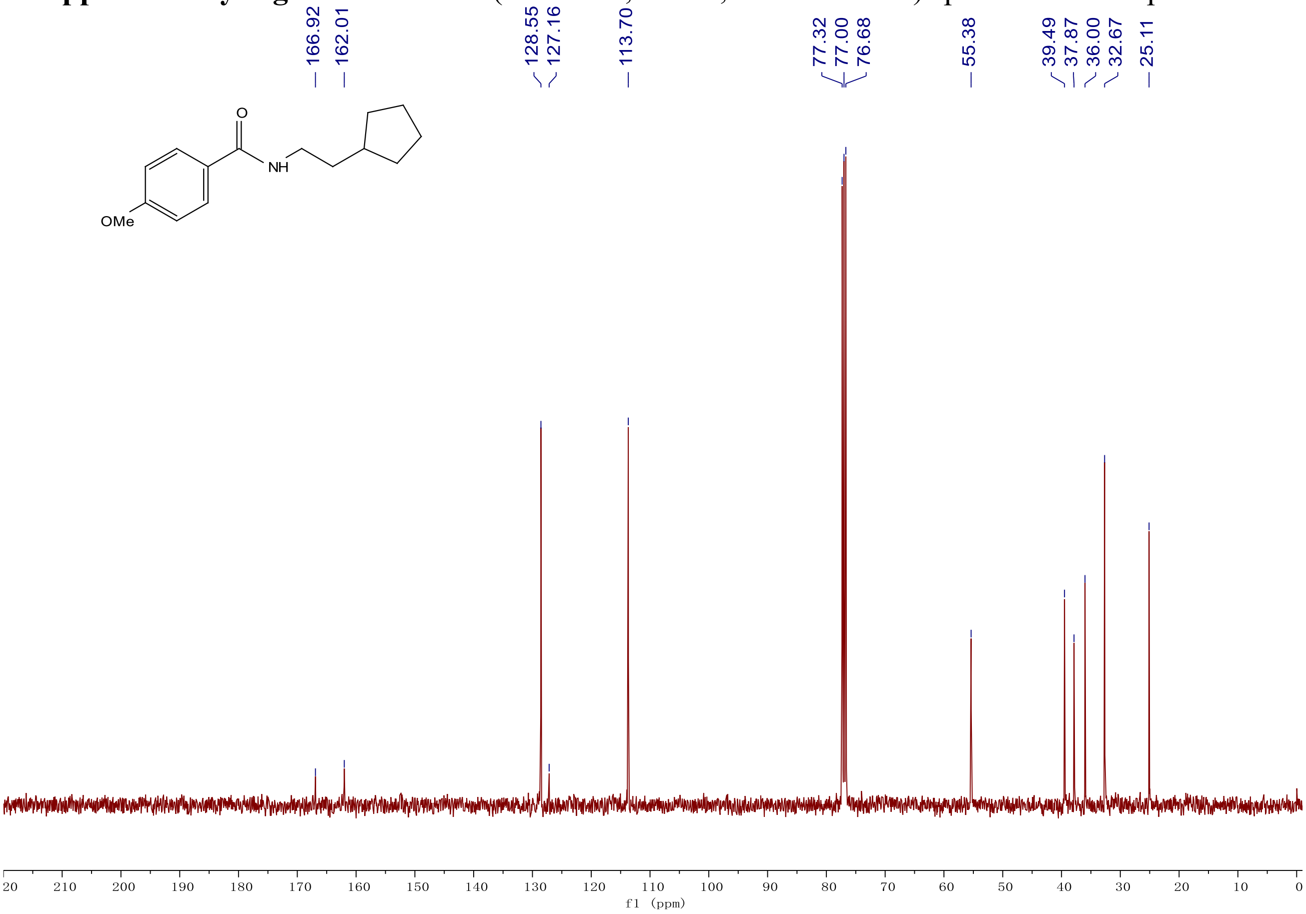

Supplementary Fig. 178. ^{13}C NMR (101 MHz, 298 K, Chloroform-*d*) spectrum of compound **1-i**.

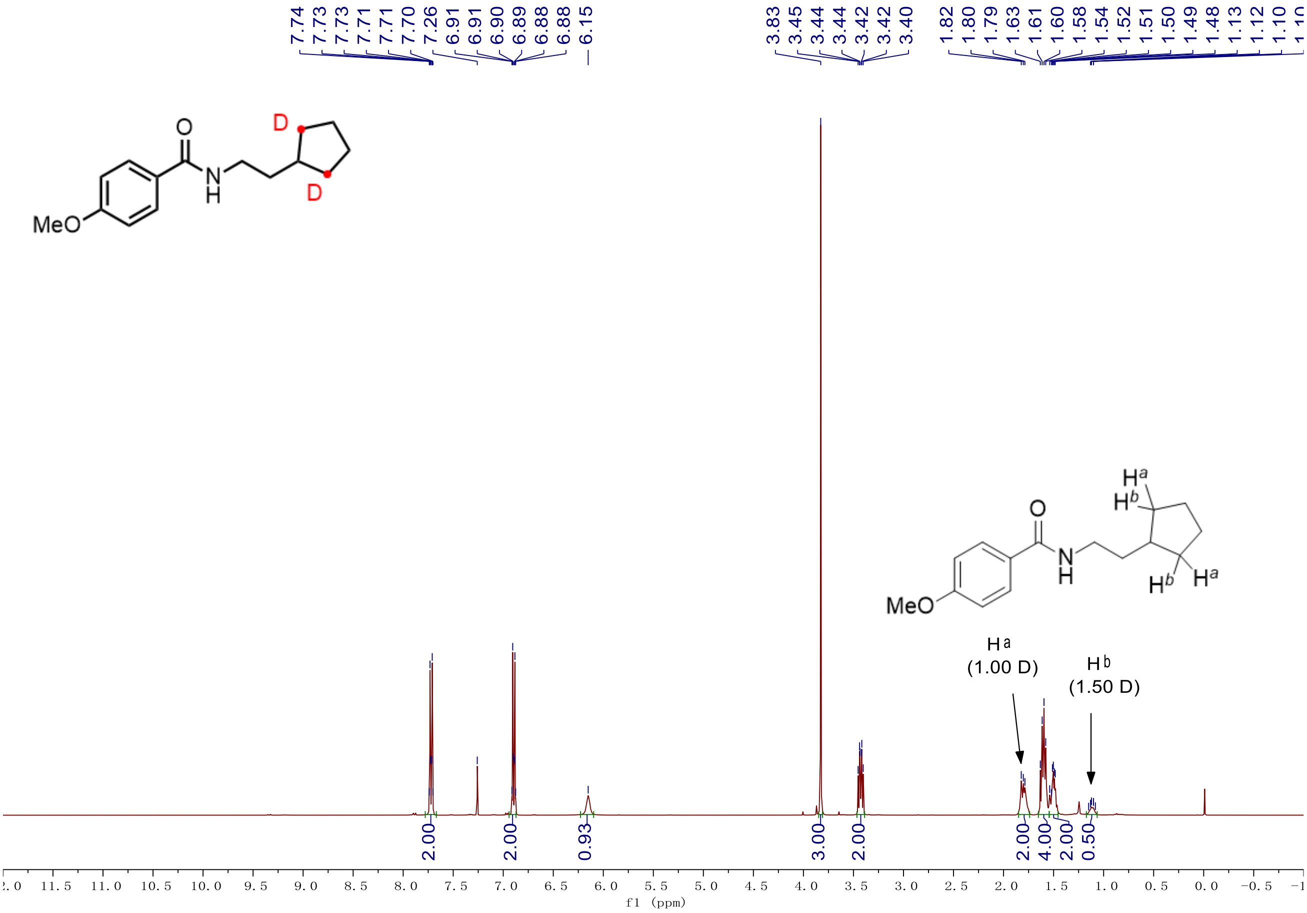

Supplementary Fig. 179. ^{1}H NMR (400 MHz, 298 K, Chloroform-d) spectrum of compound **4i**.

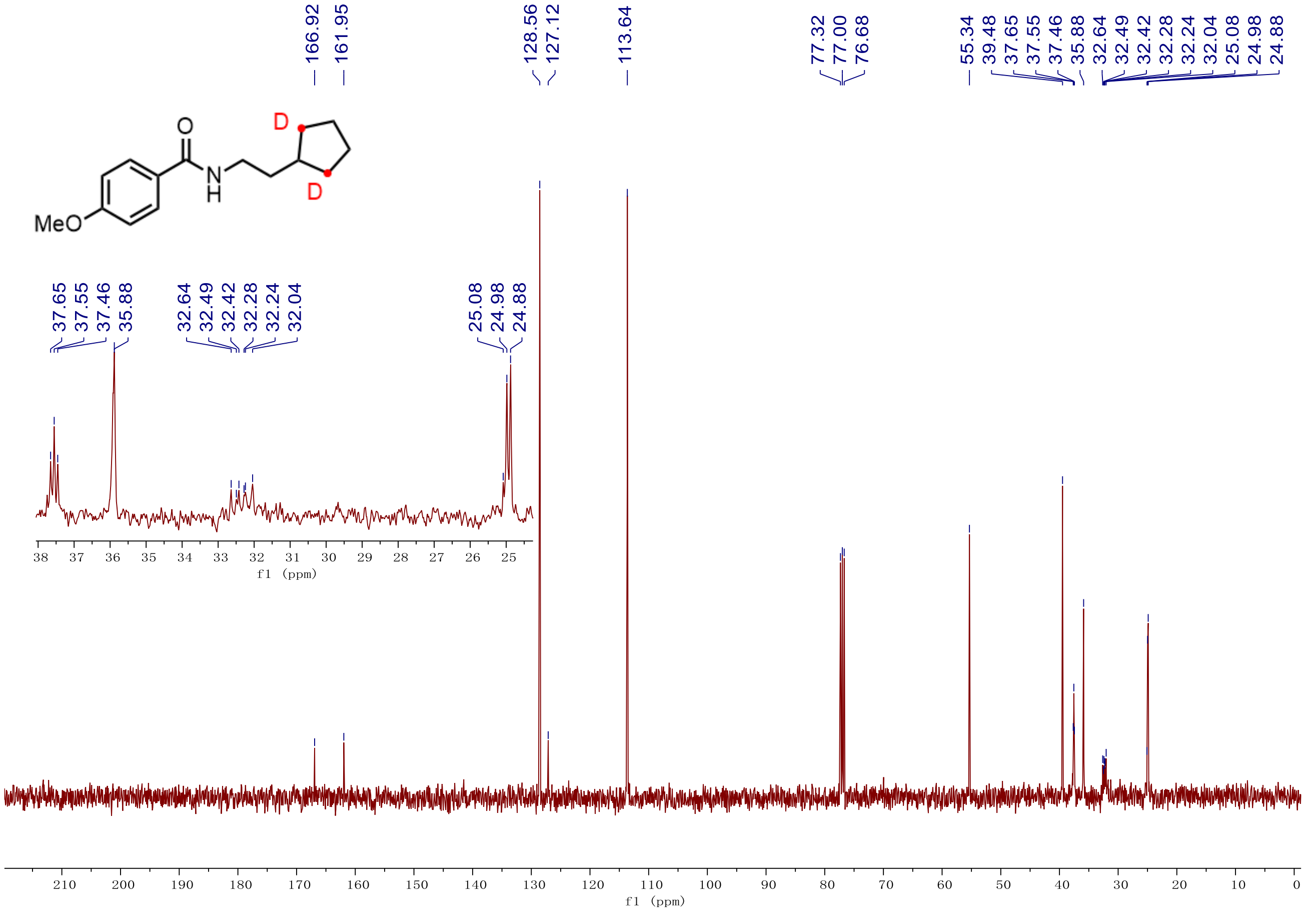

Supplementary Fig. 180. ^{13}C NMR (101 MHz, 298 K, Chloroform-d) spectrum of compound **4i**.

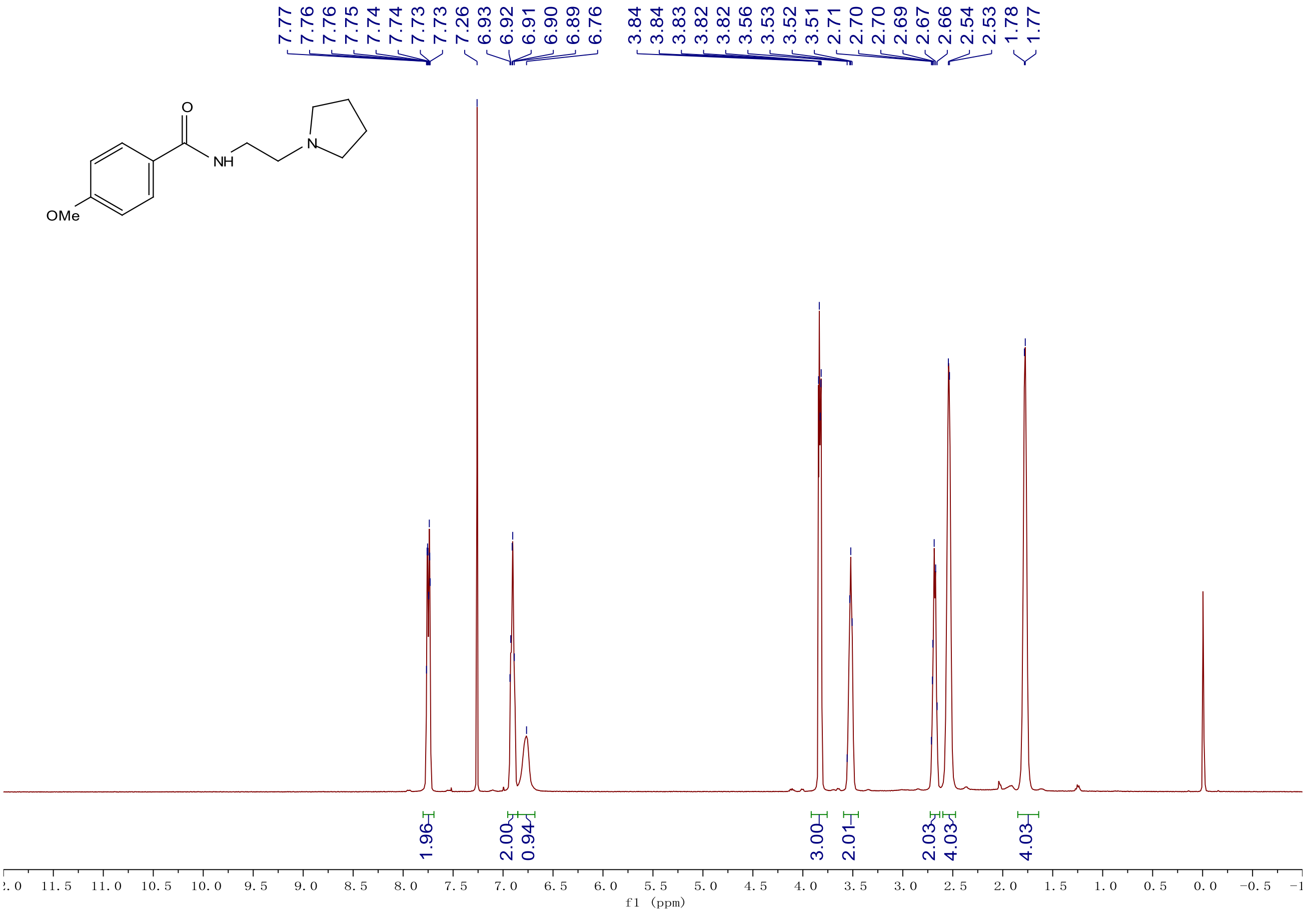

Supplementary Fig. 181. ^{1}H NMR (400 MHz, 298 K, Chloroform-*d*) spectrum of compound **1-j**.

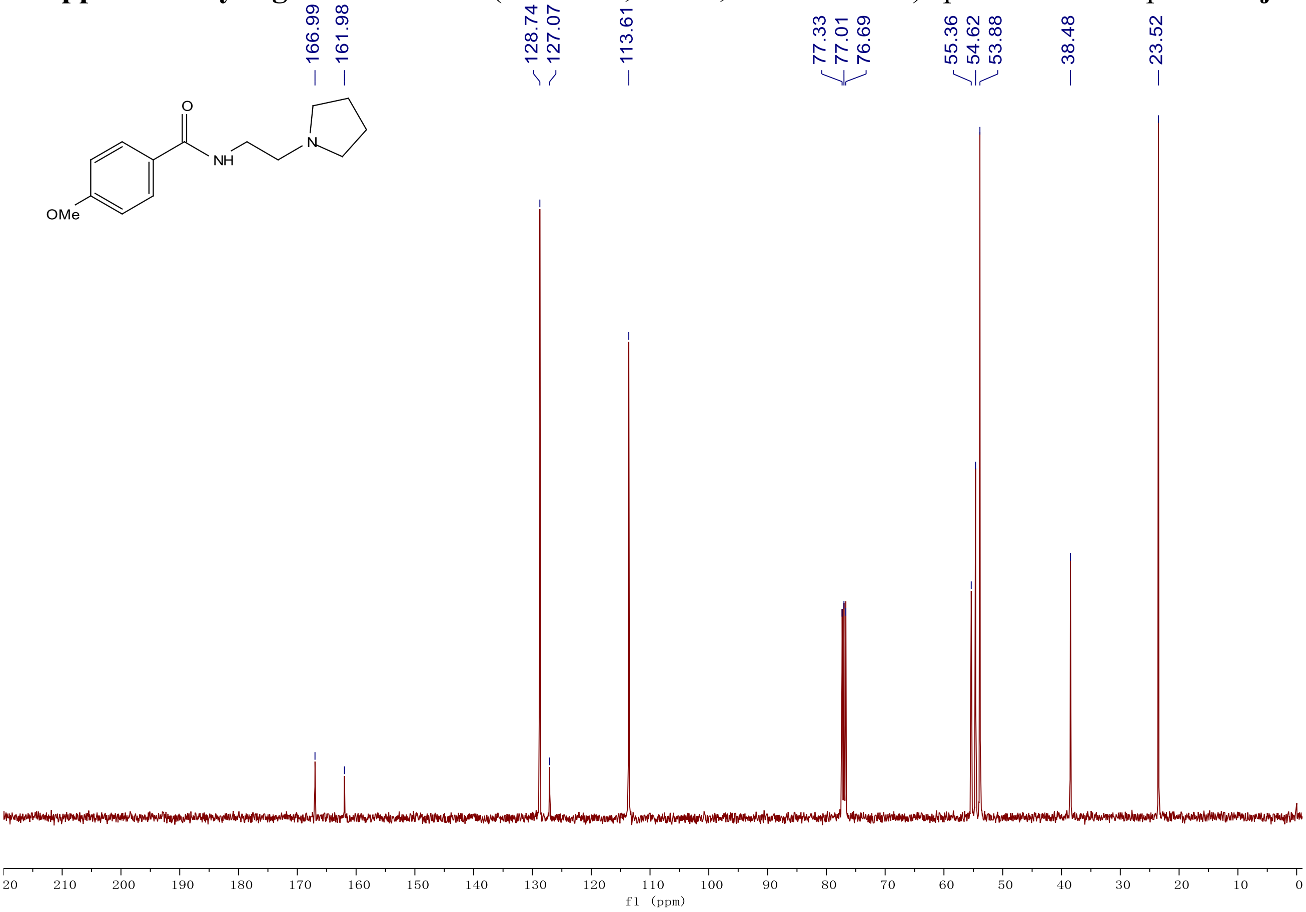

Supplementary Fig. 182. ^{13}C NMR (101 MHz, 298 K, Chloroform-*d*) spectrum of compound **1-j**.

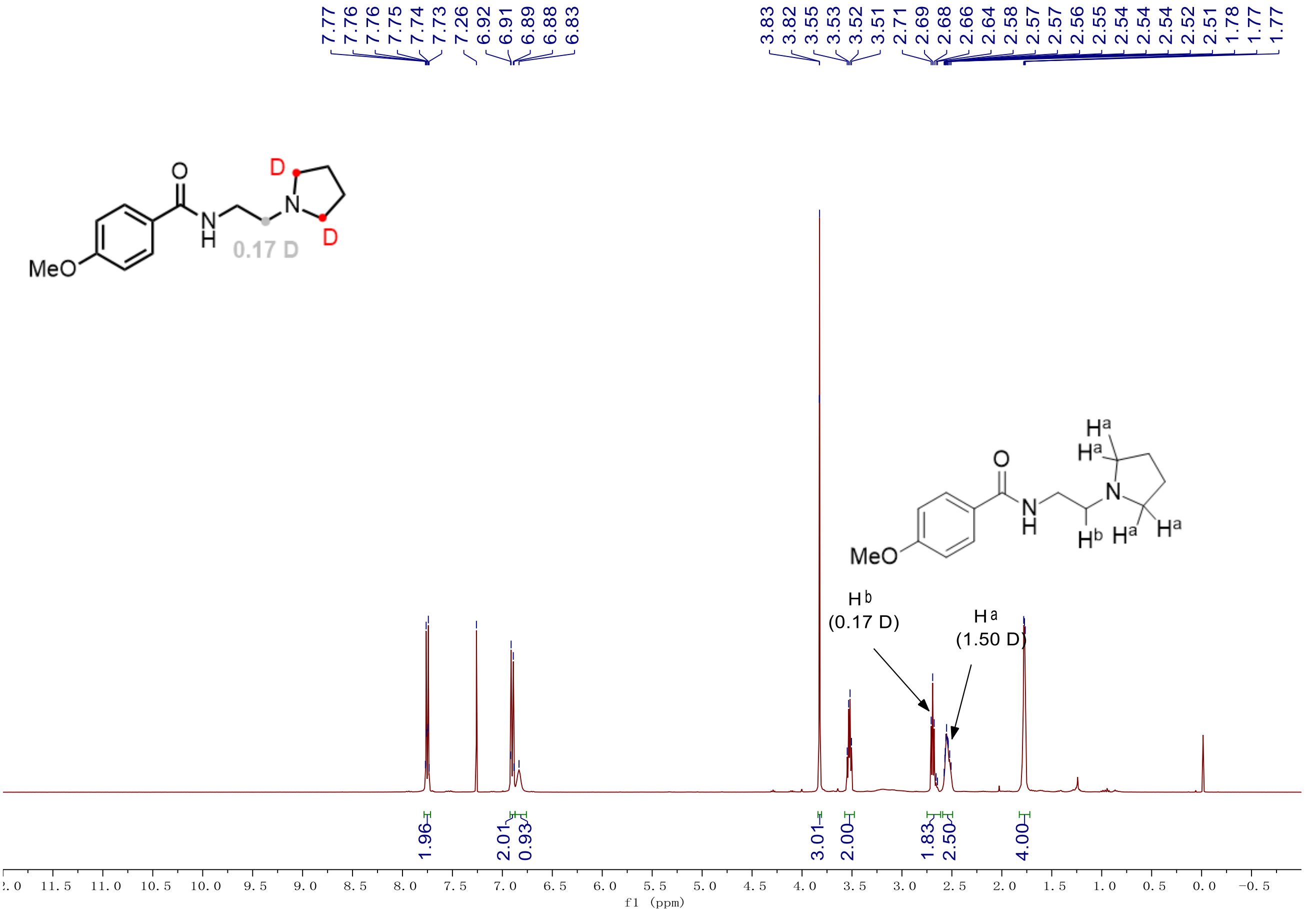

Supplementary Fig. 183. ^{1}H NMR (400 MHz, 298 K, Chloroform-*d*) spectrum of compound **4j**.

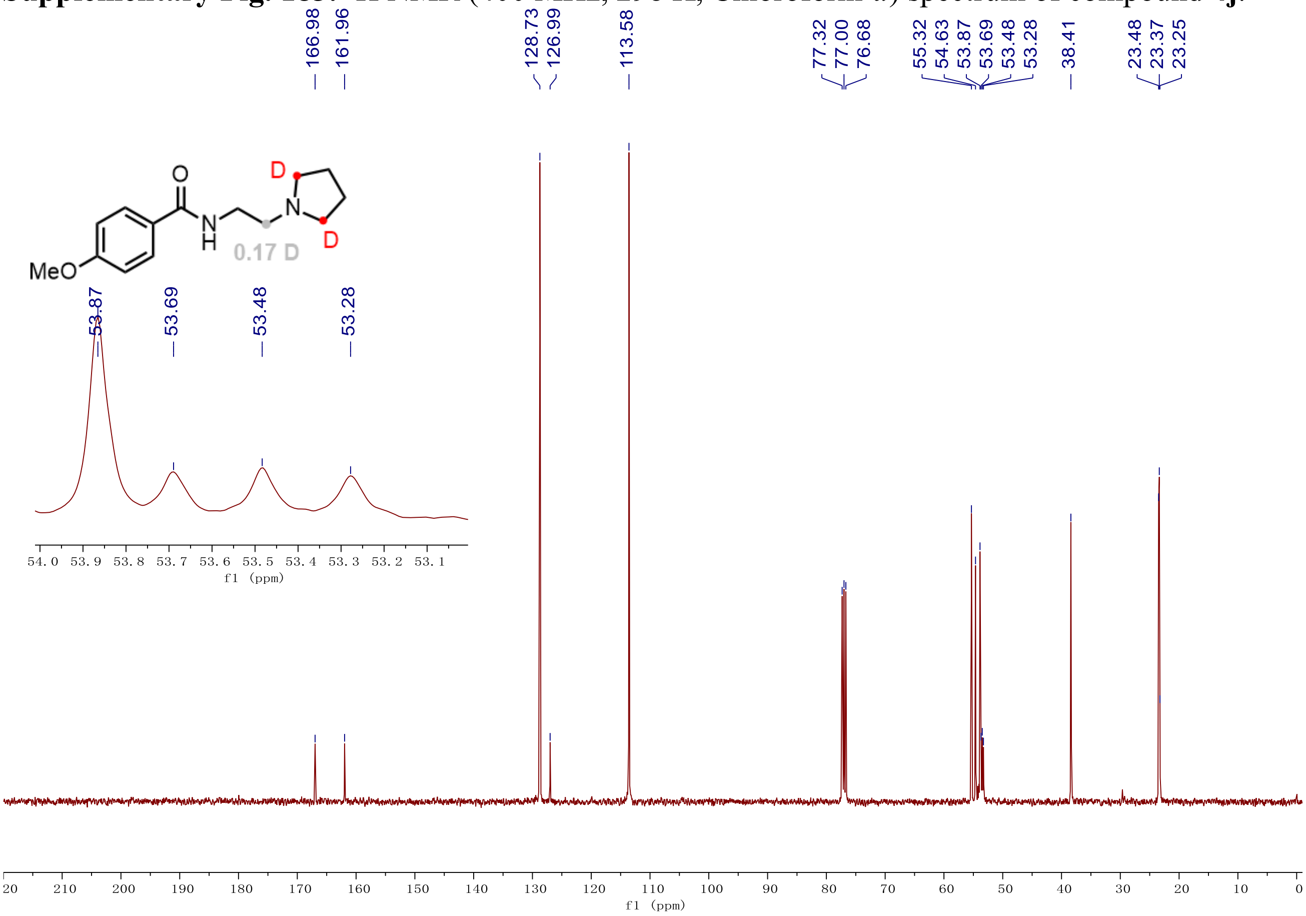

Supplementary Fig. 184. ^{13}C NMR (101 MHz, 298 K, Chloroform-*d*) spectrum of compound **4j**.

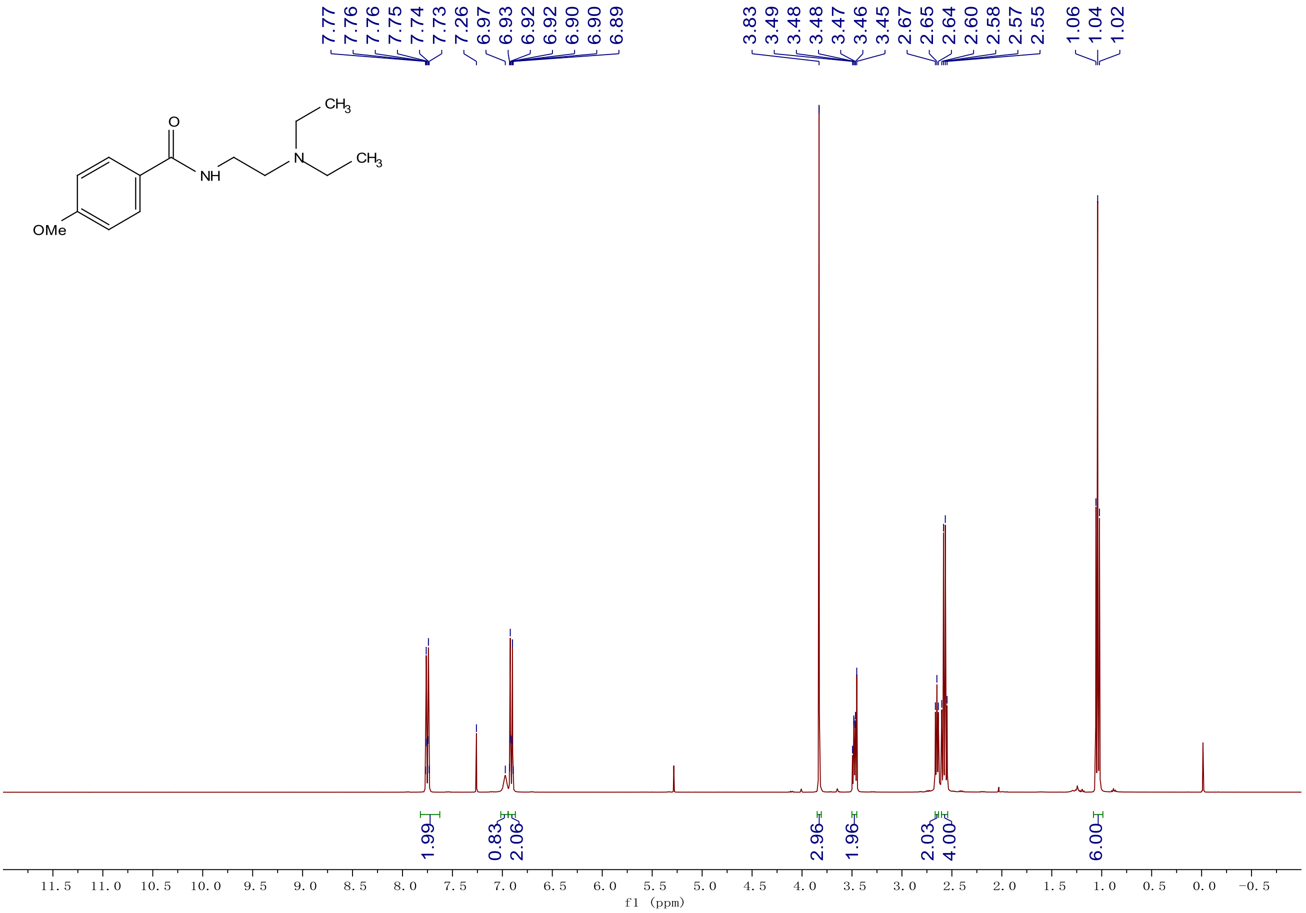

Supplementary Fig. 185. ^{1}H NMR (400 MHz, 298 K, Chloroform-*d*) spectrum of compound **1-k**.

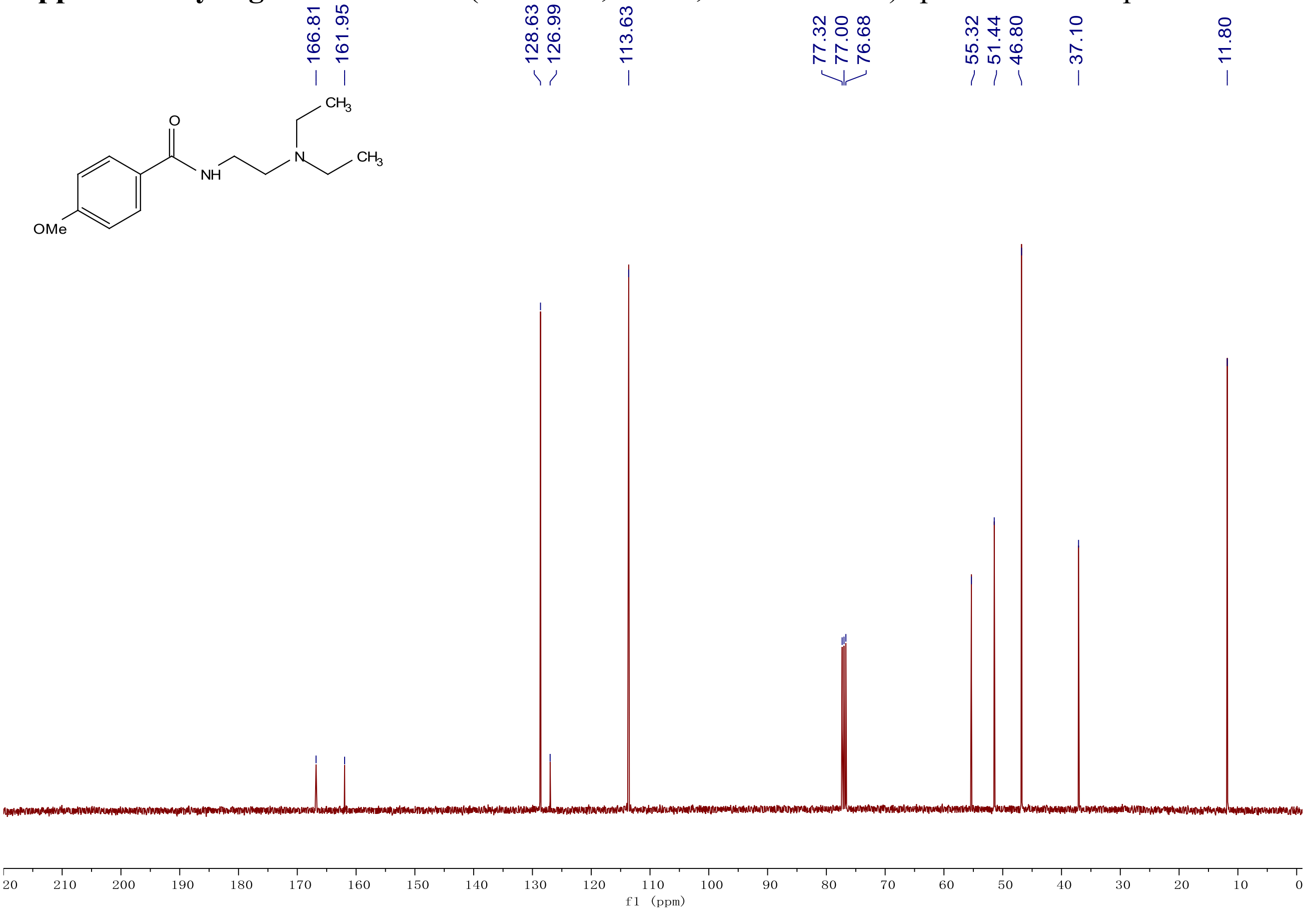

Supplementary Fig. 186. ^{13}C NMR (101 MHz, 298 K, Chloroform-*d*) spectrum of compound **1-k**.

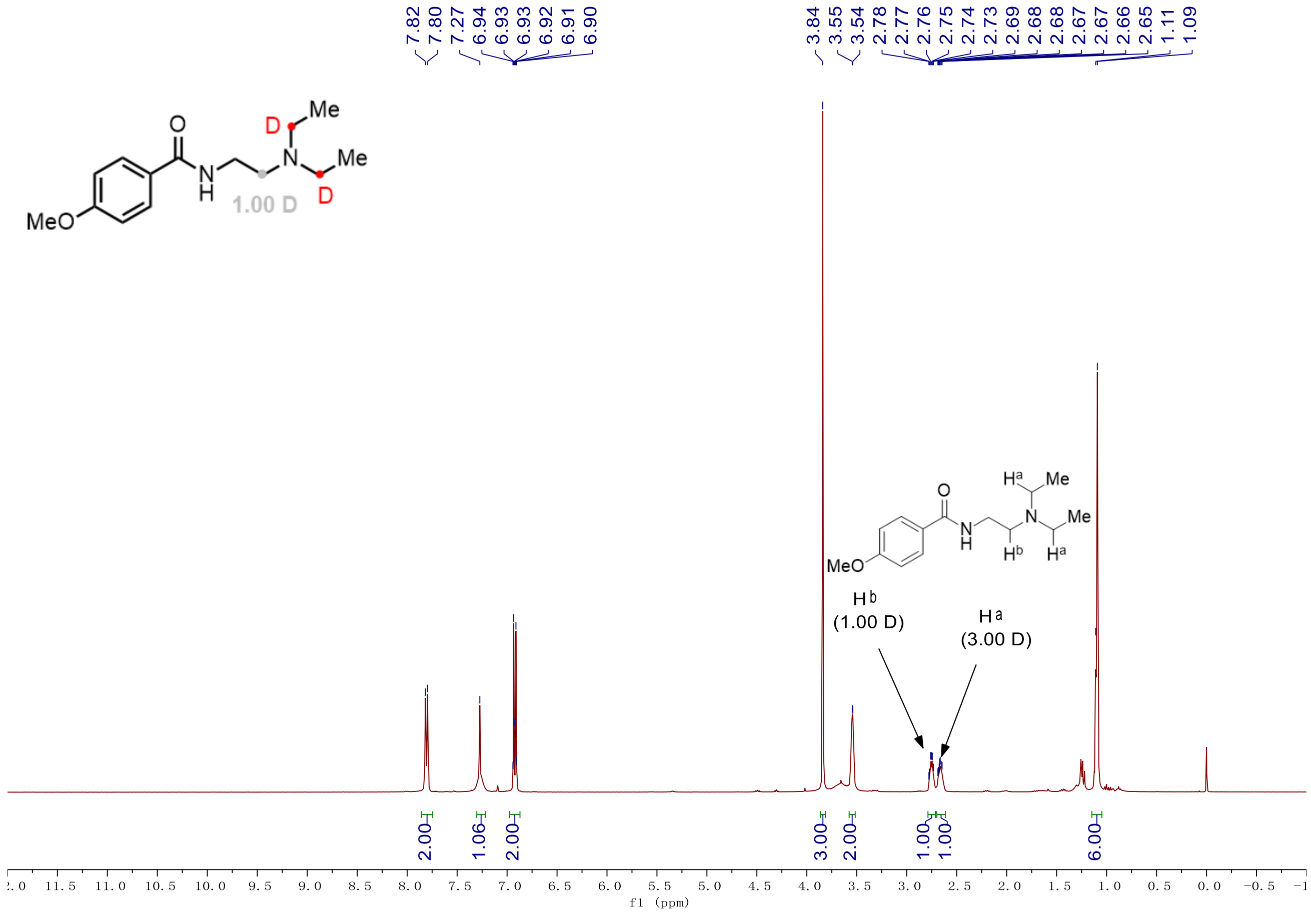

Supplementary Fig. 187. ^{1}H NMR (400 MHz, 298 K, Chloroform-*d*) spectrum of compound **4k**.

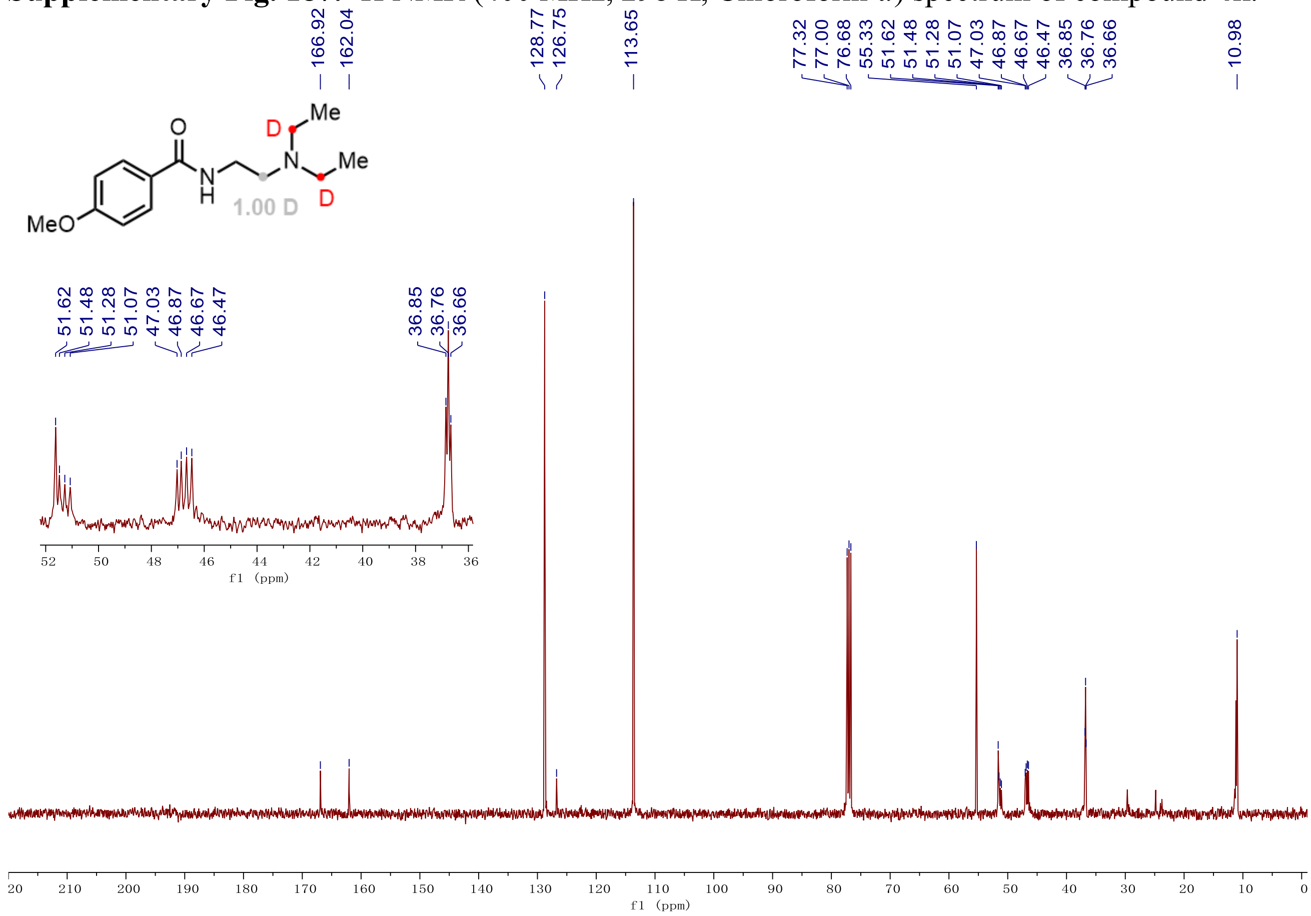

Supplementary Fig. 188. ^{13}C NMR (101MHz, 298 K, Chloroform-*d*) spectrum of compound **4k**.

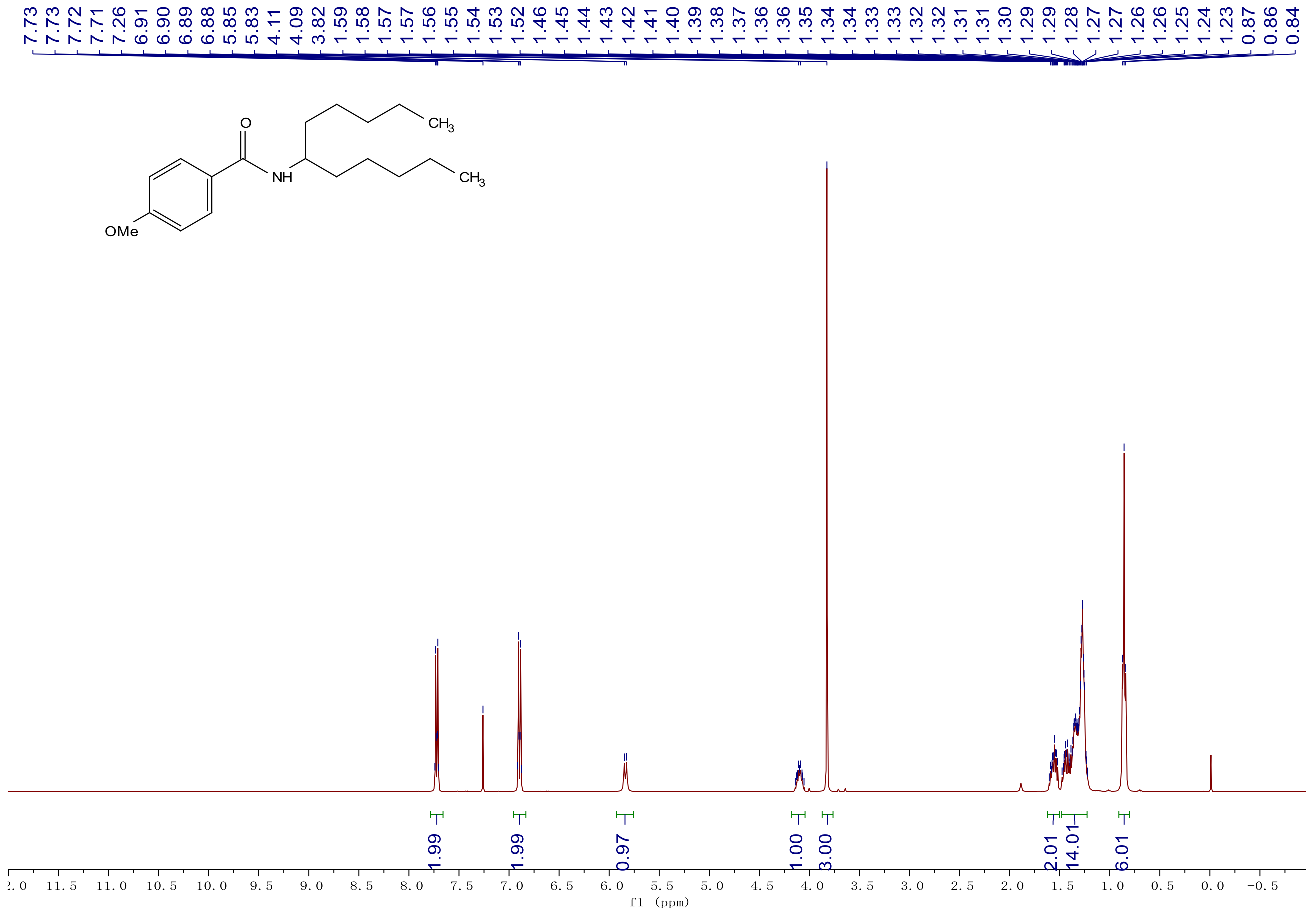

Supplementary Fig. 189. ^{1}H NMR (400 MHz, 298 K, Chloroform-*d*) spectrum of compound **1-l**.

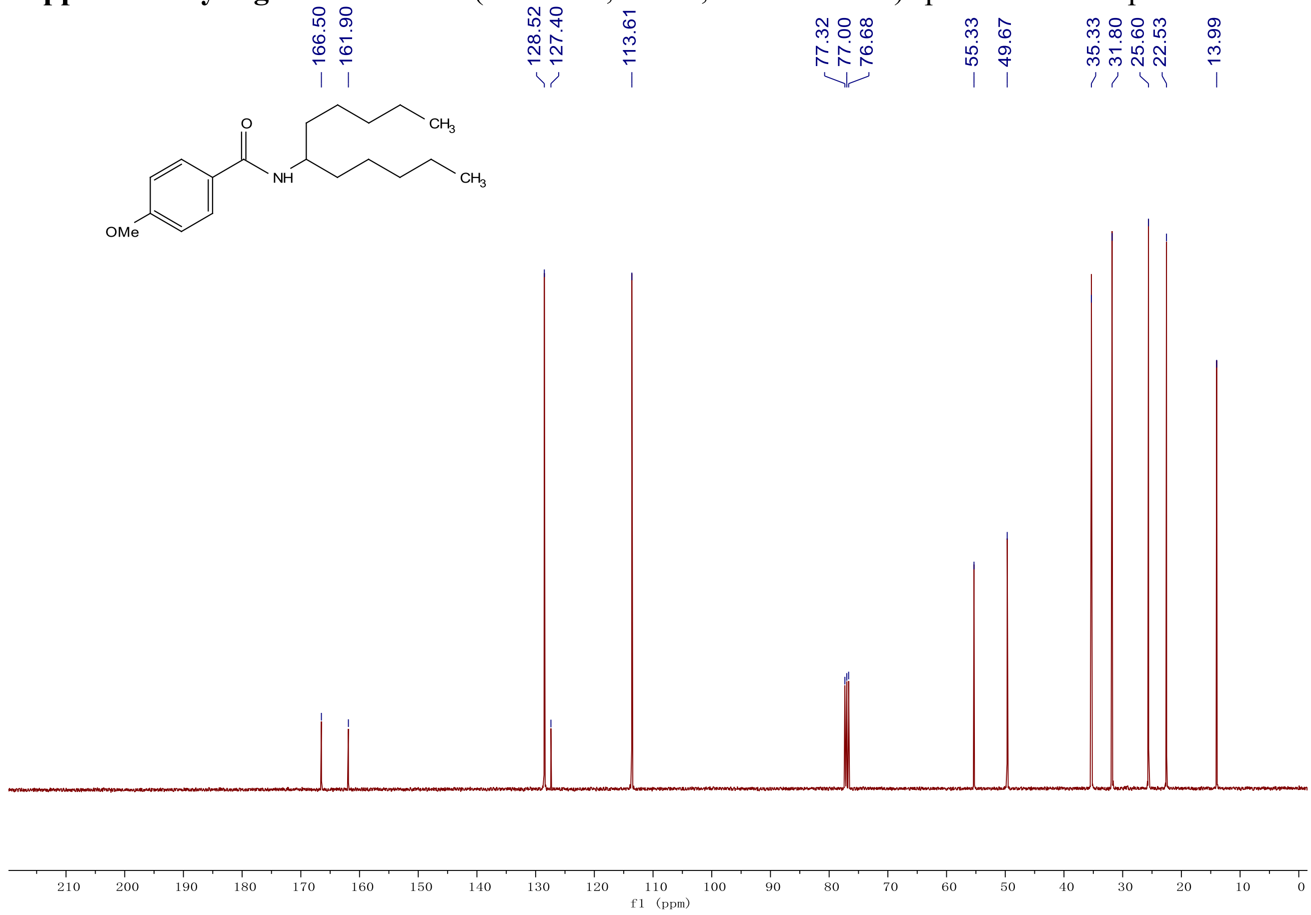

Supplementary Fig. 190. ^{13}C NMR (101 MHz, 298 K, Chloroform-*d*) spectrum of compound **1-l**.

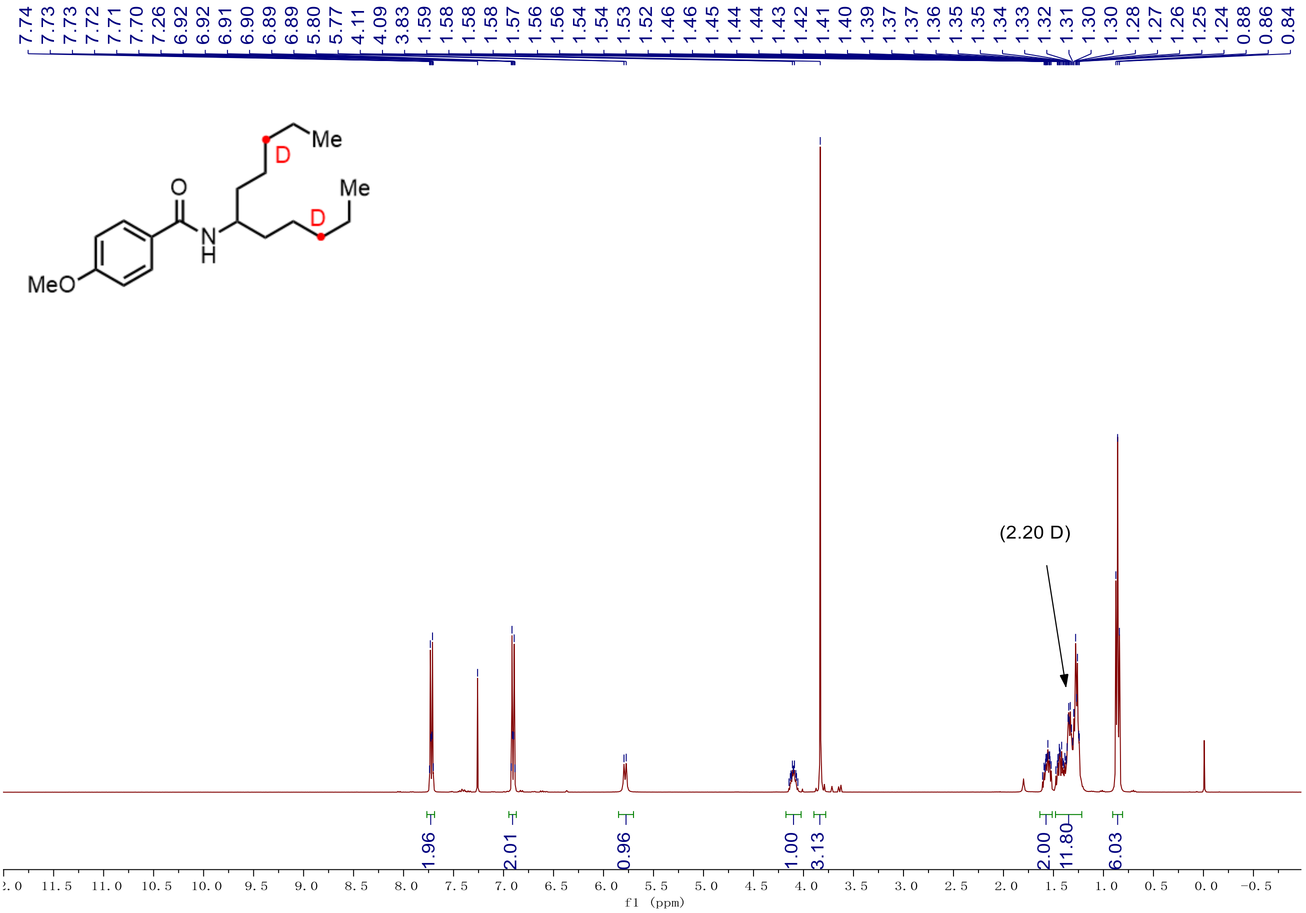

Supplementary Fig. 191. ^{1}H NMR (400 MHz, 298 K, Chloroform-*d*) spectrum of compound **4l**.

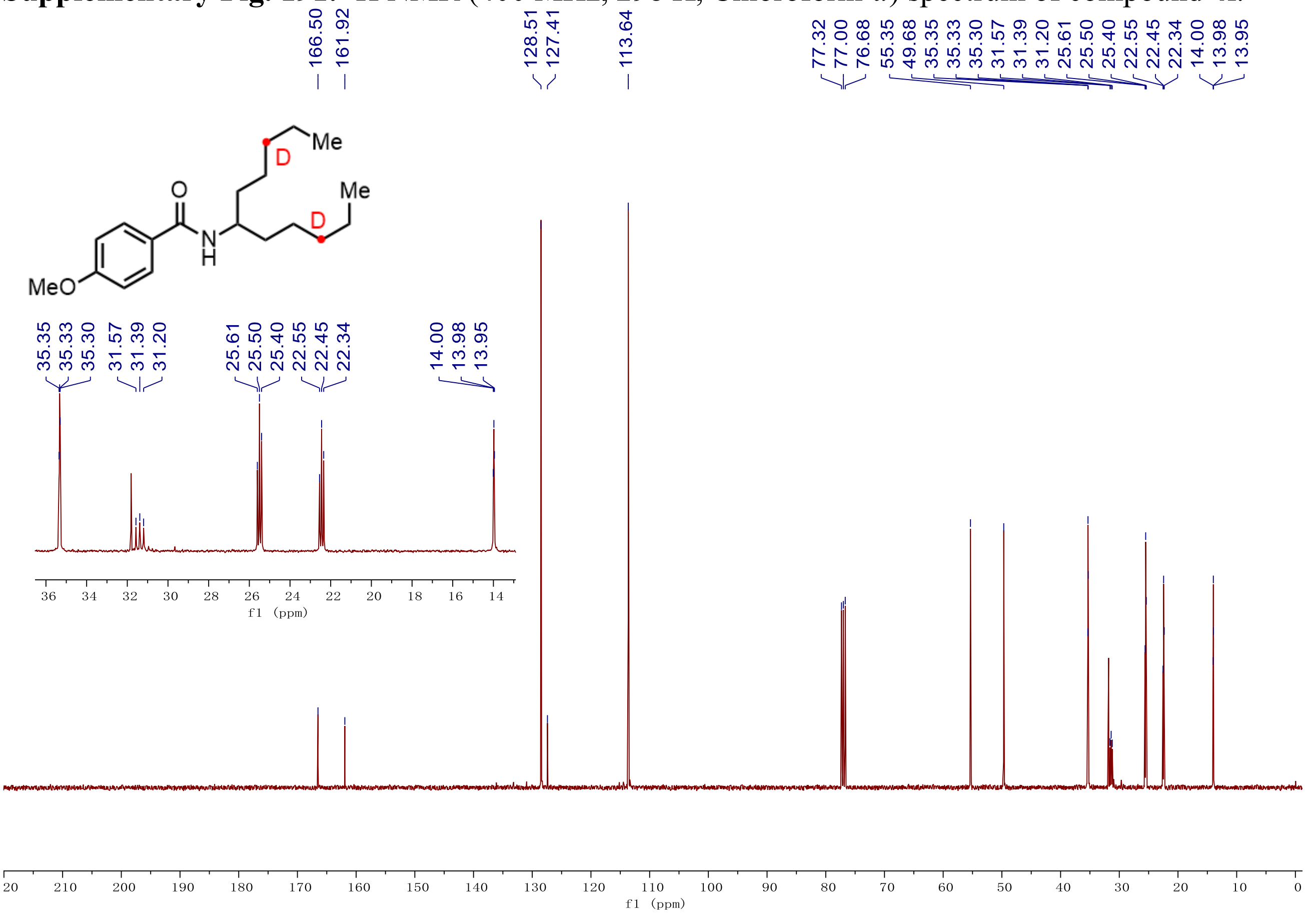

Supplementary Fig. 192. ^{13}C NMR (101 MHz, 298 K, Chloroform-*d*) spectrum of compound **4l**.

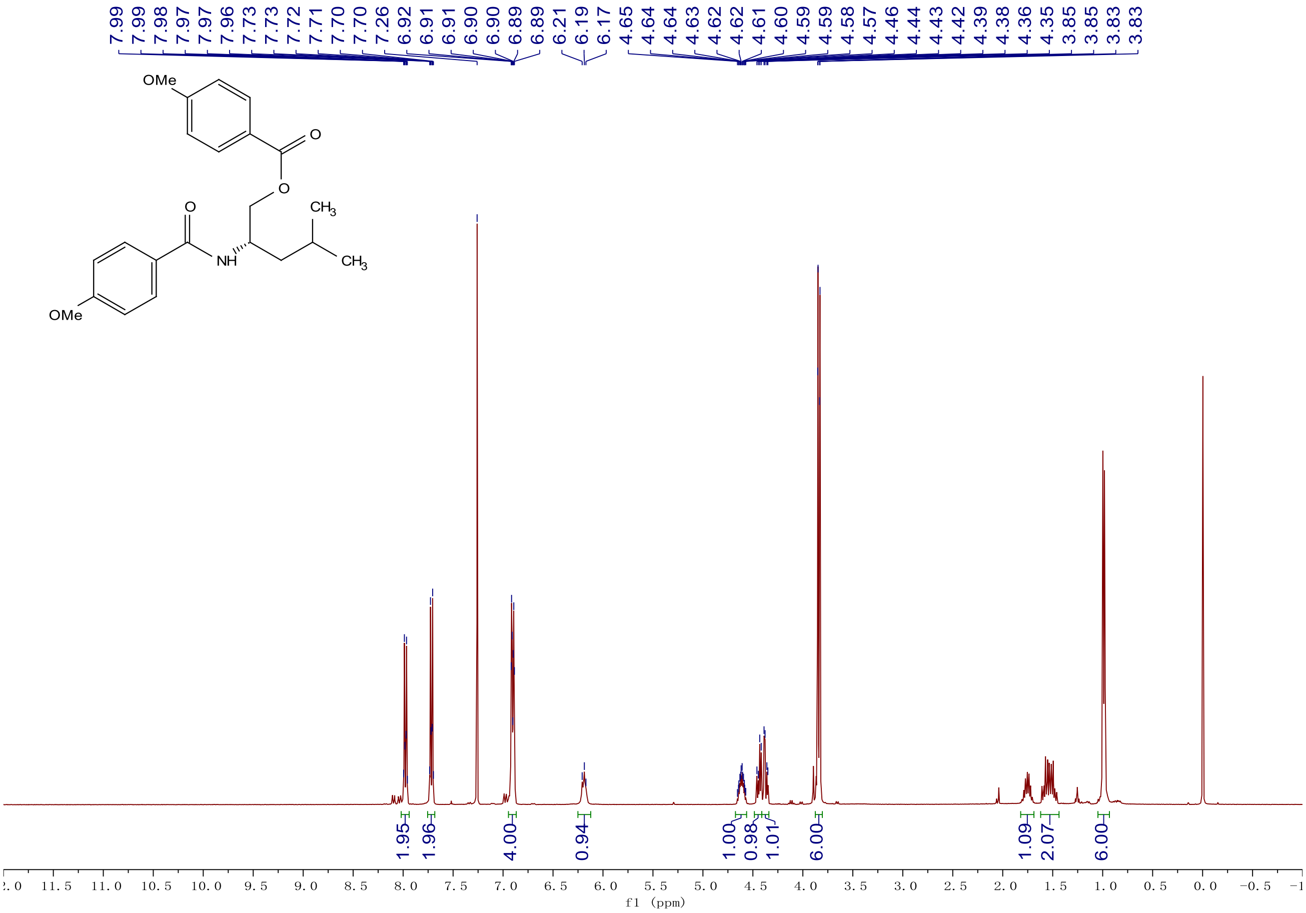

Supplementary Fig. 193. ^{1}H NMR (400 MHz, 298 K, Chloroform-*d*) spectrum of compound **1-m**.

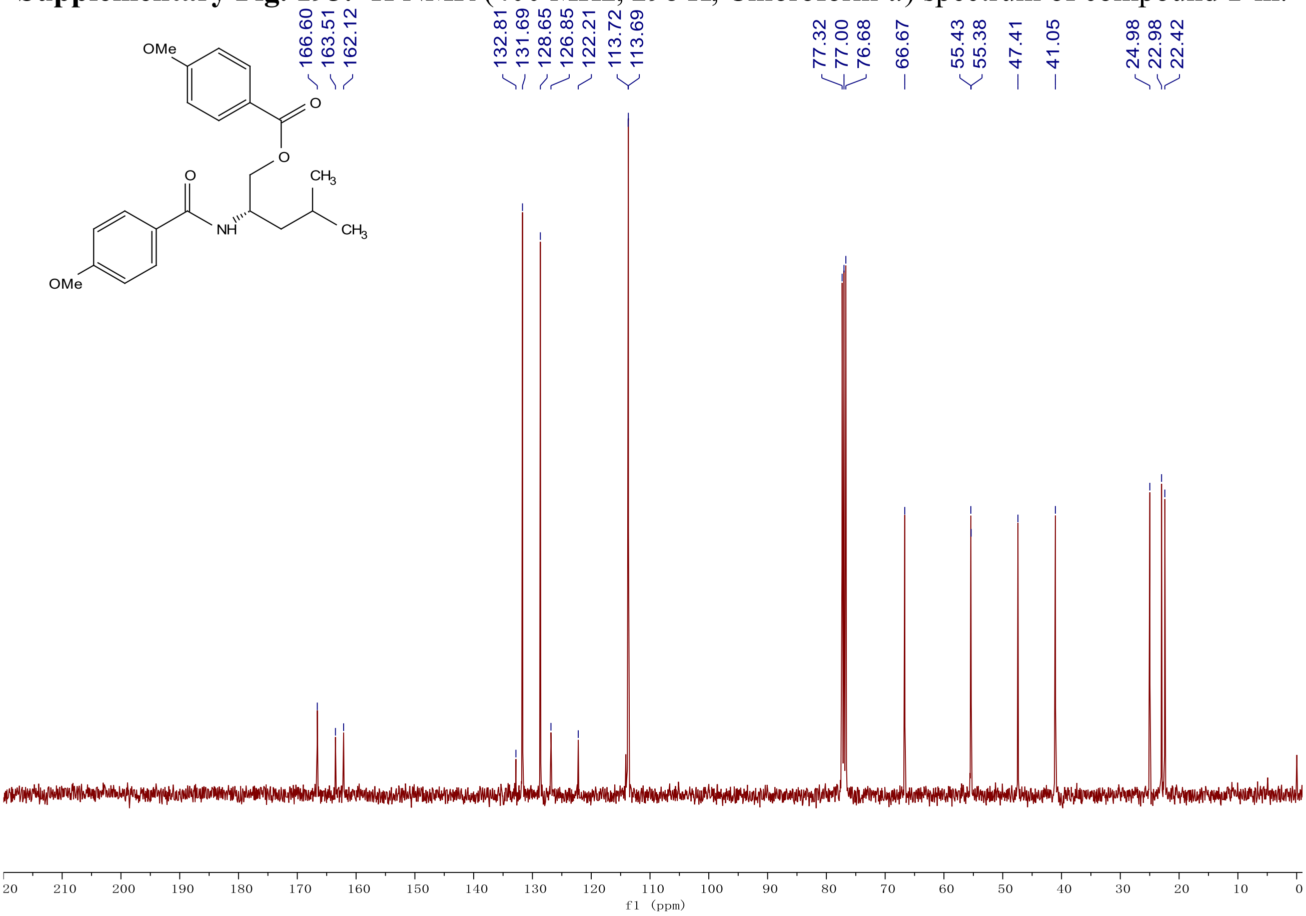

Supplementary Fig. 194. ^{13}C NMR (101 MHz, 298 K, Chloroform-*d*) spectrum of compound **1-m**.

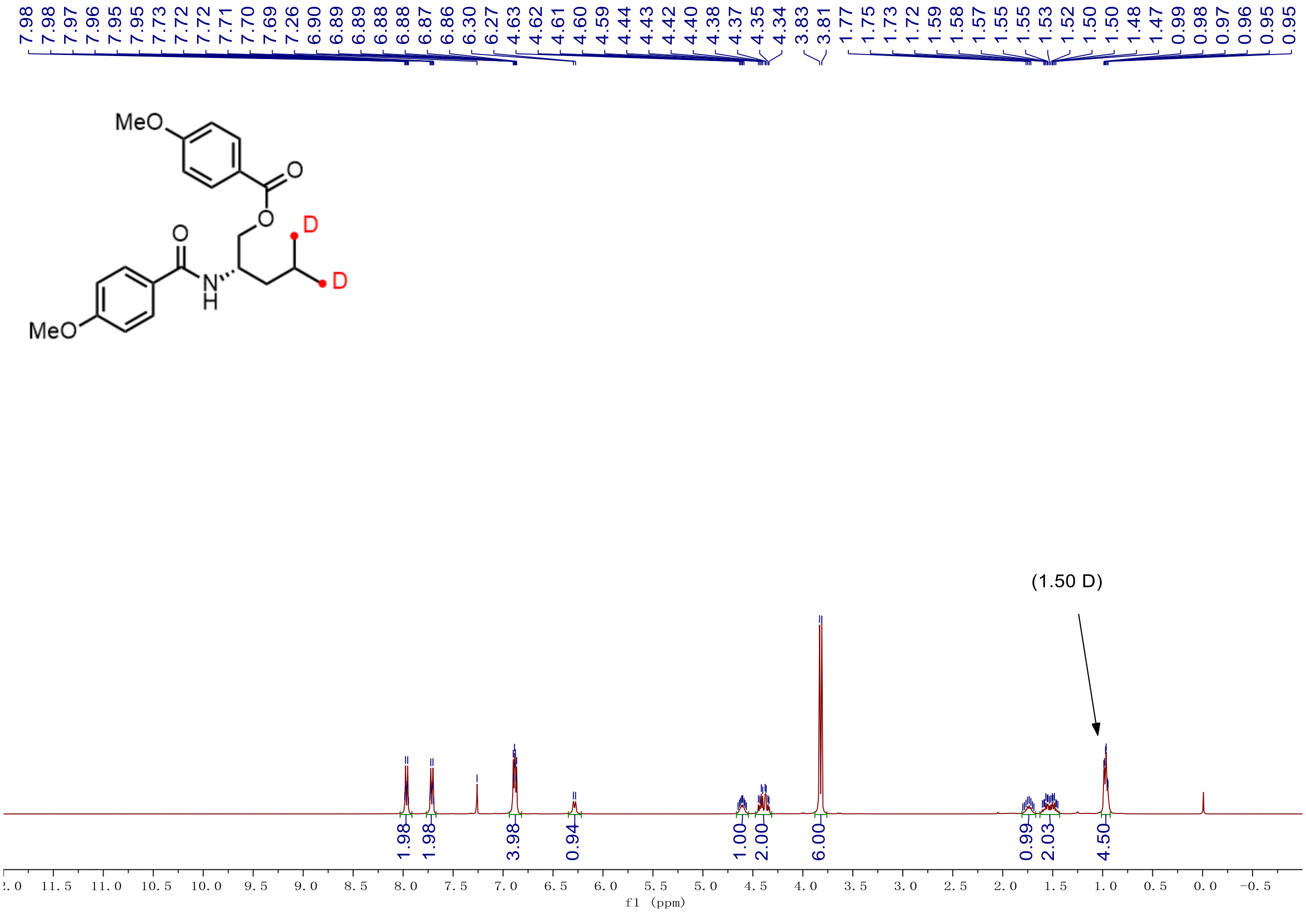

Supplementary Fig. 195. ^{1}H NMR (400 MHz, 298 K, Chloroform-*d*) spectrum of compound **4m**.

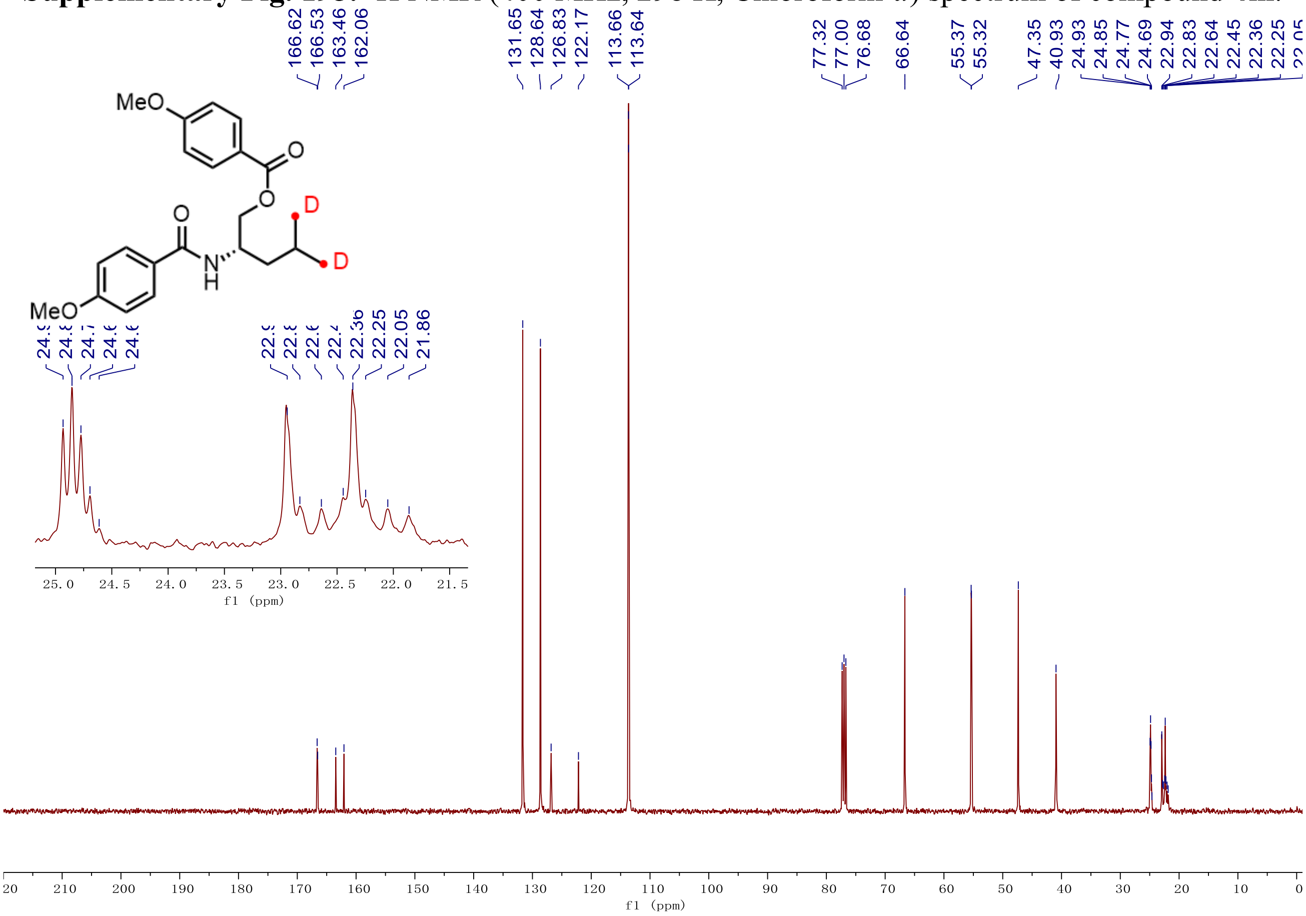

Supplementary Fig. 196. ^{13}C NMR (101 MHz, 298 K, Chloroform-*d*) spectrum of compound **4m**.

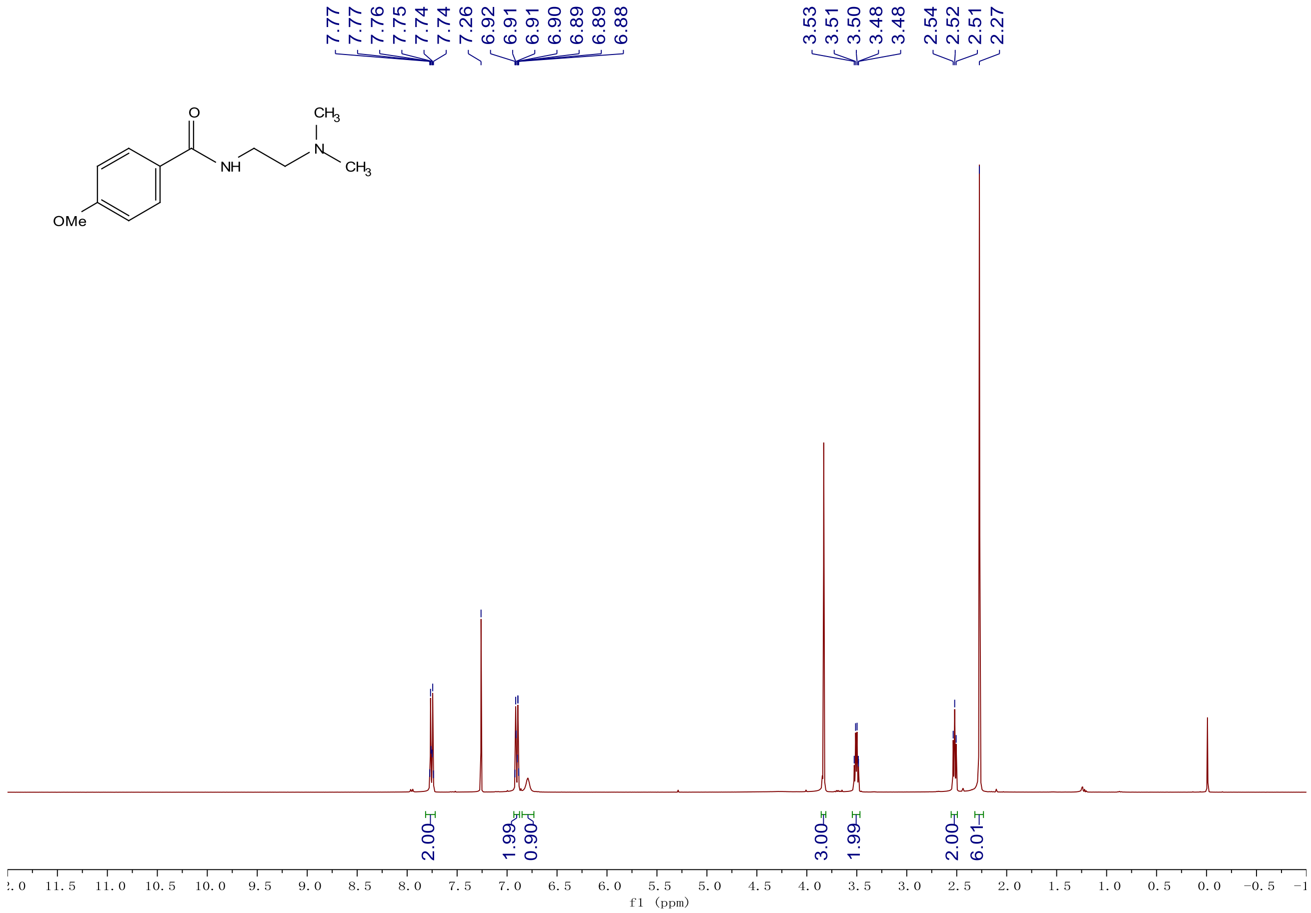

Supplementary Fig. 197. ^{1}H NMR (400 MHz, 298 K, Chloroform-*d*) spectrum of compound **1-n**.

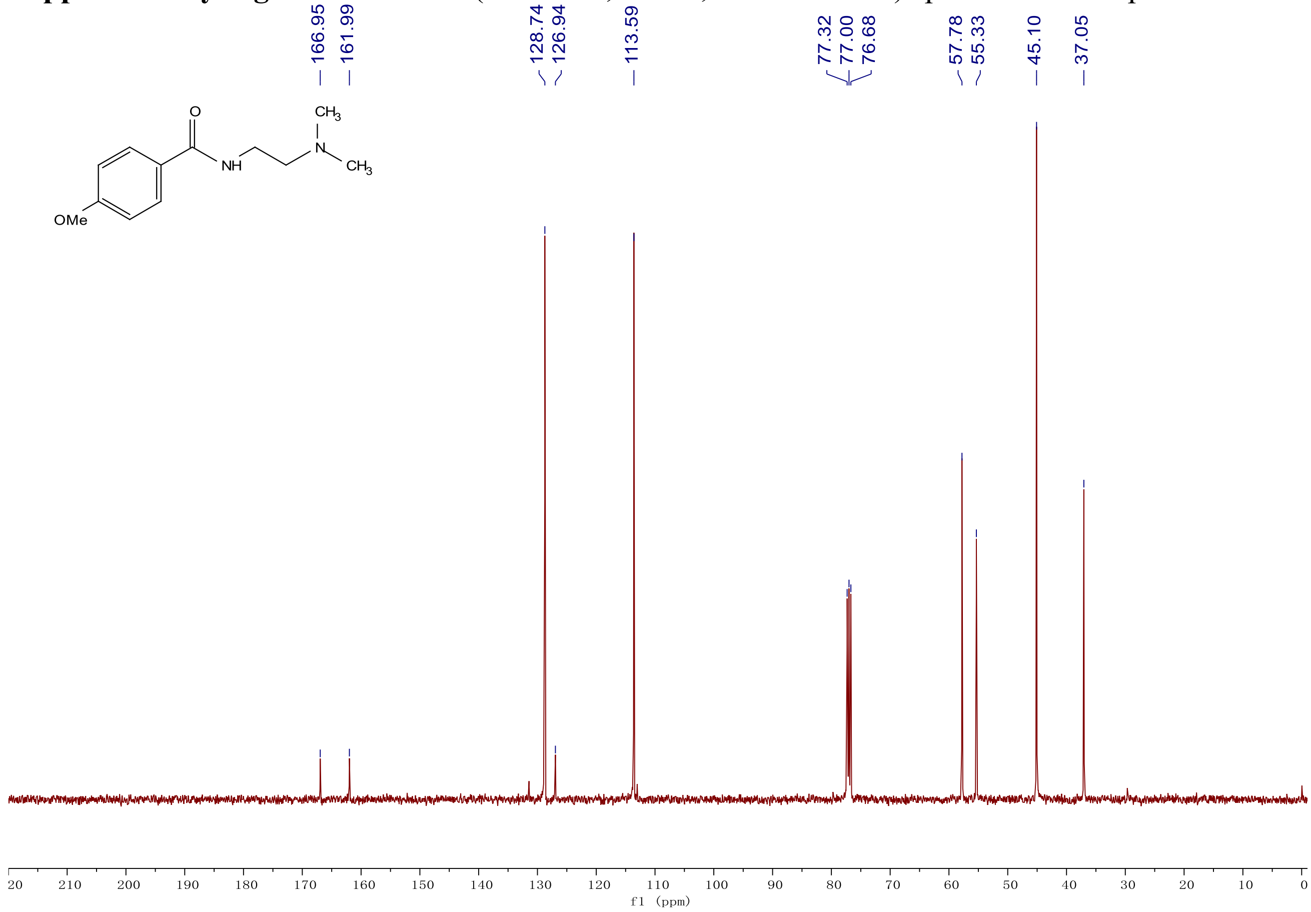

Supplementary Fig. 198. ^{13}C NMR (101 MHz, 298 K, Chloroform-*d*) spectrum of compound **1-n**.

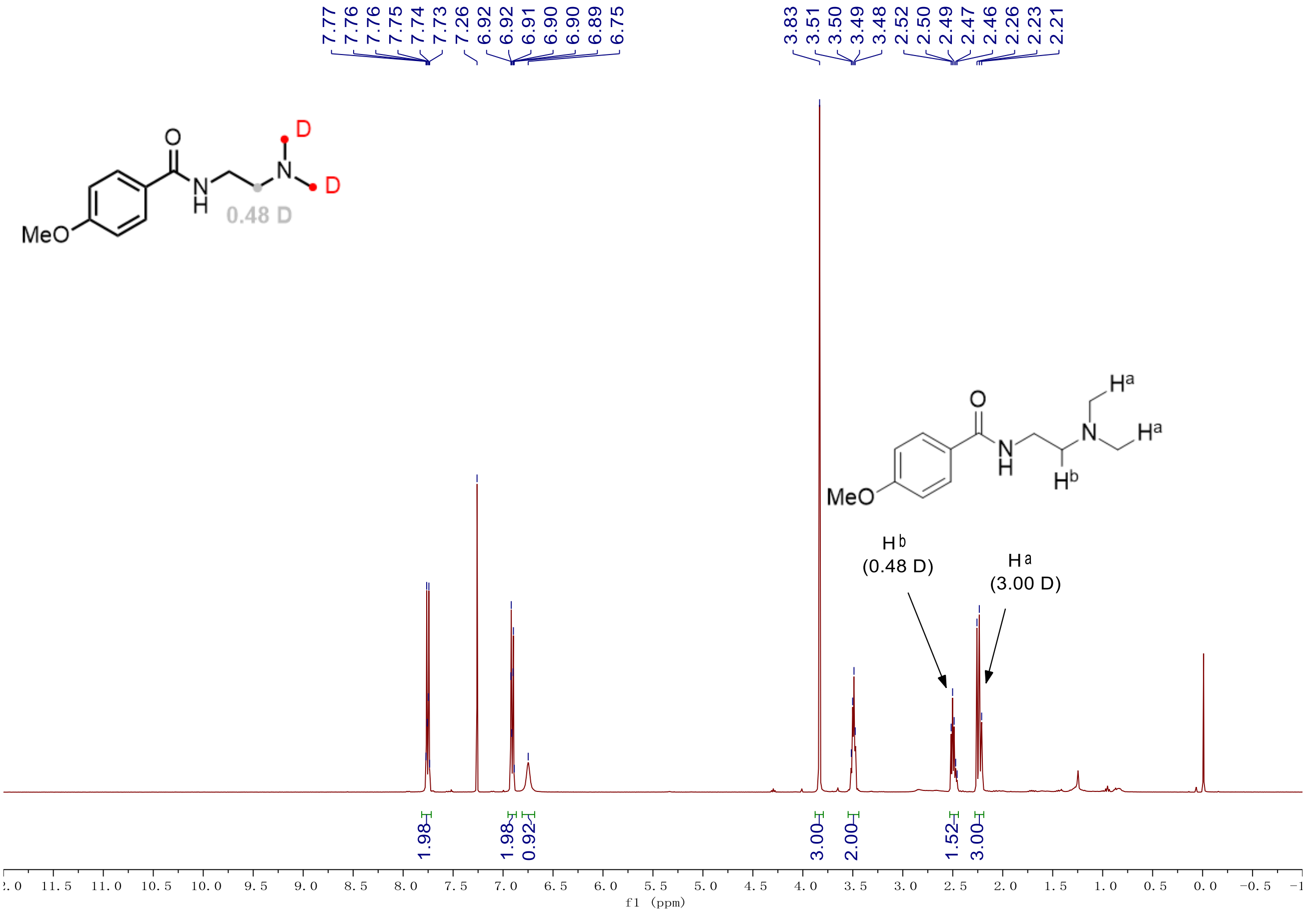

Supplementary Fig. 199. ^{1}H NMR (400 MHz, 298 K, Chloroform-*d*) spectrum of compound **4n**.

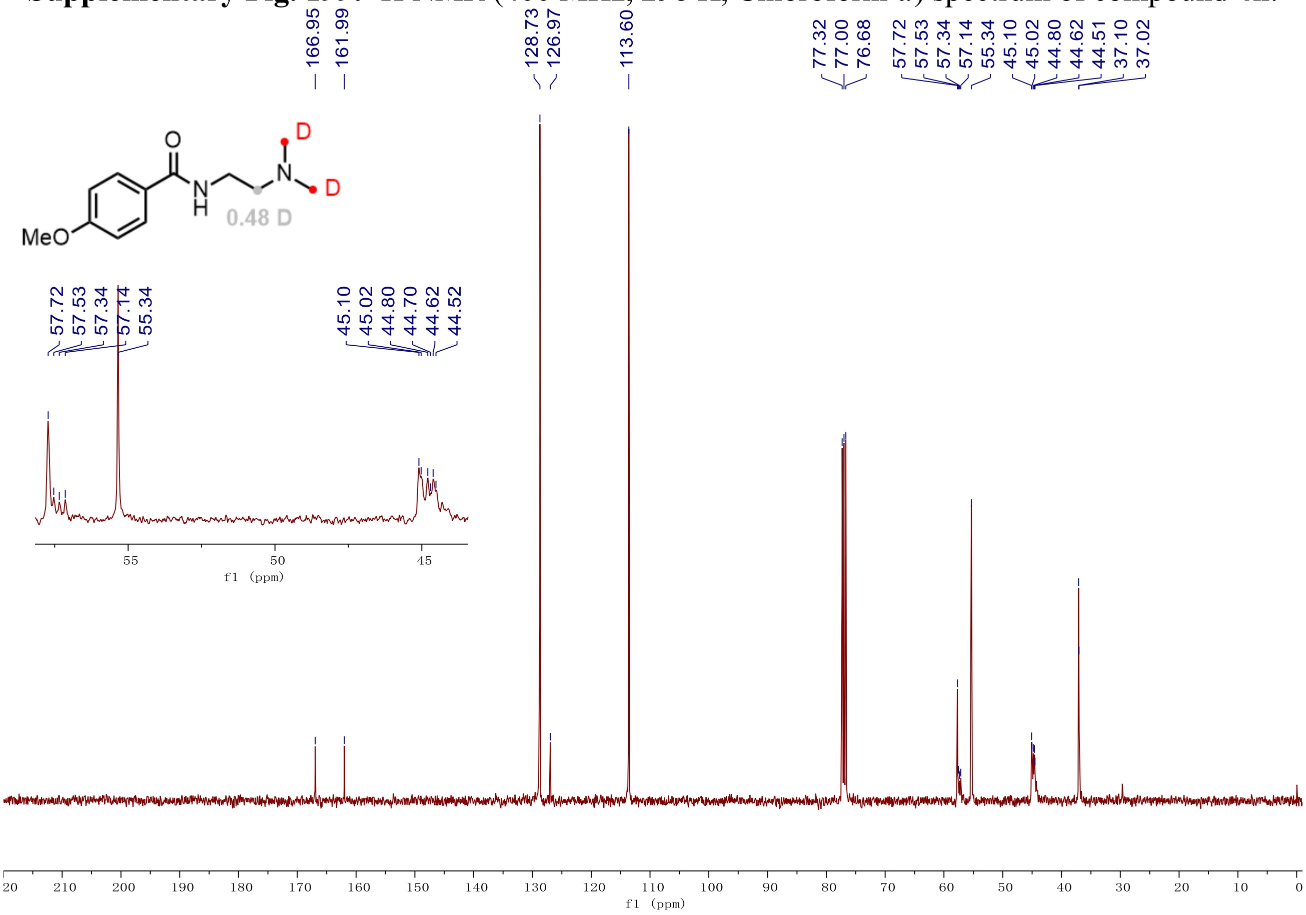

Supplementary Fig. 200. ^{13}C NMR (101 MHz, 298 K, Chloroform-*d*) spectrum of compound **4n**.

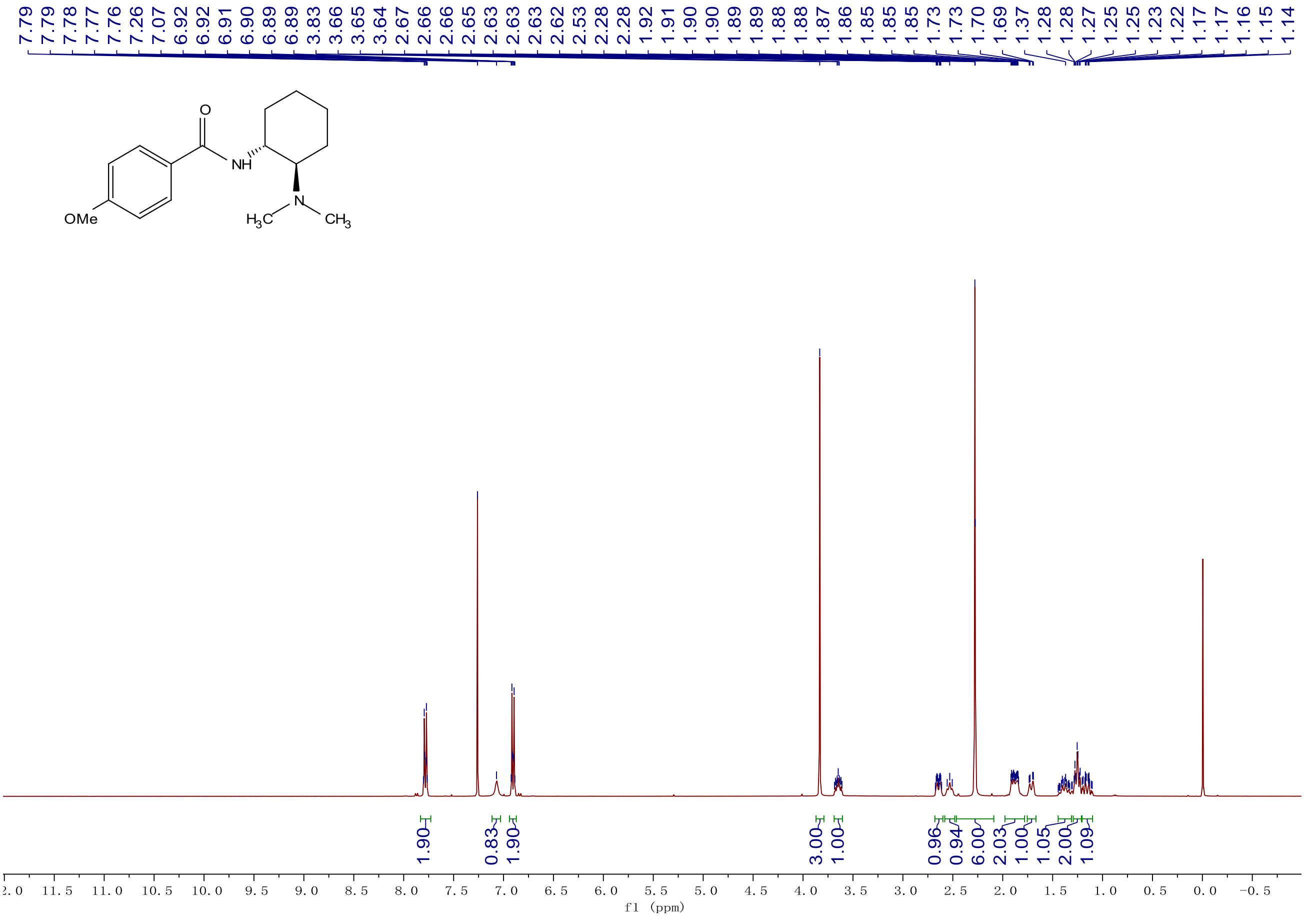

Supplementary Fig. 201. ^{1}H NMR (400 MHz, 298 K, Chloroform-*d*) spectrum of compound **1-o**.

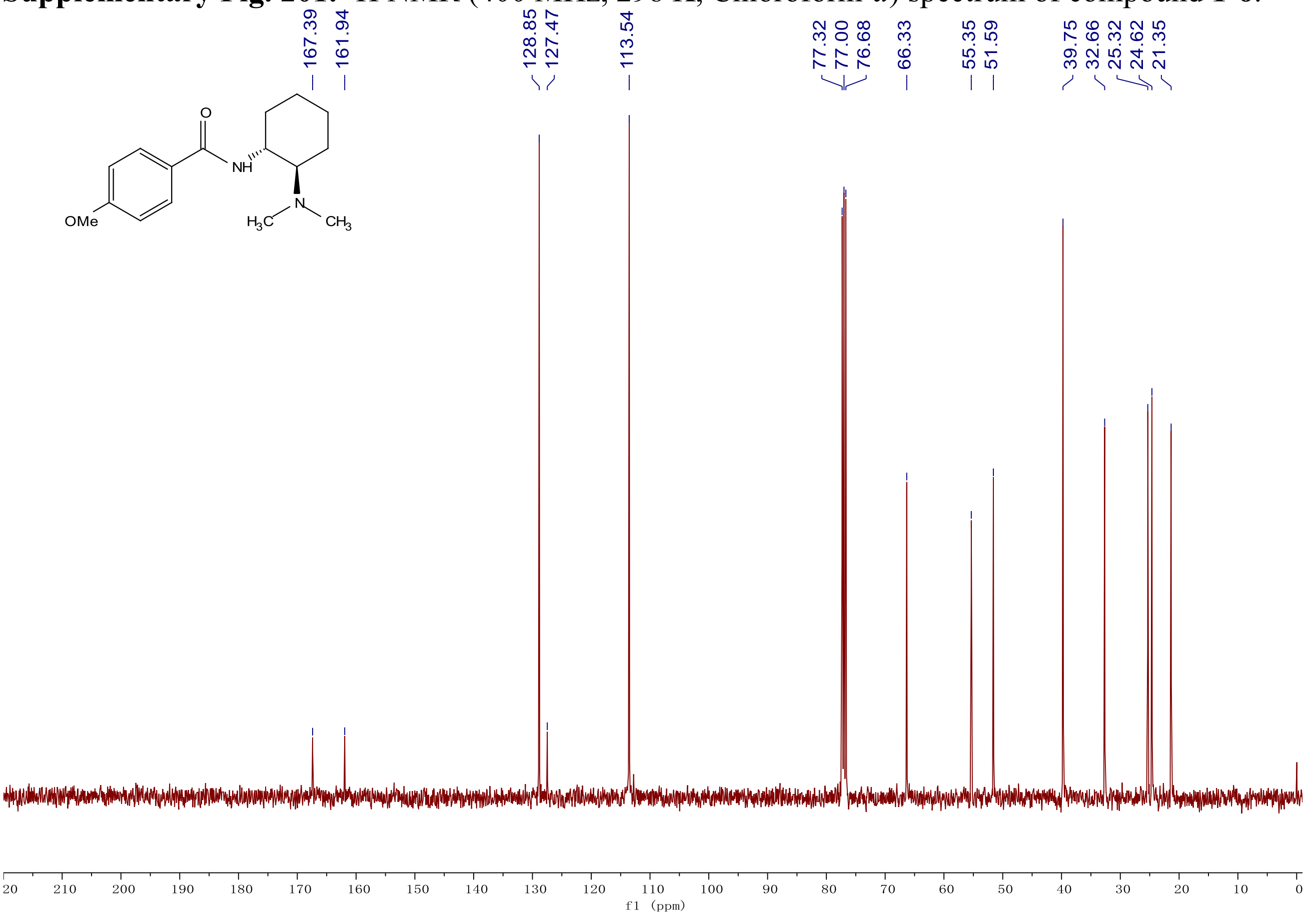

Supplementary Fig. 202. ^{13}C NMR (101 MHz, 298 K, Chloroform-*d*) spectrum of compound **1-o**.

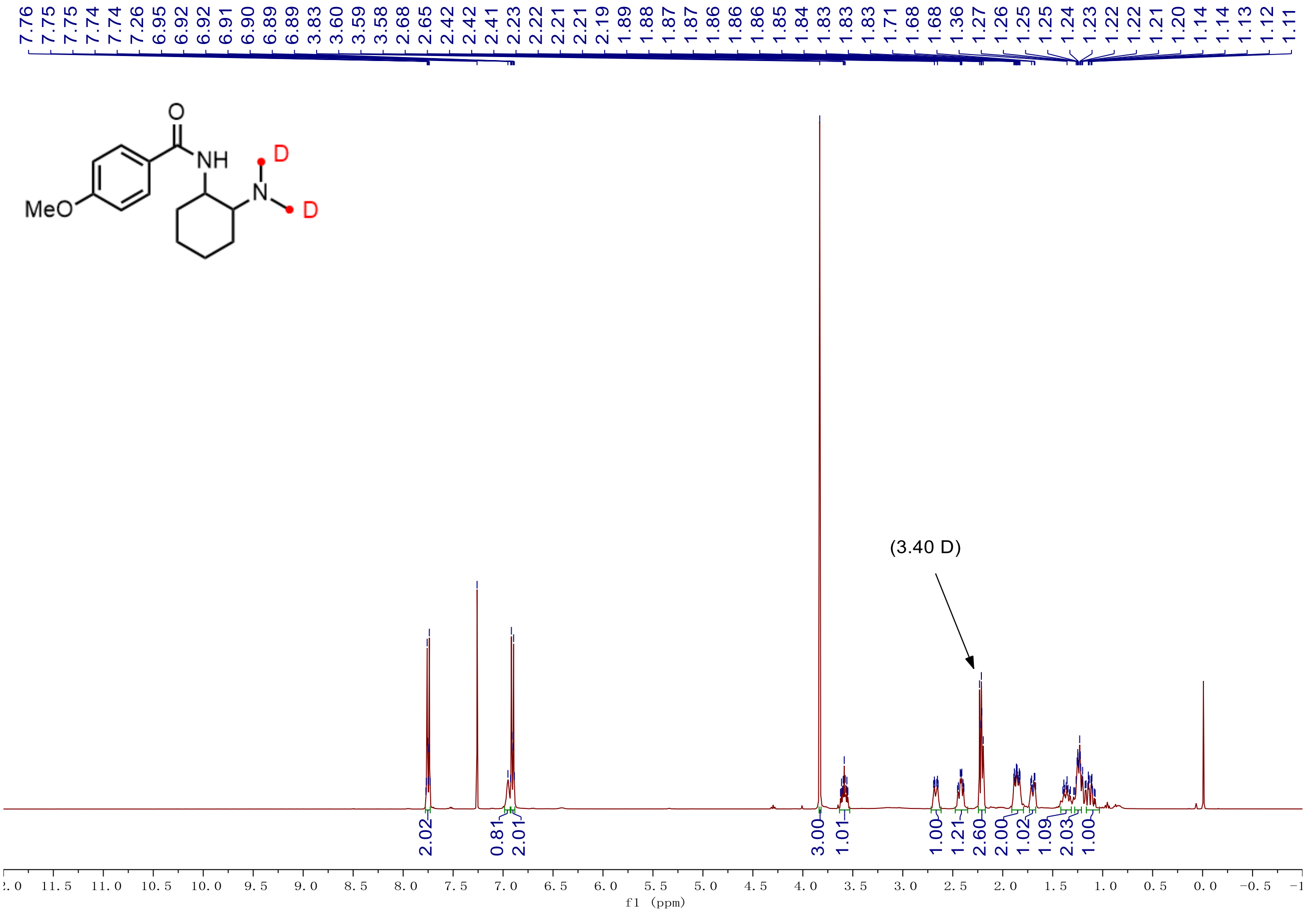

Supplementary Fig. 203. ^{1}H NMR (400 MHz, 298 K, Chloroform-*d*) spectrum of compound **4o**.

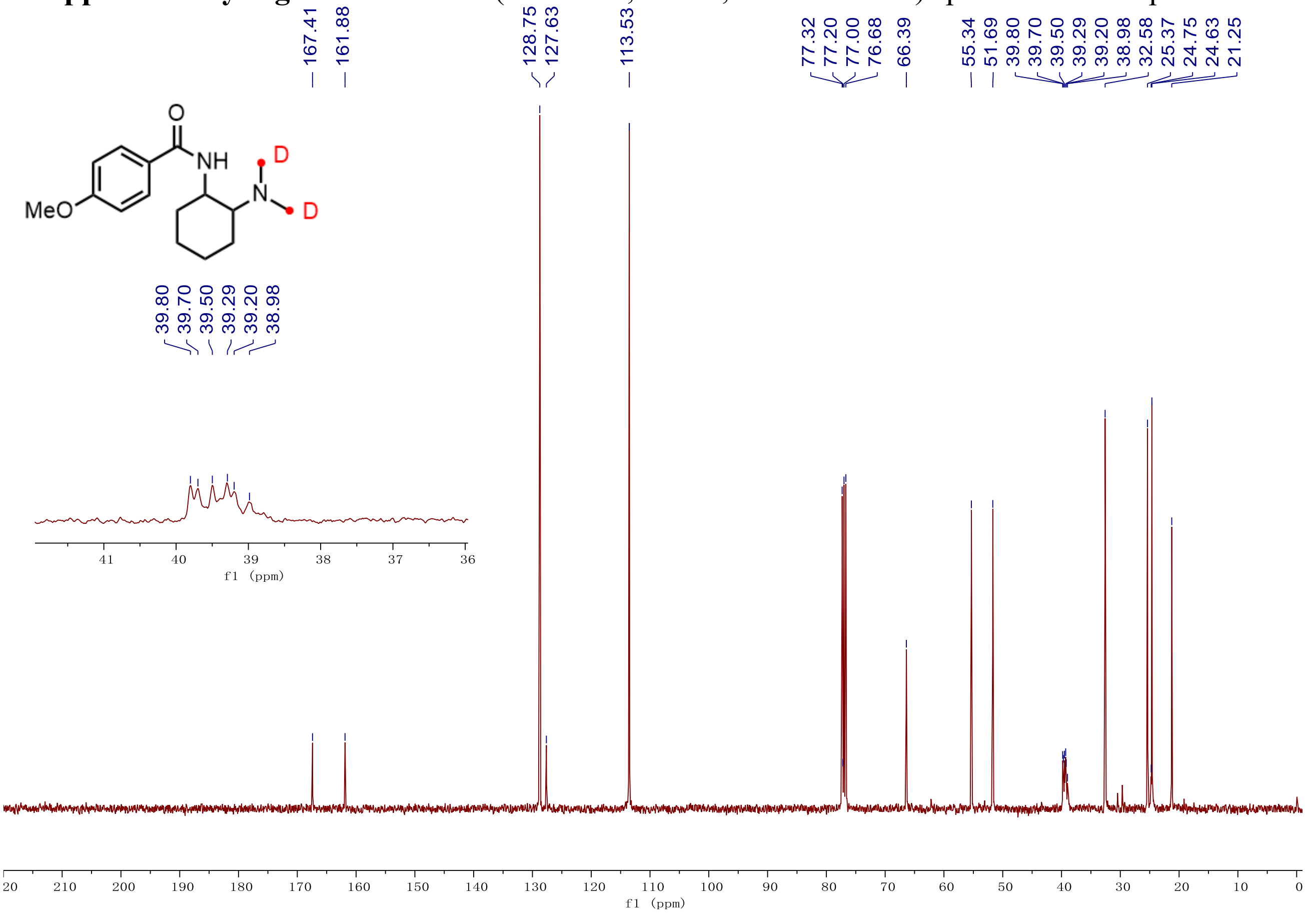

Supplementary Fig. 204. ^{13}C NMR (101 MHz, 298 K, Chloroform-*d*) spectrum of compound **4o**.

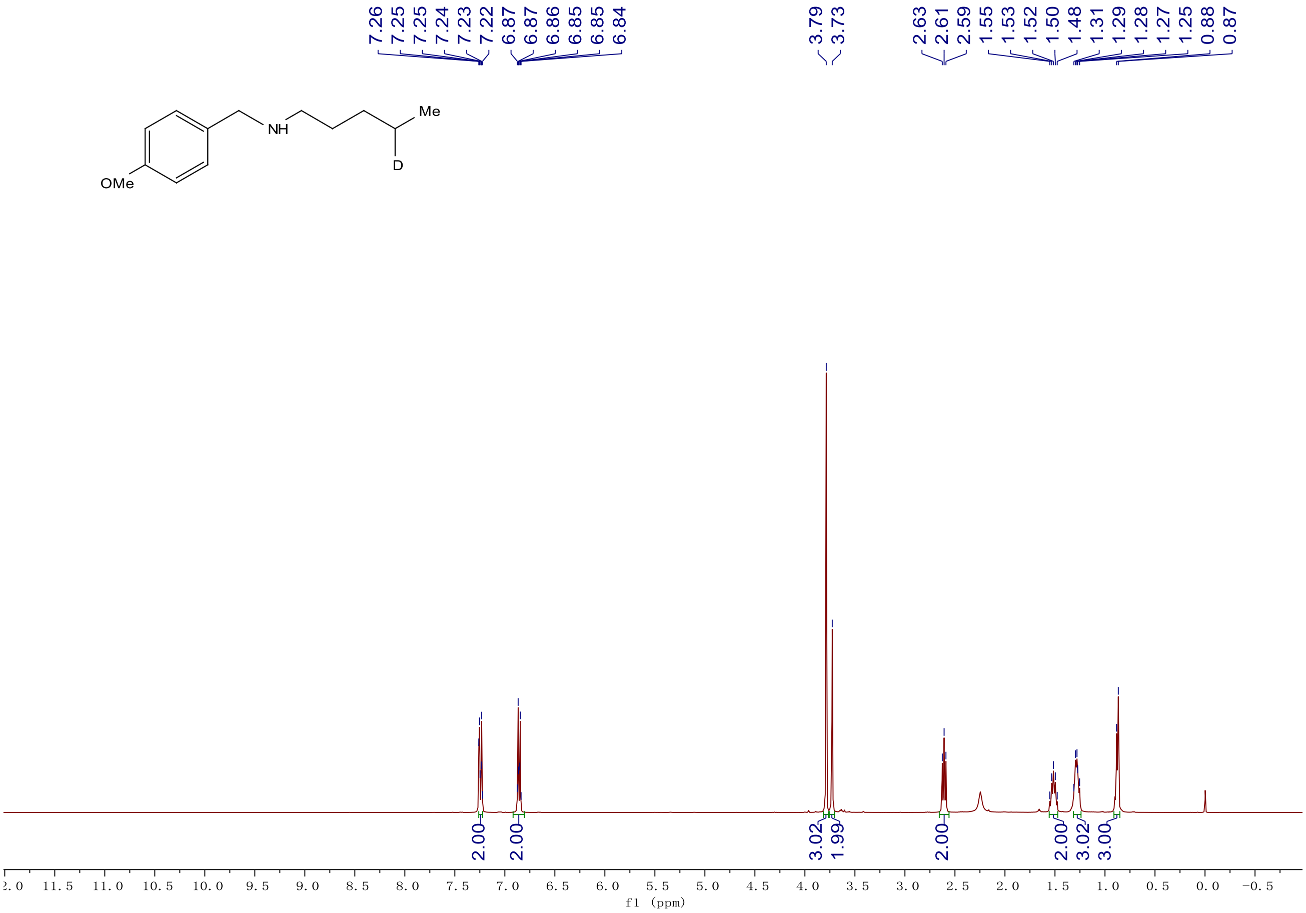

Supplementary Fig. 205. ^{1}H NMR (400 MHz, 298 K, Chloroform-d) spectrum of compound **5**.

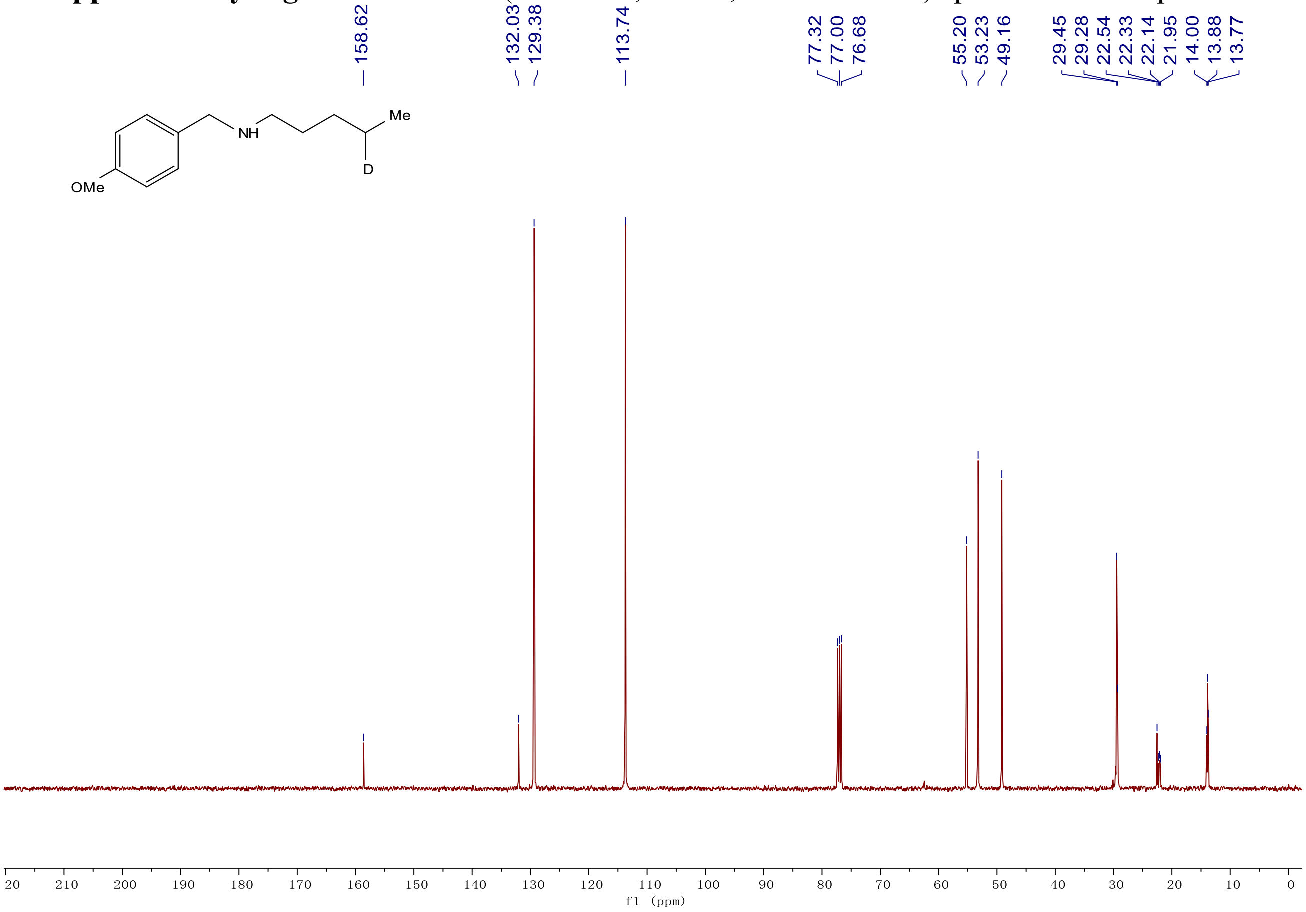

Supplementary Fig. 206. ^{13}C NMR (101 MHz, 298 K, Chloroform-d) spectrum of compound **5**.

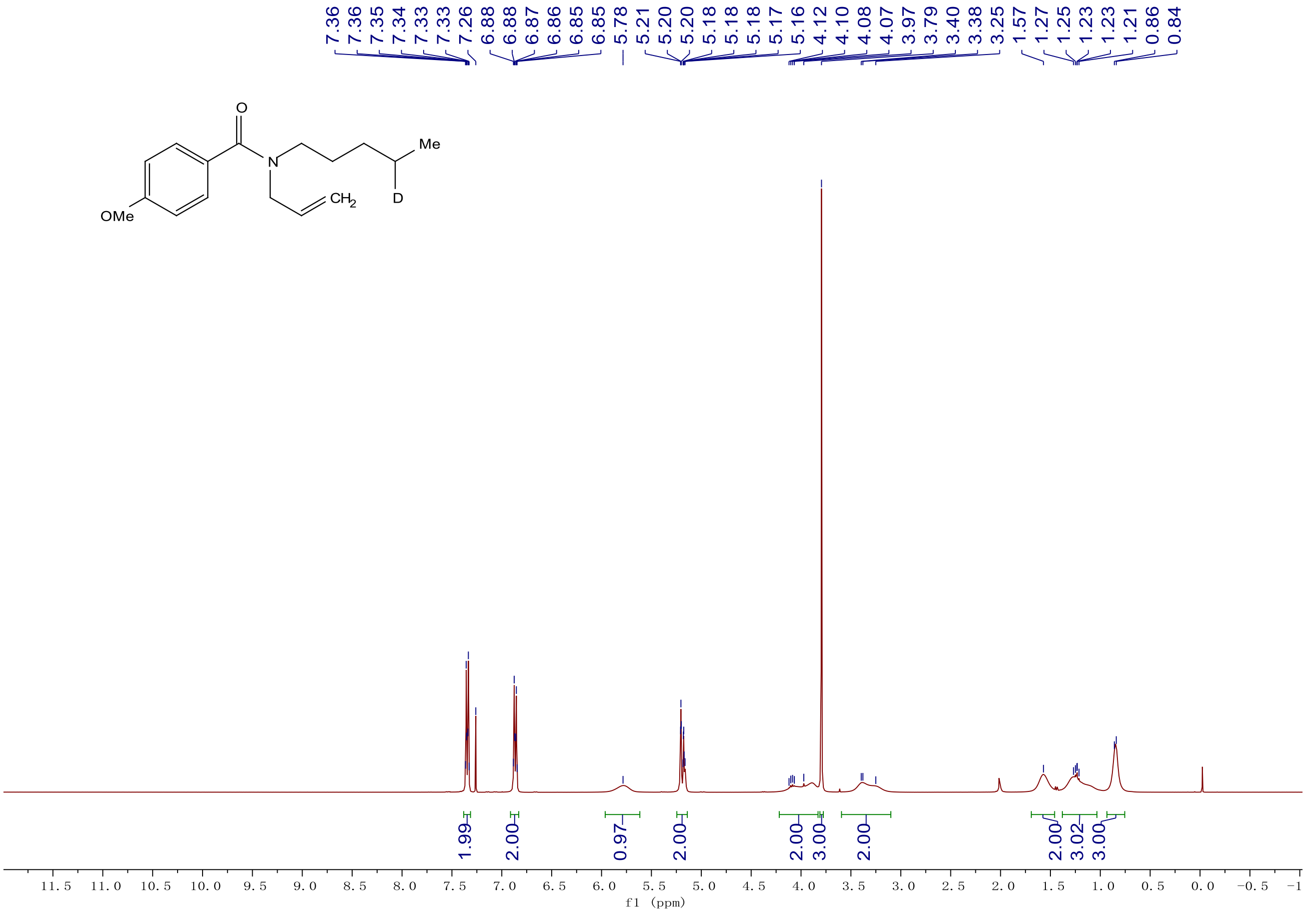

Supplementary Fig. 207. ^{1}H NMR (400 MHz, 298 K, Chloroform-*d*) spectrum of compound **6**.

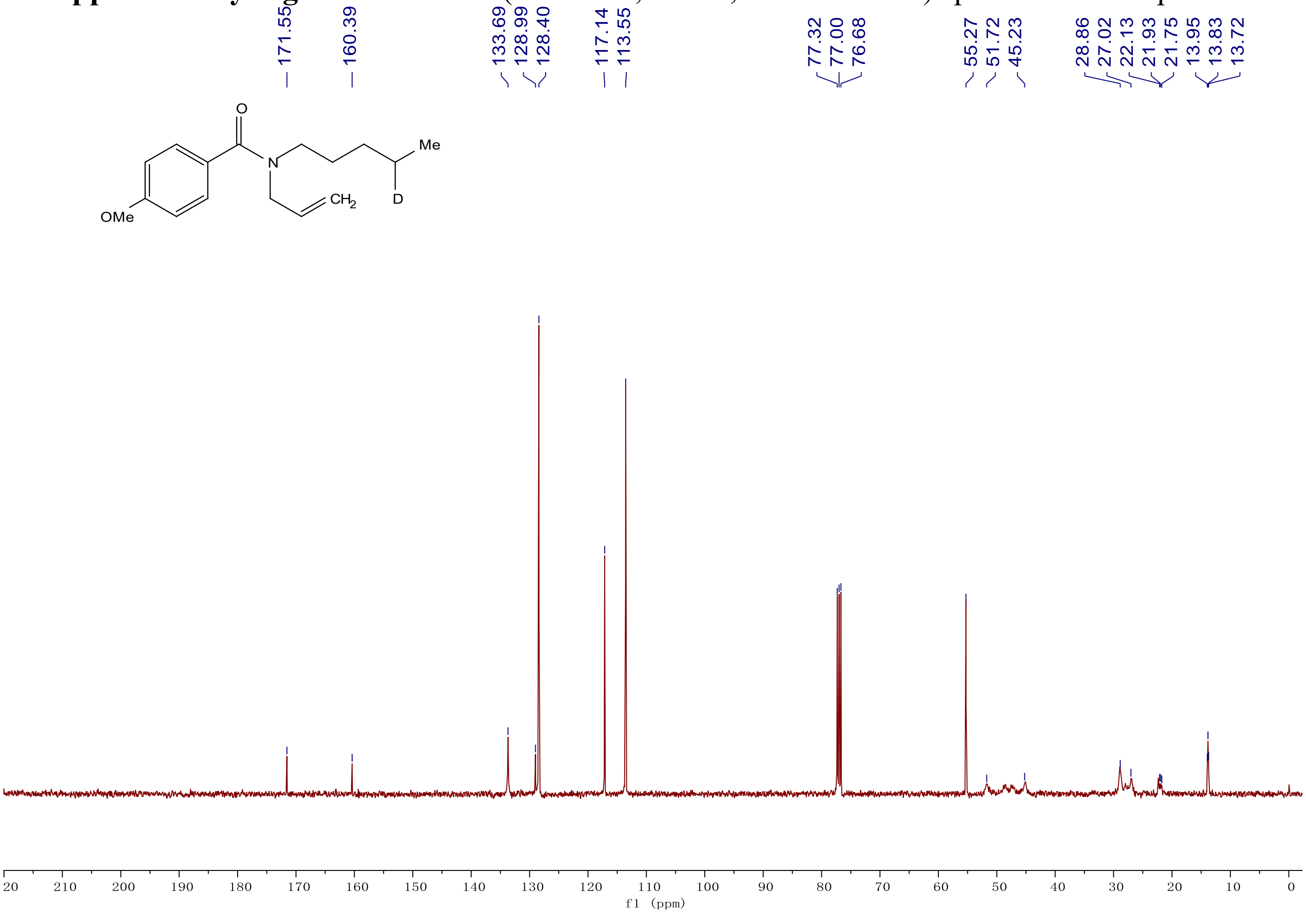

Supplementary Fig. 208. ^{13}C NMR (101 MHz, 298 K, Chloroform-*d*) spectrum of compound **6**.

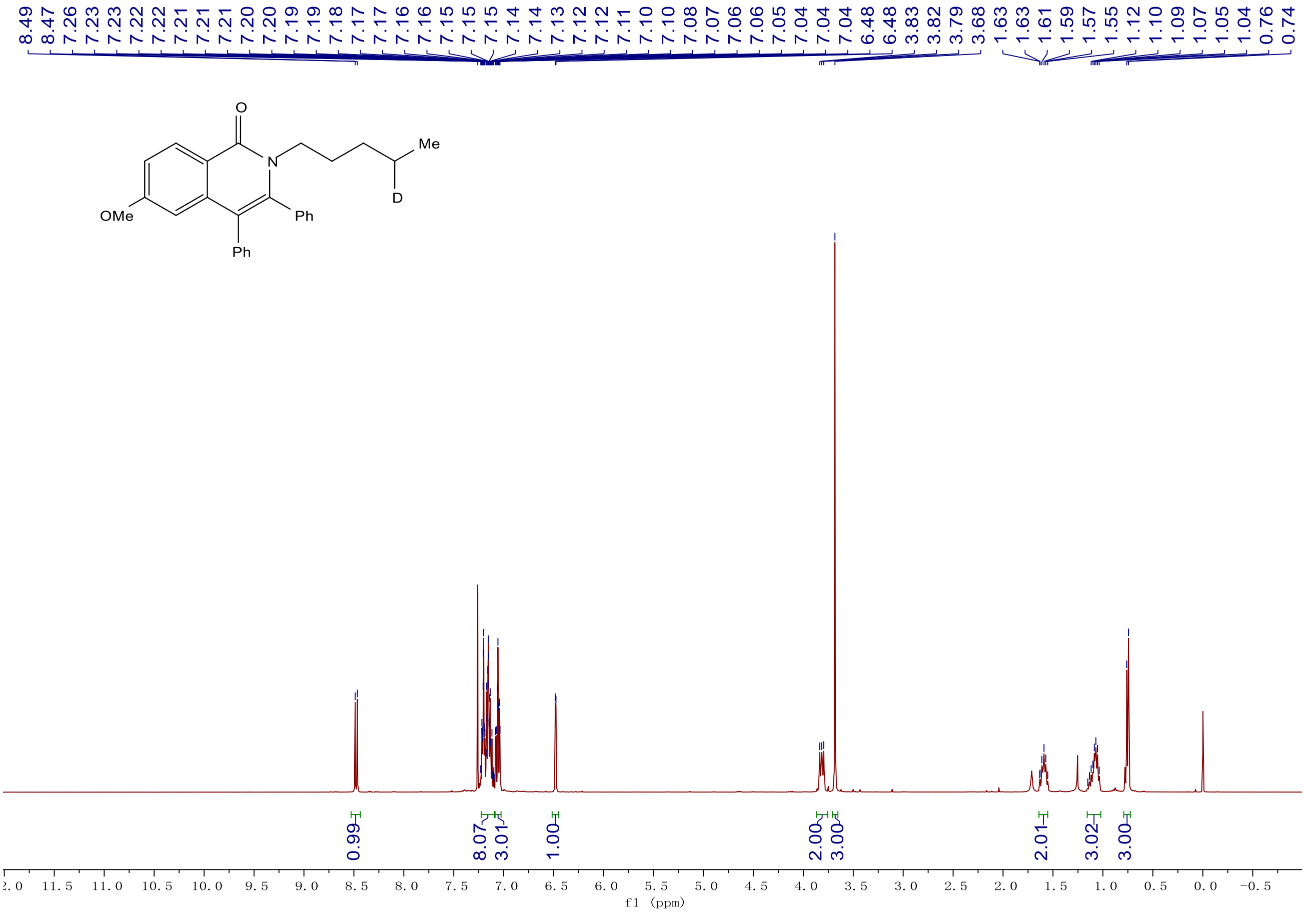

Supplementary Fig. 209. ^{1}H NMR (400 MHz, 298 K, Chloroform-*d*) spectrum of compound **7**.

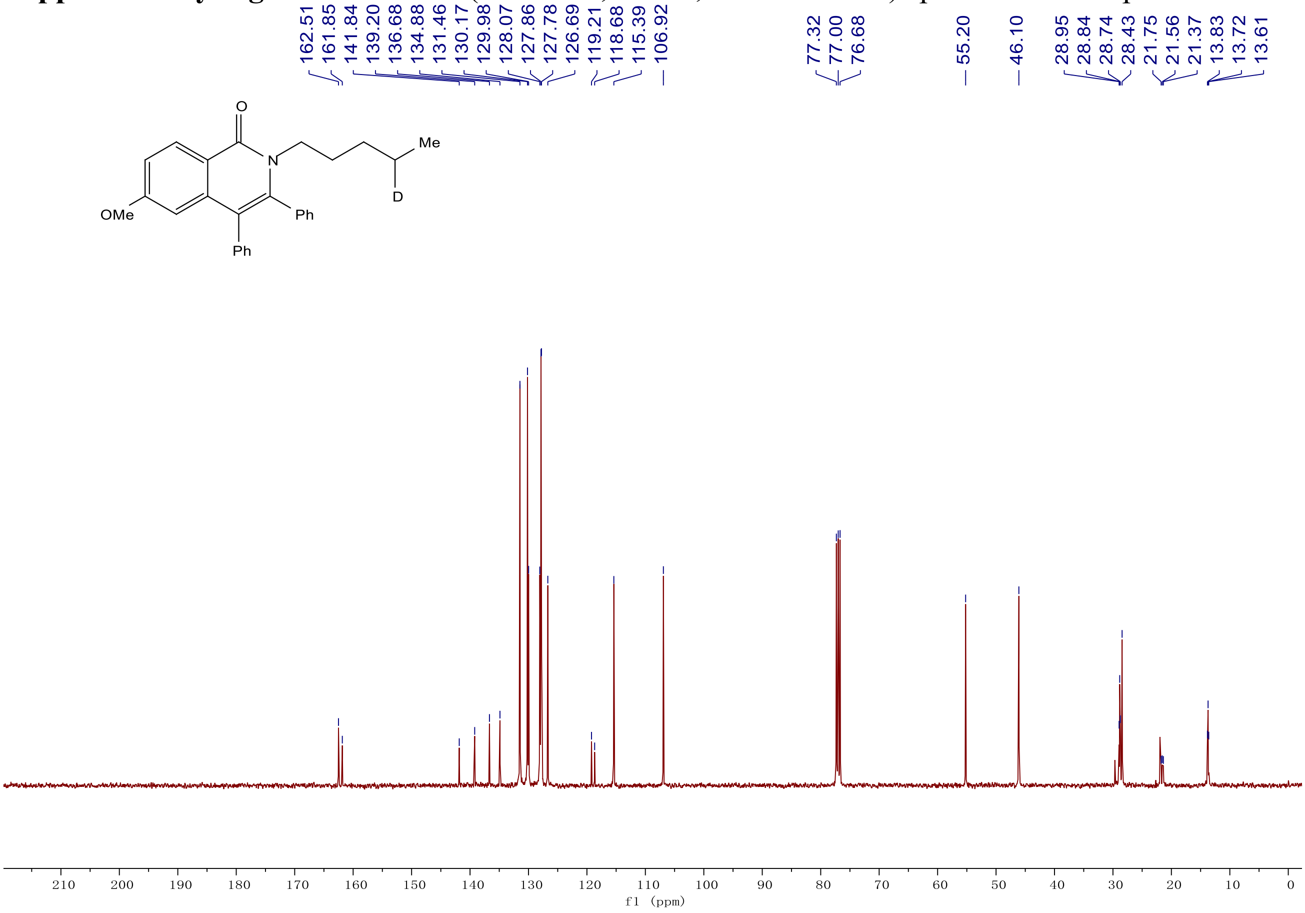

Supplementary Fig. 210. ^{13}C NMR (101 MHz, 298 K, Chloroform-*d*) spectrum of compound **7**.

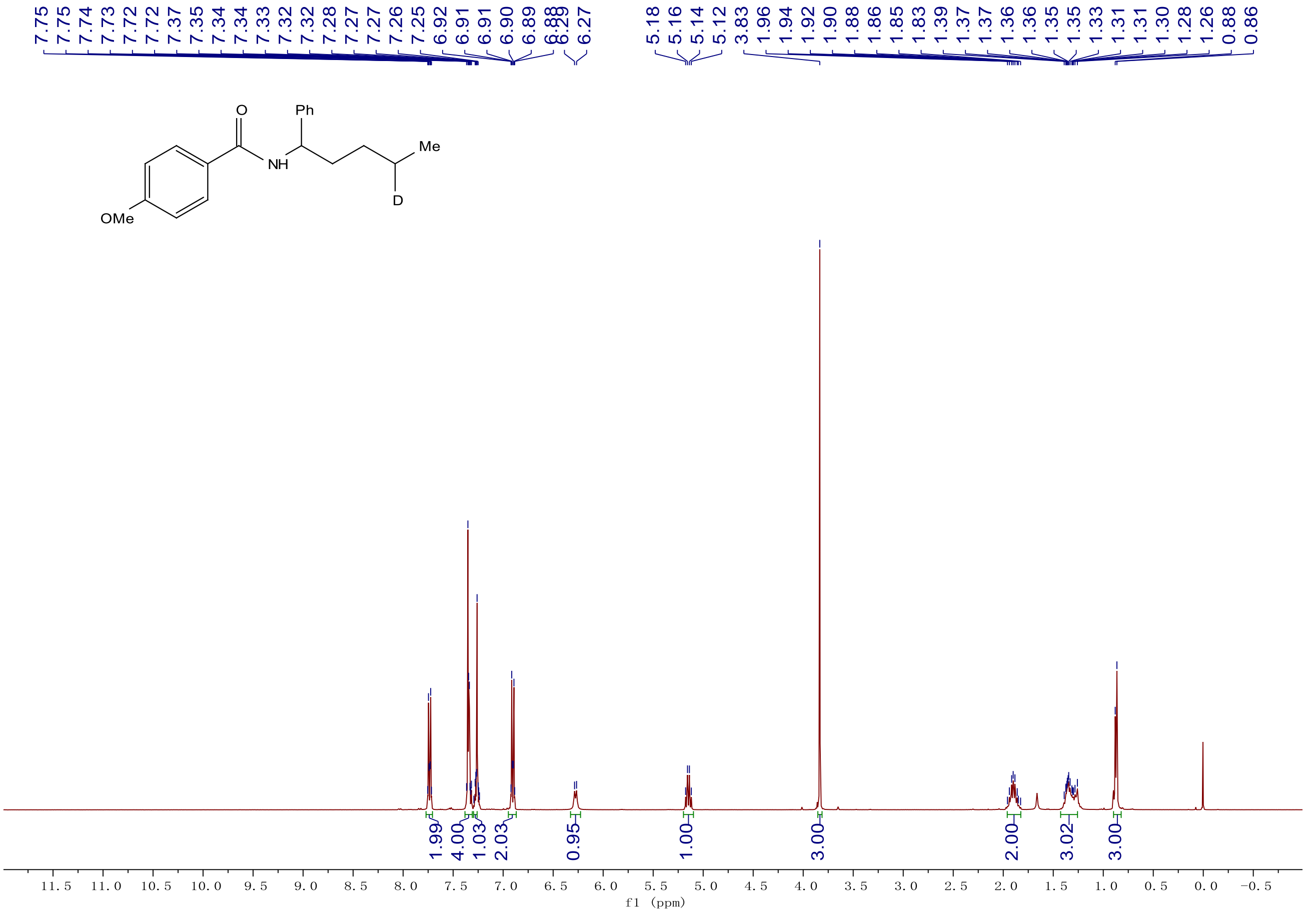

Supplementary Fig. 211. ^{1}H NMR (400 MHz, 298 K, Chloroform-*d*) spectrum of compound **8**.

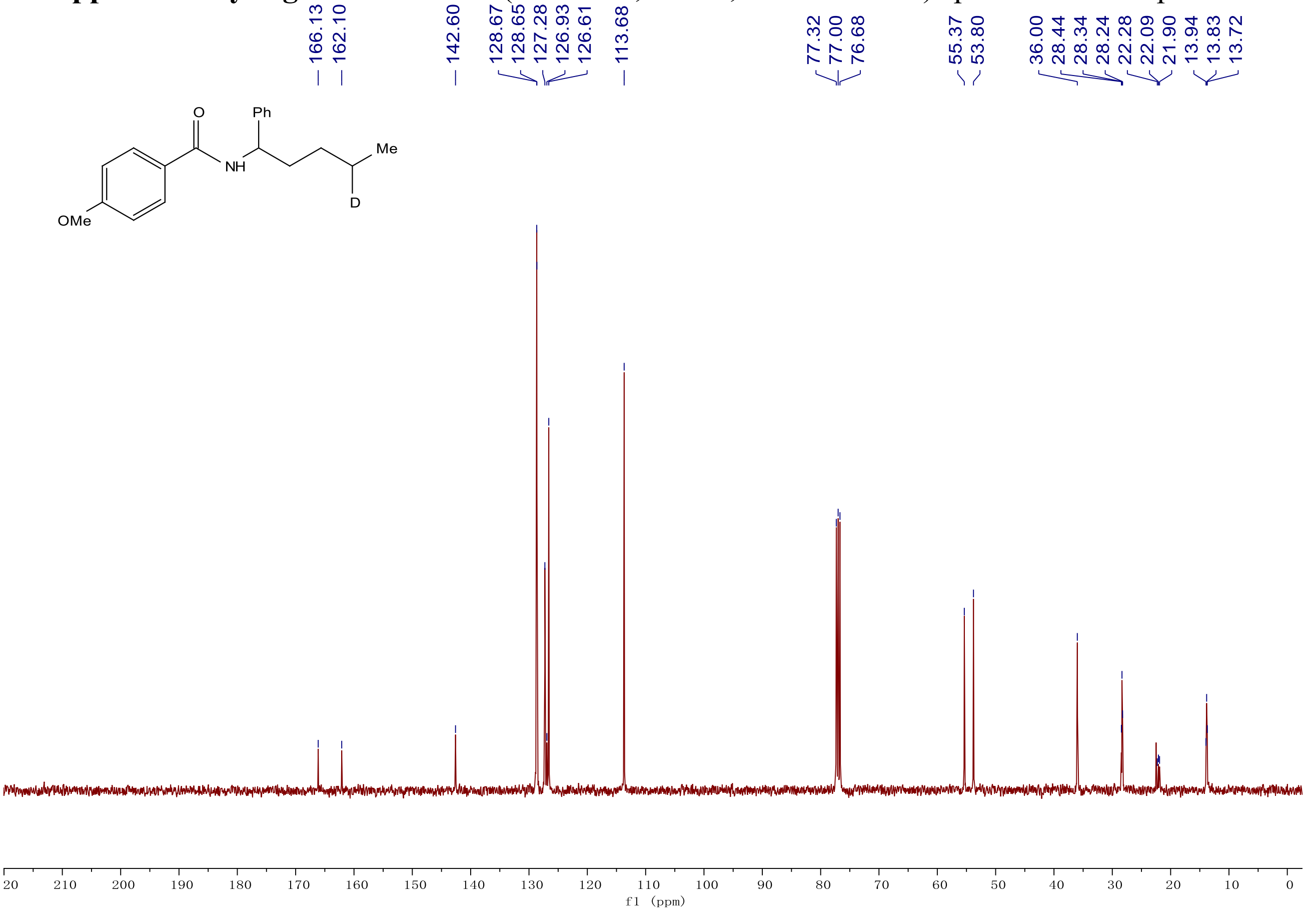

Supplementary Fig. 212. ^{13}C NMR (101 MHz, 298 K, Chloroform-*d*) spectrum of compound **8**.

Supplementary References

1 G. Kundu, T. Sperger, K. Rissanen, F. Schoenebeck, *Angew. Chem. Int. Ed.* **2020**, *59*, 21930.

2 T. K. Hyster, T. Ro126is, *J. Am. Chem. Soc.* **2010**, *132*, 10565.

3 A. W. Rand, H. Yin, L. Xu, J. Giacoboni, R. Martin-Montero, C. Romano, J. Montgomery, R. Martin, *ACS Catal.* **2020**, *10*, 4671.

4 Gaussian 16, Revision C.01, M. J. Frisch, G. W. Trucks, H. B. Schlegel, G. E. Scuseria, M. A. Robb, J. R. Cheeseman, G. Scalmani, V. Barone, G. A. Petersson, H. Nakatsuji, X. Li, M. Caricato, A. V. Marenich, J. Bloino, B. G. Janesko, R. Gomperts, B. Mennucci, H. P. Hratchian, J. V. Ortiz, A. F. Izmaylov, J. L. Sonnenberg, D. Williams-Young, F. Ding, F. Lipparini, F. Egidi, J. Goings, B. Peng, A. Petrone, T. Henderson, D. Ranasinghe, V. G. Zakrzewski, J. Gao, N. Rega, G. Zheng, W. Liang, M. Hada, M. Ehara, K. Toyota, R. Fukuda, J. Hasegawa, M. Ishida, T. Nakajima, Y. Honda, O. Kitao, H. Nakai, T. Vreven, K. Throssell, J. A. Montgomery, Jr., J. E. Peralta, F. Ogliaro, M. J. Bearpark, J. J. Heyd, E. N. Brothers, K. N. Kudin, V. N. Staroverov, T. A. Keith, R. Kobayashi, J. Normand, K. Raghavachari, A. P. Rendell, J. C. Burant, S. S. Iyengar, J. Tomasi, M. Cossi, J. M. Millam, M. Klene, C. Adamo, R. Cammi, J. W. Ochterski, R. L. Martin, K. Morokuma, O. Farkas, J. B. Foresman, and D. J. Fox, Gaussian, Inc., Wallingford CT, **2016**.

5 Y. Zhao, D. G. Truhlar, *Theor. Chem. Acc.*, **2008**, *120*, 215.

6 CYLview, 1.0b, C. Y .Legault, Université de Sherbrooke, **2009.**

7 G. J. Choi1, Q. Zhu, D. C. miller, C. J. Gu1, R. R. Knowles, *Nature*, **2016**, *539*, 268.

8 W. Yuan, Z. Zhou, L. Gong, E. Meggers, *Chem. Commun.*, **2017**,*53*, 8964.

9 J. C. K. Chu, T. Rovis, *Nature*, **2016**, *539*, 272.

10 T. Kajitani, S. Kohmoto, M. Yamamoto, K. Kishikawa, *J. Mater. Chem.*, **2004**,*14*, 3449.

11 C. D. Campbell, N. Duguet, K. A. Gallagher, J. E. Thomson, A. G. Lindsay, A. C. O'Donoghueb, A. D. Smith, *Chem. Commun.*, **2008**, *30*, 3528.

12 D. T. Racys, S. A. I. Sharif, S. L. Pimlott, A. Sutherland, *J. Org. Chem.* **2016**, *81*, 772.

13 A. L. Bodnar, L. A. Cortes-Burgos, K. K. Cook, D. M. Dinh, V. E. Groppi, M. Hajos, N. R. Higdon, W. E. Hoffmann, R. S. Hurst, J. K. Myers, B. N. Rogers, T. M. Wall, M. L. Wolfe, E. Wong, *J. Med. Chem.* **2005**, *48*, 905.

14 B. V. Cheney, J. Szmuszkovicz, R. A. Lahti, D. A. Zichi, *J. Med. Chem.* **1985**, *28*, 1853.
